# Copper-Catalyzed Enantioselective Intra- and Intermolecular Desymmetrization of Azetidiniums by Carbon Nucleophiles

**DOI:** 10.1021/jacs.5c17131

**Published:** 2025-11-21

**Authors:** Minghui Zhu, Chaoshen Zhang, Jianwei Sun

**Affiliations:** † Jiangsu Key Laboratory of Advanced Catalytic Materials & Technology, School of Petrochemical Engineering, Changzhou University, Changzhou 213164, China; ‡ Department of Chemistry and the Hong Kong Branch of Chinese National Engineering Research Centre for Tissue Restoration & Reconstruction, 58207 The Hong Kong University of Science and Technology, Clear Water Bay, Kowloon, Hong Kong SAR 999077, China

## Abstract

Disclosed here is a transition-metal-catalyzed enantioselective ring-opening of azetidiniums. It also represents the first demonstration of catalytic enantioselective intramolecular desymmetrization of prochiral azetidiniums. With a cheap copper salt as the catalyst and a commercially available ferrocene-derived chiral oxazoline-phosphine as the chiral ligand, a mild borylation/cyclization cascade provided convenient access to diverse densely functionalized tetrahydropyrans and piperidine compounds bearing an α-methylene unit from readily available enone-tethered azetidinium substrates. Three new stereogenic centers and two new bonds were concomitantly constructed together with the ring formation, which also represents an unorthodox disconnection strategy in the related heterocycle synthesis. Control experiments suggested that borylation served as the enantiodetermining step. Moreover, this protocol was also extended to intermolecular C­(sp^3^)–C­(sp^3^) formation between azomethine ylides and azetidiniums, thereby providing expedient access to the valuable ornithine derivatives with high enantioselectivity. DFT studies also provided insights into the enantio-determining transition states.

## Graphical abstract

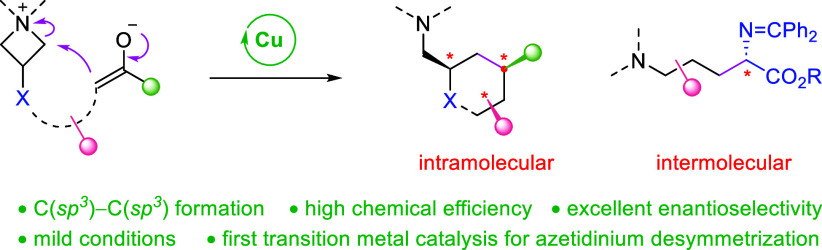

## Introduction

Azetidiniums are a family of versatile building blocks in organic synthesis.^1–4^ Owing to the ring strain and the strong polarization of the C–N bond, they exhibit high propensity toward ring-opening upon reacting with nucleophiles, thus permitting the rapid installation of a three-carbon fragment with a remote amine functionality. While such reactivity has been well established in achiral or racemic synthesis,^2^ the enantioselective opening of the prochiral 3-substituted azetidiniums induced by chiral catalysts has only been achieved very recently and still remains in its infancy (Scheme 1a).^3^ In 2018, we reported the first example using a type of mercaptobenzothiazole nucleophiles catalyzed by chiral phosphoric acids.^3a^ Subsequently, Gouverneur and co-workers pioneered the use of hydrogen bond-donor catalysis for the enantioselective opening of azetidiniums by fluoride.^3b^ More recently, Jacobsen and co-workers made further advances by developing an elegant charge recognition approach for the enantioselective opening of *in situ* formed azetidiniums by chloride and bromide.^4^ Other than organocatalysis, Mg-based Lewis acid catalysis also proved useful for this type of process, as pioneered by Lin, Feng, and co-workers.^3c^ However, despite this notable progress, there remains an unmet need for additional effective catalytic protocols in view of the broad utility of such transformations in rapid access to highly functionalized chiral amines. For example, approaches based on powerful transition-metal catalysis have remained unexplored. Moreover, an intramolecular variant that can provide convenient access to functionalized heterocycles has not been demonstrated before.

**Scheme 1:**
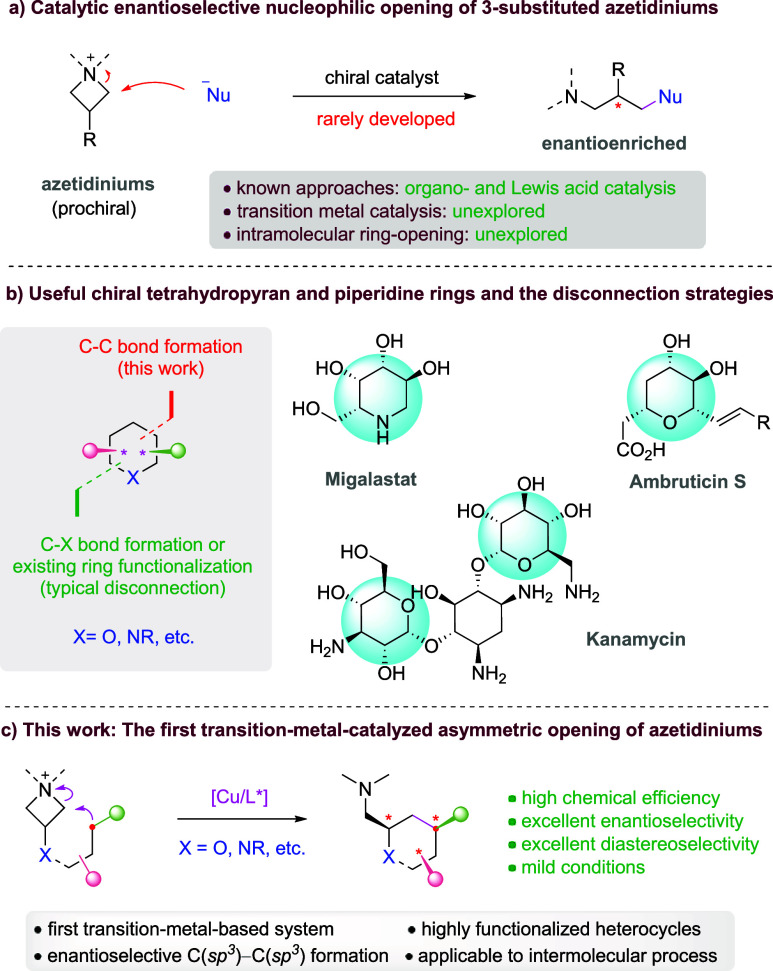
Background of Enantioselective Opening of Azetidiniums and Reaction Design

Chiral-functionalized tetrahydropyrans and piperidines are prevalent units in a wide array of natural products and pharmaceutical agents, such as kanamycin, ambruticin S, and migalastat (Scheme 1b).^5,6^ Notably, the tetrahydropyran and piperidine rings in these molecules not only have multiple functionalities and chiral centers but also bear a functionalized methylene substituent (e.g., hydroxymethyl, aminomethyl) in the α-position, making such heterocycles less straightforward to construct in an expedient and enantioselective manner. Typically, such ring structures and their stereocenters as well as functionalities are constructed/established in separate steps,^7^ which often involve carbon-heteroatom or carbon-functionality bond formation. Herein, we report an unorthodox but exceptionally convenient approach featuring remote C–C bond formation to construct such a heterocycle with concomitant installation of multiple functional groups and stereogenic centers (Scheme 1b).

In continuation of our efforts in the enantioselective desymmetrization of strained rings,^8^ we envisioned that the prochiral azetidinium unit can potentially undergo intramolecular ring-opening by an internal carbon nucleophile linked by a heteroatom (Scheme 1c). With a suitable *in situ* generation of a well-positioned organocopper nucleophile, together with a chiral ligand, it was expected to form an intramolecular C­(sp^3^)–C­(sp^3^) bond with good efficiency and stereoselectivity. Here, we report our progress in this design, which represents the first transition-metal-catalyzed enantio- and diastereoselective example, as well as the first intramolecular nucleophilic opening of azetidiniums, which can also be extended to the intermolecular variant for the synthesis of medicinally valuable ornithine derivatives.

## Results and Discussion

### Intramolecular Reaction

We started our exploration by employing azetidinium **1a** tethered with an enone motif by an ether linker, which was easily synthesized as a mixture of *cis*- and *trans*-isomers regarding the relative configuration of substituents on the azetidinium ring (Table 1). We hypothesized that a copper-catalyzed borylative conjugate addition to the enone motif should be able to generate a copper enolate,^9^ which is well positioned for the intramolecular ring-opening of the azetidinium and thus leads to highly functionalized tetrahydropyrans bearing an adjacent aminomethyl group. In addition to the C–B and C–C bond formation, three nonconsecutive stereogenic centers should be constructed simultaneously, hopefully with the absolute and relative configurations to be controlled by a suitable chiral ligand.

**Table 1:**
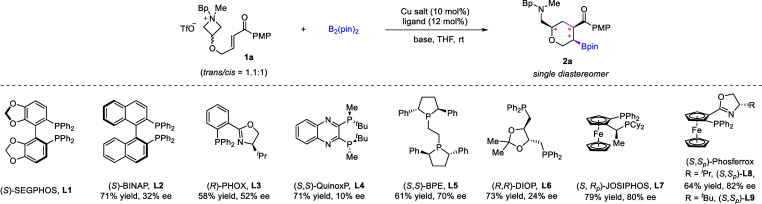
Optimization of Reaction Conditions^a^

entry	Cu salt	ligand	base	additive	yield (%)^b^	ee (%)^c^
1	Cu(CH_3_CN)_4_PF_6_	**L1**	NaO^*t*^Bu		trace	
2	Cu(CH_3_CN)_4_PF_6_	**L1**	Na_2_CO_3_		trace	
3	Cu(CH_3_CN)_4_PF_6_	**L1**	Cs_2_CO_3_		29	42
4	Cu(CH_3_CN)_4_PF_6_	**L2-L8**	Cs_2_CO_3_		58–79	10–82
5	Cu(CH_3_CN)_4_PF_6_	**L9**	Cs_2_CO_3_		70	84
6	Cu(CH_3_CN)_4_BF_4_	**L9**	Cs_2_CO_3_		72	86
7	CuBr	**L9**	Cs_2_CO_3_		55	84
8	CuCl	**L9**	Cs_2_CO_3_		65	84
9	Cu(TFA)_2_·H_2_O	**L9**	Cs_2_CO_3_		69	88
10	Cu(TFA)_2_·H_2_O	**L9**	Cs_2_CO_3_	MeOH	72	88
11	Cu(TFA)_2_·H_2_O	**L9**	Cs_2_CO_3_	^*t*^BuOH	75	88
12^d^	Cu(TFA)_2_·H_2_O	**L9**	Cs_2_CO_3_	^*t*^BuOH	78	88
13^d^ ^,^ ^e^	Cu(TFA)_2_·H_2_O	**L9**	Cs_2_CO_3_	^*t*^BuOH	76	92
14^d^ ^,^ ^e^ ^,^ ^f^	Cu(TFA)_2_·H_2_O	**L9**	Cs_2_CO_3_	^*t*^BuOH	80	92
15^d^ ^,^ ^e^ ^,^ ^g^	Cu(TFA)_2_·H_2_O	**L9**	Cs_2_CO_3_	^*t*^BuOH	72	92

a Reaction conditions: **1a** (0.05 mmol), B_2_pin_2_ (0.06 mmol), Cu salt (10 mol %), ligand (12 mol %), base (2.0 equiv), additive (2.0 equiv), THF (0.25 mL), rt, 12 h.

b Determined by^1^H NMR spectroscopy of the crude product with 1,2-dibromoethane as an internal standard.

c Determined by chiral HPLC.

d **1a** (0.06 mmol), B_2_pin_2_ (0.05 mmol).

e –40 °C to rt.

f Pure *cis*-**1a** was used.

g Pure *trans*-**1a** was used. dr > 20:1. Bp = Ph_2_CH, PMP = *p*-methoxyphenyl.

The initial study was performed with Cu­(CH_3_CN)_4_PF_6_ as the catalyst, (*S*)-SEGPHOS **L1** as the chiral ligand, and B_2_pin_2_ as the boron source. Different bases were examined (Table 1, entries 1–3). It is worth noting that the base is crucial to this reaction. Strong bases may lead to the decomposition of the azetidinium substrate, but weak bases may not be able to promote this reaction. While ^*t*^BuONa and Na_2_CO_3_ as well as other bases (e.g., MeONa, Et_3_N, DIPEA, DBU) proved ineffective, Cs_2_CO_3_ promoted this process to afford the desired tetrahydropyran **2a**. Although the yield was low, it was encouraging to observe promising enantioselectivity and excellent diastereoselectivity (entry 3, 29% yield, 42% ee, single diastereomer). Next, various commercially available chiral phosphine ligands, including monodentate and bidentate ones, were examined (entries 4–5). Among them, ligands with a ferrocene backbone provided relatively higher enantioselectivity, with the bidentate phosphine-oxazoline ligand (*S*,*S*_*p*_)-**L9** performing best (entry 5). Other copper salts were also examined (entries 6–9), revealing that both Cu­(I) and Cu­(II) catalysts were catalytically active, providing almost the same outcome. Cu­(TFA)_2_·H_2_O was chosen for further study in view of its low cost. To further enhance the yield, several proton sources were tested (entries 10–11). It was found that the addition of ^*t*^BuOH slightly improved the yield (entry 11), and its beneficial role was more significant with sterically hindered substrates bearing 3,3-disubstitution (*vide infra*). Finally, decreasing the initial reaction temperature to −40 °C allowed further improvement of the enantioselectivity (92% ee). The use of slightly excess **1a** (vs B_2_Pin_2_) maintained the good yield. We also performed control experiments using pure *cis*-**1a** or *trans*-**1a** (entries 14–15). In fact, both isomers separately reacted with essentially the same efficiency and enantioselectivity, suggesting that the azetidinium configuration does not influence the outcome. It is worth noting that this reaction not only generated the densely functionalized tetrahydropyran ring by sequential C­(sp^3^)–B and C­(sp^3^)–C­(sp^3^) bond formation but also concomitantly established three stereogenic centers with complete diastereocontrol.

With the optimized conditions, we next investigated the reaction scope (Scheme 2). A wide variety of azetidiniums bearing different substituents on the ring or the ketone motif reacted smoothly to yield the desired chiral-substituted tetrahydropyrans with good to excellent diastereoselectivity and enantioselectivity. Of note, some of the substrates are mixtures of *cis*- and *trans*-stereoisomers relative to the azetidinium ring. The absolute configuration of **2b** was confirmed by X-ray crystallography. The electronic property of the aryl ketone motif does not have an obvious impact on the reactivity. Furthermore, azetidiniums (**1j**–**1o**) bearing various substituents at the 3-position, including both aryl and alkyl groups, underwent smooth reactions toward the desired products, thus generating a tetrasubstituted carbon stereogenic center. Notably, the use of an alcohol additive significantly enhanced the yield of those bearing a quaternary stereogenic center (see more details in the Supporting Information). We believe that the additive may help to tune the basicity of the reaction system and facilitate the turnover of the catalytic cycle. Finally, we also evaluated the possibility of applying this protocol for the synthesis of chiral piperidines. Indeed, by replacing the ether linker to the protected amine linker in the substrate, the same protocol could be successfully applied in the convenient synthesis of densely functionalized piperidine **2p** in high yield and with excellent enantioselectivity. Notably, these substituents can be easily converted to other functionalities, which would permit facile access to other diverse valuable building blocks.

**Scheme 2:**
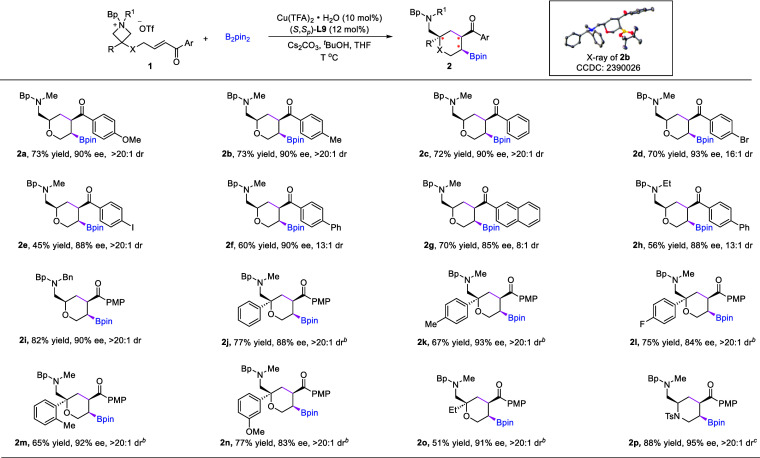
Reaction Scope of the Intramolecular Reaction^a^

a Reaction conditions: **1** (0.24 mmol), B_2_pin_2_ (0.2 mmol), Cu­(TFA)_2_·H_2_O (10 mol %), (*S*,*S*_*p*_)-**L9** (12 mol %), Cs_2_CO_3_ (0.4 mmol), ^*t*^BuOH (0.4 mmol), THF (2.0 mL), −40 °C to rt.
b –40 to 40 °C.
c Cu­(TFA)_2_·H_2_O (20 mol %), (*S*,*S*_*p*_)-**L9** (24 mol %). Isolated yield. The ee value was determined by chiral HPLC analysis. The dr value was determined by ^1^H NMR analysis of the crude product.

In addition to the synthesis of tetrahydropyran and piperidine rings, we further explored a borylation/oxidation/cyclization cascade under slightly modified conditions (Scheme 3). In the absence of a base, the standard reaction mixture of *trans*-**1b** was treated with the oxidant NaBO_3_. Interestingly, substituted 1,4-dioxane **3** was obtained in 62% yield and 86% ee with good diastereoselectivity. This process likely proceeded via initial conjugate addition to generate the formal hydroboration product followed by oxidation to β-hydroxyl ketone **IM-3**, which then underwent an intramolecular opening of the azetidinium ring with the hydroxy nucleophile.

**Scheme 3:**
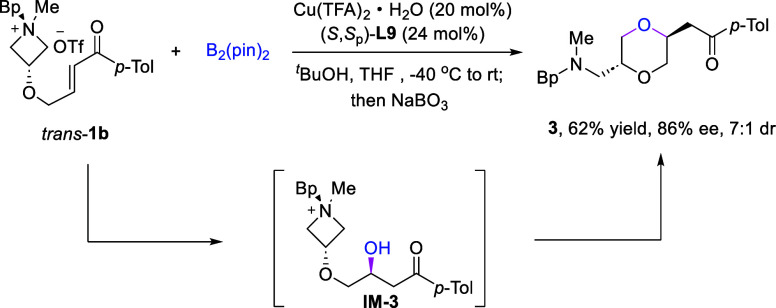
One-Pot Sequential Borylation/Oxidation/Cyclization

### Intermolecular Reaction

Inspired by the intramolecular desymmetrization process, we continued to explore the possibility of extending this protocol to an intermolecular variant by suitable copper enolate nucleophiles. Indeed, copper-bound azomethine ylides represent a type of highly versatile and robust carbon nucleophiles in organic synthesis.^10^ Wang and others have pioneered the ingenious use of such species for the synthesis of a broad range of highly enantioenriched α-amino acid derivatives with suitable metal catalytic systems.^11^ While various electrophiles have been demonstrated as useful reaction partners, the use of strained rings as electrophiles has remained underexplored. To further enhance the application of both azomethine ylides and azetidiniums, we envisioned intermolecular C­(sp^3^)–C­(sp^3^) bond formation between the two versatile species. To our delight, after the comprehensive evaluation of the reaction parameters (see details in the SI), we found that, with the combined use of CuBr (10 mol %), ligand (*S*,*S*_*p*_)-**L9** (12 mol %), and Cs_2_CO_3_ (2.0 equiv), the reaction between **4a** and **5a** in PhCF_3_ proceeded successfully at 60 °C with high efficiency and excellent enantioselectivity (Scheme 4a). Notably, the resulting product **6a**, a chiral ornithine derivative, represents a type of useful molecules in medicinal chemistry (Scheme 4b).^12^

**Scheme 4:**
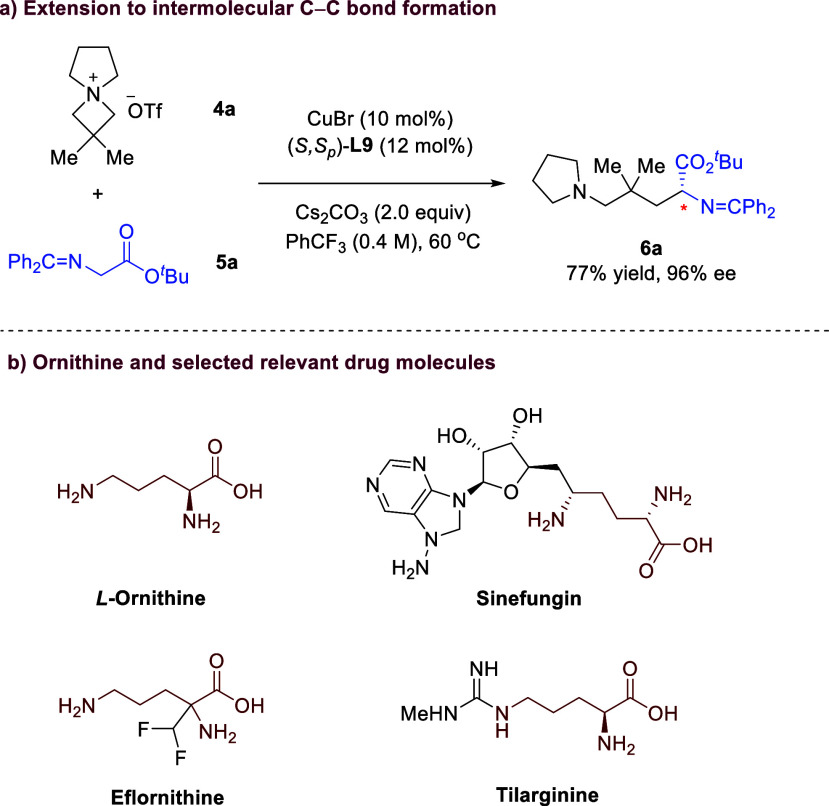
Intermolecular Reaction for the Synthesis of Ornithine Derivatives

a Reaction conditions: **4a** (0.3 mmol), **5** (0.45 mmol), CuBr (10 mol %), (*S*,*S*_*p*_)-**L9** (12 mol %), Cs_2_CO_3_ (2.0 equiv), PhCF_3_ (0.75 mL), 60 °C, 72 h. Isolated yield. The ee value was determined by chiral HPLC analysis.

This protocol was applicable to different azetidiniums **4** and imine esters **5** (Scheme 5). Azetidiniums bearing different substituents on the nitrogen atom, in either a cyclic or acyclic setting, all produced the corresponding ornithine derivatives with good to excellent efficiency and enantioselectivity. Notably, substitution with a benzyl group, an easily removable *N*-protective group, also led to good results. Moreover, azetidiniums bearing different substituents in the 3-position, including spirobicyclic azetidiniums **4d** and **4e**, also reacted smoothly to give the desired products. Heterocycles, such as thiophene, morpholine, tetrahydroisoquinoline, were successfully incorporated into the reaction products with good enantiocontrol. When the azetidinium ring was monosubstituted at the 3-position, high yield and enantioselectivity were obtained, albeit with moderate diastereoselectivity (**6q**–**6t**). Subsequently, replacing the ^*t*^Bu group in the ester motif by Me, Et, and ^*i*^Pr groups also afforded the desired products with good levels of enantioselectivity (**6u**–**6w**). Finally, we examined the effect of the imine moiety. The imine derived from benzophenone bearing two *para*-Cl groups gave low efficiency and enantioselectivity. However, the use of *para*-OMe groups resulted in both an excellent yield and enantioselectivity (**6y**, 82% yield, 99% ee). Since this moiety plays a similar role as a protective group, one can choose a suitable imine motif for the synthesis when needed.

**Scheme 5:**
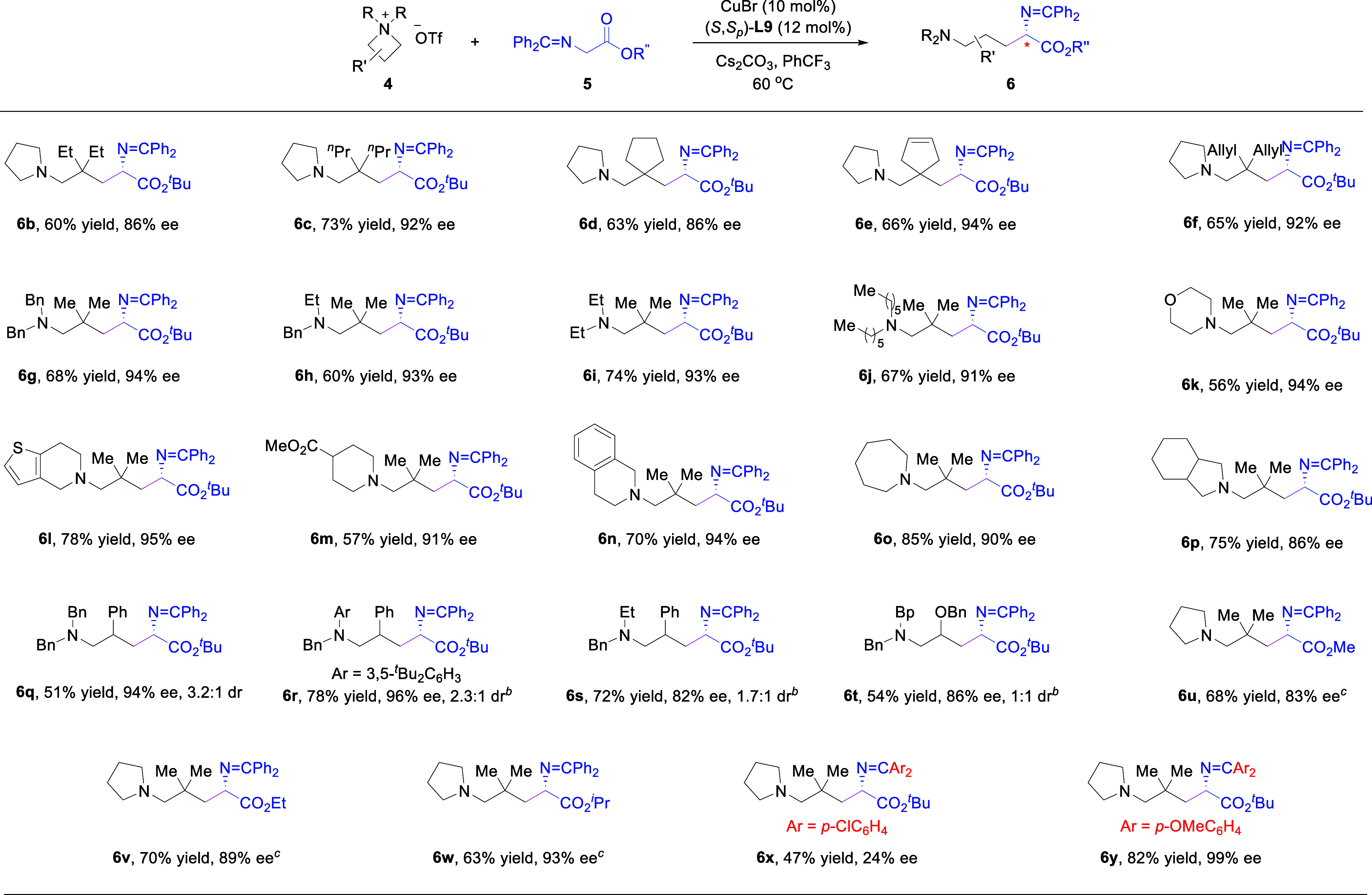
Reaction Scope of the Intermolecular Reaction with Azomethine Ylides^a^

a Reaction conditions: **4** (0.3 mmol), **5** (0.45 mmol), CuBr (10 mol %), (*S*,*S*_*p*_)**-L9** (12 mol %), Cs_2_CO_3_ (0.6 mmol), PhCF_3_ (0.75 mL), 60 °C.
b CuBr (20 mol %), (*S*,*S*_*p*_)-**L9**.
c
**4a** (0.1 mmol), **5** (0.15 mmol), CuBr (20 mol %), (*S*,*S*_*p*_)-**L9** (24 mol %). Isolated yield. The ee value was determined by chiral HPLC analysis.

To further showcase the synthetic utility of the above processes, a 1 mmol scale synthesis of tetrahydropyran **2i** was performed, and high yield and excellent stereoselectivity were obtained. The potassium trifluoroborate salt **7** was readily obtained by stirring **2i** with KHF_2_. Additionally, treatment of **2i** with NaBO_3_ provided secondary alcohol **8** in 87% yield.

Some derivatizations of intermolecular products **6g** and **6h** were also demonstrated. The imine motif could be easily removed under weakly acidic conditions. The resulting free amine functionality was further protected in the form of tosyl amides **9** and **10**, for the sake of purification and selective manipulation of the other amine motif. The absolute configuration of compound **10** was unambiguously confirmed by X-ray crystallography. Next, the removal of the *N*-benzyl protective group would allow the facile modification of the ornithine skeleton of the remote amine functionality. Indeed, under either Pd/C- or Pd­(OH)_2_-catalyzed hydrogenation conditions, the desired deprotection proceeded smoothly to form secondary amine **11** and free primary amine **12**, from two precursors **9** and **10**, respectively. In addition, reduction of ester **10** with LiAlH_4_ led to chiral amino alcohol **13** in good yield. In all of these transformations, no obvious loss of enantiopurity was observed (Scheme 6).

**Scheme 6:**
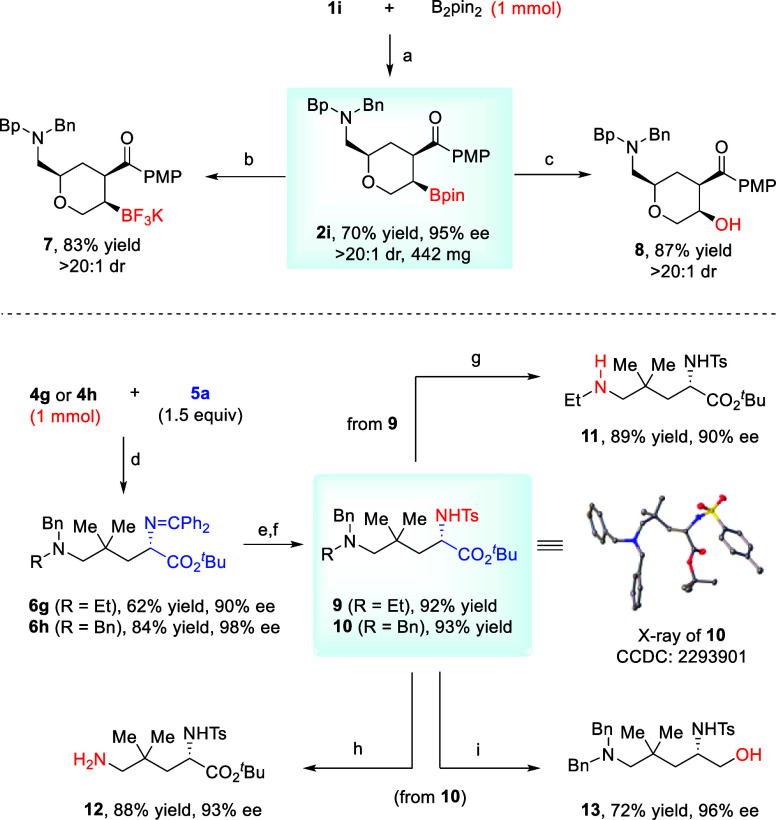
Scale-Up Reactions and Product Transformations^a^

a Reaction conditions: (a) **1i** (1.2 mmol), B_2_pin_2_ (1.0 mmol), Cu­(TFA)_2_·H_2_O (20 mol %), (*S*,*S*_*p*_)-**L9** (24 mol %), Cs_2_CO_3_ (0.6 mmol), ^*t*^BuOH (0.6 mmol), THF (3.0 mL), −40 °C to rt. (b) KHF_2_ (10.0 equiv), MeOH/H_2_O = 10:1, rt. (c) NaBO_3_ (5.0 equiv), THF/H_2_O = 1:1, rt. (d) CuBr (15 mol %), (*S*,*S*_*p*_)-**L9** (18 mol %), Cs_2_CO_3_ (2.0 equiv), PhCF_3_ (0.2 M), 60 °C. (e) Citric acid (10% aq.), THF/H_2_O, rt, 2 h. (f) TsCl, DMAP, DCM, rt, 12 h. (g) Pd/C (10 mol %), H_2_ (1 atm), MeOH, rt, 18 h. (h) Pd­(OH)_2_ (10 mol %), H_2_ (1 atm), MeOH, rt, 18 h. (i) LiAlH_4_, THF, 0 °C to rt, 12 h.

### Mechanism Study

To gain more insights into the reaction mechanism, we performed several control experiments. Enantioenriched β-borylated ketone **INT** (96% ee) was synthesized by an altered sequence of enantioselective borylation (with the same chiral ligand (*S*,*S*_*p*_)-**L9**) and azetidinium formation (see the Supporting Information for details). The reactivity of **INT** was studied under various conditions (Figure 1a). Under the standard conditions (without B_2_pin_2_), it resulted in the successful formation of the enantioenriched product **2k** in 75% yield with no loss of ee (96% ee, >20:1 dr). Next, the same reaction was performed with (*R*,*R*_*p*_)-**L9**, the opposite enantiomeric ligand, which formed **2k** in 65% yield, with a 96% ee, with the absolute configuration observed in the previous reaction. Indeed, even with the racemic ligand, the same result was observed. More surprisingly, even without the copper salt, the same reaction also proceeded to form **2k** without a loss of enantiopurity. These observations suggested that the enantiodetermining step of the standard reaction is likely the conjugate addition. It was the established stereochemistry in the β-position, but not the chiral ligand, that governed the azetidinium ring-opening.

**Figure 1:**
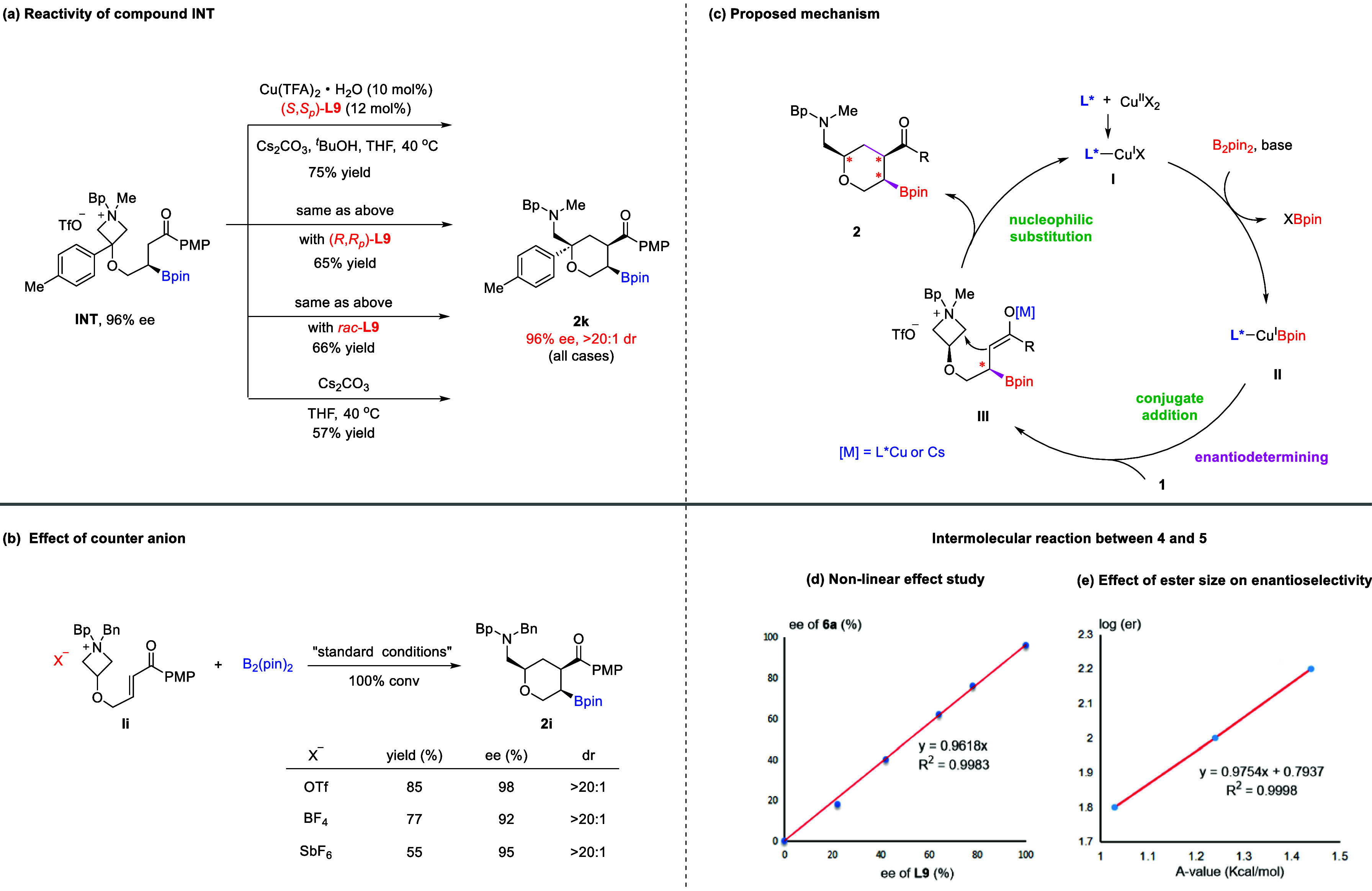
Mechanistic studies. (a) The reactivity of compound **INT** under different conditions. (b) The effect of counteranions. (c) Proposed mechanism. (d) Study of the nonlinear effect. (e) The effect of ester size for the intermolecular process.

Next, we also explored the influence of counteranions in the azetidinium substrate. In fact, among TfO^–^, BF_4_^–^, and SbF_6_^–^, the counteranion showed some effect on reactivity, but almost no impact on enantioselectivity or diastereoselectivity (Figure 1b). Based on the above results and related literature,^13^ a possible mechanism is proposed in Figure 1c. The reaction begins by forming chiral Cu­(I)-ligand complex **I** from the Cu­(II) precatalyst and the phosphine ligand. In the presence of a base, ligand exchange with B_2_pin_2_ occurs to generate active Cu­(I)–Bpin species **II**, a key intermediate for enantioselective conjugate addition to the enone motif of substrate **1**. This is the enantio-determining step, forming enolate **III** with the absolute stereochemistry already established in the β-position. Notably, the metal cation in this enolate intermediate can be either Cu^+^ or Cs^+^. The subsequent intramolecular nucleophilic ring-opening of the azetidinium ring proceeds to furnish observed product **2**, with the new stereogenic center governed by the β-position chirality. Meanwhile, the mechanism of intermolecular ring-opening was also studied. The product ee showed no nonlinear relationship with the catalyst ee values (Figure 1d), suggesting that higher order catalyst aggregates with differential catalytic activity are likely not involved. Moreover, from the results of **6u**–**6w**, plotting log­(er) versus the steric descriptor of the ester substituent showed a linear relationship, which indicated a direct correlation between the size of the ester substituent and the enantiomeric ratio (Figure 1e).

Based on these observations and the previous literature,^11^ possible transition states are proposed, and insights were obtained by density functional theory (DFT) calculations (Figure 2). The calculated enantiodetermining transition states were optimized and characterized in PhCF_3_ with the SMD solvent model (SCRF = SMD) at the B3LYP-D3/6-31G­(d)+SDD­(Cu, Fe)/SMD­(PhCF_3_) level, and single point energies were further calculated at the M06/6-311++G­(d,p)+SDD­(Cu, Fe)/SMD­(PhCF_3_) level.

**Figure 2:**
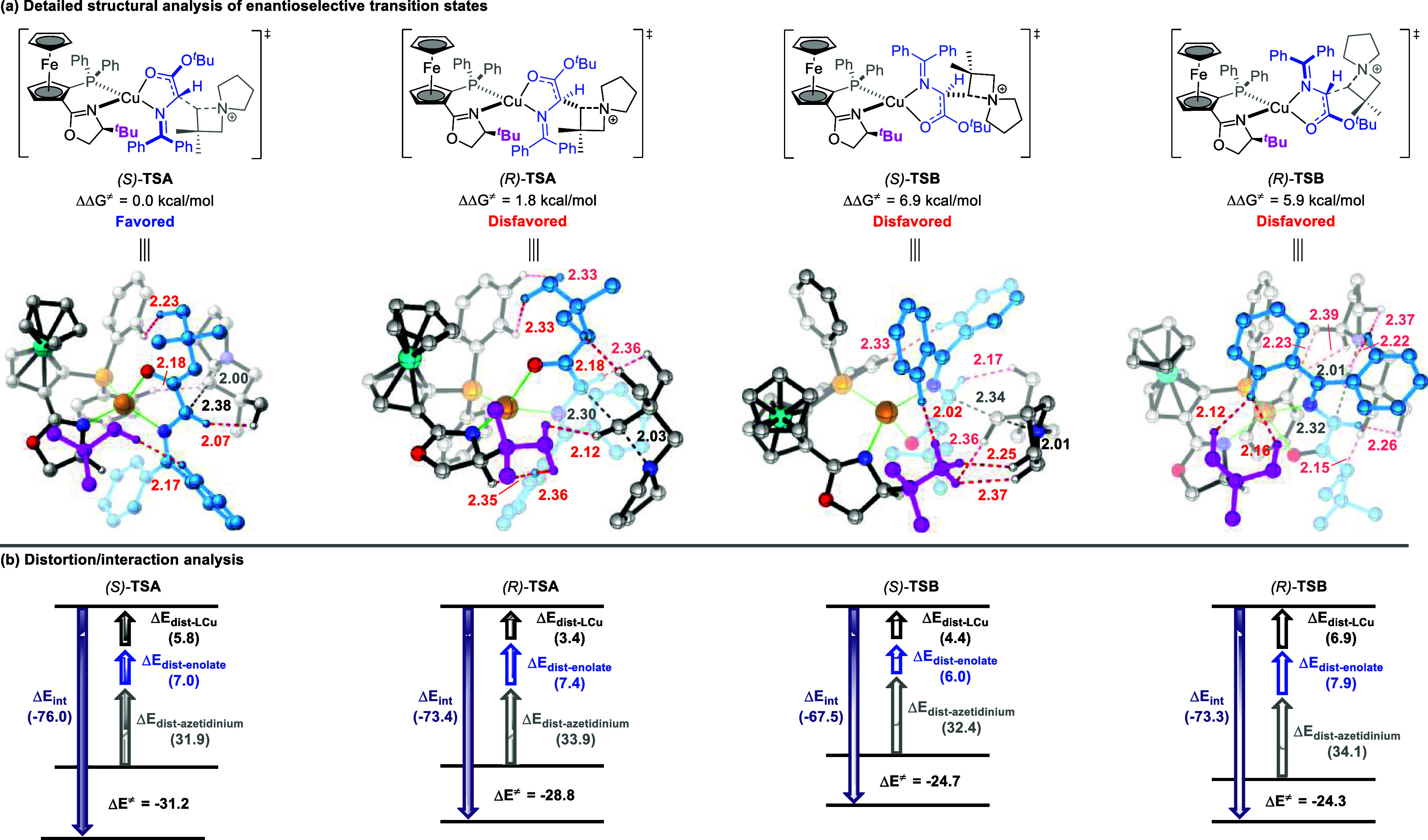
Structural and distortion/interaction analyses of the transition states. (a) Possible enantioselective transition states in the intermolecular Cu-catalyzed reaction of azomethine ylides and azetidiniums with detailed structural analysis. Steric clashes of H···H are shown in red values. (b) Distortion/interaction analysis of **TSA** and **TSB**. All energies (dispersion interaction energies, distortion energies, and activation energies) are shown in kcal/mol. The key bond lengths in the enantioselective transition states are given in angstroms, and some hydrogen atoms are omitted for clarity.

In the enantiodetermining transition states, the enolate configuration is locked by the ring structure in two different orientations, namely, **TSA** and **TSB**. With the bulky ^*t*^Bu group of the ligand blocking the front face, the azetidinium electrophile approaches the nucleophilic enolate from the back face through (*S*)-**TSA**, which is energetically most favorable by at least 1.8 kcal/mol relative to the other three isomers, thereby effectively circumventing the influence of the *tert*-butyl group in the chiral ligand. To further understand how the chiral catalyst induces enantioselectivity, we conducted detailed structural and distortion–interaction analyses^14^ of the four competing transition states. As illustrated in Figure 2a, (*S*)-**TSA** exhibits less steric repulsion among the chiral ligand, enolate, and azetidinium motifs, as indicated by the fewer steric clashes of H···H repulsive interactions. In contrast, in (*R*)-**TSA**, the azetidinium motif approaches from the front face of the enolate plane, which leads to a greater steric congestion.

We also examined the corresponding **TSB** structures, which were found to be energetically more disfavored, likely due to more pronounced steric repulsion and the lack of significant dispersion interactions (e.g., C–H···π, π···π interaction; see Figure S1 for details on noncovalent interactions). Indeed, further distortion–interaction analysis in Figure 2b revealed that the distortion energy of the azetidinium fragment (Δ*E*_dist‑azetidinium_) in the four transition states is much larger than that of Δ*E*_dist‑LCu_ and Δ*E*_dist‑enolate_. Notably, (*S*)-**TSA** possesses the smallest azetidinium deformation energy, which correlates with its low steric strain. In addition, it also benefits from a more favorable interaction energy (Δ*E*_int_ = −76.0 kcal/mol), thus, further favoring this pathway. Overall, the key enantiodetermining factor arises from steric repulsions between the ligand-substrate and substrate–substrate, which determine the formation of the major (*S*)-enantiomer. The computed energy difference of 1.8 kcal/mol between (*S*)-**TSA** and (*R*)-**TSA** agrees qualitatively with the observed experimental enantioselectivity.

## Conclusion

In summary, we have developed efficient Cu-catalyzed protocols for the enantioselective ring-opening of azetidiniums, representing the first example of this transformation by transition-metal catalysis. With the suitable design of a borylation/cyclization sequence as well as the choice of a superior ferrocene-based bidentate ligand, diverse densely functionalized tetrahydropyrans and piperidine compounds bearing an α-methylene unit were conveniently accessed from readily available enone-tethered azetidinium substrates. Three new stereogenic centers and two new bonds (C–B and C–C) were concomitantly constructed with good efficiency and high enantioselectivity and diastereoselectivity. This also serves as an unorthodox disconnection strategy in related heterocycle synthesis. Control experiments suggested that the borylation step served as the enantiodetermining step, which governs the stereochemistry in the subsequent intramolecular C­(sp^3^)–C­(sp^3^) formation. This protocol was also extended to an intermolecular variant. Efficient C­(sp^3^)–C­(sp^3^) bond formation between azomethine ylides and azetidiniums was achieved, providing expedient access to the valuable ornithine derivatives with high enantioselectivity. DFT studies provided insights into the enantiodetermining transition states. The highly enantioenriched products from the processes described above were demonstrated as useful precursors to other chiral synthetic building blocks.

## Supplementary Material



## Notes

Experimental details, materials, and methods, including spectroscopic data of all compounds, intramolecular and intermolecular asymmetric opening, DFT, NMR, effect of the alcohol additive, optimization of reaction conditions, effect of the counter anion, synthesis and evaluation of compound INT, non-covalent interaction analyses, product structure determination, and crystal data and structure refinement (PDF)
