# Supplementary material for: Copper-Catalyzed Enantioselective Intra- and Intermolecular Desymmetrization of Azetidiniums by Carbon Nucleophiles
Source: J Am Chem Soc. 2025 Nov 21;147(49):45639–47. doi: 10.1021/jacs.5c17131 (PMC12703672; doi:10.1021/jacs.5c17131)
Supplement: Supplementary file 1 [file ja5c17131_si_001.pdf]

Supporting Information

Copper-Catalyzed Enantioselective Intra- and Intermolecular Desymmetrization of Azetidiniums by Carbon Nucleophiles

Minghui Zhu,[1,2] Chaoshen Zhang,[1,2*] and Jianwei Sun[1,2*]

[1]*Jiangsu Key Laboratory of Advanced Catalytic Materials & Technology, School of Petrochemical Engineering, Changzhou University, Changzhou 213164, China*

[2]*Department of Chemistry and the Hong Kong Branch of Chinese National Engineering Research Centre for Tissue Restoration & Reconstruction, The Hong Kong University of Science and Technology, Clear Water Bay, Kowloon, Hong Kong SAR 999077, China*

Table of Contents

I. General Information S-2

II. Preparation of Substrates S-3

III. Intramolecular Asymmetric Opening of Azetidiniums S-26

IV. Intermolecular Asymmetric Opening of Azetidiniums S-45

V. Large-Scale Reactions S-64

VI. Product Transformations S-66

VII. Mechanistic Experiments S-72

VIII. DFT Calculation S-77

IX. Product Structure Determination S-99

X. References S-104

NMR Spectra and HPLC Traces

I. General Information

Flash column chromatography was performed over silica gel (200-300 or 300-400 mesh) purchased from Qindao Haiyang Co., China or SiliCycle® Inc., Canada. All air or moisture sensitive reactions were conducted in oven-dried glassware under nitrogen atmosphere using anhydrous solvents. Tetrahydrofuran was distilled from sodium/benzophenone. Anhydrous dichloromethane, methanol, toluene, acetonitrile, and *n*-hexane were purified by the Innovative® solvent purification system. Other anhydrous solvents were purchased from Sigma-Aldrich®, J&K® and Energy® and used as received. Chemicals were purchased from commercial suppliers, such as Sigma-Aldrich®, J&K®, Energy® and used without further purification unless otherwise stated. Bruker AVII, AVIII or NEO 400 spectrometer at 400 (^{1}H NMR), 101 MHz (^{13}C NMR), and 376 MHz (^{19}F NMR). Chemical shifts (δ values) were reported in ppm down field from an internal standard (^{1}H NMR: Me_4Si at 0.00 ppm and ^{13}C NMR: $CDCl_3$ at 77.00 ppm). Data for ^{1}H NMR were recorded as follows: chemical shift (δ, ppm), multiplicity (s = singlet; d = doublet; t = triplet; q = quarter; p = pentet; sept = septet; m = multiplet; br = broad), coupling constant (Hz), integration. The mass spectra were collected from a Waters Xevo G2-XS Tof, with ESI source; or a Waters GCT premier with EI/CI source. Optical rotations were measured on a JASCO P-2000 polarimeter or an AUTOPOL I Automatic polarimeter with $[\alpha]_D$ values reported in degrees; concentration (c) is in 10 mg/mL. Enantioselectivities were recorded on an Agilent HPLC instrument, using a chiral stationary phase column (Daicel CHIRALPAK® AD-3, AY-3, IA-3, IB N-3, IC-3, IE-3, CHIRALCEL® OD-3). The chiral HPLC methods were calibrated with the corresponding racemic mixtures.

II. Preparation of Substrates

The substrates were synthesized according to the following general procedures.

General Procedure A.

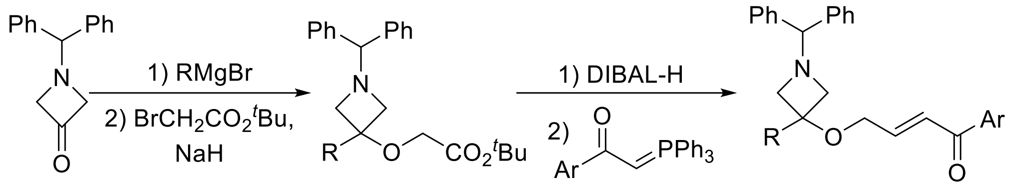

S0 **S1** **S2**

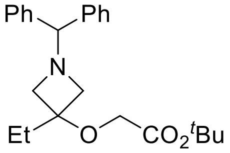

S1a

***tert*-Butyl 2-((1-benzhydryl-3-ethylazetidin-3-yl)oxy)acetate (S1a).** At -78 °C under nitrogen, to a solution of the ketone **S0** (7.1 g, 30.0 mmol) in anhydrous THF (60 mL) was added EtMgBr (36.0 mL, 36.0 mmol, 1.0 M in THF). The mixture was then stirred at the same temperature for 2 h. Next, a saturated NH_4Cl solution (30 mL) was added. The layers were separated, and the aqueous layer was extracted with ethyl acetate (50 mL × 2). The combined organic layers were washed with brine, dried over Na_2SO_4, filtered, and concentrated. The residue was used for the next step without further purification.

In a separate flask, to a suspension of sodium hydride (1.8 g, 45.0 mmol, 60 wt%) in THF (40 mL) at 0 °C was added dropwise a solution of the above-prepared crude 1-benzhydryl-3-ethylazetidin-3-ol in THF (20 mL). The mixture was warmed to room temperature and stirred for 2 h. Then, *tert*-butyl bromoacetate (5.2 mL, 36.0 mmol) was added dropwise at 0 °C. The reaction was stirred at room temperature, and the progress was monitored by thin layer chromatography. Upon completion (~10 h), water (20 mL) was added slowly to quench the reaction. The organic layer was separated, and the aqueous layer

was extracted with ethyl acetate (30 mL × 2). The combined organic layers were dried over anhydrous Na_2SO_4 and concentrated under reduced pressure. The residue was purified by silica gel column chromatography (*n*-hexane/EtOAc = 10:1) to afford the desired product **S1a** as a colorless oil (9.4 g, 82% yield).

^{1}H NMR (400 MHz, $CDCl_3$) δ 7.40 (d, *J* = 7.2 Hz, 4H), 7.25 (t, *J* = 7.5 Hz, 4H), 7.17 (t, *J* = 7.3 Hz, 2H), 4.42 (s, 1H), 3.89 (s, 2H), 3.20 – 2.96 (m, 4H), 1.84 (q, *J* = 7.4 Hz, 2H), 1.47 (s, 9H), 0.94 (t, *J* = 7.4 Hz, 3H).

^{13}C NMR (101 MHz, $CDCl_3$) δ 169.8, 142.5, 128.5, 127.5, 127.2, 81.6, 77.8, 75.7, 61.9, 28.5, 28.2, 7.6.

HRMS (ESI) Calcd for $C_{18}H_{22}NO^+$ $[M + H]^+$: 268.1696, Found: 268.1703.

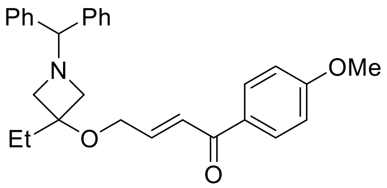

S2a

(*E*)-4-((1-Benzhydryl-3-ethylazetidin-3-yl)oxy)-1-(4-methoxyphenyl)but-2-en-1-one (S2a). To a stirred suspension of *tert*-butyl 2-((1-benzhydryl-3-ethylazetidin-3-yl)oxy)acetate **S1o** (7.6 g, 20.0 mmol) in CH_2Cl_2 (80 mL) at -78 °C was slowly added DIBAL-H (2.0 M solution in heptane, 10.0 mL, 20.0 mmol). After stirring for 30 min, a saturated potassium sodium tartrate tetrahydrate solution (20 mL) was added slowly to quench the reaction. The layers were separated, and the aqueous layer was extracted with DCM (3 × 20 mL), dried over anhydrous Na_2SO_4, filtered, and concentrated. The residue was used for the next step without further purification.

Under N_2, the crude aldehyde was dissolved in $CHCl_3$ (40 mL). To this solution was added the 1-(4-methoxyphenyl)-2-(triphenylphosphoranylidene)ethanone (9.8 g, 24 mmol) in one portion. The reaction mixture stirred at 65 °C for 5 h and then concentrated *in vacuo*. The crude mixture was purified by column chromatography (*n*-hexane/EtOAc = 5:1)

to afford the desired product **S2a** as a light yellow oil (3.7 g, 42% yield).

^{1}H NMR (400 MHz, $CDCl_3$) δ 8.02 – 7.95 (m, 2H), 7.41 (d, *J* = 7.4 Hz, 4H), 7.29 – 7.23 (m, 4H), 7.22 – 7.14 (m, 3H), 7.06 (dt, *J* = 15.3, 3.9 Hz, 1H), 6.99 – 6.92 (m, 2H), 4.42 (s, 1H), 4.13 (dd, *J* = 3.8, 2.0 Hz, 2H), 3.87 (s, 3H), 3.18 (d, *J* = 7.9 Hz, 2H), 3.06 (d, *J* = 8.2 Hz, 2H), 1.90 (q, *J* = 7.3 Hz, 2H), 0.95 (t, *J* = 7.4 Hz, 3H).

^{13}C NMR (101 MHz, $CDCl_3$) δ 188.8, 163.6, 144.2, 142.5, 131.1, 130.8, 128.5, 127.6, 127.2, 124.5, 113.9, 78.0, 75.3, 62.3, 62.2 (2C), 55.6, 28.3, 7.6.

HRMS (ESI) Calcd for $C_{29}H_{32}NO_3^+$ $[M + H]^+$: 442.2377, Found: 442.2381.

General Procedure B.

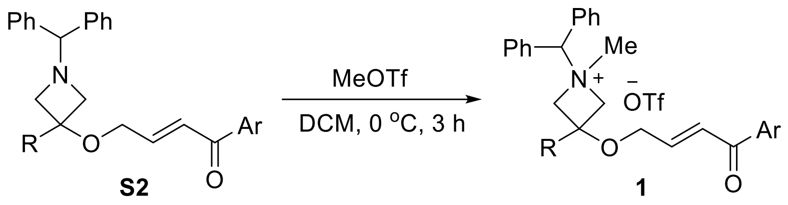

Under N_2 at 0 °C, methyl trifluoromethanesulfonate (0.51 mL, 3.0 mmol, 1.0 equiv) was added dropwise to a solution of azetidine **S2** (3.0 mmol) in DCM (15 mL). The reaction mixture was stirred at 0 °C for 3 h before it was concentrated under reduced pressure. Then, the crude mixture was purified by flash column chromatography to afford the desired product **1**.

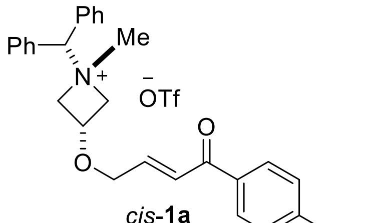

cis-**1a**

(*E*)-1-Benzhydryl-3-((4-(4-methoxyphenyl)-4-oxobut-2-en-1-yl)oxy)-1-methylazetidin-1-ium trifluoromethanesulfonate (*cis*-1a) was prepared according to the General Procedure B as a white foam (chromatography eluent: MeOH/DCM = 20:1) in 45% yield (779.2 mg, a single diastereomer).

^{1}H NMR (400 MHz, $CDCl_3$) δ 7.92 (d, *J* = 8.9 Hz, 2H), 7.60 (d, *J* = 7.4 Hz, 4H),

7.45 – 7.35 (m, 6H), 6.95 (d, *J* = 8.9 Hz, 2H), 6.87 (d, *J* = 15.4 Hz, 1H), 6.73 (dt, *J* = 15.4, 4.3 Hz, 1H), 5.96 (s, 1H), 4.85 (p, *J* = 6.2 Hz, 1H), 4.77 – 4.57 (m, 4H), 4.11 – 4.03 (m, 2H), 3.84 (s, 3H), 3.37 (s, 3H).

^{13}C NMR (101 MHz, $CDCl_3$) δ 188.0, 163.5, 132.1, 130.8, 130.0, 129.91, 129.86, 129.5, 125.3, 120.7 (q, J_{C-F} = 320.3 Hz), 113.7, 78.7, 69.1, 68.3, 64.7, 55.3, 51.5.

^{19}F NMR (377 MHz, $CDCl_3$) δ -78.15.

HRMS (ESI) Calcd for $C_{28}H_{30}NO_3^+$ [M - OTf]$^+$: 428.2220, Found: 428.2222.

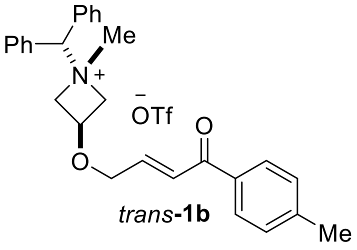

trans-**1b**

(*E*)-1-Benzhydryl-1-methyl-3-((4-oxo-4-(p-tolyl)but-2-en-1-yl)oxy)azetidin-1-ium trifluoromethanesulfonate (*trans*-1b) was prepared according to the General Procedure B as a white foam (chromatography eluent: MeOH/DCM = 20:1) in 52% yield (875.5 mg, a single diastereomer).

^{1}H NMR (400 MHz, $CDCl_3$) δ 7.84 (d, *J* = 8.2 Hz, 2H), 7.67 (d, *J* = 6.9 Hz, 4H), 7.49 – 7.34 (m, 6H), 7.23 (d, *J* = 8.1 Hz, 2H), 7.14 (d, *J* = 15.5 Hz, 1H), 6.88 (dt, *J* = 15.5, 4.5 Hz, 1H), 6.15 (s, 1H), 5.23 – 5.14 (m, 2H), 4.31 – 4.19 (m, 5H), 3.53 (s, 3H), 2.35 (s, 3H).

^{13}C NMR (101 MHz, $CDCl_3$) δ 189.1, 143.8, 141.1, 134.3, 131.8, 129.9, 129.8, 129.4, 129.1, 128.5, 125.6, 120.6 (q, J_{C-F} = 320.4 Hz), 78.1, 69.4 (2C), 68.2, 66.6, 50.9, 21.3.

^{19}F NMR (377 MHz, $CDCl_3$) δ -78.07.

HRMS (ESI) Calcd for $C_{28}H_{30}NO_2^+$ [M - OTf]$^+$: 412.2271, Found: 412.2276.

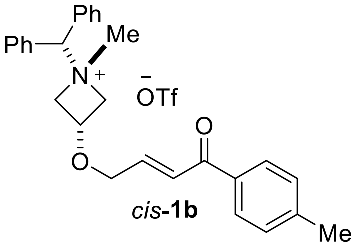

cis-**1b**

(*E*)-1-Benzhydryl-1-methyl-3-((4-oxo-4-(*p*-tolyl)but-2-en-1-yl)oxy)azetidin-1-ium trifluoromethanesulfonate (*cis*-1b) was prepared according to the General Procedure B as a white foam (chromatography eluent: MeOH/DCM = 20:1) in 47% yield (791.3 mg, a single diastereomer).

^{1}H NMR (400 MHz, $CDCl_3$) δ 7.80 (d, *J* = 8.2 Hz, 2H), 7.59 (d, *J* = 7.4 Hz, 4H), 7.45 – 7.35 (m, 6H), 7.27 (d, *J* = 8.0 Hz, 2H), 6.84 (dd, *J* = 15.5, 1.5 Hz, 1H), 6.78 – 6.69 (m, 1H), 5.94 (s, 1H), 4.85 (p, *J* = 6.3 Hz, 1H), 4.69 (td, *J* = 11.1, 5.0 Hz, 4H), 4.07 (dd, *J* = 4.1, 1.5 Hz, 2H), 3.37 (s, 3H), 2.40 (s, 3H).

^{13}C NMR (101 MHz, $CDCl_3$) δ 189.4, 144.0, 141.4, 134.6, 132.2, 130.1, 129.9, 129.6, 129.2, 128.7, 125.4, 120.7 (d, J_{C-F} = 320.2 Hz), 78.8, 69.2 (2C), 68.3, 64.9, 51.6, 21.5.

^{19}F NMR (377 MHz, $CDCl_3$) δ -78.17.

HRMS (ESI) Calcd for $C_{28}H_{30}NO_2^+$ [M - OTf]$^+$: 412.2271, Found: 412.2276.

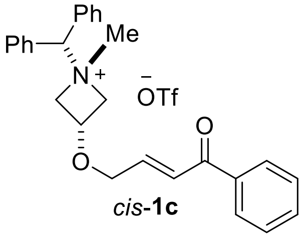

cis-**1c**

(*E*)-1-Benzhydryl-1-methyl-3-((4-oxo-4-phenylbut-2-en-1-yl)oxy)azetidin-1-ium trifluoromethanesulfonate (*cis*-1c) was prepared according to the General Procedure B as a white foam (chromatography eluent: MeOH/DCM = 20:1) in 41% yield (673.5 mg, a single diastereomer).

^{1}H NMR (400 MHz, $CDCl_3$) δ 7.92 – 7.85 (m, 2H), 7.60 – 7.53 (m, 4H), 7.50 – 7.34 (m, 9H), 6.83 (d, *J* = 15.5 Hz, 1H), 6.75 (dt, *J* = 15.5, 4.0 Hz, 1H), 5.91 (s, 1H), 4.85 (p, *J* = 6.3 Hz, 1H), 4.66 (d, *J* = 6.2 Hz, 4H), 4.07 (dd, *J* = 4.0, 1.4 Hz, 2H), 3.37 (s,

3H).

^{13}C NMR (101 MHz, $CDCl_3$) δ 190.0, 142.0, 137.2, 133.1, 132.2, 130.2, 130.0, 129.7, 128.66, 128.59, 125.5, 120.7 (d, $J_{C\text{-}F}$ = 320.1 Hz), 78.9, 69.3 (2C), 68.4, 65.0, 51.7.

^{19}F NMR (377 MHz, $CDCl_3$) δ -78.22.

HRMS (ESI) Calcd for $C_{27}H_{28}NO_2^+$ [M - OTf]$^+$: 398.2115, Found: 398.2113.

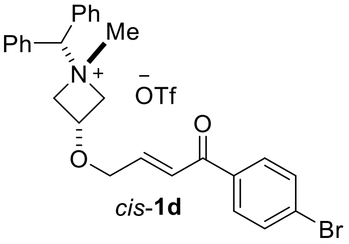

cis-**1d**

(*E*)-1-Benzhydryl-3-((4-(4-bromophenyl)-4-oxobut-2-en-1-yl)oxy)-1-methylazetidin-1-ium trifluoromethanesulfonate (*cis*-1d) was prepared according to the General Procedure B as a white foam (chromatography eluent: MeOH/DCM = 20:1) in 42% yield (787.6 mg, a single diastereomer).

^{1}H NMR (400 MHz, $CDCl_3$) δ 7.77 (d, *J* = 8.6 Hz, 2H), 7.63 – 7.55 (m, 6H), 7.44 – 7.36 (m, 6H), 6.88 – 6.72 (m, 2H), 5.94 (s, 1H), 4.86 (p, *J* = 6.3 Hz, 1H), 4.75 – 4.53 (m, 4H), 4.09 (d, *J* = 2.8 Hz, 2H), 3.36 (s, 3H).

^{13}C NMR (101 MHz, $CDCl_3$) δ 188.8, 142.5, 135.8, 132.1, 131.8, 130.1, 129.9, 129.6, 128.1, 124.9, 120.7 (q, $J_{C\text{-}F}$ = 320.1 Hz), 78.9, 69.2 (2C), 68.2, 64.8, 51.5.

^{19}F NMR (377 MHz, $CDCl_3$) δ -78.16.

HRMS (ESI) Calcd for $C_{27}H_{27}BrNO_2^+$ [M - OTf]$^+$: 476.1220, Found: 476.1237.

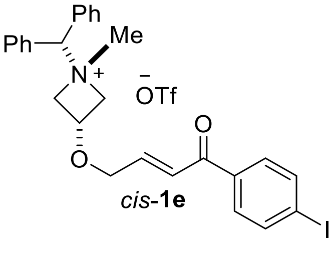

cis-**1e**

(*E*)-1-Benzhydryl-3-((4-(4-iodophenyl)-4-oxobut-2-en-1-yl)oxy)-1-methylazetidin-1-ium trifluoromethanesulfonate (*cis*-1e) was prepared

according to the General Procedure B as a white foam (chromatography eluent: MeOH/DCM = 20:1) in 42% yield (848.6 mg, a single diastereomer).

1H NMR (400 MHz, $CDCl_3$) δ 7.83 (d, *J* = 8.4 Hz, 2H), 7.61 (d, *J* = 8.4 Hz, 2H), 7.56 (d, *J* = 7.3 Hz, 4H), 7.47 – 7.37 (m, 6H), 6.86 – 6.70 (m,2H), 5.90 (s, 1H), 4.87 (p, *J* = 6.2 Hz, 1H), 4.66 (d, *J* = 6.3 Hz, 4H), 4.08 (d, *J* = 3.0 Hz, 2H), 3.37 (s, 3H).

^{13}C NMR (101 MHz, $CDCl_3$) δ 189.2, 142.5, 137.9, 136.5, 132.1, 130.2, 130.03, 129.97, 129.7, 125.1, 120.7 (d, J_{C-F} = 320.1 Hz), 101.1, 79.1, 69.4 (2C), 68.4, 65.0, 51.6.

^{19}F NMR (377 MHz, $CDCl_3$) δ -78.20.

HRMS (ESI) Calcd for $C_{27}H_{27}INO_2^+$ [M - OTf]$^+$: 524.1081, Found: 524.1086.

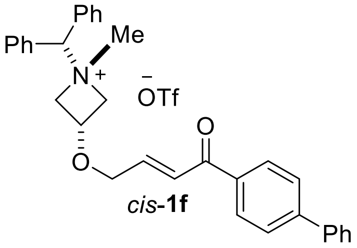

cis-**1f**

(*E*)-3-((4-([1,1'-Biphenyl]-4-yl)-4-oxobut-2-en-1-yl)oxy)-1-benzhydryl-1-methylazetidin-1-ium trifluoromethanesulfonate (*cis*-1f) was prepared according to the General Procedure B as a white foam (chromatography eluent: MeOH/DCM = 20:1) in 43% yield (803.9 mg, a single diastereomer).

^{1}H NMR (400 MHz, $CDCl_3$) δ 7.98 (d, *J* = 8.4 Hz, 2H), 7.69 (d, *J* = 8.5 Hz, 2H), 7.64 – 7.53 (m, 7H), 7.49 – 7.33 (m, 9H), 6.89 (d, *J* = 15.5 Hz, 1H), 6.78 (dt, *J* = 15.5, 4.2 Hz, 1H), 5.93 (s, 1H), 4.86 (p, *J* = 6.3 Hz, 1H), 4.74 – 4.61 (m, 4H), 4.09 (dd, *J* = 4.2, 1.5 Hz, 2H), 3.37 (s, 3H).

^{13}C NMR (101 MHz, $CDCl_3$) δ 189.3, 145.7, 141.8, 139.6, 135.9, 132.2, 130.2, 130.0, 129.6, 129.2, 128.9, 128.2, 127.1, 125.5, 120.7 (d, J_{C-F} = 320.2 Hz), 78.9, 69.3 (2C), 68.4, 65.0, 51.6.

^{19}F NMR (377 MHz, $CDCl_3$) δ -78.12.

HRMS (ESI) Calcd for $C_{33}H_{32}NO_2^+$ [M - OTf]$^+$: 474.2428, Found: 474.2444.

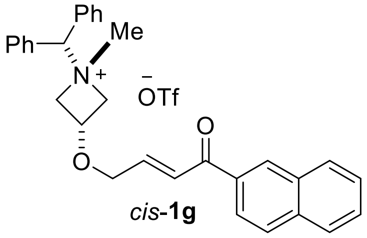

(*E*)-1-Benzhydryl-1-methyl-3-((4-(naphthalen-2-yl)-4-oxobut-2-en-1-yl)oxy)azetidin-1-ium trifluoromethanesulfonate (*cis*-1g) was prepared according to the General Procedure B as a white foam (chromatography eluent: MeOH/DCM = 20:1) in 41% yield (734.6 mg, a single diastereomer).

^{1}H NMR (400 MHz, $CDCl_3$) δ 8.43 (s, 1H), 8.05 – 7.91 (m, 2H), 7.86 (dd, *J* = 13.0, 8.4 Hz, 2H), 7.62 – 7.49 (m, 6H), 7.40 (t, *J* = 7.5 Hz, 4H), 7.36 – 7.23 (m, 2H), 7.01 (d, *J* = 15.5 Hz, 1H), 6.80 (dt, *J* = 15.4, 4.4 Hz, 1H), 5.93 (s, 1H), 4.85 (p, *J* = 6.2 Hz, 1H), 4.68 (td, *J* = 11.8, 6.5 Hz, 4H), 4.09 (dd, *J* = 4.3, 1.6 Hz, 2H), 3.35 (s, 3H).

^{13}C NMR (101 MHz, $CDCl_3$) δ 189.7, 141.8, 135.4, 134.5, 132.4, 132.1, 130.4, 130.1, 129.9, 129.6, 129.5, 128.52, 128.47, 127.7, 126.8, 125.6, 124.1, 120.8 (q, $J_{C\text{-}F}$ = 320.3 Hz), 78.9, 69.3 (2C), 68.4, 64.9, 51.5.

^{19}F NMR (377 MHz, $CDCl_3$) δ -78.09.

HRMS (ESI) Calcd for $C_{31}H_{30}NO_2^+$ [M - OTf]$^+$: 448.2271, Found: 448.2273.

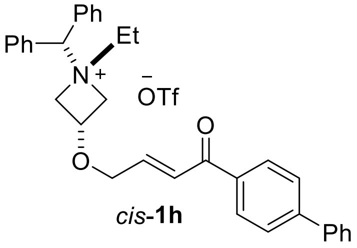

(*E*)-3-((4-([1,1'-Biphenyl]-4-yl)-4-oxobut-2-en-1-yl)oxy)-1-benzhydryl-1-ethylazetidin-1-ium trifluoromethanesulfonate (*cis*-1h) was prepared according to the General Procedure B as a white foam (chromatography eluent: MeOH/DCM = 20:1) in 41% yield (784.4 mg, a single diastereomer).

^{1}H NMR (400 MHz, $CDCl_3$) δ 7.97 (d, *J* = 8.4 Hz, 2H), 7.70 (d, *J* = 8.4 Hz, 2H), 7.66 – 7.60 (m, 2H), 7.57 (d, *J* = 7.4 Hz, 4H), 7.50 – 7.33 (m, 9H), 6.80 – 6.66 (m,

2H), 5.62 (s, 1H), 4.82 – 4.63 (m, 4H), 4.01 (d, J = 2.7 Hz, 2H), 3.70 (q, J = 7.0 Hz, 2H), 1.39 (t, J = 7.1 Hz, 3H).

^{13}C NMR (101 MHz, $CDCl_3$) δ 189.4, 145.7, 141.9, 139.7, 135.9, 132.0, 130.2, 130.1, 129.7, 129.3, 128.9, 128.2, 127.18, 127.15, 127.1, 125.3, 120.8 (d, J_{C-F} = 320.3 Hz), 77.2, 75.1, 68.1, 65.9 (2C), 64.9, 58.6, 8.7.

^{19}F NMR (377 MHz, $CDCl_3$) δ -78.14, -78.15.

HRMS (ESI) Calcd for $C_{34}H_{34}NO_2^+$ [M - OTf]$^+$: 488.2584, Found: 488.2580.

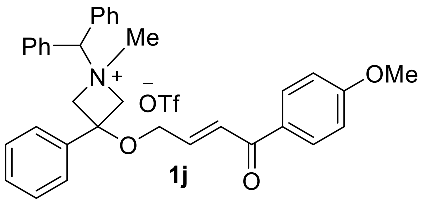

1j

(*E*)-1-Benzhydryl-3-((4-(4-methoxyphenyl)-4-oxobut-2-en-1-yl)oxy)-1-methyl-3-phenylazetidin-1-ium trifluoromethanesulfonate (1j) was prepared according to the General Procedure B as a white foam (without purification) in 97% yield (1.9 g, a mixture of two diastereomers).

^{1}H NMR (400 MHz, $CDCl_3$) δ 7.94 (d, J = 8.9 Hz, 2H), 7.66 (d, J = 7.2 Hz, 4H), 7.43 – 7.29 (m, 6H), 7.22 – 7.13 (m, 4H), 6.92 (d, J = 8.9 Hz, 2H), 6.85 – 6.80 (m, 3H), 6.06 (s, 1H), 5.25 (d, J = 13.0 Hz, 2H), 4.82 (d, J = 13.0 Hz, 2H), 3.98 (dd, J = 4.3, 1.7 Hz, 2H), 3.82 (s, 3H), 3.67 (s, 3H).

^{13}C NMR (101 MHz, $CDCl_3$) δ 188.2, 163.6, 141.0, 134.8, 132.1, 131.0, 130.1, 130.04, 130.03, 129.7, 129.2, 129.0, 125.2, 125.1, 120.8 (d, J_{C-F} = 320.2 Hz), 113.8, 78.3, 75.4, 69.9 (2C), 63.8, 55.4, 53.1.

^{19}F NMR (377 MHz, $CDCl_3$) δ -78.07.

HRMS (ESI) Calcd for $C_{34}H_{34}NO_3^+$ [M - OTf]$^+$: 504.2533, Found: 504.2537.

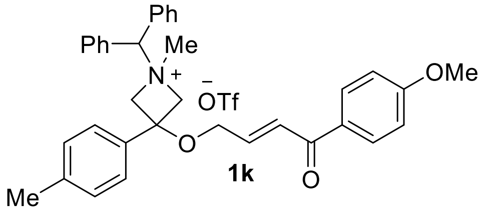

1k

(*E*)-1-Benzhydryl-3-((4-(4-methoxyphenyl)-4-oxobut-2-en-1-yl)oxy)-1-methyl-3-(p-tolyl)azetidin-1-ium trifluoromethanesulfonate (2k) was prepared according to the General Procedure B as a white foam (without purification) in 93% yield (1.9 g, a mixture of two diastereomers).

^{1}H NMR (400 MHz, $CDCl_3$) major isomer δ 7.95 (d, *J* = 8.9 Hz, 2H), 7.65 (d, *J* = 7.2 Hz, 4H), 7.45 – 7.32 (m, 6H), 7.17 (d, *J* = 15.4 Hz, 1H), 6.95 (dd, *J* = 16.8, 8.5 Hz, 4H), 6.85 (dt, *J* = 15.6, 4.5 Hz, 1H), 6.68 (d, *J* = 8.1 Hz, 2H), 6.08 (s, 1H), 5.20 (d, *J* = 12.8 Hz, 2H), 4.78 (d, *J* = 12.9 Hz, 2H), 4.00 – 3.91 (m, 2H), 3.83 (s, 3H), 3.67 (s, 3H), 2.23 (s, 3H).

^{13}C NMR (101 MHz, $CDCl_3$) δ 188.2, 163.6, 141.0, 139.3, 132.2, 131.9, 131.0, 130.23, 130.18, 130.10, 130.06, 129.72, 129.67, 125.2, 125.0, 120.9 (d, J_{C-F}= 320.3 Hz), 113.9, 78.2, 75.5, 70.0 (2C), 63.8, 55.4, 53.1, 21.0.

^{19}F NMR (377 MHz, $CDCl_3$) δ -78.10.

HRMS (ESI) Calcd for $C_{35}H_{36}NO_3^+$ [M - OTf]$^+$: 518.2690, Found:518.2842.

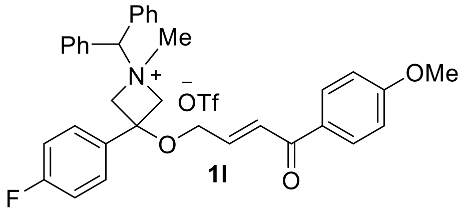

1l

(*E*)-1-Benzhydryl-3-(4-fluorophenyl)-3-((4-(4-methoxyphenyl)-4-oxobut-2-en-1-yl)oxy)-1-methylazetidin-1-ium trifluoromethanesulfonate (1l) was prepared according to the General Procedure B as a white foam (without purification) in 95% yield (1.9 g, a mixture of two diastereomers).

^{1}H NMR (400 MHz, $CDCl_3$) major isomer δ 7.94 (d, *J* = 8.9 Hz, 2H), 7.65 (d, *J* = 7.1 Hz, 4H), 7.43 – 7.35 (m, 7H), 7.17 (d, *J* = 15.4 Hz, 1H), 6.98 – 6.77 (m, 6H), 6.06

(s, 1H), 5.24 (d, *J* = 13.0 Hz, 2H), 4.80 (d, *J* = 13.0 Hz, 2H), 4.00 – 3.93 (m, 2H), 3.83 (s, 3H), 3.65 (s, 3H).

^{13}C NMR (101 MHz, $CDCl_3$) δ 188.1, 163.7, 162.8 (d, J_{C-F} = 250.3 Hz), 140.7, 132.1, 131.0, 130.2, 130.1, 130.0, 129.8, 127.4 (d, J_{C-F} = 8.4 Hz), 125.4, 120.9 (d, J_{C-F} = 320.1 Hz), 116.1 (d, J_{C-F} = 21.8 Hz), 113.9, 78.4, 75.0, 70.1 (2C), 63.9, 55.4, 53.2.

^{19}F NMR (377 MHz, $CDCl_3$) δ -78.12, -111.05.

HRMS (ESI) Calcd for $C_{34}H_{33}FNO_3^+$ [M - OTf]$^+$: 522.2439, Found: 522.2439.

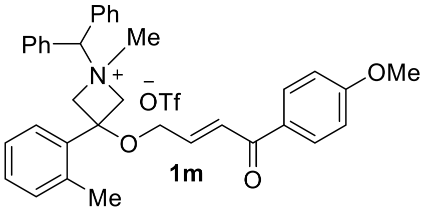

1m

(*E*)-1-Benzhydryl-3-((4-(4-methoxyphenyl)-4-oxobut-2-en-1-yl)oxy)-1-methyl-3-(*o*-tolyl)azetidin-1-ium trifluoromethanesulfonate (1m) was prepared according to the General Procedure B as a white foam (without purification) in 97% yield (1.9 g, a mixture of two diastereomers).

^{1}H NMR (400 MHz, $CDCl_3$) major isomer δ 7.91 (d, *J* = 8.9 Hz, 2H), 7.66 (d, *J* = 7.5 Hz, 4H), 7.35 (t, *J* = 7.5 Hz, 4H), 7.27 (d, *J* = 6.8 Hz, 2H), 7.17 – 7.05 (m, 2H), 7.00 (dd, *J* = 17.1, 7.6 Hz, 2H), 6.91 (d, *J* = 8.9 Hz, 2H), 6.85 (d, *J* = 7.4 Hz, 1H), 6.79 (dt, *J* = 15.4, 4.6 Hz, 1H), 6.04 (s, 1H), 5.58 – 5.13 (m, 2H), 4.91 (d, *J* = 12.4 Hz, 2H), 3.82 – 3.80 (m, 4H), 3.68 – 3.62 (m, 4H), 2.20 (s, 3H).

^{13}C NMR (101 MHz, $CDCl_3$) δ 188.1, 163.5, 141.2, 135.9, 132.1, 132.0, 131.8, 130.9, 130.0, 129.9, 129.6, 129.5, 126.6, 125.7, 125.0, 120.9 (d, J_{C-F} = 320.3 Hz), 113.8, 78.5, 76.0, 63.6, 55.4 (2C), 52.9, 19.2.

^{19}F NMR (377 MHz, $CDCl_3$) δ -78.05.

HRMS (ESI) Calcd for $C_{35}H_{36}NO_3^+$ [M - OTf]$^+$: 518.2690, Found: 518.2704.

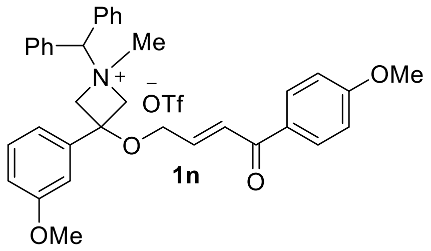

(*E*)-1-Benzhydryl-3-(3-methoxyphenyl)-3-((4-(4-methoxyphenyl)-4-oxobut-2-en-1-yl)oxy)-1-methylazetidin-1-ium trifluoromethanesulfonate (1n) was prepared according to the General Procedure B as a white foam (without purification) in 94% yield (1.9 g, a mixture of two diastereomers).

^{1}H NMR (400 MHz, $CDCl_3$) major isomer δ 7.94 (d, *J* = 8.9 Hz, 2H), 7.66 (d, *J* = 7.2 Hz, 4H), 7.41 – 7.31 (m, 6H), 7.18 (d, *J* = 15.4 Hz, 1H), 7.08 (t, *J* = 8.0 Hz, 1H), 6.92 (d, *J* = 8.9 Hz, 2H), 6.85 (dt, *J* = 15.4, 4.4 Hz, 1H), 6.75 (dd, *J* = 8.2, 2.1 Hz, 1H), 6.47 – 6.44 (m, 1H), 6.33 (d, *J* = 8.4 Hz, 1H), 6.06 (s, 1H), 5.24 (d, *J* = 13.1 Hz, 2H), 4.79 (d, *J* = 12.9 Hz, 2H), 3.99 (dd, *J* = 4.3, 1.6 Hz, 2H), 3.83 (s, 3H), 3.70 (s, 3H), 3.67 (s, 3H).

^{13}C NMR (101 MHz, $CDCl_3$) δ 188.1, 163.6, 160.0, 140.9, 136.4, 132.1, 131.0, 130.13, 130.08, 129.7, 125.2, 120.9 (d, $J_{C\text{-}F}$ = 320.3 Hz), 117.2, 114.9, 113.8, 110.7, 78.3, 75.4, 69.9 (2C), 63.9, 55.4, 55.3, 53.1.

^{19}F NMR (377 MHz, $CDCl_3$) δ -78.09.

HRMS (ESI) Calcd for $C_{35}H_{36}NO_4^+$ [M - OTf]$^+$: 534.2639, Found: 534.2639.

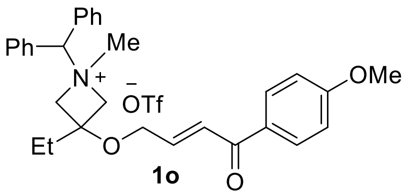

(*E*)-1-Benzhydryl-3-ethyl-3-((4-(4-methoxyphenyl)-4-oxobut-2-en-1-yl)oxy)-1-methylazetidin-1-ium trifluoromethanesulfonate (2o) was prepared according to the General Procedure B as a white foam (without purification) in 90% yield (1.6 g, a mixture of two diastereomers).

^{1}H NMR (400 MHz, $CDCl_3$) δ 7.96 (d, *J* = 8.9 Hz, 2H), 7.68 (d, *J* = 7.1 Hz, 4H),

7.50 – 7.37 (m, 6H), 7.22 (d, *J* = 15.4 Hz, 1H), 7.01 – 6.88 (m, 3H), 6.05 (s, 1H), 4.80 (d, *J* = 12.8 Hz, 2H), 4.39 (d, *J* = 12.8 Hz, 2H), 4.18 (dd, *J* = 4.1, 1.5 Hz, 2H), 3.82 (s, 3H), 3.51 (s, 3H), 1.26 (q, *J* = 7.3 Hz, 2H), 0.57 (t, *J* = 7.4 Hz, 3H).

^{13}C NMR (101 MHz, $CDCl_3$) δ 188.2, 163.6, 141.3, 132.3, 130.9, 130.1, 130.0, 129.6, 125.1, 120.8 (d, *J* = 320.3 Hz), 113.8, 78.0, 74.9, 70.1 (2C), 62.5, 55.4, 52.7, 25.3, 6.3.

^{19}F NMR (377 MHz, $CDCl_3$) δ -78.10.

HRMS (ESI) Calcd for $C_{30}H_{34}NO_3^+$ [M - OTf]$^+$: 456.2533, Found: 456.2541.

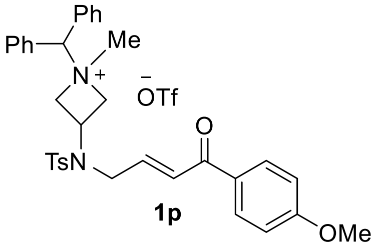

1p

(*E*)-1-Benzhydryl-3-((N-(4-(4-methoxyphenyl)-4-oxobut-2-en-1-yl)-4-methylphenyl)sulfonamido)-1-methylazetidin-1-ium trifluoromethane sulfonate (1p) was prepared according to the General Procedure B as a white foam (without purification) in 91% yield (2.0 g, a single diastereomer).

^{1}H NMR (400 MHz, $CDCl_3$) δ 7.90 (d, *J* = 8.9 Hz, 2H), 7.63 (d, *J* = 8.3 Hz, 2H), 7.49 (d, *J* = 7.3 Hz, 4H), 7.40 (t, *J* = 7.3 Hz, 4H), 7.33 (dd, *J* = 12.3, 7.7 Hz, 4H), 6.96 (d, *J* = 8.9 Hz, 4H), 6.80 (d, *J* = 15.4 Hz, 2H), 6.52 – 6.39 (m, 1H), 5.86 (s, 1H), 4.95 (q, *J* = 7.5 Hz, 1H), 4.91 – 4.82 (m, 2H), 4.57 – 4.47 (m, 2H), 3.88 (s, 3H), 3.50 (d, *J* = 4.4 Hz, 2H), 3.39 (s, 3H), 2.36 (s, 3H).

^{13}C NMR (101 MHz, $CDCl_3$) δ 187.9, 163.8, 145.2, 140.4, 133.5, 131.7, 131.2, 130.4, 130.0, 129.88, 129.85, 127.8, 127.5, 120.6 (d, J_{C-F} = 320.0 Hz), 114.0, 79.3, 77.2, 66.1, 55.5, 50.8, 47.5, 45.8, 21.5.

^{19}F NMR (377 MHz, $CDCl_3$) δ -78.29.

HRMS (ESI) Calcd for $C_{35}H_{37}N_2O_4S^+$ [M - OTf]$^+$: 581.2469, Found: 581.2574.

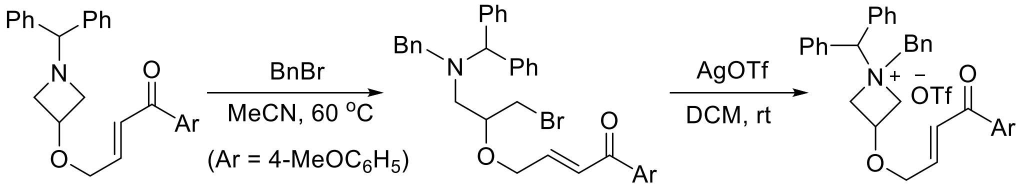

S2b **1i'** **1i**

(*E*)-1-Benzhydryl-1-benzyl-3-((4-(4-methoxyphenyl)-4-oxobut-2-en-1-yl) oxy) azetidin-1-ium trifluoromethanesulfonate (1i). Under N_2, to an oven-dried flask charged with **S2b** (2.1 g, 5.0 mmol, 1.0 equiv) were added MeCN (10 mL) and benzyl bromide (1.2 mL, 10.0 mmol, 2.0 equiv). The mixture was stirred at 60 °C for 12 h and then concentrated. The residue was purified by flash column chromatography (chromatography eluent: *n*-hexane/ethyl acetate = 10:1) to form the amine **1i'**.

The amine **1i'** was dissolved in DCM (20 mL) and the solution was added to an oven-dried flask charged with AgOTf (1.3 g, 5.0 mmol, 1.0 equiv). The reaction mixture was stirred in the darkness at room temperature for 12 h before it was concentrated under reduced pressure. The residue was purified by flash column chromatography to afford the desired product **1i** as a white solid in 75% yield over 2 steps (2.4 g, a mixture of two diastereomers) .

^{1}H NMR (400 MHz, $CDCl_3$) δ 7.94 – 7.82 (m, 2.12H), 7.72 – 7.65 (m, 4.93H), 7.50 – 7.38 (m, 10.60H), 6.99 – 6.90 (m, 2.05H), 6.76 (d, *J* = 15.4 Hz, 0.63H), 6.68 – 6.47 (m, 1.39H), 6.07 (s, 0.36H), 6.01 (s, 0.61H), 5.12 (dd, *J* = 12.8, 7.0 Hz, 1.26H), 4.90 (dd, *J* = 12.5, 6.9 Hz, 0.79H), 4.78 (dd, *J* = 12.5, 6.3 Hz, 0.80H), 4.69 – 4.65 (m, 2.39H), 4.62 (s, 0.77H), 3.88 – 3.86 (m, 4.17H), 3.77 (d, *J* = 3.9 Hz, 0.76H), 3.38 – 3.20 (m, 1.04H).

^{13}C NMR (101 MHz, $CDCl_3$) δ 188.0, 187.9, 163.6, 140.3, 133.0, 132.8, 132.72, 132.69, 131.00, 130.96, 130.9, 130.3, 130.14, 130.09, 130.0, 129.9, 129.8, 129.6, 129.4, 128.1, 127.8, 125.6, 125.4, 120.9 (d, J_{C-F} = 320.3 Hz), 113.8, 78.3, 77.5, 67.9, 67.9, 66.1, 65.5, 64.8, 64.6, 64.3, 64.1, 55.5, 55.4.

^{19}F NMR (377 MHz, $CDCl_3$) δ -78.04, -78.05.

HRMS (ESI) Calcd for $C_{34}H_{34}NO_3^+$ [M - OTf]$^+$: 504.2533, Found: 504.2696.

General Procedure C.

All the imine esters **5** used are known compounds and were prepared according to the literature procedure.[1] Azetidinium **4q** was prepared according to the literature procedure.[2] Other azetidiniums **4** were prepared according to the following procedure.

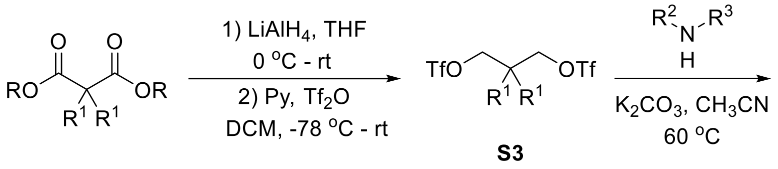

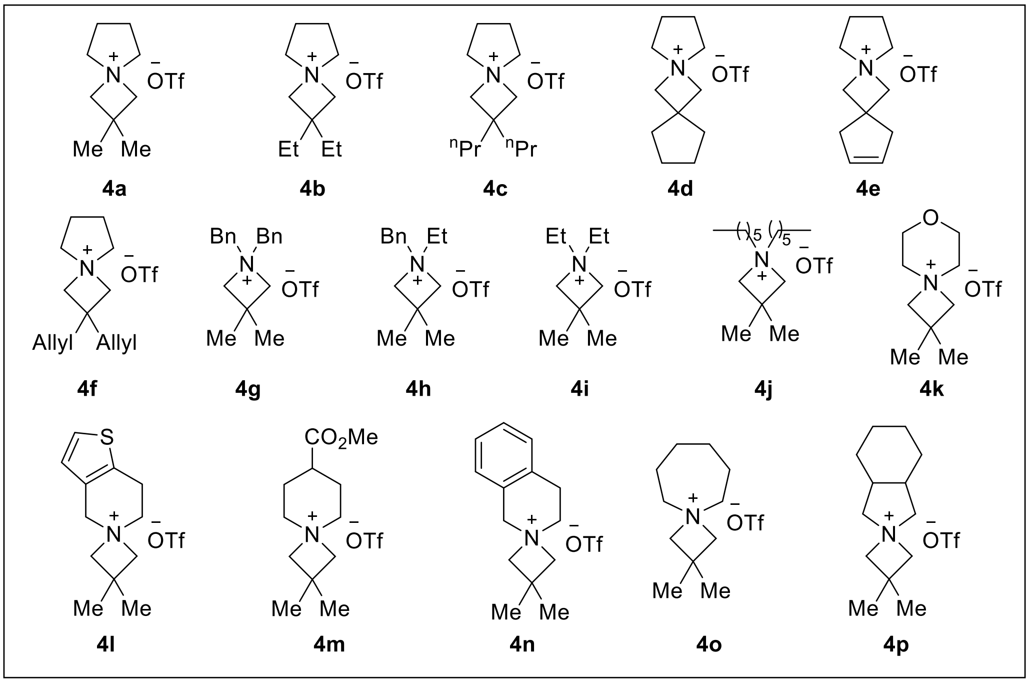

Substrates **S3** were all prepared according to the literature procedure.[3]

At room temperature, to a solution of the bis(triflate) **S3** (5.0 mmol, 1.0 equiv) in CH_3CN (10 mL) were added K_2CO_3 (829.2 mg, 6.0 mmol, 1.2 equiv) and the corresponding secondary amine (5.0 mmol, 1.0 equiv). The reaction mixture was stirred at 70 °C for 18 h before it was cooled to room temperature and concentrated under reduced pressure. Then DCM (15 mL) was added to the reaction mixture, which was filtered through a pad of celite. The filtrate was concentrated. The residue was recrystallized from DCM and *n*-hexane to give the pure azetidinium **4**.

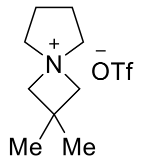

4a

2,2-Dimethyl-4-azaspiro[3.4]octan-4-ium trifluoromethanesulfonate (4a) was prepared according to the General Procedure C as a pale yellow solid in 70% yield (1.0 g).

^{1}H NMR (400 MHz, DMSO-d_6) δ 4.12 (s, 4H), 3.60 (t, J = 7.0 Hz, 4H), 1.94 (p, J = 3.6 Hz, 4H), 1.33 (s, 6H).

^{13}C NMR (101 MHz, DMSO-d_6) δ 72.7, 64.3, 28.7, 26.9, 20.9.

^{19}F NMR (376 MHz, DMSO-d_6) δ -77.77.

HRMS (ESI) Calcd for $C_9H_{18}N^+$ [M - OTf]$^+$: 140.1434, Found: 140.1439.

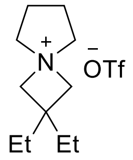

4b

2,2-Diethyl-4-azaspiro[3.4]octan-4-ium trifluoromethanesulfonate (4b) was prepared according to the General Procedure C as a pale yellow solid in 64% yield (1.0 g).

^{1}H NMR (400 MHz, DMSO-d_6) δ 4.08 (s, 4H), 3.59 (t, J = 6.9 Hz, 4H), 1.94 (p, J = 3.6 Hz, 4H), 1.70 (q, J = 7.4 Hz, 4H), 0.78 (t, J = 7.4 Hz, 6H).

^{13}C NMR (101 MHz, DMSO-d_6) δ 70.3, 64.6, 35.2, 27.8, 20.8, 7.8.

^{19}F NMR (376 MHz, DMSO-d_6) δ -77.77.

HRMS (ESI) Calcd for $C_{11}H_{22}N^+$ [M - OTf]$^+$: 168.1747, Found: 167.1751.

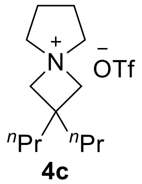

4c

2,2-Dipropyl-4-azaspiro[3.4]octan-4-ium trifluoromethanesulfonate (4c) was

prepared according to the General Procedure C as a pale yellow solid in 68% yield (1.2 g).

^{1}H NMR (400 MHz, DMSO-d_6) δ 4.11 (s, 4H), 3.61 – 3.58 (m, 1H), 1.96 – 1.92 (m, 4H), 1.66 – 1.62 (m, 4H), 1.20 – 1.14 (m, 4H), 0.90 (t, *J* = 7.2 Hz, 6H).

^{13}C NMR (101 MHz, DMSO-d_6) δ 120.8 (d, *J* = 322.3 Hz), 71.1, 64.6, 37.9, 34.4, 20.8, 16.6, 14.1.

^{19}F NMR (376 MHz, DMSO-d_6) δ -77.81.

HRMS (ESI) Calcd for $C_{13}H_{26}N^+$ [M - OTf]$^+$: 196.2060, Found: 196.2067.

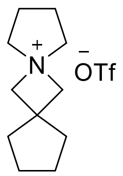

4d

5-Dzadispiro[4.1.47.15]dodecan-5-ium trifluoromethanesulfonate (4d) was prepared according to the General Procedure C as a pale yellow solid in 58% yield (0.9 g).

^{1}H NMR (400 MHz, DMSO-d_6) *δ* 4.24 (s, 4H), 3.59 (d, *J* = 6.6 Hz, 4H), 1.95 – 1.88 (m, 8H), 1.53 (t, *J* = 7.0 Hz, 4H).

^{13}C NMR (101 MHz, DMSO-d_6) *δ* 72.2, 63.5, 38.9, 37.3, 23.9, 21.0.

^{19}F NMR (376 MHz, DMSO-d_6) *δ* -77.80.

HRMS (ESI) Calcd for $C_{11}H_{20}N^+$ [M - OTf]$^+$: 166.1590, Found:.166.1595.

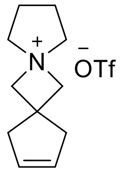

4e

5-Dzadispiro[4.1.47.15]dodec-9-en-5-ium trifluoromethanesulfonate (4e) was prepared according to the General Procedure C as a pale yellow solid in 50% yield (0.8 g).

^{1}H NMR (400 MHz, DMSO-*d*$_6$) δ 5.66 (s, 2H), 4.33 (s, 2H), 3.59 (t, *J* = 7.1 Hz, 2H), 2.75 (s, 2H), 2.00 – 1.90 (m, 3H).

^{13}C NMR (101 MHz, DMSO-*d*$_6$) δ 129.3, 73.6, 63.7, 44.8, 37.9, 21.4.

^{19}F NMR (376 MHz, DMSO-*d*$_6$) δ -77.76.

HRMS (ESI) Calcd for $C_{11}H_{18}N^+$ [M - OTf]$^+$: 164.1434, Found:164.1441.

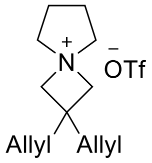

4f

2,2-Diallyl-4-azaspiro[3.4]octan-4-ium trifluoromethanesulfonate (4f) was prepared according to the General Procedure C as a pale yellow solid in 71% yield (1.2 g).

^{1}H NMR (400 MHz, DMSO-*d*$_6$) δ 5.86 – 5.75 (m, 2H), 5.24 – 5.17 (m, 4H), 4.16 (s, 4H), 3.64 – 3.61 (m, 4H), 2.42 (d, *J* = 7.2 Hz, 4H), 1.97 – 1.94 (m, 4H).

^{13}C NMR (101 MHz, DMSO-*d*$_6$) δ 132.9, 120.8 (d, *J* = 322.1 Hz), 119.7, 69.4, 64.5, 40.2, 34.1, 20.9.

^{19}F NMR (376 MHz, DMSO-*d*$_6$) δ -77.76.

HRMS (ESI) Calcd for $C_{13}H_{22}N^+$ [M - OTf]$^+$: 192.1747, Found:192.1751.

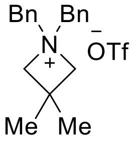

4g

1,1-Dibenzyl-3,3-dimethylazetidin-1-ium trifluoromethanesulfonate (4g) was prepared according to the General Procedure C as a pale yellow solid in 42% yield (0.9 g).

^{1}H NMR (400 MHz, DMSO-*d*$_6$) δ 7.66 (dd, *J* = 7.4, 1.9 Hz, 4H), 7.60 – 7.53 (m, 6H), 4.56 (s, 4H), 4.16 (s, 4H), 0.66 (s, 6H).

^{13}C NMR (101 MHz, DMSO-*d*$_6$) δ 133.5, 131.0, 129.6, 129.6, 68.9, 65.8, 27.3, 25.6.

^{19}F NMR (376 MHz, DMSO-d_6) δ -77.75.

HRMS (ESI) Calcd for $C_{19}H_{24}N^+$ [M - OTf]$^+$: 266.1903, Found:266.1907.

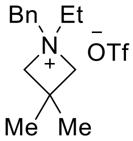

4h

1-Benzyl-1-ethyl-3,3-dimethylazetidin-1-ium trifluoromethanesulfonate (4h) was prepared according to the General Procedure C as a pale yellow solid in 62% yield (1.1 g).

^{1}H NMR (400 MHz, DMSO-d_6) δ 7.63 – 7.60 (m, 2H), 7.54 – 7.51 (m, 3H), 4.48 (s, 2H), 4.22 – 4.18 (m, 2H), 4.05 (d, *J* = 11.9 Hz, 2H), 3.36 – 3.35 (m, 2H), 1.30 (s, 6H), 1.00 – 0.98 (m, 3H).

^{13}C NMR (101 MHz, DMSO-d_6) δ 132.7, 130.8 (2C), 129.6, 71.2, 62.4, 58.1, 27.9, 27.8, 26.8, 8.4.

^{19}F NMR (376 MHz, DMSO-d_6) δ -77.74.

HRMS (ESI) Calcd for $C_{14}H_{22}N^+$ [M - OTf]$^+$: 204.1747, Found:204.1752.

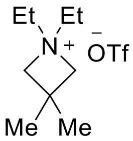

4i

1,1-Diethyl-3,3-dimethylazetidin-1-ium trifluoromethanesulfonate (4i) was prepared according to the General Procedure C as a pale yellow solid in 75% yield (1.1 g).

^{1}H NMR (400 MHz, DMSO-d_6) δ 4.01 (s, 4H), 3.37 (q, *J* = 7.0 Hz, 4H), 1.32 (s, 6H), 1.09 (t, *J* = 7.1 Hz, 6H).

^{13}C NMR (101 MHz, DMSO-d_6) δ 72.1, 55.1, 27.6, 27.5, 7.5.

^{19}F NMR (376 MHz, DMSO-d_6) δ -77.77.

HRMS (ESI) Calcd for $C_9H_{20}N^+$ [M - OTf]$^+$: 142.1590, Found: 142.1595.

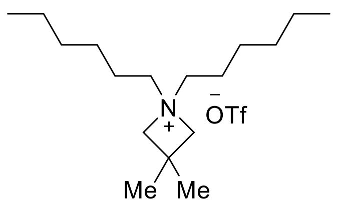

4j

1,1-Dihexyl-3,3-dimethylazetidin-1-ium trifluoromethanesulfonate (4j) was prepared according to the General Procedure C as a pale yellow solid in 73% yield (1.5 g).

^{1}H NMR (400 MHz, DMSO-*d*$_6$) δ 4.04 (s, 4H), 3.30 – 3.26 (m, 4H), 1.48 – 1.41 (m, 4H), 1.33 – 1.29 (m, 18H), 0.87 (t, *J* = 6.4 Hz, 6H).

^{13}C NMR (101 MHz, DMSO-*d*$_6$) δ 73.2, 60.4, 30.8, 27.8, 27.6, 25.3, 22.0, 21.8, 13.9.

^{19}F NMR (376 MHz, DMSO-*d*$_6$) δ -77.81.

HRMS (ESI) Calcd for $C_{17}H_{36}N^+$ [M - OTf]$^+$: 254.2842, Found: 254.2848.

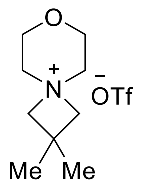

4k

2,2-Dimethyl-7-oxa-4-azaspiro[3.5]nonan-4-ium trifluoromethanesulfonate (4k) was prepared according to the General Procedure C as a pale yellow solid in 63% yield (1.1 g).

^{1}H NMR (400 MHz, DMSO-*d*$_6$) δ 4.14 (s, 4H), 3.78 – 3.75 (m, 4H), 3.54 – 3.51 (m, 4H), 1.34 (s, 6H).

^{13}C NMR (101 MHz, DMSO-*d*$_6$) δ 121.1 (d, *J* = 322.3 Hz), 73.3, 61.5, 60.8, 28.6, 27.8.

^{19}F NMR (377 MHz, DMSO-*d*$_6$) δ -77.76.

HRMS (ESI) Calcd for $C_9H_{18}NO^+$ [M - OTf]$^+$: 156.1383, Found: 156.1382.

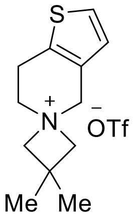

4l

3,3-Dimethyl-6',7'-dihydro-4'H-spiro[azetidine-1,5'-thieno[3,2-c]pyridin]-1-ium trifluoromethanesulfonate (4l) was prepared according to the General Procedure C as a pale yellow solid in 63% yield (1.1 g).

^{1}H NMR (400 MHz, DMSO-d_6) δ 7.51 (d, J = 5.2 Hz, 1H), 6.90 (d, J = 5.2 Hz, 1H), 4.71 (s, 2H), 4.28 (d, J = 11.7 Hz, 2H), 4.04 (d, J = 11.7 Hz, 2H), 3.83 (t, J = 6.1 Hz, 2H), 3.22 (t, J = 6.0 Hz, 2H), 1.39 (d, J = 7.9 Hz, 6H).

^{13}C NMR (101 MHz, DMSO-d_6) δ 131.1, 127.3, 125.82, 125.8, 73.0, 61.6, 58.2, 28.4, 27.6, 27.6, 20.3.

^{19}F NMR (376 MHz, DMSO-d_6) δ -77.75.

HRMS (ESI) Calcd for $C_{12}H_{18}NS^+$ [M - OTf]$^+$: 208.1154, Found: 208.1159.

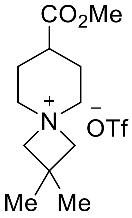

4m

7-(Methoxycarbonyl)-2,2-Dimethyl-4-azaspiro[3.5]nonan-4-ium trifluoro methanesulfonate (4m) was prepared according to the General Procedure C as a pale yellow solid in 70% yield (1.3 g).

^{1}H NMR (400 MHz, DMSO-d_6) δ 4.07 (d, J = 6.6 Hz, 4H), 3.63 – 3.61 (m, 5H), 3.37 (dd, J = 23.7, 2.9 Hz, 2H), 2.61 (tt, J = 10.6, 4.0 Hz, 1H), 1.99 – 1.95 (m, 2H), 1.85 – 1.74 (m, 2H), 1.32 (s, 6H).

^{13}C NMR (101 MHz, DMSO-d_6) δ 173.2, 120.78 (d, J = 322.3 Hz), 74.2, 70.8, 60.3, 52.0, 36.2, 28.0, 27.6, 23.0.

^{19}F NMR (376 MHz, DMSO-d_6) δ -77.77.

HRMS (ESI) Calcd for $C_{12}H_{22}NO_2^+$ [M - OTf]$^+$: 212.1645, Found:212.1650.

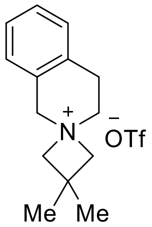

4n

3,3-Dimethyl-3',4'-dihydro-1'H-spiro[azetidine-1,2'-isoquinolin]-1-ium trifluoromethanesulfonate (4n) was prepared according to the General Procedure C as a pale yellow solid in 66% yield (1.2 g).

^{1}H NMR (400 MHz, DMSO-*d*$_6$) *δ* 7.34 – 7.20 (m, 4H), 4.77 (s, 2H), 4.28 (d, *J* = 11.6 Hz, 2H), 4.05 (d, *J* = 11.6 Hz, 2H), 3.81 (t, *J* = 6.4 Hz, 2H), 3.18 (t, *J* = 6.3 Hz, 2H), 1.40 (d, *J* = 12.4 Hz, 6H).

^{13}C NMR (101 MHz, DMSO-*d*$_6$) *δ* 130.3, 128.6, 128.3, 127.7, 127.1, 126.9, 120.8 (d, *J* = 322.3 Hz), 73.0, 62.9, 58.0, 28.3, 27.7, 27.6, 23.2.

^{19}F NMR (377 MHz, DMSO-*d*$_6$) *δ* -77.73 (d, *J* = 1.9 Hz).

HRMS (ESI) Calcd for $C_{14}H_{20}N^+$ [M - OTf]$^+$: 202.1590, Found:202.1595.

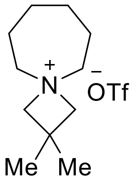

4o

2,2-Dimethyl-4-azaspiro[3.6]decan-4-ium trifluoromethanesulfonate (4o) was prepared according to the General Procedure C as a pale yellow solid in 74% yield (1.2 g).

^{1}H NMR (400 MHz, DMSO-*d*$_6$) *δ* 4.08 (s, 4H), 3.60 – 3.58 (m, 4H), 1.74 – 1.70 (m, 4H), 1.59 (dt, *J* = 5.9, 3.0 Hz, 4H), 1.33 (s, 6H).

^{13}C NMR (101 MHz, DMSO-*d*$_6$) *δ* 74.5, 66.1, 27.6, 27.6, 24.7, 21.1.

^{19}F NMR (376 MHz, DMSO-*d*$_6$) *δ* -77.78.

HRMS (ESI) Calcd for $C_{11}H_{22}N^+$ [M - OTf]$^+$: 168.1747, Found:168.1752.

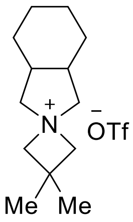

4p

3,3-Dimethyloctahydrospiro[azetidine-1,2'-isoindol]-1-ium trifluoro methanesulfonate (4p) was prepared according to the General Procedure C as a pale yellow solid in 60% yield (1.0 g).

^{1}H NMR (400 MHz, DMSO-*d*$_6$) δ 4.28 (d, *J* = 43.8 Hz, 4H), 3.89 (dd, *J* = 11.7, 6.4 Hz, 2H), 3.61 (dd, *J* = 11.8, 7.0 Hz, 2H), 2.29 (t, *J* = 10.6 Hz, 2H), 1.61 – 1.54 (m, 2H), 1.45 – 1.36 (m, 2H), 1.30 (s, 10H).

^{13}C NMR (101 MHz, DMSO-*d*$_6$) δ 78.7, 77.2, 70.4, 35.9, 29.3, 26.4, 24.2, 21.6.

^{19}F NMR (376 MHz, DMSO-*d*$_6$) δ -77.78.

HRMS (ESI) Calcd for $C_{13}H_{24}N^+$ [M - OTf]$^+$: 194.1903, Found:168.194.1908.

III. Intramolecular Asymmetric Opening of Azetidiniums

Table S1. Effect of the Alcohol Additive in the Reaction of 1n[a]

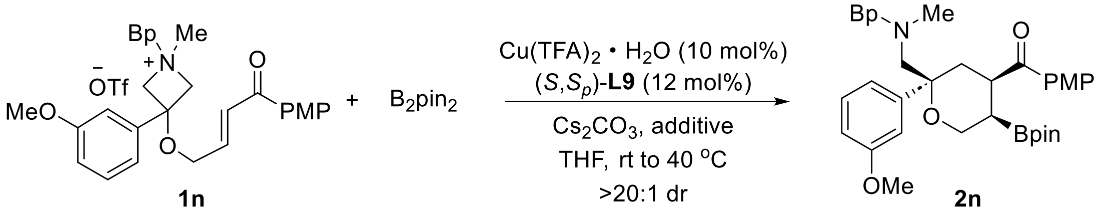

entry	additive	NMR yield (%)	ee (%)
1	–	69	94
2	MeOH	92	94
3	^{t}BuOH	>95	94

[a]Reaction conditions: **1n** (0.06 mmol), B_2pin_2 (0.05 mmol), $Cu(TFA)_2 \cdot H_2O$ (10 mol%), (S,S_p)-**L9** (12 mol%), Cs_2CO_3 (0.1 mmol), additive (0.1 mmol), THF (2.0 mL), rt to 40 °C. The ee value was determined by chiral HPLC analysis. The dr value was determined by ^{1}H NMR analysis of the crude product.

General Procedure D.

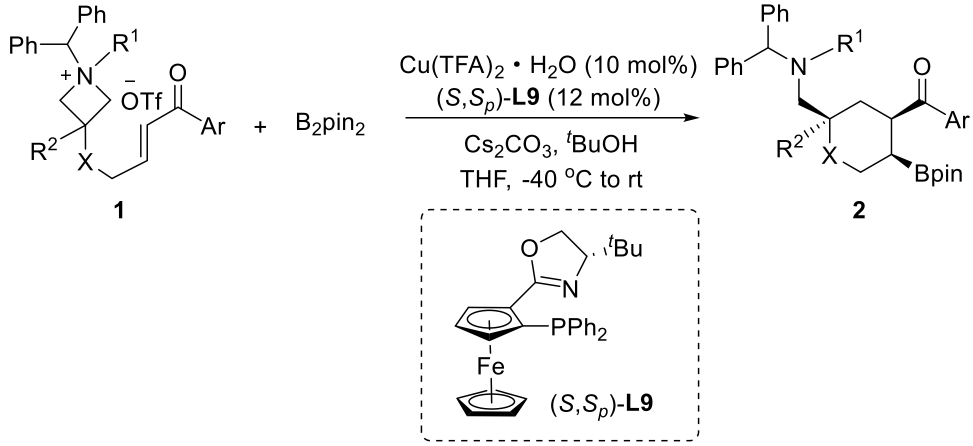

In a glove box, a mixture of $Cu(TFA)_2{\cdot}H_2O$ (5.8 mg, 0.02 mmol, 10 mol%) and the chiral ligand (S,S_p)-**L9** (11.9 mg, 0.024 mmol, 12 mol%) in dry THF (0.2 M, 1.0 mL) was stirred at room temperature for 0.5 h. Then, azetidinium **1** (0.24 mmol, 1.2 equiv), Cs_2CO_3 (130.3 mg, 0.4 mmol, 2.0 equiv), and ^{t}BuOH (38.3 uL,

0.4 mmol, 2.0 equiv) were added sequentially. The mixture was cooled to -40 °C and stirred for 10 min before a solution of B_2pin_2 (50.7 mg, 0.2 mmol, 1.0 equiv) in dry THF (1.0 mL) was added dropwise. The mixture was stirred at -40 °C for 0.5 h before it was warmed to room temperature and stirred for 5 h. Next, the mixture was concentrated *in vacuo*, and the residue was purified by column chromatography on silica gel to give the desired product **2**.

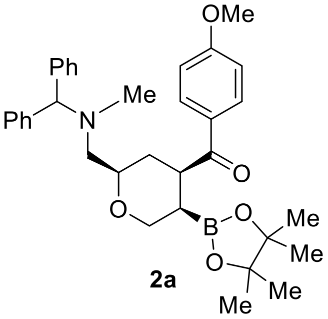

2a

((2*R*,4*R*,5*R*)-2-((Benzhydryl(methyl)amino)methyl)-5-(4,4,5,5-tetramethyl-1,3,2-dioxaborolan-2-yl)tetrahydro-2*H*-pyran-4-yl)(4-methoxyphenyl) methanone (2a) was prepared according to the General Procedure D as a white solid (chromatography eluent: *n*-hexane/EtOAc = 10:1) in 73% yield (81.0 mg, 90% ee, >20:1 dr).

$[\alpha]_D^{25}$: +3.7 (*c* = 2.7, $CHCl_3$). HPLC analysis of the product: Daicel CHIRALPAK® AD3 column; 10% *i*-PrOH in *n*-hexane; 1.0 mL/min; retention times: 9.5 min (major), 5.4 min (minor).

^{1}H NMR (400 MHz, $CDCl_3$) δ 7.87 (d, *J* = 8.9 Hz, 2H), 7.38 (t, *J* = 7.4 Hz, 4H), 7.22 (t, *J* = 7.5 Hz, 4H), 7.20 – 7.09 (m, 2H), 6.93 (d, *J* = 8.9 Hz, 2H), 4.46 (s, 1H), 4.18 (d, *J* = 10.6 Hz, 1H), 3.85 (s, 3H), 3.70 (dd, *J* = 11.2, 3.1 Hz, 1H), 3.64 (dq, *J* = 10.6, 4.0, 3.2 Hz, 1H), 3.43 (dt, *J* = 12.1, 3.9 Hz, 1H), 2.60 (dd, *J* = 13.0, 6.3 Hz, 1H), 2.35 (dd, *J* = 13.0, 4.7 Hz, 1H), 2.24 (s, 3H), 2.03 – 1.91 (m, 1H), 1.85 (d, *J* = 13.3 Hz, 1H), 1.40 – 1.36 (m, 1H), 1.20 (s, 12H).

^{13}C NMR (101 MHz, $CDCl_3$) δ 200.6, 163.0, 142.7, 142.5, 130.6, 128.9, 128.4, 128.3, 128.1, 126.7, 126.6, 113.6, 82.8, 76.3, 75.6, 69.1, 59.8, 55.4, 46.3, 41.3, 31.1, 24.8, 24.5.

HRMS (ESI) Calcd for $C_{34}H_{43}BNO_5^+$ $[M + H]^+$: 556.3229, Found: 556.3256.

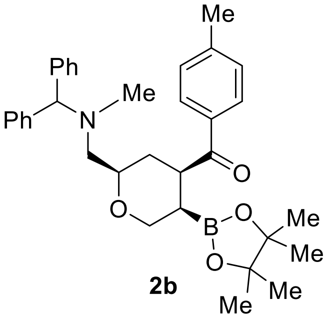

2b

((2*R*,4*R*,5*R*)-2-((Benzhydryl(methyl)amino)methyl)-5-(4,4,5,5-tetramethyl-1,3,2-dioxaborolan-2-yl)tetrahydro-2*H*-pyran-4-yl)(*p*-tolyl)methanone (2b) was prepared according to the General Procedure D as a white solid (chromatography eluent: *n*-hexane/EtOAc = 10:1) in 73% yield (78.7 mg, 90% ee, >20:1 dr).

$[\alpha]_D^{25}$: +9.0 (*c* = 2.6, $CHCl_3$). HPLC analysis of the product: Daicel CHIRALPAK® AD3 column; 10% *i*-PrOH in *n*-hexane; 1.0 mL/min; retention times: 7.5 min (major), 4.4 min (minor).

¹H NMR (400 MHz, CDC_3) δ 7.76 (d, *J* = 8.2 Hz, 1H), 7.37 (t, *J* = 7.4 Hz, 3H), 7.28 – 7.18 (m, 6H), 7.15 (td, *J* = 7.3, 3.2 Hz, 2H), 4.45 (s, 1H), 4.18 (d, *J* = 11.1 Hz, 1H), 3.70 (dd, *J* = 11.2, 3.1 Hz, 1H), 3.62 (dt, *J* = 10.6, 4.4 Hz, 1H), 3.43 (dt, *J* = 11.7, 4.1 Hz, 1H), 2.59 (dd, *J* = 13.0, 6.4 Hz, 1H), 2.41 (s, 2H), 2.34 (dd, *J* = 13.0, 4.7 Hz, 1H), 2.23 (s, 2H), 2.00 – 1.81 (m, 2H), 1.43 (s, 1H), 1.19 (s, 7H).

¹³C NMR (101 MHz, $CDCl_3$) δ 201.9, 143.1, 142.7, 142.5, 133.6, 129.1, 128.4, 128.4, 128.3, 128.1, 126.7, 126.6, 82.9, 76.3, 75.6, 69.2, 59.8, 46.4, 41.4 (2C), 30.9, 24.8, 24.5, 21.6.

HRMS (ESI) Calcd for $C_{34}H_{43}BNO_4^+$ $[M + H]^+$: 540.3280, Found: 540.3290.

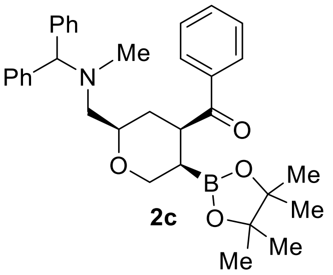

2c

((2*R*,4*R*,5*R*)-2-((Benzhydryl(methyl)amino)methyl)-5-(4,4,5,5-tetramethyl-1,3,2-dioxaborolan-2-yl)tetrahydro-2*H*-pyran-4-yl)(phenyl)methanone (2c) was prepared according to the General Procedure D for as a white solid (chromatography eluent: *n*-hexane/EtOAc = 10:1) in 72% yield (75.6 mg, 90% ee, >20:1 dr).

$[\alpha]_D^{25}$: +13.5 (*c* = 3.8, $CHCl_3$). HPLC analysis of the product: Daicel CHIRALPAK® AD3 column; 10% *i*-PrOH in *n*-hexane; 1.0 mL/min; retention times: 6.2 min (major), 4.3 min (minor).

^{1}H NMR (400 MHz, $CDCl_3$) δ 7.84 (d, *J* = 7.5 Hz, 2H), 7.53 (t, *J* = 7.3 Hz, 1H), 7.45 (t, *J* = 7.5 Hz, 2H), 7.37 (t, *J* = 7.6 Hz, 4H), 7.22 (t, *J* = 7.5 Hz, 4H), 7.18 – 7.11 (m, 2H), 4.45 (s, 1H), 4.19 (d, *J* = 11.1 Hz, 1H), 3.77 – 3.53 (m, 2H), 3.43 (dt, *J* = 10.7, 4.3 Hz, 1H), 2.59 (dd, *J* = 13.0, 6.3 Hz, 1H), 2.35 (dd, *J* = 13.0, 4.5 Hz, 1H), 2.24 (s, 3H), 1.94 – 1.86 (m, 2H), 1.46 (s, 1H), 1.19 (s, 12H).

^{13}C NMR (101 MHz, $CDCl_3$) δ 202.4, 142.8, 142.5, 136.4, 132.4, 128.4, 128.4, 128.29, 128.28, 128.2, 126.7, 126.7, 83.0, 76.4, 75.7, 69.2, 59.8, 46.4, 41.4, 30.8, 24.8, 24.5.

HRMS (ESI) Calcd for $C_{33}H_{41}BNO_4^+$ $[M + H]^+$: 526.3123, Found: 526.3135.

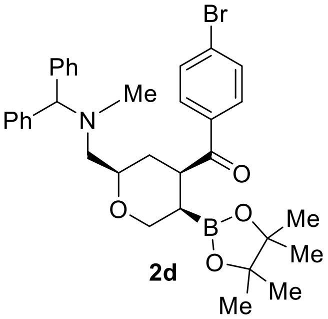

2d

((2*R*,4*R*,5*R*)-2-((Benzhydryl(methyl)amino)methyl)-5-(4,4,5,5-tetramethyl-1,3,2-dioxaborolan-2-yl)tetrahydro-2*H*-pyran-4-yl)(4-bromophenyl) methanone (2d) was prepared according to the General Procedure D as a white solid (chromatography eluent: *n*-hexane/EtOAc = 10:1) in 70% yield (84.4 mg, 93% ee, 16:1 dr).

$[\alpha]_D^{25}$: +10.2 (*c* = 2.2, $CHCl_3$). HPLC analysis of the product: Daicel CHIRALPAK® AD3 column; 10% *i*-PrOH in *n*-hexane; 1.0 mL/min; retention times: 6.6 min (major), 4.4 min (minor).

^{1}H NMR (400 MHz, $CDCl_3$) δ 7.70 (d, *J* = 8.5 Hz, 2H), 7.59 (d, *J* = 8.5 Hz, 2H), 7.37 (t, *J* = 7.0 Hz, 4H), 7.23 (t, *J* = 7.5 Hz, 4H), 7.17 – 7.13 (m, 2H), 4.45 (s, 1H), 4.19 (d, *J* = 11.2 Hz, 1H), 3.68 (dd, *J* = 11.2, 3.1 Hz, 1H), 3.65 – 3.57 (m, 1H), 3.37 (dt, *J* = 11.2, 4.4 Hz, 1H), 2.58 (dd, *J* = 13.0, 6.2 Hz, 1H), 2.35 (dd, *J* = 13.0, 4.8 Hz, 1H), 2.23 (s, 3H), 1.91 – 1.83 (m, 2H), 1.46 – 1.41 (m, 1H), 1.21 (s, 12H).

^{13}C NMR (101 MHz, $CDCl_3$) δ 201.3, 142.7, 142.5, 135.1, 131.8, 129.8, 128.32, 128.28, 128.2, 127.3, 126.7, 126.7, 83.1, 76.3, 75.7, 69.2, 59.8, 46.4, 41.4, 30.7, 24.7, 24.5.

HRMS (ESI) Calcd for $C_{33}H_{40}BBrNO_4^+$ $[M + H]^+$: 604.2228, Found: 604.2227.

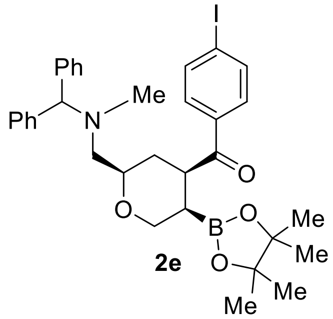

((2*R*,4*R*,5*R*)-2-((Benzhydryl(methyl)amino)methyl)-5-(4,4,5,5-tetramethyl-1,3,2-dioxaborolan-2-yl)tetrahydro-2*H*-pyran-4-yl)(4-iodophenyl)methanone (2e) was prepared according to the General Procedure D for as a white solid (chromatography eluent: *n*-hexane/EtOAc = 10:1) in 45% yield (58.6 mg, 88% ee, >20:1 dr).

$[\alpha]_D^{25}$: +4.9 (c = 1.8, $CHCl_3$). HPLC analysis of the product: Daicel CHIRALPAK® AD3 column; 10% *i*-PrOH in *n*-hexane; 1.0 mL/min; retention times: 6.9 min (major), 4.7 min (minor).

^{1}H NMR (400 MHz, $CDCl_3$) δ 7.81 (d, J = 8.5 Hz, 2H), 7.55 (d, J = 8.5 Hz, 2H), 7.37 (t, J = 6.9 Hz, 4H), 7.23 (t, J = 7.5 Hz, 4H), 7.19 – 7.10 (m, 2H), 4.45 (s, 1H), 4.19 (d, J = 12.1 Hz, 1H), 3.68 (dd, J = 11.2, 3.0 Hz, 1H), 3.62 (dt, J = 8.8, 4.5 Hz, 1H), 3.36 (dt, J = 10.7, 4.5 Hz, 1H), 2.58 (dd, J = 13.0, 6.3 Hz, 1H), 2.35 (dd, J = 13.0, 4.7 Hz, 1H), 2.23 (s, 3H), 1.92 – 1.80 (m, 2H), 1.43 (s, 1H), 1.18 (s, 12H).

^{13}C NMR (101 MHz, $CDCl_3$) δ 201.6, 142.7, 142.5, 137.7, 135.7, 129.8, 128.3, 128.3, 128.28, 128.15, 126.73, 126.67, 100.0, 83.1, 76.3, 75.7, 69.2, 59.8, 46.3, 41.4, 30.7, 24.7, 24.5.

HRMS (ESI) Calcd for $C_{33}H_{40}BINO_4^+$ [M + H]$^+$: 652.2090, Found: 652.2114.

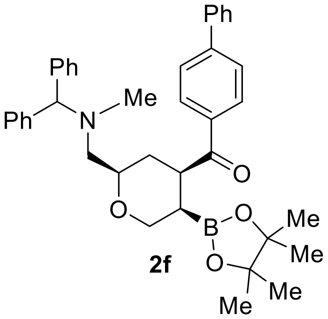

2f

[1,1'-Biphenyl]-4-yl((2*R*,4*R*,5*R*)-2-((benzhydryl(methyl)amino)methyl)-5-(4,4,5,5-tetramethyl-1,3,2-dioxaborolan-2-yl)tetrahydro-2*H*-pyran-4-yl) methanone (2f) was prepared according to the General Procedure D as a white solid (chromatography eluent: *n*-hexane/EtOAc = 10:1) in 70% yield (84.2 mg, 85% ee, 8:1 dr).

$[\alpha]_D^{25}$: +2.0 (c = 2.8, $CHCl_3$). HPLC analysis of the product: Daicel CHIRALPAK® AD3 column; 10% *i*-PrOH in *n*-hexane; 1.0 mL/min; retention times: 5.9 min (major), 5.3 min (minor).

^{1}H NMR (400 MHz, $CDCl_3$) δ 7.93 (d, *J* = 8.4 Hz, 2H), 7.69 – 7.57 (m, 4H), 7.48 (t, *J* = 7.5 Hz, 2H), 7.39 (q, *J* = 7.2 Hz, 5H), 7.27 – 7.19 (m, 4H), 7.17 – 7.13 (m, 2H), 4.46 (s, 1H), 4.21 (d, *J* = 11.0 Hz, 1H), 3.72 (dd, *J* = 11.2, 3.1 Hz, 1H), 3.70 – 3.60 (m, 1H), 3.48 (dt, *J* = 11.4, 4.3 Hz, 1H), 2.61 (dd, *J* = 13.0, 6.3 Hz, 1H), 2.36 (dd, *J* = 13.0, 4.6 Hz, 1H), 2.25 (s, 3H), 1.94 (q, *J* = 11.4 Hz, 2H), 1.48 (s, 1H), 1.20 (s, 12H).

^{13}C NMR (101 MHz, $CDCl_3$) δ 201.9, 145.1, 142.8, 142.5, 140.0, 135.0, 128.9, 128.4, 128.3, 128.2, 128.1, 127.3, 127.2, 127.1, 126.71, 126.66, 83.0, 76.4, 75.7, 69.2, 59.9, 46.5, 41.4, 30.9, 24.8, 24.5.

HRMS (ESI) Calcd for $C_{39}H_{45}BNO_4^+$ $[M + H]^+$: 602.3436, Found: 602.3460.

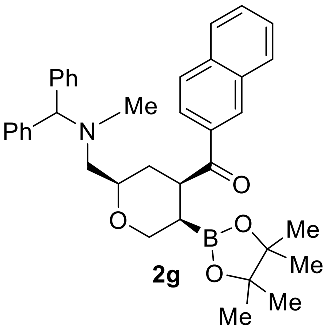

2g

((2*R*,4*R*,5*R*)-2-((Benzhydryl(methyl)amino)methyl)-5-(4,4,5,5-tetramethyl-1,3,2-dioxaborolan-2-yl)tetrahydro-2*H*-pyran-4-yl)(naphthalen-2-yl)methanone (2g) was prepared according to the General Procedure D for 5 h as a white solid (chromatography eluent: *n*-hexane/EtOAc = 10:1) in 60% yield (69.0 mg, 90% ee, 13:1 dr).

$[\alpha]_D^{25}$: -5.8 (c = 2.3, $CHCl_3$). HPLC analysis of the product: Daicel CHIRALPAK® AD3 column; 10% *i*-PrOH in *n*-hexane; 1.0 mL/min; retention times: 10.9 min (major), 5.4 min (minor).

^{1}H NMR (400 MHz, $CDCl_3$) δ 8.35 (s, 1H), 7.98 – 7.83 (m, 4H), 7.64 – 7.50 (m, 2H), 7.37 (t, J = 8.0 Hz, 4H), 7.29 – 7.08 (m, 6H), 4.46 (s, 1H), 4.22 (d, J = 10.5 Hz, 1H), 3.76 (dd, J = 11.2, 3.0 Hz, 1H), 3.69 (dq, J = 10.4, 5.7 Hz, 1H), 3.60 (dt, J = 10.0, 4.7 Hz, 1H), 2.61 (dd, J = 13.0, 6.3 Hz, 1H), 2.36 (dd, J = 13.0, 4.6 Hz, 1H), 2.25 (s, 3H), 2.00 – 1.95 (m, 2H), 1.55 – 1.51 (m, 1H), 1.20 (d, J = 2.9 Hz, 12H).

^{13}C NMR (101 MHz, $CDCl_3$) δ 202.3, 142.7, 142.5, 135.3, 133.7, 132.5, 129.4, 129.3, 128.4, 128.3, 128.1, 127.7, 126.7, 126.6, 124.6, 83.0, 76.4, 75.6, 69.2, 59.8, 46.5, 41.4, 31.0, 24.8, 24.5.

HRMS (ESI) Calcd for $C_{37}H_{43}BNO_4^+$ $[M + H]^+$: 576.3280, Found: 576.3293.

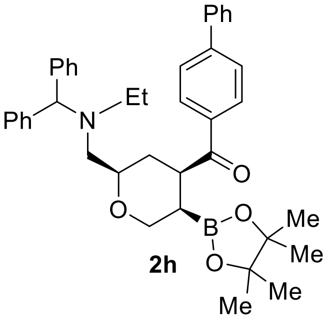

2h

[1,1'-Biphenyl]-4-yl((2*R*,4*R*,5*R*)-2-((benzhydryl(ethyl)amino)methyl)-5-(4,4,5,5-tetramethyl-1,3,2-dioxaborolan-2-yl)tetrahydro-2*H*-pyran-4-yl)methanone (2h) was prepared according to the General Procedure D as a white solid (chromatography eluent: *n*-hexane/EtOAc = 10:1) in 56% yield (68.9 mg, 88% ee, 13:1 dr).

$[\alpha]_D^{25}$: +3.2 (c = 2.3, $CHCl_3$). HPLC analysis of the product: Daicel CHIRALPAK® AD3 column; 10% *i*-PrOH in *n*-hexane; 1.0 mL/min; retention times: 4.9 min (major), 4.6 min (minor).

^{1}H NMR (400 MHz, $CDCl_3$) δ 7.92 (d, J = 8.1 Hz, 2H), 7.65 (dd, J = 13.0, 7.9 Hz, 4H), 7.47 (t, J = 7.4 Hz, 2H), 7.41 – 7.36 (m , 5H), 7.24 (t, J = 7.4 Hz, 3H), 7.18 – 7.16 (m, 2H), 4.93 (s, 1H), 4.17 (d, J = 11.1 Hz, 1H), 3.68 (dd, J = 11.2, 2.6 Hz, 1H), 3.55 – 3.48 (m, 1H), 3.43 (dt, J = 11.1, 3.8 Hz, 1H), 2.61 (dddd, J = 51.5, 23.4, 13.7, 5.6 Hz, 4H), 2.00 – 1.78 (m, 2H), 1.45 (s, 1H), 1.20 (s, 12H), 0.98 (t, J = 7.0 Hz, 3H).

^{13}C NMR (101 MHz, $CDCl_3$) δ 201.9, 145.1, 142.6, 142.3, 140.0, 135.0, 128.9, 128.9, 128.7, 128.1, 128.0, 127.2, 127.1, 126.6, 82.9, 77.1, 70.9, 69.2, 54.8, 46.7, 44.8, 31.2, 24.8, 24.6, 11.3.

HRMS (ESI) Calcd for $C_{40}H_{47}BNO_4^+$ $[M + H]^+$: 616.3593, Found: 616.3599.

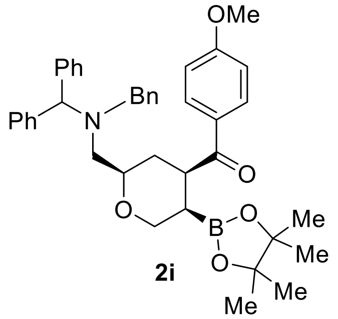

2i

((2*R*,4*R*,5*R*)-2-((Benzhydryl(benzyl)amino)methyl)-5-(4,4,5,5-tetramethyl-1,3,2-dioxaborolan-2-yl)tetrahydro-2*H*-pyran-4-yl)(4-methoxyphenyl) methanone (2i) was prepared according to the General Procedure D as a white solid (chromatography eluent: *n*-hexane/EtOAc = 10:1) in 73% yield (92.1 mg, 90% ee, >20:1 dr).

$[\alpha]_D^{25}$: +5.1 (c = 3.3, $CHCl_3$). HPLC analysis of the product: Daicel CHIRALPAK® AD3 column; 10% *i*-PrOH in *n*-hexane; 1.0 mL/min; retention times: 7.4 min (major), 4.4 min (minor).

^{1}H NMR (400 MHz, $CDCl_3$) δ 7.84 (d, J = 8.9 Hz, 2H), 7.46 – 7.32 (m, 6H), 7.31 – 7.23 (m, 6H), 7.23 – 7.16 (m, 3H), 6.93 (d, J = 8.9 Hz, 2H), 5.09 (s, 1H), 4.13 (d, J = 11.0 Hz, 1H), 3.86 (s, 3H), 3.78 (d, J = 14.1 Hz, 1H), 3.67 – 3.57 (m, 1H), 3.55 – 3.46 (m, 1H), 3.34 (dt, J = 12.2, 3.9 Hz, 1H), 2.70 (dd, J = 13.7, 6.2 Hz, 1H), 2.52 (dd, J = 13.7, 4.4 Hz, 1H), 1.94 (q, J = 12.4, 12.0 Hz, 1H), 1.81 (d, J = 13.3 Hz, 1H), 1.34 (s, 1H), 1.13 (d, J = 1.5 Hz, 12H).

^{13}C NMR (101 MHz, $CDCl_3$) δ 200.6, 163.0, 141.2, 140.7, 140.3, 130.6, 129.4, 129.2, 128.8, 128.1, 127.91, 127.90, 126.74, 126.66, 126.6, 113.6, 82.7, 69.1, 69.0, 55.6, 55.4, 54.7, 46.5, 31.1, 24.7, 24.4.

HRMS (ESI) Calcd for $C_{40}H_{47}BNO_5^+$ [M + H]$^+$: 632.3542, Found: 632.3552.

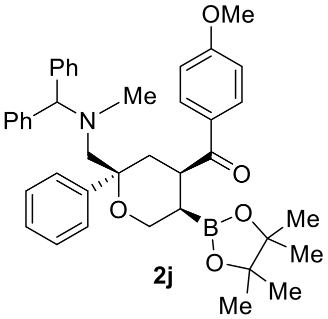

2j

((2*S*,4*R*,5*R*)-2-((Benzhydryl(methyl)amino)methyl)-2-phenyl-5-(4,4,5,5-tetramethyl-1,3,2-dioxaborolan-2-yl)tetrahydro-2*H*-pyran-4-yl)(4-methoxyphenyl)methanone (2j) was prepared according to the General Procedure D with slight modifications (the mixture was stirred at -40 °C for 0.5 h before it was warmed to 40 °C and stirred for 5 h) as a white solid (chromatography eluent: *n*-hexane/EtOAc = 10:1) in 77% yield (97.2 mg, 88% ee, >20:1 dr).

$[\alpha]_D^{25}$: +56.7 (*c* = 3.2, $CHCl_3$). HPLC analysis of the product: Daicel CHIRALPAK® AD3 column; 10% *i*-PrOH in *n*-hexane 1.0 mL/min; retention times: 5.7 min (major), 5.3 min (minor).

^{1}H NMR (400 MHz, $CDCl_3$) δ 7.84 (d, *J* = 8.8 Hz, 2H), 7.38 (dt, *J* = 15.2, 7.6 Hz, 4H), 7.28 (t, *J* = 7.0 Hz, 1H), 7.26 – 7.02 (m, 10H), 6.93 (d, *J* = 8.8 Hz, 2H), 4.39 (s, 1H), 3.97 – 3.73 (m, 5H), 3.41 (dt, *J* = 12.8, 3.3 Hz, 1H), 2.86 (t, *J* = 13.5 Hz, 1H), 2.57 (d, *J* = 13.9 Hz, 1H), 2.44 (d, *J* = 13.9 Hz, 1H), 2.34 – 2.30 (m, 4H), 1.34 – 1.30 (m, 13H).

^{13}C NMR (101 MHz, $CDCl_3$) *δ* 201.7, 163.1, 143.1, 142.7, 142.6, 130.5, 129.0, 128.5, 128.40, 128.38, 128.0, 127.9, 127.0, 126.8, 126.5, 113.7, 82.8, 81.6, 75.9, 67.4, 63.1, 55.4, 42.5, 42.2, 31.3, 24.9, 24.7.

HRMS (ESI) Calcd for $C_{40}H_{47}BNO_5^+$ $[M + H]^+$: 632.3542, Found: 632.3554.

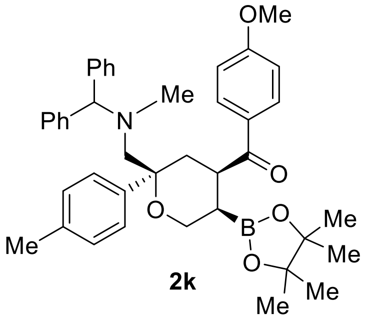

2k

((2*S*,4*R*,5*R*)-2-((Benzhydryl(methyl)amino)methyl)-5-(4,4,5,5-tetramethyl-1,3,2-dioxaborolan-2-yl)-2-(*p*-tolyl)tetrahydro-2*H*-pyran-4-yl)(4-meth oxyphenyl)methanone (2k) was prepared according to the General Procedure D with slight modifications (the mixture was stirred at -40 °C for 0.5 h before it was warmed to 40 °C and stirred for 5 h) as a white solid (chromatography eluent: *n*-hexane/EtOAc = 10:1) in 67% yield (86.4 mg, 93% ee, >20:1 dr).

$[\alpha]_D^{25}$: +65.6 (*c* = 2.9, $CHCl_3$). HPLC analysis of the product: Daicel CHIRALPAK® AD3 column; 10% *i*-PrOH in *n*-hexane; 1.0 mL/min; retention times: 6.0 min (major), 4.8 min (minor).

^{1}H NMR (400 MHz, $CDCl_3$) δ 7.85 (d, *J* = 8.9 Hz, 2H), 7.31 – 7.04 (m, 15H), 6.94 (d, *J* = 8.9 Hz, 2H), 4.39 (s, 1H), 3.90 – 3.87 (m, 4H), 3.80 (dd, *J* = 11.5, 3.6 Hz, 1H), 3.42 (dt, *J* = 13.0, 3.3 Hz, 1H), 2.89 (t, *J* = 13.5 Hz, 1H), 2.55 (d, *J* = 13.9 Hz, 1H), 2.41 (d, *J* = 13.9 Hz, 1H), 2.37 (s, 3H), 2.30 – 2.27 (m, 4H), 1.35 – 1.31 (m, 13H).

^{13}C NMR (101 MHz, $CDCl_3$) δ 201.8, 163.1, 143.2, 142.8, 139.5, 136.3, 130.6, 129.2, 129.0, 128.5, 128.4, 128.0, 127.9, 126.9, 126.5, 113.7, 82.8, 81.5, 75.9, 67.6, 63.0, 55.4, 42.5, 42.3, 31.3, 24.9, 24.7, 21.0.

HRMS (ESI) Calcd for $C_{41}H_{49}BNO_5^+$ $[M + H]^+$: 646.3698, Found: 646.3721.

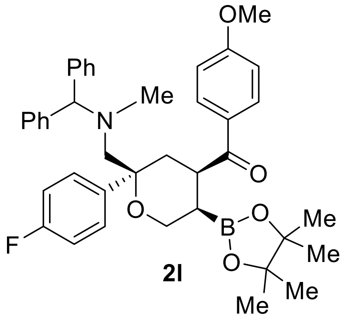

2l

((2*S*,4*R*,5*R*)-2-((Benzhydryl(methyl)amino)methyl)-2-(4-fluorophenyl)-5-(4,4,5,5-tetramethyl-1,3,2-dioxaborolan-2-yl)tetrahydro-2*H*-pyran-4-yl)(4-methoxyphenyl)methanone (2l) was prepared according to the General Procedure D with slight modifications (the mixture was stirred at -40 °C for 0.5 h before it was warmed to 40 °C and stirred for 5 h) as a white solid (chromatography eluent: *n*-hexane/EtOAc = 10:1) in 75% yield (97.4 mg, 84% ee, >20:1 dr).

$[\alpha]_D^{25}$: +53.4 (*c* = 3.3, $CHCl_3$). HPLC analysis of the product: Daicel CHIRALPAK® AD3 column; 10% *i*-PrOH in *n*-hexane; 1.0 mL/min; retention times: 6.5 min (major), 5.3 min (minor).

^{1}H NMR (400 MHz, $CDCl_3$) δ 7.81 (d, *J* = 8.8 Hz, 2H), 7.30 (dd, *J* = 8.2, 5.5 Hz, 2H), 7.23 – 7.01 (m, 12H), 6.93 (d, *J* = 8.8 Hz, 1H), 4.39 (s, 1H), 3.92 – 3.87 (m, 4H), 3.75 (dd, *J* = 11.5, 3.6 Hz, 1H), 3.38 (dt, *J* = 12.8, 3.4 Hz, 1H), 2.78 (t, *J* = 13.5 Hz, 1H), 2.55 (d, *J* = 13.9 Hz, 1H), 2.42 (d, *J* = 13.8 Hz, 1H), 2.28 – 2.25 (m, 4H), 1.30 1.28 (m, 13H).

^{13}C NMR (101 MHz, $CDCl_3$) δ 201.5, 163.1, 163.0, 160.6, 143.1, 142.6, 138.19, 138.16, 130.5, 129.0, 128.7, 128.6, 128.5, 128.4, 128.00, 127.98, 126.6, 126.5, 115.3, 115.1, 113.7, 82.9, 81.2, 75.9, 67.4, 63.1, 55.4, 42.6, 42.1, 31.5, 24.9, 24.7.

^{19}F NMR (377 MHz, $CDCl_3$) δ -116.34.

HRMS (ESI) Calcd for $C_{40}H_{46}BFNO_5^+$ $[M + H]^+$: 650.3448, Found: 650.3459.

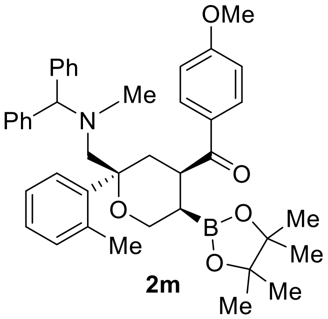

2m

((2*S*,4*R*,5*R*)-2-((Benzhydryl(methyl)amino)methyl)-5-(4,4,5,5-tetramethyl-1,3,2-dioxaborolan-2-yl)-2-(o-tolyl)tetrahydro-2*H*-pyran-4-yl)(4-methoxyphenyl)methanone (2m) was prepared according to the General Procedure D with slight modifications (the mixture was stirred at -40 °C for 0.5 h before it was warmed to 40 °C and stirred for 5 h) as a white solid (chromatography eluent: *n*-hexane/EtOAc = 10:1) in 65% yield (83.9 mg, 92% ee, >20:1 dr).

$[\alpha]_D^{25}$: +34.4 (*c* = 2.8, $CHCl_3$). HPLC analysis of the product: Daicel CHIRALPAK® IE3 column; 10% *i*-PrOH in *n*-hexane; 1.0 mL/min; retention times: 6.9 min (major), 8.1 min (minor).

^{1}H NMR (400 MHz, $CDCl_3$) δ 7.86 (d, *J* = 8.2 Hz, 2H), 7.35 – 7.02 (m, 15H), 6.94 (d, *J* = 8.7 Hz, 2H), 4.37 (s, 1H), 3.91 – 3.87 (s, 4H), 3.63 – 3.54 (m, 1H), 2.91 (t, *J* = 13.7 Hz, 1H), 2.62 – 2.60 (m, 1H), 2.56 – 2.39 (m, 3H), 2.30 (s, 3H), 1.28 – 1.25 (m, 13H).

^{13}C NMR (101 MHz, $CDCl_3$) δ 202.1, 163.0, 143.0, 133.2, 130.5, 129.4, 128.5, 128.4, 128.01, 127.98, 126.9, 126.6, 126.5, 125.9, 113.6, 83.7, 82.9, 76.2, 64.5, 63.0, 55.5, 42.8, 42.1, 31.9, 24.9, 24.7, 22.2.

HRMS (ESI) Calcd for $C_{41}H_{49}BNO_5^+$ $[M + H]^+$: 646.3698, Found: 646.3708.

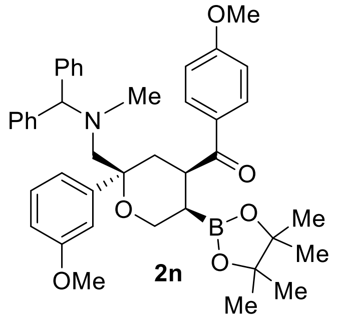
2n

((2*S*,4*R*,5*R*)-2-((Benzhydryl(methyl)amino)methyl)-2-(3-methoxyphenyl)-5-(4,4,5,5-tetramethyl-1,3,2-dioxaborolan-2-yl)tetrahydro-2*H*-pyran-4-yl)(4-methoxyphenyl)methanone (2n) was prepared according to the General Procedure D with slight modifications (the mixture was stirred at -40 °C for 0.5 h before it was warmed to 40 °C and stirred for 5 h) as a white solid (chromatography eluent: *n*-hexane/EtOAc = 10:1) in 77% yield (101.8 mg, 83% ee, >20:1 dr).

$[\alpha]_D^{25}$: +56.7 (*c* = 3.4, $CHCl_3$). HPLC analysis of the product: Daicel CHIRALPAK® AY3 column; 10% *i*-PrOH in *n*-hexane; 1.0 mL/min; retention times: 6.5 min (major), 4.9 min (minor).

^{1}H NMR (400 MHz, $CDCl_3$) δ 7.87 (d, *J* = 8.9 Hz, 2H), 7.30 (t, *J* = 8.0 Hz, 1H), 7.26 – 7.04 (m, 10H), 6.96 – 6.87 (m, 4H), 6.83 (dd, *J* = 8.0, 2.3 Hz, 1H), 4.39 (s, 1H), 3.92 (d, *J* = 11.5 Hz, 1H), 3.85 – 3.82 (m, 4H), 3.78 (s, 3H), 3.43 (dt, *J* = 12.9, 3.3 Hz, 1H), 2.88 (t, *J* = 13.5 Hz, 1H), 2.58 (d, *J* = 13.9 Hz, 1H), 2.43 (d, *J* = 13.9 Hz, 1H), 2.31 – 2.28 (m, 4H), 1.36 – 1.32 (m , 13H).

^{13}C NMR (101 MHz, $CDCl_3$) δ 201.6, 163.1, 159.9, 144.5, 143.2, 142.7, 130.6, 129.4, 128.9, 128.5, 128.4, 128.0, 127.9, 126.52, 126.48, 119.2, 113.6, 112.5, 112.4, 82.8, 81.6, 75.8, 67.4, 63.2, 55.4, 55.1, 42.5, 42.3, 31.5, 24.9, 24.7.

HRMS (ESI) Calcd for $C_{41}H_{49}BNO_6^+$ $[M + H]^+$: 662.3647, Found: 662.3660.

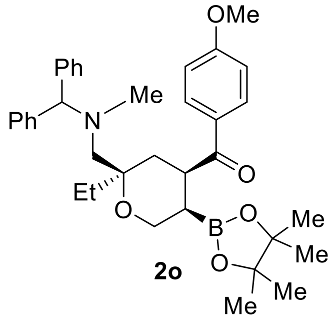

2o

((2*R*,4*R*,5*R*)-2-((Benzhydryl(methyl)amino)methyl)-2-ethyl-5-(4,4,5,5-tetra methyl-1,3,2-dioxaborolan-2-yl)tetrahydro-2*H*-pyran-4-yl)(4-methoxy phenyl)methanone (2o) was prepared according to the General Procedure D with slight modifications (the mixture was stirred at -40 °C for 0.5 h before it was warmed to 40 °C and stirred for 5 h) as a white solid (chromatography eluent: *n*-hexane/EtOAc = 10:1) in 51% yield (59.6 mg, 91% ee, >20:1 dr).

$[\alpha]_D^{25}$: -0.3 (c = 1.9, $CHCl_3$). HPLC analysis of the product: Daicel CHIRALPAK® AD3 column; 10% *i*-PrOH in *n*-hexane; 1.0 mL/min; retention times: 6.3 min (major), 4.8 min (minor).

^{1}H NMR (400 MHz, $CDCl_3$) δ 7.86 (d, J = 8.9 Hz, 2H), 7.33 (t, J = 8.4 Hz, 4H), 7.27 – 7.10 (m, 6H), 6.93 (d, J = 8.8 Hz, 2H), 4.60 (s, 1H), 3.89 – 3.87 (m, 5H), 3.66 (dt, J = 11.9, 4.3 Hz, 1H), 2.54 (d, J = 14.0 Hz, 1H), 2.46 (t, J = 13.3 Hz, 1H), 2.27 (s, 3H), 2.21 (d, J = 13.9 Hz, 1H), 1.95 (dq, J = 14.9, 7.5 Hz, 1H), 1.61 (dt, J = 13.7, 5.8 Hz, 2H), 1.36 – 1.35 (m, 1H), 1.28 (s, 12H), 0.81 (t, J = 7.4 Hz, 3H).

^{13}C NMR (101 MHz, $CDCl_3$) δ 201.9, 163.1, 142.9, 142.3, 130.6, 128.8, 128.7, 128.5, 128.0, 127.9, 126.5, 113.7, 82.7, 77.6, 75.2, 61.3, 61.2, 55.4, 41.7, 32.5, 24.9, 24.7, 7.9.

HRMS (ESI) Calcd for $C_{36}H_{47}BNO_5^+$ $[M + H]^+$: 584.3542, Found: 584.3555.

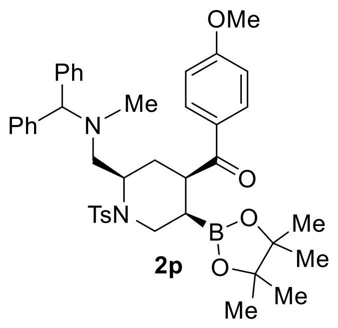

2p

((2*R*,4*R*,5*R*)-2-((Benzhydryl(methyl)amino)methyl)-5-(4,4,5,5-tetramethyl-1,3,2-dioxaborolan-2-yl)-1-tosylpiperidin-4-yl)(4-methoxyphenyl) methanone (2p) was prepared according to the General Procedure D (20 mol% of Cu(TFA)$_2$·H$_2$O and 24 mol% of (*S*,*S*$_p$)-**L9** were used) as a white solid (chromatography eluent: *n*-hexane/EtOAc = 5:1) in 88% yield (124.6 mg, 95% ee, >20:1 dr).

$[\alpha]_D^{25}$: +8.7 (c = 3.8, CHCl$_3$). HPLC analysis of the product: Daicel CHIRALPAK® AD3 column; 10% *i*-PrOH in *n*-hexane; 1.0 mL/min; retention times: 19.9 min (major), 10.4 min (minor).

^{1}H NMR (400 MHz, CDCl$_3$) δ 7.75 (dd, *J* = 17.8, 8.5 Hz, 4H), 7.24 – 7.10 (m, 8H), 6.95 (dd, *J* = 6.5, 2.8 Hz, 2H), 6.90 – 6.86 (m, 2H), 6.83 (d, *J* = 8.8 Hz, 2H), 4.35 (s, 1H), 4.26 (q, *J* = 6.7, 5.8 Hz, 1H), 3.90 (dd, *J* = 14.7, 5.0 Hz, 1H), 3.81 (s, 3H), 3.56 (dt, *J* = 7.4, 2.7 Hz, 1H), 3.47 – 3.32 (m, 1H), 2.43 – 2.40 (s, 4H), 2.17 – 2.08 (m, 2H), 1.82 (s, 3H), 1.36 (dt, *J* = 12.3, 5.1 Hz, 1H), 1.20 (s, 6H), 1.11 (s, 6H)

^{13}C NMR (101 MHz, CDCl$_3$) δ 202.9, 163.2, 142.6, 140.4, 139.9, 138.8, 130.7, 129.5, 128.8, 128.6, 128.4, 127.8, 127.7, 127.1, 126.6, 126.5, 113.6, 83.1, 71.5, 55.3, 54.3, 49.9, 39.9, 39.73, 37.71, 28.8, 24.8, 24.5, 21.5.

HRMS (ESI) Calcd for $C_{41}H_{50}BN_2O_6S^+$ [M + H]$^+$: 709.3477, Found: 709.3483.

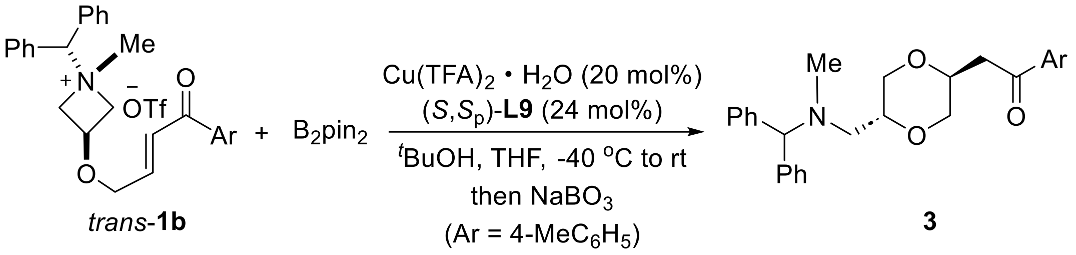

2-((2*S*,5*R*)-5-((Benzhydryl(methyl)amino)methyl)-1,4-dioxan-2-yl)-1-(*p*-tolyl)ethan-1-one (3). In a glove box, a mixture of $Cu(TFA)_2{\cdot}H_2O$ (11.6 mg, 0.04 mmol, 20 mol%) and the chiral ligand (*S*,*S*$_p$)-**L9** (23.8 mg, 0.048 mmol, 24 mol%) in dry THF (0.2 M, 1.0 mL) was stirred at room temperature for 0.5 h. Then, azetidinium **1b** (137.7 mg, 0.24 mmol, 1.2 equiv), and ^{t}BuOH (38.3 uL, 0.4 mmol, 2.0 equiv) were added sequentially. The mixture was cooled to -40 °C and stirred for 10 min before a solution of B_2pin_2 (50.7 mg, 0.2 mmol, 1.0 equiv) in dry THF (1.0 mL) was added dropwise. The mixture was stirred at -40 °C for 0.5 h before it was warmed to room temperature and stirred for 5 h. Then, a solution of $NaBO_3{\cdot}H_2O$ (99.8 mg, 1.0 mmol, 5.0 equiv) in H_2O (1 mL) was added in one portion. The mixture was stirred vigorously in open air at room temperature for 3 h before it was quenched with a saturated aqueous NH_4Cl solution (5 mL) and extracted with EtOAc (3 x 10 mL). The combined organic layers were dried over anhydrous Na_2SO_4, filtered, and concentrated *in vacuo*. The residue was then purified by column chromatography (chromatography eluent: *n*-hexane/EtOAc = 10:1) on silica gel to give the desired product **3** (53.2 mg, 62% yield, 86% ee, 7:1 dr).

$[\alpha]_D^{25}$: -1.2 (*c* = 0.8, $CHCl_3$). HPLC analysis of the product: Daicel CHIRALPAK® IB N-3 column; 10% *i*-PrOH in *n*-hexane; 1.0 mL/min; retention times: 10.1 min (major), 14.1 min (minor).

^{1}H NMR (400 MHz, $CDCl_3$) δ 7.85 (d, *J* = 8.2 Hz, 2H), 7.36 (t, *J* = 6.6 Hz, 4H), 7.28 – 7.24 (m, 6H), 7.17 (t, *J* = 7.0 Hz, 2H), 4.36 (s, 1H), 4.11 (dtd, *J* = 12.6, 6.4, 2.6 Hz, 1H), 3.93 (td, *J* = 11.4, 2.4 Hz, 2H), 3.74 (dtd, *J* = 9.0, 6.3, 2.4 Hz, 1H), 3.38 (dt, *J* = 25.3, 11.2 Hz, 2H), 3.19 (dd, *J* = 16.3, 6.7 Hz, 1H), 2.81 (dd, *J* = 16.3, 5.8 Hz, 1H),

2.49 – 2.32 (m, 5H), 2.22 (s, 3H).

^{13}C NMR (101 MHz, $CDCl_3$) δ 196.7, 144.2, 142.6, 142.5, 134.4, 129.3, 128.4, 128.4, 128.3, 128.1, 128.0, 127.0, 126.9, 76.0, 73.5, 71.6, 71.0, 70.6, 56.3, 41.6, 40.5, 21.6.

HRMS (ESI) Calcd for $C_{28}H_{32}NO_3^+$ $[M + H]^+$: 430.2377, Found: 430.2387.

IV. Intermolecular Asymmetric Opening of Azetidiniums

Table S2. Optimization of Reaction Conditions[a]

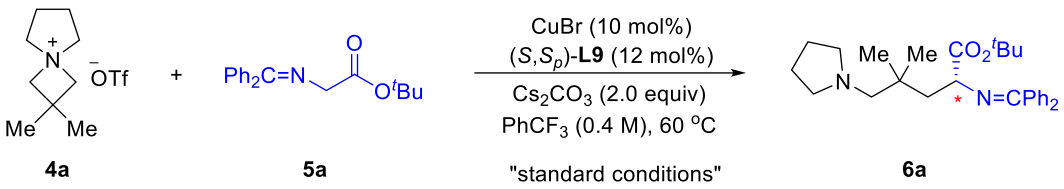

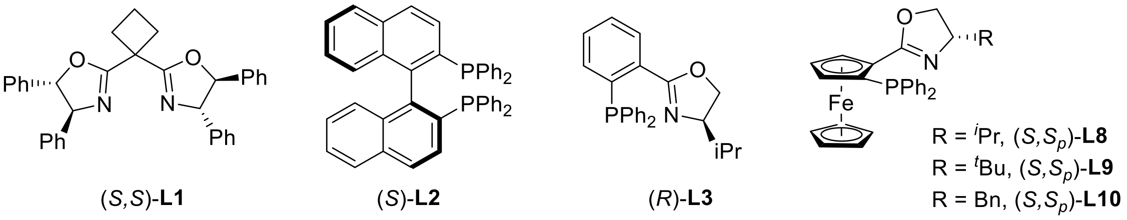

entry	deviation from the "standard conditions"	yield (%)[b]	ee (%)[c]
1	none	82	96
2	no CuBr, no (S,S_p)-**L5**	20	-
3	(S,S_p)-**L1** instead of (S,S_p)-**L9**	27	-
4	(S,S_p)-**L2** instead of (S,S_p)-**L9**	32	12
5	(S,S_p)-**L3** instead of (S,S_p)-**L9**	35	8
6	(S,S_p)-**L8** instead of (S,S_p)-**L9**	50	72
7	(S,S_p)-**L10** instead of (S,S_p)-**L9**	68	80
8	$Cu(CH_3CN)_4BF_4$ instead of CuBr	85	94
9	CuCl instead of CuBr	66	92
10	NaO^{t}Bu instead of Cs_2CO_3	79	8
11	Na_2CO_3 instead of Cs_2CO_3	<5	-
12	Et_3N instead of Cs_2CO_3	<5	-
13	THF instead of $PhCF_3$	25	62
14	toluene instead of $PhCF_3$	15	32
15	CH_3CN instead of $PhCF_3$	10	94
16	80 °C instead of 60 °C	89	86
17	25 °C instead of 60 °C	<5	-
18	c = 0.1 M instead of 0.4 M	75	95

[a]Reaction conditions: **4a** (0.1 mmol), **5a** (0.15 mmol), Cu salt (10 mol%), ligand (12 mol%), base (2.0 equiv), solvent (0.25 mL), 60 °C, 72 h. [b]Determined by ^{1}H NMR spectroscopy of the crude product with 1,2-dibromoethane as an internal standard. [c]Determined by chiral HPLC.

General Procedure E.

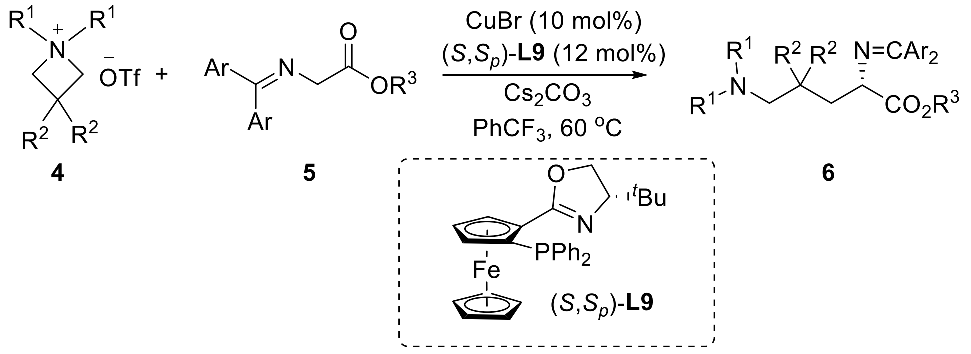

In a glove box, CuBr (4.3 mg, 0.03 mmol, 10 mol%) and the chiral ligand (*S*,*S*$_p$)-**L9** (17.8 mg, 0.036 mmol, 12 mol%) were dissolved in dry $PhCF_3$ (0.4 M, 0.75 mL). The mixture was stirred at room temperature for 0.5 h before the keto imine ester **5** (0.45 mmol, 1.5 equiv), Cs_2CO_3 (0.6 mmol, 2.0 equiv), and the azetidinium **4** (0.3 mmol, 1.0 equiv) were added sequentially. The reaction mixture was stirred at 60 °C for a specified period of time (72 – 168 h) before it was concentrated *in vacuo*. The residue was then purified by column chromatography on silica gel[4] to give the desired product **6**.

General Procedure F.

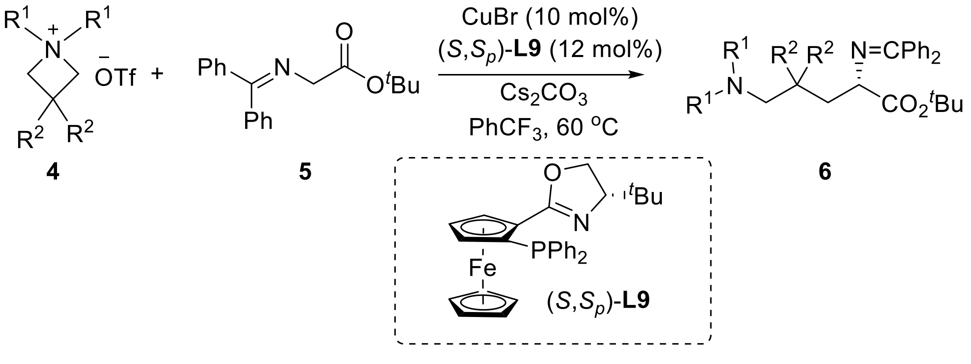

In a glove box, CuBr (4.3 mg, 0.03 mmol, 10 mol%) and the chiral ligand (*S*,*S*$_p$)-**L9** (17.8 mg, 0.036 mmol, 12 mol%) were dissolved in dry $PhCF_3$ (0.4 M, 0.75 mL). The mixture was stirred at room temperature for 0.5 h before the keto imine ester **5a** (0.225 mmol), Cs_2CO_3 (0.3 mmol), and azetidinium **4** (0.15 mmol) were added sequentially. The reaction mixture was stirred at 60 °C for 72 h. Then another batch of the keto imine ester **5a** (0.225 mmol), Cs_2CO_3 (0.3 mmol),

and azetidinium **4** (0.15 mmol) were added. The reaction mixture was stirred at 60 °C for another 72 h before it was concentrated *in vacuo*. The residue was then purified by column chromatography on silica gel[4] to give the desired product **6**.

General Procedure G.

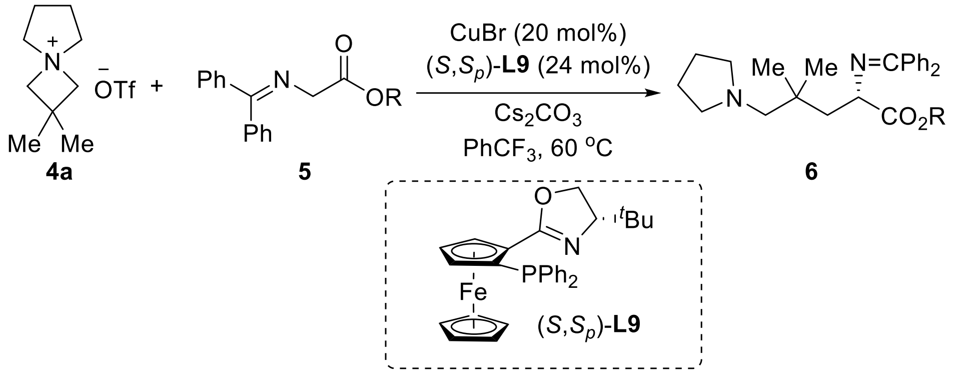

In a glove box, CuBr (2.9 mg, 0.02 mmol, 20 mol%) and chiral ligand (*S,S*$_p$)-**L9** (11.9 mg, 0.024 mmol, 24 mol%) were dissolved in dry $PhCF_3$ (0.2 M, 0.5 mL). The mixture was stirred at room temperature for 0.5 h before the keto imine ester **5** (0.15 mmol, 1.5 equiv), Cs_2CO_3 (0.2 mmol, 2.0 equiv), and azetidinium **4a** (0.1 mmol, 1.0 equiv) were added sequentially. The reaction mixture was stirred at 60 °C for 15 – 24 h before it was concentrated *in vacuo*. The residue was then purified by column chromatography on silica gel[4] to give the desired product **6**.

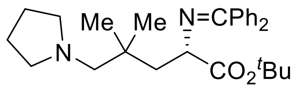

6a

***tert*-Butyl (*S*)-2-((diphenylmethylene)amino)-4,4-dimethyl-5-(pyrrolidin-1-yl)pentanoate (6a)** was prepared according to the General Procedure E for 72 h as a light yellow oil (chromatography eluent: *n*-hexane/EtOAc = 2:1) in 77% yield (100.3 mg, 96% ee).

$[\alpha]_D^{25}$: -10.6 (*c* = 1.0, $CHCl_3$). HPLC analysis of the product: Daicel

CHIRALPAK® AY3 column; 10% *i*-PrOH in *n*-hexane; 1.0 mL/min; retention times: 4.3 min (major), 3.6 min (minor).

^{1}H NMR (400 MHz, $CDCl_3$) δ 7.64 (d, J = 8.3 Hz, 2H), 7.47 – 7.43 (m, 3H), 7.39 – 7.30 (m, 3H), 7.20 (dd, J = 6.4, 2.9 Hz, 2H), 4.07 (dd, J = 6.7, 5.2 Hz, 1H), 2.52 – 2.46 (m, 4H), 2.17 – 2.12 (m, 3H), 1.82 (dd, J = 14.0, 6.7 Hz, 1H), 1.68 – 1.64 (m, 4H), 1.45 (s, 9H), 0.77 (d, J = 5.7 Hz, 6H).

^{13}C NMR (101 MHz, $CDCl_3$) δ 172.5, 168.8, 139.9, 137.0, 130.1, 128.9, 128.6, 128.4, 128.1, 128.0, 80.8, 68.5, 64.3, 57.1, 44.5, 35.5, 28.1, 26.6, 26.1, 24.2.

HRMS (ESI) Calcd for $C_{28}H_{39}N_2O_2^+$ [M + H]$^+$: 435.3006, Found: 435.3009.

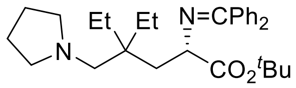

6b

***tert*-Butyl(*S*)-2-((diphenylmethylene)amino)-4-ethyl-4-(pyrrolidin-1-ylmethyl)hexanoate (6b)** was prepared according to the General Procedure F for 144 h as a light yellow oil (chromatography eluent: *n*-hexane/EtOAc = 5:1) in 60% yield (83.2 mg, 86% ee).

$[\alpha]_D^{25}$: -9.9 (c = 1.0, $CHCl_3$). HPLC analysis of the product: Daicel CHIRALPAK® AY3 column; 10% *i*-PrOH in *n*-hexane; 1.0 mL/min; retention times: 4.4 min (major), 3.4 min (minor).

^{1}H NMR (400 MHz, $CDCl_3$) δ 7.66 – 7.64 (m, 2H), 7.44 – 7.42 (m, 3H), 7.39 – 7.29 (m, 3H), 7.19 (dd, J = 6.6, 3.0 Hz, 2H), 4.10 (dd, J = 7.1, 4.3 Hz, 1H), 2.45 – 2.40 (m, 4H), 2.24 – 2.09 (m, 2H), 2.06 – 1.97 (m, 2H), 1.62 – 1.59 (m, 4H), 1.43 (s, 9H), 1.27 – 1.00 (m, 4H), 0.76 (td, J = 7.4, 1.4 Hz, 6H).

^{13}C NMR (101 MHz, $CDCl_3$) δ 172.8, 168.8, 139.9, 137.1, 130.1, 129.0, 128.6, 128.3, 128.04, 127.95, 80.7, 63.6, 61.4, 56.8, 40.4, 38.7, 28.1, 27.2, 24.1, 7.83, 7.80.

HRMS (ESI) Calcd for $C_{30}H_{43}N_2O_2^+$ [M + H]$^+$: 463.3319, Found: 463.3344.

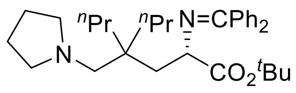

6c

***tert*-Butyl (*S*)-2-((diphenylmethylene)amino)-4-propyl-4-(pyrrolidin-1-ylmethyl)heptanoate (6c)** was prepared according to the General Procedure F for 144 h as a light yellow oil (chromatography eluent: *n*-hexane/EtOAc = 5:1) in 73% yield (107.3 mg, 92% ee).

$[\alpha]_D^{25}$: -8.3 (c = 1.0, $CHCl_3$). HPLC analysis of the product: Daicel CHIRALPAK® AY3 column; 10% *i*-PrOH in *n*-hexane; 1.0 mL/min; retention times: 4.0 min (major), 3.3 min (minor).

^{1}H NMR (400 MHz, $CDCl_3$) δ 7.66 – 7.64 (m, 2H), 7.45 – 7.42 (m, 3H), 7.39 – 7.29 (m, 3H), 7.21 – 7.19 (m, 2H), 4.10 (dd, J = 7.1, 4.5 Hz, 1H), 2.46 – 2.41 (m, 4H), 2.26 – 2.13 (m, 2H), 2.07 – 1.93 (m, 2H), 1.63 – 1.59 (m, 4H), 1.43 (s, 9H), 1.21 – 1.24 (m, 2H), 1.21-1.05 (m, 5H), 0.97 – 0.90 (m, 1H), 0.74 (dt, J = 19.1, 6.8 Hz, 6H).

^{13}C NMR (101 MHz, $CDCl_3$) δ 172.8, 168.8, 139.8, 137.0, 130.1, 128.9, 128.6, 128.3, 128.04, 128.01, 80.7, 63.7, 62.5, 56.9, 40.5, 39.7, 39.0, 38.2, 28.1, 24.2, 16.34, 16.30, 15.0.

HRMS (ESI) Calcd for $C_{32}H_{47}N_2O_2^+$ $[M + H]^+$: 491.3632, Found: 491.3646.

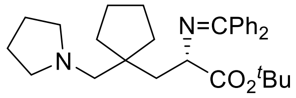

6d

***tert*-Butyl (*S*)-2-((diphenylmethylene)amino)-3-(1-(pyrrolidin-1-ylmethyl) cyclopentyl)propanoate (6d)** was prepared according to the General Procedure E for 144 h as a light yellow oil (chromatography eluent: *n*-hexane/EtOAc = 5:1) in 63% yield (86.9 mg, 86% ee).

$[\alpha]_D^{25}$: -9.0 (c = 1.0, $CHCl_3$). HPLC analysis of the product: Daicel CHIRALPAK® AY3 column; 10% *i*-PrOH in *n*-hexane; 1.0 mL/min; retention times: 4.6 min (major), 3.6 min (minor).

^{1}H NMR (400 MHz, $CDCl_3$) δ 7.64 (d, J = 7.1 Hz, 2H), 7.44 – 7.42 (m, 3H), 7.39 – 7.30 (m, 3H), 7.21 (dd, J = 6.4, 2.7 Hz, 2H), 4.16 (dd, J = 7.6, 4.1 Hz, 1H), 2.33 – 1.98 (m, 8H), 1.59 – 1.44 (m, 19H), 1.36 – 1.20 (m, 2H).

^{13}C NMR (101 MHz, $CDCl_3$) δ 172.7, 168.8, 140.0, 137.0, 130.1, 129.0, 128.6, 128.3, 128.03, 127.96, 80.7, 64.6, 63.9, 56.2, 46.7, 41.4, 37.7, 36.5, 28.1, 24.5, 23.94, 23.87.

HRMS (ESI) Calcd for $C_{30}H_{41}N_2O_2^+$ [M + H]$^+$: 461.3163, Found: 461.3165.

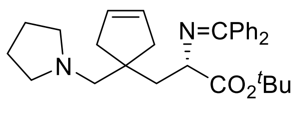

6e

***tert*-Butyl (*S*)-2-((diphenylmethylene)amino)-3-(1-(pyrrolidin-1-ylmethyl)cyclopent-3-en-1-yl)propanoate (6e)** was prepared according to the General Procedure E for 113 h as a light yellow oil (chromatography eluent: *n*-hexane/EtOAc = 5:1) in 66% yield (90.7mg, 94% ee).

$[\alpha]_D^{25}$: -5.6 (c = 1.0, $CHCl_3$). HPLC analysis of the product: Daicel CHIRALPAK® AY3 column; 10% *i*-PrOH in *n*-hexane; 1.0 mL/min; retention times: 5.5 min (major), 3.7 min (minor).

^{1}H NMR (400 MHz, $CDCl_3$) δ 7.65 – 7.63 (m, 2H), 7.43 – 7.43 (m, 3H), 7.39 – 7.29 (m, 3H), 7.19 (dd, J = 6.6, 3.0 Hz, 2H), 5.47 (q, J = 5.9 Hz, 2H), 4.11 (dd, J = 8.0, 4.0 Hz, 1H), 2.41 – 1.97 (m, 12H), 1.66 – 1.63 (s, 4H), 1.44 (s, 9H).

^{13}C NMR (101 MHz, $CDCl_3$) δ 172.4, 168.9, 139.8, 136.8, 130.1, 129.4, 129.2, 128.9, 128.6, 128.3, 128.1, 128.0, 80.7, 65.0, 64.4, 56.2, 45.8, 43.9, 42.9, 42.7, 28.1, 23.9.

HRMS (ESI) Calcd for $C_{30}H_{39}N_2O_2^+$ [M + H]$^+$: 459.3006, Found: 459.3013.

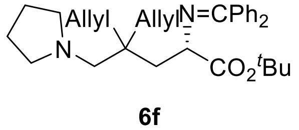

6f

***tert*-Butyl (*S*)-4-allyl-2-((diphenylmethylene)amino)-4-(pyrrolidin-1-ylmethyl)hept-6-enoate (6f)** was prepared according to the General Procedure

E for 144 h as a light yellow oil (chromatography eluent: *n*-hexane/EtOAc = 5:1) in 65% yield (94.8 mg, 92% ee).

$[\alpha]_D^{25}$: -11.6 (*c* = 1.0, $CHCl_3$). HPLC analysis of the product: Daicel CHIRALPAK® AY3 column; 10% *i*-PrOH in *n*-hexane; 1.0 mL/min; retention times: 5.3 min (major), 4.4 min (minor).

^{1}H NMR (400 MHz, $CDCl_3$) δ 7.65 – 7.63 (m, 2H), 7.46 – 7.42 (m, 3H), 7.39 – 7.29 (m, 3H), 7.23 – 7.20 (m, 2H), 5.92 – 5.81 (m, 2H), 5.00 – 4.85 (m, 4H), 4.20 (dd, *J* = 7.2, 4.4 Hz, 1H), 2.47 (s, 4H), 2.50 – 2.43 (m, 2H), 2.32 – 2.27 (m, 4H), 2.18 – 2.00 (m, 2H), 1.64 – 1.60 (m, 4H), 1.44 (s, 9H).

^{13}C NMR (101 MHz, $CDCl_3$) δ 171.7, 168.3, 139.1, 136.3, 134.7, 129.4, 128.3, 128.0, 127.7, 127.3, 127.2, 116.6, 116.4, 80.2, 62.8, 62.0, 56.1, 40.3, 40.2, 39.7, 39.1, 27.4, 23.5.

HRMS (ESI) Calcd for $C_{32}H_{43}N_2O_2^+$ $[M + H]^+$: 487.3319, Found: 487.3322.

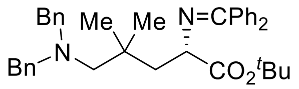

6g

***tert*-Butyl (*S*)-5-(dibenzylamino)-2-((diphenylmethylene)amino)-4,4-dimethylpentanoate (6g)** was prepared according to the General Procedure E for 144 h as a light yellow oil (chromatography eluent: *n*-hexane/EtOAc = 20:1) in 68% yield (114.2 mg, 94% ee).

$[\alpha]_D^{25}$: -6.3 (*c* = 1.0, $CHCl_3$). HPLC analysis of the product: Daicel CHIRALPAK® AY3 column; 10% *i*-PrOH in *n*-hexane; 1.0 mL/min; retention times: 4.5 min (major), 3.9 min (minor).

^{1}H NMR (400 MHz, $CDCl_3$) δ 7.63 – 7.61 (m, 2H), 7.42 – 7.41 (m, 3H), 7.37 – 7.19 (m, 13H), 7.12 (dd, *J* = 6.5, 2.9 Hz, 2H), 3.95 (dd, *J* = 7.1, 4.8 Hz, 1H), 3.59 – 3.50 (m, 4H), 2.35 – 2.27 (m, 2H), 2.08 (dd, *J* = 14.0, 4.8 Hz, 1H), 1.75 (dd, *J* = 14.0, 7.2 Hz, 1H), 1.41 (s, 9H), 0.67 (d, *J* = 2.6 Hz, 6H).

^{13}C NMR (101 MHz, $CDCl_3$) δ 172.4, 168.9, 140.0, 139.8, 137.0, 130.2, 129.2, 128.9,

128.7, 128.5, 128.2, 128.1, 127.9, 126.9, 80.8, 65.5, 64.1, 60.6, 44.6, 36.2, 28.1, 26.0.

HRMS (ESI) Calcd for $C_{38}H_{45}N_2O_2^+$ [M + H]$^+$: 561.3476, Found: 561.3480.

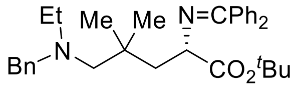

6h

***tert*-Butyl (*S*)-5-(benzyl(ethyl)amino)-2-((diphenylmethylene)amino)-4,4-dimethylpentanoate (6h)** was prepared according to the General Procedure E for 120 h as a light yellow oil (chromatography eluent: *n*-hexane/EtOAc = 20:1) in 60% yield (89.6 mg, 93% ee).

$[\alpha]_D^{25}$: -14.8 (c = 1.0, $CHCl_3$). HPLC analysis of the product: Daicel CHIRALPAK® AY3 column; 10% *i*-PrOH in *n*-hexane; 1.0 mL/min; retention times: 3.5 min (major), 4.0 min (minor).

^{1}H NMR (400 MHz, $CDCl_3$) δ 7.69 – 7.67 (m, 2H), 7.48 – 7.46 (m, 3H), 7.41 – 7.28 (m, 7H), 7.23 (dt, J = 5.5, 2.5 Hz, 3H), 4.06 (dd, J = 6.8, 5.0 Hz, 1H), 3.63 (s, 2H), 2.44 (q, J = 7.1 Hz, 2H), 2.25 – 2.19 (m, 3H), 1.86 (dd, J = 14.0, 7.0 Hz, 1H), 1.47 (s, 9H), 0.92 (t, J = 7.1 Hz, 3H), 0.79 (d, J = 5.5 Hz, 6H).

^{13}C NMR (101 MHz, $CDCl_3$) δ 172.5, 169.0, 140.9, 139.9, 137.0, 130.2, 128.9, 128.7, 128.5, 128.5, 128.15, 128.09, 127.9, 126.6, 80.8, 65.9, 64.2, 60.7, 49.2, 44.5, 36.2, 28.1, 26.0, 25.6, 11.7.

HRMS (ESI) Calcd for $C_{33}H_{43}N_2O_2^+$ [M + H]$^+$: 499.3319, Found: 499.3323.

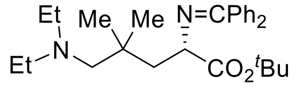

6i

***tert*-Butyl (*S*)-5-(diethylamino)-2-((diphenylmethylene)amino)-4,4-dimethylpentanoate (6i)** was prepared according to the General Procedure E for 120 h as a light yellow oil (chromatography eluent: *n*-hexane/EtOAc = 5:1) in 74% yield (96.8 mg, 93% ee).

$[\alpha]_D^{25}$: -4.6 (c = 1.0, $CHCl_3$). HPLC analysis of the product: Daicel CHIRALPAK®

AY3 column; 10% *i*-PrOH in *n*-hexane; 1.0 mL/min; retention times: 4.0 min (major), 3.4 min (minor).

^{1}H NMR (400 MHz, $CDCl_3$) δ 7.64 – 7.62 (m, 2H), 7.45 – 7.43 (m, 3H), 7.37 – 7.30 (m, 3H), 7.20 (dd, *J* = 6.5, 3.0 Hz, 2H), 4.03 (dd, *J* = 6.7, 5.2 Hz, 1H), 2.47 – 2.44 (m, 4H), 2.12 – 2.06 (m, 3H), 1.76 (dd, *J* = 14.0, 6.8 Hz, 1H), 1.44 (s, 9H), 0.91 (t, *J* = 6.9 Hz, 6H), 0.72 (d, *J* = 8.6 Hz, 6H).

^{13}C NMR (101 MHz, $CDCl_3$) δ 172.5, 168.9, 139.9, 137.0, 130.2, 128.9, 128.7, 128.5, 128.1, 127.9, 80.8, 66.1, 64.3, 49.2, 44.4, 35.9, 28.1, 25.9, 25.5, 12.1.

HRMS (ESI) Calcd for $C_{28}H_{41}N_2O_2^+$ [M + H]$^+$: 437.3163, Found: 437.3169.

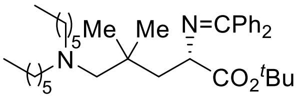

6j

***tert*-Butyl (*S*)-5-(dihexylamino)-2-((diphenylmethylene)amino)-4,4-dimethylpentanoate (6j)** was prepared according to the General Procedure E for 168 h as a light yellow oil (chromatography eluent: *n*-hexane/EtOAc = 5:1) in 67% yield (110.1 mg, 91% ee).

$[\alpha]_D^{25}$: -5.1 (*c* = 1.0, $CHCl_3$). HPLC analysis of the product: Daicel CHIRALPAK® AY3 column; 10% *i*-PrOH in *n*-hexane; 1.0 mL/min; retention times: 4.0 min (major), 3.7 min (minor).

^{1}H NMR (400 MHz, $CDCl_3$) δ 7.66 – 7.63 (m, 2H), 7.47 – 7.43 (m, 3H), 7.40 – 7.30 (m, 3H), 7.22 – 7.20 (m, 2H), 4.02 (dd, *J* = 6.8, 5.0 Hz, 1H), 2.37 – 2.33 (m, 4H), 2.12 – 2.03 (m, 3H), 1.77 (dd, *J* = 14.0, 6.9 Hz, 1H), 1.46 (s, 9H), 1.35 – 1.21 (m, 16H), 0.88 (t, *J* = 7.0 Hz, 6H), 0.72 (d, *J* = 7.4 Hz, 6H).

^{13}C NMR (101 MHz, $CDCl_3$) δ 172.6, 168.8, 139.9, 137.1, 130.1, 128.9, 128.7, 128.5, 128.1, 128.0, 80.8, 67.5, 64.3, 56.6, 44.5, 36.1, 32.1, 28.1, 27.3, 26.0, 25.6, 22.9, 14.2.

HRMS (ESI) Calcd for $C_{36}H_{57}N_2O_2^+$ [M + H]$^+$: 549.4415, Found: 549.4419.

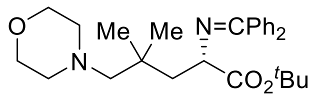

6k

***tert*-Butyl (*S*)-2-((diphenylmethylene)amino)-4,4-dimethyl-5-morpholinopentanoate (6k)** was prepared according to the General Procedure E for 144 h as a light yellow oil (chromatography eluent: *n*-hexane/EtOAc = 5:1) in 56% yield (75.6 mg, 94% ee).

$[\alpha]_D^{25}$: -11.7 (*c* = 1.0, $CHCl_3$). HPLC analysis of the product: Daicel CHIRALPAK® AY3 column; 10% *i*-PrOH in *n*-hexane; 1.0 mL/min; retention times: 6.0 min (major), 4.6 min (minor).

^{1}H NMR (400 MHz, $CDCl_3$) δ 7.64 – 7.62 (m, 2H), 7.46 – 7.44 (m, 3H), 7.40 – 7.30 (m, 3H), 7.20 (dd, *J* = 6.5, 3.0 Hz, 2H), 4.06 (dd, *J* = 6.9, 4.9 Hz, 1H), 3.60 (d, *J* = 9.1 Hz, 4H), 2.47 – 2.37 (m, 4H), 2.12 (dd, *J* = 14.0, 4.9 Hz, 1H), 1.98 (s, 2H), 1.82 (dd, *J* = 14.0, 7.0 Hz, 1H), 1.45 (s, 9H), 0.74 (d, *J* = 8.0 Hz, 6H).

^{13}C NMR (101 MHz, $CDCl_3$) δ 172.4, 169.0, 139.8, 136.9, 130.2, 128.9, 128.7, 128.5, 128.0, 127.9, 80.9, 70.1, 67.5, 64.1, 56.2, 43.9, 36.0, 28.1, 26.1, 25.4.

HRMS (ESI) Calcd for $C_{28}H_{39}N_2O_3^+$ $[M + H]^+$: 451.2955, Found: 451.2959.

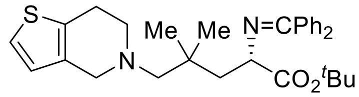

6l

***tert*-Butyl (*S*)-5-(6,7-dihydrothieno[3,2-c]pyridin-5(4H)-yl)-2-((diphenyl methylene)amino)-4,4-dimethylpentanoate (6l)** was prepared according to the General Procedure E for 168 h as a light yellow oil (chromatography eluent: *n*-hexane/EtOAc = 10:1) in 73% yield (109.9 mg, 95% ee).

$[\alpha]_D^{25}$: -50.7 (*c* = 2.0, $CHCl_3$). HPLC analysis of the product: Daicel CHIRALPAK® AY3 column; 10% *i*-PrOH in *n*-hexane; 1.0 mL/min; retention times: 5.4 min (major), 4.2 min (minor).

^{1}H NMR (400 MHz, $CDCl_3$) δ 7.64 – 7.62 (m, 2H), 7.42 – 7.40 (m, 3H), 7.38 – 7.29

(m, 3H), 7.19 (dd, J = 6.4, 2.9 Hz, 2H), 7.03 (d, J = 5.1 Hz, 1H), 6.66 (d, J = 5.1 Hz, 1H), 4.08 (dd, J = 6.7, 5.1 Hz, 1H), 3.56 (s, 2H), 2.80 – 2.76 (m, 4H), 2.19 (s, 2H), 2.13 (dd, J = 14.0, 5.1 Hz, 1H), 1.85 (dd, J = 14.0, 6.9 Hz, 1H), 1.43 (s, 9H), 0.78 (d, J = 3.0 Hz, 6H).

^{13}C NMR (101 MHz, $CDCl_3$) δ 172.4, 169.0, 139.8, 137.0, 135.0, 133.8, 130.2, 128.9, 128.7, 128.5, 128.1, 127.9, 125.4, 122.2, 80.9, 68.7, 64.2, 55.7, 53.3, 44.0, 36.1, 28.1, 25.9, 25.5, 25.4.

HRMS (ESI) Calcd for $C_{31}H_{39}N_2O_2S^+$ [M + H]$^+$: 503.2727, Found: 503.2736.

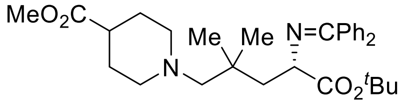

6m

Methyl (*S*)-1-(5-(*tert*-butoxy)-4-((diphenylmethylene)amino)-2,2-dimethyl-5-oxopentyl)piperidine-4-carboxylate (6m) was prepared according to the General Procedure E for 168 h as a light yellow oil (chromatography eluent: *n*-hexane/EtOAc = 5:1) in 57% yield (86.5 mg, 91% ee).

$[\alpha]_D^{25}$: -7.9 (c = 2.0, $CHCl_3$). HPLC analysis of the product: Daicel CHIRALPAK® IC3 column; 10% *i*-PrOH in *n*-hexane; 1.0 mL/min; retention times: 6.9 min (major), 6.1 min (minor).

^{1}H NMR (400 MHz, $CDCl_3$) δ 7.64 – 7.62 (m, 2H), 7.47 – 7.41 (m, 3H), 7.37 – 7.29 (m, 3H), 7.20 (dd, J = 7.2, 2.3 Hz, 2H), 4.05 (dd, J = 6.8, 5.0 Hz, 1H), 3.66 (s, 3H), 2.70 (dd, J = 31.9, 11.3 Hz, 2H), 2.21 – 2.07 (m, 4H), 1.96 (s, 2H), 1.82 – 1.60 (m, 5H), 1.45 (s, 9H), 0.72 (d, J = 6.6 Hz, 6H).

^{13}C NMR (101 MHz, $CDCl_3$) δ 176.0, 172.5, 168.9, 139.8, 136.9, 130.1, 128.9, 128.7, 128.43, 128.41, 128.0, 127.9, 80.8, 69.4, 64.2, 55.7, 51.6, 43.8, 40.9, 36.0, 28.8, 28.1, 25.9, 25.1.

HRMS (ESI) Calcd for $C_{31}H_{43}N_2O_4^+$ [M + H]$^+$: 507.3217, Found: 507.3221.

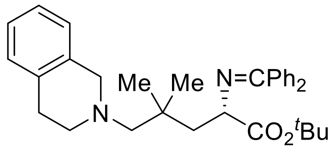

6n

***tert*-Butyl (*S*)-5-(3,4-dihydroisoquinolin-2(1H)-yl)-2-((diphenyl methylene) amino)-4,4-dimethylpentanoate (6n)** was prepared according to the General Procedure E for 168 h as a light yellow oil (chromatography eluent: *n*-hexane/EtOAc = 25:1) in 70% yield (104.2 mg, 94% ee).

$[\alpha]_D^{25}$: -5.0 (*c* = 4.8, $CHCl_3$). HPLC analysis of the product: Daicel CHIRALPAK® AY3 column; 10% *i*-PrOH in *n*-hexane; 1.0 mL/min; retention times: 4.8 min (major), 3.9 min (minor).

^{1}H NMR (400 MHz, $CDCl_3$) δ 7.64 – 7.62 (m, 2H), 7.40 – 7.38 (m, 3H), 7.36 – 7.28 (m, 3H), 7.21 – 7.17 (m, 2H), 7.10 – 7.04 (m, 3H), 6.94 – 6.91 (m, 1H), 4.10 (dd, *J* = 6.8, 5.1 Hz, 1H), 3.63 (s, 2H), 2.78 – 2.67 (m, 4H), 2.18 – 2.13 (m, 3H), 1.87 (dd, *J* = 14.0, 6.9 Hz, 1H), 1.43 (s, 9H), 0.79 (d, *J* = 3.7 Hz, 6H).

^{13}C NMR (101 MHz, $CDCl_3$) δ 172.4, 169.0, 139.8, 136.9, 136.0, 134.8, 130.1, 128.9, 128.7, 128.7, 128.4, 128.0, 127.9, 126.5, 125.9, 125.4, 80.8, 69.3, 64.2, 58.5, 53.6, 44.0, 36.1, 29.5, 28.1, 26.0, 25.4.

HRMS (ESI) Calcd for $C_{33}H_{41}N_2O_2^+$ $[M + H]^+$: 497.3163, Found: 497.3171.

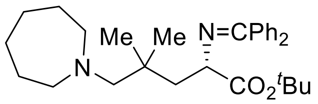

6o

***tert*-Butyl (*S*)-5-(azepan-1-yl)-2-((diphenylmethylene)amino)-4,4-dimethyl pentanoate (6o)** was prepared according to the General Procedure E for 168 h as a light yellow oil (chromatography eluent: dichloromethane/EtOAc = 5:1) in 85% yield (117.8 mg, 90% ee).

$[\alpha]_D^{25}$: -8.4 (*c* = 2.5, $CHCl_3$). HPLC analysis of the product: Daicel CHIRALPAK® AY3 column; 10% *i*-PrOH in *n*-hexane; 1.0 mL/min; retention times: 4.1 min

(major), 3.5 min (minor).

^{1}H NMR (400 MHz, $CDCl_3$) δ 7.65 – 7.63 (m, 2H), 7.44 – 7.43 (m, 3H), 7.38 – 7.29 (m, 3H), 7.20 (dd, J = 6.4, 3.1 Hz, 2H), 4.02 (dd, J = 7.0, 5.0 Hz, 1H), 2.66 – 2.59 (m, 4H), 2.17 – 2.10 (m, 3H), 1.78 (dd, J = 14.0, 7.1 Hz, 1H), 1.54 – 1.51 (s, 8H), 1.45 (s, 9H), 0.71 (d, J = 8.3 Hz, 6H).

^{13}C NMR (101 MHz, $CDCl_3$) δ 172.6, 168.8, 139.9, 137.0, 130.1, 128.9, 128.7, 128.4, 128.1, 128.0, 80.7, 70.4, 64.3, 58.9, 43.8, 36.6, 29.8, 27.2, 25.9, 25.2.

HRMS (ESI) Calcd for $C_{30}H_{43}N_2O_2^+$ [M + H]$^+$: 463.3319, Found: 463.3326.

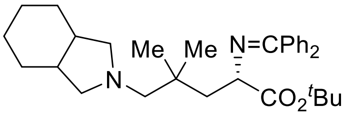

6p

***tert*-Butyl (2*S*)-2-((diphenylmethylene)amino)-4,4-dimethyl-5-(octahydro-2*H*-isoindol-2-yl)pentanoate (6p)** was prepared according to the General Procedure F for 168 h as a light yellow oil (chromatography eluent: *n*-hexane /EtOAc = 10:1) in 75% yield (109.8 mg, 86% ee).

$[\alpha]_D^{25}$: -5.5 (c = 1.0, $CHCl_3$). HPLC analysis of the product: Daicel CHIRALPAK® AY3 column; 10% *i*-PrOH in *n*-hexane; 1.0 mL/min; retention times: 4.0 min (major), 3.5 min (minor).

^{1}H NMR (400 MHz, $CDCl_3$) δ 7.64 (d, J = 7.5 Hz, 2H), 7.45 – 7.44 (m, 3H), 7.39 – 7.29 (m, 3H), 7.21 (d, J = 4.7 Hz, 2H), 4.05 (t, J = 5.8 Hz, 1H), 2.69 – 2.68 (m, 2H), 2.57 – 2.51 (m, 2H), 2.26 (s, 2H), 2.12 (dd, J = 14.0, 4.7 Hz, 1H), 2.04 – 1.98 (m, 2H), 1.80 (dd, J = 14.0, 7.0 Hz, 1H), 1.45 – 1.39 (m, 15H), 1.29 – 1.26 (m, 2H), 0.73 (d, J = 8.9 Hz, 6H).

^{13}C NMR (101 MHz, $CDCl_3$) δ 172.6, 168.8, 140.0, 137.0, 130.1, 129.0, 128.7, 128.4, 128.1, 128.0, 80.7, 70.9, 64.4, 61.9, 61.8, 44.1, 38.02, 37.98, 36.4, 28.1, 26.9, 26.6, 25.9, 23.23, 23.18

HRMS (ESI) Calcd for $C_{32}H_{45}N_2O_2^+$ [M + H]$^+$: 489.3476, Found: 489.3478.

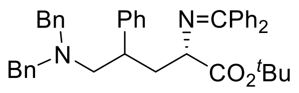

6q

***tert*-Butyl (*S*)-5-(dibenzylamino)-2-((diphenylmethylene)amino)-4-phenyl pentanoate (6q)** was prepared according to the General Procedure F for 168 h as a light yellow oil (chromatography eluent: *n*-hexane/EtOAc = 10:1) in 51% yield (93.0 mg, 94% ee, 3.2:1 dr, a mixture of two diastereomers).

$[\alpha]_D^{25}$: -3.2 (*c* = 1.0, $CHCl_3$). HPLC analysis of the product: Daicel CHIRALPAK® IE3 column; 10% *i*-PrOH in *n*-hexane; 1.0 mL/min; retention times: 5.3 min (major), 4.8 min (minor).

^{1}H NMR (400 MHz, $CDCl_3$) δ 7.64 (d, *J* = 7.1 Hz, 0.51H), 7.53 (d, *J* = 7.1 Hz, 1.77H), 7.42 – 7.09 (m, 20H), 6.93 – 6.87 (m, 3H), 3.82 (dd, *J* = 8.0, 5.5 Hz, 0.77H), 3.67 – 3.42 (m, 4H), 3.12 – 3.03 (m, 10H), 2.65 – 2.51 (m, 3H), 1.82 – 1.77 (m, 1H), 1.44 (s, 6.19H), 1.41 (s, 1.95H).

^{13}C NMR (101 MHz, $CDCl_3$) δ 170.9, 169.0, 142.9, 139.1, 139.0, 138.9, 135.6, 129.4, 128.2, 128.1, 127.8, 127.6, 127.5, 127.4, 127.4, 127.3, 127.0, 126.9, 126.0, 125.4, 80.2, 64.1, 62.6. 60.3, 59.3, 57.9, 57.7, 40.5, 39.9, 37.6, 27.5, 27.4.

HRMS (ESI) Calcd for $C_{42}H_{45}N_2O_2^+$ $[M + H]^+$: 609.3476, Found: 609.3477.

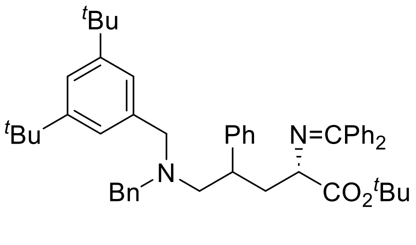

6r

***tert*-Butyl (2*S*)-5-(benzyl(3,5-di-*tert*-butylbenzyl)amino)-2-((diphenyl methylene)amino)-4-phenylpentanoate (6s)** was prepared according to the General Procedure **E** for 168 h as a light yellow oil (chromatography eluent: *n*-hexane/EtOAc = 10:1) in 78% yield (168.6 mg, 96% ee, 2.3:1 dr, a mixture of two diastereomers). To accurately determine the product ee value and dr value, the

product was hydrolyzed with 10% citric acid.

[α]$_D^{25}$: -1.9 (*c* = 3.3, $CHCl_3$). HPLC analysis of the product: Daicel CHIRALPAK® ID3 column; 10% *i*-PrOH in *n*-hexane; 1.0 mL/min; retention times: 7.0 min (major), 5.5 min (minor).

^{1}H NMR (400 MHz, $CDCl_3$) δ 7.27 – 7.04 (m, 13H), 3.63 (d, *J* = 13.4 Hz, 1H), 3.51 (d, *J* = 13.4 Hz, 1H), 3.45 (s, 2H), 3.22 – 3.05 (m, 2H), 2.61 (d, *J* = 7.5 Hz, 2H), 2.18 (ddd, *J* = 19.0, 7.7, 3.6 Hz, 1H), 2.01 (dd, *J* = 14.6, 8.9 Hz, 1H), 1.94 – 1.83 (m, 2H), 1.43 (s, 9H), 1.29 (s, 18H).

^{13}C NMR (101 MHz, $CDCl_3$) δ 175.1, 150.4, 143.8, 139.5, 138.2, 128.9, 128.3, 128.1, 128.0, 126.7, 126.3, 123.2, 120.6, 81.0, 59.9, 58.7, 58.4, 53.5, 40.9, 34.7, 31.5, 28.1.

HRMS (ESI) Calcd for $C_{37}H_{53}N_2O_2^+$ [M + H]$^+$: 557.4102, Found: 557.4111.

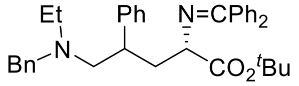

6s

***tert*-Butyl (2*S*)-5-(benzyl(ethyl)amino)-2-((diphenylmethylene)amino)-4-phenylpentanoate (6s)** was prepared according to the General Procedure **E** for 168 h as a light yellow oil (chromatography eluent: *n*-hexane/EtOAc = 5:1) in 72% yield (118.0 mg, 82% ee, 1.7:1 dr, a mixture of two diastereomers). To accurately determine the product ee value and dr value, the product was hydrolyzed with 10% citric acid.

[α]$_D^{25}$: +5.0 (c = 1.6, $CHCl_3$). HPLC analysis of the product: Daicel CHIRALPAK® IE3 column; 10% *i*-PrOH in *n*-hexane; 1.0 mL/min; retention times: 7.6 min (major), 9.5 min (minor).

^{1}H NMR (400 MHz, $CDCl_3$) δ 7.33 – 7.09 (m, 10H), 3.66 (dd, *J* = 31.1, 13.7 Hz, 1H), 3.51 (dd, *J* = 13.6, 2.9 Hz, 1H), 3.38 – 2.86 (m, 2H), 2.76 – 2.42 (m, 4H), 2.42 - 2.28 (m, 2H), 2.09 – 1.73 (m, 2H), 1.43 (d, *J* = 12.4 Hz, 9H), 1.05 – 0.94 (m, 3H).

^{13}C NMR (101 MHz, $CDCl_3$) δ 176.9, 144.0, 129.0, 128.8, 128.43, 128.38, 128.2, 128.0, 127.8, 126.9, 126.7, 126.4, 126.4, 81.1, 60.4, 60.1, 58.5, 58.1, 53.5, 47.6, 41.1,

40.8, 29.3, 28.1, 28.0, 11.4.

HRMS (ESI) Calcd for $C_{24}H_{35}N_2O_2^+$ [M + H]$^+$: 383.2693, Found: 383.2700.

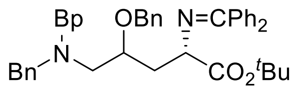

6t

***tert*-Butyl (2*S*)-5-(benzhydryl(benzyl)amino)-4-(benzyloxy)-2-((diphenyl methylene)amino)pentanoate (6t)** was prepared according to the General Procedure E for 168 h as a Light yellow oil (chromatography eluent: *n*-hexane/EtOAc = 10:1) in 54% yield (93.0 mg, 86% ee, 1:1 dr, a mixture of two diastereomers). To accurately determine the product ee value and dr value, the product was hydrolyzed with 10% citric acid.

$[\alpha]_D^{25}$: +3.4 (c = 2.1, $CHCl_3$). HPLC analysis of the product: Daicel CHIRALPAK® IBN3 column; 10% *i*-PrOH in *n*-hexane; 1.0 mL/min; retention times: 5.4 min (major), 7.1 min (minor).

^{1}H NMR (400 MHz, $CDCl_3$) δ 7.40 – 7.20 (m, 20H), 4.99 (d, J = 11.2 Hz, 1H), 4.57 – 4.32 (m, 2H), 3.78 – 3.30 (m, 4H), 2.79 (dd, J = 13.4, 4.3 Hz, 1H), 2.55 (dd, J = 13.3, 5.2 Hz, 1H), 2.27 – 1.94 (m, 4H), 1.43 (s, 9H).

^{13}C NMR (101 MHz, $CDCl_3$) δ 174.0, 140.7, 140.6, 140.5, 139.4, 139.3, 138.33, 138.25, 129.29, 129.26, 129.2, 128.98, 128.96, 128.41, 128.38, 128.32, 128.30, 128.2, 128.14, 128.1, 127.7, 127.6, 127.09, 127.06, 127.0, 81.4, 74.8, 72.0, 71.7, 69.8, 69.5, 56.1, 55.9, 54.0, 53.7, 35.9, 29.3, 28.0.

HRMS (ESI) Calcd for $C_{36}H_{43}N_2O_3^+$ [M + H]$^+$: 551.3268, Found: 551.3275.

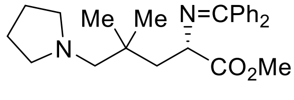

6u

Methyl (*S*)-2-((diphenylmethylene)amino)-4,4-dimethyl-5-(pyrrolidin-1-yl)pentanoate (6u) was prepared according to the General Procedure G for 15

h as a light yellow oil (chromatography eluent: *n*-hexane/EtOAc = 2:1) in 68% yield (26.7 mg, 83% ee).

$[\alpha]_D^{25}$: -8.2 (c = 1.0, $CHCl_3$). HPLC analysis of the product: Daicel CHIRALPAK® AY3 column; 10% *i*-PrOH in *n*-hexane; 1.0 mL/min; retention times: 4.7 min (major), 4.2 min (minor).

^{1}H NMR (400 MHz, $CDCl_3$) δ 7.63 (d, J = 7.1 Hz, 2H), 7.46 – 7.45 (m, 3H), 7.40 – 7.30 (m, 3H), 7.20 (d, J = 5.2 Hz, 2H), 4.26 (t, J = 6.1 Hz, 1H), 3.71 (s, 3H), 2.48 – 2.45 (s, 4H), 2.19 – 2.08 (m, 3H), 1.83 (dd, J = 14.1, 6.8 Hz, 1H), 1.68 – 1.63 (s, 4H), 0.75 (d, J = 12.6 Hz, 6H).

^{13}C NMR (101 MHz, $CDCl_3$) δ 173.9, 169.4, 139.8, 136.7, 130.3, 129.0, 128.9, 128.6, 128.1, 128.0, 68.6, 63.6, 57.0, 52.2, 44.8, 35.5, 26.7, 26.1, 24.2.

HRMS (ESI) Calcd for $C_{25}H_{33}N_2O_2^+$ $[M + H]^+$: 393.2537, Found:393.2542.

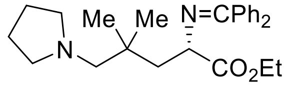

6v

Ethyl (*S*)-2-((diphenylmethylene)amino)-4,4-dimethyl-5-(pyrrolidin-1-yl)pentanoate (6v) was prepared according to the General Procedure G for 24 h as a light yellow oil (chromatography eluent: *n*-hexane/EtOAc = 2:1) in 70% yield (28.5 mg, 89% ee).

$[\alpha]_D^{25}$: -3.2 (c = 1.0, $CHCl_3$). HPLC analysis of the product: Daicel CHIRALPAK® AY3 column; 10% *i*-PrOH in *n*-hexane; 1.0 mL/min; retention times: 4.9 min (major), 4.3 min (minor).

^{1}H NMR (400 MHz, $CDCl_3$) δ 7.64 – 7.62 (m, 2H), 7.46 – 7.43 (m, 3H), 7.39 – 7.29 (m, 3H), 7.21 – 7.19 (m, 2H), 4.23 – 4.14 (m, 3H), 2.55 – 2.48 (s, 4H), 2.20 – 2.15 (m, 3H), 1.85 (dd, J = 14.1, 6.8 Hz, 1H), 1.67 (s, 4H), 1.26 (t, J = 7.1 Hz, 3H), 0.78 (d, J = 8.3 Hz, 6H).

^{13}C NMR (101 MHz, $CDCl_3$) δ 173.3, 169.3, 139.8, 136.7, 130.3, 128.9, 128.8, 128.5, 128.1, 127.9, 68.5, 63.6, 60.9, 57.0, 44.6, 35.4, 26.6, 26.1, 24.1, 14.3.

HRMS (ESI) Calcd for $C_{26}H_{35}N_2O_2^+$ [M + H]$^+$: 407.2693, Found:407.2692.

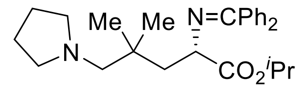

6w

Isopropyl (*S*)-2-((diphenylmethylene)amino)-4,4-dimethyl-5-(pyrrolidin-1-yl)pentanoate (6w) was prepared according to the General Procedure G for 24 h as a light yellow oil (chromatography eluent: *n*-hexane/EtOAc = 2:1) in 63% yield (26.5 mg, 93% ee).

$[\alpha]_D^{25}$: -6.0 (c = 1.0, $CHCl_3$). HPLC analysis of the product: Daicel CHIRALPAK® AY3 column; 10% *i*-PrOH in *n*-hexane; 1.0 mL/min; retention times: 4.9 min (major), 4.1 min (minor).

^{1}H NMR (400 MHz, $CDCl_3$) δ 7.64 – 7.62 (m, 2H), 7.46 – 7.44 (m, 3H), 7.40 – 7.30 (m, 3H), 7.21 – 7.19 (m, 2H), 5.02 (p, J = 6.3 Hz, 1H), 4.16 (dd, J = 6.7, 5.4 Hz, 1H), 2.53 – 2.49 (m, 4H), 2.19 – 2.13 (m, 3H), 1.84 (dd, J = 14.1, 6.7 Hz, 1H), 1.68 – 1.65 (m, 4H), 1.23 (dd, J = 6.3, 3.1 Hz, 6H), 0.77 (d, J = 5.6 Hz, 6H).

^{13}C NMR (101 MHz, $CDCl_3$) δ 172.8, 169.3, 139.8, 136.8, 130.2, 128.9, 128.7, 128.5, 128.1, 128.0, 68.5, 68.2, 63.7, 57.0, 44.5, 35.5, 26.6, 26.1, 24.2, 21.8.

HRMS (ESI) Calcd for $C_{27}H_{37}N_2O_2^+$ [M + H]$^+$: 421.2850, Found:421.2852.

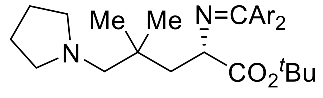

Ar = *p*-ClC$_6$H$_4$

6x

***tert*-Butyl (*S*)-2-((bis(4-chlorophenyl)methylene)amino)-4,4-dimethyl-5-(pyrrolidin-1-yl)pentanoate (6x)** was prepared according to the General Procedure E for 48 h as a light yellow oil (chromatography eluent: *n*-hexane/EtOAc = 2:1) in 47% yield (70.8 mg, 24% ee).

$[\alpha]_D^{25}$: -83.4 (c = 1.0, $CHCl_3$). HPLC analysis of the product: Daicel CHIRALPAK® IE3 column; 3% *i*-PrOH in *n*-hexane; 1.0 mL/min; retention

times: 7.5 min (major), 6.4 min (minor).

^{1}H NMR (400 MHz, $CDCl_3$) δ 7.55 (d, *J* = 8.6 Hz, 2H), 7.44 (d, *J* = 8.3 Hz, 2H), 7.29 (d, *J* = 8.6 Hz, 2H), 7.15 (d, *J* = 8.3 Hz, 2H), 4.02 (dd, *J* = 6.6, 5.2 Hz, 1H), 2.54 - 2.44 (m, 4H), 2.18 – 2.12 (m, 3H), 1.82 (dd, *J* = 14.1, 6.8 Hz, 1H), 1.70 - 1.62 (m, 4H), 1.44 (s, 9H), 0.76 (d, *J* = 6.8 Hz, 6H).

^{13}C NMR (101 MHz, $CDCl_3$) δ 171.3, 165.8, 137.2, 135.9, 134.3, 134.0, 129.4, 128.7, 128.2, 127.7, 80.4, 67.9, 63.7, 56.4, 43.6, 34.8, 27.4, 25.9, 25.4, 23.5.

HRMS (ESI) Calcd for $C_{28}H_{37}Cl_2N_2O_2^+$ [M + H]$^+$: 503.2227, Found:503.2236.

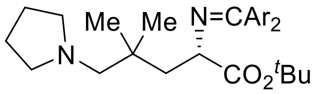

Ar = *p*-OMeC$_6$H$_4$

6y

***tert*-Butyl (*S*)-2-((bis(4-methoxyphenyl)methylene)amino)-4,4-dimethyl-5-(pyrrolidin-1-yl)pentanoate (6y)** was prepared according to the General Procedure E for 72 h as a light yellow oil (chromatography eluent: *n*-hexane/EtOAc = 1:1) in 82% yield (121.6 mg, 99% ee).

$[\alpha]_D^{25}$: -135.6 (c = 1.0, $CHCl_3$). HPLC analysis of the product: Daicel CHIRALPAK® IE3 column; 10% *i*-PrOH in *n*-hexane; 1.0 mL/min; retention times: 16.3 min (major), 11.9 min (minor).

^{1}H NMR (400 MHz, $CDCl_3$) δ 7.59 (d, *J* = 8.9 Hz, 2H), 7.14 (d, *J* = 8.7 Hz, 2H), 6.96 (d, *J* = 8.7 Hz, 2H), 6.83 (d, *J* = 8.9 Hz, 2H), 4.08 (dd, *J* = 6.8, 5.0 Hz, 1H), 3.87 (s, 3H), 3.81 (s, 3H), 2.53 - 2.46 (m, 4H), 2.20 – 2.07 (m, 3H), 1.81 (dd, *J* = 14.0, 6.9 Hz, 1H), 1.74 – 1.58 (m, 4H), 1.45 (s, 9H), 0.77 (d, *J* = 9.6 Hz, 6H).

^{13}C NMR (101 MHz, $CDCl_3$) δ 172.1, 167.3, 160.5, 158.9, 132.6, 129.9, 128.8, 128.6, 113.0, 112.6, 79.9, 67.8, 63.4, 56.3, 54.7, 43.9, 34.8, 27.4, 25.9, 25.4, 23.5.

HRMS (ESI) Calcd for $C_{30}H_{43}N_2O_4^+$ [M + H]$^+$: 495.3217, Found:495.3222.

V. Large-Scale Reactions

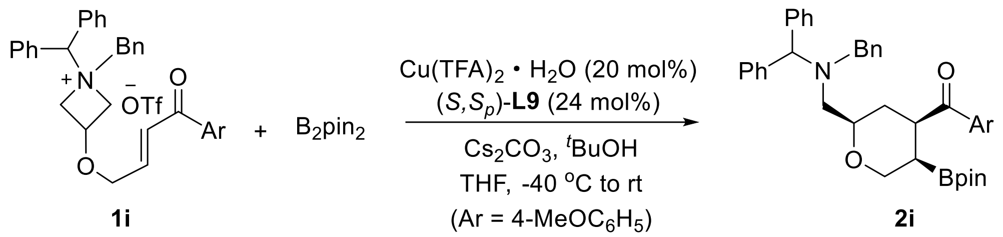

In a glove box, a mixture of Cu(TFA)$_2$·H$_2$O (57.9 mg, 0.2 mmol, 20 mol%) and the chiral ligand (*S*,*S*$_p$)-**L9** (118.9 mg, 0.24 mmol, 24 mol%) in dry THF (0.2 M, 5.0 mL) was stirred at room temperature for 0.5 h. Then, azetidinium **1i** (604.0 mg, 1.2 mmol, 1.2 equiv), Cs$_2$CO$_3$ (651.6 mg, 2.0 mmol, 2.0 equiv), and ^{t}BuOH (191.5 uL, 2.0 mmol, 2.0 equiv) were added sequentially. The mixture was cooled to -40 °C and stirred for 10 min before a solution of B$_2$pin$_2$ (253.5 mg, 1.0 mmol, 1.0 equiv) in dry THF (5.0 mL) was added dropwise. The mixture was stirred at -40 °C for 0.5 h before it was warmed to room temperature and stirred for 5 h. Next, the mixture was concentrated *in vacuo*, and the residue was purified by column chromatography on silica gel to give the desired product **2i** (chromatography eluent: *n*-hexane/EtOAc = 10:1, 442.0 mg, 70% yield, 95% ee, >20:1 dr).

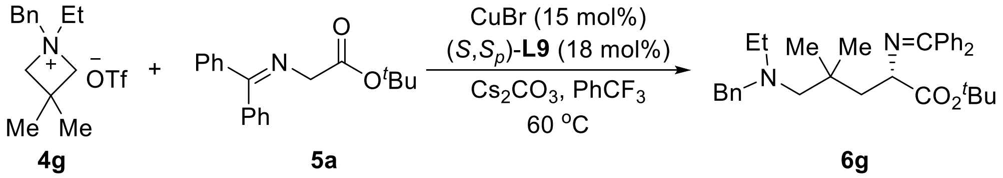

In a glove box, CuBr (21.5 mg, 0.15 mmol, 15 mol%) and chiral ligand (*S*,*S*$_p$)-**L9** (89.2 mg, 0.18 mmol, 18 mol%) were dissolved in dry PhCF$_3$ (0.2 M, 5 mL). The mixture was stirred at room temperature for 0.5 h before the keto imine ester **5a** (442.8 mg, 1.5 mmol, 1.5 equiv.), Cs$_2$CO$_3$ (651.6 mg, 1.0 mmol, 2.0 equiv.), and azetidinium **4g** (353.1 mg, 1.0 mmol, 1.0 equiv) were added sequentially. The reaction mixture was stirred at 60 °C for 168 h. before it was concentrated

in vacuo. The residue was then purified by column chromatography on silica gel[4] to give the desired product **6g** (chromatography eluent: *n*-hexane/EtOAc = 10:1, 309.2 mg, 62% yield, 90% ee).

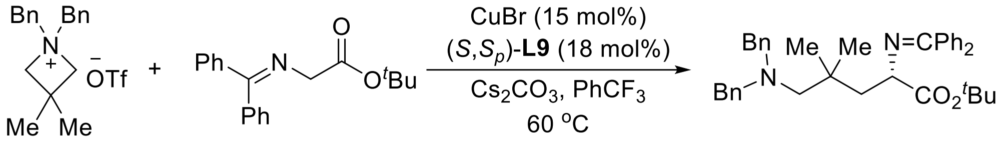

4h **5a** **6h**

In a glove box, CuBr (21.5 mg, 0.15 mmol, 15 mol%) and chiral ligand (S,S_p)-**L9** (89.2 mg, 0.18 mmol, 18 mol%) were dissolved in dry $PhCF_3$ (0.2 M, 5 mL). the mixture was stirred at room temperature for 0.5 h before the keto imine ester **5a** (442.8 mg, 1.5 mmol, 1.5 equiv), Cs_2CO_3 (651.6 mg, 1.0 mmol, 2.0 equiv), and azetidinium **4h** (415.1 mg, 1.0 mmol, 1.0 equiv) were added sequentially. The reaction mixture was stirred at 60 °C for 168 h before it was concentrated *in vacuo*. The residue was then purified by column chromatography on silica gel[4] to give the desired product **6h** (chromatography eluent: *n*-hexane/EtOAc = 10:1, 470.7 mg, 84% yield, 98% ee).

VI. Product Transformations

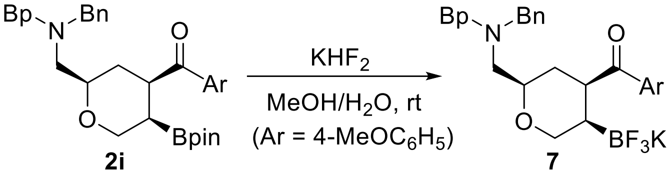

2i **7**

((2*R*,4*R*,5*R*)-2-((Benzhydryl(benzyl)amino)methyl)-5-(trifluoro-l4 boranyl) tetrahydro-2*H*-pyran-4-yl)(4-methoxyphenyl)methanone, potassium salt (7). A mixture of **2a** (63.2 mg, 0.1 mmol, 1.0 equiv) and KHF_2 (78.0 mg, 1.0 mmol, 10.0 equiv) in MeOH/H_2O (1.1 mL, v/v = 10:1) was stirred at room temperature for 13 h. The mixture was then concentrated *in vacuo* and then acetone was added. Next, the mixture was filtered through celite. The filtrate was concentrated, and the residue was recrystallized with *n*-hexane and Et_2O to afford the desired product **7** as a white solid (50.7 mg, 83% yield).

$[\alpha]_D^{25}$: -12.2 (c = 1.0, CH_2Cl_2).

^{1}H NMR (400 MHz, $CDCl_3$) δ 7.91 (d, *J* = 8.9 Hz, 2H), 7.43 – 7.35 (m, 6H), 7.34 – 7.27 (m, 5H), 7.27 – 7.16 (m, 4H), 6.96 (d, *J* = 8.9 Hz, 2H), 5.68 (s, 2H), 4.99 (s, 1H), 4.28 (d, *J* = 10.6 Hz, 1H), 3.89 (s, 3H), 3.77 (d, *J* = 14.2 Hz, 1H), 3.66 (d, *J* = 14.2 Hz, 1H), 3.65 – 3.53 (m, 2H), 2.71 (dd, *J* = 13.8, 6.6 Hz, 1H), 2.56 (dd, *J* = 13.8, 4.8 Hz, 1H), 1.68 (d, *J* = 13.9 Hz, 1H), 1.41 (d, *J* = 8.0 Hz, 1H).

^{13}C NMR (101 MHz, $CDCl_3$) δ 202.6, 164.1, 141.7, 140.9, 140.3, 131.1, 129.3, 129.0, 128.8, 128.5, 128.4, 128.3, 128.3, 127.2, 127.1, 127.0, 114.1, 77.3, 71.2, 70.4, 56.3, 55.7, 55.7 (2C), 45.8, 32.1.

^{19}F NMR (377 MHz, $CDCl_3$) δ -78.11.

HRMS (ESI) Calcd for $C_{34}H_{34}BF_3NO_3^-$ [M - K]$^-$: 572.2589, Found: 527.2587.

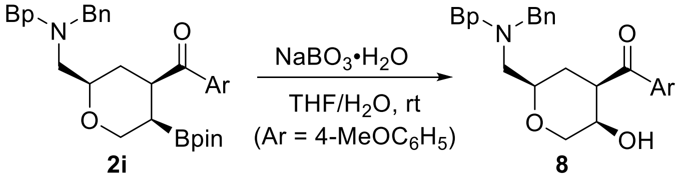

((2*R*,4*R*,5*R*)-2-((Benzhydryl(benzyl)amino)methyl)-5-hydroxytetrahydro-2*H*-pyran-4-yl)(4-methoxyphenyl)methanone (8). A mixture of **2i** (126.3 mg, 0.2 mmol, 1.0 equiv) and $NaBO_3 \cdot H_2O$ (99.8 mg, 1.0 mmol, 5.0 equiv) in THF/H_2O (2.0 mL, v/v = 1:1) was stirred at room temperature for 2 h before it was diluted with H_2O (10 mL) and extracted with ethyl acetate (10 mL × 3). The combined organic layers were washed with brine (10 mL), dried over Na_2SO_4, and concentrated *in vacuo*. The residue was purified by flash silica gel chromatography to afford the product **8** as a white solid (chromatography eluent: *n*-hexane/EtOAc = 10:1, 109.8 mg, 87% yield).

$[\alpha]_D^{25}$: +4.2 (c = 2.0, CH_2Cl_2).

^{1}H NMR (400 MHz, $CDCl_3$) δ 7.87 (d, *J* = 8.8 Hz, 2H), 7.4- - 7.17 (m, 15H), 6.97 (d, *J* = 8.8 Hz, 2H), 4.97 (s, 1H), 4.00 - 3.97 (m, 2H), 3.89 (s, 3H), 3.79 (d, *J* = 14.2 Hz, 1H), 3.63 (d, *J* = 14.2 Hz, 1H), 3.50 – 3.40 (m, 2H), 3.37 – 3.27 (m, 1H), 2.82 – 2.55 (m, 2H), 1.80 – 1.69 (m, 2H), 1.22 (s, 1H).

^{13}C NMR (101 MHz, $CDCl_3$) δ 201.3, 163.9, 141.7, 140.3, 140.2, 130.7, 129.3, 128.8, 128.6, 128.2, 128.1, 128.1, 127.0, 126.8, 126.8, 114.0, 76.4, 71.8, 70.2, 65.5, 56.4, 55.7, 55.51, 45.5, 28.0, 24.8.

HRMS (ESI) Calcd for $C_{34}H_{36}NO_4^+$ [M + H]$^+$: 522.2639, Found:522.2648.

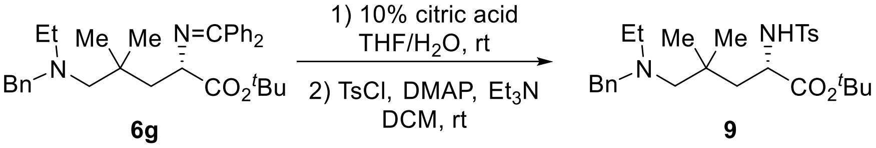

***tert*-Butyl (*S*)-5-(benzyl(ethyl)amino)-4,4-dimethyl-2-((4-methylphenyl) sulfonamido)pentanoate (9).** In a 10-mL vial, to a solution of keto imine ester **6g** (0.3 mmol, 149.5 mg, 1.0 equiv) in THF (2 mL) was added an aqueous solution of citric acid (2 mL, 10 wt%). The mixture was stirred for 2 h before it

was neutralized with solid K_2CO_3 and extracted with EtOAc (10 mL × 3).The combined organic layers were dried over $MgSO_4$ and concentrated *in vacuo*. The residue was dissolved in DCM (4 mL) and treated with TsCl (0.33 mmol, 62.9 mg, 1.1 equiv), Et_3N (0.45 mmol, 62.5 μL, 1.5 equiv), and DMAP (0.03 mmol, 3.7 mg, 10 mol%). The mixture was stirred for another 12 h and then concentrated *in vacuo*. The residue was purified by flash column chromatography (EtOAc/*n*-hexane = 10:1) to give ester **9** (134.7 mg, 92% yield).

^{1}H NMR (400 MHz, $CDCl_3$) δ 7.73 (d, *J* = 8.2 Hz, 2H), 7.36 – 7.21 (m, 7H), 6.00 (s, 1H), 3.87 (dd, *J* = 9.5, 2.5 Hz, 1H), 3.66 (s, 2H), 2.50 (q, *J* = 6.9 Hz, 2H), 2.39 (s, 3H), 2.34 (d, *J* = 2.6 Hz, 2H), 1.67 (dd, *J* = 14.4, 3.0 Hz, 1H), 1.46 (dd, *J* = 14.4, 9.7 Hz, 1H), 1.20 (s, 9H), 1.02 – 0.91 (m, 9H).

^{13}C NMR (101 MHz, $CDCl_3$) δ 171.3, 142.7, 139.2, 136.4, 128.9, 128.1, 127.5, 126.9, 126.2, 81.1, 64.4, 59.2, 53.5, 48.3, 42.6, 35.6, 26.9, 26.0, 25.4, 20.8, 10.5.

HRMS (ESI) Calcd for $C_{27}H_{41}N_2O_4S^+$ [M + H]$^+$: 489.2782, Found:489.2784.

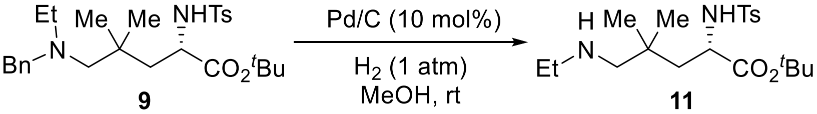

***tert*-Butyl (*S*)-5-(ethylamino)-4,4-dimethyl-2-((4-methylphenyl) sulfon amido)pentanoate (11).** To a mixture of amino ester **9** (0.1 mmol, 48.8 mg, 1.0 equiv) and Pd/C (10 wt%, 1.0 μmol, 10.6 mg, 10 mol%) in anhydrous degassed MeOH (1 mL) was bubbled H_2 gas for 1 h. The reaction mixture was then stirred under H_2 atmosphere (1 atm) for 18 h before it was filtrated through a pad of celite, which was rinsed with EtOAc (10 mL). The filtrate was concentrated under reduced pressure, and the residue was purified by flash column chromatography (EtOAc/*n*-hexane = 1:1) to afford the desired product **11** (35.5 mg, 89% yield, 90% ee).

$[\alpha]_D^{25}$: -1.0 (*c* = 1.0, $CHCl_3$). HPLC analysis of the product: Daicel CHIRALPAK® IA3 column; 10% *i*-PrOH in *n*-hexane; 1.0 mL/min; retention times: 6.7 min

(major), 6.4 min (minor).

^{1}H NMR (400 MHz, $CDCl_3$) δ 7.75 (d, J = 8.3 Hz, 2H), 7.32 – 7.25 (m, 2H), 3.95 – 3.91 (m, 1H), 3.30 – 2.97 (m, 2H), 2.61 (d, J = 12.4 Hz, 1H), 2.41 (s, 3H), 2.33 – 2.12 (m, 1H), 1.45 (t, J = 7.2 Hz, 3H), 1.35 – 1.23 (m, 2H), 1.24 – 1.02 (m, 15H).

^{13}C NMR (101 MHz, $CDCl_3$) δ 143.3, 137.2, 129.5, 127.4, 81.8, 54.2, 44.3, 40.6, 33.7, 29.0, 27.8, 27.5, 25.8, 21.5.

HRMS (ESI) Calcd for $C_{20}H_{35}N_2O_4S^+$ $[M + H]^+$: 399.2312, Found:399.2316.

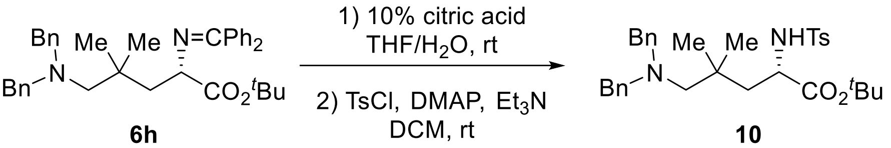

6h   10

***tert*-Butyl (*S*)-5-(dibenzylamino)-4,4-dimethyl-2-((4-methylphenyl) sulfon amide) pentanoate (10).** To a 10-mL vial charged with keto imine ester **6h** (0.3 mmol, 168.1 mg, 1.0 equiv) and THF (2 mL) was added a citric acid solution (2 mL, 10 wt%). The mixture was stirred for 2 h before it was neutralized with K_2CO_3 and extracted with EtOAc (10 mL × 3). The combined organic layers were dried over $MgSO_4$ and concentrated *in vacuo*. The residue was dissolved in DCM (4 mL), to which were then added TsCl (0.33 mmol, 62.9 mg, 1.1 equiv), Et_3N (0.45 mmol, 62.5 μL, 1.5 equiv), and DMAP (0.03 mmol, 3.7 mg, 10 mol%). The mixture was stirred for another 12 h before it was concentrated *in vacuo*. The residue was purified by flash column chromatography (EtOAc/*n*-hexane = 5:1) to give the desired amine ester **10** (153.5 mg, 93% yield).

^{1}H NMR (400 MHz, $CDCl_3$) δ 7.70 (d, J = 8.3 Hz, 2H), 7.36 – 7.19 (m, 12H), 5.01 (d, J = 10.4 Hz, 1H), 3.81 (td, J = 9.9, 2.8 Hz, 1H), 3.70 – 3.43 (m, 4H), 2.42 (d, J = 2.4 Hz, 2H), 2.37 (s, 3H), 1.54 (dd, J = 14.5, 2.8 Hz, 1H), 1.24 – 1.16 (m, 10H), 0.92 (d, J = 2.9 Hz, 6H).

^{13}C NMR (101 MHz, $CDCl_3$) δ 171.1, 142.8, 139.0, 136.2, 129.0, 129.0, 127.5, 126.9, 126.3, 81.1, 64.5, 59.8, 53.2, 42.8, 35.5, 26.9, 25.4, 25.3, 20.8.

HRMS (ESI) Calcd for $C_{32}H_{43}N_2O_4S^+$ $[M + H]^+$: 551.2938, Found: 551.2941.

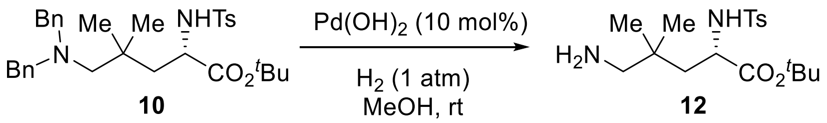

***tert*-Butyl (*S*)-5-amino-4,4-dimethyl-2-((4-methylphenyl) sulfon amido) pentanoate (12).** To a mixture of amino ester **10** (0.1 mmol, 34.9 mg, 1.0 equiv) and $Pd(OH)_2$ (10% on carbon, 1.0 μmol, 14.0 mg, 10.0 mol%) in anhydrous degassed MeOH (1 mL) was bubbled H_2 gas for 1 h. the reaction mixture was then stirred under H_2 atmosphere (1 atm) for 18 h before it was filtrated through a pad of celite, which was rinsed with EtOAc (10 mL). The filtrate was concentrated under reduced pressure, and the residue was purified by flash column chromatography (EtOAc/*n*-hexane = 1:1) to afford the desired product **12** (32.6 mg, 88% yield, 93% ee).

$[\alpha]_D^{25}$: -0.72 (*c* = 1.0, $CHCl_3$). HPLC analysis of the product: Daicel CHIRALPAK® OD3 column; 10% *i*-PrOH in *n*-hexane; 1.0 mL/min; retention times: 13.0 min (major), 10.9 min (minor).

^{1}H NMR (400 MHz, $CDCl_3$) δ 7.74 (d, J = 7.5 Hz, 2H), 7.26 (d, J = 7.2 Hz, 2H), 5.59 (s, 3H), 3.86 (d, J = 10.2 Hz, 1H), 3.08 (d, J = 12.8 Hz, 1H), 2.67 (d, J = 12.3 Hz, 1H), 2.38 (s, 3H), 1.99 – 1.83 (m, 1H), 1.47 (t, J = 12.2 Hz, 1H), 1.20 (s, 9H), 1.05 (d, J = 16.3 Hz, 6H).

^{13}C NMR (101 MHz, $CDCl_3$) δ 170.8, 143.3, 137.1, 129.5, 127.5, 81.8, 54.0, 49.4, 40.5, 34.0, 27.6, 27.4, 25.0, 21.4.

HRMS (ESI) Calcd for $C_{18}H_{31}N_2O_4S^+$ $[M + H]^+$: 371.1999, Found: 371.2004.

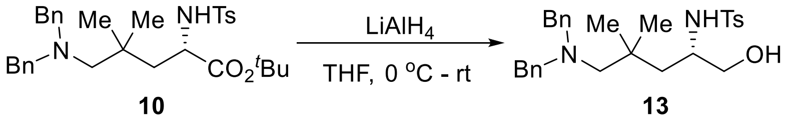

(*S*)-*N*-(5-(Dibenzylamino)-1-hydroxy-4,4-dimethylpentan-2-yl)-4-methyl benzenesulfonamide (13). At 0 °C, to a solution of amino ester **10** (0.1 mmol, 55.0 mg, 1.0 equiv) in dry THF (2 mL) was added $LiAlH_4$ (0.15 mmol, 5.7 mg,

1.5 equiv). The mixture was stirred for 10 min before it was warmed to room temperature and then stirred for 12 h. The reaction mixture was quenched with brine and extracted with EtOAc (3 × 5 mL). The combined organic layers were dried over $MgSO_4$, filtered, and then concentrated under reduced pressure. The crude product was purified by flash column chromatography (EtOAc/*n*-hexane = 1:1) to give amino alcohol **13** (34.6 mg, 72% yield, 96% ee).

$[\alpha]_D^{25}$: -0.2 (*c* = 1.0, $CHCl_3$). HPLC analysis of the product: Daicel CHIRALPAK® IA3 column; 10% *i*-PrOH in *n*-hexane; 1.0 mL/min; retention times: 15.4 min (major), 16.3 min (minor).

^{1}H NMR (400 MHz, $CDCl_3$) δ 7.79 (d, *J* = 8.0 Hz, 2H), 7.37 – 7.28 (m, 12H), 5.91 (s, 1H), 3.75 – 3.50 (m, 4H), 3.46 – 3.19 (m, 3H), 2.44 (s, 3H), 2.30 (q, *J* = 13.9 Hz, 3H), 1.34 – 1.01 (m, 2H), 0.73 (d, *J* = 10.7 Hz, 6H).

^{13}C NMR (101 MHz, $CDCl_3$) δ 143.4, 138.7, 137.8, 129.7 (3 C), 128.2, 127.2, 66.5, 64.5, 60.2, 52.7, 42.1, 35.1, 27.4, 26.0, 21.6.

HRMS (ESI) Calcd for $C_{28}H_{37}N_2O_3S^+$ $[M + H]^+$: 481.2519, Found: 481.2518.

VII. Mechanistic Experiments

(a) Effect of the Counter Anion

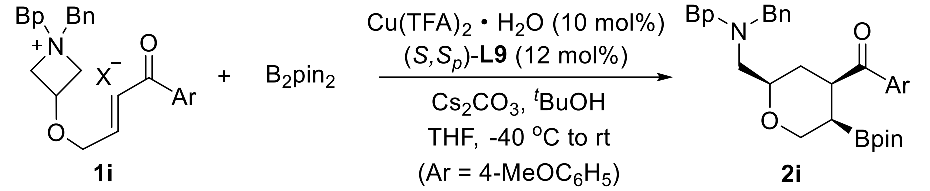

X⁻	yield (%)	ee (%)	dr
OTf	85	98	>20:1
BF_4	77	92	>20:1
SbF_6	55	95	>20:1

The above reactions were performed according to the General Procedure D (0.05 mmol scale). The yield and dr values of product **2i** were determined by crude NMR spectroscopy with dibromomethane as the internal standard. The ee value was determined by chiral HPLC.

(b) Synthesis and Evaluation of Compound INT

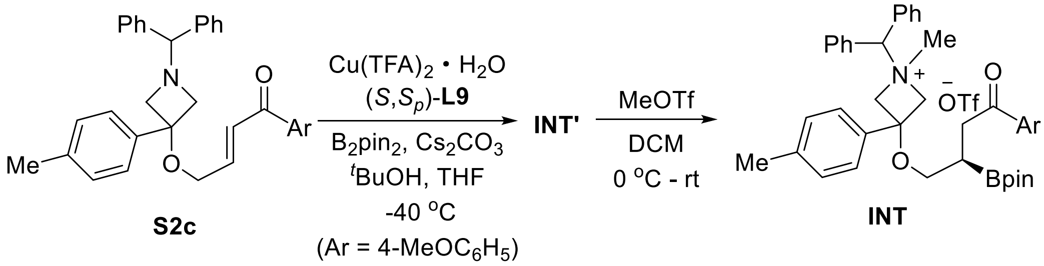

INT':

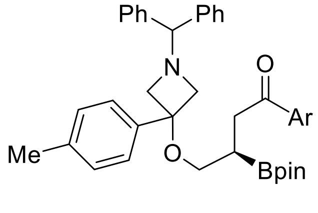

In a glove box, a mixture of $Cu(TFA)_2{\cdot}H_2O$ (23.2 mg, 0.08 mmol, 20 mol%) and the chiral ligand (S,S_p)-**L9** (47.6 mg, 0.096 mmol, 24 mol%) in dry THF (0.2 M, 2.0 mL) was stirred at room temperature for 0.5 h. Then, azetidine **S2c** (241.5 mg, 0.48 mmol, 1.2 equiv), Cs_2CO_3 (260.6 mg, 0.8 mmol, 2.0 equiv), and ^{t}BuOH (76.6 μL, 0.8 mmol, 2.0 equiv) were added sequentially. The mixture was stirred

at -40 °C for 10 min before a solution of B_2pin_2 (101.4 mg, 0.4 mmol, 1.0 equiv) in dry THF (2.0 mL) was added dropwise. The mixture was stirred at -40 °C for 0.5 h before it was warmed to room temperature and stirred for 0.5 h. The mixture was concentrated *in vacuo*. The residue was then purified by flash column chromatography on silica gel to give the desired product **INT'**. Next, the General Procedure B was followed to prepare the key intermediate **INT** (260.9 mg, 82% yield, 96% ee).

HPLC analysis of the product **INT'**: Daicel CHIRALPAK® AD3 column; 10% *i*-PrOH in *n*-hexane; 1.0 mL/min; retention times: 9.9 min (major), 7.1 min (minor).

^{1}H NMR (400 MHz, $CDCl_3$) two isomers δ 7.88 (dd, *J* = 17.4, 8.5 Hz, 2H), 7.75 – 7.55 (m, 3.91H), 7.42 (dd, *J* = 22.5, 7.5 Hz, 6.07H), 7.22 – 7.09 (m, 0.85H), 6.99 – 6.78 (m, 3.58H), 6.53 (d, *J* = 7.8 Hz, 1.40H), 6.24 (s, 0.67H), 6.20 (s, 0.18H), 5.17 – 4.96 (m, 1.82H), 4.77 (dd, *J* = 31.6, 11.8 Hz, 0.53H), 4.55 (t, *J* = 8.1 Hz, 1.40H), 3.91 (s, 0.62H), 3.87 (s, 2.26H), 3.64 (s, 2.22H), 3.35 – 3.25 (m, 0.80H), 3.17 (td, *J* = 21.1, 18.4, 9.7 Hz, 2.75H), 2.29 (s, 0.56H), 2.21 (s, 2.05H), 1.65 (t, *J* = 6.3 Hz, 0.86H), 1.27 – 1.07 (m, 11.78H).

^{13}C NMR (101 MHz, $CDCl_3$) two isomers δ 198.1, 163.5, 138.8, 130.4, 130.3, 130.3, 130.2, 130.0, 130.0, 129.7, 129.5, 126.4, 124.8, 120.9 (d, J_{C-F} = 320.3 Hz). 113.7, 83.5, 77.6, 74.8, 70.1, 69.5, 64.9, 55.5, 53.2, 36.8, 24.8, 24.7, 20.9.

HRMS (ESI) Calcd for $C_{41}H_{49}BNO_5^+$ $[M + H]^+$: 646.3698, Found: 646.3699.

The reactivity of compound **INT** was examined by the following reactions.

Reaction (a)

In a glove box, a mixture of **INT** (0.05 mmol, 32.3 mg, 1.0 equiv) and Cs_2CO_3 (32.6 mg, 0.1 mmol, 2.0 equiv) in dry THF (0.2 M, 0.25 mL) was stirred at 40 °C for 5 h. Next, the mixture was concentrated *in vacuo*, and the residue was filtered through a pad of silica gel to remove the polar compounds. After

evaporation of the solvent, the crude product **2k** was subject to ^{1}H NMR and HPLC analysis. The yield and dr values were determined by ^{1}H NMR spectroscopy using dibromomethane as the internal standard, and the ee value was determined by chiral HPLC.

Reactions (b–d)

In a glove box, a mixture of $Cu(TFA)_2{\cdot}H_2O$ (1.5 mg, 0.005 mmol, 10 mol%) and the ligand ((*S*,S_p)-**L9**, (*R*,R_p)-**L9**, or *rac*-**L9**) (3.0 mg, 0.006 mmol, 12 mol%) in dry THF (0.2 M, 0.25 mL) was stirred at room temperature for 0.5 h. Then, **INT** (0.05 mmol, 32.3 mg, 1.0 equiv), Cs_2CO_3 (32.6 mg, 0.1 mmol, 2.0 equiv), and ^{t}BuOH (9.6 uL, 0.1 mmol, 2.0 equiv) were added sequentially. The mixture was stirred at 40 ^{o}C for 5 h. Next, the mixture was concentrated *in vacuo*, and the residue was filtered through a pad of silica gel to remove the polar compounds. After evaporation of the solvent, the crude product **2k** was subject to ^{1}H NMR and HPLC analysis. The yield and dr values were determined by ^{1}H NMR spectroscopy using dibromomethane as the internal standard, and the ee value was determined by chiral HPLC.

(a) **INT**, 96% ee → (Cs_2CO_3; THF, 40 ^{o}C) → **2k,** 57% yield, 96% ee, >20:1 dr

(b) **INT**, 96% ee → ($Cu(TFA)_2 \cdot H_2O$ (10 mol%), (*S*,S_p)-**L9** (12 mol%); Cs_2CO_3, ^{t}BuOH, THF, 40 ^{o}C) → **2k,** 75% yield, 96% ee, >20:1 dr

(c) **INT**, 96% ee → ($Cu(TFA)_2 \cdot H_2O$ (10 mol%), *rac*-**L9** (12 mol%); Cs_2CO_3, ^{t}BuOH, THF, 40 ^{o}C) → **2k,** 66% yield, 96% ee, >20:1 dr

(d) **INT**, 96% ee → ($Cu(TFA)_2 \cdot H_2O$ (10 mol%), (*R*,R_p)-**L9** (12 mol%); Cs_2CO_3, ^{t}BuOH, THF, 40 ^{o}C) → **2k,** 65% yield, 96% ee, >20:1 dr

(c) Non-linear Effects

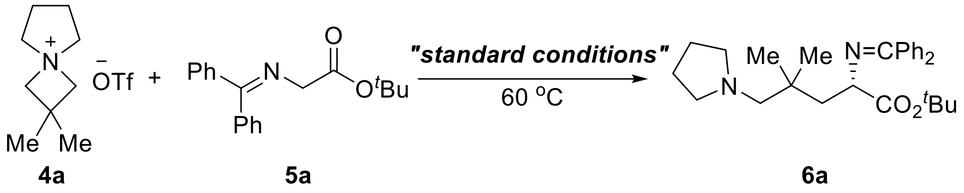

Under the standard conditions, the reaction was carried out using **L9** with different enantiopurities (1st run: 0% ee, 2nd run: 22% ee; 3rd run: 42% ee; 4th run: 64% ee; 5th run: 78% ee; 6th run: 100% ee). The ee values of the product **6a** were determined by HPLC.

ee% (**L9**)	0	22	42	64	78	100
ee% (**6a**)	0	18	40	62	76	96

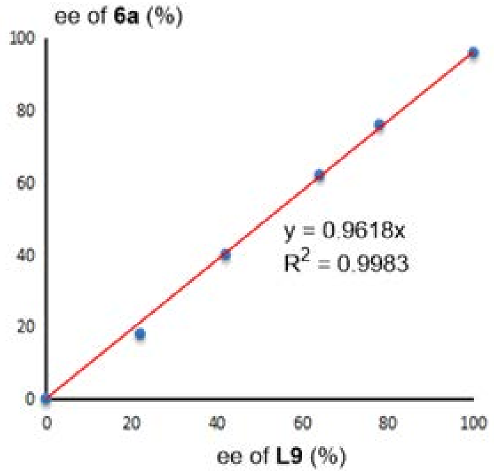

(d) Relationship between log(er of 6) and A-value of the Ester Substituent

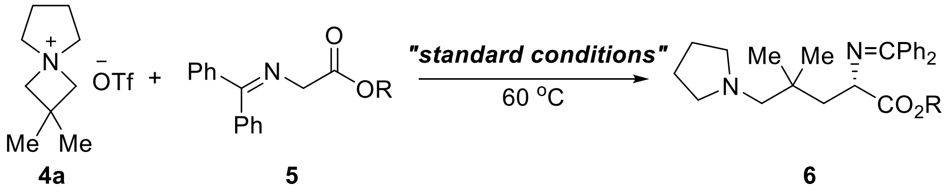

R	Log (er of **6**)	A-values (kcal/mol)
CH_3	1.03	1.8
CH_2CH_3	1.24	2.0
i-C_3H_7	1.44	2.2

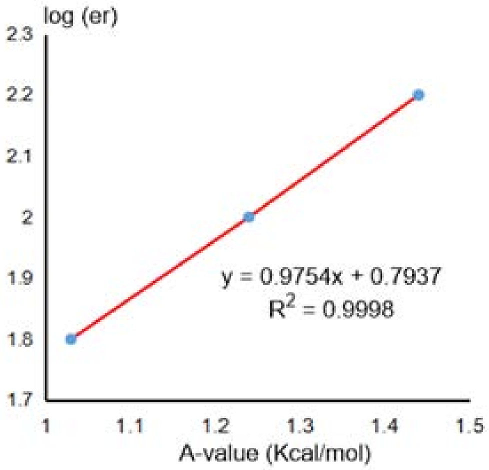
log (er)
2.3
2.2
2.1
2
1.9
1.8
1.7
1
1.1
1.2
1.3
1.4
1.5
A-value (Kcal/mol)
y = 0.9754x + 0.7937
R2 = 0.9998

VIII. DFT Calculation

Computational Details

All structures were optimized and characterized in experimental solvent ($PhCF_3$) with the SMD[5] solvent model (SCRF = SMD) at B3LYP[6]-D3[7]/BSI level, BSI represents a basis set with SDD[8] for Cu, Fe and 6-31G(d) for other atoms. The parameter definition for $PhCF_3$ in the SMD solvent model is as follows:

Eps = 9.4

EpsInf = 2.00183

HbondAcidity = 0.00

HbondBasicity = 0.10

SurfaceTensionAtInterface = 30.801448

CarbonAromaticity = 0.6

ElectronegativeHalogenicity = 0.3

Harmonic frequency analysis calculations at the same level were performed to verify the optimized geometries to be minima (no imaginary frequency) or transition states (TSs, having unique one imaginary frequency). The energies were further improved by M06[9]/BSII//B3LYP-D3/BSI single-point calculations with solvent effects accounted by the SMD solvent model, using $PhCF_3$, BSII denotes a basis set with SDD for Cu, Fe and 6-311++G(d,p) for other atoms. When necessary, intrinsic reaction coordinate (IRC) calculations[10] were carried out at the B3LYP-D3/BSI level to verify a transition state that correctly connects with its nearby minima (reactant and product). To analyze the origins of enantioselectivity, non-covalent interactions (NCIs) analyses were carried out. The cubic files for NCI analyses were generated with Multiwfn program,[11] and the results were visualized by the VMD program.[12] All DFT calculations were carried out using the Gaussian 09 program.[13] The 3D structures of enantioselective transition states were generated by CYLview 2.0.[14]

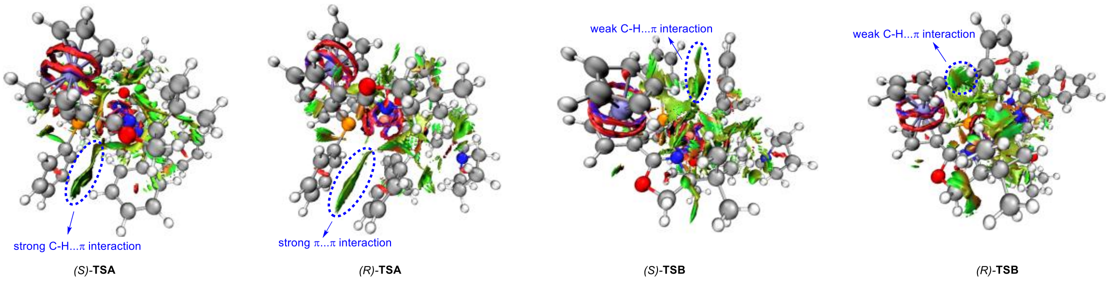

Figure S1. Non-covalent interaction analyses of the enantioselective transition states. Isovalue is 0.6.

Cartesian Coordinates in Å, SCF Energies and Free Energies (in a.u.) at 298.15 K and 1 atm for the Optimized Structures [BSI= 6-31G(d), BSII=6-311++G(d,p)]

(*S*)-**TSA**

B3LYP-D3/BSI SCF energy in $PhCF_3$ -3265.716608 a.u.

M06/BSII SCF energy in $PhCF_3$: -3264.302231 a.u.

M06/BSII free energy in $PhCF_3$: -3263.263953 a.u.

C	3.18375500	-1.89625300	0.50191500
C	4.54480400	-2.32686900	0.39124600
H	4.86284100	-3.32449000	0.12047400
C	5.38533700	-1.20726600	0.63971900
H	6.46610100	-1.20147600	0.57935100
C	3.18707200	-0.48104600	0.82800100
C	4.56022700	-0.08029600	0.91588200
H	4.90420300	0.92472300	1.11629900
C	3.34263300	-1.28603400	-2.83802600
H	2.66148700	-2.10474300	-3.02082200
C	4.76797100	-1.35550300	-2.86303400
H	5.35942400	-2.23921400	-3.06695500
C	5.27522300	-0.06406700	-2.52032800
H	6.31992600	0.20131500	-2.41609300
C	2.96536800	0.04144400	-2.47578000
H	1.95049800	0.38221000	-2.32895500
C	4.16193800	0.79875600	-2.28004600
H	4.21172900	1.83088600	-1.95882400
Fe	4.11444100	-0.79990700	-0.97074800
P	1.69844600	0.52856800	1.11580800
C	1.63323800	0.69884000	2.94666800

C	0.64946200	1.54821100	3.48109100
C	2.46938900	-0.00968400	3.81971500
C	0.50846000	1.69360900	4.85984000
H	-0.00540800	2.09784800	2.81071700
C	2.32361400	0.13233500	5.20380800
H	3.23587500	-0.67044800	3.42703400
C	1.34630000	0.98193300	5.72667600
H	-0.25502500	2.35672600	5.25805000
H	2.97838800	-0.42119100	5.87164900
H	1.23577100	1.08994200	6.80221900
C	2.20009900	2.21004000	0.56588700
C	1.66288600	2.69767000	-0.63494500
C	3.09585800	3.00927400	1.29446200
C	2.04622400	3.94937700	-1.12243500
H	0.94479400	2.09427900	-1.18032800
C	3.46962300	4.26430600	0.81050500
H	3.49344000	2.65694000	2.24189100
C	2.95328300	4.73237900	-0.40297600
H	1.63110300	4.31305800	-2.05856100
H	4.16304500	4.87714800	1.38009700
H	3.24965300	5.70817000	-0.77828000
C	2.02469100	-2.77217800	0.31891400
N	0.84602300	-2.44486400	-0.08913800
O	2.22974400	-4.07354400	0.62004300
C	0.90768600	-4.69557200	0.59387300
H	1.01270000	-5.68522400	0.15062900
H	0.57068600	-4.78950600	1.63132500
C	0.05238700	-3.69493200	-0.20605700
H	-0.90916300	-3.53043700	0.28128200

C	-0.22393200	-4.09003500	-1.68743100
C	-0.81845900	-2.88170000	-2.42873600
H	-0.10097800	-2.06150900	-2.50254600
H	-1.12692600	-3.16958900	-3.44132900
H	-1.69409300	-2.49240100	-1.90103300
C	-1.25094400	-5.23732700	-1.68367600
H	-2.19429600	-4.92046900	-1.22036600
H	-1.47017700	-5.55560100	-2.70994500
H	-0.88465100	-6.11489600	-1.13671300
C	1.06043100	-4.54152500	-2.40491300
H	1.84895900	-3.78763100	-2.31802100
H	1.45048500	-5.48579200	-2.00820700
H	0.86142600	-4.69454000	-3.47230500
Cu	0.03650300	-0.51094700	0.05961800
N	-2.06498000	-0.58337200	-0.04249400
C	-2.84262900	-1.35629700	0.67677100
C	-4.25360800	-1.69828800	0.32389000
C	-4.60436800	-2.14327000	-0.96430800
C	-5.26126100	-1.62540100	1.30299600
C	-5.92260300	-2.48543000	-1.26562200
H	-3.84156900	-2.23013700	-1.73084800
C	-6.58230300	-1.95441800	0.99726700
H	-5.00432600	-1.30411600	2.30812100
C	-6.91845200	-2.38384200	-0.28935200
H	-6.17167900	-2.83369400	-2.26452200
H	-7.34721300	-1.87933500	1.76559600
H	-7.94590000	-2.64498100	-0.52797900
C	-2.29353900	-1.94728600	1.92195800
C	-1.41980500	-1.21062000	2.74052900

C	-2.62077800	-3.26211300	2.30503700
C	-0.85786600	-1.77720900	3.88462600
H	-1.19159400	-0.18395100	2.48043800
C	-2.05154100	-3.83352600	3.44364600
H	-3.30528300	-3.84308400	1.69411600
C	-1.16409700	-3.09561500	4.23535300
H	-0.18967200	-1.18372000	4.50140600
H	-2.30196700	-4.85613000	3.71360000
H	-0.72687300	-3.54051000	5.12531100
C	-2.51195300	0.17188500	-1.11999900
H	-3.54099900	0.11117100	-1.44239400
C	-1.53682400	0.51102100	-2.11084000
O	-0.30089500	0.38833500	-1.96191900
O	-2.09041000	1.05955900	-3.21360800
C	-1.29004400	1.49742600	-4.36873900
C	-0.54629400	0.30400200	-4.97507800
H	-0.07439700	0.60817900	-5.91606400
H	0.22786600	-0.06621000	-4.30074500
H	-1.24668600	-0.51091900	-5.18977100
C	-0.33685500	2.62936400	-3.97014800
H	0.08482000	3.08438400	-4.87360400
H	-0.87820000	3.40767200	-3.41937900
H	0.48384000	2.26198700	-3.35383100
C	-2.36185200	2.01428300	-5.32981700
H	-1.89807200	2.36147200	-6.25893000
H	-3.07641400	1.22060100	-5.57234700
H	-2.91106400	2.84972200	-4.88158200
C	-1.70721300	4.31745300	1.87605900
C	-1.38130400	5.21001600	-0.34402500

C	-0.45800100	5.93042000	0.64825600
C	-0.29929500	4.90137400	1.77672100
H	-2.36951700	4.98803500	2.44102500
H	-1.74632400	3.32909900	2.34099400
H	-0.80166700	4.64102000	-1.08000800
H	-2.04748800	5.88754200	-0.88470800
H	0.49303400	6.21611400	0.19191900
H	-0.94556900	6.83523500	1.02985500
H	0.41777300	4.12733200	1.48651700
H	0.03192300	5.33703900	2.72358500
C	-3.65232200	4.20296300	0.26839100
C	-2.38640300	2.39118000	-0.26724900
C	-3.86268000	2.68837300	0.04897400
H	-3.90137000	4.77203600	-0.63086300
H	-4.20006500	4.62888600	1.11723700
H	-1.99286200	2.67532000	-1.23301200
H	-1.72162400	1.89975300	0.43039500
N	-2.17893300	4.24205500	0.46832000
C	-4.32728000	1.97922700	1.32645100
H	-5.35072500	2.28418900	1.57466000
H	-4.32496800	0.89675200	1.18579900
H	-3.68839200	2.21323000	2.18475700
C	-4.84696200	2.42657600	-1.09955700
H	-5.15079700	1.37778600	-1.15050300
H	-5.75380300	3.02335000	-0.94107900
H	-4.41597500	2.70898800	-2.06648400

(*R*)-**TSA**

B3LYP-D3/BSI SCF energy in $PhCF_3$ -3265.71161 a.u.

M06/BSII SCF energy in $PhCF_3$: -3264.298336 a.u.

M06/BSII free energy in $PhCF_3$: -3263.261137 a.u.

C	-3.50678200	0.13747200	-1.77413800
C	-4.88082900	-0.01777500	-2.16541100
H	-5.22091400	-0.53098800	-3.05364900
C	-5.69747000	0.56858300	-1.16352800
H	-6.77992600	0.56854900	-1.14903900
C	-3.48701200	0.83981300	-0.50171800
C	-4.85072600	1.09815900	-0.15016500
H	-5.18058100	1.57851100	0.75989100
C	-3.86484800	-2.91248500	-0.43789800
H	-3.29778300	-3.32058600	-1.26394200
C	-5.28807200	-2.85867000	-0.34177200
H	-5.99042700	-3.22596800	-1.07953100
C	-5.62133100	-2.18415200	0.87304600
H	-6.62138700	-1.94922900	1.21539800
C	-3.31531800	-2.26969400	0.71379800
H	-2.26240500	-2.10405700	0.89868600
C	-4.40394400	-1.82211500	1.52613200
H	-4.31627700	-1.26267600	2.44765600
Fe	-4.48800000	-0.94028700	-0.34390000
P	-1.97929500	1.28939100	0.41820000
C	-1.86171400	3.10748300	0.15121900
C	-1.25204800	3.92637300	1.11699400
C	-2.30603300	3.68528700	-1.04842500
C	-1.11614100	5.29798900	0.89749700
H	-0.89277000	3.49576900	2.04743300

C	-2.16404200	5.05725600	-1.26664600
H	-2.76732200	3.06678200	-1.81326400
C	-1.57581200	5.86857000	-0.29287700
H	-0.65079800	5.91974300	1.65790600
H	-2.51876900	5.49153500	-2.19760800
H	-1.47243600	6.93691000	-0.46243700
C	-2.45260600	1.14699100	2.18831900
C	-1.85618800	0.12364100	2.93936200
C	-3.38159800	2.00060400	2.80688000
C	-2.20320700	-0.06431000	4.27998100
H	-1.13403600	-0.53131000	2.46453200
C	-3.72730500	1.81166100	4.14555600
H	-3.82606500	2.81851000	2.24781300
C	-3.14209100	0.77595700	4.88280200
H	-1.74106700	-0.86562000	4.84892200
H	-4.44960100	2.47432100	4.61476000
H	-3.41244600	0.63154200	5.92542500
C	-2.40288700	-0.42166600	-2.55863900
N	-1.18226500	-0.66746600	-2.21108500
O	-2.75656200	-0.73476300	-3.82344300
C	-1.62454200	-1.41511700	-4.42396000
H	-1.92328200	-2.45350200	-4.58602400
H	-1.41730000	-0.94413000	-5.38606700
C	-0.48373000	-1.24733800	-3.39184500
H	0.23547300	-0.49868400	-3.74639800
C	0.30725100	-2.55506700	-3.09931600
C	1.56268000	-2.23016100	-2.27513700
H	1.29792700	-1.80148500	-1.30805500
H	2.13839100	-3.14463100	-2.08701400

H	2.21349300	-1.52524900	-2.80667600
C	0.76435500	-3.15678600	-4.44356900
H	1.33424700	-2.42509200	-5.03132600
H	1.41564000	-4.01982400	-4.26213100
H	-0.07386800	-3.50575000	-5.05720100
C	-0.55671900	-3.56085700	-2.32147400
H	-0.81765700	-3.16105700	-1.33682400
H	-1.48259900	-3.80726000	-2.85502700
H	-0.00442600	-4.49679700	-2.17372500
Cu	-0.39790300	-0.08179100	-0.38538500
N	1.50998000	0.40227400	0.43780400
C	2.02185700	1.60184400	0.31409000
C	2.92254500	2.22286400	1.33017300
C	4.14777400	2.80001900	0.95164600
C	2.55017300	2.25946600	2.68549400
C	4.99308400	3.36698900	1.90628800
H	4.43705400	2.79426100	-0.09549900
C	3.39088100	2.83612500	3.63820000
H	1.59526900	1.83787500	2.98774100
C	4.61823000	3.38463400	3.25336500
H	5.94199300	3.79778400	1.59781600
H	3.08594600	2.86028200	4.68103900
H	5.27450200	3.82900400	3.99673300
C	1.69642500	2.38434400	-0.90282800
C	1.63183000	3.78896200	-0.87792800
C	1.45769000	1.72822600	-2.12688900
C	1.30846000	4.50823900	-2.02849600
H	1.81098800	4.31716200	0.05249900
C	1.13311600	2.44713900	-3.27573000

H	1.53720100	0.64807300	-2.16855600
C	1.05298600	3.84286300	-3.23112700
H	1.24244700	5.59144400	-1.98132800
H	0.95481900	1.91766200	-4.20864900
H	0.80164700	4.40665600	-4.12550200
C	1.95842600	-0.54902700	1.35051500
H	2.53217300	-0.26138600	2.22277700
C	1.09938500	-1.69944500	1.50389100
O	0.06370200	-1.90505600	0.83515600
O	1.52997200	-2.55239700	2.45431000
C	0.77843500	-3.75845300	2.85860100
C	0.52330700	-4.69631500	1.67424200
H	0.09869500	-5.63305100	2.05303400
H	-0.17273100	-4.26028300	0.95744300
H	1.45799100	-4.93625200	1.15663800
C	-0.52170600	-3.33228100	3.54236200
H	-1.01895800	-4.21187700	3.96627100
H	-0.30819700	-2.63335400	4.35799100
H	-1.20648900	-2.85828500	2.83755900
C	1.73064000	-4.41182900	3.86158500
H	1.27679600	-5.32060300	4.27034500
H	2.67448300	-4.68295000	3.37679400
H	1.94803300	-3.72902900	4.68995500
C	5.42426800	-1.57530300	-2.30063200
C	6.19660400	0.35297600	-1.18922700
C	5.44281500	0.88410800	-2.41128500
C	5.14581100	-0.38822300	-3.25262000
H	6.36098700	-2.08943200	-2.56179800
H	4.62258100	-2.31773400	-2.28409400

H	6.09058100	0.97216100	-0.29404100
H	7.27017900	0.22227700	-1.39886900
H	4.50690600	1.35419900	-2.08997700
H	6.02193800	1.63192900	-2.95963000
H	4.11041100	-0.39420000	-3.60563100
H	5.79175400	-0.44904500	-4.13279700
C	6.03797900	-1.79389600	0.15603600
C	3.88280900	-0.97854500	0.17488400
C	4.70234400	-2.06005200	0.89658100
H	6.73396400	-1.20940200	0.76442800
H	6.55484900	-2.69662200	-0.18966600
H	4.02498900	0.05852300	0.43214500
H	3.22288500	-1.21262100	-0.64210200
N	5.56413300	-0.95610300	-0.97063100
C	4.16656500	-3.46578100	0.59919800
H	4.81264500	-4.22165100	1.06159800
H	3.15987200	-3.58446700	1.00201000
H	4.13439300	-3.66473900	-0.47770100
C	4.82932100	-1.82806300	2.40736700
H	3.89609300	-2.06115000	2.92514100
H	5.61672700	-2.47392900	2.81554700
H	5.10025900	-0.78754600	2.62469100

(*S*)-**TSB**

B3LYP-D3/BSI SCF energy in $PhCF_3$ -3265.705213 a.u.

M06/BSII SCF energy in $PhCF_3$: -3264.29184 a.u.

M06/BSII free energy in $PhCF_3$: -3263.252938 a.u.

C	3.52552600	0.86340800	-1.56929500
C	4.88733400	1.12224800	-1.94978300
H	5.23446700	2.01465200	-2.45103200
C	5.68957300	0.04296500	-1.49336300
H	6.76573700	-0.02853900	-1.58512200
C	3.49665600	-0.41364400	-0.87678800
C	4.84456900	-0.89569400	-0.83727600
H	5.16324200	-1.81033900	-0.35648700
C	4.20707000	2.69926600	1.10735300
H	3.58134200	3.47257100	0.68081800
C	5.62404100	2.59116600	0.96785100
H	6.26412100	3.27498600	0.42467600
C	6.04039200	1.38956800	1.61991200
H	7.05196500	1.00526600	1.65745400
C	3.74653600	1.56465400	1.84198600
H	2.71487600	1.34092500	2.07477200
C	4.88134000	0.75432000	2.15967300
H	4.85561000	-0.19623500	2.67467800
Fe	4.68509900	0.95460600	0.10597900
P	2.00237000	-1.22942400	-0.23426400
C	1.66064400	-2.60115100	-1.41413600
C	0.46341400	-3.30822700	-1.20575100
C	2.50067200	-2.97398300	-2.47213500
C	0.13055300	-4.39280500	-2.01590700
H	-0.20809400	-2.99987900	-0.40883300
C	2.15645300	-4.05244600	-3.29398200
H	3.42419200	-2.43295600	-2.65562200
C	0.98015100	-4.76949400	-3.06191500
H	-0.79548000	-4.93395600	-1.84015700

H	2.81381900	-4.33556100	-4.11189500
H	0.72023500	-5.61097400	-3.69850500
C	2.61829200	-2.13150400	1.24778100
C	2.36676700	-1.59048800	2.51616400
C	3.35255300	-3.32525100	1.14529800
C	2.86456500	-2.21407700	3.66305600
H	1.78166600	-0.68330900	2.60997100
C	3.85005800	-3.94660700	2.29197800
H	3.53530000	-3.77102400	0.17170900
C	3.61160500	-3.38922800	3.55283400
H	2.65865000	-1.78230100	4.63813700
H	4.41933700	-4.86793000	2.20121500
H	3.99806600	-3.87560700	4.44451000
C	2.45400200	1.84098600	-1.77231500
N	1.28300900	1.90422600	-1.23191600
O	2.78345700	2.82635700	-2.64036000
C	1.72420200	3.81653200	-2.55584000
H	2.12129200	4.67021900	-1.99838100
H	1.47113700	4.12907000	-3.56931800
C	0.58509100	3.07789400	-1.82298100
H	-0.13780700	2.68200300	-2.54667000
C	-0.19835300	3.93466400	-0.79152200
C	-1.50614200	3.20621900	-0.43776800
H	-1.33076200	2.17082000	-0.14063000
H	-2.02546500	3.71792200	0.37977200
H	-2.17604700	3.18440100	-1.30570200
C	-0.57254500	5.28494900	-1.43503600
H	-1.10692400	5.13746600	-2.38226400
H	-1.23313800	5.84791700	-0.76435700

H	0.30426800	5.91207800	-1.63096900
C	0.64198900	4.17574900	0.47385400
H	0.91952200	3.23189400	0.95223700
H	1.56650500	4.72097400	0.24734500
H	0.07516700	4.77567100	1.19603800
Cu	0.35544600	0.27011000	-0.30119100
N	-1.52463900	-0.50905100	0.45431000
C	-1.74377200	-0.81150700	1.71263300
C	-2.77768300	-1.79224600	2.16412300
C	-2.80255700	-3.10703500	1.67015400
C	-3.72844300	-1.41921500	3.13011300
C	-3.76900800	-4.01382500	2.10859500
H	-2.05591100	-3.42228100	0.94694200
C	-4.69700000	-2.32389400	3.56657600
H	-3.70377700	-0.41282000	3.53852500
C	-4.72399900	-3.62358200	3.05207600
H	-3.77174900	-5.02790900	1.71779100
H	-5.42892700	-2.01396600	4.30771200
H	-5.47717500	-4.32974300	3.39082700
C	-0.92710400	-0.16441900	2.76754000
C	-0.39044300	1.12589200	2.58933300
C	-0.66414700	-0.82403400	3.98417700
C	0.40278600	1.72035000	3.56988100
H	-0.62256200	1.67383200	1.68553100
C	0.12720200	-0.22852200	4.96687700
H	-1.06226900	-1.81936300	4.15237100
C	0.67201000	1.04306800	4.76412600
H	0.79896200	2.71879700	3.40494200
H	0.32621400	-0.76544800	5.89052800

H	1.28929700	1.50474900	5.52979200
C	-2.33388500	-0.92986600	-0.59548600
H	-2.96794700	-1.79943400	-0.48695400
C	-1.80932500	-0.67422000	-1.91926100
O	-0.90579900	0.14310300	-2.17047300
O	-2.41247600	-1.41106300	-2.87490200
C	-2.08559700	-1.25571800	-4.30474800
C	-0.61622100	-1.60451000	-4.55962600
H	-0.43607200	-1.63980500	-5.64031600
H	0.04967800	-0.86373300	-4.11574700
H	-0.37924500	-2.58591400	-4.13969800
C	-2.43112000	0.15930200	-4.77636200
H	-2.28801800	0.22632100	-5.86084300
H	-3.47734900	0.39338900	-4.55467600
H	-1.79165600	0.90072200	-4.29375400
C	-3.00297700	-2.28540400	-4.96618600
H	-2.85318400	-2.27892200	-6.05098900
H	-2.78442000	-3.29122200	-4.59119000
H	-4.05452900	-2.05834000	-4.75993300
C	-5.53841900	1.91083000	1.87230100
C	-5.20735700	3.40811100	0.04451200
C	-4.76318900	4.11293000	1.34792000
C	-4.54187400	2.96274400	2.35272000
H	-6.57000800	2.17458200	2.15209100
H	-5.33116100	0.89915100	2.22891300
H	-4.46606400	3.47903200	-0.75720700
H	-6.15507300	3.81159800	-0.33312600
H	-3.86809700	4.72341100	1.20336100
H	-5.56119300	4.77227900	1.70435200

H	-3.52257400	2.56606400	2.27020300
H	-4.71084300	3.26056500	3.39109700
C	-6.28749100	1.15414000	-0.42609200
C	-4.06206700	0.61156900	-0.24997900
C	-5.36510200	-0.05015100	-0.74505400
H	-6.54637000	1.71418300	-1.32876300
H	-7.21319700	0.90103500	0.10423000
H	-3.52578400	1.29378100	-0.89398200
H	-3.68028000	0.44077600	0.74380400
N	-5.38204000	1.98019800	0.40729300
C	-5.75869500	-1.29212200	0.06671200
H	-6.76711600	-1.61253000	-0.22127900
H	-5.07770400	-2.12808400	-0.11552300
H	-5.76263700	-1.09122000	1.14126300
C	-5.36338800	-0.37314900	-2.24163500
H	-4.69910500	-1.20722700	-2.47080200
H	-6.37932100	-0.63765000	-2.56069400
H	-5.04031200	0.49174900	-2.83240100

(*R*)-**TSB**

B3LYP-D3/BSI SCF energy in $PhCF_3$ -3265.700483 a.u.

M06/BSII SCF energy in $PhCF_3$: -3264.291189 a.u.

M06/BSII free energy in $PhCF_3$: -3263.254522 a.u.

C	3.71909400	0.52287700	-1.00694700
C	5.09501800	0.29416200	-1.34476900
H	5.89983200	0.99865000	-1.18779600
C	5.21149300	-1.02081100	-1.87034700

H	6.13337200	-1.50523200	-2.16561900
C	2.97788500	-0.67925800	-1.34266300
C	3.91774200	-1.61647200	-1.87711200
H	3.68355400	-2.62364600	-2.19300000
C	4.35551300	-0.61197200	2.07168200
H	4.04520900	0.35381500	2.44850800
C	5.68385600	-0.97793000	1.69640900
H	6.55800200	-0.34113000	1.74723900
C	5.64459100	-2.31187900	1.18470000
H	6.48513200	-2.86191200	0.78063000
C	3.49552000	-1.71892900	1.79510300
H	2.42532500	-1.73931000	1.93794700
C	4.29221100	-2.76962100	1.24500800
H	3.92647600	-3.72631600	0.89433300
Fe	4.41778300	-1.07831200	0.06124800
P	1.16935300	-0.81971800	-1.22051300
C	0.65156300	-0.52241200	-2.96289800
C	-0.43587600	-1.19352200	-3.53911300
C	1.27114800	0.51157500	-3.68634200
C	-0.88615500	-0.85052400	-4.81687400
H	-0.93031900	-1.99138000	-2.99862700
C	0.82006900	0.85223300	-4.96186600
H	2.10382400	1.05823800	-3.25312300
C	-0.26074100	0.17253000	-5.53258200
H	-1.72728200	-1.38492500	-5.25056300
H	1.31323000	1.65119800	-5.50914700
H	-0.61103100	0.43993800	-6.52572700
C	0.84449500	-2.58716200	-0.86708000
C	0.34781200	-2.89258800	0.41055000

C	1.08211000	-3.62751600	-1.78019800
C	0.11701500	-4.21924200	0.78146200
H	0.14590600	-2.08602500	1.11118200
C	0.83852000	-4.95230900	-1.41209200
H	1.44427200	-3.40291200	-2.77916200
C	0.36198000	-5.24993200	-0.13028600
H	-0.25904300	-4.44374500	1.77571400
H	1.02062600	-5.75233400	-2.12440500
H	0.17690400	-6.28226800	0.15344100
C	3.21107400	1.80533800	-0.50815300
N	2.12428900	2.07022900	0.13869300
O	4.00490700	2.84756400	-0.84262800
C	3.41368800	4.03077100	-0.24423700
H	4.07417600	4.34943900	0.56651400
H	3.37510700	4.81032800	-1.00649000
C	2.02336600	3.55590700	0.23508800
H	1.25110800	3.85545000	-0.48329200
C	1.60632000	4.11308100	1.62293500
C	0.12791500	3.78334900	1.89169300
H	-0.05030100	2.70880900	1.91331200
H	-0.18392900	4.20131800	2.85697600
H	-0.51751900	4.20583000	1.11258500
C	1.74964000	5.64923200	1.58253400
H	1.16313500	6.07905100	0.76039400
H	1.38070500	6.08441300	2.51884000
H	2.79089800	5.97047600	1.46234400
C	2.48565700	3.53579200	2.74487100
H	2.42716000	2.44347900	2.77325500
H	3.54052500	3.81009900	2.62413800

H	2.15667500	3.91678100	3.71894500
Cu	0.42930400	0.75393100	0.14086900
N	-1.38844400	0.79949900	1.20602000
C	-1.71815500	0.32261900	2.38241200
C	-3.11773900	0.47211900	2.91571800
C	-4.04289800	-0.58215500	2.93942800
C	-3.51665300	1.73020400	3.39746200
C	-5.33674000	-0.38441800	3.42984000
H	-3.75152500	-1.56209100	2.57429400
C	-4.80986800	1.92971900	3.88237000
H	-2.80809700	2.55449600	3.38041500
C	-5.72455400	0.87216600	3.89995200
H	-6.03977900	-1.21325800	3.44194200
H	-5.10291000	2.91038700	4.24774500
H	-6.73146000	1.02683200	4.27794000
C	-0.70838500	-0.26758600	3.29483500
C	0.63303600	0.16076300	3.29976200
C	-1.08437200	-1.25122800	4.23418200
C	1.55237800	-0.36183100	4.20851500
H	0.95276600	0.93212000	2.61095300
C	-0.15457800	-1.79820300	5.11775900
H	-2.11198300	-1.59298200	4.27940000
C	1.16947200	-1.35260300	5.11591900
H	2.57150700	0.01080600	4.21040600
H	-0.47227600	-2.56478200	5.81967100
H	1.89000000	-1.76167600	5.81875800
C	-2.36022600	1.30559400	0.34165500
H	-3.33309600	1.56143300	0.73914600
C	-1.89020500	2.10238000	-0.76471700

O	-0.70746400	2.16400800	-1.14611200
O	-2.89043300	2.77966700	-1.37319500
C	-2.63450000	3.76050900	-2.44442000
C	-2.00848400	3.09107500	-3.67170300
H	-1.91147300	3.83197100	-4.47378000
H	-1.02237900	2.68561000	-3.44505700
H	-2.64429500	2.27971100	-4.03913200
C	-1.75895900	4.89430400	-1.90096600
H	-1.69246200	5.69512400	-2.64593400
H	-2.19952300	5.31205700	-0.98860800
H	-0.75030600	4.54168900	-1.67718000
C	-4.03998000	4.27000800	-2.76629200
H	-3.99145700	5.03525800	-3.54834500
H	-4.67567100	3.45169700	-3.12160900
H	-4.50550100	4.70915600	-1.87732200
C	-2.82522600	-3.53095500	-1.77572400
C	-3.50048400	-3.54563500	0.47641200
C	-3.46704500	-5.02524600	0.00487000
C	-3.37831400	-4.94075600	-1.53635600
H	-3.01546500	-3.13023300	-2.77257800
H	-1.74793800	-3.50523300	-1.58211300
H	-2.60268400	-3.29708200	1.05311200
H	-4.37244900	-3.29888200	1.08469900
H	-2.58788800	-5.52859100	0.41781300
H	-4.35343500	-5.57516800	0.33288000
H	-2.73961700	-5.71432600	-1.97294700
H	-4.37379800	-5.02892000	-1.98657800
C	-4.77088300	-2.04871100	-1.14636000
C	-2.98172400	-0.77511300	-0.48473800

C	-4.29211900	-0.58323300	-1.27181900
H	-5.50319400	-2.16766400	-0.34360600
H	-5.18971600	-2.47638400	-2.06380400
H	-3.00465300	-0.92177100	0.58060900
H	-2.01630300	-0.80226500	-0.97327400
N	-3.49003400	-2.70187300	-0.74582000
C	-4.00246800	-0.20009500	-2.72742700
H	-4.93734700	-0.14480700	-3.29778400
H	-3.51541600	0.77621100	-2.76975700
H	-3.34721600	-0.92670500	-3.21713100
C	-5.28952800	0.40071200	-0.64896500
H	-4.97582200	1.43621300	-0.80189200
H	-6.27042200	0.27047100	-1.12368400
H	-5.40761100	0.22334000	0.42578200

IX. Product Structure Determination

The structures of products **2b**, **S4** and **10** were determined by X-ray crystallography. The X-ray data have been deposited at the Cambridge Crystallographic Data Center (CCDC 2390026 for **2b**, CCDC 2390027 for **S4**, and CCDC 2293901 for **10**). The structures of other products were assumed by analogy.

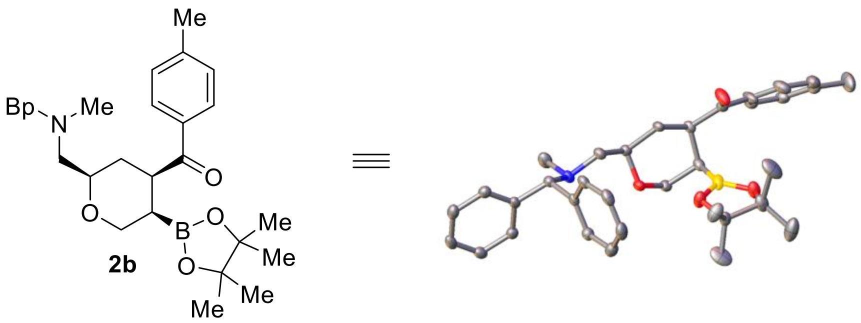

Table S3. Crystal Data and Structure Refinement for 2b

Identification code	**2b**
Empirical formula	$C_{34}H_{42}BNO_4$
Formula weight	539.49
Temperature/K	100.00
Crystal system	monoclinic
Space group	C2
a/Å	38.3788(14)
b/Å	6.1418(2)
c/Å	13.0636(5)
α/°	90
β/°	101.0230(10)
γ/°	90
Volume/Å^3	3022.47(19)
Z	4

ρ_{calc}g/cm^3	1.186
μ/mm^{-1}	0.381
F(000)	1160.0
Crystal size/mm^3	0.15 × 0.14 × 0.12
Radiation	GaKα (λ = 1.34139)
2Θ range for data collection/	4.082 to 114.038
Index ranges	$-47 \leqslant h \leqslant 47, -7 \leqslant k \leqslant 7, -16 \leqslant l \leqslant 16$
Reflections collected	29564
Independent reflections	6179 [R_{int} = 0.0565, R_{sigma} = 0.0343]
Data/restraints/parameters	6179/79/479
Goodness-of-fit on F^2	1.037
Final R indexes [I>=2σ (I)]	R_1 = 0.0355, wR_2 = 0.0942
Final R indexes [all data]	R_1 = 0.0365, wR_2 = 0.0950
Largest diff. peak/hole / e Å^{-3}	0.20/-0.17
Flack parameter	-0.11(13)

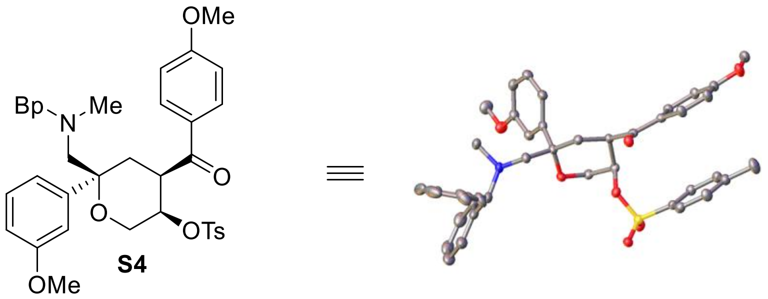

Table S4. Crystal Data and Structure Refinement for S4

Identification code	**S4**
Empirical formula	$C_{42}H_{43}NO_7S$
Formula weight	705.83
Temperature/K	99.98(10)
Crystal system	monoclinic

Space group	$P2_1$
a/Å	7.72764(14)
b/Å	11.1793(2)
c/Å	20.9268(4)
α/°	90
β/°	91.3429(18)
γ/°	90
Volume/Å^3	1807.36(6)
Z	2
ρ_{calc}g/cm^3	1.297
μ/mm^{-1}	1.226
F(000)	748.0
Crystal size/mm^3	0.241 × 0.158 × 0.019
Radiation	CuKα (λ = 1.54184)
2Θ range for data collection/°	8.452 to 150.032
Index ranges	-9 ≤ h ≤ 4, -13 ≤ k ≤ 13, -23 ≤ l ≤ 25
Reflections collected	11011
Independent reflections	6961 [R_{int} = 0.0575, R_{sigma} = 0.0757]
Data/restraints/parameters	6961/1/464
Goodness-of-fit on F^2	1.053
Final R indexes [I>=2σ (I)]	R_1 = 0.0655, wR_2 = 0.1567
Final R indexes [all data]	R_1 = 0.0707, wR_2 = 0.1636
Largest diff. peak/hole / e Å^{-3}	0.76/-0.67
Flack parameter	-0.08(2)

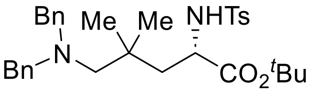

10

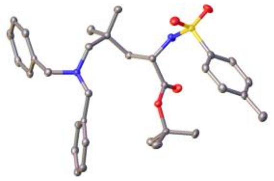

Table S5. Crystal Data and Structure Refinement for 10

Identification code	**10**
Empirical formula	$C_{32}H_{41}N_2O_4S$
Formula weight	549.73
Temperature/K	100.01(10)
Crystal system	monoclinic
Space group	$P2_1$
a/Å	13.0942(2)
b/Å	5.69870(10)
c/Å	19.9924(2)
α/°	90
β/°	99.9420(10)
γ/°	90
Volume/Å^3	1469.43(4)
Z	2
ρ_{calc}g/cm^3	1.242
μ/mm^{-1}	1.284
F(000)	590.0
Crystal size/mm^3	0.16 × 0.15 × 0.13
Radiation	Cu Kα (λ = 1.54184)
2Θ range for data collection/°	4.488 to 153.786
Index ranges	-16 ≤ h ≤ 15, -4 ≤ k ≤ 7, -24 ≤ l ≤ 25
Reflections collected	8905

Independent reflections	4531 [R_{int} = 0.0238, R_{sigma} = 0.0359]
Data/restraints/parameters	4531/1/358
Goodness-of-fit on F^2	1.049
Final R indexes [I>=2σ (I)]	R_1 = 0.0298, wR_2 = 0.0735
Final R indexes [all data]	R_1 = 0.0320, wR_2 = 0.0754
Largest diff. peak/hole / e $Å^{-3}$	0.49/-0.29
Flack parameter	0.048(12)

X. References

(1) Xia, J.; Hirai, T.; Katayama, S.; Nagae, H.; Zhang, W.; Mashima, K. Mechanistic Study of Ni and Cu Dual Catalyst for Asymmetric C-C Bond Formation; Asymmetric Coupling of 1,3-Dienes with C-nucleophiles to Construct Vicinal Stereocenters. *ACS Catal*. **2021**, *11*, 6643–6655.

(2) Roagna, G.; Ascough, D.; F. Ibba, H M.; Vicini, A. C.; Fontana, A.; Christensen, K. E.; Peschiulli, A.; Oehlrich, D.; Misale, A.; Trabanco, A. A.; Paton, R. S.; Pupo, G.; Gouverneur, V. Hydrogen Bonding Phase-Transfer Catalysis with Ionic Reactants: Enantioselective Synthesis of γ-Fluoroamines. *J. Am. Chem. Soc*. **2020**, *142*, 14045–14051.

(3) Ramesh, S.; Balakumar, R.; Rizzo, J. R.; Zhang, T. Y. Facile Synthesis of 2-Azaspiro[3.4]octane. *Org. Biomol. Chem*. **2019**, *17*, 3056–3065.

(4) To minimize product decomposition, the silica gel used for purification was pretreated with a solution of triethylamine in *n*-hexane (1 vol%).

(5) Marenich, A. V.; Cramer, C. J.; Truhlar, D. G. Universal Solvation Model Based on Solute Electron Density and on a Continuum Model of the Solvent Defined by the Bulk Dielectric Constant and Atomic Surface Tensions. *J. Phys. Chem. B*. **2009**, *113*, 6378–6396.

(6) (a) Noodleman, L.; Lovell, T.; Han, W.-G.; Li, J.; Himo, F. Quantum Chemical Studies of Intermediates and Reaction Pathways in Selected Enzymes and Catalytic Synthetic Systems. *Chem. Rev*. **2004**, *104*, 459–508; (b) Noodleman, L. Valence Bond Description of Antiferromagnetic Coupling in Transition Metal Dimers. *J. Chem. Phys*. **1981**, *74*, 5737–5743; (c) Noodleman, L.; Case, D. A. Density-Functional Theory of Spin Polarization and Spin Coupling in Iron—Sulfur Clusters. *Adv. Inorg. Chem*. **1992**, *38*, 423–458.

(7) Grimme, S.; Antony, J.; Ehrlich, S.; Krieg, H. A Consistent and Accurate ab initio Parametrization of Density Functional Dispersion Correction

(DFT-D) for the 94 Elements H-Pu. *J. Chem. Phys*. **2010**, *132*, 154104.

(8) (a) Dolg, M.; Wedig, U.; Stoll, H.; Preuss, H. Energy-adjusted ab initio Pseudopotentials for the First Row Transition Elements. *J. Chem. Phys*. **1987**, *86*, 866–872; (b) Roy, L. E.; Hay, P. J.; Martin, R. L. Revised Basis Sets for the LANL Effective Core Potentials. *J. Chem. Theory. Comput*. **2008**, *4*, 1029–1031.

(9) (a) Zhao, Y.; Truhlar, D. G. Benchmark Energetic Data in a Model System for Grubbs II Metathesis Catalysis and Their Use for the Development, Assessment, and Validation of Electronic Structure Methods. *J. Chem. Theory Comput*. **2009**, *5*, 324–333; (b) Zhao, Y.; Truhlar, D. G. The M06 Suite of Density Functionals for Main Group Thermochemistry, Thermochemical Kinetics, Noncovalent Interactions, Excited States, and Transition Elements: Two New Functionals and Systematic Testing of Four M06-class Functionals and 12 other Functionals. *Theor. Chem. Acc*. **2008**, *120*, 215–241; (c) Zhao, Y.; Truhlar, D. G. Density Functionals with Broad Applicability in Chemistry. *Acc. Chem. Res*. **2008**, *41*, 157–167.

(10) Fukui, K. The Path of Chemical Reactions —The IRC Approach. *Acc. Chem. Res*. **1981**, *14*, 363–368.

(11) (a) Lu, T.; Chen, F. Multiwfn: A Multifunctional Wavefunction Analyzer. *J. Comput. Chem.* **2012**, *33*, 580−592. (b) Johnson, E. R.; Keinan, S.; Mori-Sanchez, P.; ContrerasGarcia, J.; Cohen, A. J.; Yang, W. Revealing Noncovalent Interactions. *J. Am. Chem. Soc.* **2010**, *132*, 6498−6506.

(12) Humphrey, W.; Dalke, A.; Schulten, K. VMD: Visual Molecular Dynamics. *J. Mol. Graphics.* **1996**, *14*, 33−38.

(13) Frisch, M. J.; Trucks, G. W.; Schlegel, H. B.; Scuseria, G. E.; Robb, M. A.; Cheeseman, J. R.; Scalmani, G.; Barone, V.; Mennucci, B.; Petersson, G. A.; Nakatsuji, H.; Caricato, M.; Li, X.; Hratchian, H. P.; Izmaylov, A. F.; Bloino, J.; Zheng, G.; Sonnenberg, J. L.; Hada, M.; Ehara, M.; Toyota, K.; Fukuda, R.; Hasegawa, J.; Ishida, M.; Nakajima, T.; Honda, Y.; Kitao, O.; Nakai, H.;

Vreven, T.; Montgomery, J. A., Jr.; Peralta, J. E.; Ogliaro, F.; Bearpark, M.; Heyd, J. J.; Brothers, E.; Kudin, K. N.; Staroverov, V. N.; Keith, T.; Kobayashi, R.; Normand, J.; Raghavachari, K.; Rendell, A.; Burant, J. C.; Iyengar, S. S.; Tomasi, J.; Cossi, M.; Rega, N.; Millam, J. M.; Klene, M.; Knox, J. E.; Cross, J. B.; Bakken, V.; Adamo, C.; Jaramillo, J.; Gomperts, R.; Stratmann, R. E.; Yazyev, O.; Austin, A. J.; Cammi, R.; Pomelli, C.; Ochterski, J. W.; Martin, R. L.; Morokuma, K.; Zakrzewski, V. G.; Voth, G. A.; Salvador, P.; Dannenberg, J. J.; Dapprich, S.; Daniels, A. D.; Farkas, O.; Foresman, J. B.; Ortiz, J. V.; Cioslowski, J.; Fox, D. J. Gaussian 09, Rev. A.01; Gaussian, Inc.: Wallingford, CT, 2010.

(14) CYLview20; Legault, C. Y. Université de Sherbrooke, 2020 (http://www.cylview.org).

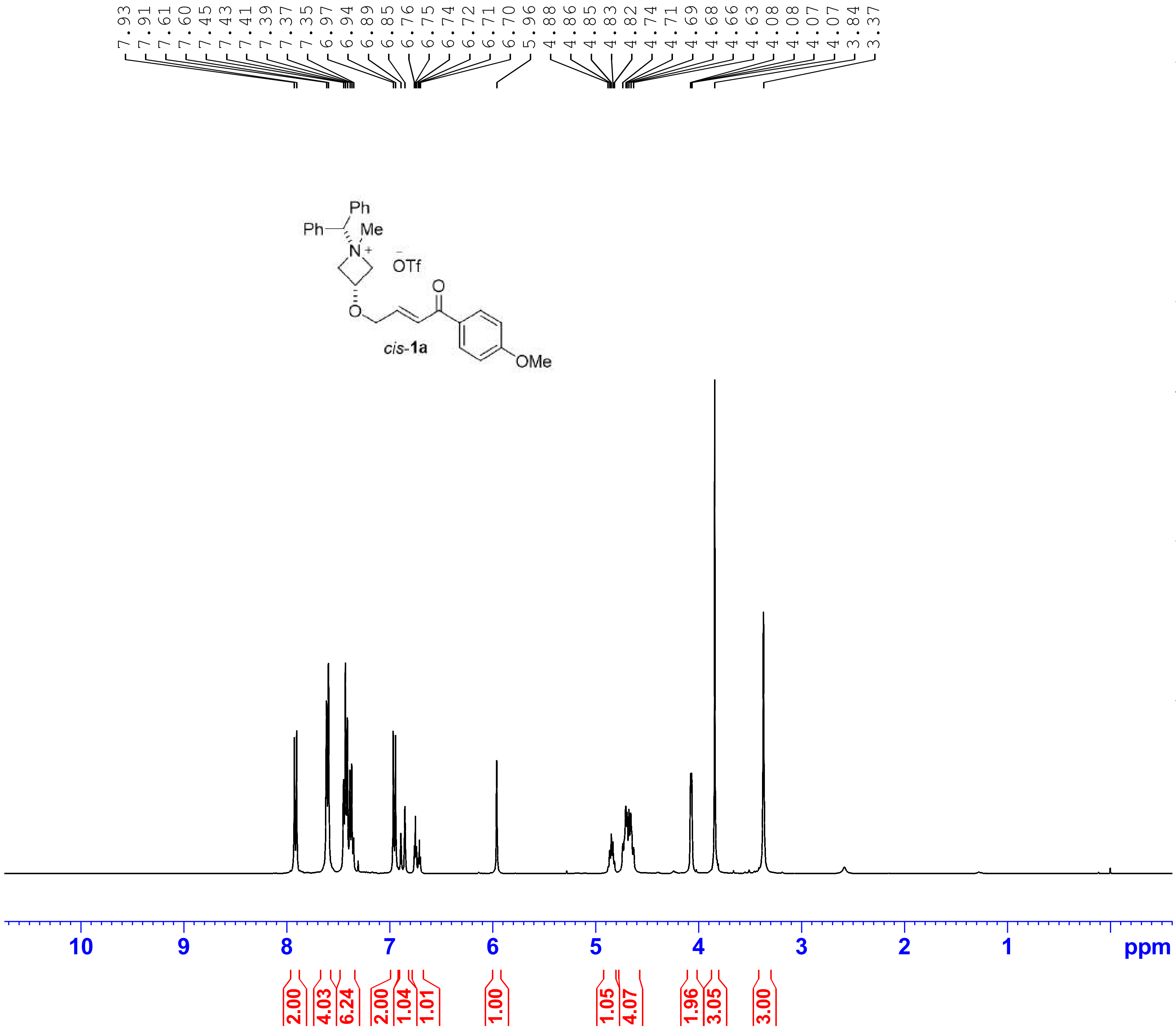

Current Data Parameters
NAME zmh-sm-180j-xia
EXPNO 22
PROCNO 1

F2 - Acquisition Parameters
Date_ 20240607
Time_ 0.41 h
INSTRUM AvanceNeo 400MHz
PROBHD Z163739_0629 (
PULPROG zg30
TD 65536
SOLVENT CDCl3
NS 8
DS 2
SWH 8196.722 Hz
FIDRES 0.250144 Hz
AQ 3.9976959 sec
RG 32
DW 61.000 usec
DE 13.89 usec
TE 297.1 K
D1 1.00000000 sec
TD0 1
SFO1 400.1824711 MHz
NUC1 1H
P0 2.67 usec
P1 8.00 usec
PLW1 21.26700020 W

F2 - Processing parameters
SI 65536
SF 400.1799900 MHz
WDW EM
SSB 0
LB 0.30 Hz
GB 0
PC 1.00

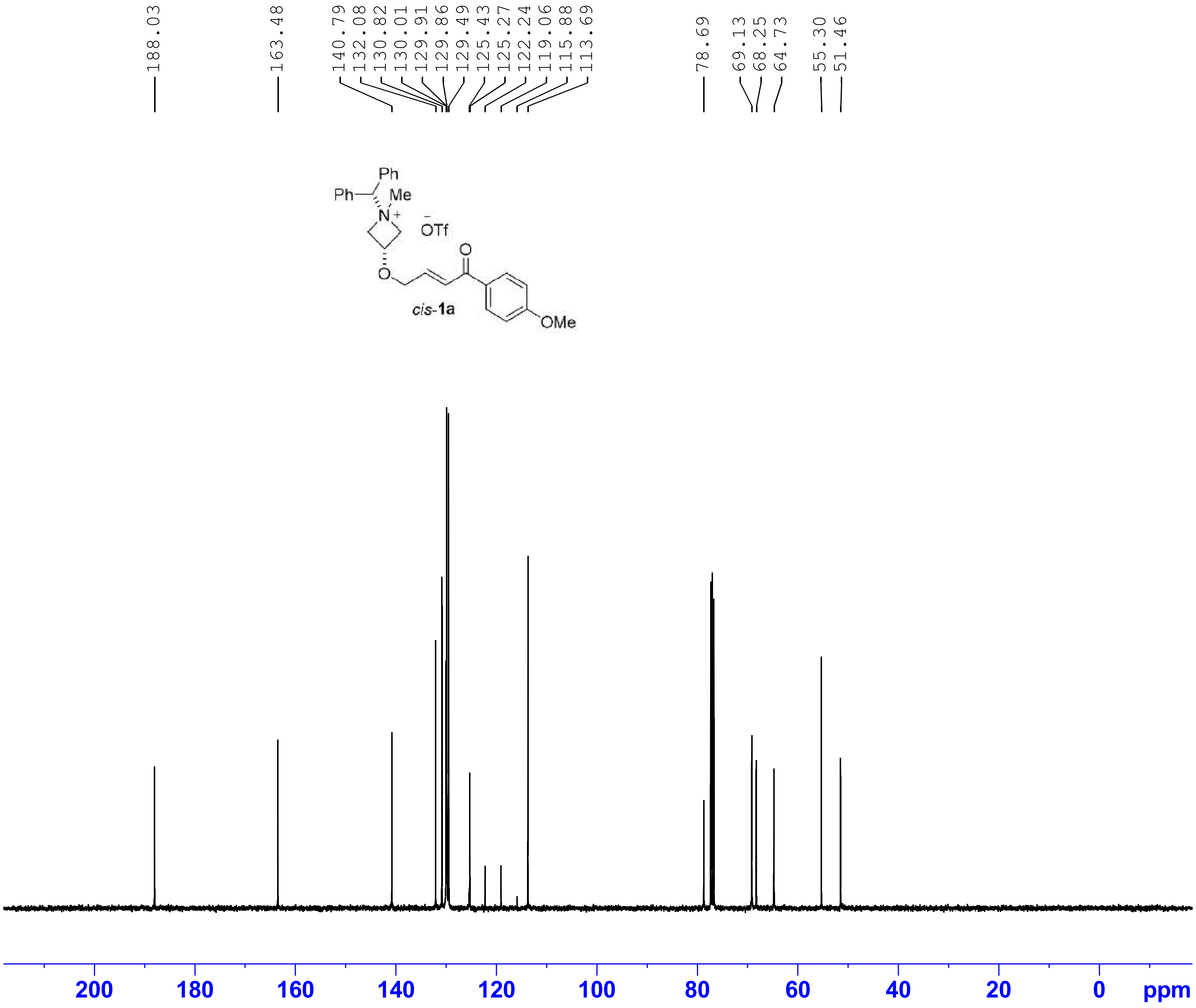

cis-**1a**

Current Data Parameters	
NAME	zmh-sm-180j-xia
EXPNO	23
PROCNO	1
F2 - Acquisition Parameters	
Date_	20240607
Time	0.54 h
INSTRUM	AvanceNeo 400MHz
PROBHD	Z163739_0629 (
PULPROG	zgpg30
TD	65536
SOLVENT	CDCl3
NS	200
DS	4
SWH	23809.523 Hz
FIDRES	0.726609 Hz
AQ	1.3762560 sec
RG	22.6
DW	21.000 usec
DE	6.50 usec
TE	297.9 K
D1	2.00000000 sec
D11	0.03000000 sec
TD0	1
SFO1	100.6354036 MHz
NUC1	13C
P0	2.67 usec
P1	8.00 usec
PLW1	85.25399780 W
SFO2	400.1816007 MHz
NUC2	1H
CPDPRG[2	waltz65
PCPD2	90.00 usec
PLW2	21.26700020 W
PLW12	0.16802999 W
PLW13	0.08452000 W
F2 - Processing parameters	
SI	32768
SF	100.6253643 MHz
WDW	EM
SSB	0
LB	1.00 Hz
GB	0
PC	1.40

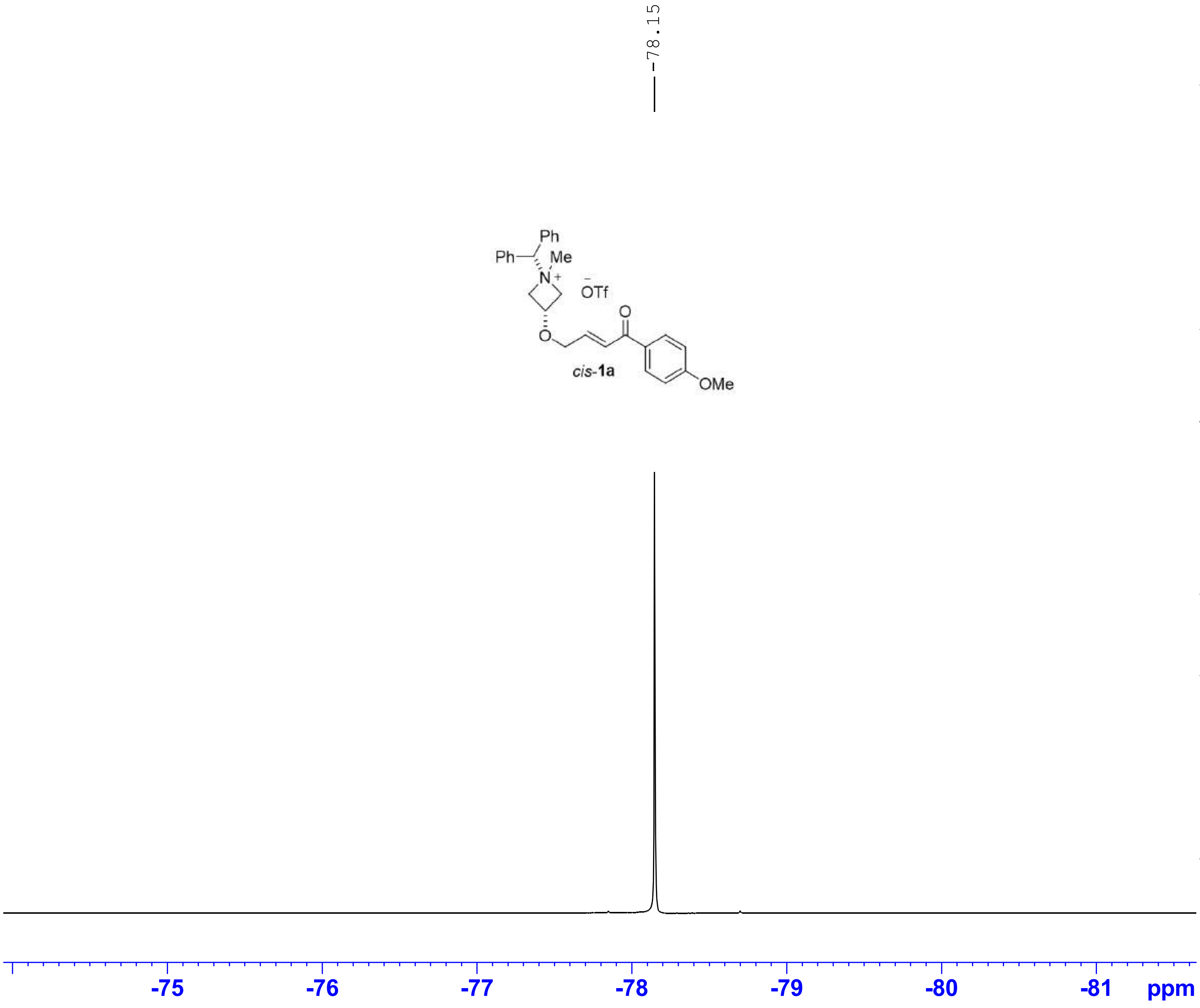

Current Data Parameters

NAME	zmh-sm-180j-xia
EXPNO	24
PROCNO	1

F2 - Acquisition Parameters

Date_	20240607
Time	0.57 h
INSTRUM	AvanceNeo 400MHz
PROBHD	Z163739_0629 (
PULPROG	zgig
TD	131072
SOLVENT	CDCl3
NS	8
DS	4
SWH	90909.094 Hz
FIDRES	1.387163 Hz
AQ	0.7208960 sec
RG	101
DW	5.500 usec
DE	6.50 usec
TE	297.5 K
D1	1.00000000 sec
D11	0.03000000 sec
TD0	1
SFO1	376.5077587 MHz
NUC1	19F
P1	12.00 usec
PLW1	33.72800064 W
SFO2	400.1816007 MHz
NUC2	1H
CPDPRG[2	waltz16
PCPD2	90.00 usec
PLW2	21.26700020 W
PLW12	0.16802999 W

F2 - Processing parameters

SI	65536
SF	376.5454132 MHz
WDW	EM
SSB	0
LB	0.30 Hz
GB	0
PC	1.00

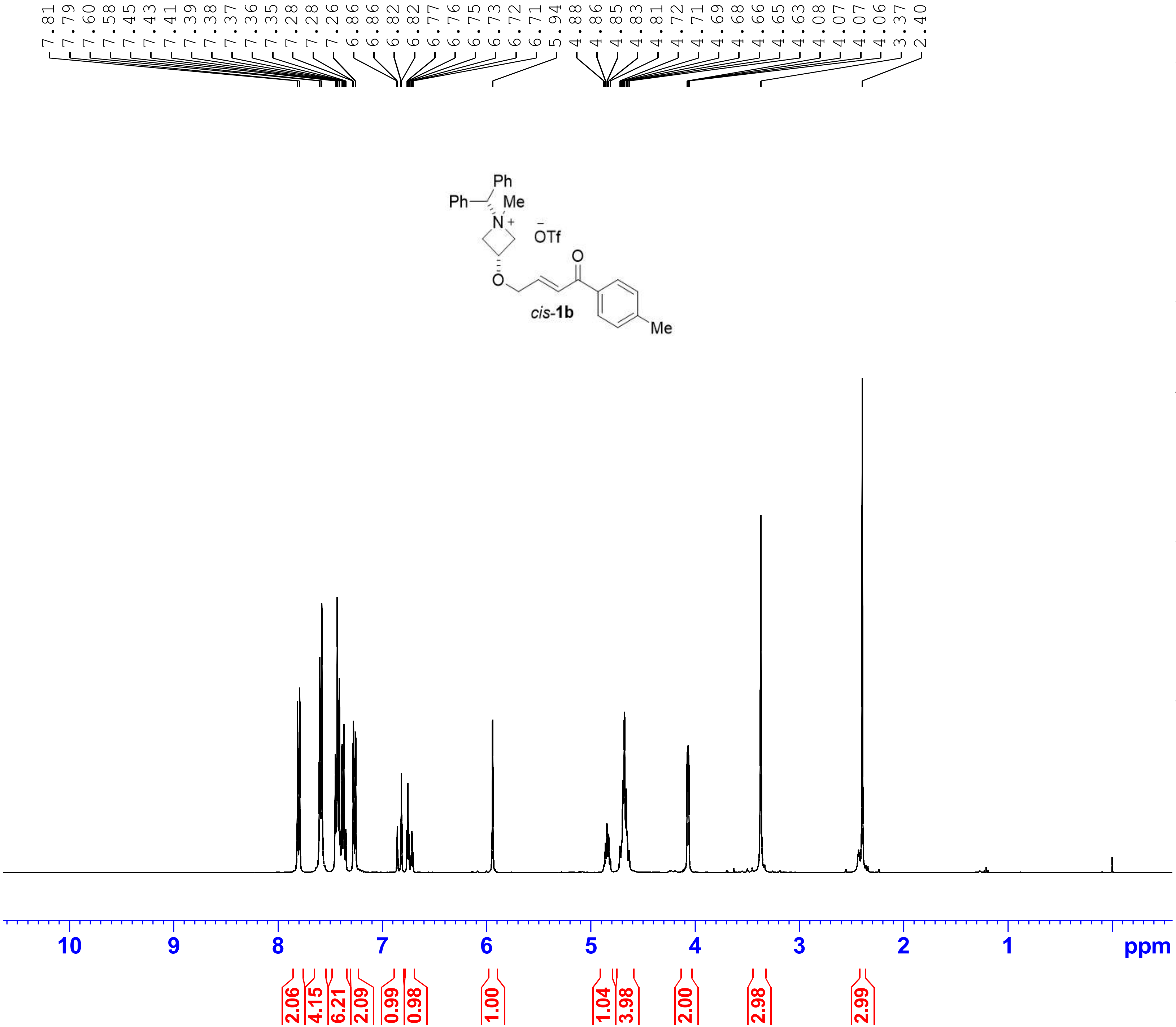

Current Data Parameters
NAME zmh-sm-180a-xia
EXPNO 10
PROCNO 1

F2 - Acquisition Parameters
Date_ 20240606
Time 23.38 h
INSTRUM AvanceNeo 400MHz
PROBHD Z163739_0629 (
PULPROG zg30
TD 65536
SOLVENT CDCl3
NS 8
DS 2
SWH 8196.722 Hz
FIDRES 0.250144 Hz
AQ 3.9976959 sec
RG 36
DW 61.000 usec
DE 13.89 usec
TE 297.0 K
D1 1.00000000 sec
TD0 1
SFO1 400.1824711 MHz
NUC1 1H
P0 2.67 usec
P1 8.00 usec
PLW1 21.26700020 W

F2 - Processing parameters
SI 65536
SF 400.1799996 MHz
WDW EM
SSB 0
LB 0.30 Hz
GB 0
PC 1.00

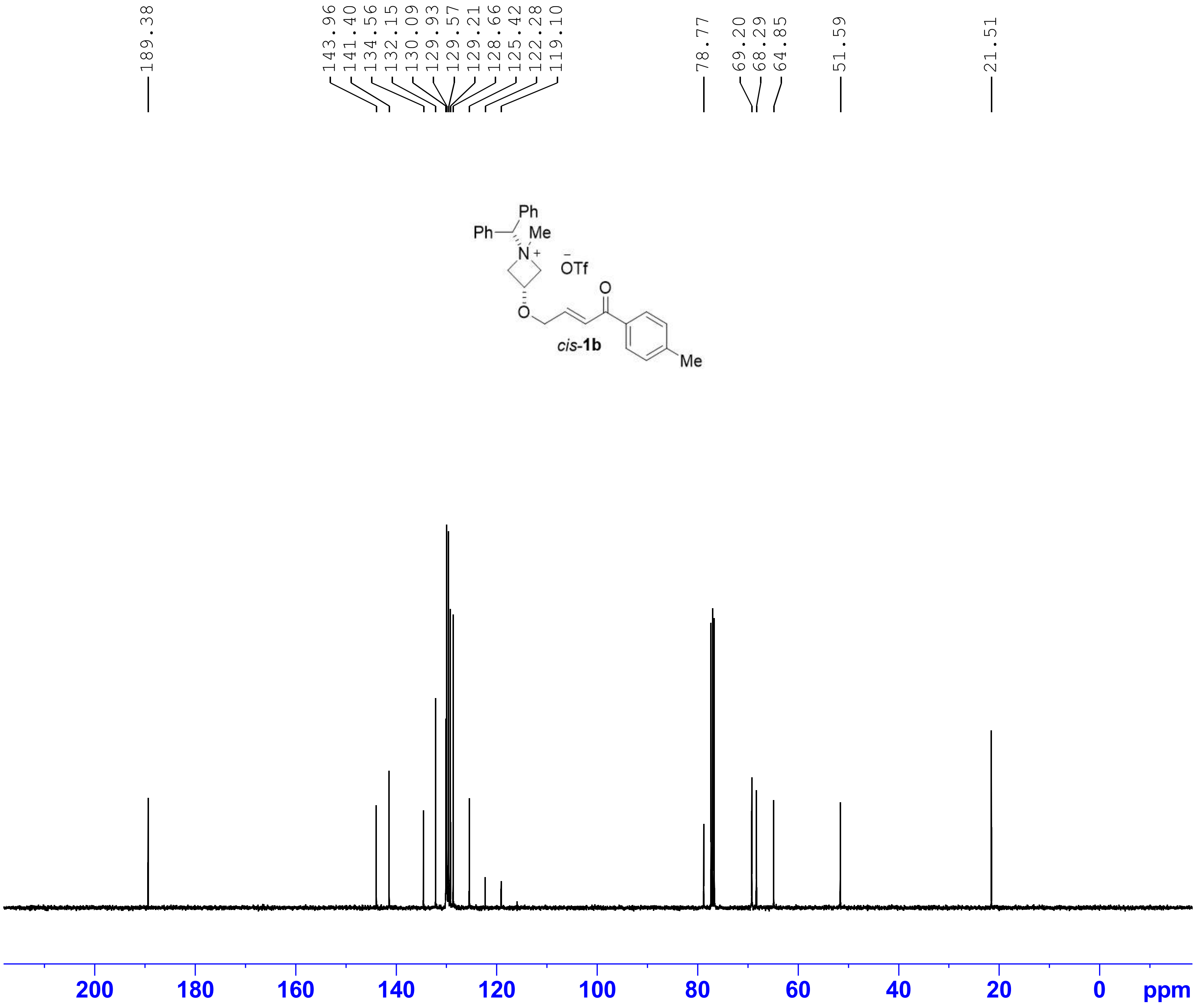

Current Data Parameters
NAME zmh-sm-180a-xia
EXPNO 11
PROCNO 1

F2 - Acquisition Parameters
Date_ 20240606
Time 23.52 h
INSTRUM AvanceNeo 400MHz
PROBHD Z163739_0629 (
PULPROG zgpg30
TD 65536
SOLVENT CDCl3
NS 200
DS 4
SWH 23809.523 Hz
FIDRES 0.726609 Hz
AQ 1.3762560 sec
RG 22.6
DW 21.000 usec
DE 6.50 usec
TE 297.8 K
D1 2.00000000 sec
D11 0.03000000 sec
TD0 1
SFO1 100.6354036 MHz
NUC1 13C
P0 2.67 usec
P1 8.00 usec
PLW1 85.25399780 W
SFO2 400.1816007 MHz
NUC2 1H
CPDPRG[2 waltz65
PCPD2 90.00 usec
PLW2 21.26700020 W
PLW12 0.16802999 W
PLW13 0.08452000 W

F2 - Processing parameters
SI 32768
SF 100.6253577 MHz
WDW EM
SSB 0
LB 1.00 Hz
GB 0
PC 1.40

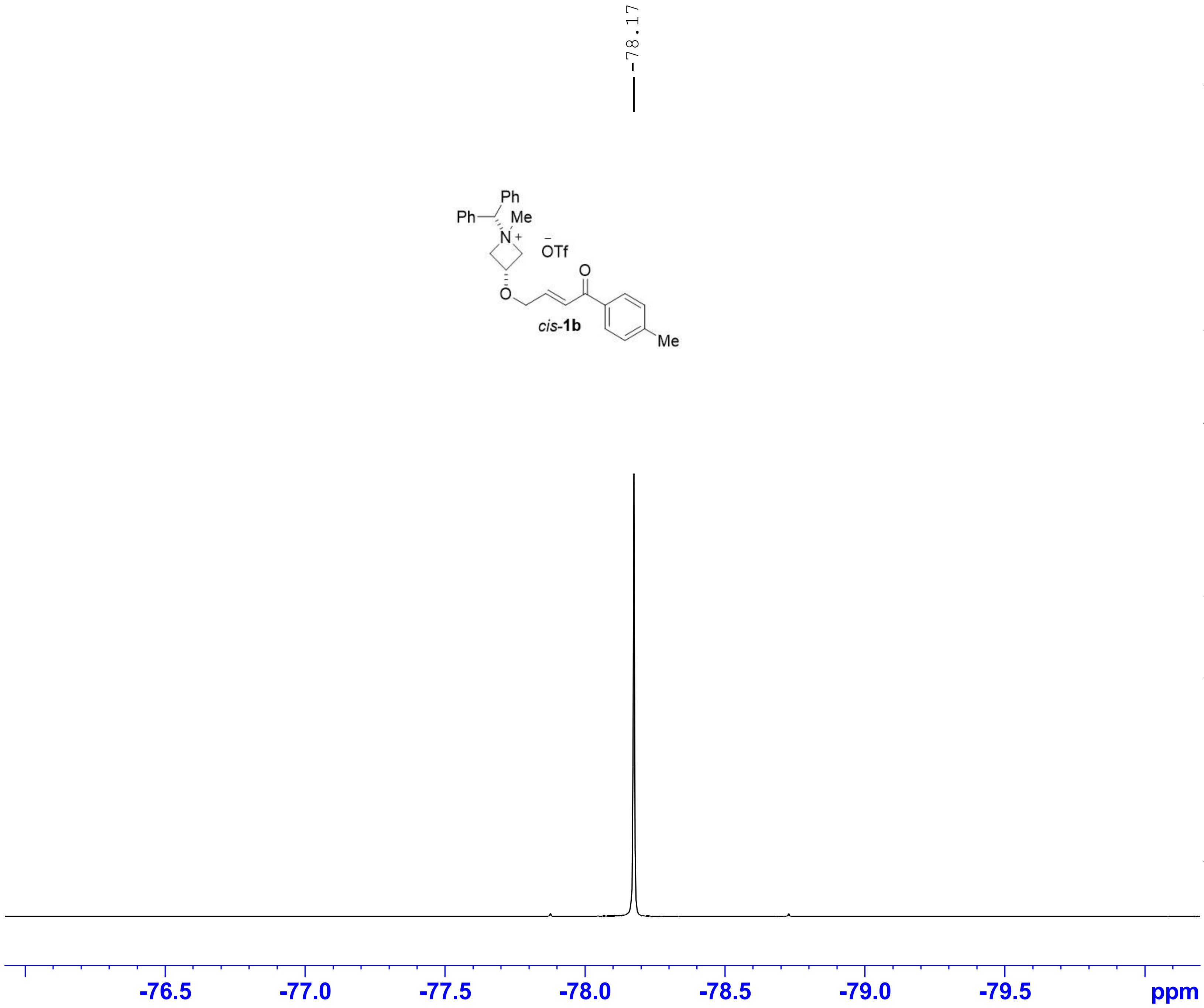

Current Data Parameters	
NAME	zmh-sm-180a-xia
EXPNO	12
PROCNO	1
F2 - Acquisition Parameters	
Date_	20240606
Time	23.54 h
INSTRUM	AvanceNeo 400MHz
PROBHD	Z163739_0629 (
PULPROG	zgig
TD	131072
SOLVENT	CDCl3
NS	8
DS	4
SWH	90909.094 Hz
FIDRES	1.387163 Hz
AQ	0.7208960 sec
RG	101
DW	5.500 usec
DE	6.50 usec
TE	297.4 K
D1	1.00000000 sec
D11	0.03000000 sec
TD0	1
SFO1	376.5077587 MHz
NUC1	19F
P1	12.00 usec
PLW1	33.72800064 W
SFO2	400.1816007 MHz
NUC2	1H
CPDPRG[2	waltz16
PCPD2	90.00 usec
PLW2	21.26700020 W
PLW12	0.16802999 W
F2 - Processing parameters	
SI	65536
SF	376.5454132 MHz
WDW	EM
SSB	0
LB	0.30 Hz
GB	0
PC	1.00

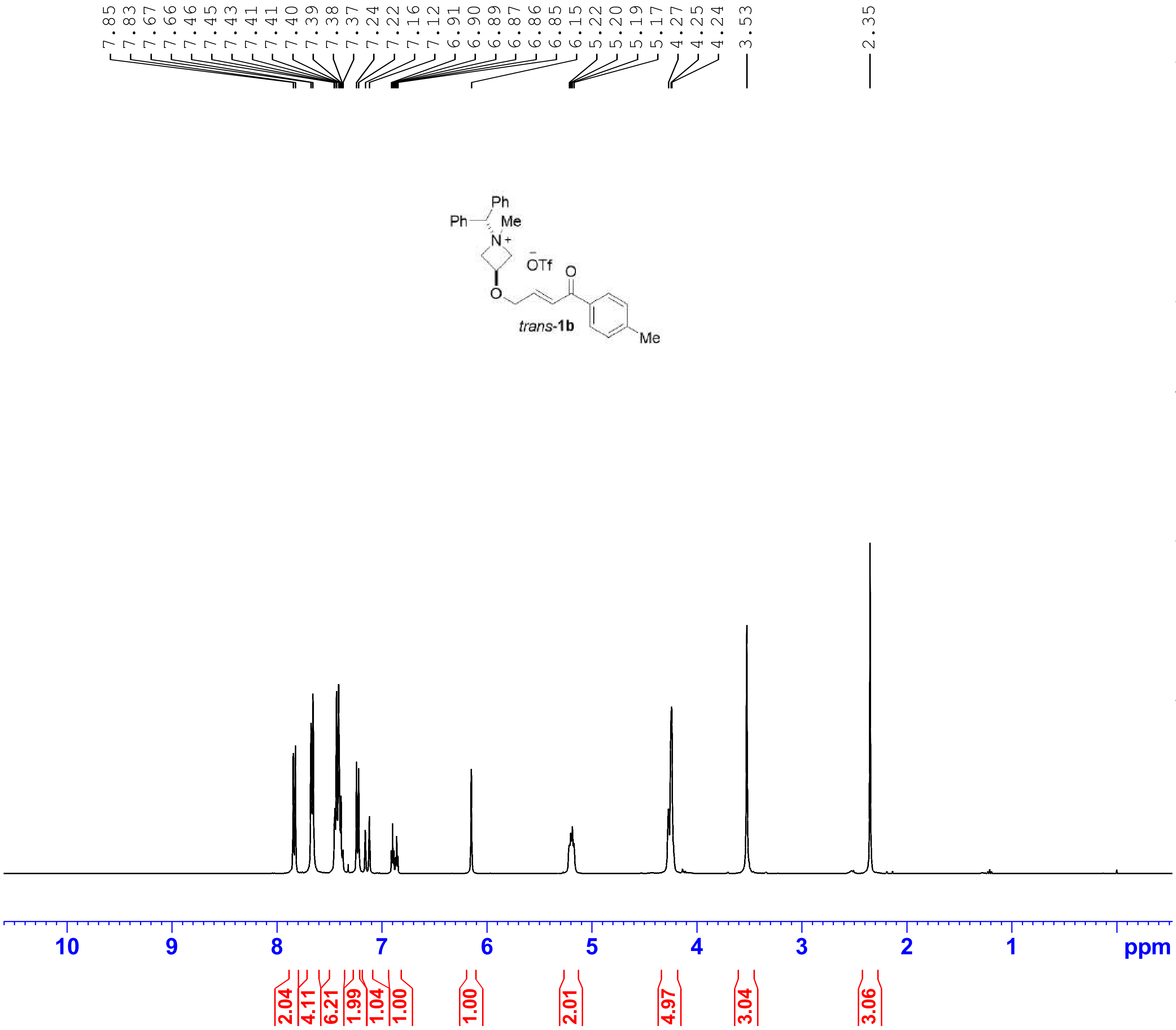


```
Current Data Parameters
NAME     zmh-sm-180a-shang
EXPNO                    7
PROCNO                   1

F2 - Acquisition Parameters
Date_             20240606
Time                23.16 h
INSTRUM   AvanceNeo 400MHz
PROBHD    Z163739_0629 (
PULPROG               zg30
TD                   65536
SOLVENT              CDCl3
NS                       8
DS                       2
SWH               8196.722 Hz
FIDRES            0.250144 Hz
AQ               3.9976959 sec
RG                    22.6
DW                  61.000 usec
DE                   13.89 usec
TE                   297.0 K
D1              1.00000000 sec
TD0                      1
SFO1           400.1824711 MHz
NUC1                    1H
P0                    2.67 usec
P1                    8.00 usec
PLW1           21.26700020 W

F2 - Processing parameters
SI                   65536
SF             400.1799839 MHz
WDW                     EM
SSB                      0
LB                    0.30 Hz
GB                       0
PC                    1.00
```

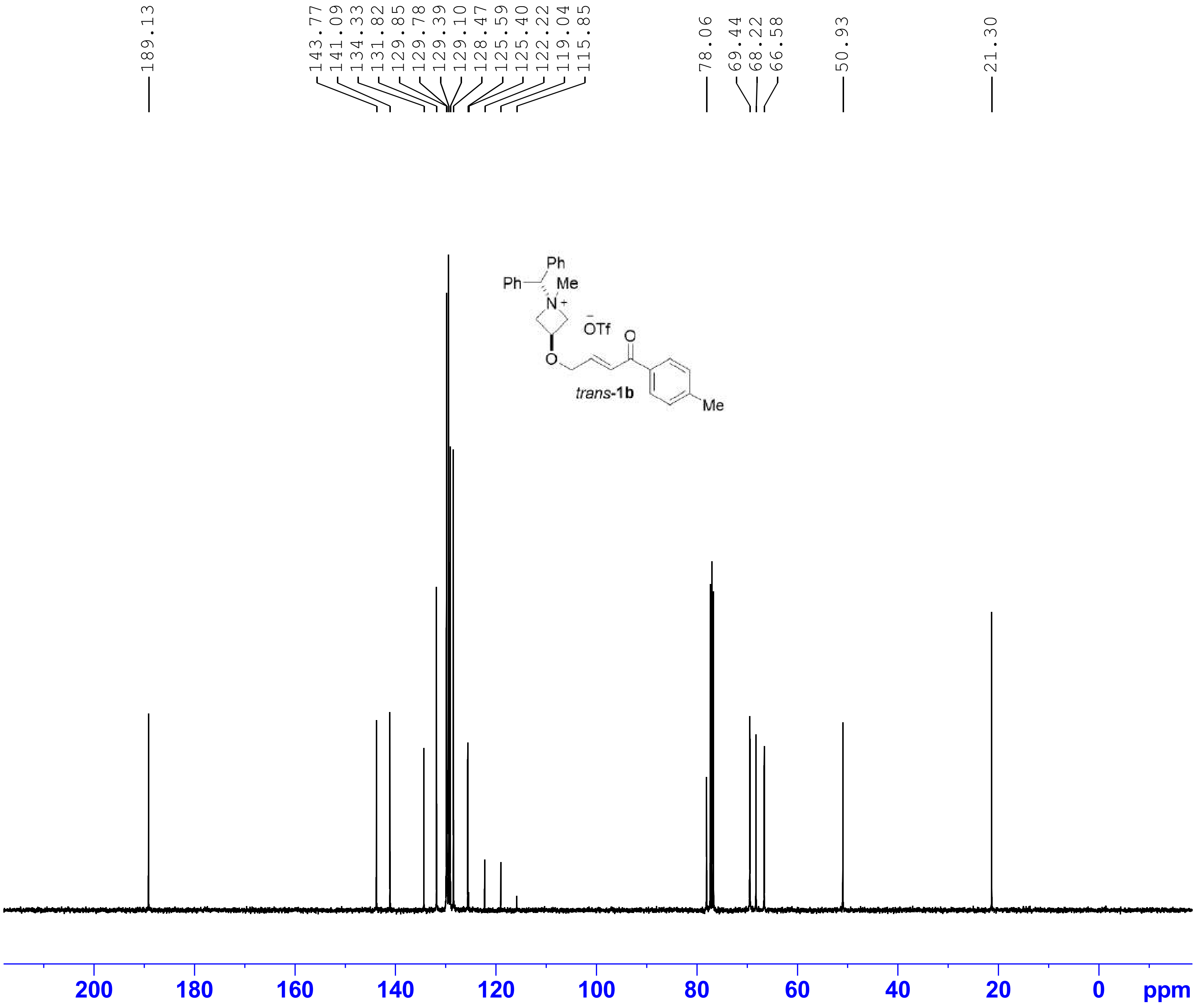

Current Data Parameters
NAME zmh-sm-180a-shang
EXPNO 8
PROCNO 1

F2 - Acquisition Parameters
Date_ 20240606
Time 23.30 h
INSTRUM AvanceNeo 400MHz
PROBHD Z163739_0629 (
PULPROG zgpg30
TD 65536
SOLVENT CDCl3
NS 200
DS 4
SWH 23809.523 Hz
FIDRES 0.726609 Hz
AQ 1.3762560 sec
RG 10
DW 21.000 usec
DE 6.50 usec
TE 297.7 K
D1 2.00000000 sec
D11 0.03000000 sec
TD0 1
SFO1 100.6354036 MHz
NUC1 13C
P0 2.67 usec
P1 8.00 usec
PLW1 85.25399780 W
SFO2 400.1816007 MHz
NUC2 1H
CPDPRG[2 waltz65
PCPD2 90.00 usec
PLW2 21.26700020 W
PLW12 0.16802999 W
PLW13 0.08452000 W

F2 - Processing parameters
SI 32768
SF 100.6253724 MHz
WDW EM
SSB 0
LB 1.00 Hz
GB 0
PC 1.40

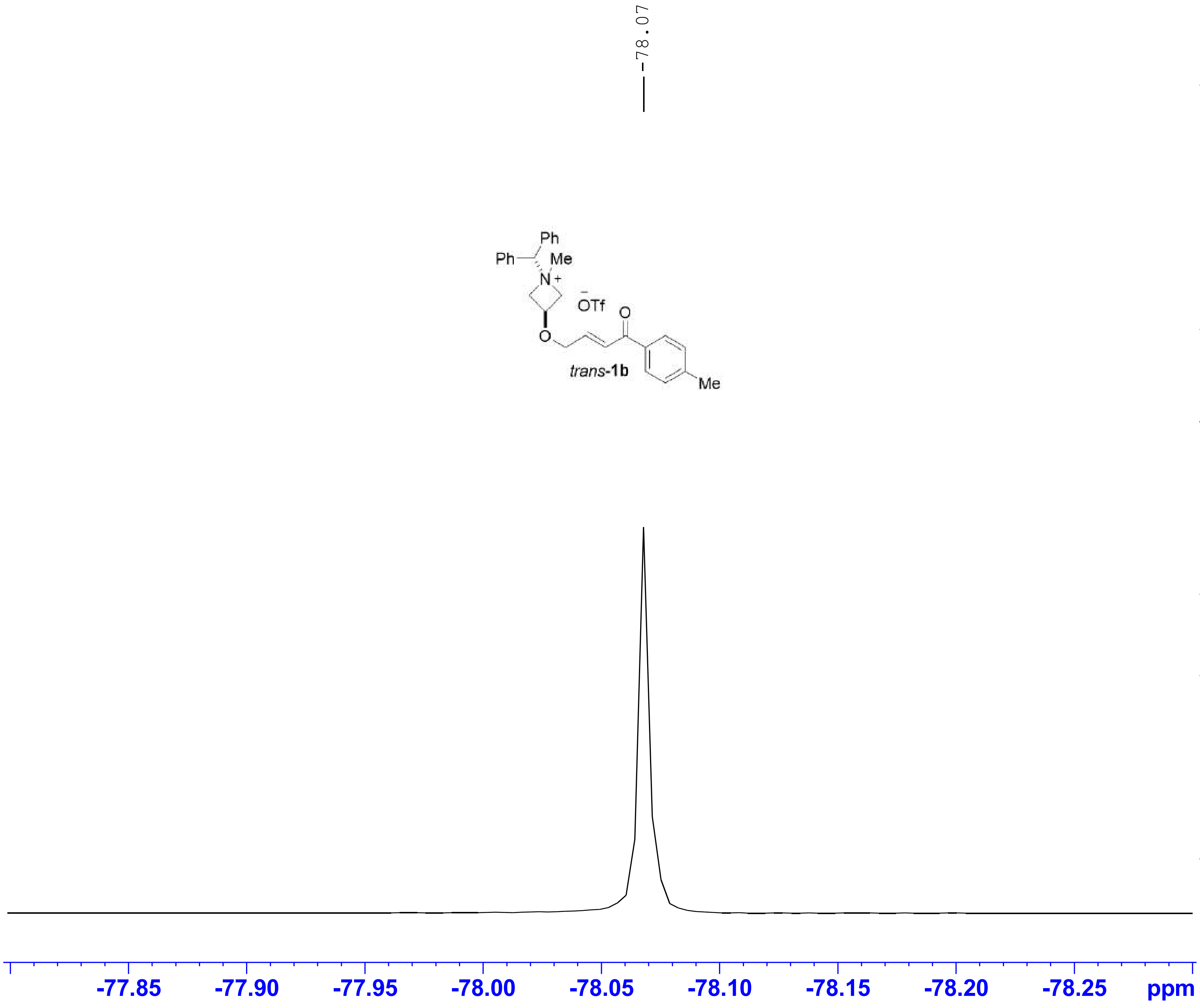


```
Current Data Parameters
NAME      zmh-sm-180a-shang
EXPNO                     9
PROCNO                    1

F2 - Acquisition Parameters
Date_              20240606
Time                23.32 h
INSTRUM   AvanceNeo 400MHz
PROBHD    Z163739_0629 (
PULPROG                zgig
TD                   131072
SOLVENT               CDCl3
NS                        8
DS                        4
SWH            90909.094 Hz
FIDRES          1.387163 Hz
AQ             0.7208960 sec
RG                      101
DW                5.500 usec
DE                 6.50 usec
TE                  297.3 K
D1            1.00000000 sec
D11           0.03000000 sec
TD0                       1
SFO1         376.5077587 MHz
NUC1                    19F
P1                12.00 usec
PLW1         33.72800064 W
SFO2         400.1816007 MHz
NUC2                     1H
CPDPRG[2            waltz16
PCPD2             90.00 usec
PLW2         21.26700020 W
PLW12         0.16802999 W

F2 - Processing parameters
SI                    65536
SF           376.5454132 MHz
WDW                      EM
SSB                       0
LB                  0.30 Hz
GB                        0
PC                     1.00
```

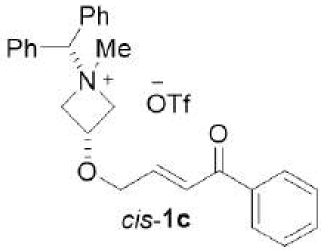

cis-**1c**

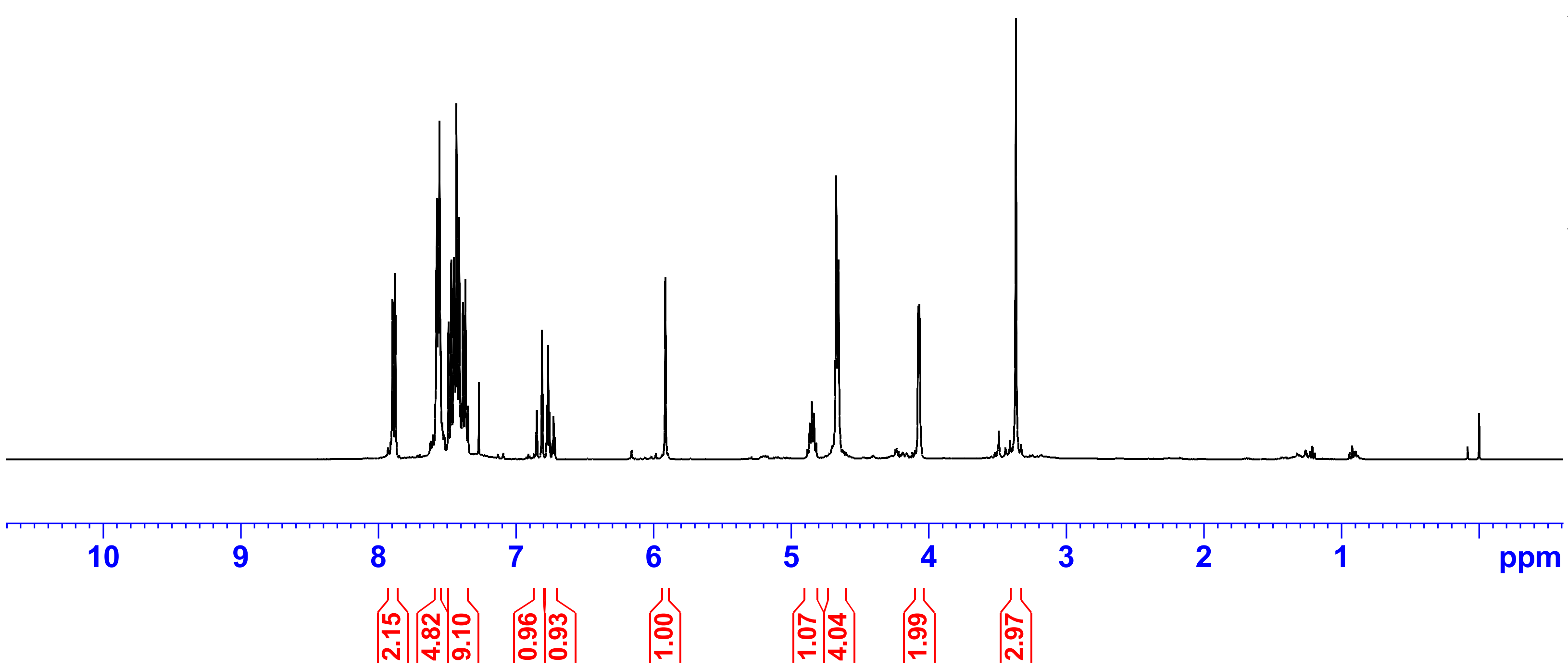

Current Data Parameters
NAME zmh-sm-180q
EXPNO 1
PROCNO 1

F2 - Acquisition Parameters
Date_ 20240607
Time 19.00 h
INSTRUM AvanceNeo 400MHz
PROBHD Z163739_0629 (
PULPROG zg30
TD 65536
SOLVENT CDCl3
NS 8
DS 2
SWH 8196.722 Hz
FIDRES 0.250144 Hz
AQ 3.9976959 sec
RG 101
DW 61.000 usec
DE 13.89 usec
TE 297.3 K
D1 1.00000000 sec
TD0 1
SFO1 400.1824711 MHz
NUC1 1H
P0 2.67 usec
P1 8.00 usec
PLW1 21.26700020 W

F2 - Processing parameters
SI 65536
SF 400.1800050 MHz
WDW EM
SSB 0
LB 0.30 Hz
GB 0
PC 1.00

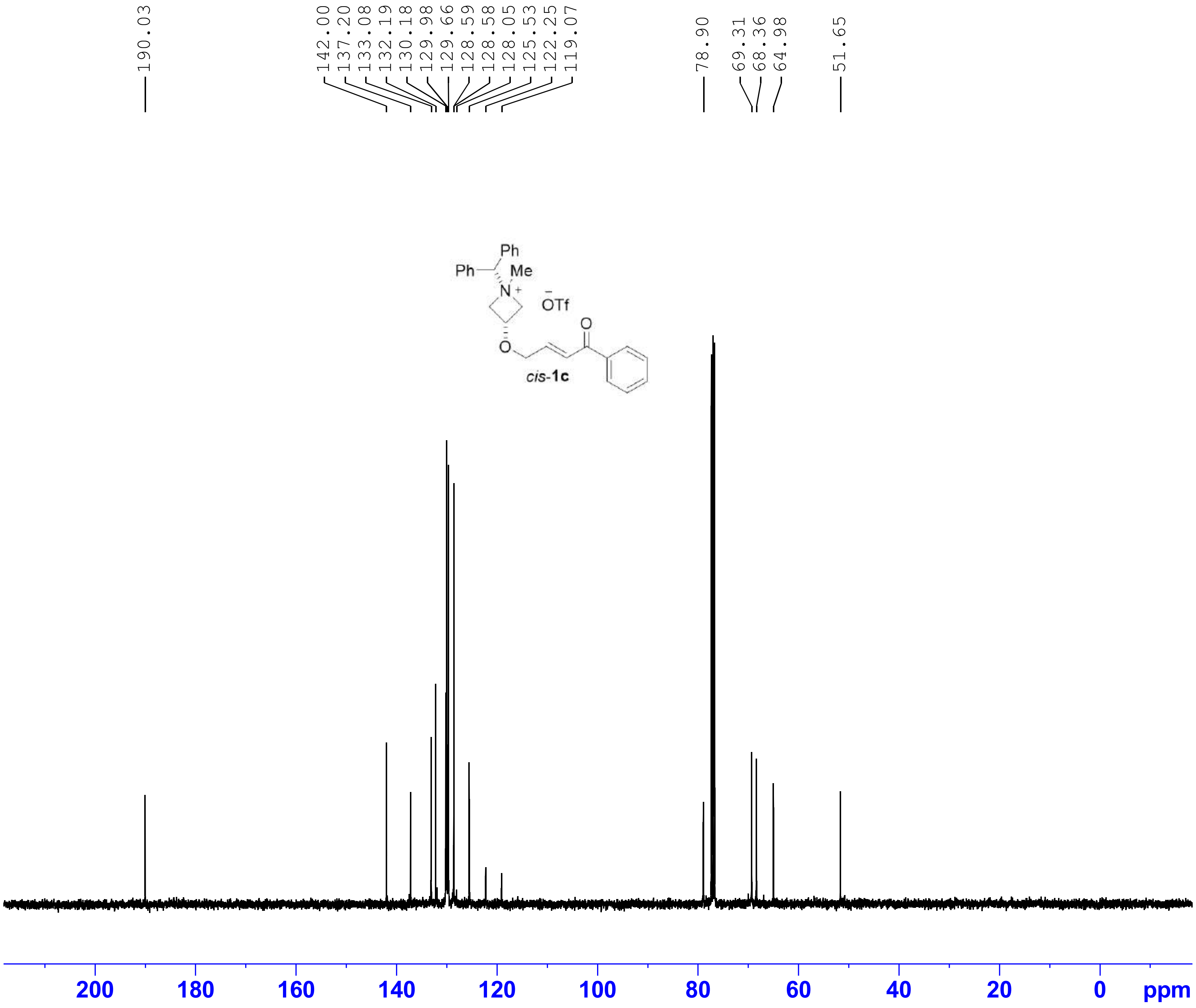

Current Data Parameters
NAME zmh-sm-180q
EXPNO 2
PROCNO 1

F2 - Acquisition Parameters
Date_ 20240607
Time 19.14 h
INSTRUM AvanceNeo 400MHz
PROBHD Z163739_0629 (
PULPROG zgpg30
TD 65536
SOLVENT CDCl3
NS 200
DS 4
SWH 23809.523 Hz
FIDRES 0.726609 Hz
AQ 1.3762560 sec
RG 22.6
DW 21.000 usec
DE 6.50 usec
TE 297.9 K
D1 2.00000000 sec
D11 0.03000000 sec
TD0 1
SFO1 100.6354036 MHz
NUC1 13C
P0 2.67 usec
P1 8.00 usec
PLW1 85.25399780 W
SFO2 400.1816007 MHz
NUC2 1H
CPDPRG[2 waltz65
PCPD2 90.00 usec
PLW2 21.26700020 W
PLW12 0.16802999 W
PLW13 0.08452000 W

F2 - Processing parameters
SI 32768
SF 100.6253506 MHz
WDW EM
SSB 0
LB 1.00 Hz
GB 0
PC 1.40

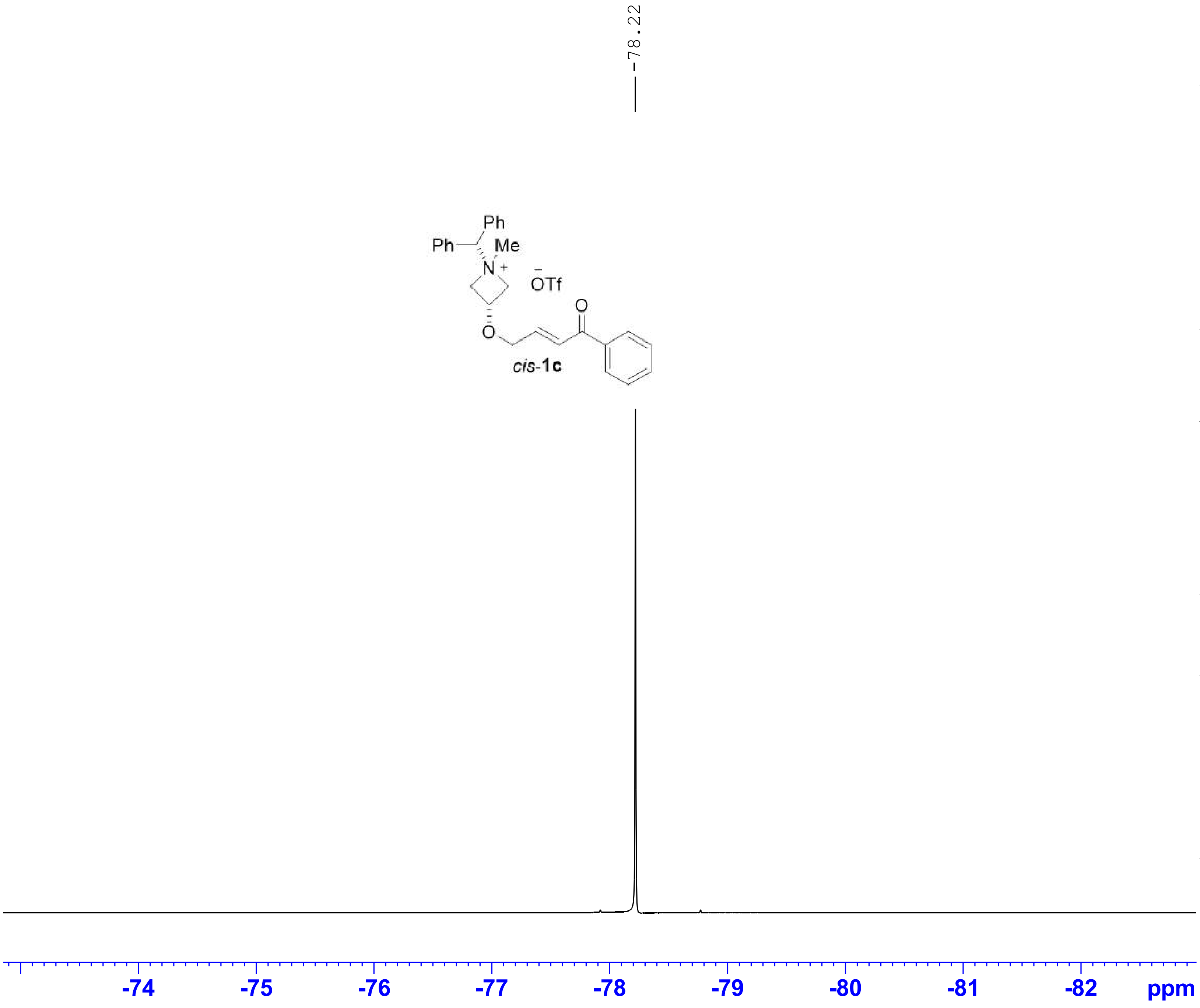

Current Data Parameters
NAME zmh-sm-180q
EXPNO 3
PROCNO 1

F2 - Acquisition Parameters
Date_ 20240607
Time 19.16 h
INSTRUM AvanceNeo 400MHz
PROBHD Z163739_0629 (
PULPROG zgig
TD 131072
SOLVENT CDCl3
NS 8
DS 4
SWH 90909.094 Hz
FIDRES 1.387163 Hz
AQ 0.7208960 sec
RG 101
DW 5.500 usec
DE 6.50 usec
TE 297.4 K
D1 1.00000000 sec
D11 0.03000000 sec
TD0 1
SFO1 376.5077587 MHz
NUC1 19F
P1 12.00 usec
PLW1 33.72800064 W
SFO2 400.1816007 MHz
NUC2 1H
CPDPRG[2 waltz16
PCPD2 90.00 usec
PLW2 21.26700020 W
PLW12 0.16802999 W

F2 - Processing parameters
SI 65536
SF 376.5454132 MHz
WDW EM
SSB 0
LB 0.30 Hz
GB 0
PC 1.00

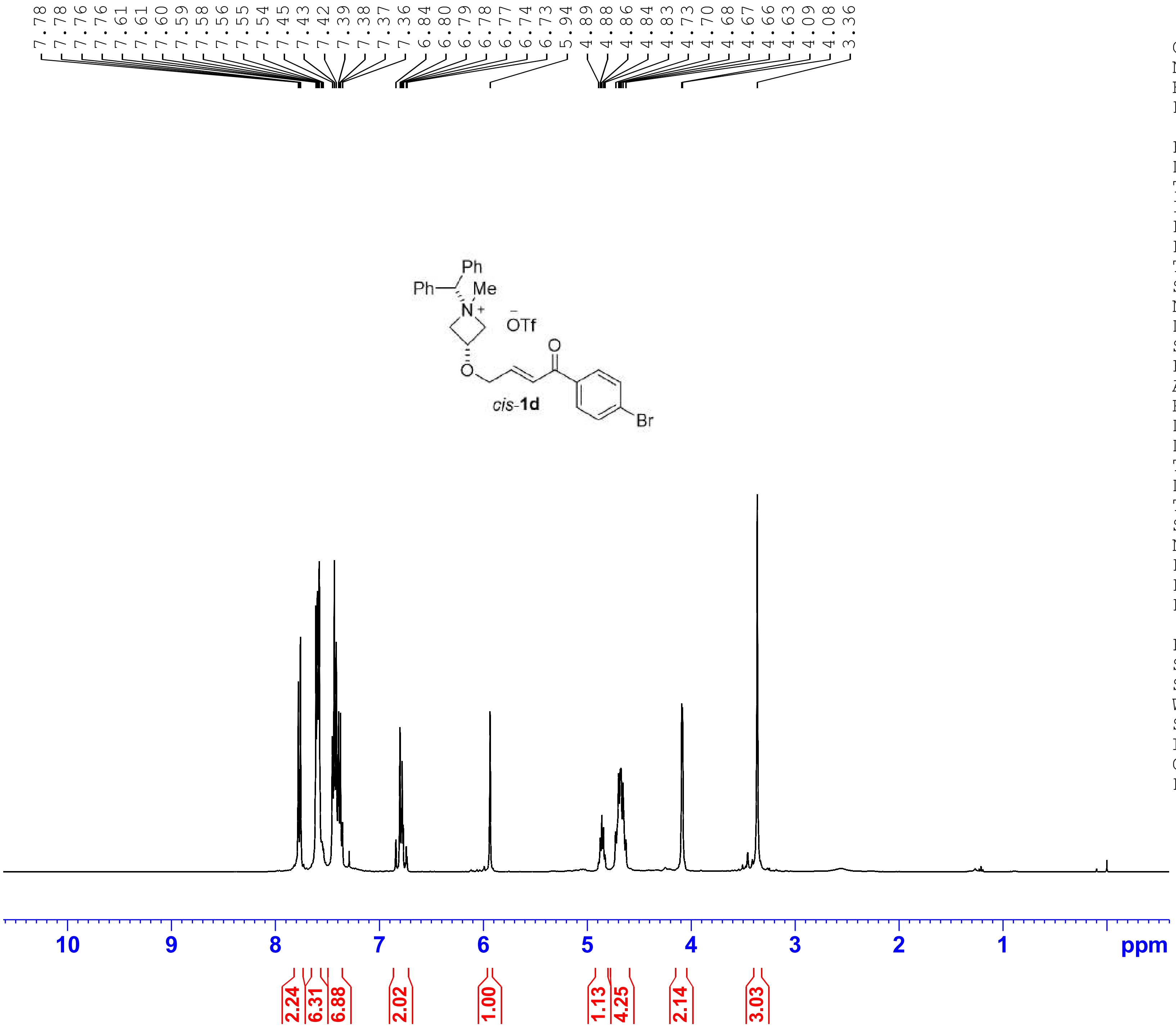

Current Data Parameters
NAME zmh-sm-180k
EXPNO 7
PROCNO 1

F2 - Acquisition Parameters
Date_ 20240607
Time 19.22 h
INSTRUM AvanceNeo 400MHz
PROBHD Z163739_0629 (
PULPROG zg30
TD 65536
SOLVENT CDCl3
NS 8
DS 2
SWH 8196.722 Hz
FIDRES 0.250144 Hz
AQ 3.9976959 sec
RG 36
DW 61.000 usec
DE 13.89 usec
TE 297.1 K
D1 1.00000000 sec
TD0 1
SFO1 400.1824711 MHz
NUC1 1H
P0 2.67 usec
P1 8.00 usec
PLW1 21.26700020 W

F2 - Processing parameters
SI 65536
SF 400.1799966 MHz
WDW EM
SSB 0
LB 0.30 Hz
GB 0
PC 1.00

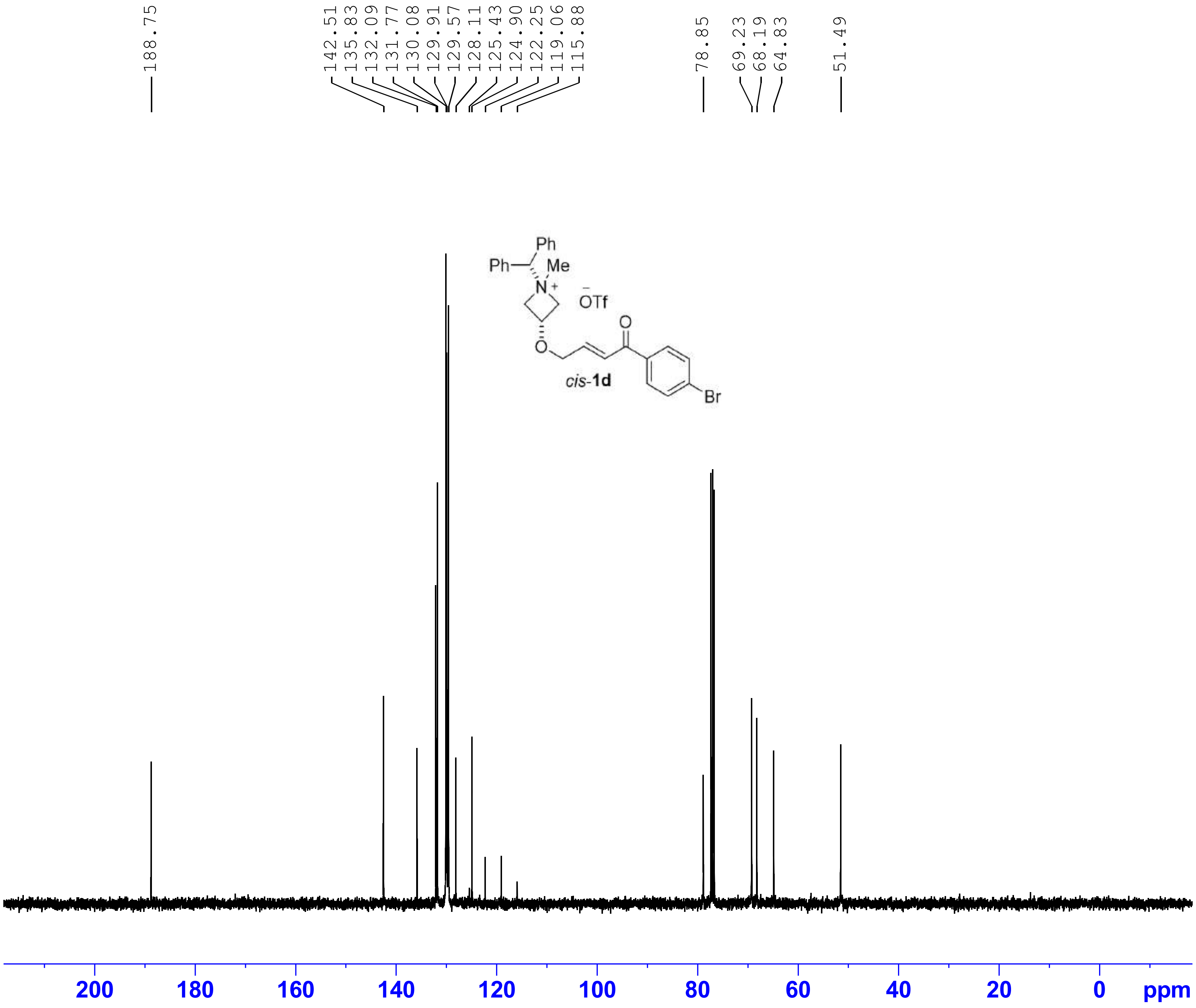

Current Data Parameters
NAME zmh-sm-180k
EXPNO 8
PROCNO 1

F2 - Acquisition Parameters
Date_ 20240607
Time 19.31 h
INSTRUM AvanceNeo 400MHz
PROBHD Z163739_0629 (
PULPROG zgpg30
TD 65536
SOLVENT CDCl3
NS 106
DS 4
SWH 23809.523 Hz
FIDRES 0.726609 Hz
AQ 1.3762560 sec
RG 10
DW 21.000 usec
DE 6.50 usec
TE 297.7 K
D1 2.00000000 sec
D11 0.03000000 sec
TD0 1
SFO1 100.6354036 MHz
NUC1 13C
P0 2.67 usec
P1 8.00 usec
PLW1 85.25399780 W
SFO2 400.1816007 MHz
NUC2 1H
CPDPRG[2 waltz65
PCPD2 90.00 usec
PLW2 21.26700020 W
PLW12 0.16802999 W
PLW13 0.08452000 W

F2 - Processing parameters
SI 32768
SF 100.6253585 MHz
WDW EM
SSB 0
LB 1.00 Hz
GB 0
PC 1.40

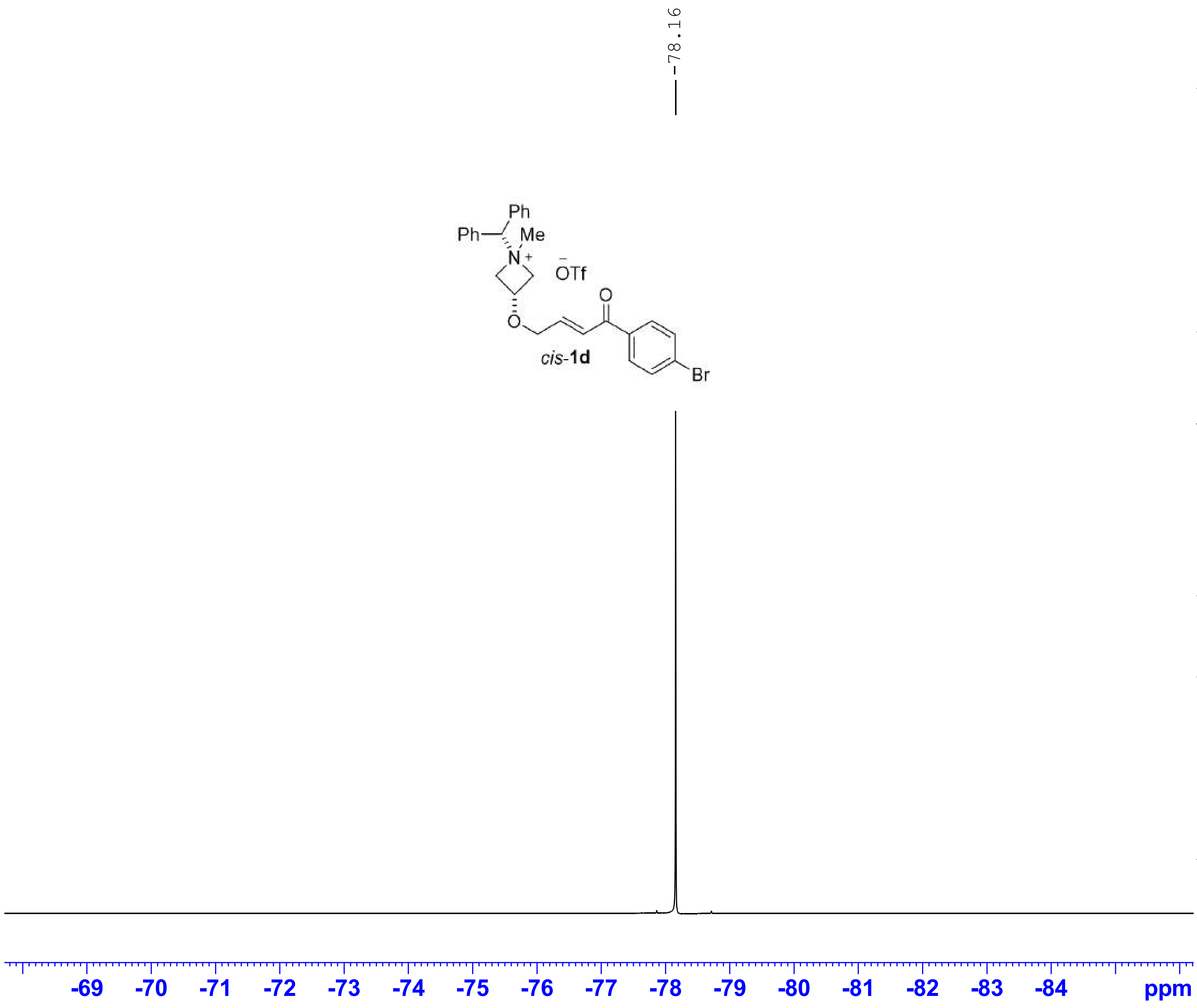

cis-**1d**

Current Data Parameters
NAME zmh-sm-180k
EXPNO 9
PROCNO 1

F2 - Acquisition Parameters
Date_ 20240607
Time 19.33 h
INSTRUM AvanceNeo 400MHz
PROBHD Z163739_0629 (
PULPROG zgig
TD 131072
SOLVENT CDCl3
NS 8
DS 4
SWH 90909.094 Hz
FIDRES 1.387163 Hz
AQ 0.7208960 sec
RG 101
DW 5.500 usec
DE 6.50 usec
TE 297.2 K
D1 1.00000000 sec
D11 0.03000000 sec
TD0 1
SFO1 376.5077587 MHz
NUC1 19F
P1 12.00 usec
PLW1 33.72800064 W
SFO2 400.1816007 MHz
NUC2 1H
CPDPRG[2 waltz16
PCPD2 90.00 usec
PLW2 21.26700020 W
PLW12 0.16802999 W

F2 - Processing parameters
SI 65536
SF 376.5454132 MHz
WDW EM
SSB 0
LB 0.30 Hz
GB 0
PC 1.00

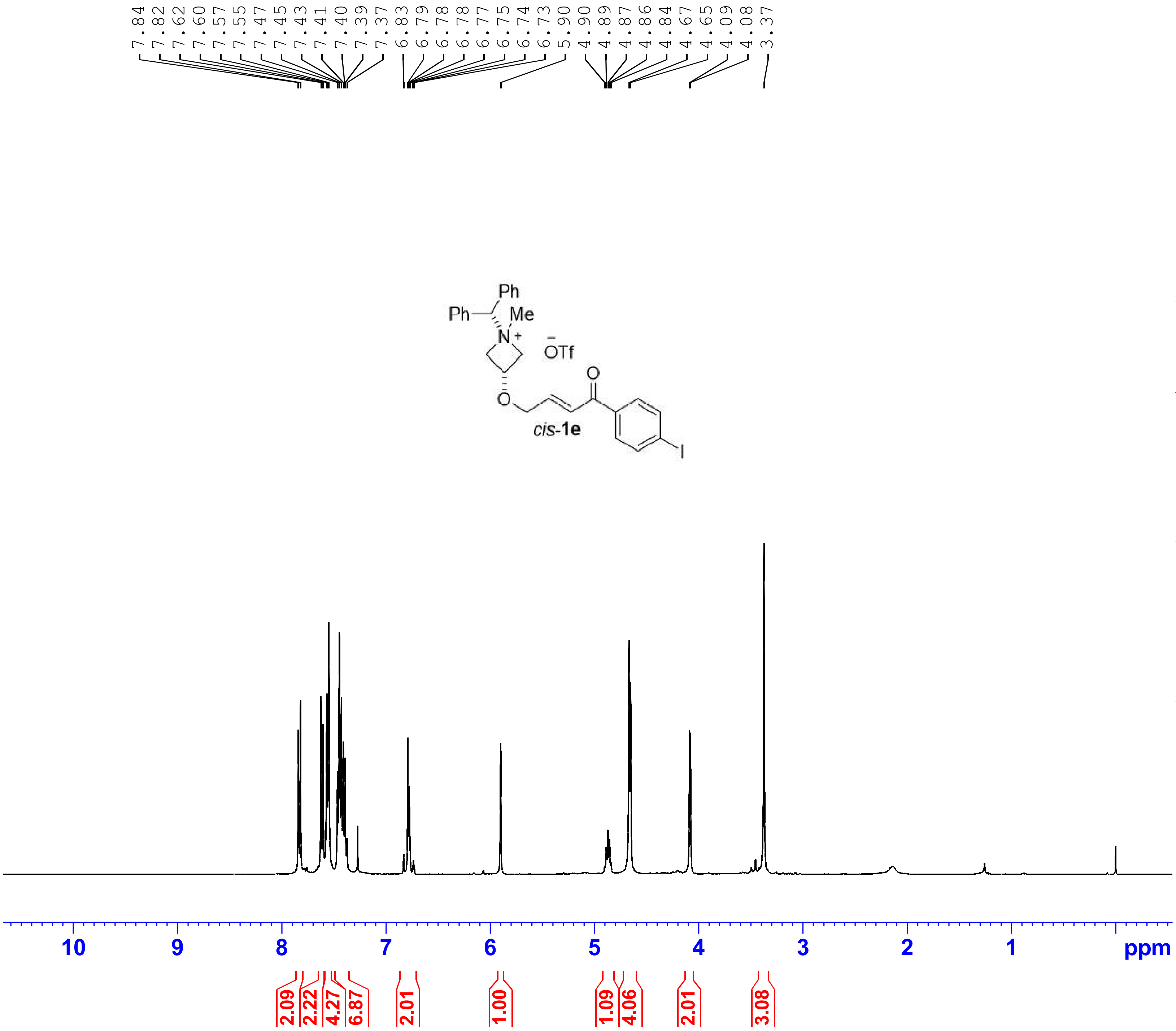

Current Data Parameters
NAME zmh-sm-180p
EXPNO 1
PROCNO 1

F2 - Acquisition Parameters
Date_ 20240607
Time 1.01 h
INSTRUM AvanceNeo 400MHz
PROBHD Z163739_0629 (
PULPROG zg30
TD 65536
SOLVENT CDCl3
NS 8
DS 2
SWH 8196.722 Hz
FIDRES 0.250144 Hz
AQ 3.9976959 sec
RG 101
DW 61.000 usec
DE 13.89 usec
TE 297.2 K
D1 1.00000000 sec
TD0 1
SFO1 400.1824711 MHz
NUC1 1H
P0 2.67 usec
P1 8.00 usec
PLW1 21.26700020 W

F2 - Processing parameters
SI 65536
SF 400.1800047 MHz
WDW EM
SSB 0
LB 0.30 Hz
GB 0
PC 1.00

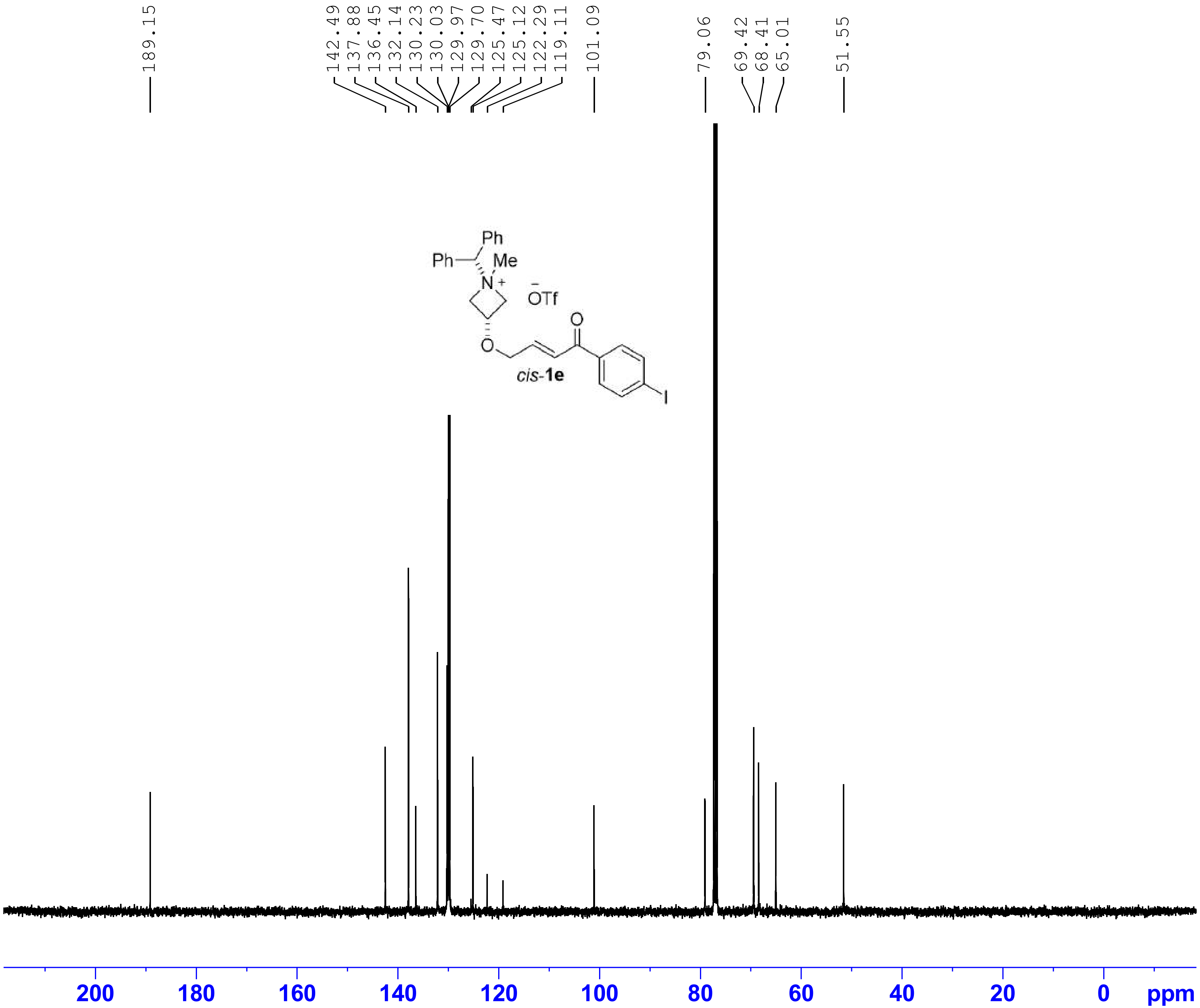

Current Data Parameters
NAME zmh-sm-180p
EXPNO 2
PROCNO 1

F2 - Acquisition Parameters
Date_ 20240607
Time 1.26 h
INSTRUM AvanceNeo 400MHz
PROBHD Z163739_0629 (
PULPROG zgpg30
TD 65536
SOLVENT CDCl3
NS 400
DS 4
SWH 23809.523 Hz
FIDRES 0.726609 Hz
AQ 1.3762560 sec
RG 10
DW 21.000 usec
DE 6.50 usec
TE 297.9 K
D1 2.00000000 sec
D11 0.03000000 sec
TD0 1
SFO1 100.6354036 MHz
NUC1 13C
P0 2.67 usec
P1 8.00 usec
PLW1 85.25399780 W
SFO2 400.1816007 MHz
NUC2 1H
CPDPRG[2 waltz65
PCPD2 90.00 usec
PLW2 21.26700020 W
PLW12 0.16802999 W
PLW13 0.08452000 W

F2 - Processing parameters
SI 32768
SF 100.6253499 MHz
WDW EM
SSB 0
LB 1.00 Hz
GB 0
PC 1.40

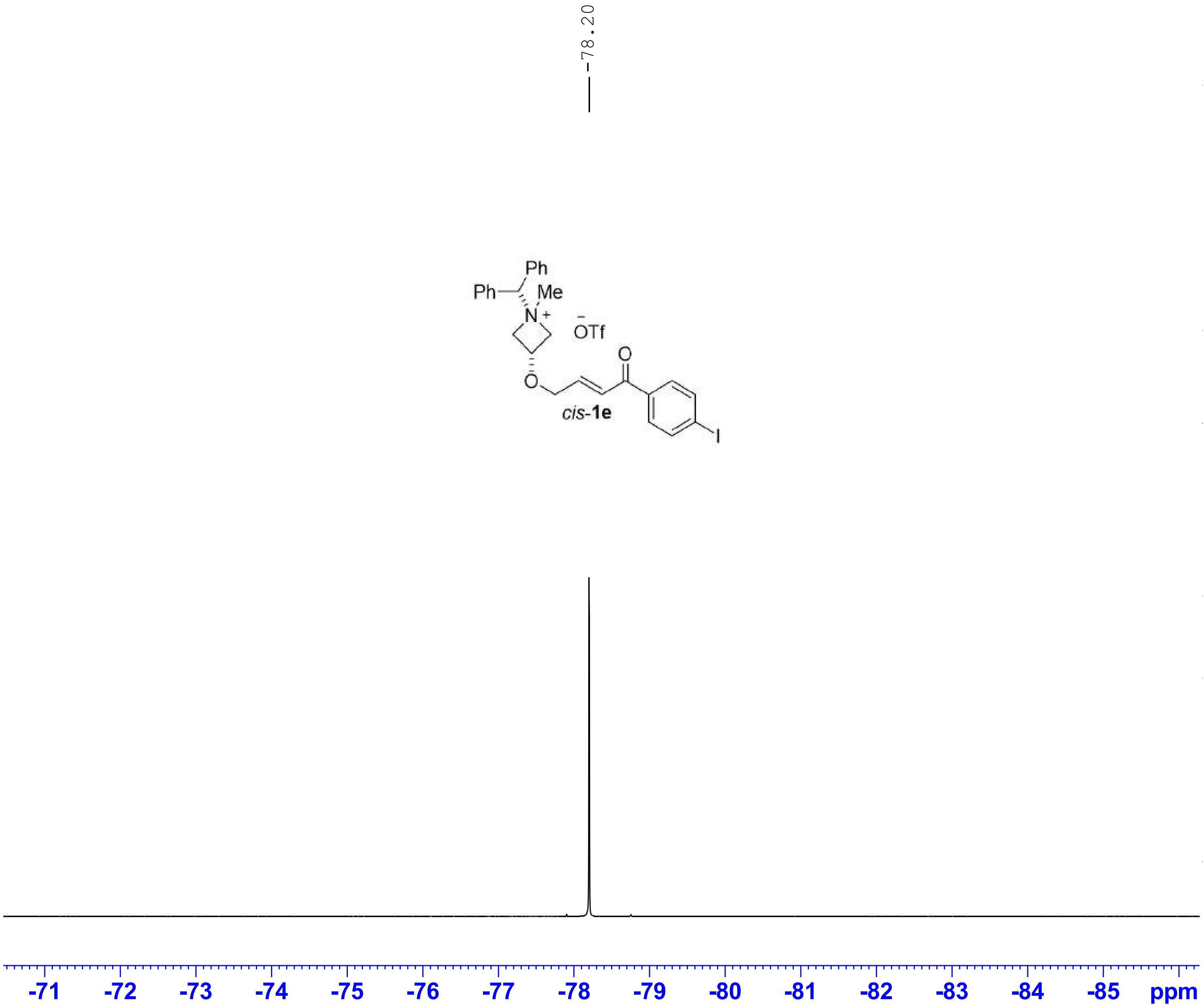

cis-**1e**

```
Current Data Parameters
NAME          zmh-sm-180p
EXPNO                   3
PROCNO                  1

F2 - Acquisition Parameters
Date_            20240607
Time                 1.28 h
INSTRUM   AvanceNeo 400MHz
PROBHD   Z163739_0629 (
PULPROG              zgig
TD                 131072
SOLVENT             CDCl3
NS                      8
DS                      4
SWH             90909.094 Hz
FIDRES           1.387163 Hz
AQ              0.7208960 sec
RG                    101
DW                  5.500 usec
DE                   6.50 usec
TE                  297.4 K
D1             1.00000000 sec
D11            0.03000000 sec
TD0                     1
SFO1          376.5077587 MHz
NUC1                  19F
P1                  12.00 usec
PLW1          33.72800064 W
SFO2          400.1816007 MHz
NUC2                   1H
CPDPRG[2          waltz16
PCPD2               90.00 usec
PLW2          21.26700020 W
PLW12          0.16802999 W

F2 - Processing parameters
SI                  65536
SF            376.5454132 MHz
WDW                    EM
SSB                     0
LB                   0.30 Hz
GB                      0
PC                   1.00
```

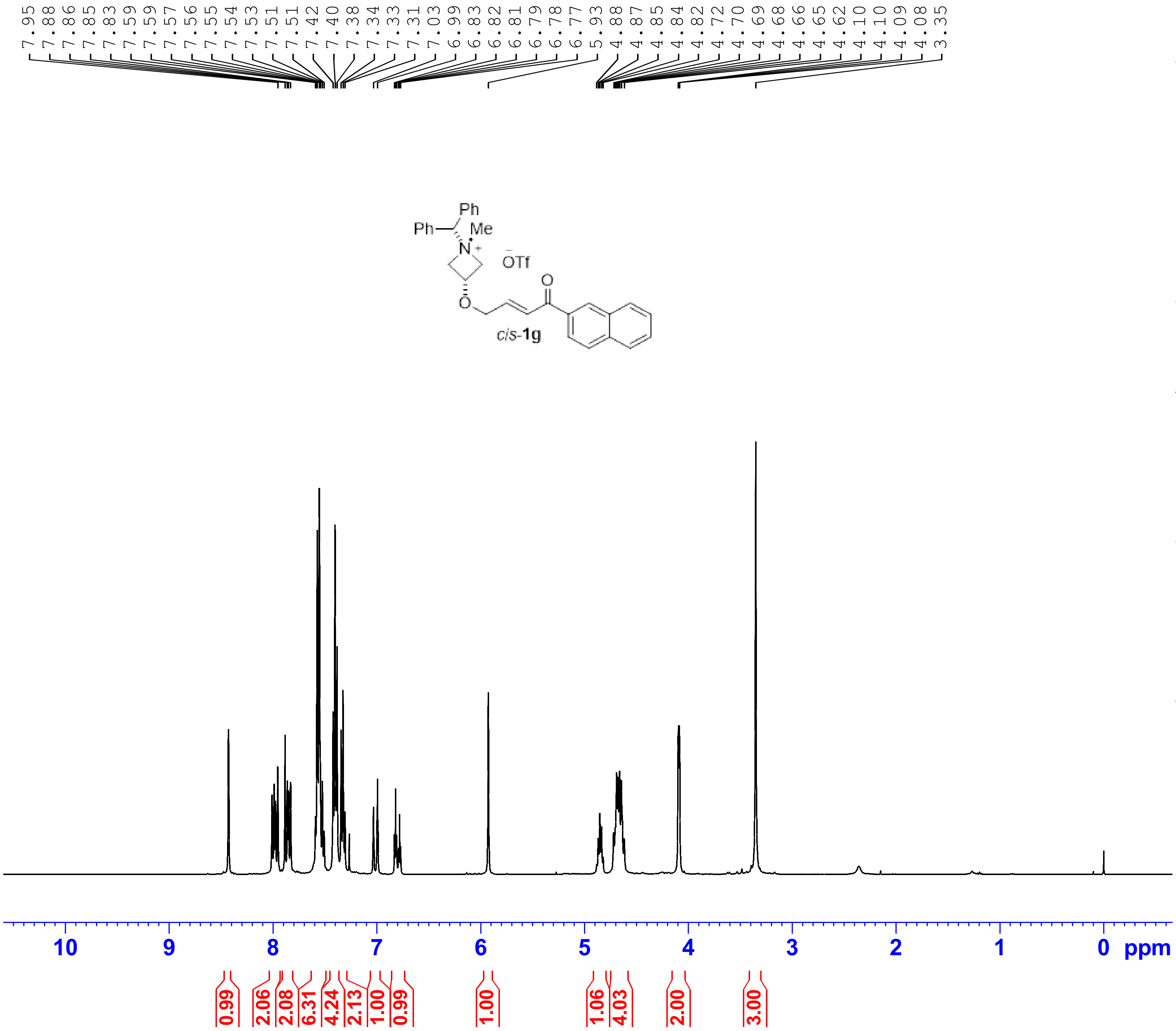

Current Data Parameters
NAME zmh-sm-1801
EXPNO 10
PROCNO 1

F2 - Acquisition Parameters
Date_ 20240607
Time 2.04 h
INSTRUM AvanceNeo 400MHz
PROBHD Z163739_0629 (
PULPROG zg30
TD 65536
SOLVENT CDCl3
NS 8
DS 2
SWH 8196.722 Hz
FIDRES 0.250144 Hz
AQ 3.9976959 sec
RG 45.2
DW 61.000 usec
DE 13.89 usec
TE 297.2 K
D1 1.00000000 sec
TD0 1
SFO1 400.1824711 MHz
NUC1 1H
P0 2.67 usec
P1 8.00 usec
PLW1 21.26700020 W

F2 - Processing parameters
SI 65536
SF 400.1800066 MHz
WDW EM
SSB 0
LB 0.30 Hz
GB 0
PC 1.00

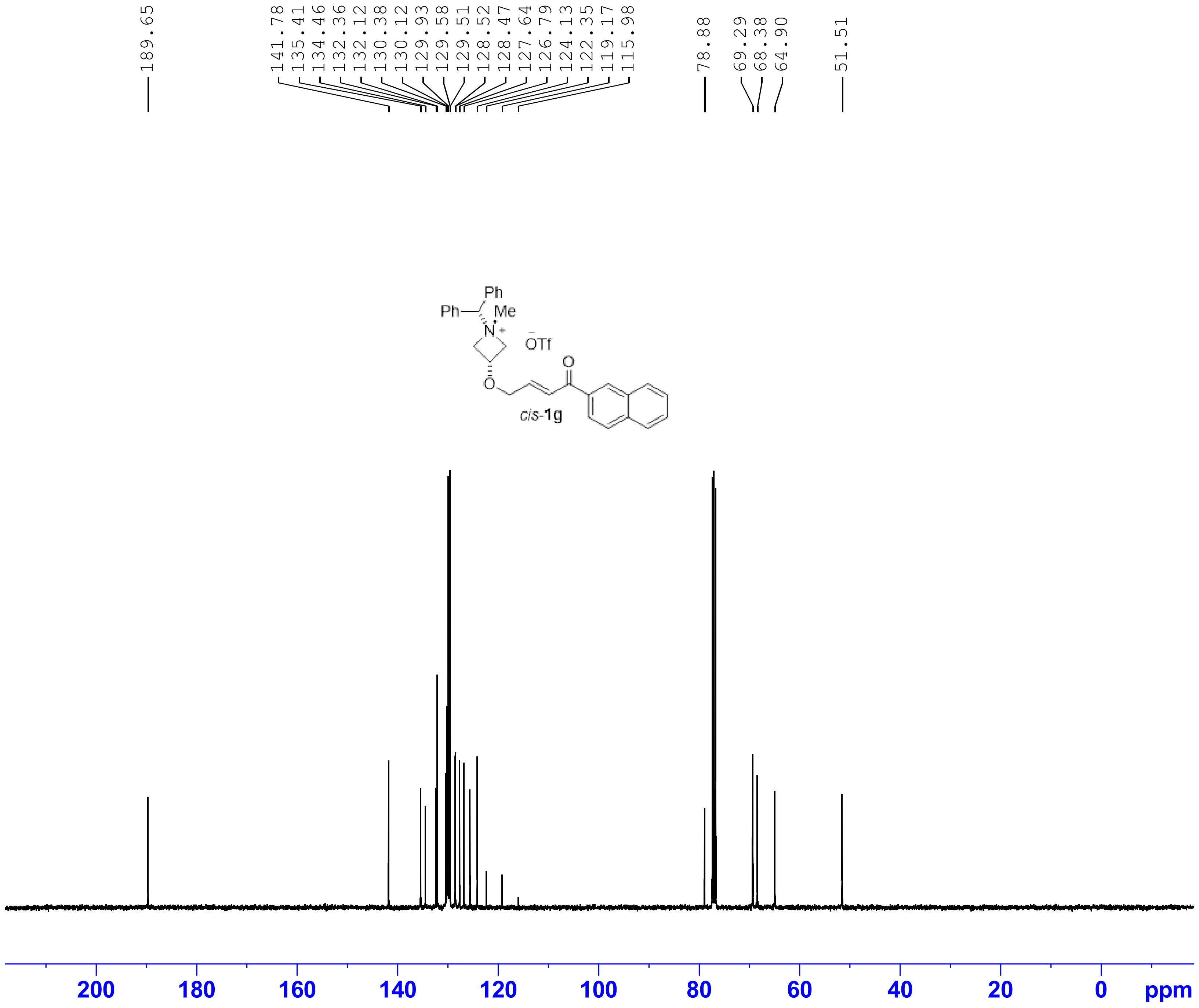

Current Data Parameters
NAME zmh-sm-1801
EXPNO 11
PROCNO 1

F2 - Acquisition Parameters
Date_ 20240607
Time 2.29 h
INSTRUM AvanceNeo 400MHz
PROBHD Z163739_0629 (
PULPROG zgpg30
TD 65536
SOLVENT CDCl3
NS 400
DS 4
SWH 23809.523 Hz
FIDRES 0.726609 Hz
AQ 1.3762560 sec
RG 10
DW 21.000 usec
DE 6.50 usec
TE 297.9 K
D1 2.00000000 sec
D11 0.03000000 sec
TD0 1
SFO1 100.6354036 MHz
NUC1 13C
P0 2.67 usec
P1 8.00 usec
PLW1 85.25399780 W
SFO2 400.1816007 MHz
NUC2 1H
CPDPRG[2 waltz65
PCPD2 90.00 usec
PLW2 21.26700020 W
PLW12 0.16802999 W
PLW13 0.08452000 W

F2 - Processing parameters
SI 32768
SF 100.6253565 MHz
WDW EM
SSB 0
LB 1.00 Hz
GB 0
PC 1.40

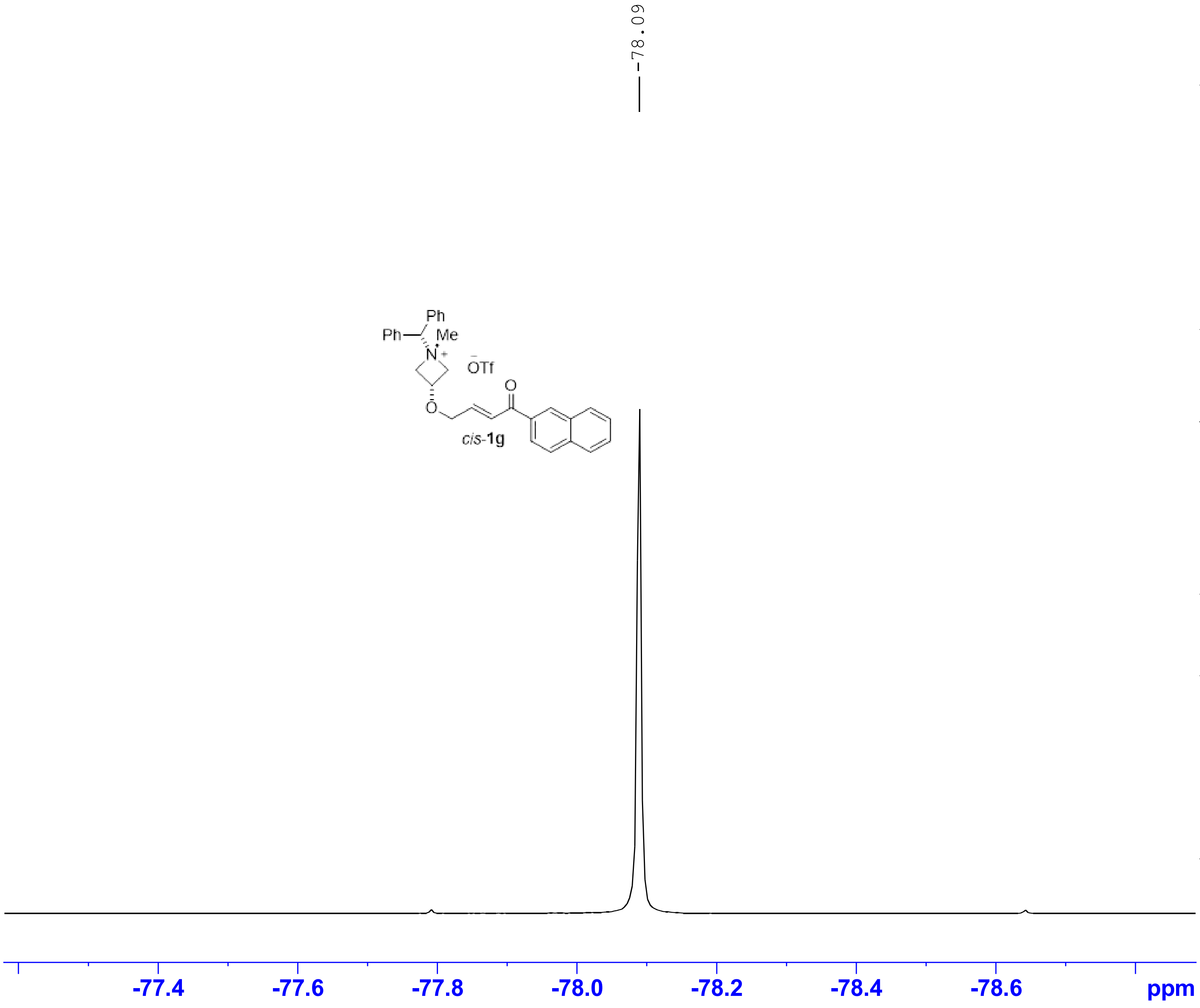

Current Data Parameters
NAME zmh-sm-1801
EXPNO 12
PROCNO 1

F2 - Acquisition Parameters
Date_ 20240607
Time 2.31 h
INSTRUM AvanceNeo 400MHz
PROBHD Z163739_0629 (
PULPROG zgig
TD 131072
SOLVENT CDCl3
NS 8
DS 4
SWH 90909.094 Hz
FIDRES 1.387163 Hz
AQ 0.7208960 sec
RG 101
DW 5.500 usec
DE 6.50 usec
TE 297.4 K
D1 1.00000000 sec
D11 0.03000000 sec
TD0 1
SFO1 376.5077587 MHz
NUC1 19F
P1 12.00 usec
PLW1 33.72800064 W
SFO2 400.1816007 MHz
NUC2 1H
CPDPRG[2 waltz16
PCPD2 90.00 usec
PLW2 21.26700020 W
PLW12 0.16802999 W

F2 - Processing parameters
SI 65536
SF 376.5454132 MHz
WDW EM
SSB 0
LB 0.30 Hz
GB 0
PC 1.00

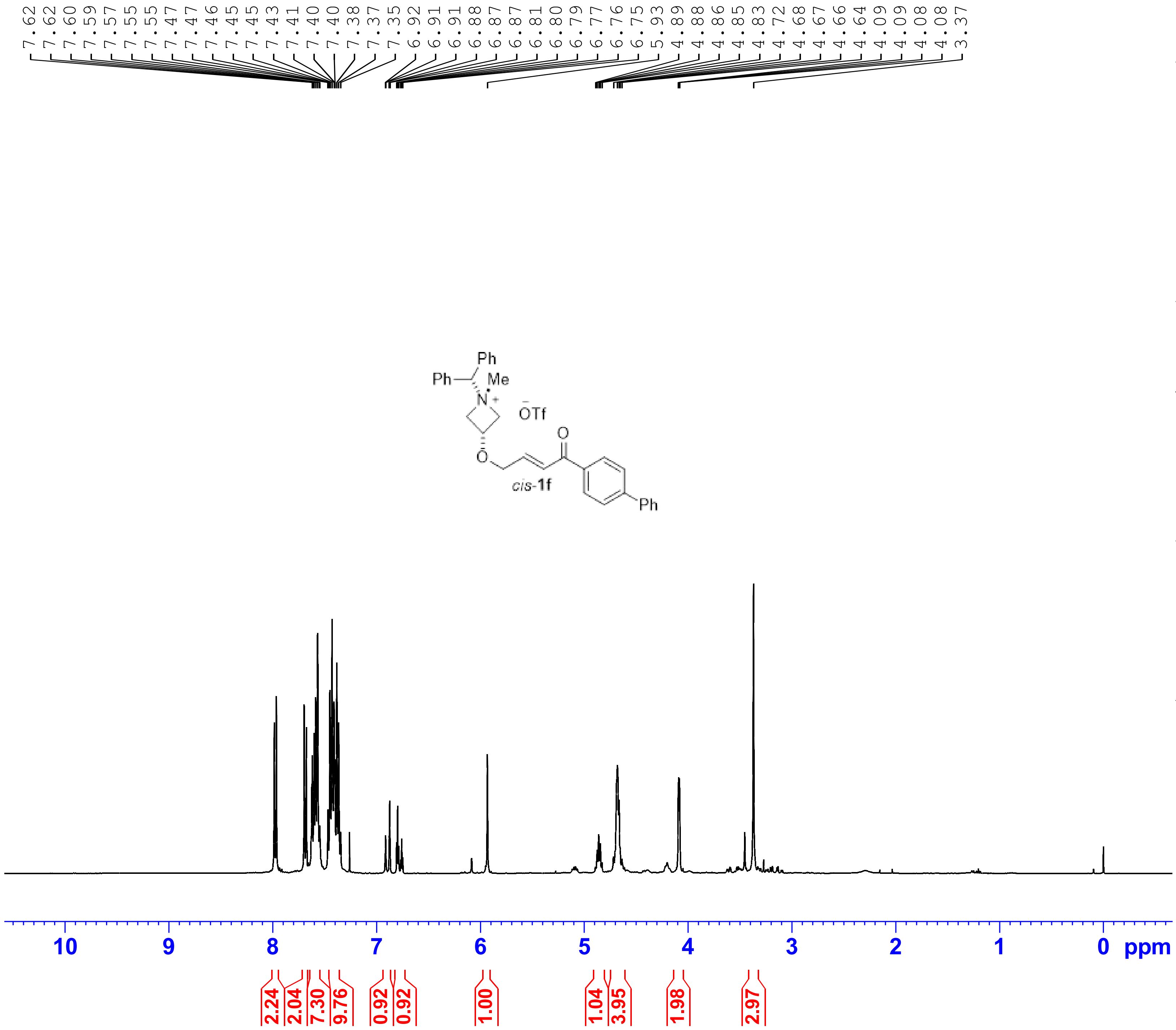

Current Data Parameters
NAME zmh-sm-180n-xia
EXPNO 17
PROCNO 1

F2 - Acquisition Parameters
Date_ 20240606
Time 23.59 h
INSTRUM AvanceNeo 400MHz
PROBHD Z163739_0629 (
PULPROG zg30
TD 65536
SOLVENT CDCl3
NS 8
DS 2
SWH 8196.722 Hz
FIDRES 0.250144 Hz
AQ 3.9976959 sec
RG 45.2
DW 61.000 usec
DE 13.89 usec
TE 297.2 K
D1 1.00000000 sec
TD0 1
SFO1 400.1824711 MHz
NUC1 1H
P0 2.67 usec
P1 8.00 usec
PLW1 21.26700020 W

F2 - Processing parameters
SI 65536
SF 400.1800090 MHz
WDW EM
SSB 0
LB 0.30 Hz
GB 0
PC 1.00

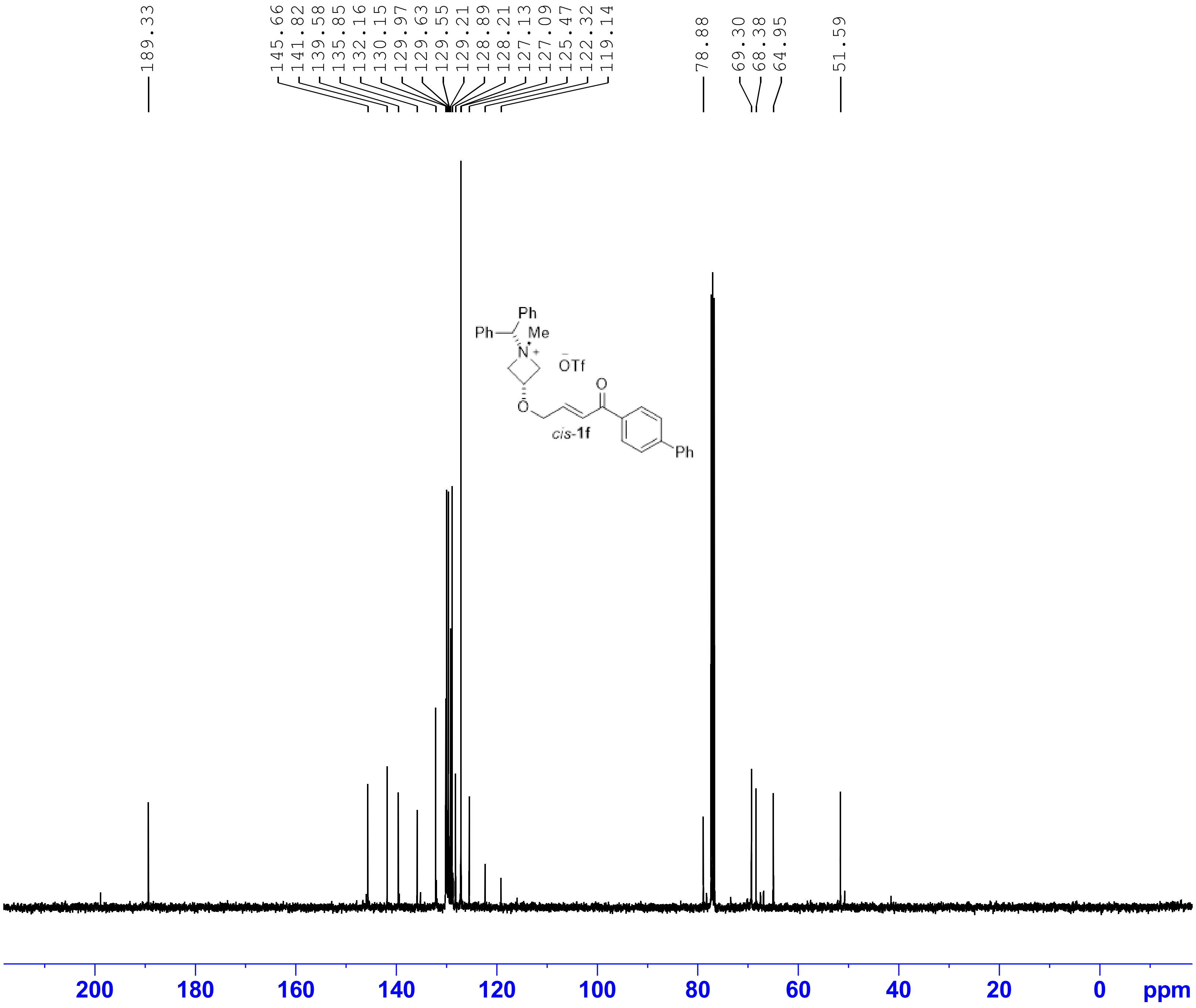

Current Data Parameters
NAME zmh-sm-180n-xia
EXPNO 18
PROCNO 1

F2 - Acquisition Parameters
Date_ 20240607
Time 0.12 h
INSTRUM AvanceNeo 400MHz
PROBHD Z163739_0629 (
PULPROG zgpg30
TD 65536
SOLVENT CDCl3
NS 200
DS 4
SWH 23809.523 Hz
FIDRES 0.726609 Hz
AQ 1.3762560 sec
RG 10
DW 21.000 usec
DE 6.50 usec
TE 297.8 K
D1 2.00000000 sec
D11 0.03000000 sec
TD0 1
SFO1 100.6354036 MHz
NUC1 13C
P0 2.67 usec
P1 8.00 usec
PLW1 85.25399780 W
SFO2 400.1816007 MHz
NUC2 1H
CPDPRG[2 waltz65
PCPD2 90.00 usec
PLW2 21.26700020 W
PLW12 0.16802999 W
PLW13 0.08452000 W

F2 - Processing parameters
SI 32768
SF 100.6253551 MHz
WDW EM
SSB 0
LB 1.00 Hz
GB 0
PC 1.40

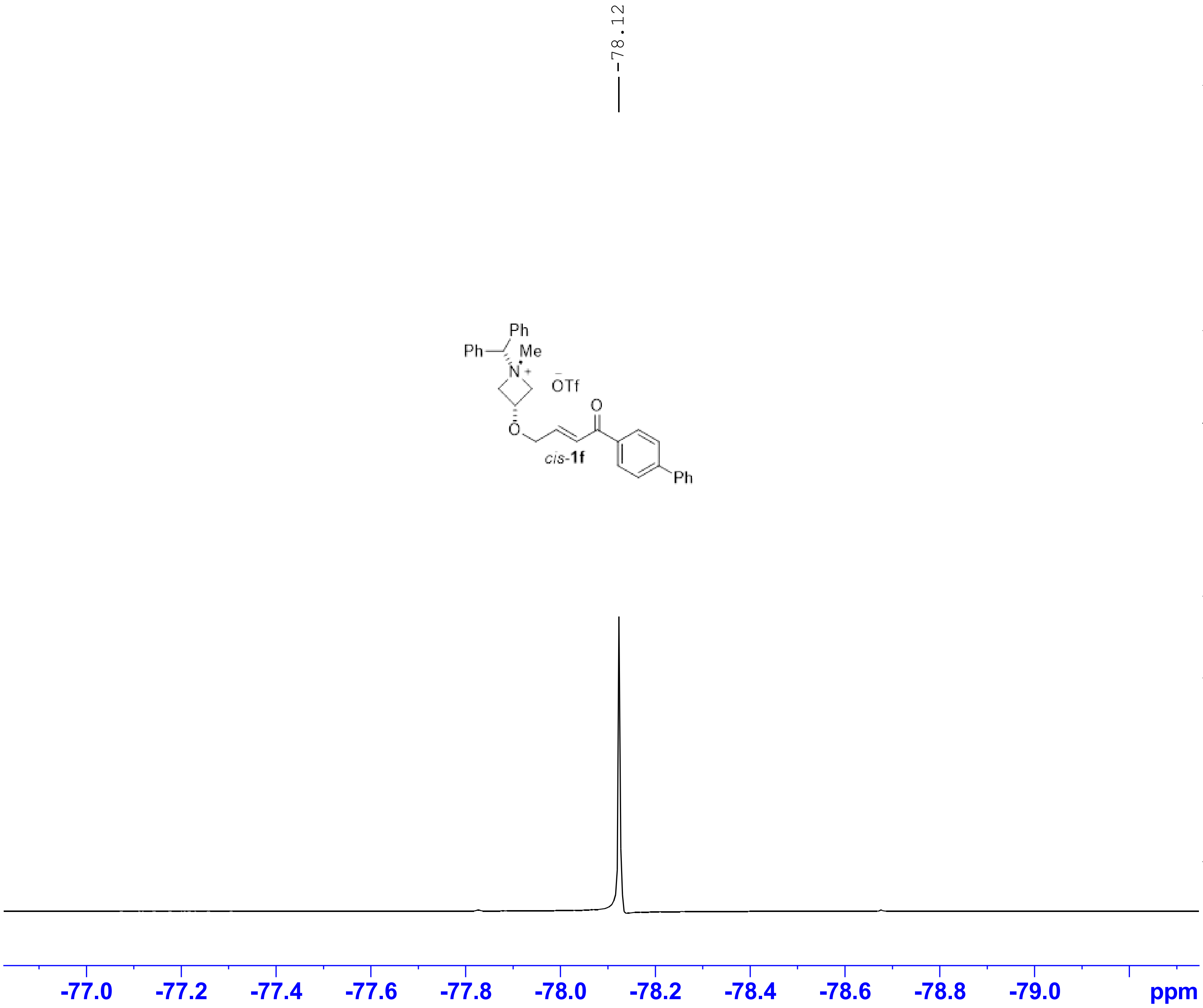

Current Data Parameters
NAME zmh-sm-180n-xia
EXPNO 19
PROCNO 1

F2 - Acquisition Parameters
Date_ 20240607
Time 0.15 h
INSTRUM AvanceNeo 400MHz
PROBHD Z163739_0629 (
PULPROG zgig
TD 131072
SOLVENT CDCl3
NS 8
DS 4
SWH 90909.094 Hz
FIDRES 1.387163 Hz
AQ 0.7208960 sec
RG 101
DW 5.500 usec
DE 6.50 usec
TE 297.4 K
D1 1.00000000 sec
D11 0.03000000 sec
TD0 1
SFO1 376.5077587 MHz
NUC1 19F
P1 12.00 usec
PLW1 33.72800064 W
SFO2 400.1816007 MHz
NUC2 1H
CPDPRG[2 waltz16
PCPD2 90.00 usec
PLW2 21.26700020 W
PLW12 0.16802999 W

F2 - Processing parameters
SI 65536
SF 376.5454132 MHz
WDW EM
SSB 0
LB 0.30 Hz
GB 0
PC 1.00

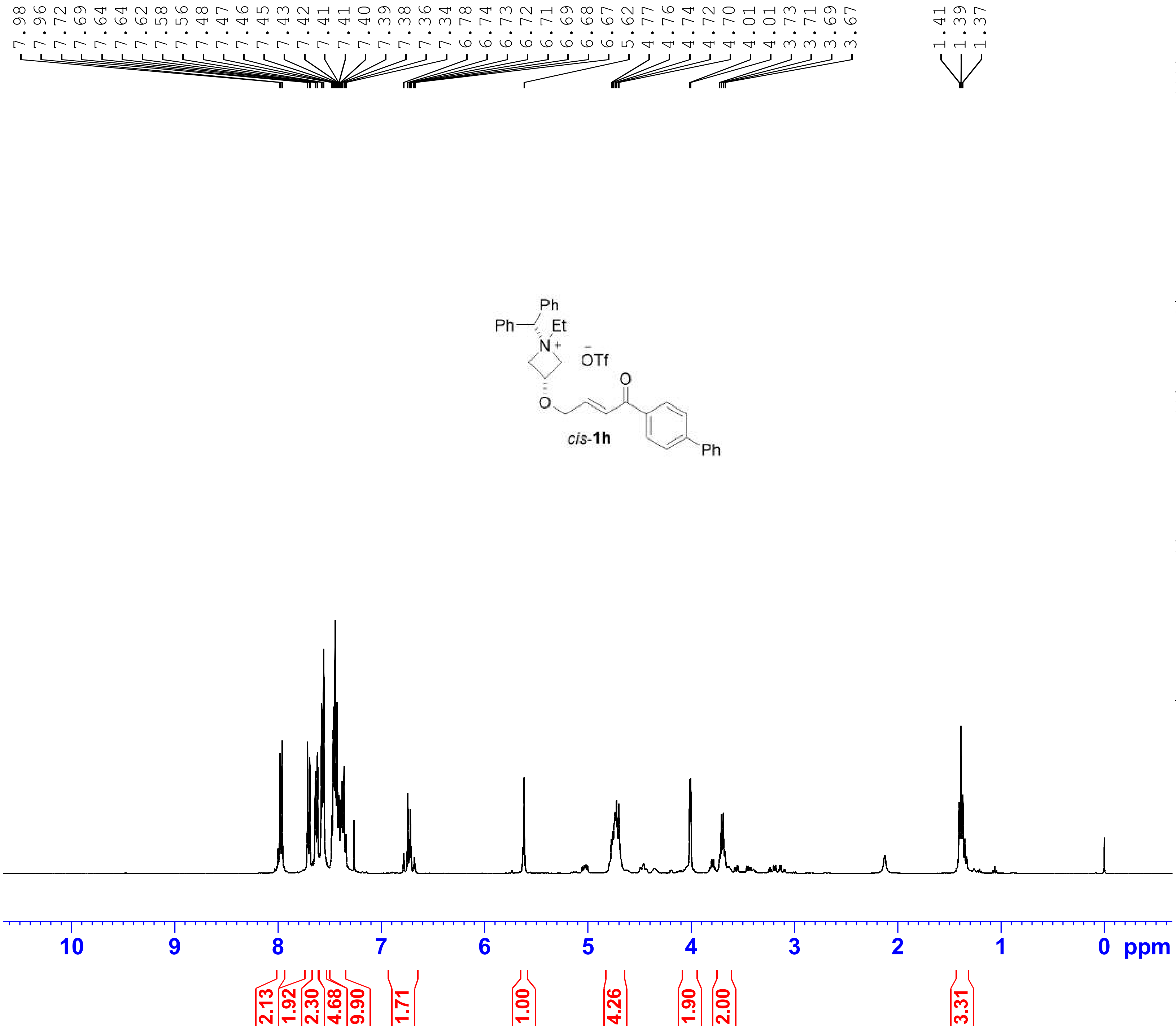

Current Data Parameters
NAME zmh-sm-180o
EXPNO 1
PROCNO 1

F2 - Acquisition Parameters
Date_ 20240606
Time 19.12 h
INSTRUM AvanceNeo 400MHz
PROBHD Z163739_0629 (
PULPROG zg30
TD 65536
SOLVENT CDCl3
NS 5
DS 2
SWH 8196.722 Hz
FIDRES 0.250144 Hz
AQ 3.9976959 sec
RG 101
DW 61.000 usec
DE 13.89 usec
TE 296.6 K
D1 1.00000000 sec
TD0 1
SFO1 400.1824711 MHz
NUC1 1H
P0 2.67 usec
P1 8.00 usec
PLW1 21.26700020 W

F2 - Processing parameters
SI 65536
SF 400.1800078 MHz
WDW EM
SSB 0
LB 0.30 Hz
GB 0
PC 1.00

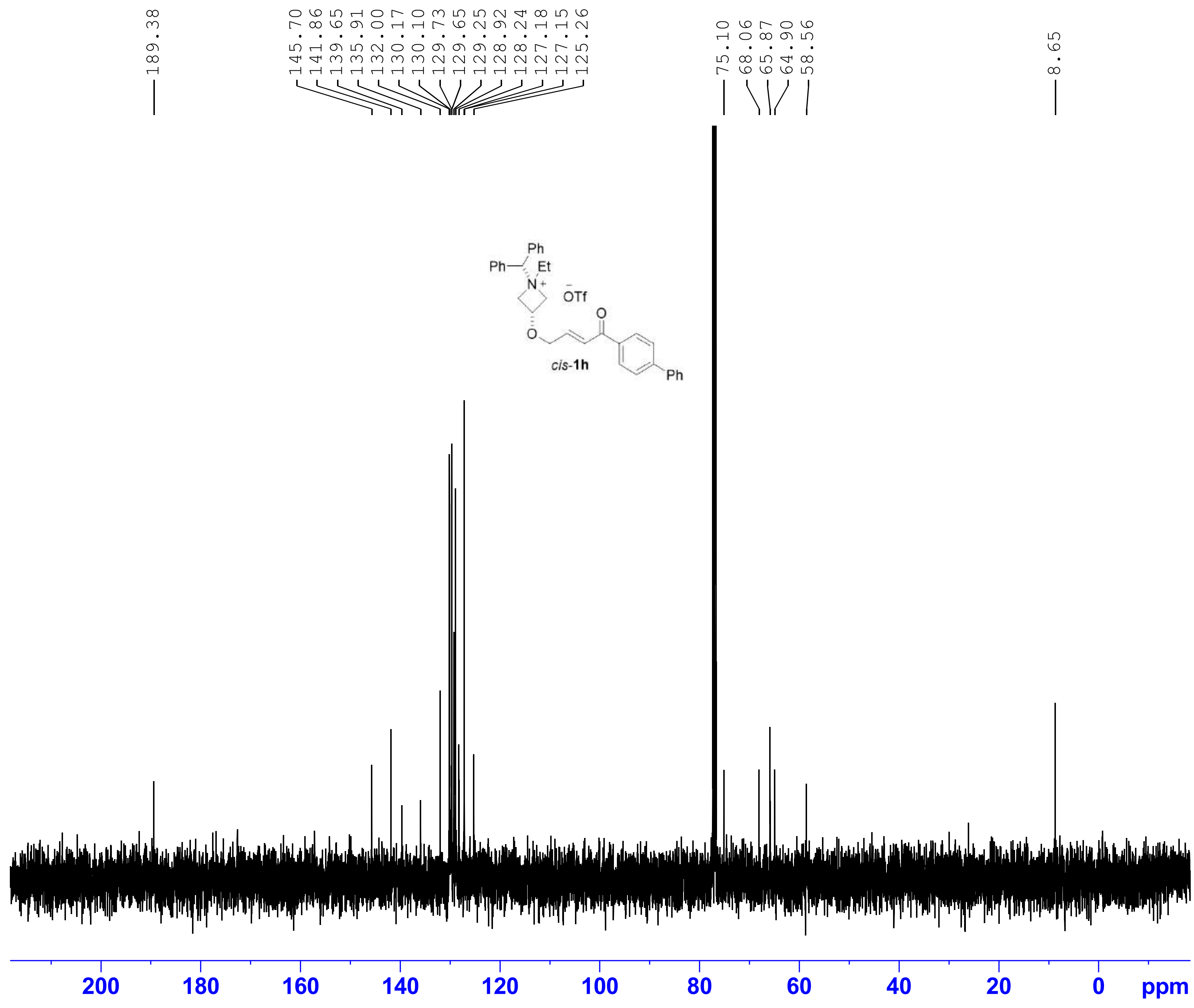

Current Data Parameters
NAME zmh-sm-180o
EXPNO 2
PROCNO 1

F2 - Acquisition Parameters
Date_ 20240606
Time 19.26 h
INSTRUM AvanceNeo 400MHz
PROBHD Z163739_0629 (
PULPROG zgpg30
TD 65536
SOLVENT CDCl3
NS 200
DS 4
SWH 23809.523 Hz
FIDRES 0.726609 Hz
AQ 1.3762560 sec
RG 10
DW 21.000 usec
DE 6.50 usec
TE 296.9 K
D1 2.00000000 sec
D11 0.03000000 sec
TD0 1
SFO1 100.6354036 MHz
NUC1 13C
P0 2.67 usec
P1 8.00 usec
PLW1 85.25399780 W
SFO2 400.1816007 MHz
NUC2 1H
CPDPRG[2 waltz65
PCPD2 90.00 usec
PLW2 21.26700020 W
PLW12 0.16802999 W
PLW13 0.08452000 W

F2 - Processing parameters
SI 32768
SF 100.6253513 MHz
WDW EM
SSB 0
LB 1.00 Hz
GB 0
PC 1.40

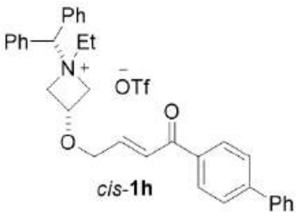

cis-**1h**

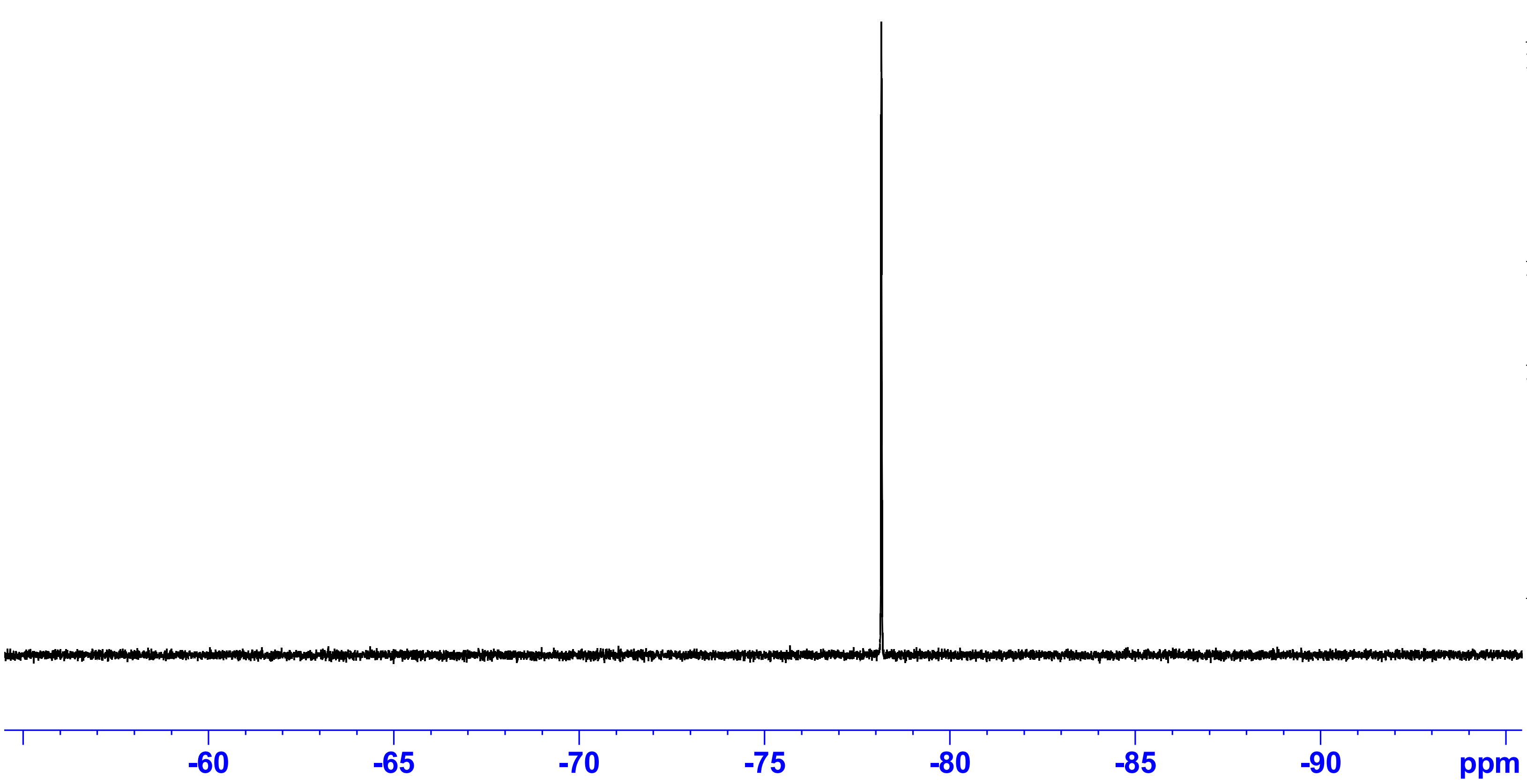

Current Data Parameters
NAME zmh-sm-180o
EXPNO 15
PROCNO 1

F2 - Acquisition Parameters
Date_ 20240605
Time 19.34 h
INSTRUM AvanceNeo 400MHz
PROBHD Z163739_0629 (
PULPROG zgig
TD 131072
SOLVENT CDCl3
NS 16
DS 4
SWH 90909.094 Hz
FIDRES 1.387163 Hz
AQ 0.7208960 sec
RG 101
DW 5.500 usec
DE 6.50 usec
TE 297.0 K
D1 1.00000000 sec
D11 0.03000000 sec
TD0 1
SFO1 376.5077587 MHz
NUC1 19F
P1 12.00 usec
PLW1 33.72800064 W
SFO2 400.1816007 MHz
NUC2 1H
CPDPRG[2 waltz16
PCPD2 90.00 usec
PLW2 21.26700020 W
PLW12 0.16802999 W

F2 - Processing parameters
SI 65536
SF 376.5454132 MHz
WDW EM
SSB 0
LB 0.30 Hz
GB 0
PC 1.00

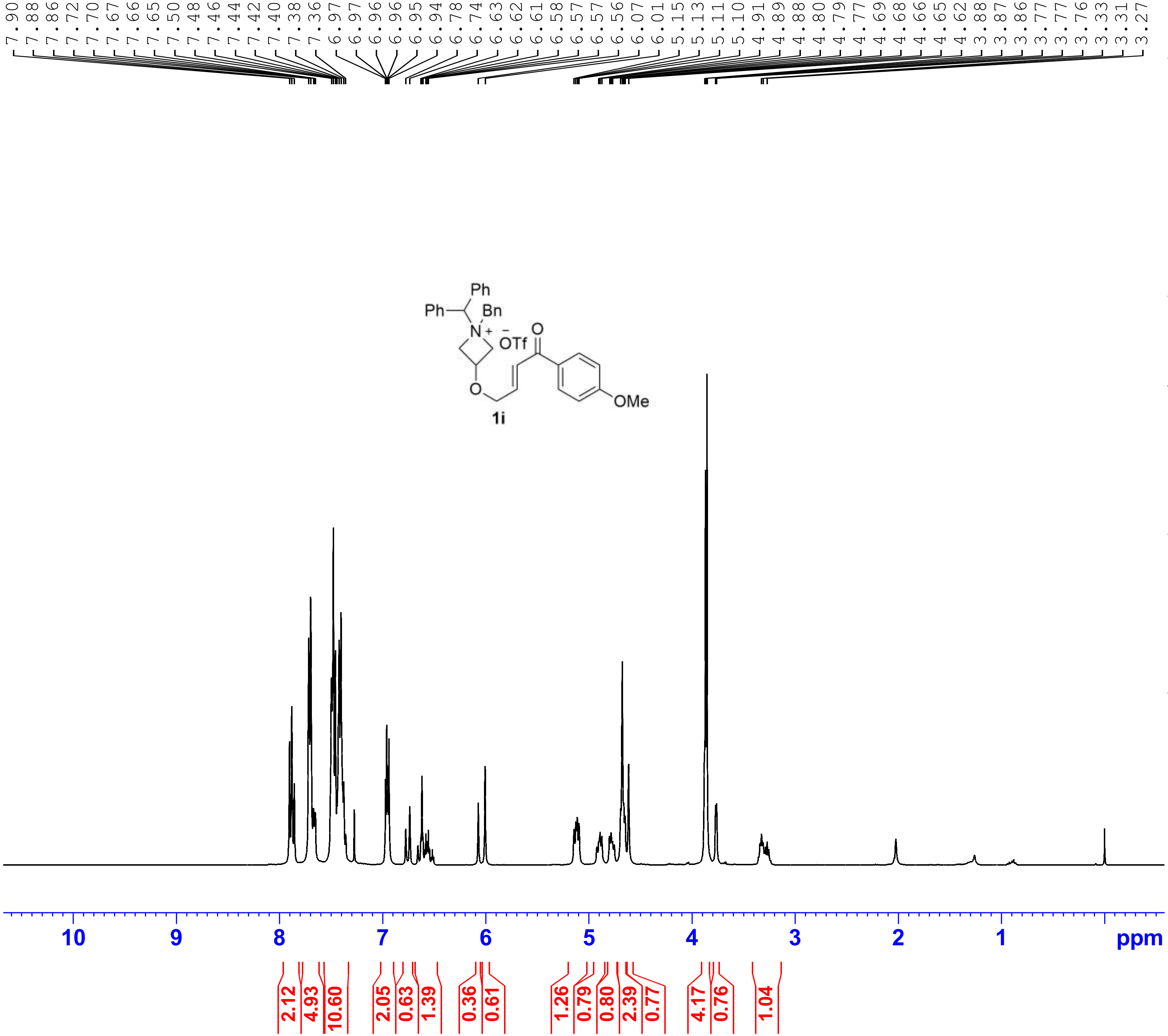

Current Data Parameters
NAME zmh-sm-180b
EXPNO 5
PROCNO 1

F2 - Acquisition Parameters
Date_ 20240606
Time_ 19.31 h
INSTRUM AvanceNeo 400MHz
PROBHD Z163739_0629 (
PULPROG zg30
TD 65536
SOLVENT CDCl3
NS 8
DS 2
SWH 8196.722 Hz
FIDRES 0.250144 Hz
AQ 3.9976959 sec
RG 101
DW 61.000 usec
DE 13.89 usec
TE 296.7 K
D1 1.00000000 sec
TD0 1
SFO1 400.1824711 MHz
NUC1 1H
P0 2.67 usec
P1 8.00 usec
PLW1 21.26700020 W

F2 - Processing parameters
SI 65536
SF 400.1800034 MHz
WDW EM
SSB 0
LB 0.30 Hz
GB 0
PC 1.00

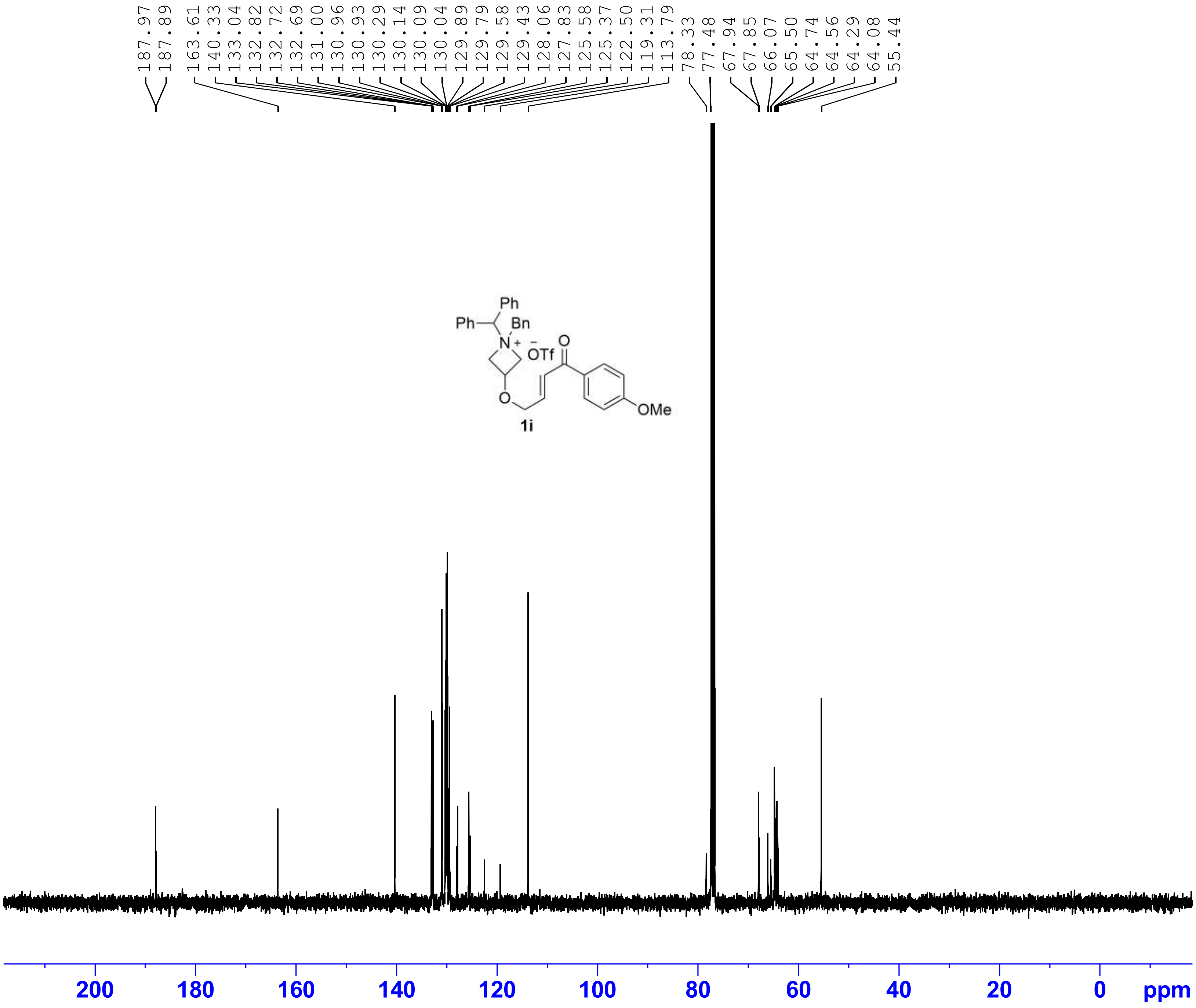

Current Data Parameters
NAME zmh-sm-180b
EXPNO 6
PROCNO 1

F2 - Acquisition Parameters
Date_ 20240606
Time 19.44 h
INSTRUM AvanceNeo 400MHz
PROBHD Z163739_0629 (
PULPROG zgpg30
TD 65536
SOLVENT CDCl3
NS 200
DS 4
SWH 23809.523 Hz
FIDRES 0.726609 Hz
AQ 1.3762560 sec
RG 10
DW 21.000 usec
DE 6.50 usec
TE 297.5 K
D1 2.00000000 sec
D11 0.03000000 sec
TD0 1
SFO1 100.6354036 MHz
NUC1 13C
P0 2.67 usec
P1 8.00 usec
PLW1 85.25399780 W
SFO2 400.1816007 MHz
NUC2 1H
CPDPRG[2 waltz65
PCPD2 90.00 usec
PLW2 21.26700020 W
PLW12 0.16802999 W
PLW13 0.08452000 W

F2 - Processing parameters
SI 32768
SF 100.6253511 MHz
WDW EM
SSB 0
LB 1.00 Hz
GB 0
PC 1.40

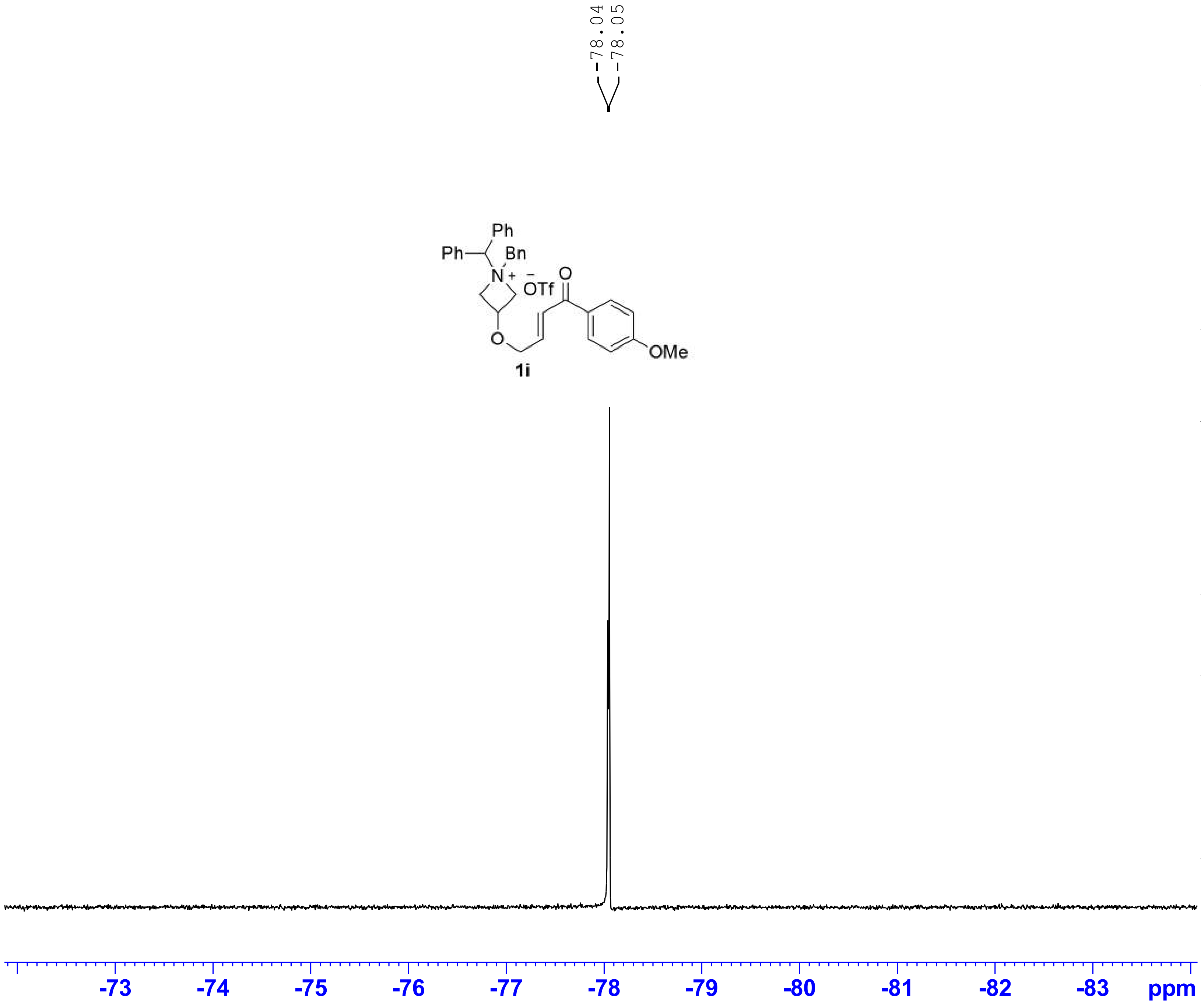

Current Data Parameters
NAME zmh-sm-180b
EXPNO 18
PROCNO 1

F2 - Acquisition Parameters
Date_ 20240605
Time 19.41 h
INSTRUM AvanceNeo 400MHz
PROBHD Z163739_0629 (
PULPROG zgig
TD 131072
SOLVENT CDCl3
NS 16
DS 4
SWH 90909.094 Hz
FIDRES 1.387163 Hz
AQ 0.7208960 sec
RG 101
DW 5.500 usec
DE 6.50 usec
TE 297.0 K
D1 1.00000000 sec
D11 0.03000000 sec
TD0 1
SFO1 376.5077587 MHz
NUC1 19F
P1 12.00 usec
PLW1 33.72800064 W
SFO2 400.1816007 MHz
NUC2 1H
CPDPRG[2 waltz16
PCPD2 90.00 usec
PLW2 21.26700020 W
PLW12 0.16802999 W

F2 - Processing parameters
SI 65536
SF 376.5454132 MHz
WDW EM
SSB 0
LB 0.30 Hz
GB 0
PC 1.00

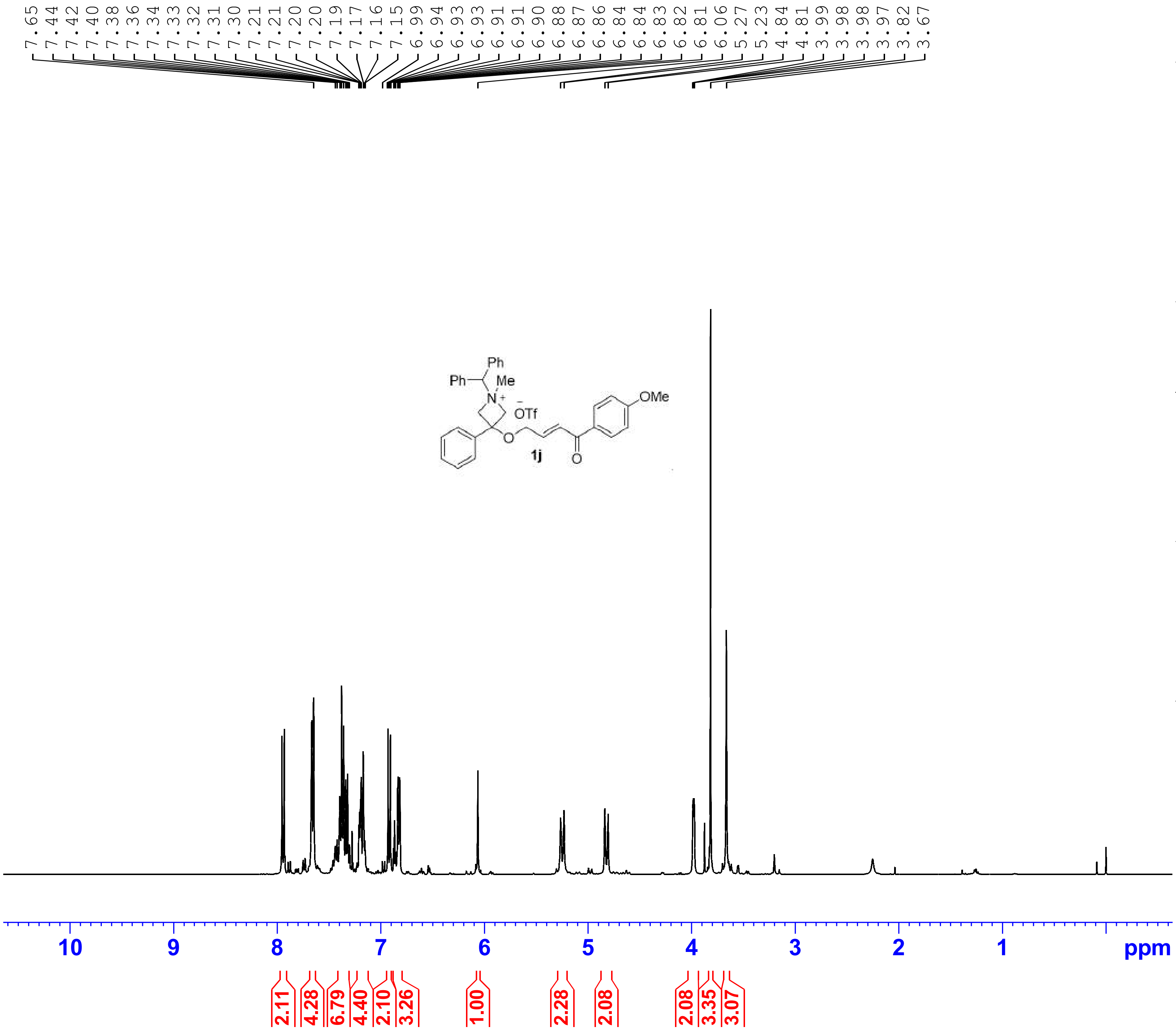

Current Data Parameters
NAME zmh-sm-180d
EXPNO 7
PROCNO 1

F2 - Acquisition Parameters
Date_ 20240607
Time 1.32 h
INSTRUM AvanceNeo 400MHz
PROBHD Z163739_0629 (
PULPROG zg30
TD 65536
SOLVENT CDCl3
NS 8
DS 2
SWH 8196.722 Hz
FIDRES 0.250144 Hz
AQ 3.9976959 sec
RG 45.2
DW 61.000 usec
DE 13.89 usec
TE 297.1 K
D1 1.00000000 sec
TD0 1
SFO1 400.1824711 MHz
NUC1 1H
P0 2.67 usec
P1 8.00 usec
PLW1 21.26700020 W

F2 - Processing parameters
SI 65536
SF 400.1800023 MHz
WDW EM
SSB 0
LB 0.30 Hz
GB 0
PC 1.00

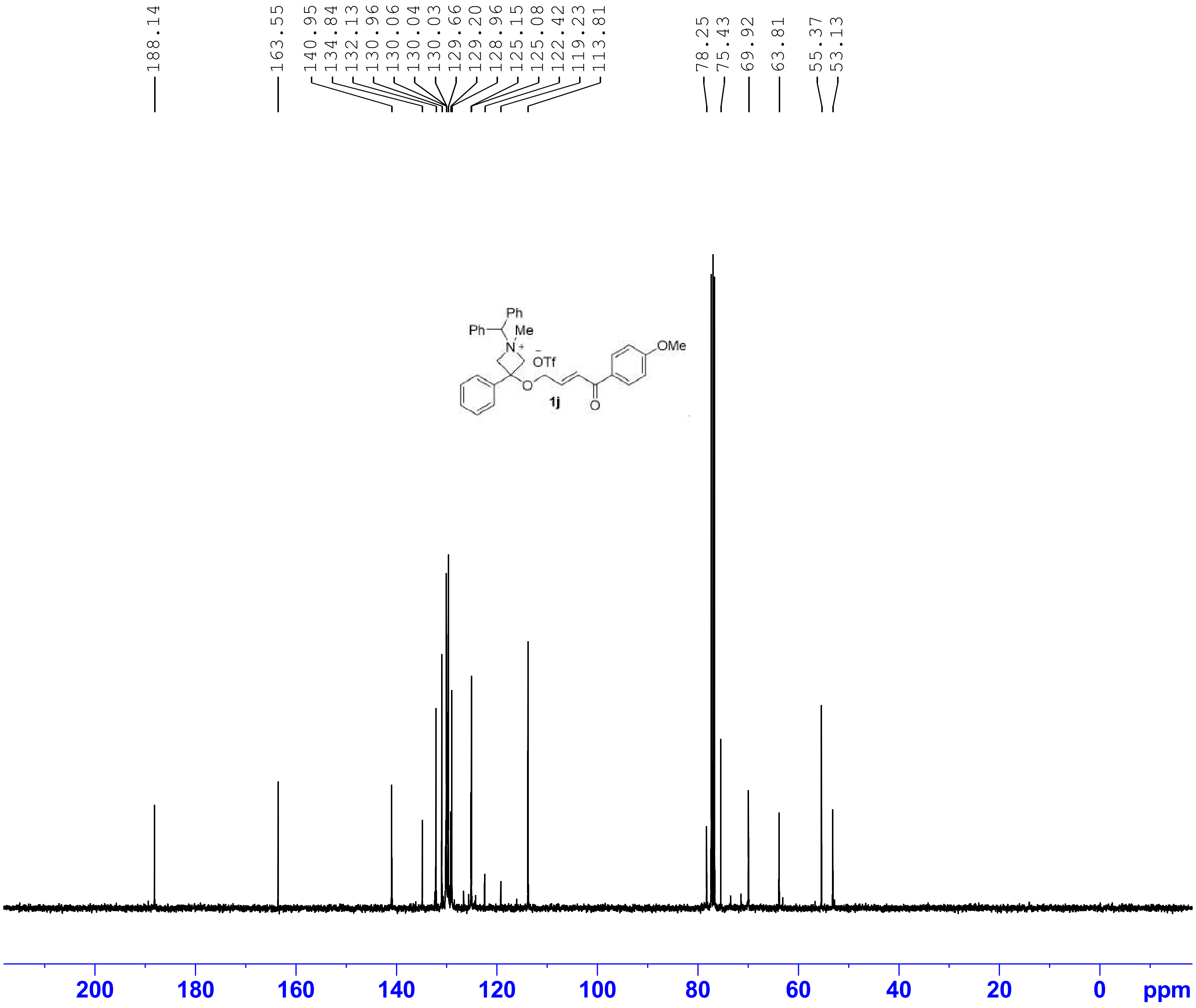

Current Data Parameters
NAME zmh-sm-180d
EXPNO 8
PROCNO 1

F2 - Acquisition Parameters
Date_ 20240607
Time 1.58 h
INSTRUM AvanceNeo 400MHz
PROBHD Z163739_0629 (
PULPROG zgpg30
TD 65536
SOLVENT CDCl3
NS 400
DS 4
SWH 23809.523 Hz
FIDRES 0.726609 Hz
AQ 1.3762560 sec
RG 10
DW 21.000 usec
DE 6.50 usec
TE 297.9 K
D1 2.00000000 sec
D11 0.03000000 sec
TD0 1
SFO1 100.6354036 MHz
NUC1 13C
P0 2.67 usec
P1 8.00 usec
PLW1 85.25399780 W
SFO2 400.1816007 MHz
NUC2 1H
CPDPRG[2 waltz65
PCPD2 90.00 usec
PLW2 21.26700020 W
PLW12 0.16802999 W
PLW13 0.08452000 W

F2 - Processing parameters
SI 32768
SF 100.6253530 MHz
WDW EM
SSB 0
LB 1.00 Hz
GB 0
PC 1.40

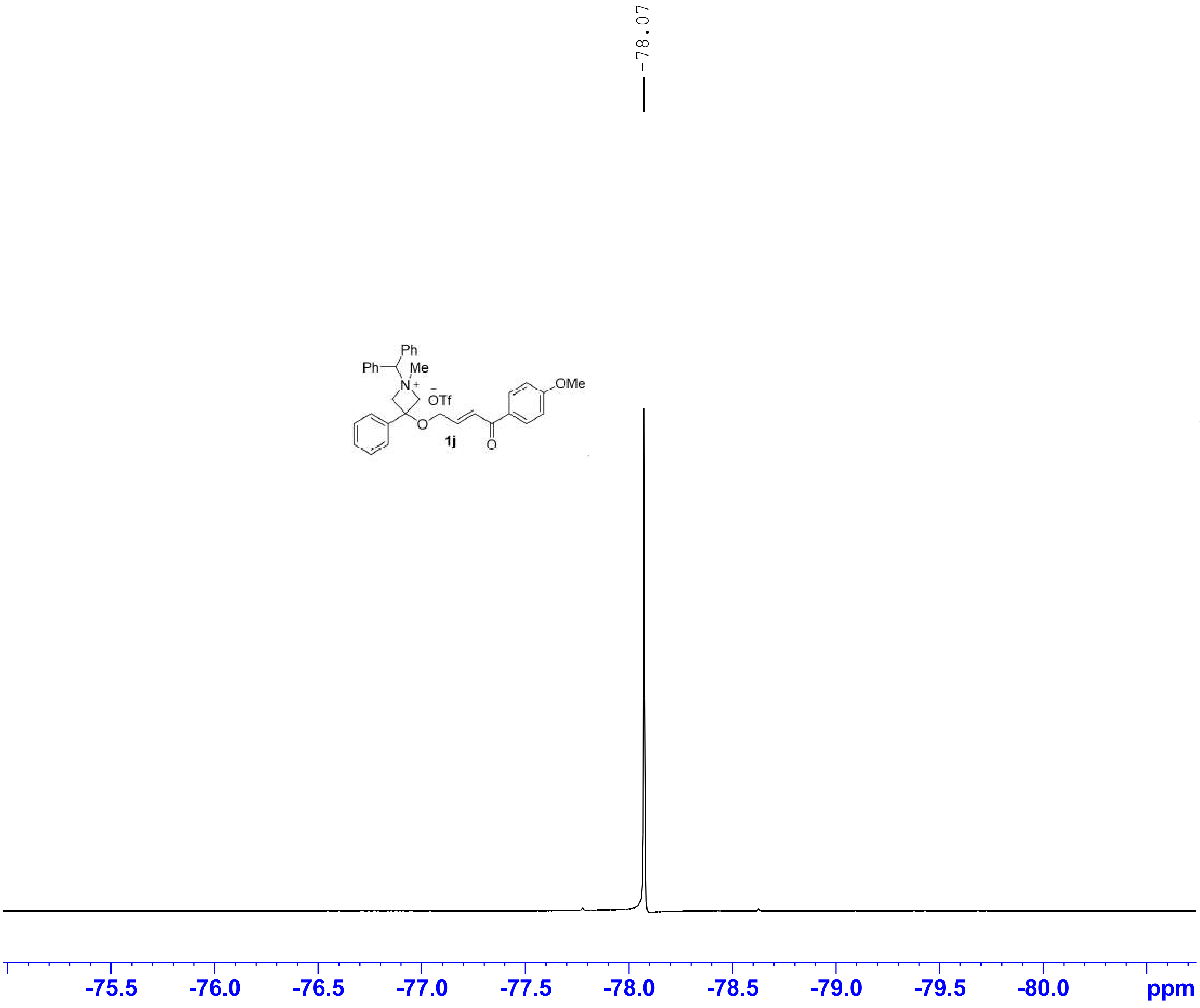

Current Data Parameters
NAME zmh-sm-180d
EXPNO 9
PROCNO 1

F2 - Acquisition Parameters
Date_ 20240607
Time 2.00 h
INSTRUM AvanceNeo 400MHz
PROBHD Z163739_0629 (
PULPROG zgig
TD 131072
SOLVENT CDCl3
NS 8
DS 4
SWH 90909.094 Hz
FIDRES 1.387163 Hz
AQ 0.7208960 sec
RG 101
DW 5.500 usec
DE 6.50 usec
TE 297.4 K
D1 1.00000000 sec
D11 0.03000000 sec
TD0 1
SFO1 376.5077587 MHz
NUC1 19F
P1 12.00 usec
PLW1 33.72800064 W
SFO2 400.1816007 MHz
NUC2 1H
CPDPRG[2 waltz16
PCPD2 90.00 usec
PLW2 21.26700020 W
PLW12 0.16802999 W

F2 - Processing parameters
SI 65536
SF 376.5454132 MHz
WDW EM
SSB 0
LB 0.30 Hz
GB 0
PC 1.00

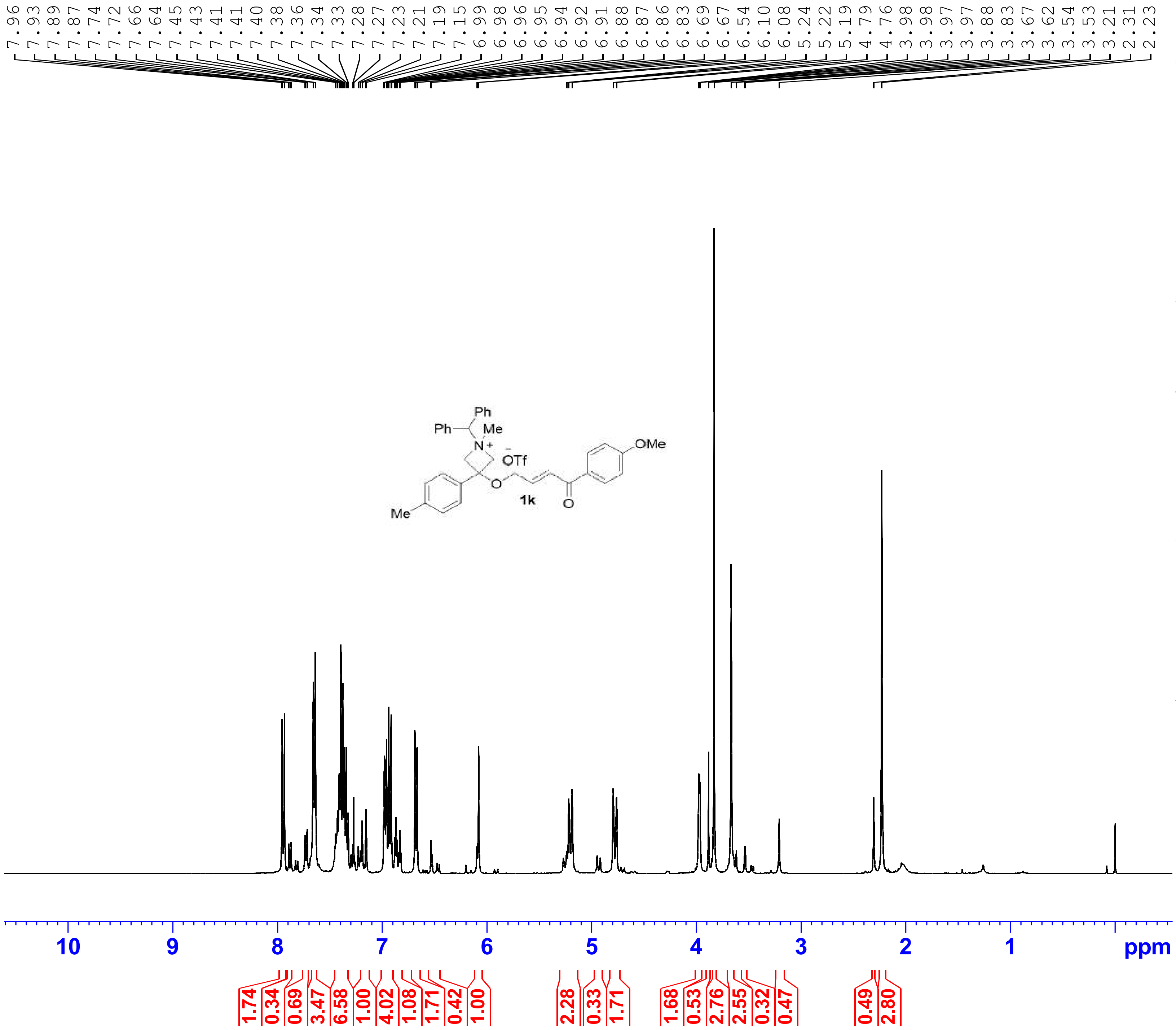

Current Data Parameters
NAME zmh-sm-180h
EXPNO 13
PROCNO 1

F2 - Acquisition Parameters
Date_ 20240606
Time 20.23 h
INSTRUM AvanceNeo 400MHz
PROBHD Z163739_0629 (
PULPROG zg30
TD 65536
SOLVENT CDCl3
NS 8
DS 2
SWH 8196.722 Hz
FIDRES 0.250144 Hz
AQ 3.9976959 sec
RG 101
DW 61.000 usec
DE 13.89 usec
TE 296.8 K
D1 1.00000000 sec
TD0 1
SFO1 400.1824711 MHz
NUC1 1H
P0 2.67 usec
P1 8.00 usec
PLW1 21.26700020 W

F2 - Processing parameters
SI 65536
SF 400.1800046 MHz
WDW EM
SSB 0
LB 0.30 Hz
GB 0
PC 1.00

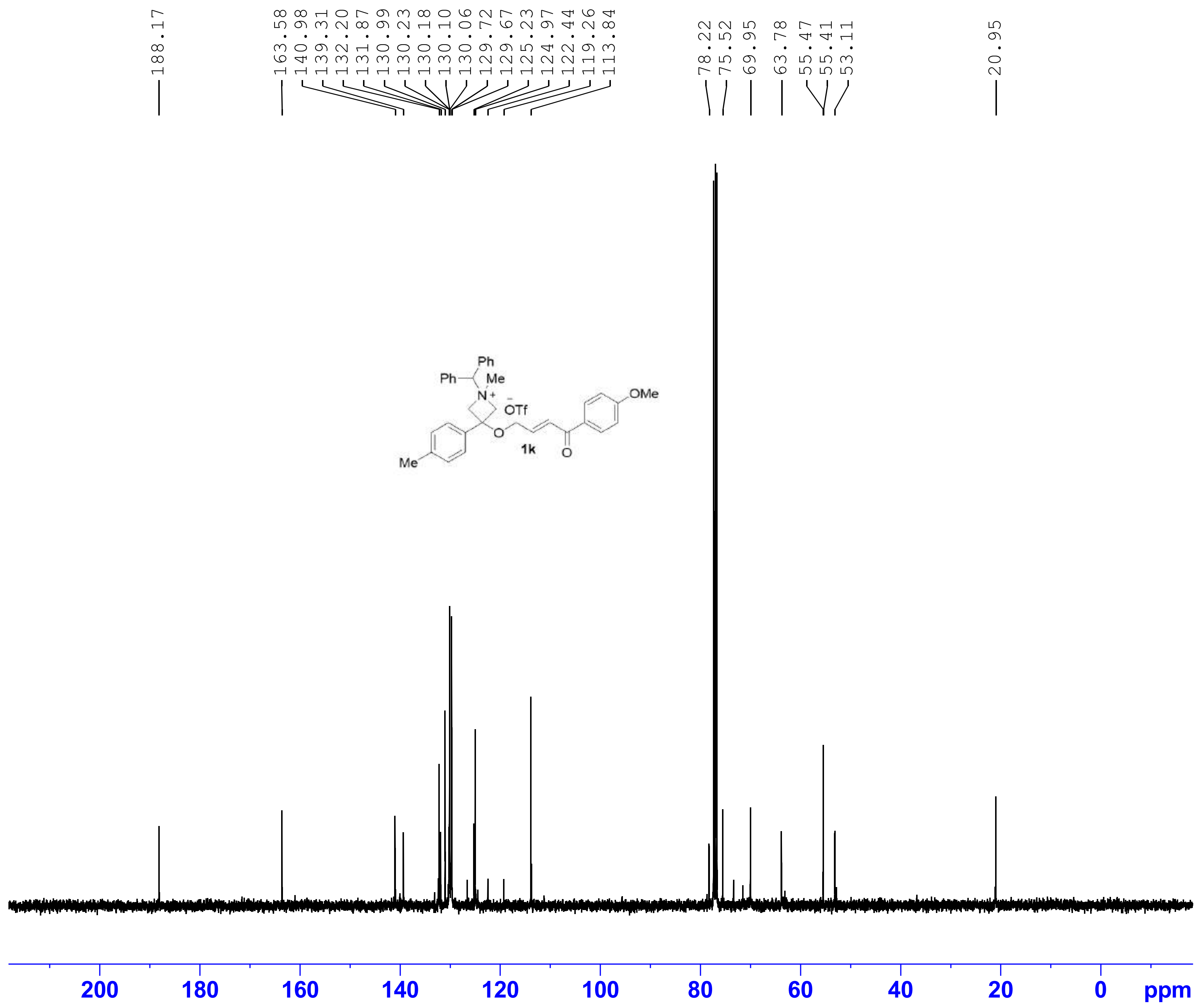

Current Data Parameters
NAME zmh-sm-180h
EXPNO 14
PROCNO 1

F2 - Acquisition Parameters
Date_ 20240606
Time 20.35 h
INSTRUM AvanceNeo 400MHz
PROBHD Z163739_0629 (
PULPROG zgpg30
TD 65536
SOLVENT CDCl3
NS 200
DS 4
SWH 23809.523 Hz
FIDRES 0.726609 Hz
AQ 1.3762560 sec
RG 22.6
DW 21.000 usec
DE 6.50 usec
TE 297.5 K
D1 2.00000000 sec
D11 0.03000000 sec
TD0 1
SFO1 100.6354036 MHz
NUC1 13C
P0 2.67 usec
P1 8.00 usec
PLW1 85.25399780 W
SFO2 400.1816007 MHz
NUC2 1H
CPDPRG[2 waltz65
PCPD2 90.00 usec
PLW2 21.26700020 W
PLW12 0.16802999 W
PLW13 0.08452000 W

F2 - Processing parameters
SI 32768
SF 100.6253493 MHz
WDW EM
SSB 0
LB 1.00 Hz
GB 0
PC 1.40

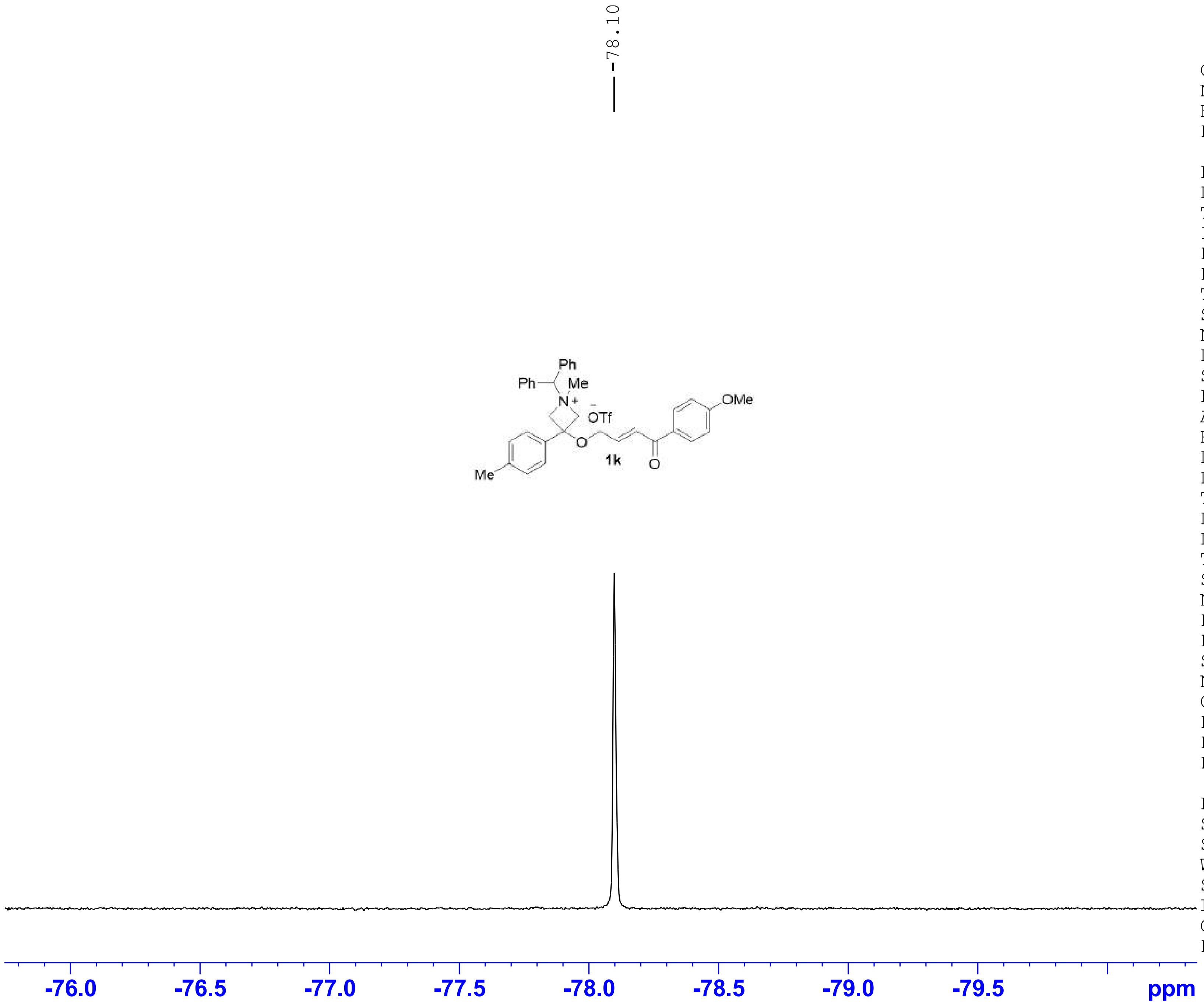

Current Data Parameters
NAME zmh-sm-180h
EXPNO 12
PROCNO 1

F2 - Acquisition Parameters
Date_ 20240605
Time 19.26 h
INSTRUM AvanceNeo 400MHz
PROBHD Z163739_0629 (
PULPROG zgig
TD 131072
SOLVENT CDCl3
NS 16
DS 4
SWH 90909.094 Hz
FIDRES 1.387163 Hz
AQ 0.7208960 sec
RG 101
DW 5.500 usec
DE 6.50 usec
TE 297.0 K
D1 1.00000000 sec
D11 0.03000000 sec
TD0 1
SFO1 376.5077587 MHz
NUC1 19F
P1 12.00 usec
PLW1 33.72800064 W
SFO2 400.1816007 MHz
NUC2 1H
CPDPRG[2 waltz16
PCPD2 90.00 usec
PLW2 21.26700020 W
PLW12 0.16802999 W

F2 - Processing parameters
SI 65536
SF 376.5454132 MHz
WDW EM
SSB 0
LB 0.30 Hz
GB 0
PC 1.00

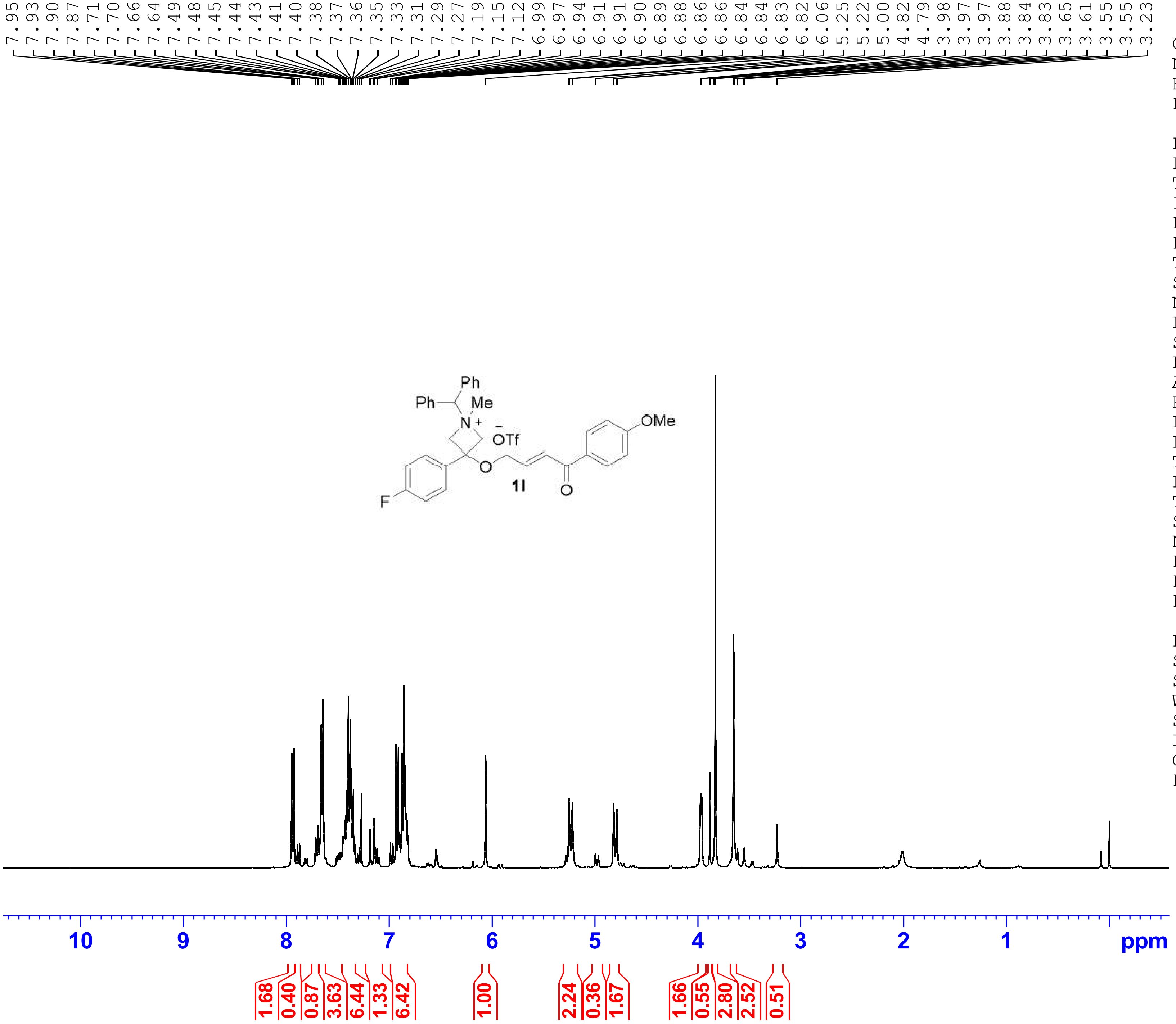

Current Data Parameters
NAME zmh-sm-180e
EXPNO 13
PROCNO 1

F2 - Acquisition Parameters
Date_ 20240606
Time 20.39 h
INSTRUM AvanceNeo 400MHz
PROBHD Z163739_0629 (
PULPROG zg30
TD 65536
SOLVENT CDCl3
NS 8
DS 2
SWH 8196.722 Hz
FIDRES 0.250144 Hz
AQ 3.9976959 sec
RG 101
DW 61.000 usec
DE 13.89 usec
TE 296.8 K
D1 1.00000000 sec
TD0 1
SFO1 400.1824711 MHz
NUC1 1H
P0 2.67 usec
P1 8.00 usec
PLW1 21.26700020 W

F2 - Processing parameters
SI 65536
SF 400.1800052 MHz
WDW EM
SSB 0
LB 0.30 Hz
GB 0
PC 1.00

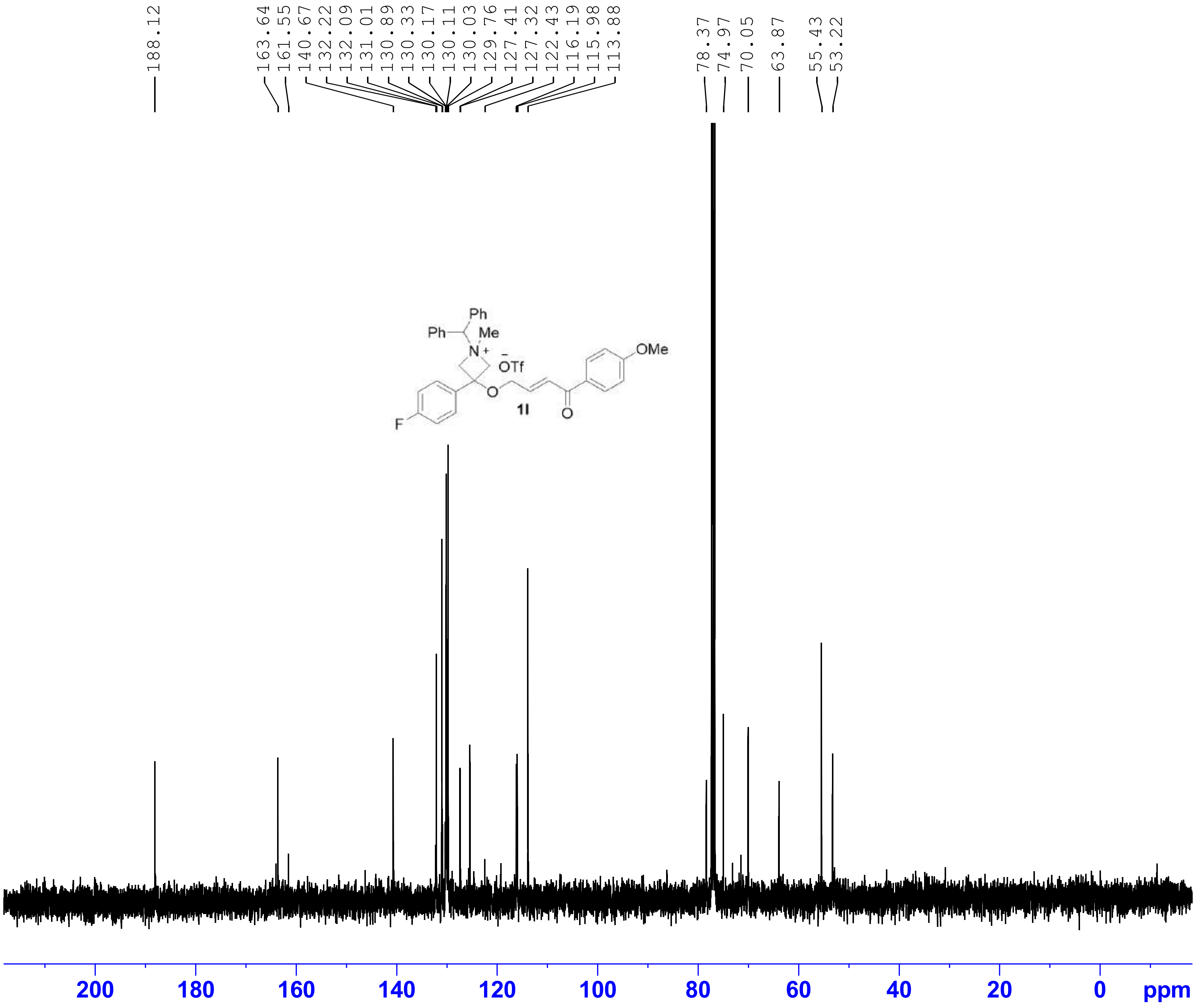

Current Data Parameters
NAME zmh-sm-180e
EXPNO 14
PROCNO 1

F2 - Acquisition Parameters
Date_ 20240606
Time 20.52 h
INSTRUM AvanceNeo 400MHz
PROBHD Z163739_0629 (
PULPROG zgpg30
TD 65536
SOLVENT CDCl3
NS 200
DS 4
SWH 23809.523 Hz
FIDRES 0.726609 Hz
AQ 1.3762560 sec
RG 10
DW 21.000 usec
DE 6.50 usec
TE 297.3 K
D1 2.00000000 sec
D11 0.03000000 sec
TD0 1
SFO1 100.6354036 MHz
NUC1 13C
P0 2.67 usec
P1 8.00 usec
PLW1 85.25399780 W
SFO2 400.1816007 MHz
NUC2 1H
CPDPRG[2 waltz65
PCPD2 90.00 usec
PLW2 21.26700020 W
PLW12 0.16802999 W
PLW13 0.08452000 W

F2 - Processing parameters
SI 32768
SF 100.6253477 MHz
WDW EM
SSB 0
LB 1.00 Hz
GB 0
PC 1.40

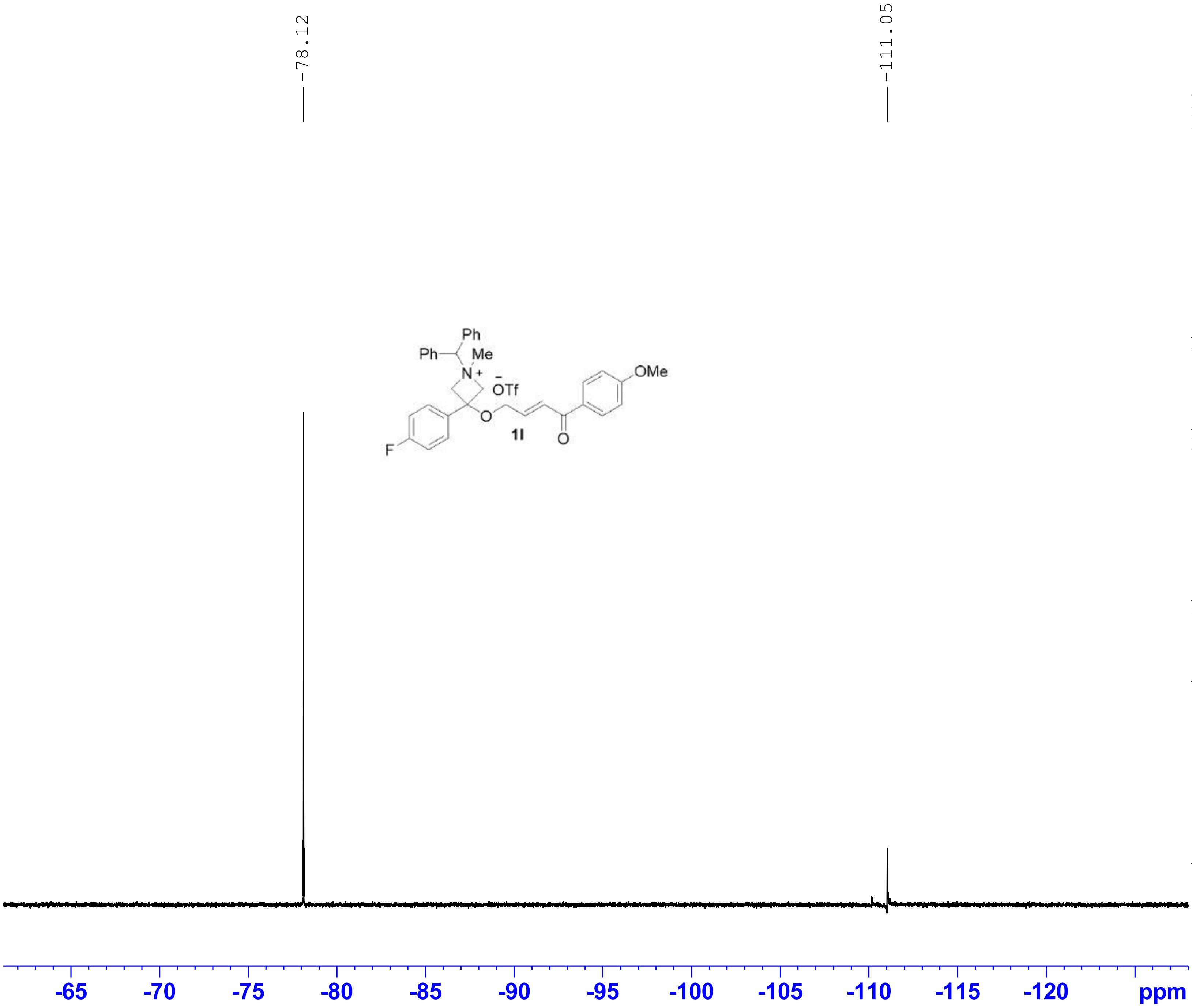

Current Data Parameters
NAME zmh-sm-180e
EXPNO 9
PROCNO 1

F2 - Acquisition Parameters
Date_ 20240605
Time 19.17 h
INSTRUM AvanceNeo 400MHz
PROBHD Z163739_0629 (
PULPROG zgig
TD 131072
SOLVENT CDCl3
NS 16
DS 4
SWH 90909.094 Hz
FIDRES 1.387163 Hz
AQ 0.7208960 sec
RG 101
DW 5.500 usec
DE 6.50 usec
TE 297.0 K
D1 1.00000000 sec
D11 0.03000000 sec
TD0 1
SFO1 376.5077587 MHz
NUC1 19F
P1 12.00 usec
PLW1 33.72800064 W
SFO2 400.1816007 MHz
NUC2 1H
CPDPRG[2 waltz16
PCPD2 90.00 usec
PLW2 21.26700020 W
PLW12 0.16802999 W

F2 - Processing parameters
SI 65536
SF 376.5454132 MHz
WDW EM
SSB 0
LB 0.30 Hz
GB 0
PC 1.00

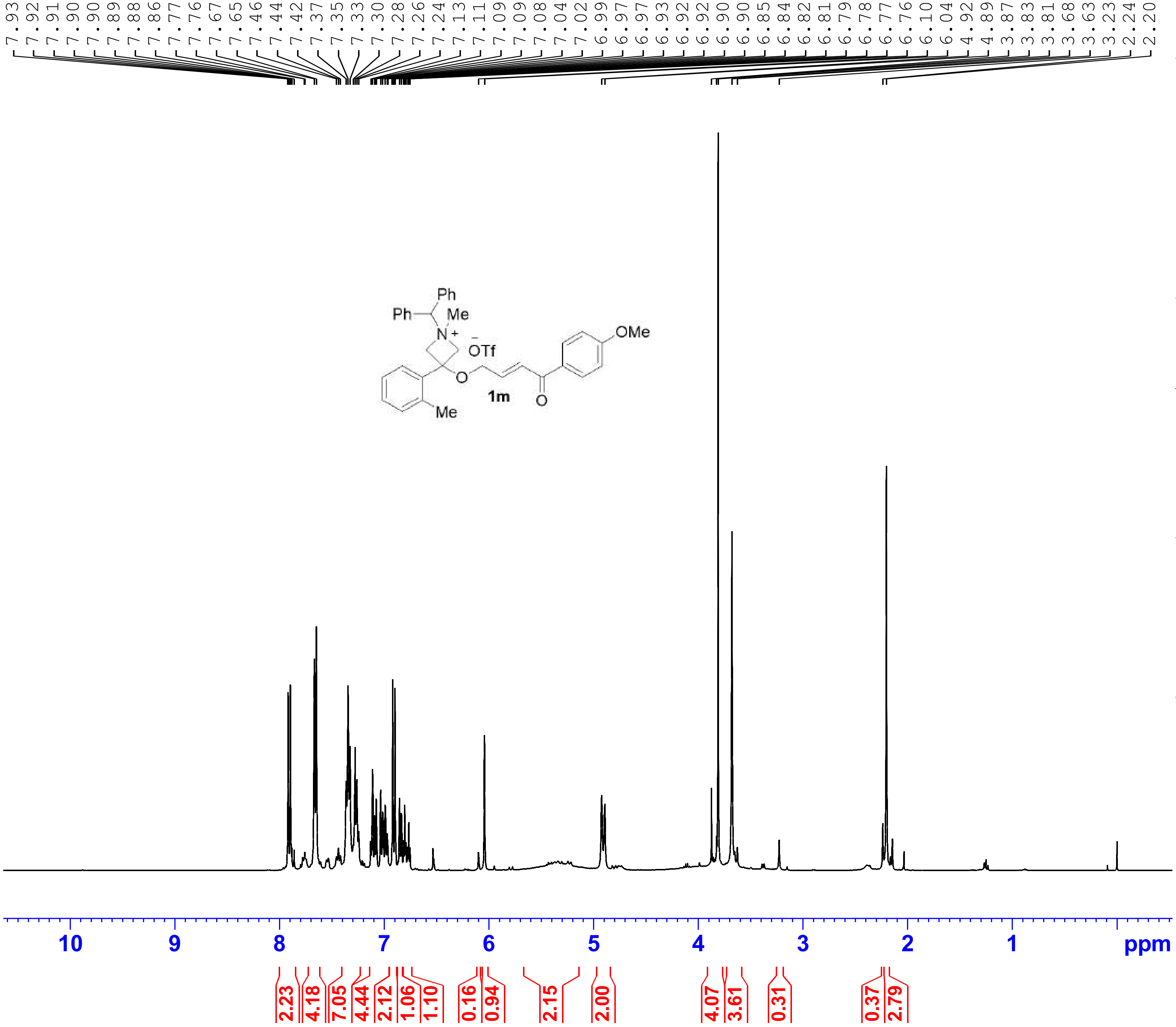

Current Data Parameters
NAME zmh-sm-180f-xia
EXPNO 19
PROCNO 1

F2 - Acquisition Parameters
Date_ 20240607
Time 0.19 h
INSTRUM AvanceNeo 400MHz
PROBHD Z163739_0629 (
PULPROG zg30
TD 65536
SOLVENT CDCl3
NS 8
DS 2
SWH 8196.722 Hz
FIDRES 0.250144 Hz
AQ 3.9976959 sec
RG 45.2
DW 61.000 usec
DE 13.89 usec
TE 297.1 K
D1 1.00000000 sec
TD0 1
SFO1 400.1824711 MHz
NUC1 1H
P0 2.67 usec
P1 8.00 usec
PLW1 21.26700020 W

F2 - Processing parameters
SI 65536
SF 400.1800015 MHz
WDW EM
SSB 0
LB 0.30 Hz
GB 0
PC 1.00

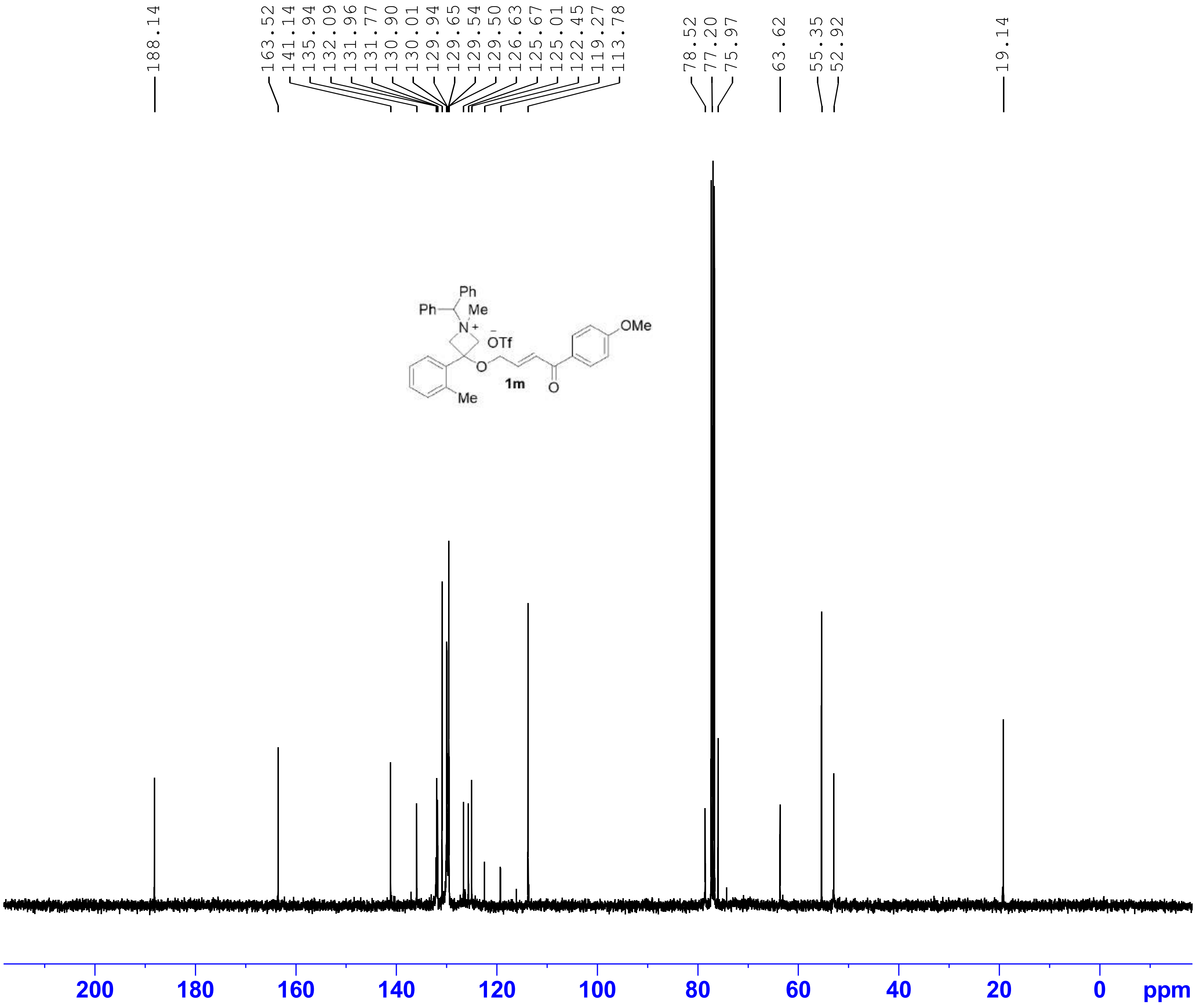

Current Data Parameters
NAME zmh-sm-180f-xia
EXPNO 20
PROCNO 1

F2 - Acquisition Parameters
Date_ 20240607
Time 0.32 h
INSTRUM AvanceNeo 400MHz
PROBHD Z163739_0629 (
PULPROG zgpg30
TD 65536
SOLVENT CDCl3
NS 200
DS 4
SWH 23809.523 Hz
FIDRES 0.726609 Hz
AQ 1.3762560 sec
RG 22.6
DW 21.000 usec
DE 6.50 usec
TE 297.8 K
D1 2.00000000 sec
D11 0.03000000 sec
TD0 1
SFO1 100.6354036 MHz
NUC1 13C
P0 2.67 usec
P1 8.00 usec
PLW1 85.25399780 W
SFO2 400.1816007 MHz
NUC2 1H
CPDPRG[2 waltz65
PCPD2 90.00 usec
PLW2 21.26700020 W
PLW12 0.16802999 W
PLW13 0.08452000 W

F2 - Processing parameters
SI 32768
SF 100.6253542 MHz
WDW EM
SSB 0
LB 1.00 Hz
GB 0
PC 1.40

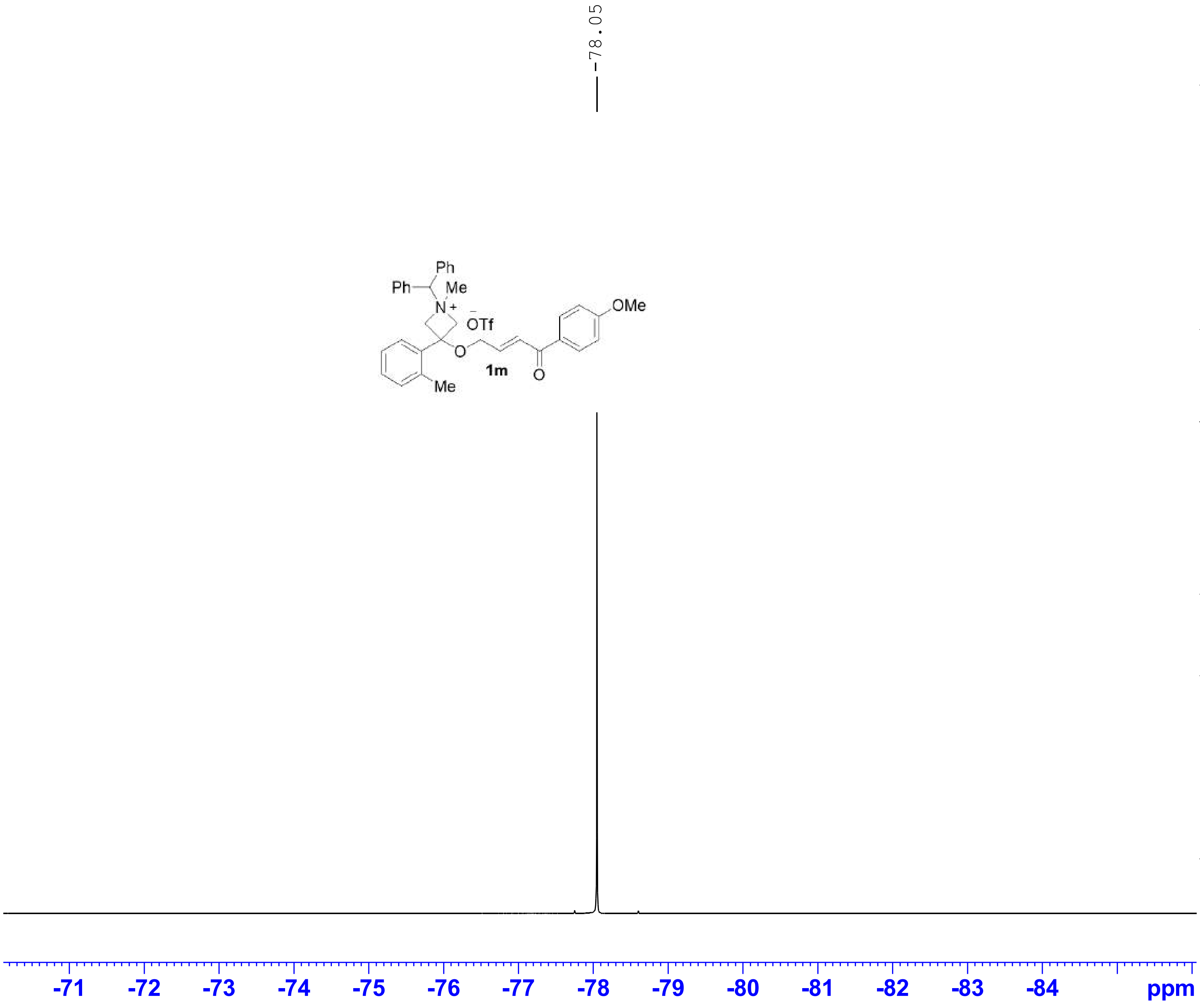

Current Data Parameters

NAME	zmh-sm-180f-xia
EXPNO	21
PROCNO	1

F2 - Acquisition Parameters

Date_	20240607
Time	0.35 h
INSTRUM	AvanceNeo 400MHz
PROBHD	Z163739_0629 (
PULPROG	zgig
TD	131072
SOLVENT	CDCl3
NS	8
DS	4
SWH	90909.094 Hz
FIDRES	1.387163 Hz
AQ	0.7208960 sec
RG	101
DW	5.500 usec
DE	6.50 usec
TE	297.4 K
D1	1.00000000 sec
D11	0.03000000 sec
TD0	1
SFO1	376.5077587 MHz
NUC1	19F
P1	12.00 usec
PLW1	33.72800064 W
SFO2	400.1816007 MHz
NUC2	1H
CPDPRG[2	waltz16
PCPD2	90.00 usec
PLW2	21.26700020 W
PLW12	0.16802999 W

F2 - Processing parameters

SI	65536
SF	376.5454132 MHz
WDW	EM
SSB	0
LB	0.30 Hz
GB	0
PC	1.00

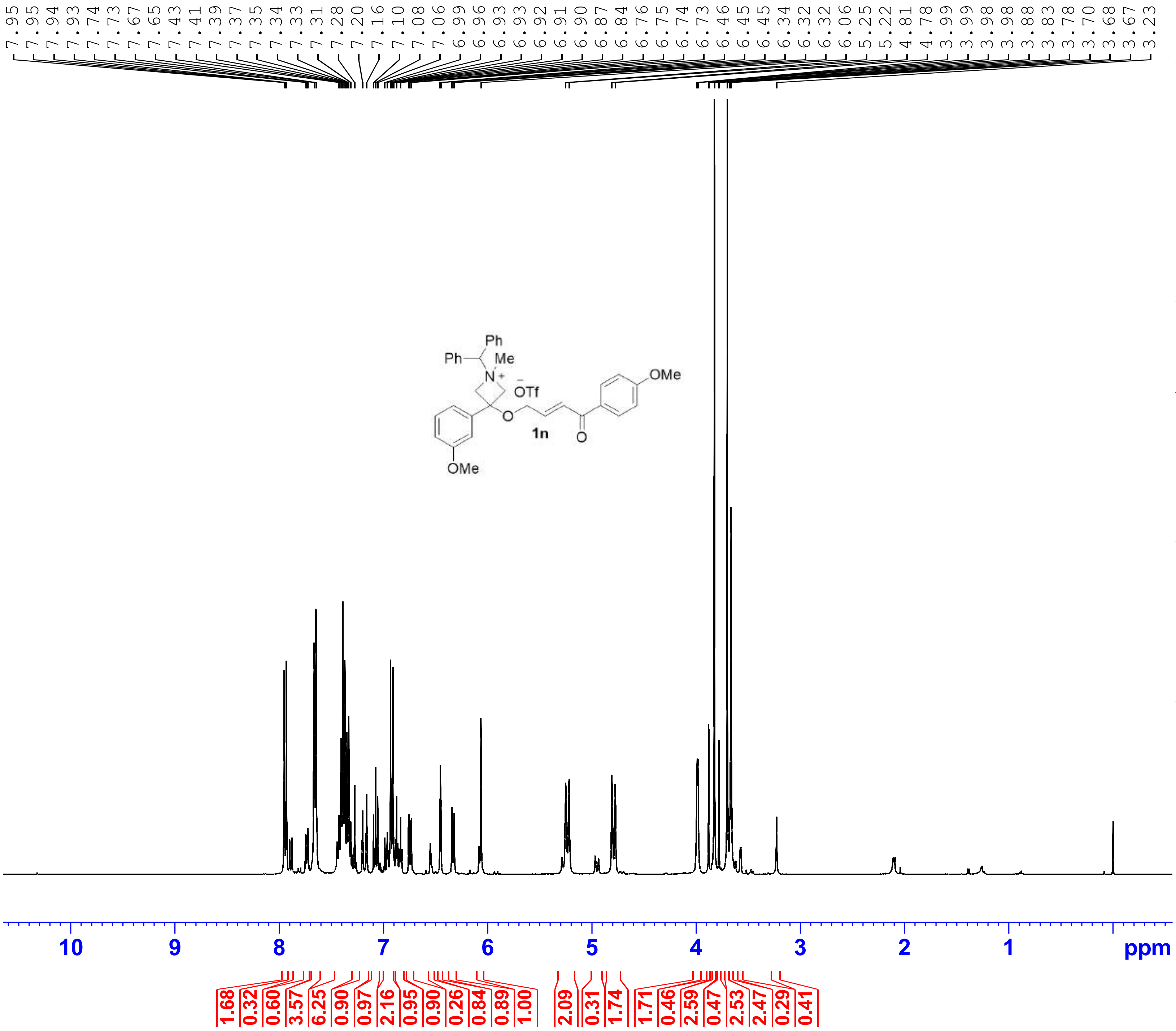

Current Data Parameters
NAME zmh-sm-180g
EXPNO 9
PROCNO 1

F2 - Acquisition Parameters
Date_ 20240606
Time 20.05 h
INSTRUM AvanceNeo 400MHz
PROBHD Z163739_0629 (
PULPROG zg30
TD 65536
SOLVENT CDCl3
NS 8
DS 2
SWH 8196.722 Hz
FIDRES 0.250144 Hz
AQ 3.9976959 sec
RG 101
DW 61.000 usec
DE 13.89 usec
TE 296.7 K
D1 1.00000000 sec
TD0 1
SFO1 400.1824711 MHz
NUC1 1H
P0 2.67 usec
P1 8.00 usec
PLW1 21.26700020 W

F2 - Processing parameters
SI 65536
SF 400.1800032 MHz
WDW EM
SSB 0
LB 0.30 Hz
GB 0
PC 1.00

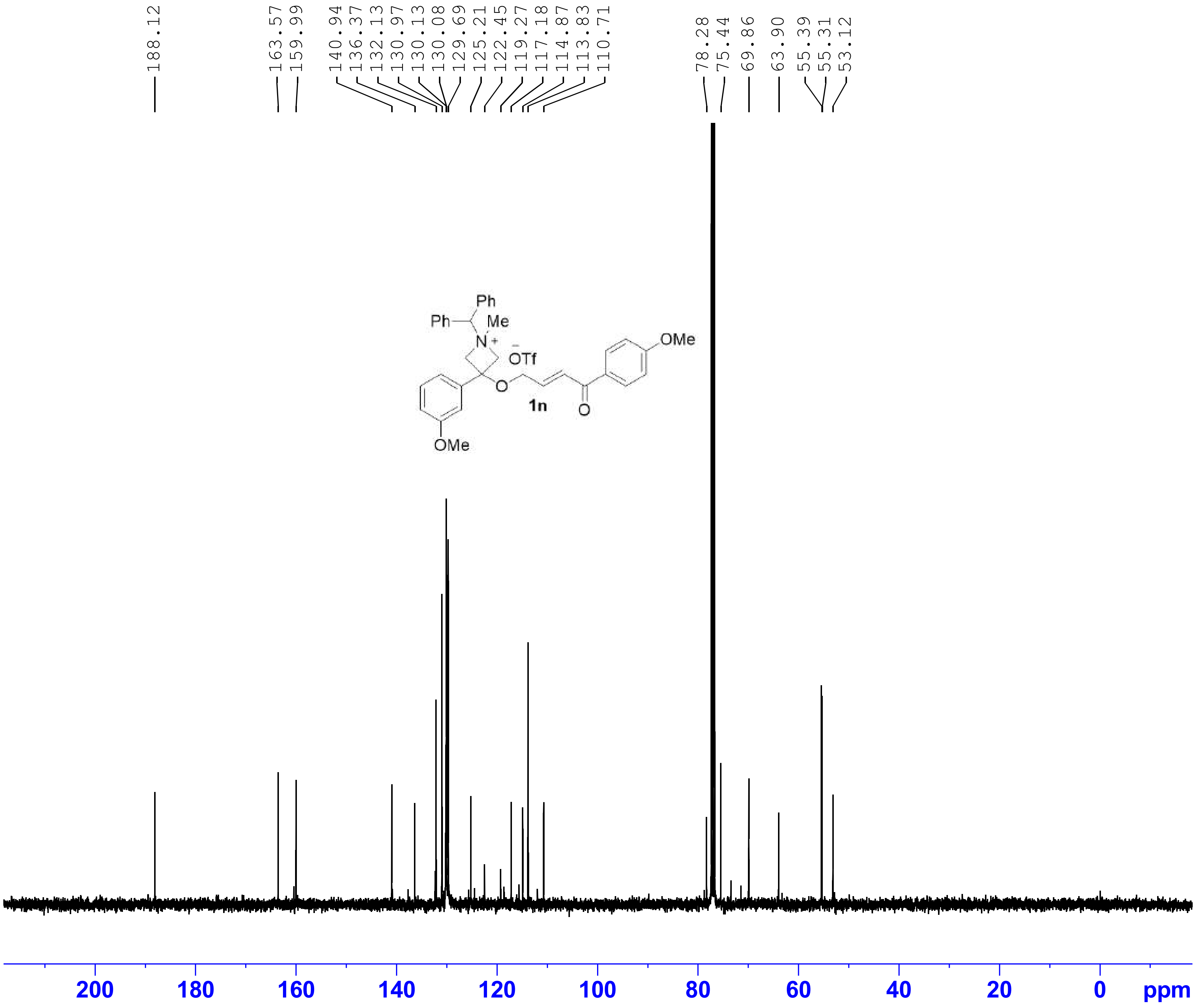

Current Data Parameters
NAME zmh-sm-180g
EXPNO 10
PROCNO 1

F2 - Acquisition Parameters
Date_ 20240606
Time 20.18 h
INSTRUM AvanceNeo 400MHz
PROBHD Z163739_0629 (
PULPROG zgpg30
TD 65536
SOLVENT CDCl3
NS 200
DS 4
SWH 23809.523 Hz
FIDRES 0.726609 Hz
AQ 1.3762560 sec
RG 22.6
DW 21.000 usec
DE 6.50 usec
TE 297.5 K
D1 2.00000000 sec
D11 0.03000000 sec
TD0 1
SFO1 100.6354036 MHz
NUC1 13C
P0 2.67 usec
P1 8.00 usec
PLW1 85.25399780 W
SFO2 400.1816007 MHz
NUC2 1H
CPDPRG[2 waltz65
PCPD2 90.00 usec
PLW2 21.26700020 W
PLW12 0.16802999 W
PLW13 0.08452000 W

F2 - Processing parameters
SI 32768
SF 100.6253507 MHz
WDW EM
SSB 0
LB 1.00 Hz
GB 0
PC 1.40

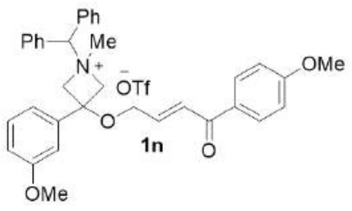

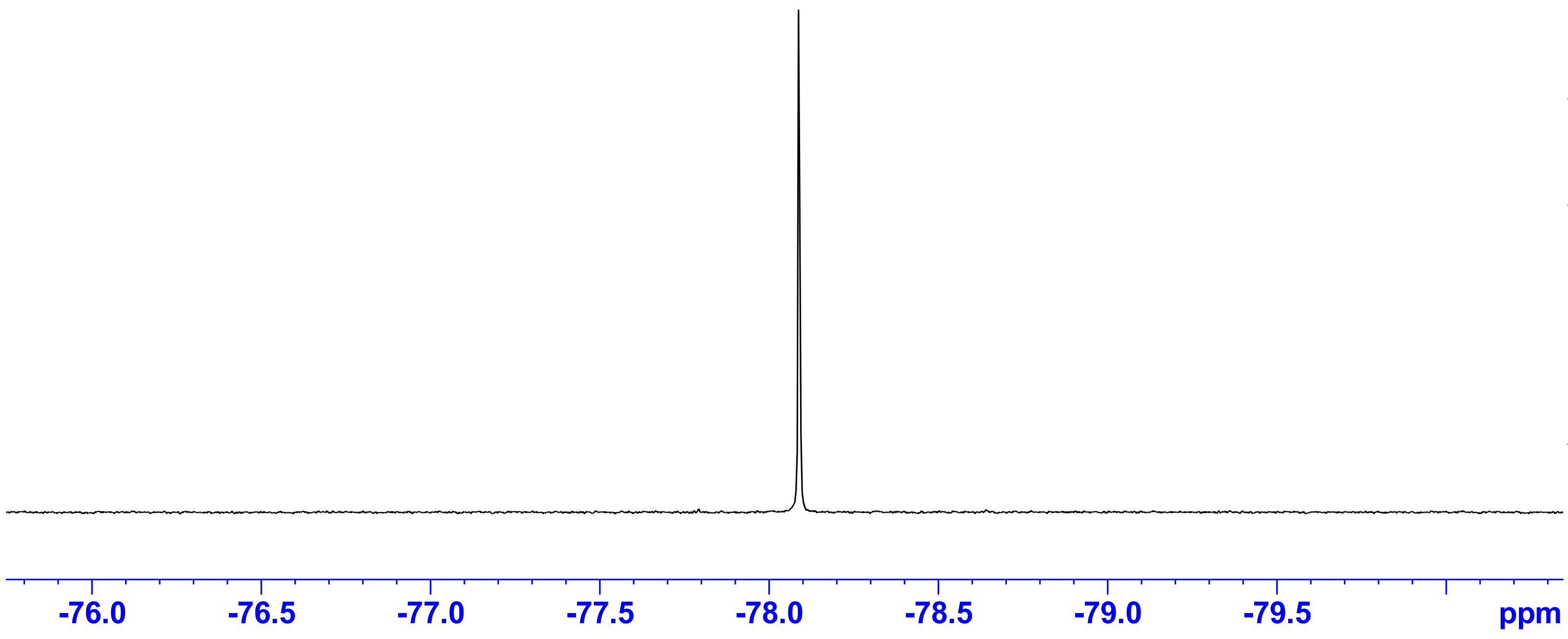

Current Data Parameters
NAME zmh-sm-180g
EXPNO 3
PROCNO 1

F2 - Acquisition Parameters
Date_ 20240605
Time 19.07 h
INSTRUM AvanceNeo 400MHz
PROBHD Z163739_0629 (
PULPROG zgig
TD 131072
SOLVENT CDCl3
NS 16
DS 4
SWH 90909.094 Hz
FIDRES 1.387163 Hz
AQ 0.7208960 sec
RG 101
DW 5.500 usec
DE 6.50 usec
TE 296.9 K
D1 1.00000000 sec
D11 0.03000000 sec
TD0 1
SFO1 376.5077587 MHz
NUC1 19F
P1 12.00 usec
PLW1 33.72800064 W
SFO2 400.1816007 MHz
NUC2 1H
CPDPRG[2 waltz16
PCPD2 90.00 usec
PLW2 21.26700020 W
PLW12 0.16802999 W

F2 - Processing parameters
SI 65536
SF 376.5454132 MHz
WDW EM
SSB 0
LB 0.30 Hz
GB 0
PC 1.00

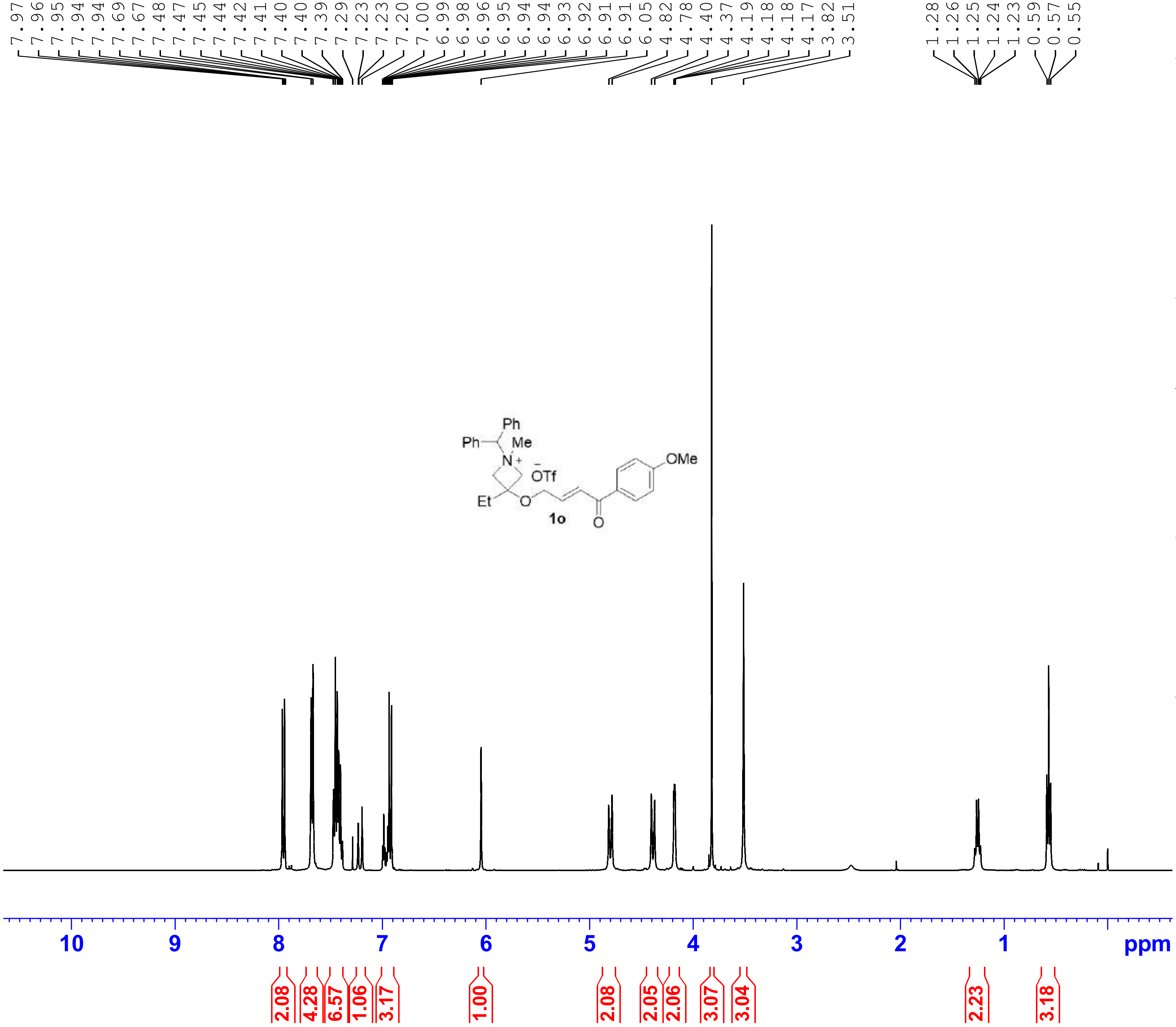

Current Data Parameters

NAME	zmh-sm-180i
EXPNO	1
PROCNO	1

F2 - Acquisition Parameters

Date_	20240606
Time	23.08 h
INSTRUM	AvanceNeo 400MHz
PROBHD	Z163739_0629 (
PULPROG	zg30
TD	65536
SOLVENT	CDCl3
NS	8
DS	2
SWH	8196.722 Hz
FIDRES	0.250144 Hz
AQ	3.9976959 sec
RG	45.2
DW	61.000 usec
DE	13.89 usec
TE	297.2 K
D1	1.00000000 sec
TD0	1
SFO1	400.1824711 MHz
NUC1	1H
P0	2.67 usec
P1	8.00 usec
PLW1	21.26700020 W

F2 - Processing parameters

SI	65536
SF	400.1799981 MHz
WDW	EM
SSB	0
LB	0.30 Hz
GB	0
PC	1.00

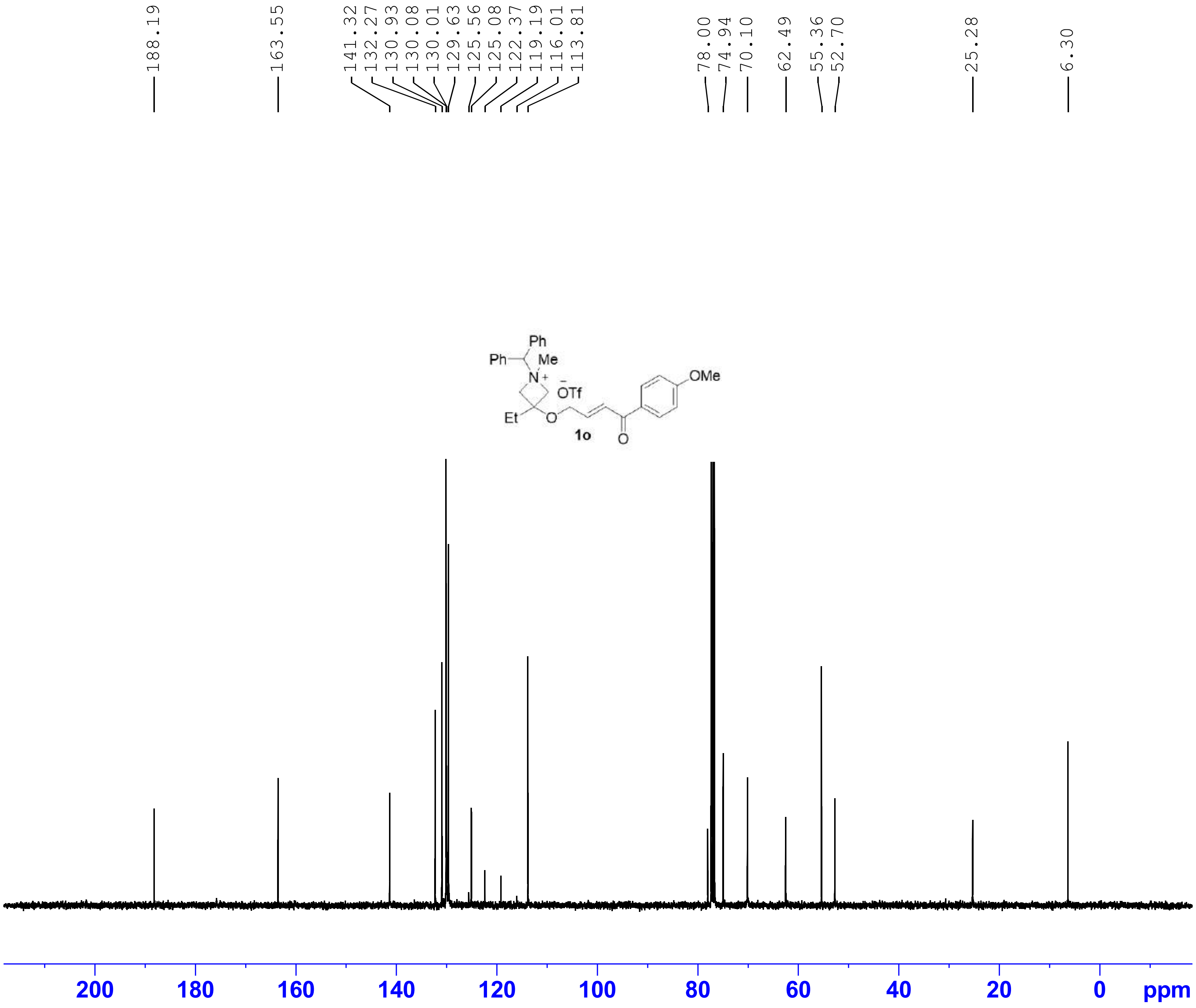


```
Current Data Parameters
NAME          zmh-sm-180i
EXPNO                   2
PROCNO                  1

F2 - Acquisition Parameters
Date_            20240606
Time              23.07 h
INSTRUM   AvanceNeo 400MHz
PROBHD    Z163739_0629 (
PULPROG           zgpg30
TD                 65536
SOLVENT            CDCl3
NS                   200
DS                     4
SWH           23809.523 Hz
FIDRES         0.726609 Hz
AQ            1.3762560 sec
RG                    10
DW               21.000 usec
DE                 6.50 usec
TE                297.7 K
D1           2.00000000 sec
D11          0.03000000 sec
TD0                    1
SFO1        100.6354036 MHz
NUC1                 13C
P0                 2.67 usec
P1                 8.00 usec
PLW1        85.25399780 W
SFO2        400.1816007 MHz
NUC2                  1H
CPDPRG[2         waltz65
PCPD2             90.00 usec
PLW2        21.26700020 W
PLW12        0.16802999 W
PLW13        0.08452000 W

F2 - Processing parameters
SI                 32768
SF          100.6253542 MHz
WDW                   EM
SSB                    0
LB                  1.00 Hz
GB                     0
PC                  1.40
```

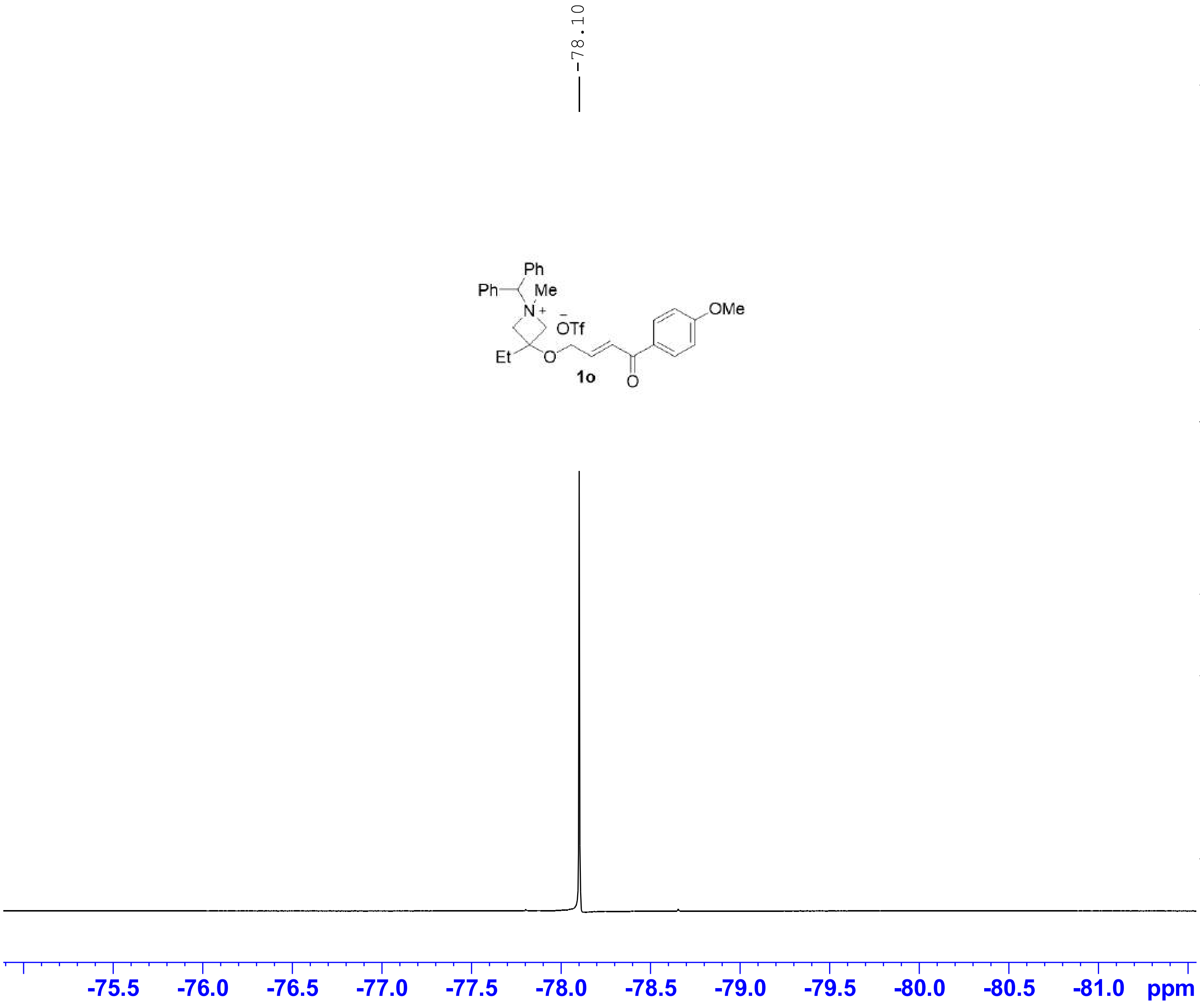

1o

Current Data Parameters
NAME zmh-sm-180i
EXPNO 3
PROCNO 1

F2 - Acquisition Parameters

Date_	20240606	
Time	23.11	h
INSTRUM	AvanceNeo 400MHz	
PROBHD	Z163739_0629 (	
PULPROG	zgig	
TD	131072	
SOLVENT	CDCl3	
NS	8	
DS	4	
SWH	90909.094	Hz
FIDRES	1.387163	Hz
AQ	0.7208960	sec
RG	101	
DW	5.500	usec
DE	6.50	usec
TE	297.1	K
D1	1.00000000	sec
D11	0.03000000	sec
TD0	1	
SFO1	376.5077587	MHz
NUC1	19F	
P1	12.00	usec
PLW1	33.72800064	W
SFO2	400.1816007	MHz
NUC2	1H	
CPDPRG[2	waltz16	
PCPD2	90.00	usec
PLW2	21.26700020	W
PLW12	0.16802999	W

F2 - Processing parameters

SI	65536	
SF	376.5454132	MHz
WDW	EM	
SSB	0	
LB	0.30	Hz
GB	0	
PC	1.00	

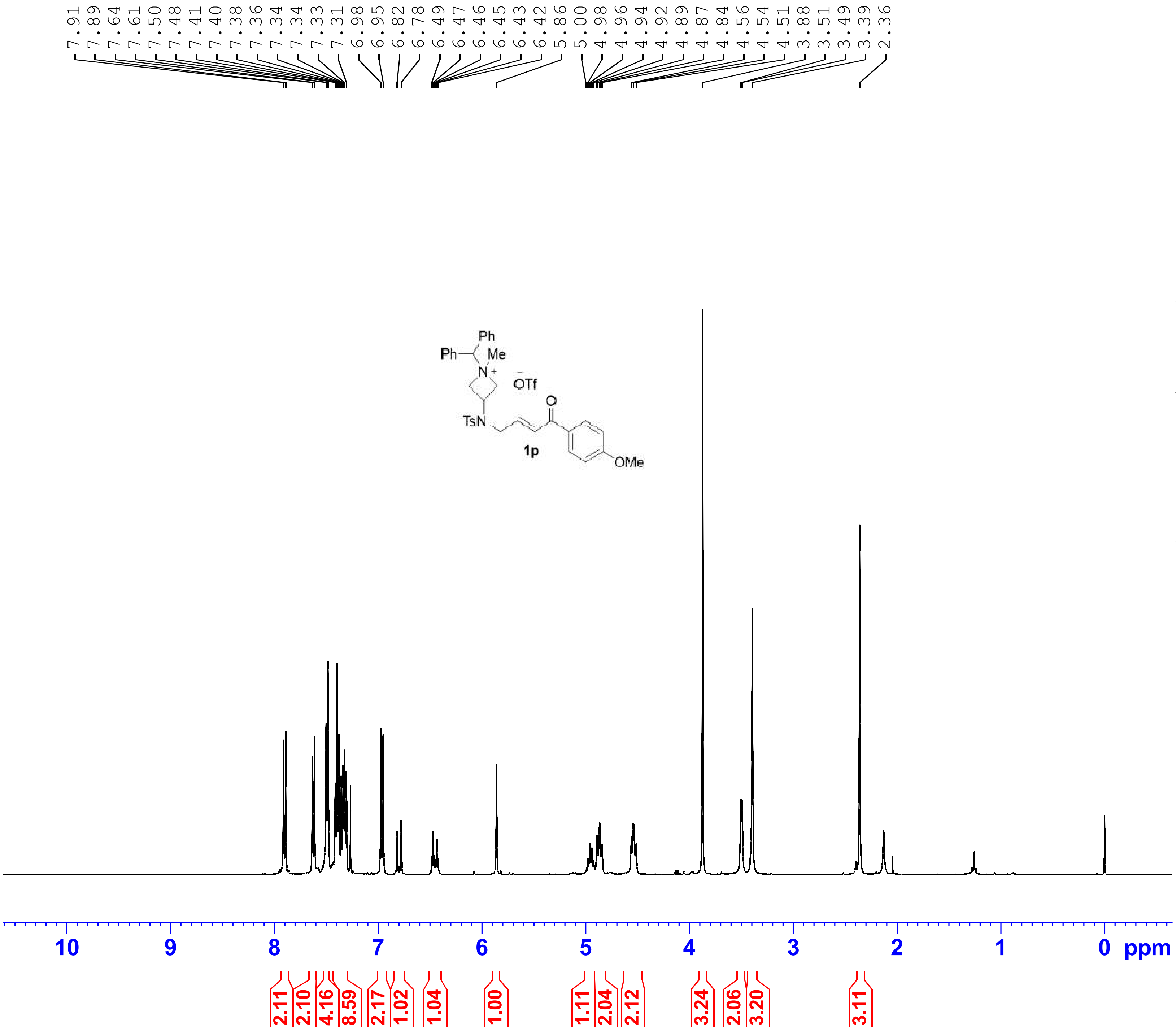

Current Data Parameters

NAME	zmh-sm-180m
EXPNO	7
PROCNO	1

F2 - Acquisition Parameters

Date_	20240606
Time	19.47 h
INSTRUM	AvanceNeo 400MHz
PROBHD	Z163739_0629 (
PULPROG	zg30
TD	65536
SOLVENT	CDCl3
NS	8
DS	2
SWH	8196.722 Hz
FIDRES	0.250144 Hz
AQ	3.9976959 sec
RG	101
DW	61.000 usec
DE	13.89 usec
TE	296.8 K
D1	1.00000000 sec
TD0	1
SFO1	400.1824711 MHz
NUC1	1H
P0	2.67 usec
P1	8.00 usec
PLW1	21.26700020 W

F2 - Processing parameters

SI	65536
SF	400.1800067 MHz
WDW	EM
SSB	0
LB	0.30 Hz
GB	0
PC	1.00

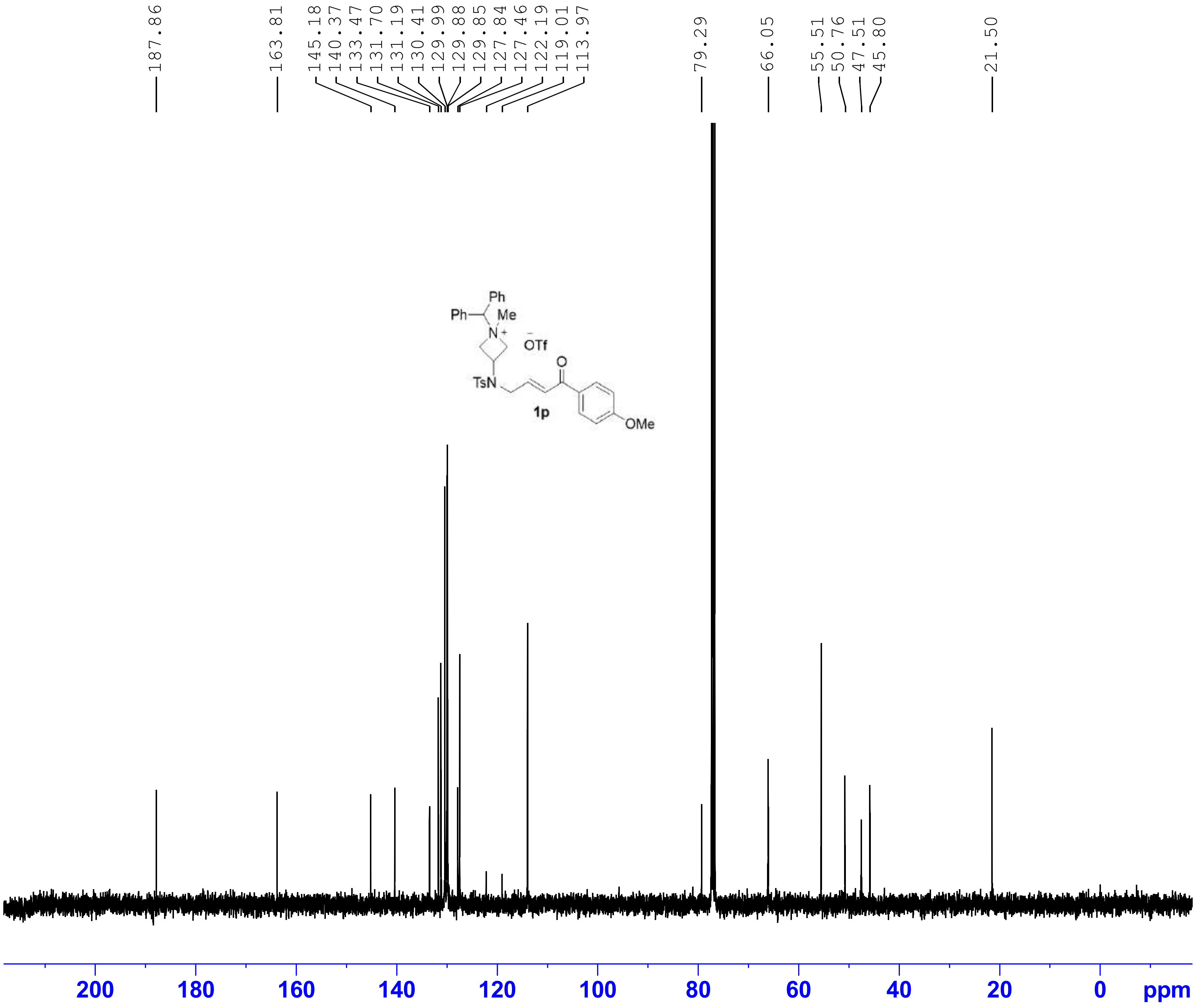


```
Current Data Parameters
NAME          zmh-sm-180m
EXPNO                   8
PROCNO                  1

F2 - Acquisition Parameters
Date_            20240606
Time                20.00 h
INSTRUM   AvanceNeo 400MHz
PROBHD    Z163739_0629 (
PULPROG           zgpg30
TD                 65536
SOLVENT            CDCl3
NS                   200
DS                     4
SWH            23809.523 Hz
FIDRES          0.726609 Hz
AQ             1.3762560 sec
RG                    10
DW                21.000 usec
DE                  6.50 usec
TE                 297.4 K
D1            2.00000000 sec
D11           0.03000000 sec
TD0                    1
SFO1         100.6354036 MHz
NUC1                 13C
P0                  2.67 usec
P1                  8.00 usec
PLW1         85.25399780 W
SFO2         400.1816007 MHz
NUC2                  1H
CPDPRG[2         waltz65
PCPD2              90.00 usec
PLW2         21.26700020 W
PLW12         0.16802999 W
PLW13         0.08452000 W

F2 - Processing parameters
SI                 32768
SF           100.6253469 MHz
WDW                   EM
SSB                    0
LB                  1.00 Hz
GB                     0
PC                  1.40
```

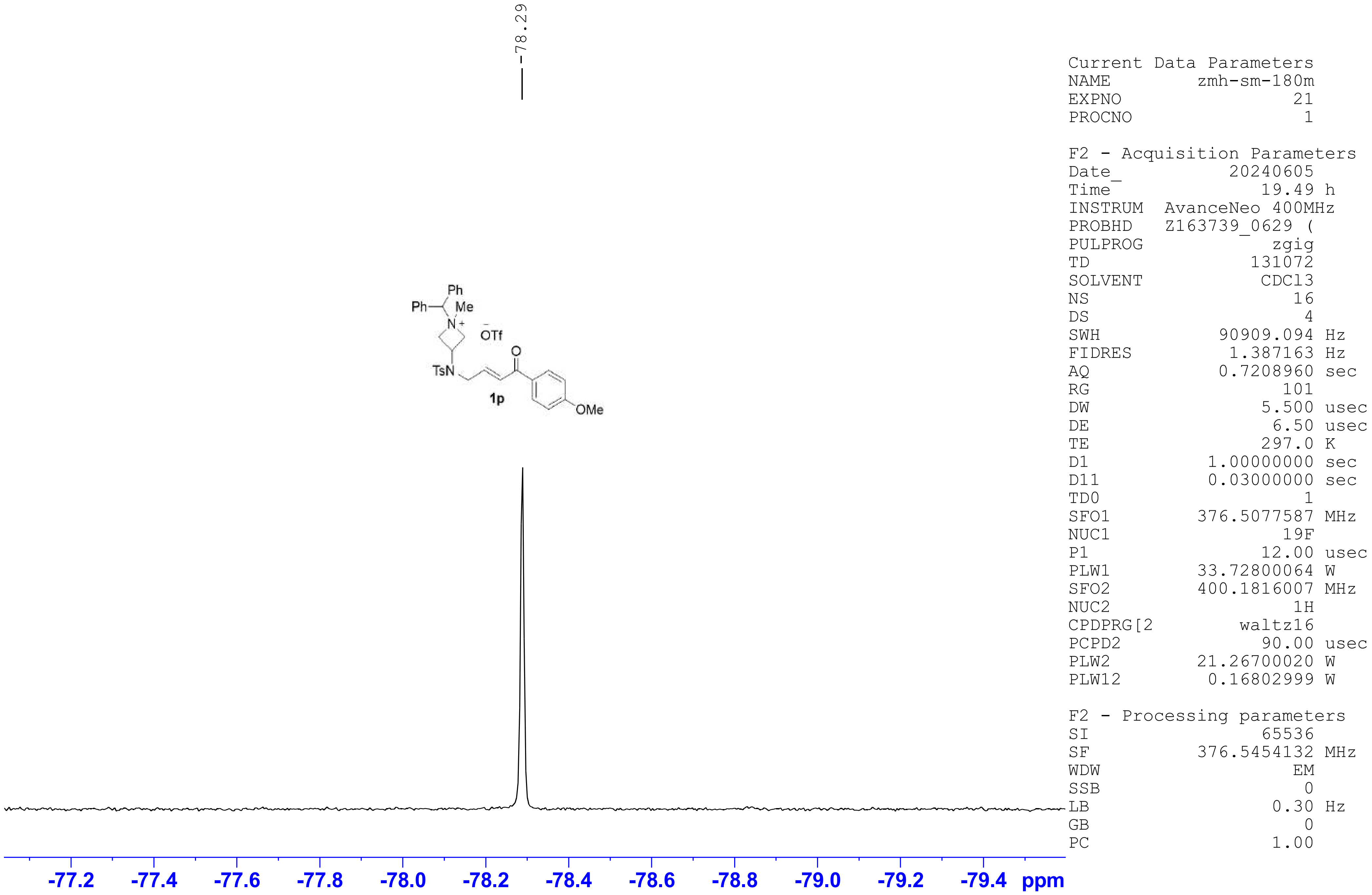
-78.29
Ph
Ph
Me
N+
-OTf
O
TsN
1p
OMe
-77.2
-77.4
-77.6
-77.8
-78.0
-78.2
-78.4
-78.6
-78.8
-79.0
-79.2
-79.4
ppm
Current Data Parameters
NAME zmh-sm-180m
EXPNO 21
PROCNO 1
F2 - Acquisition Parameters
Date_ 20240605
Time 19.49 h
INSTRUM AvanceNeo 400MHz
PROBHD Z163739_0629 (
PULPROG zgig
TD 131072
SOLVENT CDCl3
NS 16
DS 4
SWH 90909.094 Hz
FIDRES 1.387163 Hz
AQ 0.7208960 sec
RG 101
DW 5.500 usec
DE 6.50 usec
TE 297.0 K
D1 1.00000000 sec
D11 0.03000000 sec
TD0 1
SFO1 376.5077587 MHz
NUC1 19F
P1 12.00 usec
PLW1 33.72800064 W
SFO2 400.1816007 MHz
NUC2 1H
CPDPRG[2 waltz16
PCPD2 90.00 usec
PLW2 21.26700020 W
PLW12 0.16802999 W
F2 - Processing parameters
SI 65536
SF 376.5454132 MHz
WDW EM
SSB 0
LB 0.30 Hz
GB 0
PC 1.00

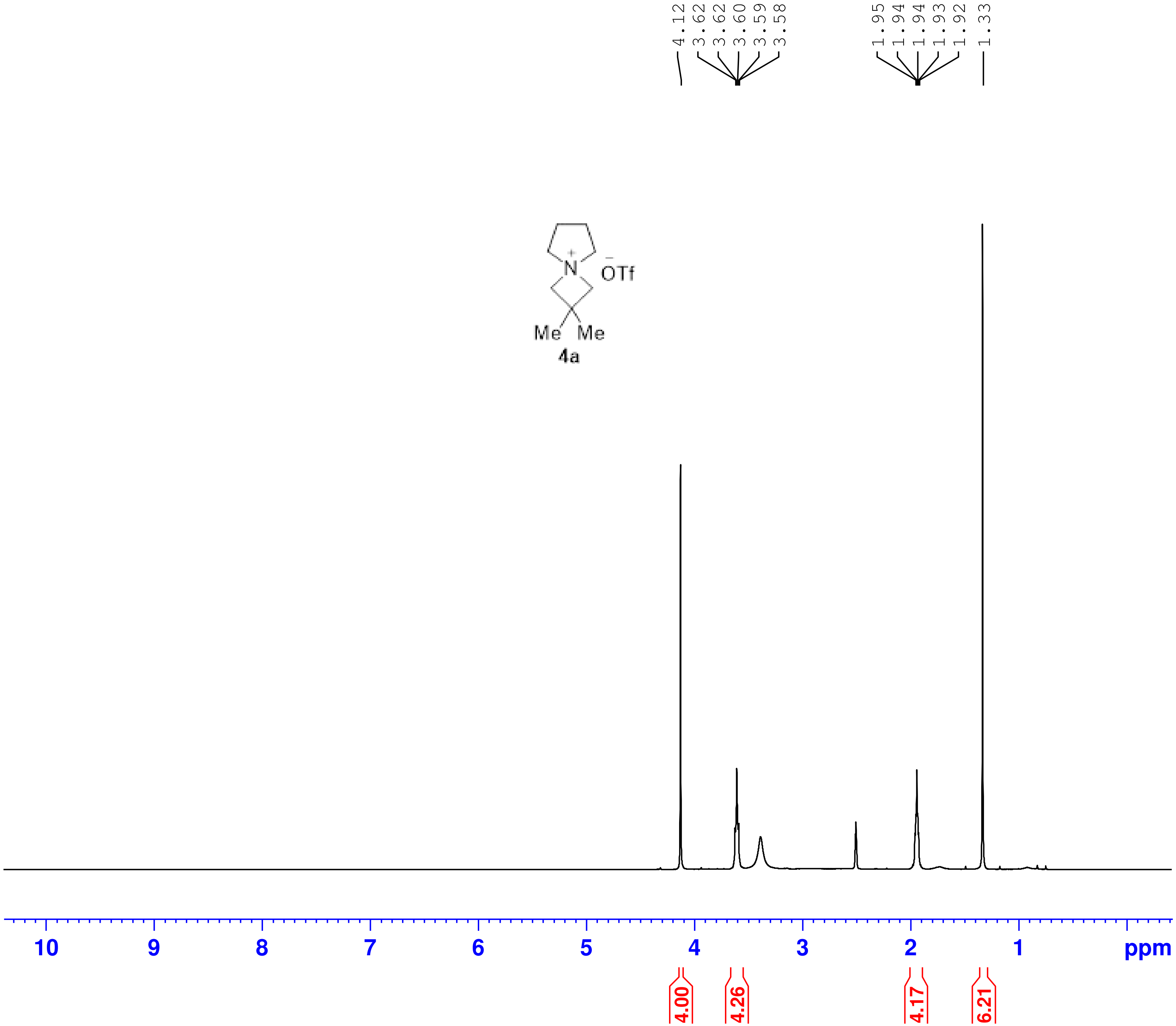

Current Data Parameters
NAME zmh-syh-6
EXPNO 2
PROCNO 1

F2 - Acquisition Parameters
Date_ 20230215
Time 13.02
INSTRUM spect
PROBHD 5 mm DUL 13C-1
PULPROG zg30
TD 65536
SOLVENT DMSO
NS 9
DS 2
SWH 8223.685 Hz
FIDRES 0.125483 Hz
AQ 3.9845889 sec
RG 203
DW 60.800 usec
DE 6.00 usec
TE 293.0 K
D1 1.00000000 sec
TD0 1

======== CHANNEL f1 ========
NUC1 1H
P1 15.80 usec
PL1 -1.00 dB
PL1W 12.17476940 W
SFO1 400.1324710 MHz

F2 - Processing parameters
SI 32768
SF 400.1300033 MHz
WDW EM
SSB 0
LB 0.30 Hz
GB 0
PC 1.00

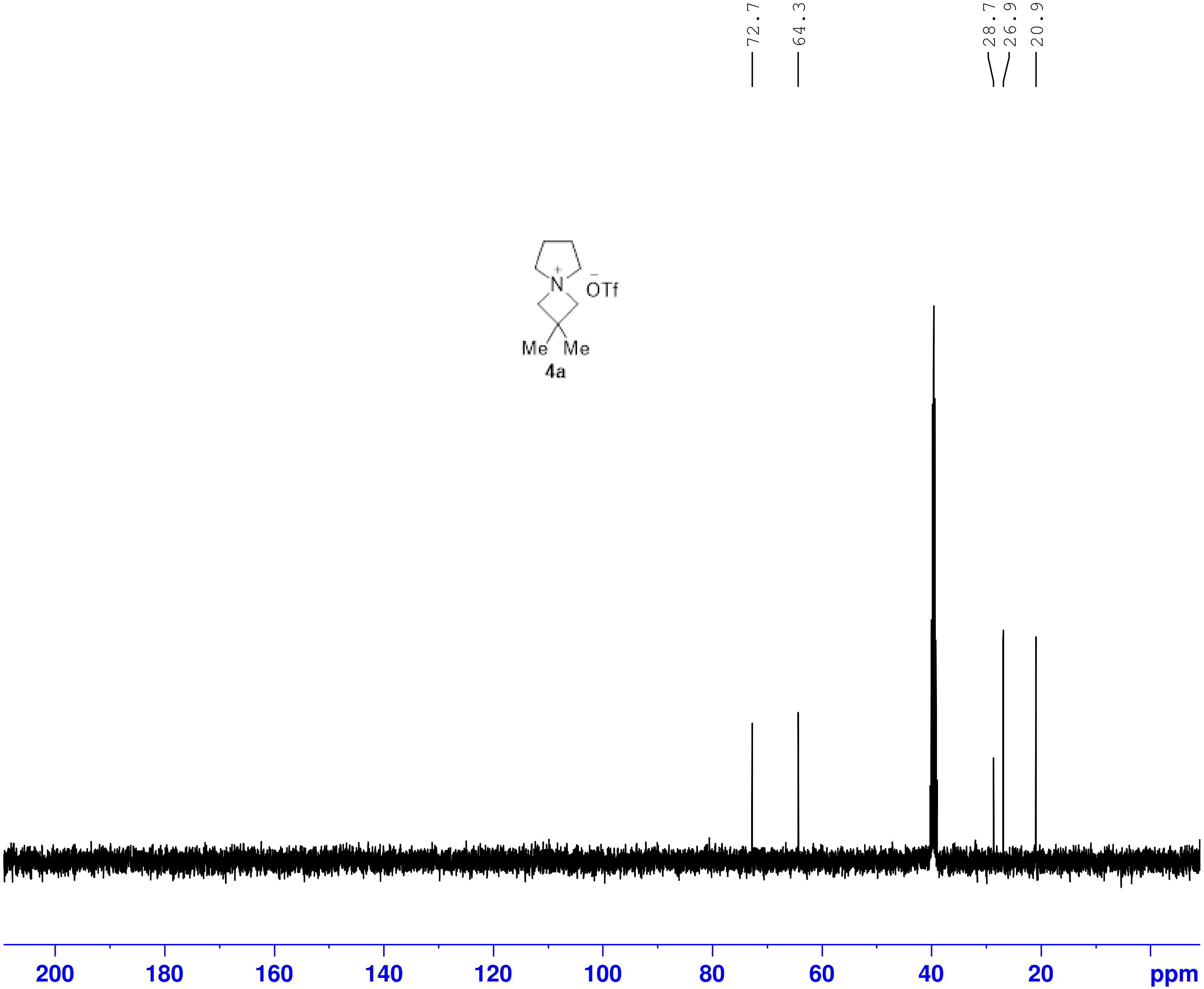


```
Current Data Parameters
NAME              zmh-syh-6
EXPNO                     1
PROCNO                    1

F2 - Acquisition Parameters
Date_              20230215
Time                  13.03
INSTRUM               spect
PROBHD   5 mm DUL 13C-1
PULPROG             zgpg30
TD                    65536
SOLVENT                DMSO
NS                       50
DS                        1
SWH            24038.461 Hz
FIDRES          0.366798 Hz
AQ             1.3631488 sec
RG                     2050
DW               20.800 usec
DE                 6.00 usec
TE                293.0 K
D1           2.00000000 sec
D11          0.03000000 sec
TD0                       1

======== CHANNEL f1 ========
NUC1                    13C
P1                40.00 usec
PL1               -3.00 dB
PL1W         60.64365387 W
SFO1        100.6228298 MHz

======== CHANNEL f2 ========
CPDPRG[2           waltz16
NUC2                     1H
PCPD2             80.00 usec
PL2               -1.00 dB
PL12              14.39 dB
PL13              18.00 dB
PL2W         12.17476940 W
PL12W         0.35193357 W
PL13W         0.15327126 W
SFO2        400.1316005 MHz

F2 - Processing parameters
SI                    32768
SF          100.6128035 MHz
WDW                      EM
SSB                       0
LB                  1.00 Hz
GB                        0
PC                     1.40
```

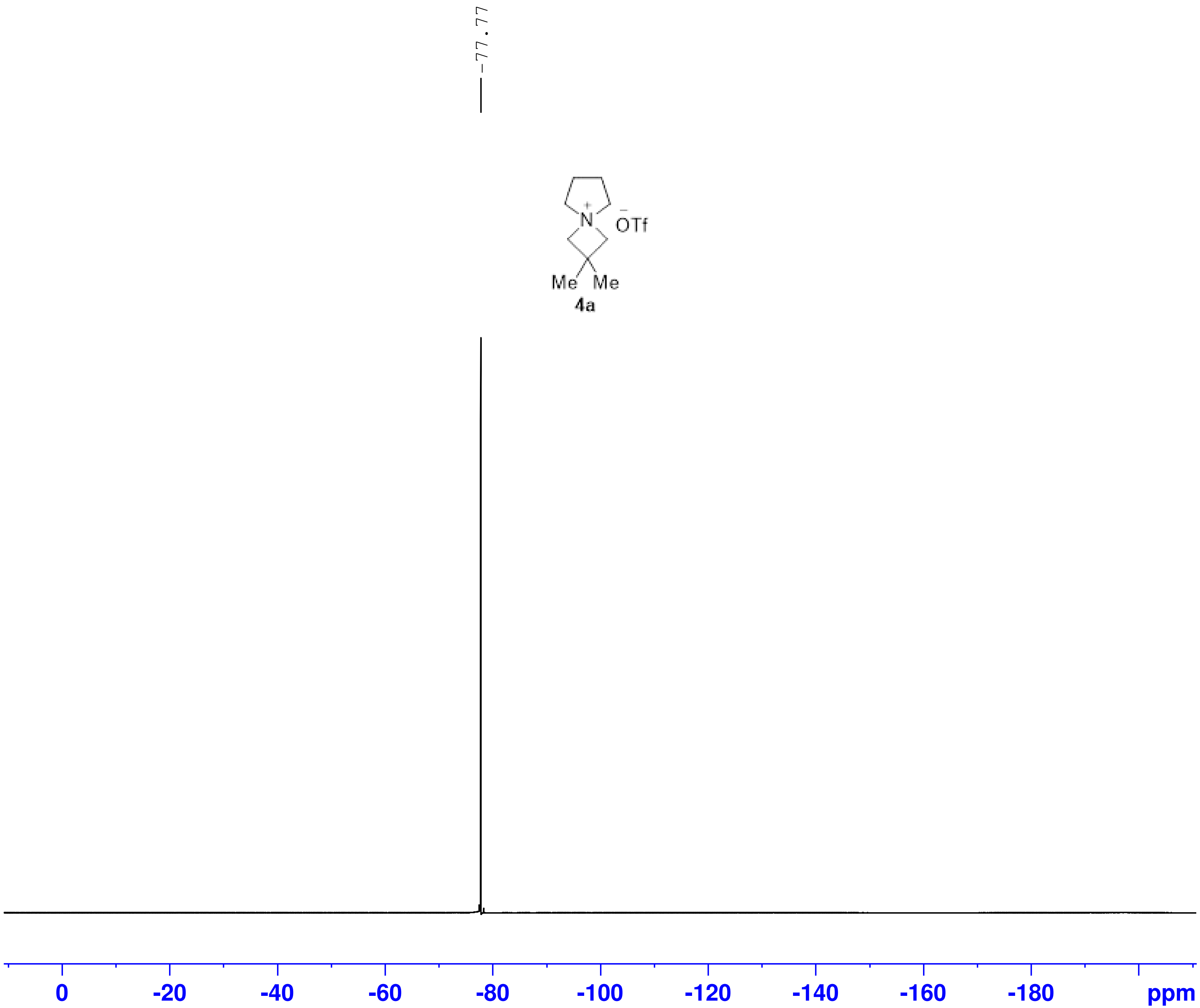

Current Data Parameters
NAME zmh-syh-6
EXPNO 1
PROCNO 1

F2 - Acquisition Parameters
Date_ 20230215
Time 18.43
INSTRUM spect
PROBHD 5 mm PABBO BB/
PULPROG zgpg30
TD 65536
SOLVENT DMSO
NS 16
DS 2
SWH 93750.000 Hz
FIDRES 1.430511 Hz
AQ 0.3495253 sec
RG 196.92
DW 5.333 usec
DE 6.50 usec
TE 294.4 K
D1 2.00000000 sec
D11 0.03000000 sec
TD0 1

======== CHANNEL f1 ========
SFO1 376.4607162 MHz
NUC1 19F
P1 14.70 usec
PLW1 15.99600029 W

======== CHANNEL f2 ========
SFO2 400.1316005 MHz
NUC2 1H
CPDPRG[2 waltz16
PCPD2 90.00 usec
PLW2 11.99499989 W
PLW12 0.34213999 W
PLW13 0.27713001 W

F2 - Processing parameters
SI 32768
SF 376.4983660 MHz
WDW EM
SSB 0
LB 1.00 Hz
GB 0
PC 1.40

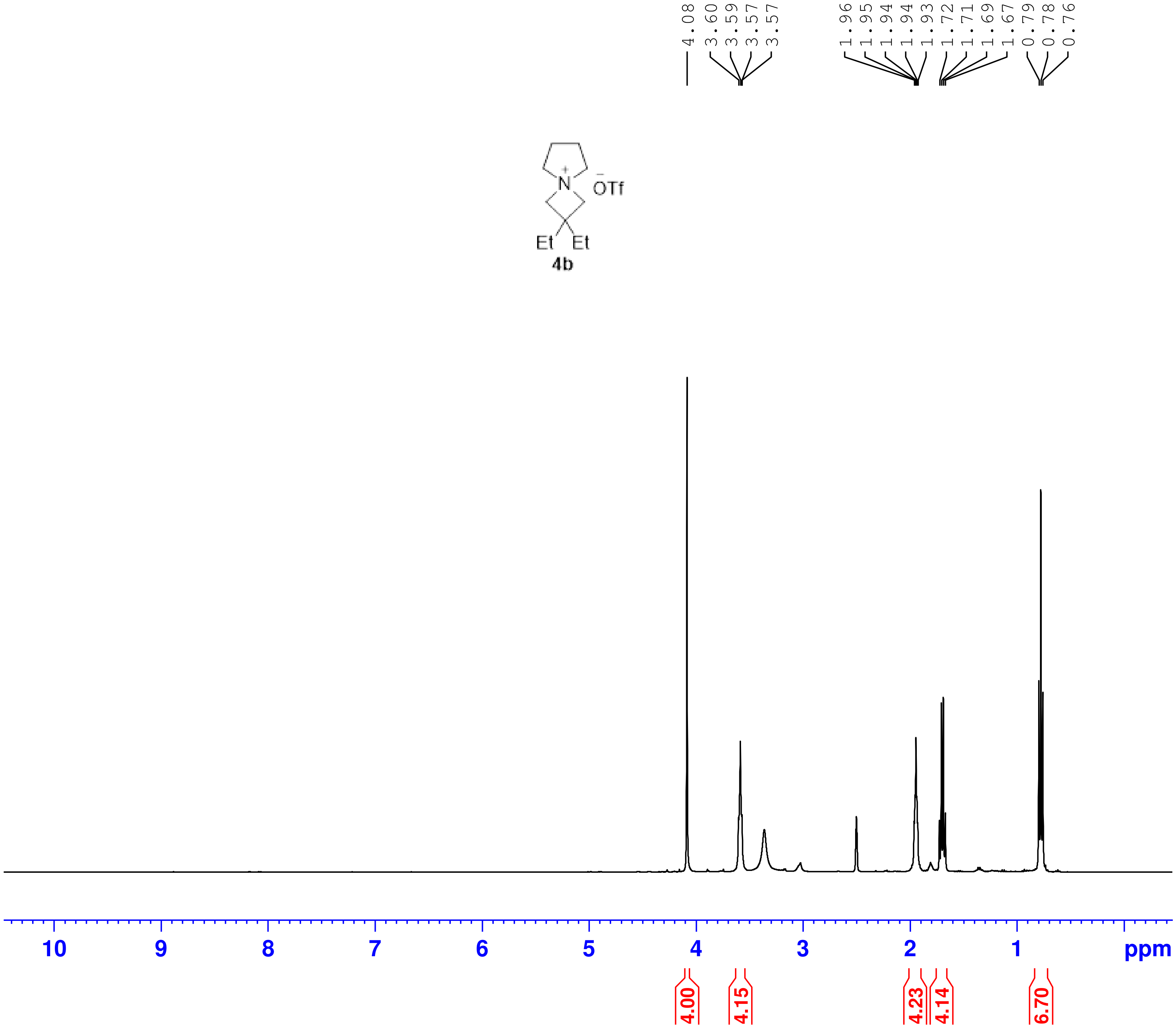

Current Data Parameters
NAME zmh-syh-5
EXPNO 2
PROCNO 1

F2 - Acquisition Parameters
Date_ 20230215
Time 13.31
INSTRUM spect
PROBHD 5 mm DUL 13C-1
PULPROG zg30
TD 65536
SOLVENT DMSO
NS 15
DS 2
SWH 8223.685 Hz
FIDRES 0.125483 Hz
AQ 3.9845889 sec
RG 161
DW 60.800 usec
DE 6.00 usec
TE 293.0 K
D1 1.00000000 sec
TD0 1

======== CHANNEL f1 ========
NUC1 1H
P1 15.80 usec
PL1 -1.00 dB
PL1W 12.17476940 W
SFO1 400.1324710 MHz

F2 - Processing parameters
SI 32768
SF 400.1300032 MHz
WDW EM
SSB 0
LB 0.30 Hz
GB 0
PC 1.00

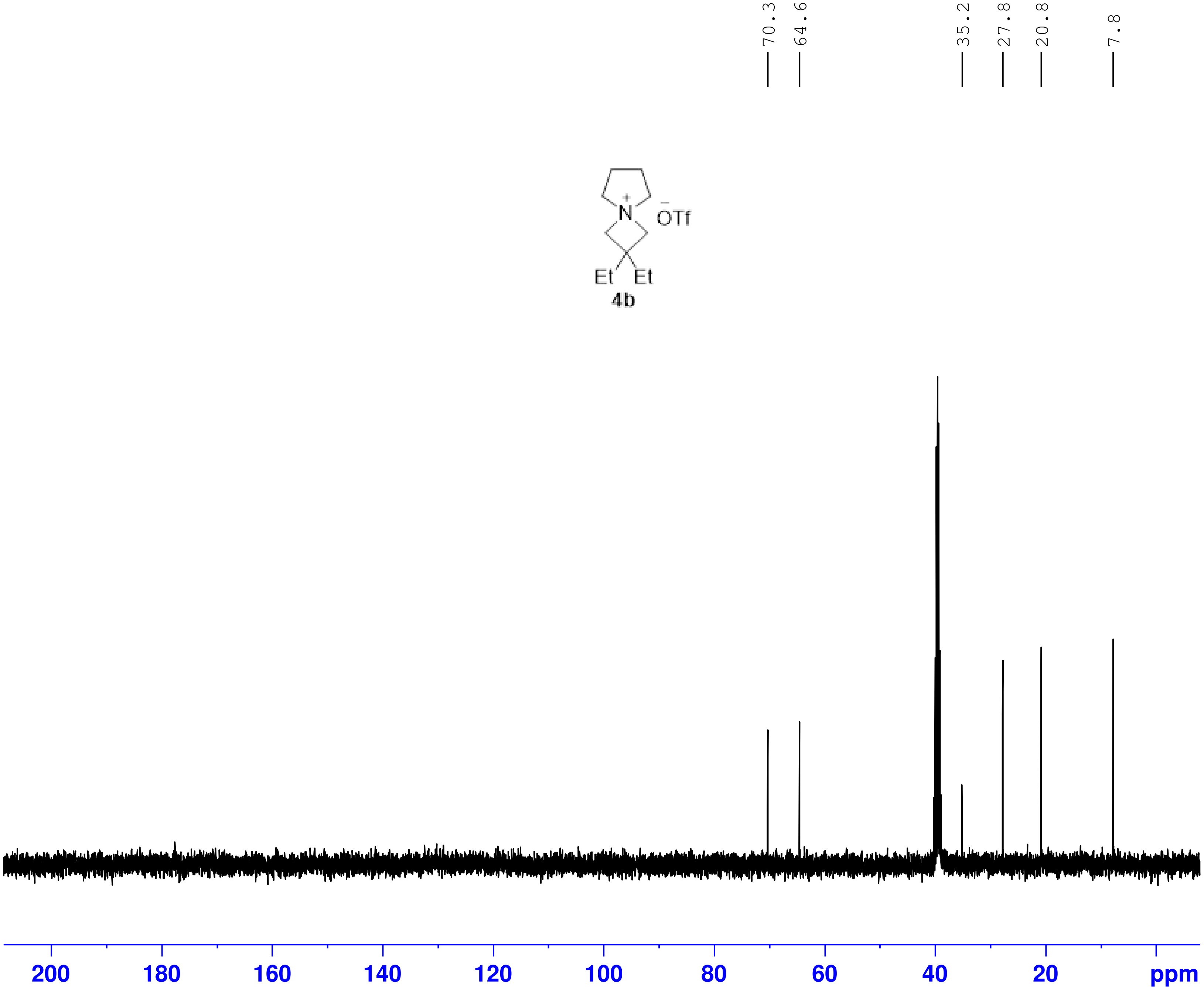

4b

Current Data Parameters
NAME zmh-syh-5
EXPNO 1
PROCNO 1

F2 - Acquisition Parameters
Date_ 20230215
Time 13.34
INSTRUM spect
PROBHD 5 mm DUL 13C-1
PULPROG zgpg30
TD 65536
SOLVENT DMSO
NS 43
DS 1
SWH 24038.461 Hz
FIDRES 0.366798 Hz
AQ 1.3631488 sec
RG 2050
DW 20.800 usec
DE 6.00 usec
TE 293.1 K
D1 2.00000000 sec
D11 0.03000000 sec
TD0 1

======== CHANNEL f1 ========
NUC1 13C
P1 40.00 usec
PL1 -3.00 dB
PL1W 60.64365387 W
SFO1 100.6228298 MHz

======== CHANNEL f2 ========
CPDPRG[2 waltz16
NUC2 1H
PCPD2 80.00 usec
PL2 -1.00 dB
PL12 14.39 dB
PL13 18.00 dB
PL2W 12.17476940 W
PL12W 0.35193357 W
PL13W 0.15327126 W
SFO2 400.1316005 MHz

F2 - Processing parameters
SI 32768
SF 100.6128037 MHz
WDW EM
SSB 0
LB 1.00 Hz
GB 0
PC 1.40

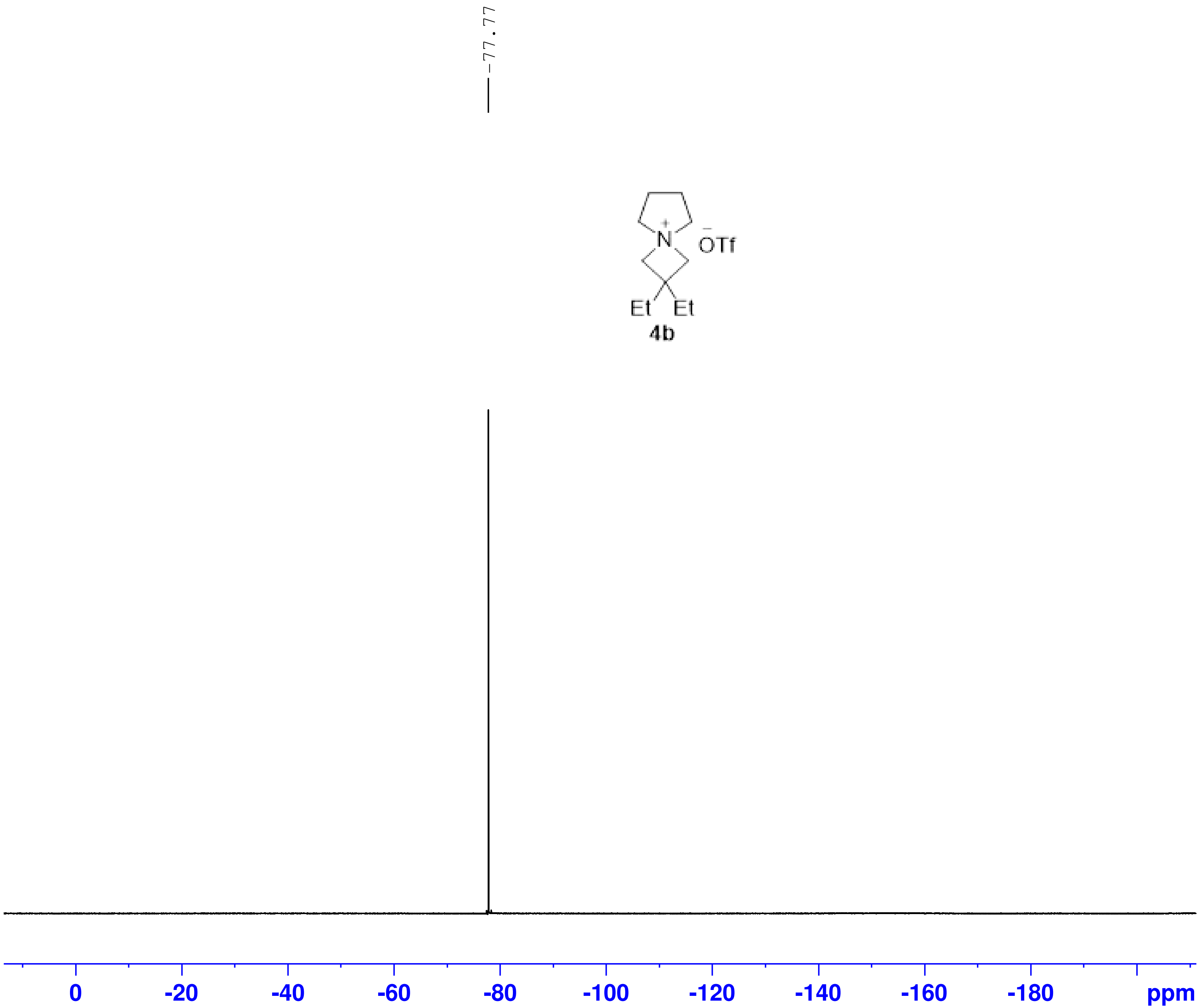

Current Data Parameters
NAME zmh-syh-5-F
EXPNO 1
PROCNO 1

F2 - Acquisition Parameters
Date_ 20230830
Time 20.30 h
INSTRUM AvanceNeo 400MHz
PROBHD Z163739_0629 (
PULPROG zgig
TD 131072
SOLVENT DMSO
NS 8
DS 4
SWH 90909.094 Hz
FIDRES 1.387163 Hz
AQ 0.7208960 sec
RG 101
DW 5.500 usec
DE 6.50 usec
TE 296.8 K
D1 1.00000000 sec
D11 0.03000000 sec
TD0 1
SFO1 376.5077587 MHz
NUC1 19F
P1 12.00 usec
PLW1 33.72800064 W
SFO2 400.1816007 MHz
NUC2 1H
CPDPRG[2 waltz16
PCPD2 90.00 usec
PLW2 21.26700020 W
PLW12 0.16802999 W

F2 - Processing parameters
SI 65536
SF 376.5454132 MHz
WDW EM
SSB 0
LB 0.30 Hz
GB 0
PC 1.00

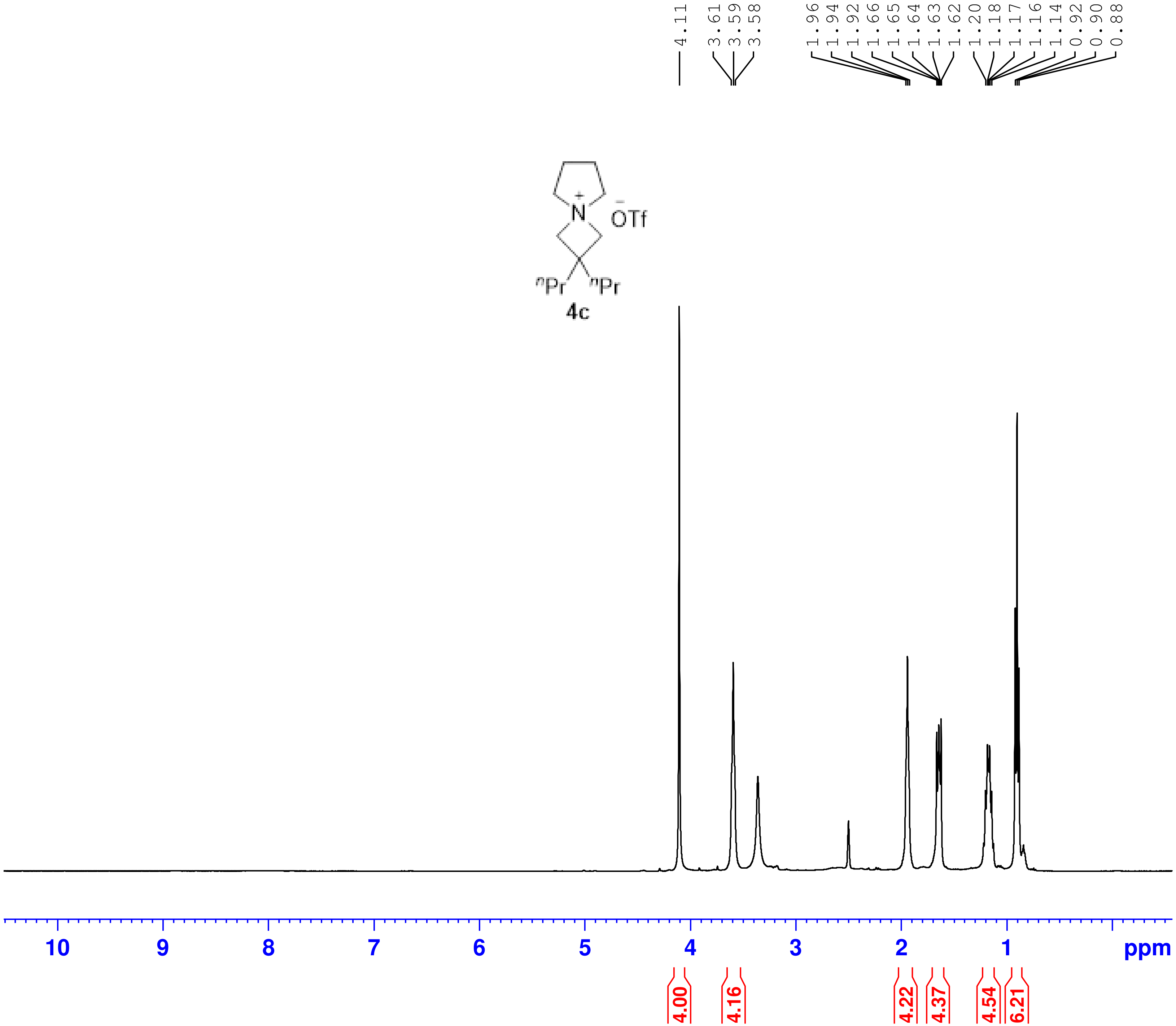

Current Data Parameters
NAME zmh-syh-11
EXPNO 1
PROCNO 1

F2 - Acquisition Parameters
Date_ 20230827
Time 19.33 h
INSTRUM AvanceNeo 400MHz
PROBHD Z163739_0629 (
PULPROG zg30
TD 65536
SOLVENT DMSO
NS 8
DS 2
SWH 8196.722 Hz
FIDRES 0.250144 Hz
AQ 3.9976959 sec
RG 36
DW 61.000 usec
DE 13.89 usec
TE 297.1 K
D1 1.00000000 sec
TD0 1
SFO1 400.1824711 MHz
NUC1 1H
P0 2.67 usec
P1 8.00 usec
PLW1 21.26700020 W

F2 - Processing parameters
SI 65536
SF 400.1800025 MHz
WDW EM
SSB 0
LB 0.30 Hz
GB 0
PC 1.00

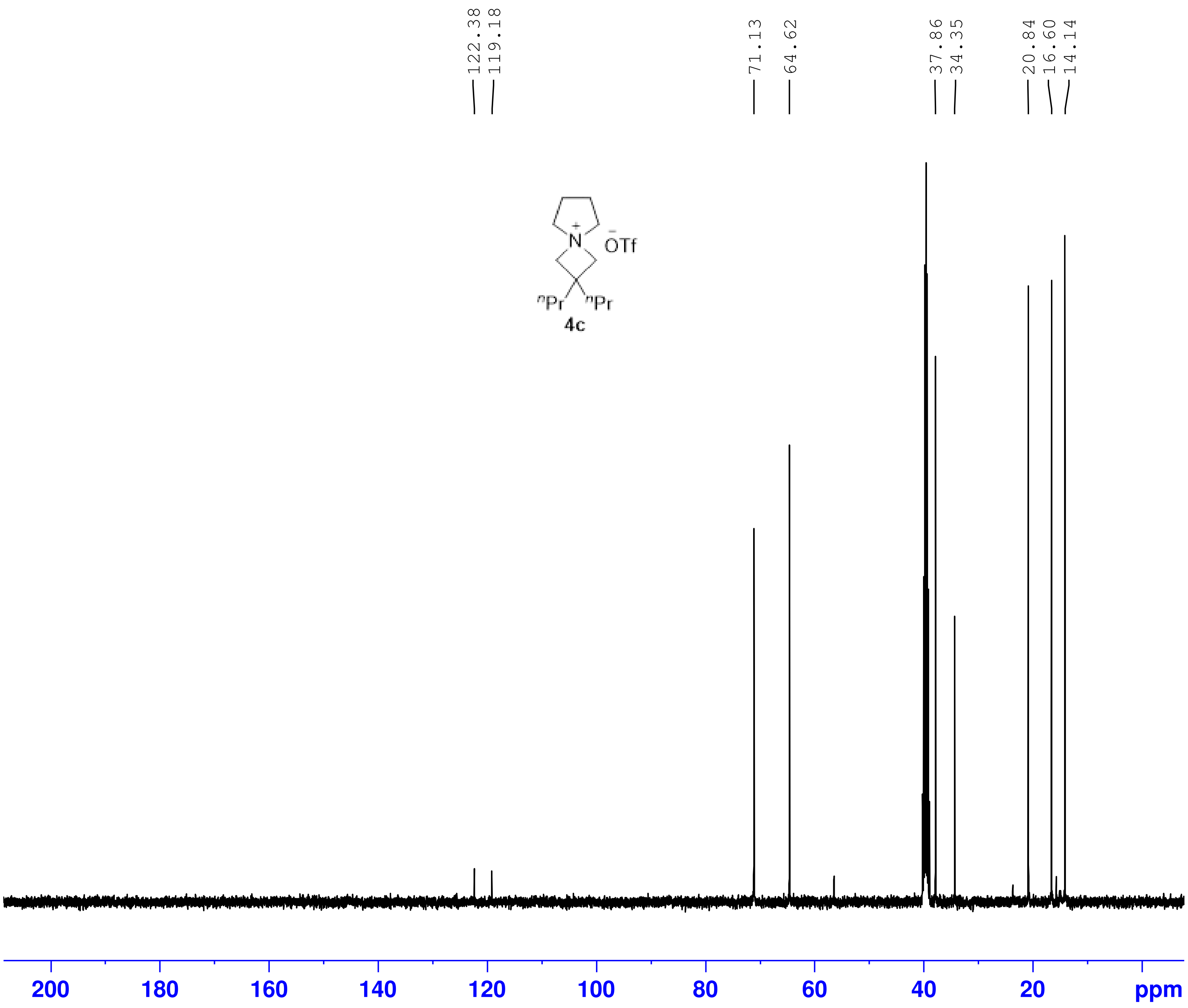

Current Data Parameters
NAME zmh-syh-11
EXPNO 2
PROCNO 1

F2 - Acquisition Parameters
Date_ 20230827
Time 19.42 h
INSTRUM AvanceNeo 400MHz
PROBHD Z163739_0629 (
PULPROG zgpg30
TD 65536
SOLVENT DMSO
NS 100
DS 4
SWH 23809.523 Hz
FIDRES 0.726609 Hz
AQ 1.3762560 sec
RG 10
DW 21.000 usec
DE 6.50 usec
TE 297.8 K
D1 2.00000000 sec
D11 0.03000000 sec
TD0 1
SFO1 100.6354036 MHz
NUC1 13C
P0 2.67 usec
P1 8.00 usec
PLW1 85.25399780 W
SFO2 400.1816007 MHz
NUC2 1H
CPDPRG[2 waltz65
PCPD2 90.00 usec
PLW2 21.26700020 W
PLW12 0.16802999 W
PLW13 0.08452000 W

F2 - Processing parameters
SI 32768
SF 100.6253770 MHz
WDW EM
SSB 0
LB 1.00 Hz
GB 0
PC 1.40

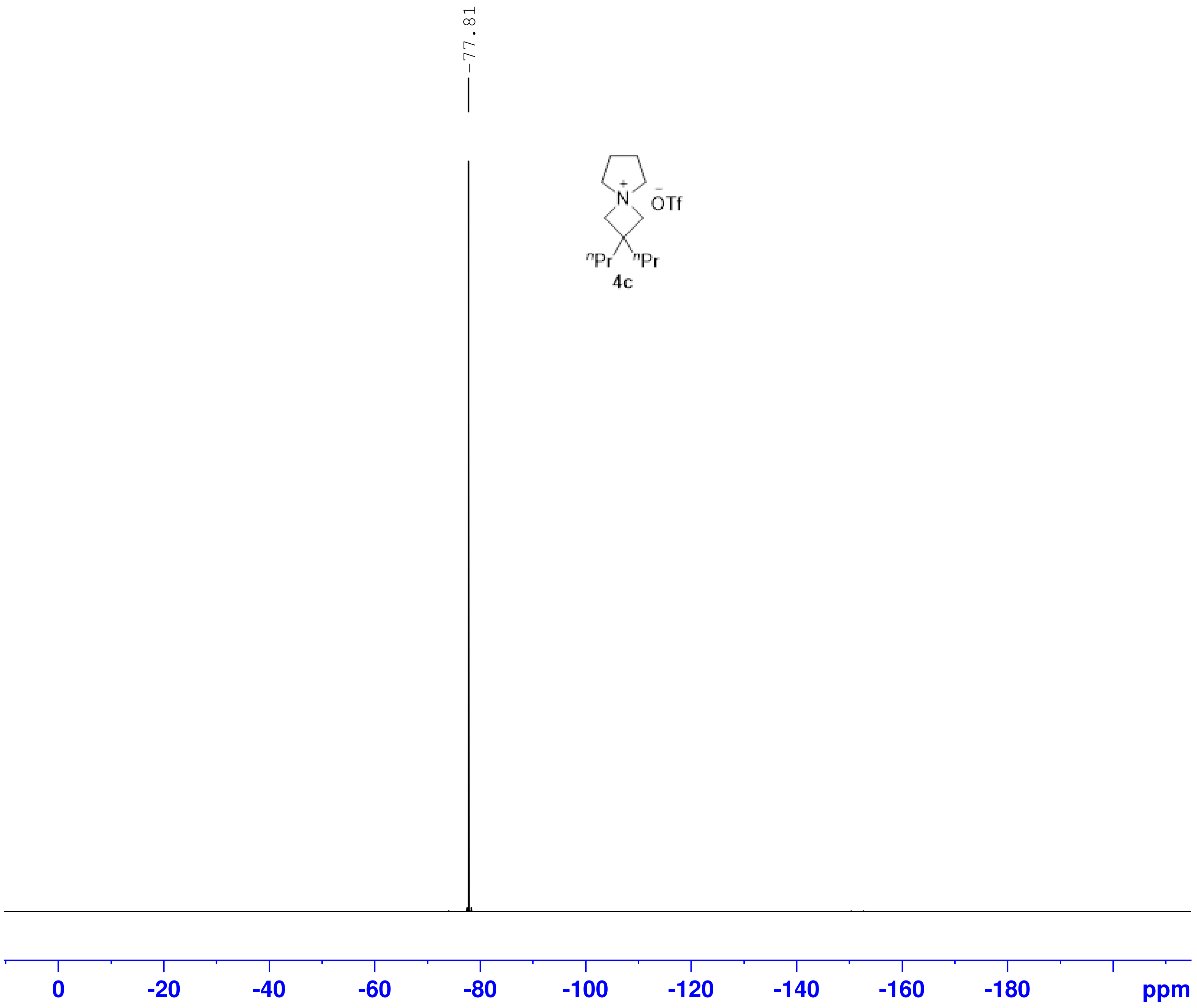

Current Data Parameters
NAME zmh-syh-11
EXPNO 3
PROCNO 1

F2 - Acquisition Parameters
Date_ 20230827
Time 19.44 h
INSTRUM AvanceNeo 400MHz
PROBHD Z163739_0629 (
PULPROG zgig
TD 131072
SOLVENT DMSO
NS 8
DS 4
SWH 90909.094 Hz
FIDRES 1.387163 Hz
AQ 0.7208960 sec
RG 101
DW 5.500 usec
DE 6.50 usec
TE 297.3 K
D1 1.00000000 sec
D11 0.03000000 sec
TD0 1
SFO1 376.5077587 MHz
NUC1 19F
P1 12.00 usec
PLW1 33.72800064 W
SFO2 400.1816007 MHz
NUC2 1H
CPDPRG[2 waltz16
PCPD2 90.00 usec
PLW2 21.26700020 W
PLW12 0.16802999 W

F2 - Processing parameters
SI 65536
SF 376.5454132 MHz
WDW EM
SSB 0
LB 0.30 Hz
GB 0
PC 1.00

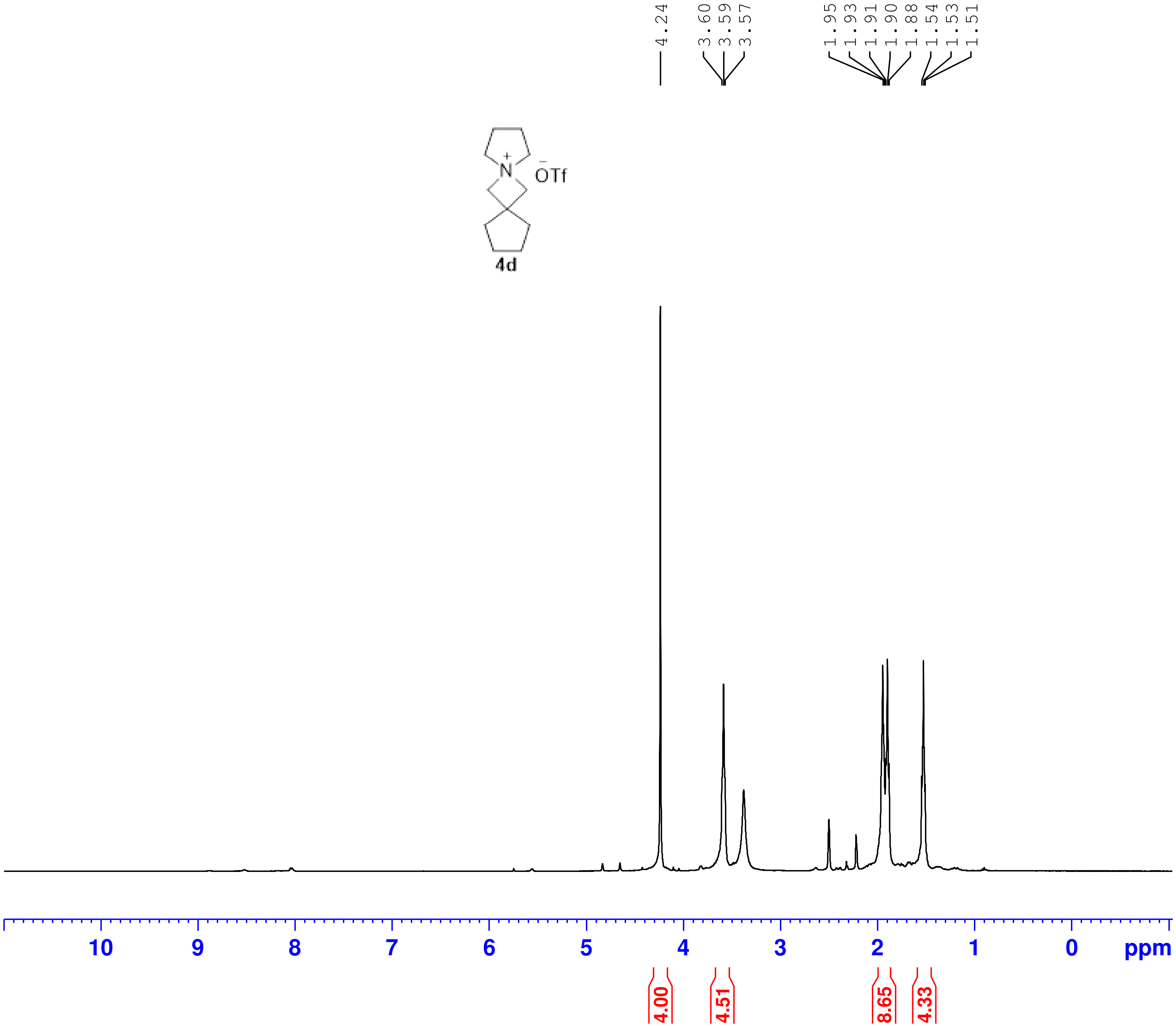

Current Data Parameters
NAME zmh-syh-4
EXPNO 2
PROCNO 1

F2 - Acquisition Parameters
Date_ 20230215
Time 13.13
INSTRUM spect
PROBHD 5 mm DUL 13C-1
PULPROG zg30
TD 65536
SOLVENT DMSO
NS 16
DS 2
SWH 8223.685 Hz
FIDRES 0.125483 Hz
AQ 3.9845889 sec
RG 128
DW 60.800 usec
DE 6.00 usec
TE 292.9 K
D1 1.00000000 sec
TD0 1

======== CHANNEL f1 ========
NUC1 1H
P1 15.80 usec
PL1 -1.00 dB
PL1W 12.17476940 W
SFO1 400.1324710 MHz

F2 - Processing parameters
SI 32768
SF 400.1300035 MHz
WDW EM
SSB 0
LB 0.30 Hz
GB 0
PC 1.00

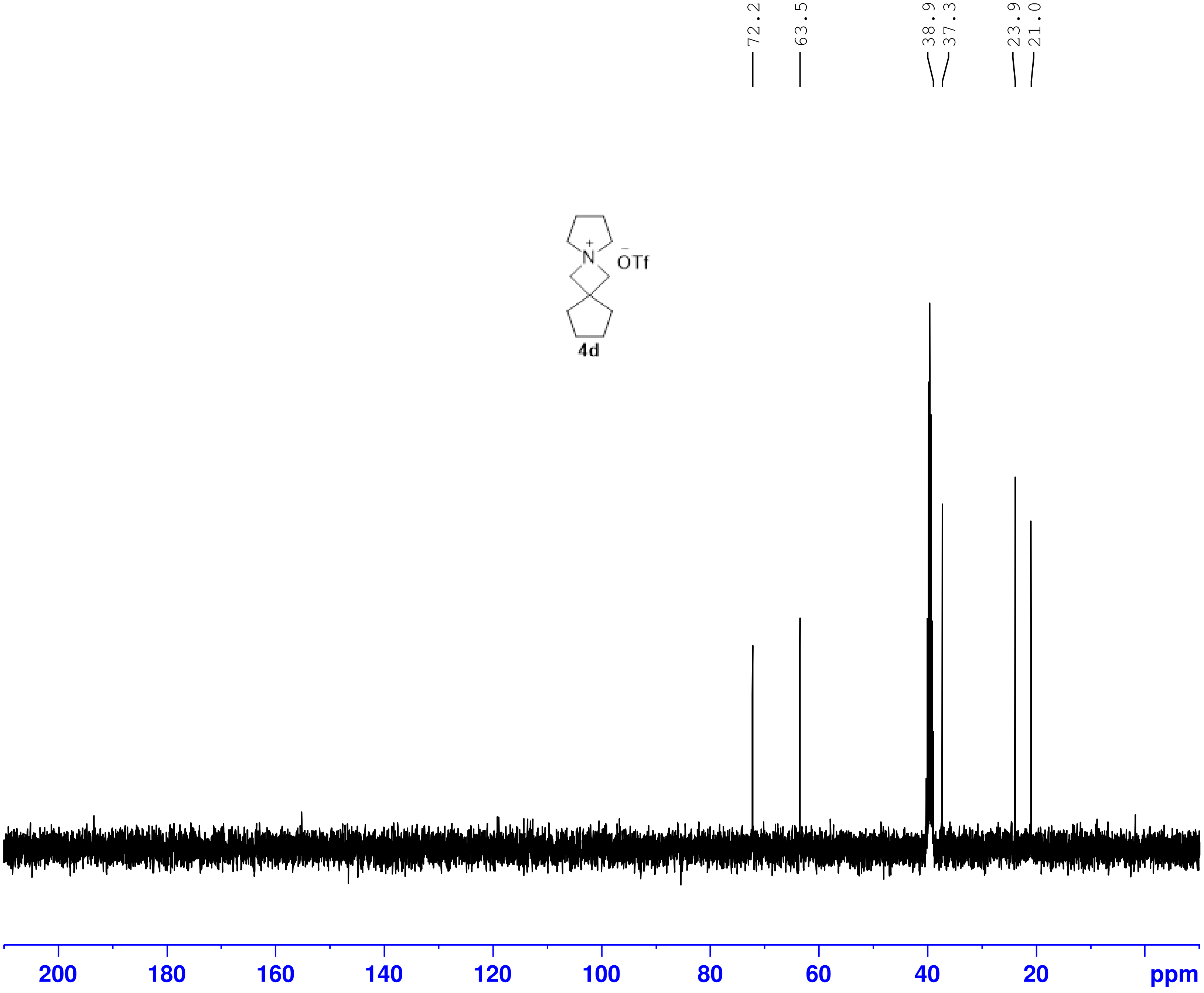


```
Current Data Parameters
NAME              zmh-syh-4
EXPNO                     1
PROCNO                    1

F2 - Acquisition Parameters
Date_              20230215
Time                  13.15
INSTRUM               spect
PROBHD     5 mm DUL 13C-1
PULPROG             zgpg30
TD                   65536
SOLVENT               DMSO
NS                      44
DS                       1
SWH              24038.461 Hz
FIDRES            0.366798 Hz
AQ               1.3631488 sec
RG                    2050
DW                  20.800 usec
DE                    6.00 usec
TE                   293.0 K
D1              2.00000000 sec
D11             0.03000000 sec
TD0                      1

======== CHANNEL f1 ========
NUC1                   13C
P1                   40.00 usec
PL1                  -3.00 dB
PL1W           60.64365387 W
SFO1           100.6228298 MHz

======== CHANNEL f2 ========
CPDPRG[2           waltz16
NUC2                    1H
PCPD2                80.00 usec
PL2                  -1.00 dB
PL12                 14.39 dB
PL13                 18.00 dB
PL2W           12.17476940 W
PL12W           0.35193357 W
PL13W           0.15327126 W
SFO2           400.1316005 MHz

F2 - Processing parameters
SI                   32768
SF             100.6128021 MHz
WDW                     EM
SSB                      0
LB                    1.00 Hz
GB                       0
PC                    1.40
```

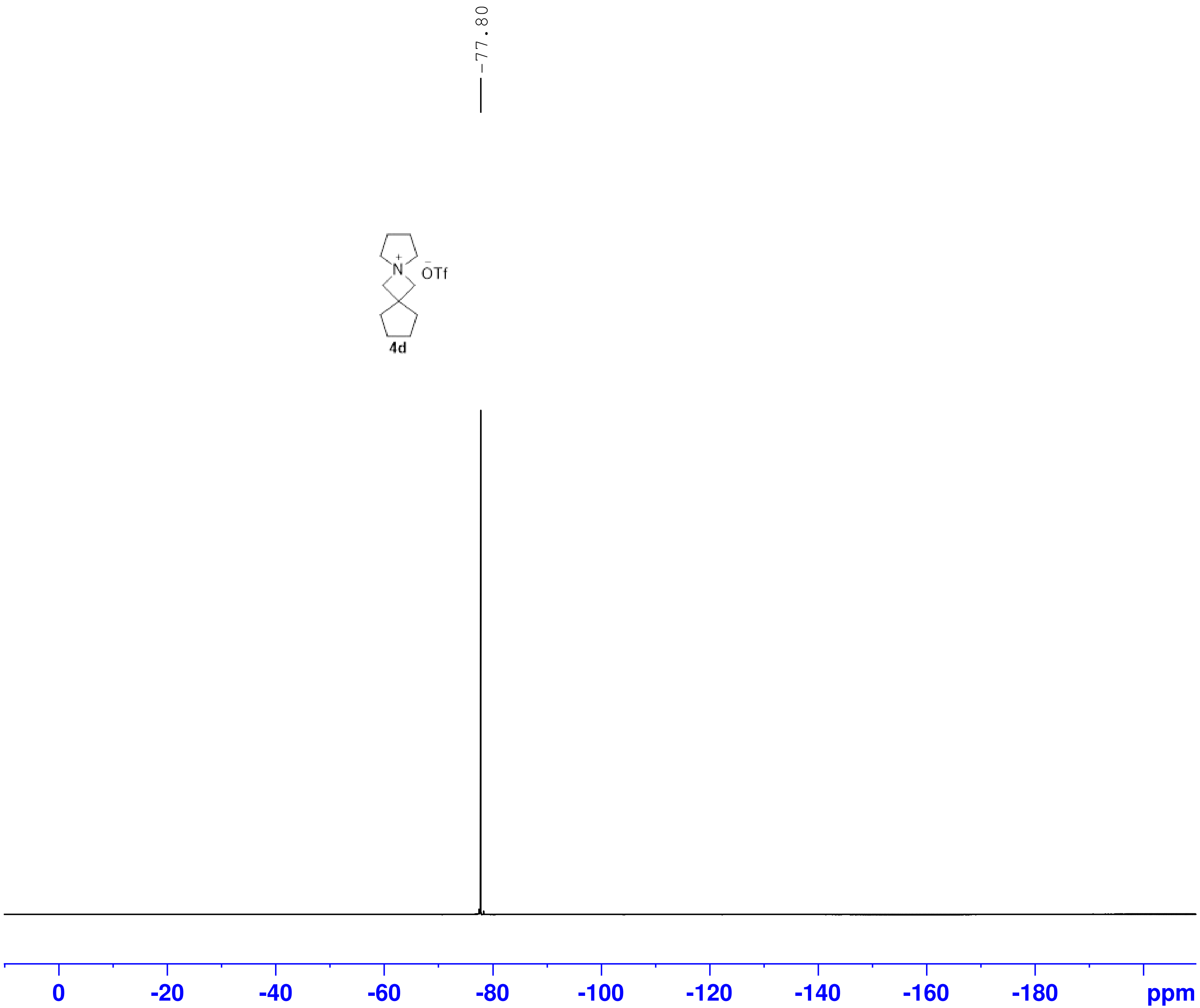

Current Data Parameters

NAME	zmh-syh-4
EXPNO	1
PROCNO	1

F2 - Acquisition Parameters

Date_	20230215
Time	18.39
INSTRUM	spect
PROBHD	5 mm PABBO BB/
PULPROG	zgpg30
TD	65536
SOLVENT	DMSO
NS	16
DS	2
SWH	93750.000 Hz
FIDRES	1.430511 Hz
AQ	0.3495253 sec
RG	196.92
DW	5.333 usec
DE	6.50 usec
TE	294.4 K
D1	2.00000000 sec
D11	0.03000000 sec
TD0	1

======== CHANNEL f1 ========

SFO1	376.4607162 MHz
NUC1	19F
P1	14.70 usec
PLW1	15.99600029 W

======== CHANNEL f2 ========

SFO2	400.1316005 MHz
NUC2	1H
CPDPRG[2	waltz16
PCPD2	90.00 usec
PLW2	11.99499989 W
PLW12	0.34213999 W
PLW13	0.27713001 W

F2 - Processing parameters

SI	32768
SF	376.4983660 MHz
WDW	EM
SSB	0
LB	1.00 Hz
GB	0
PC	1.40

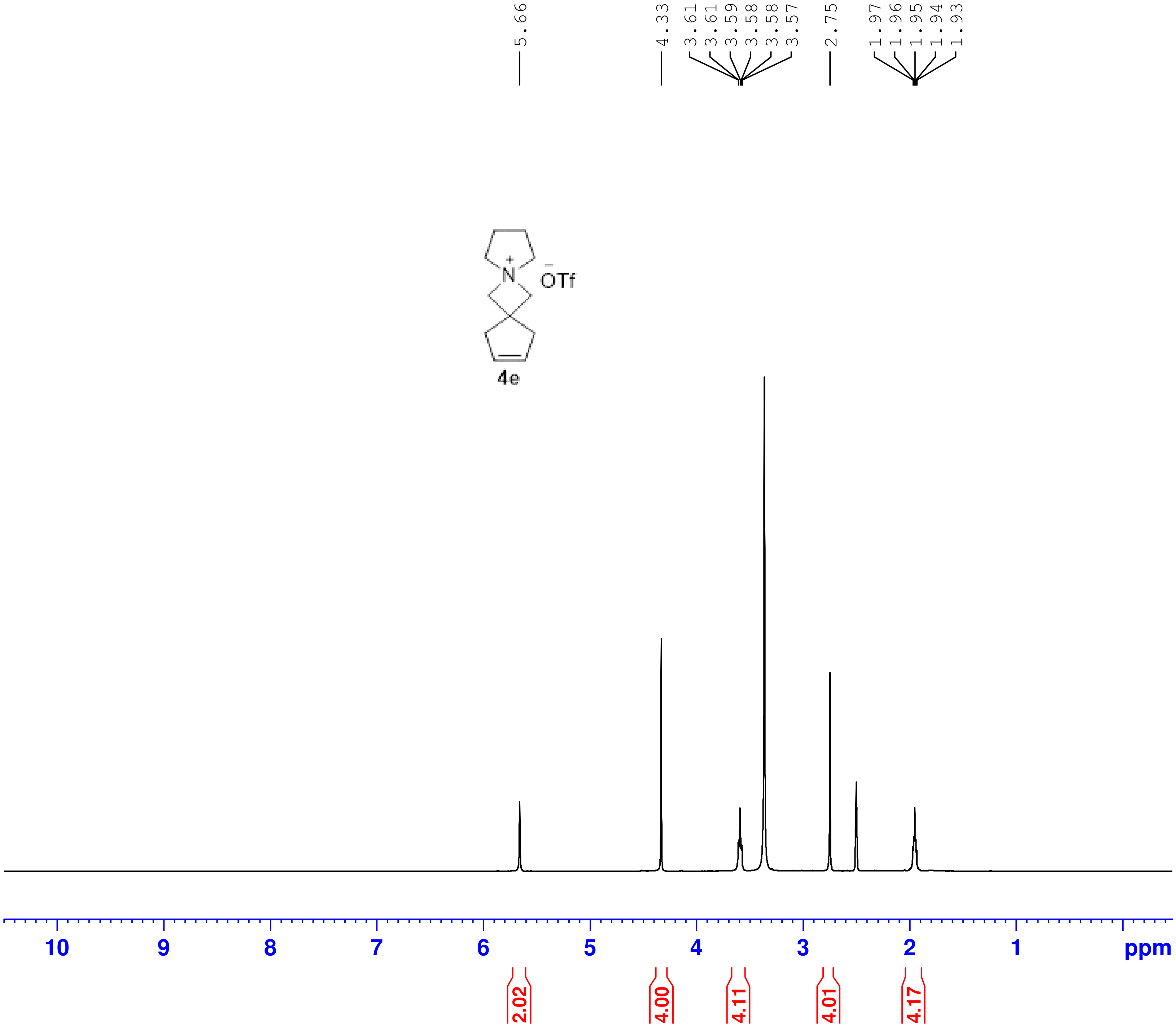


```
Current Data Parameters
NAME       zmh-syh-10-dmso
EXPNO                    1
PROCNO                   1

F2 - Acquisition Parameters
Date_             20230827
Time                 20.06
INSTRUM              spect
PROBHD   5 mm PABBO BB/
PULPROG               zg30
TD                   65536
SOLVENT               DMSO
NS                       8
DS                       2
SWH               8012.820 Hz
FIDRES            0.122266 Hz
AQ               4.0894465 sec
RG                   82.92
DW                  62.400 usec
DE                    6.50 usec
TE                   296.5 K
D1              1.00000000 sec
TD0                      1

======== CHANNEL f1 ========
SFO1           400.1324710 MHz
NUC1                    1H
P1                   14.50 usec
PLW1           11.99499989 W

F2 - Processing parameters
SI                   65536
SF             400.1300031 MHz
WDW                     EM
SSB                      0
LB                    0.30 Hz
GB                       0
PC                    1.00
```

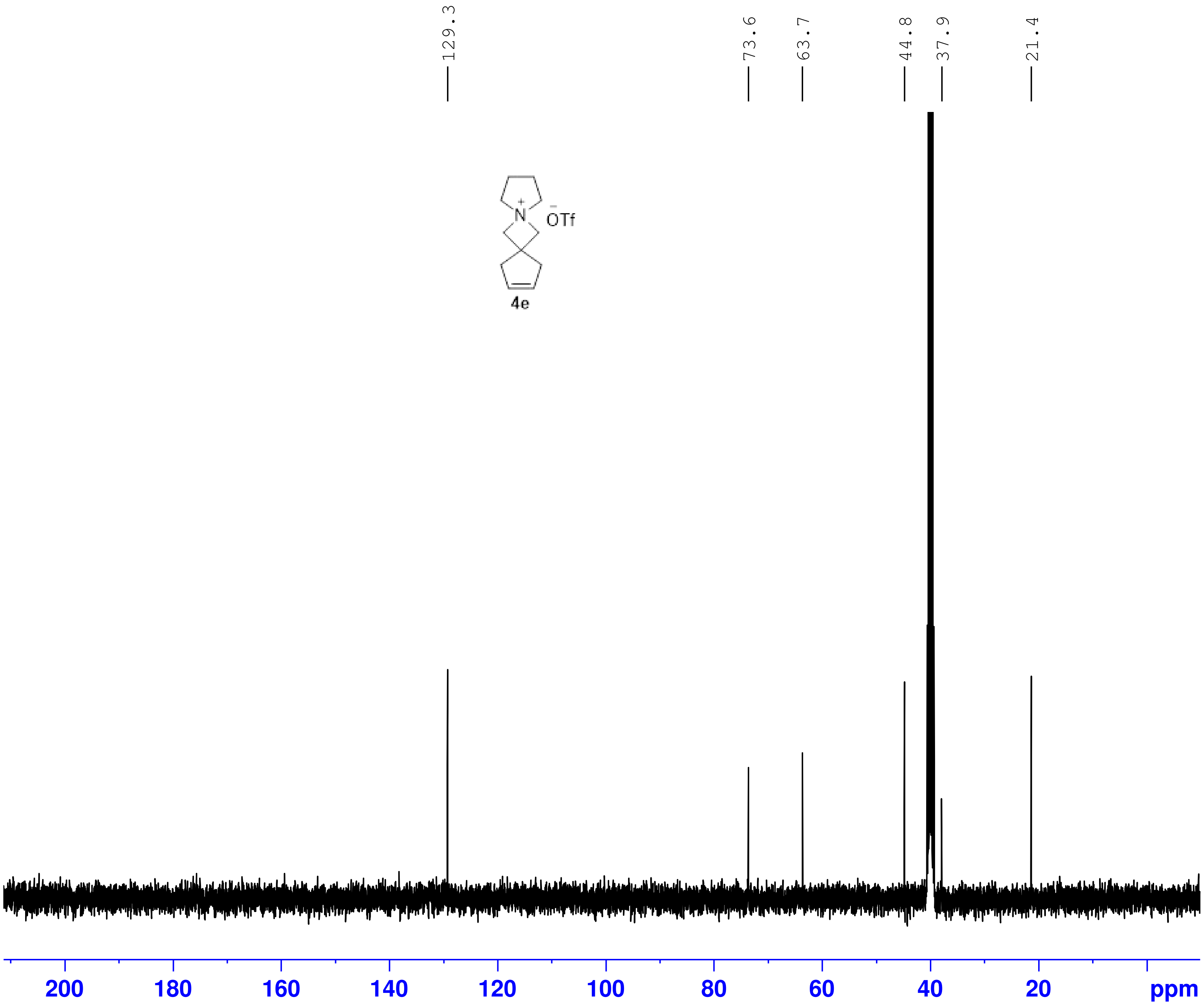

4e

Current Data Parameters
NAME zmh-syh-10-dmso
EXPNO 2
PROCNO 1

F2 - Acquisition Parameters
Date_ 20230827
Time 20.09
INSTRUM spect
PROBHD 5 mm PABBO BB/
PULPROG zgpg30
TD 65536
SOLVENT DMSO
NS 102
DS 2
SWH 24038.461 Hz
FIDRES 0.366798 Hz
AQ 1.3631488 sec
RG 196.92
DW 20.800 usec
DE 6.50 usec
TE 297.1 K
D1 2.00000000 sec
D11 0.03000000 sec
TD0 1

======== CHANNEL f1 ========
SFO1 100.6228298 MHz
NUC1 13C
P1 9.70 usec
PLW1 46.98899841 W

======== CHANNEL f2 ========
SFO2 400.1316005 MHz
NUC2 1H
CPDPRG[2 waltz16
PCPD2 90.00 usec
PLW2 11.99499989 W
PLW12 0.34213999 W
PLW13 0.27713001 W

F2 - Processing parameters
SI 32768
SF 100.6127690 MHz
WDW EM
SSB 0
LB 1.00 Hz
GB 0
PC 1.40

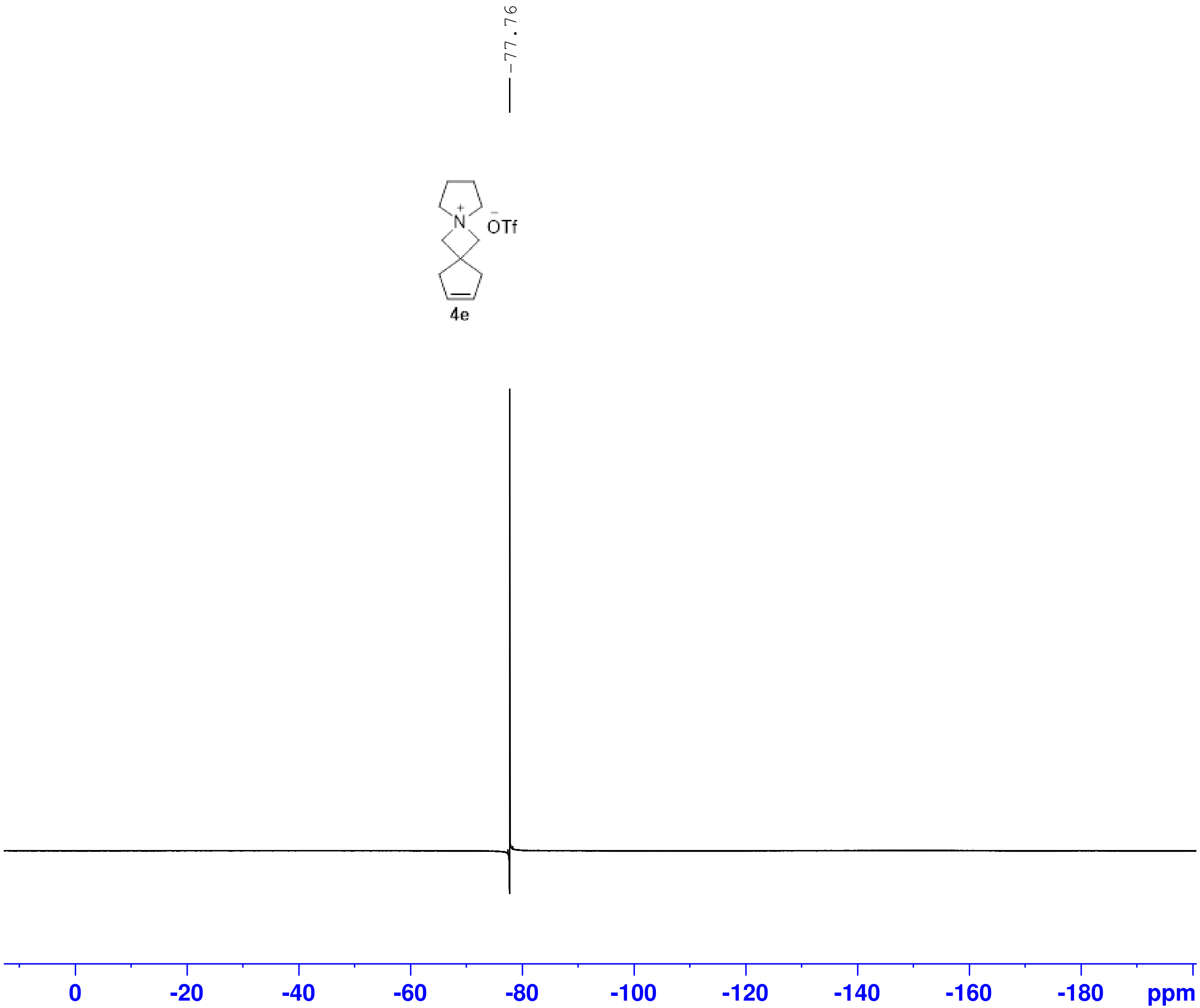

4e

Current Data Parameters
NAME zmh-syh-10-dmso
EXPNO 3
PROCNO 1

F2 - Acquisition Parameters
Date_ 20230827
Time 20.16
INSTRUM spect
PROBHD 5 mm PABBO BB/
PULPROG zgpg30
TD 65536
SOLVENT DMSO
NS 37
DS 2
SWH 93750.000 Hz
FIDRES 1.430511 Hz
AQ 0.3495253 sec
RG 196.92
DW 5.333 usec
DE 6.50 usec
TE 297.3 K
D1 2.00000000 sec
D11 0.03000000 sec
TD0 1

======== CHANNEL f1 ========
SFO1 376.4607162 MHz
NUC1 19F
P1 14.70 usec
PLW1 15.99600029 W

======== CHANNEL f2 ========
SFO2 400.1316005 MHz
NUC2 1H
CPDPRG[2 waltz16
PCPD2 90.00 usec
PLW2 11.99499989 W
PLW12 0.34213999 W
PLW13 0.27713001 W

F2 - Processing parameters
SI 32768
SF 376.4983660 MHz
WDW EM
SSB 0
LB 1.00 Hz
GB 0
PC 1.40

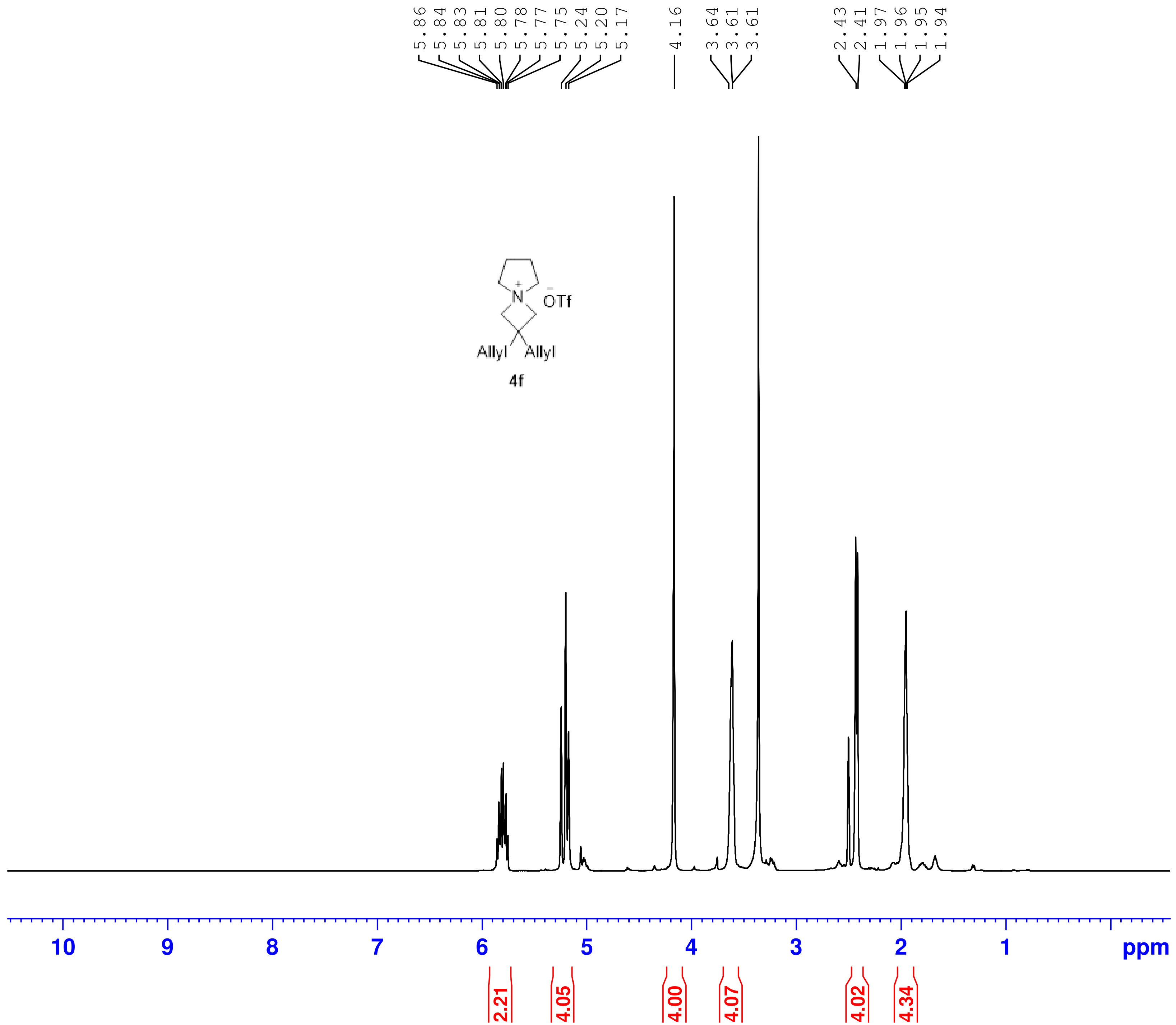

Current Data Parameters
NAME zmh-syh-14
EXPNO 13
PROCNO 1

F2 - Acquisition Parameters
Date_ 20230827
Time 20.17 h
INSTRUM AvanceNeo 400MHz
PROBHD Z163739_0629 (
PULPROG zg30
TD 65536
SOLVENT DMSO
NS 8
DS 2
SWH 8196.722 Hz
FIDRES 0.250144 Hz
AQ 3.9976959 sec
RG 101
DW 61.000 usec
DE 13.89 usec
TE 297.1 K
D1 1.00000000 sec
TD0 1
SFO1 400.1824711 MHz
NUC1 1H
P0 2.67 usec
P1 8.00 usec
PLW1 21.26700020 W

F2 - Processing parameters
SI 65536
SF 400.1800025 MHz
WDW EM
SSB 0
LB 0.30 Hz
GB 0
PC 1.00

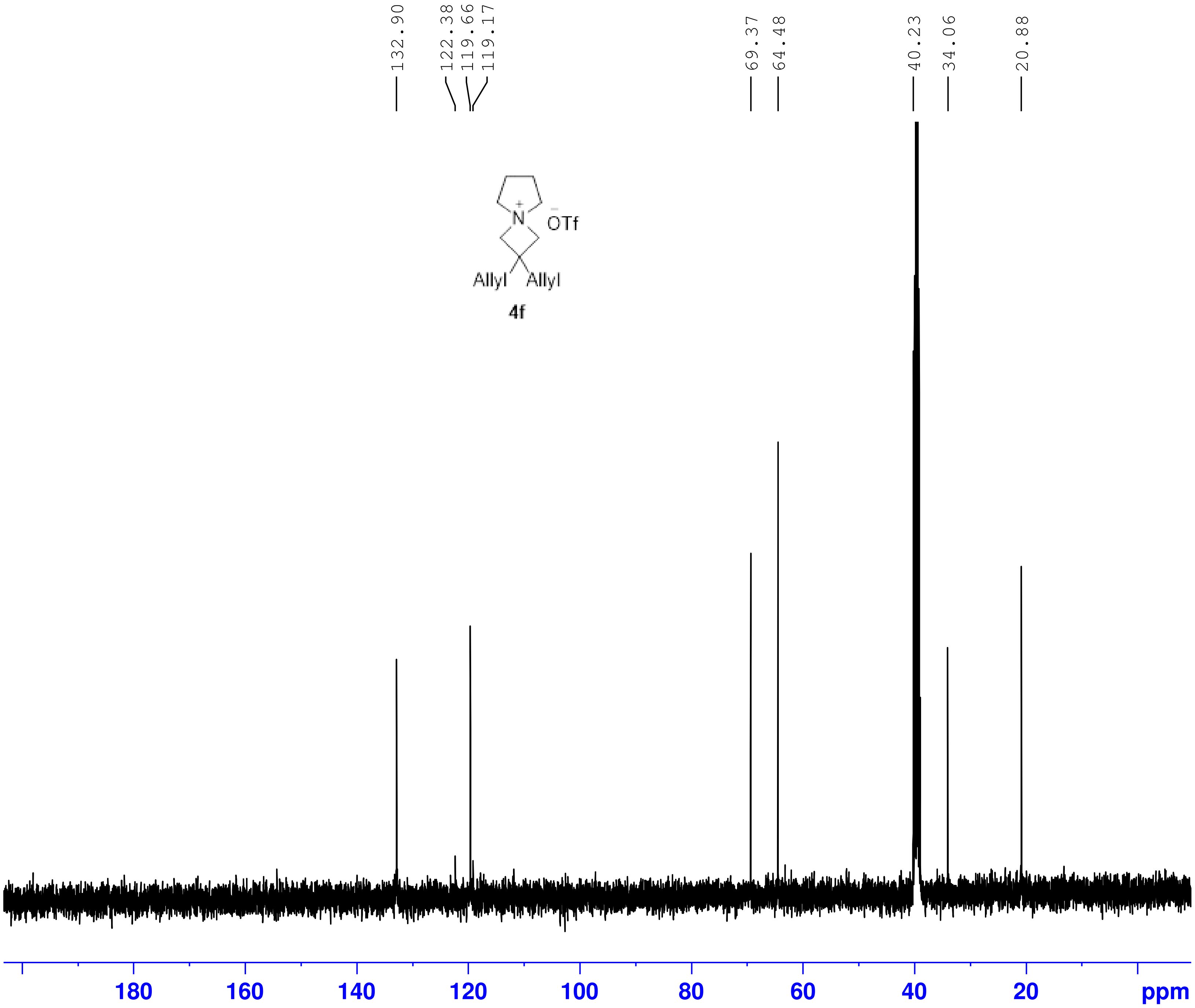

Current Data Parameters
NAME zmh-syh-14
EXPNO 14
PROCNO 1

F2 - Acquisition Parameters
Date_ 20230827
Time 20.21 h
INSTRUM AvanceNeo 400MHz
PROBHD Z163739_0629 (
PULPROG zgpg30
TD 65536
SOLVENT DMSO
NS 36
DS 4
SWH 23809.523 Hz
FIDRES 0.726609 Hz
AQ 1.3762560 sec
RG 10
DW 21.000 usec
DE 6.50 usec
TE 297.5 K
D1 2.00000000 sec
D11 0.03000000 sec
TD0 1
SFO1 100.6354036 MHz
NUC1 13C
P0 2.67 usec
P1 8.00 usec
PLW1 85.25399780 W
SFO2 400.1816007 MHz
NUC2 1H
CPDPRG[2 waltz65
PCPD2 90.00 usec
PLW2 21.26700020 W
PLW12 0.16802999 W
PLW13 0.08452000 W

F2 - Processing parameters
SI 32768
SF 100.6253769 MHz
WDW EM
SSB 0
LB 1.00 Hz
GB 0
PC 1.40

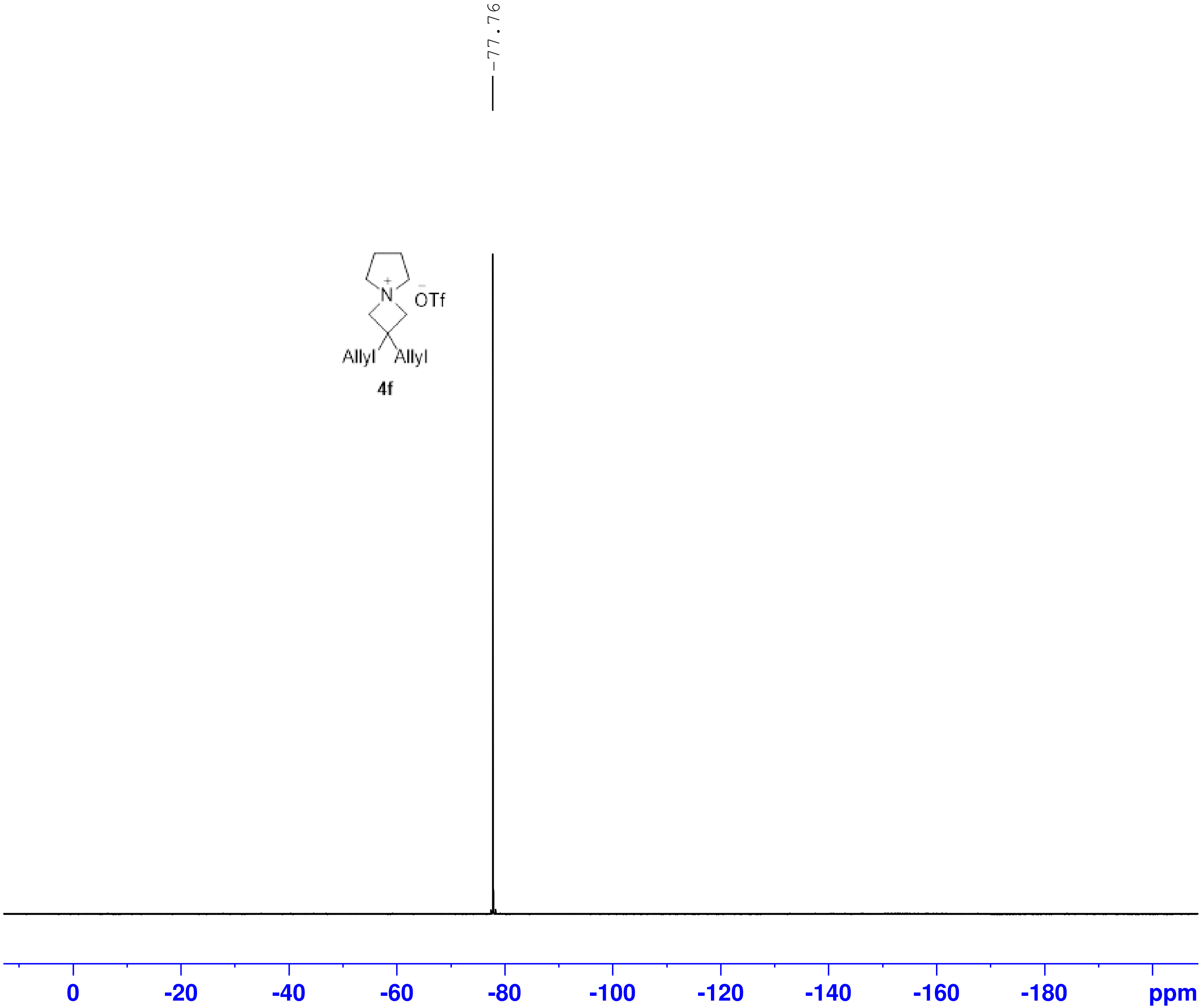

4f

Current Data Parameters
NAME zmh-syh-14
EXPNO 15
PROCNO 1

F2 - Acquisition Parameters
Date_ 20230827
Time 20.23 h
INSTRUM AvanceNeo 400MHz
PROBHD Z163739_0629 (
PULPROG zgig
TD 131072
SOLVENT DMSO
NS 8
DS 4
SWH 90909.094 Hz
FIDRES 1.387163 Hz
AQ 0.7208960 sec
RG 101
DW 5.500 usec
DE 6.50 usec
TE 297.3 K
D1 1.00000000 sec
D11 0.03000000 sec
TD0 1
SFO1 376.5077587 MHz
NUC1 19F
P1 12.00 usec
PLW1 33.72800064 W
SFO2 400.1816007 MHz
NUC2 1H
CPDPRG[2 waltz16
PCPD2 90.00 usec
PLW2 21.26700020 W
PLW12 0.16802999 W

F2 - Processing parameters
SI 65536
SF 376.5454132 MHz
WDW EM
SSB 0
LB 0.30 Hz
GB 0
PC 1.00

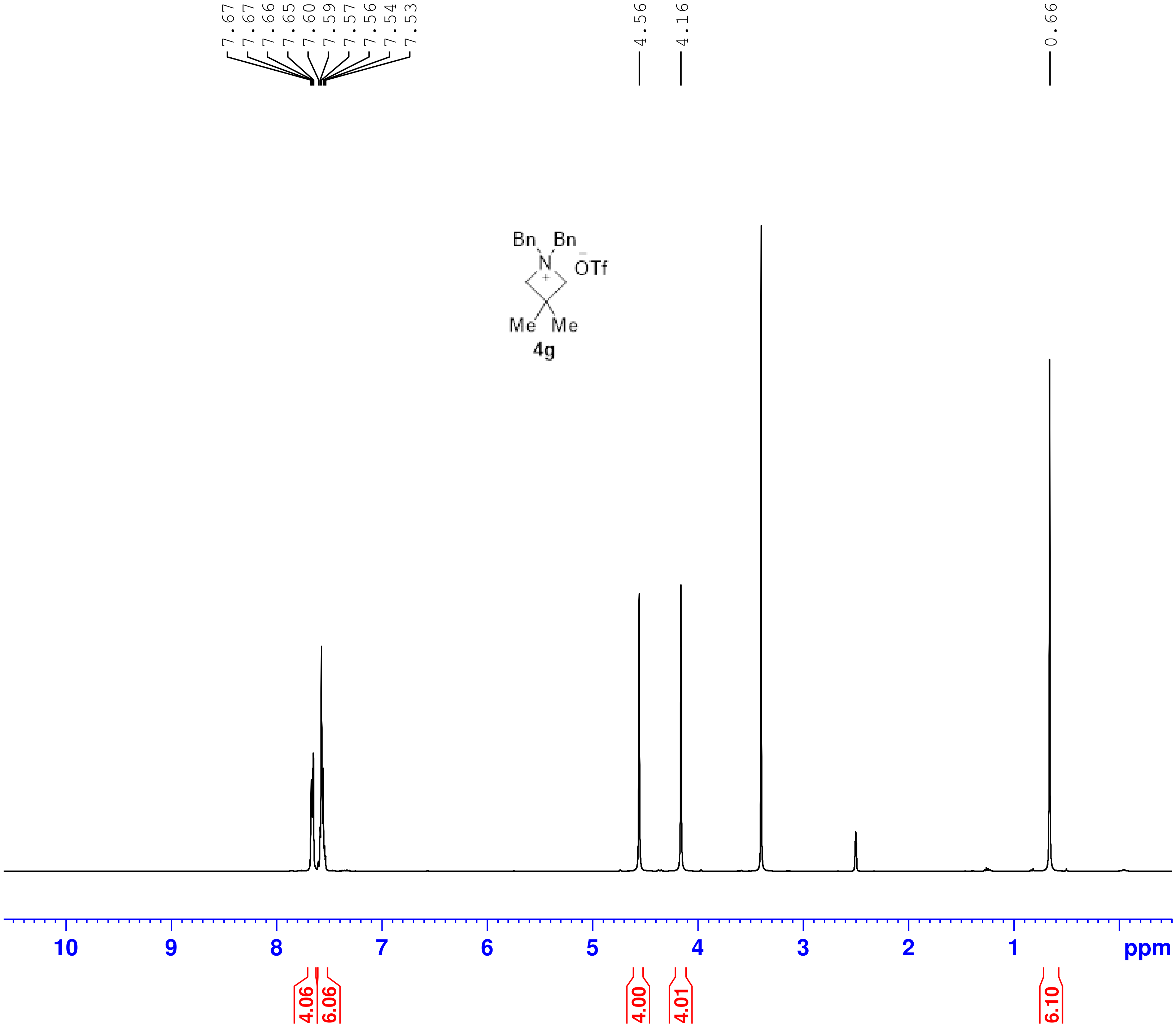


```
Current Data Parameters
NAME             zmh-syh-16
EXPNO                     1
PROCNO                    1

F2 - Acquisition Parameters
Date_              20230830
Time                  10.44
INSTRUM               spect
PROBHD     5 mm PABBO BB/
PULPROG                zg30
TD                    65536
SOLVENT                DMSO
NS                        8
DS                        2
SWH                8012.820 Hz
FIDRES             0.122266 Hz
AQ                4.0894465 sec
RG                    27.78
DW                   62.400 usec
DE                     6.50 usec
TE                    296.3 K
D1               1.00000000 sec
TD0                       1

======== CHANNEL f1 ========
SFO1            400.1324710 MHz
NUC1                     1H
P1                    14.50 usec
PLW1            11.99499989 W

F2 - Processing parameters
SI                    65536
SF              400.1300031 MHz
WDW                      EM
SSB                       0
LB                     0.30 Hz
GB                        0
PC                     1.00
```

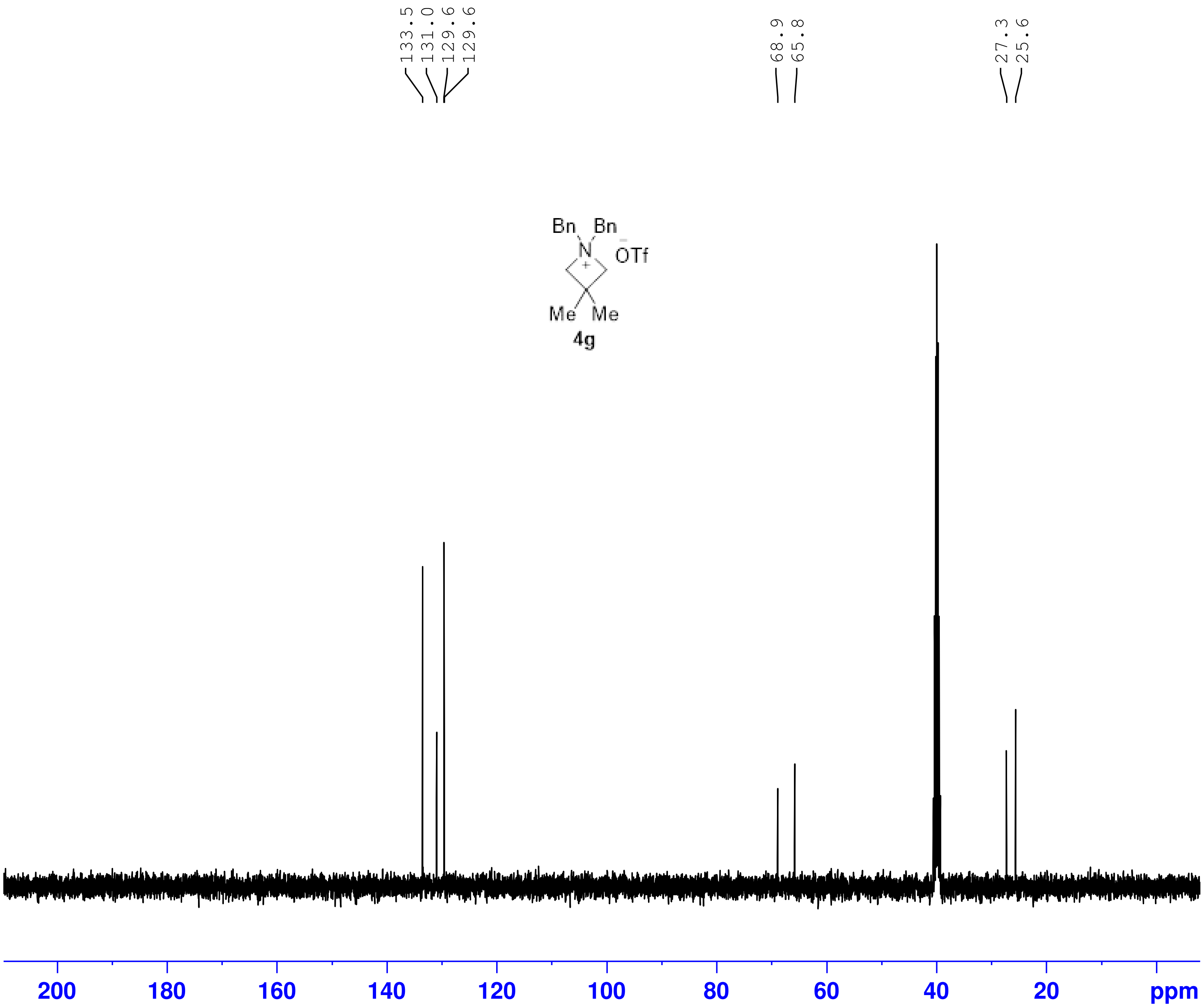

Current Data Parameters
NAME zmh-syh-16
EXPNO 2
PROCNO 1

F2 - Acquisition Parameters
Date_ 20230830
Time 10.46
INSTRUM spect
PROBHD 5 mm PABBO BB/
PULPROG zgpg30
TD 65536
SOLVENT DMSO
NS 37
DS 2
SWH 24038.461 Hz
FIDRES 0.366798 Hz
AQ 1.3631488 sec
RG 196.92
DW 20.800 usec
DE 6.50 usec
TE 296.5 K
D1 2.00000000 sec
D11 0.03000000 sec
TD0 1

======== CHANNEL f1 ========
SFO1 100.6228298 MHz
NUC1 13C
P1 9.70 usec
PLW1 46.98899841 W

======== CHANNEL f2 ========
SFO2 400.1316005 MHz
NUC2 1H
CPDPRG[2 waltz16
PCPD2 90.00 usec
PLW2 11.99499989 W
PLW12 0.34213999 W
PLW13 0.27713001 W

F2 - Processing parameters
SI 32768
SF 100.6127690 MHz
WDW EM
SSB 0
LB 1.00 Hz
GB 0
PC 1.40

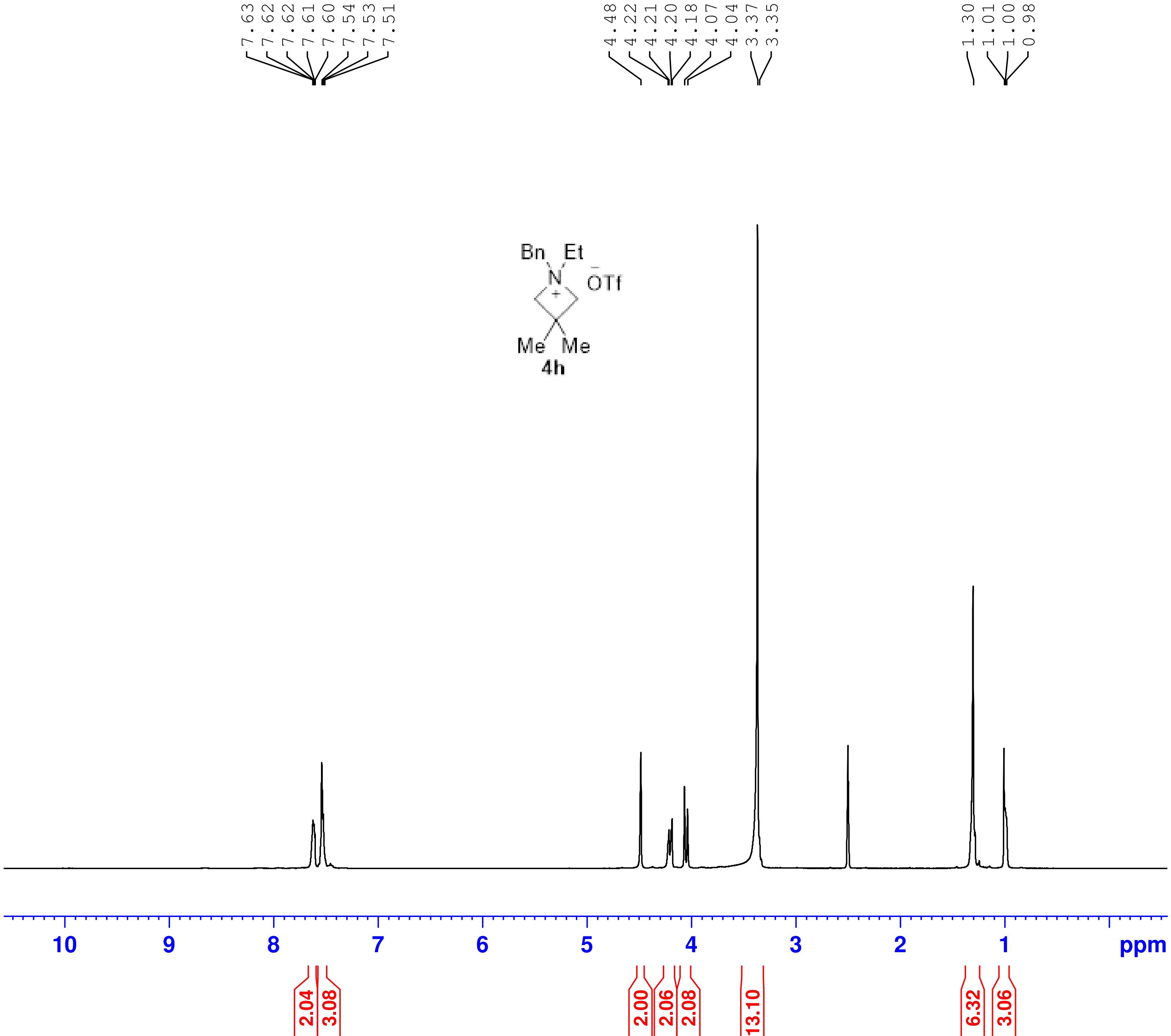

4h

Current Data Parameters
NAME zmh-syh-19-c
EXPNO 1
PROCNO 1

F2 - Acquisition Parameters
Date_ 20230827
Time 19.30
INSTRUM spect
PROBHD 5 mm PABBO BB/
PULPROG zg30
TD 65536
SOLVENT DMSO
NS 8
DS 2
SWH 8012.820 Hz
FIDRES 0.122266 Hz
AQ 4.0894465 sec
RG 62.93
DW 62.400 usec
DE 6.50 usec
TE 296.4 K
D1 1.00000000 sec
TD0 1

======== CHANNEL f1 ========
SFO1 400.1324710 MHz
NUC1 1H
P1 14.50 usec
PLW1 11.99499989 W

F2 - Processing parameters
SI 65536
SF 400.1300029 MHz
WDW EM
SSB 0
LB 0.30 Hz
GB 0
PC 1.00

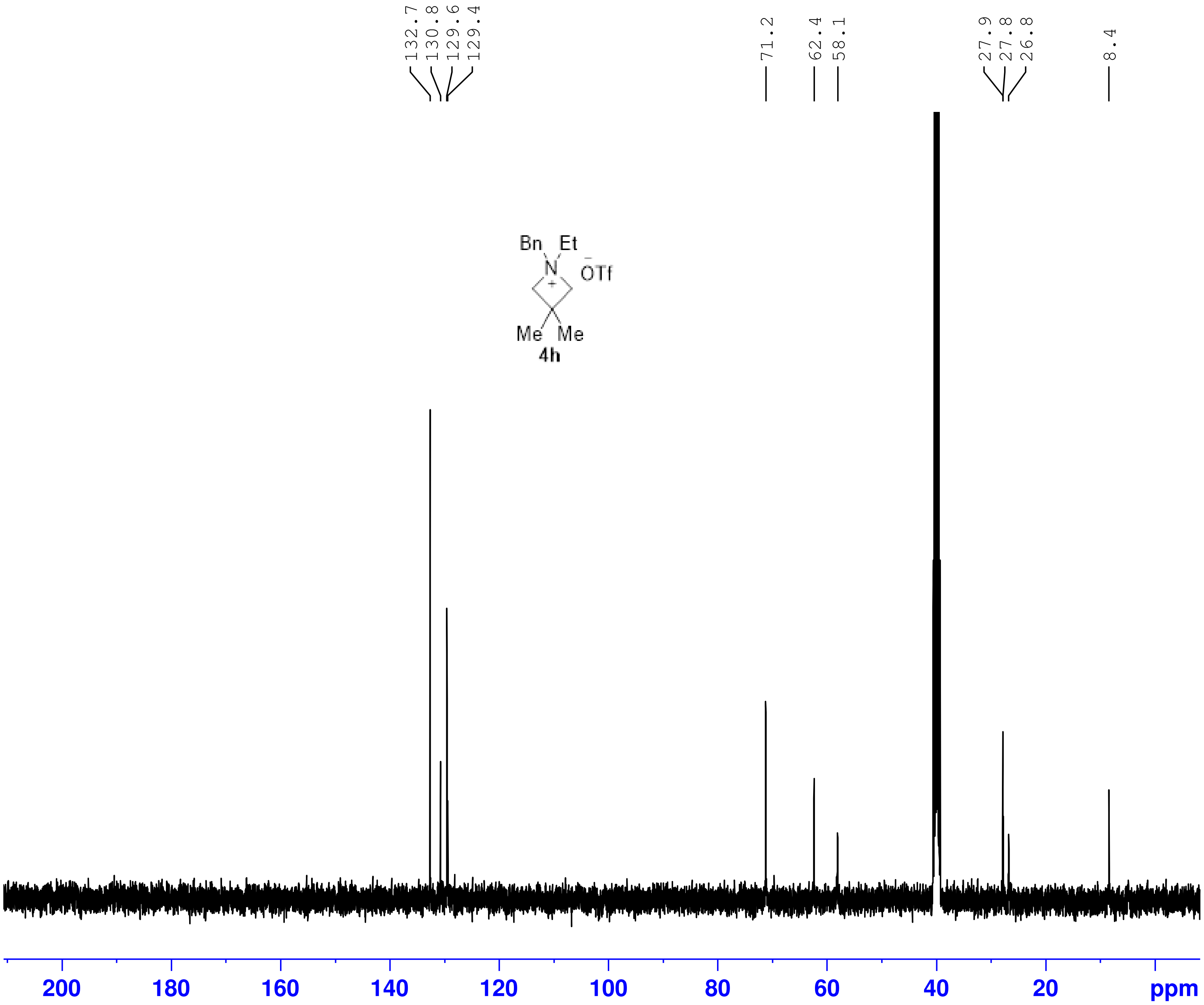

Current Data Parameters
NAME zmh-syh-19-c
EXPNO 2
PROCNO 1

F2 - Acquisition Parameters
Date_ 20230827
Time 19.33
INSTRUM spect
PROBHD 5 mm PABBO BB/
PULPROG zgpg30
TD 65536
SOLVENT DMSO
NS 202
DS 2
SWH 24038.461 Hz
FIDRES 0.366798 Hz
AQ 1.3631488 sec
RG 196.92
DW 20.800 usec
DE 6.50 usec
TE 297.1 K
D1 2.00000000 sec
D11 0.03000000 sec
TD0 1

======== CHANNEL f1 ========
SFO1 100.6228298 MHz
NUC1 13C
P1 9.70 usec
PLW1 46.98899841 W

======== CHANNEL f2 ========
SFO2 400.1316005 MHz
NUC2 1H
CPDPRG[2 waltz16
PCPD2 90.00 usec
PLW2 11.99499989 W
PLW12 0.34213999 W
PLW13 0.27713001 W

F2 - Processing parameters
SI 32768
SF 100.6127690 MHz
WDW EM
SSB 0
LB 1.00 Hz
GB 0
PC 1.40

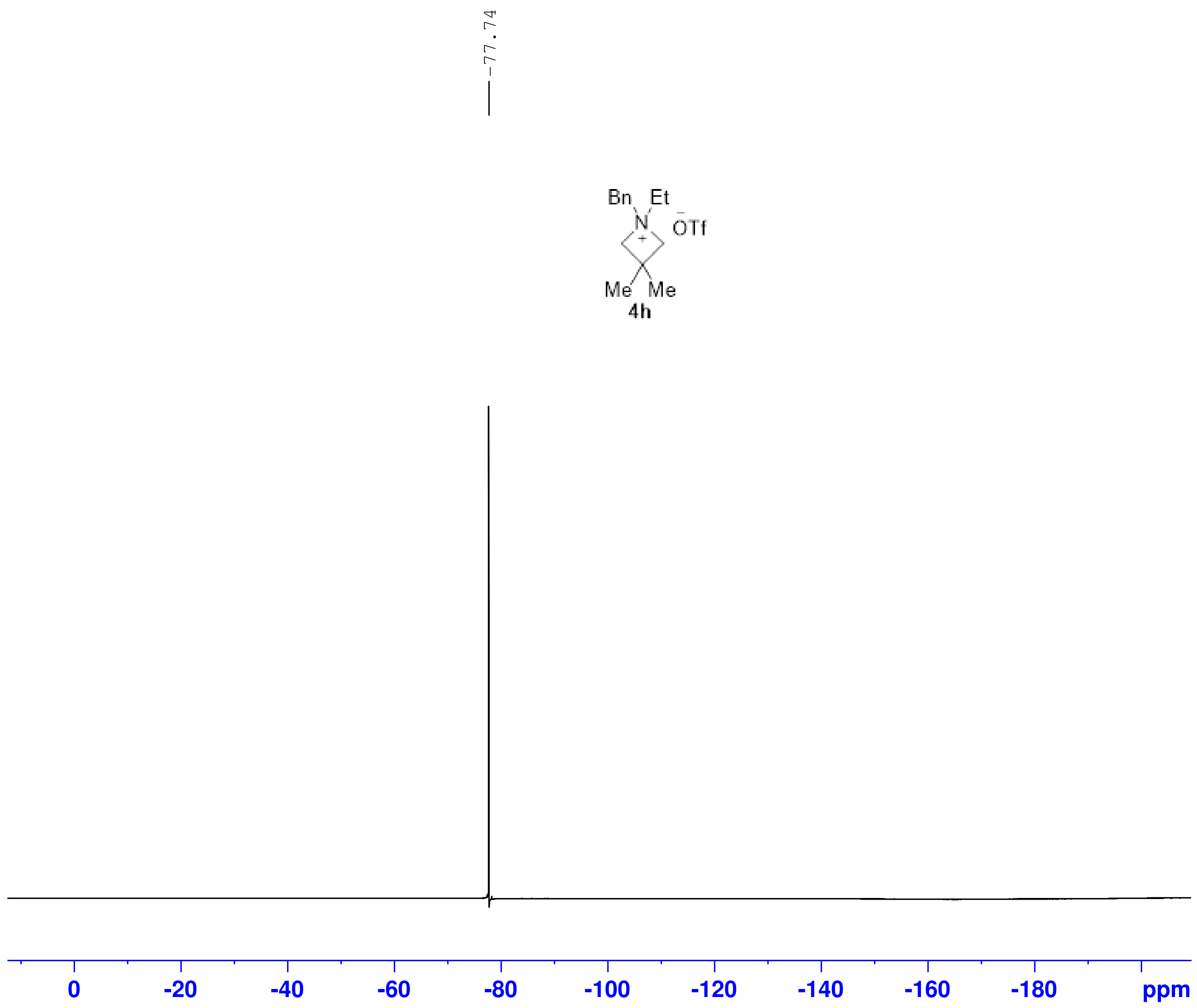

Current Data Parameters

NAME	zmh-syh-19-c	
EXPNO	3	
PROCNO	1	

F2 - Acquisition Parameters

Date_	20230827	
Time	19.46	
INSTRUM	spect	
PROBHD	5 mm PABBO BB/	
PULPROG	zgpg30	
TD	65536	
SOLVENT	DMSO	
NS	64	
DS	2	
SWH	93750.000	Hz
FIDRES	1.430511	Hz
AQ	0.3495253	sec
RG	196.92	
DW	5.333	usec
DE	6.50	usec
TE	297.4	K
D1	2.00000000	sec
D11	0.03000000	sec
TD0	1	

======== CHANNEL f1 ========

SFO1	376.4607162	MHz
NUC1	19F	
P1	14.70	usec
PLW1	15.99600029	W

======== CHANNEL f2 ========

SFO2	400.1316005	MHz
NUC2	1H	
CPDPRG[2	waltz16	
PCPD2	90.00	usec
PLW2	11.99499989	W
PLW12	0.34213999	W
PLW13	0.27713001	W

F2 - Processing parameters

SI	32768	
SF	376.4983660	MHz
WDW	EM	
SSB	0	
LB	1.00	Hz
GB	0	
PC	1.40	

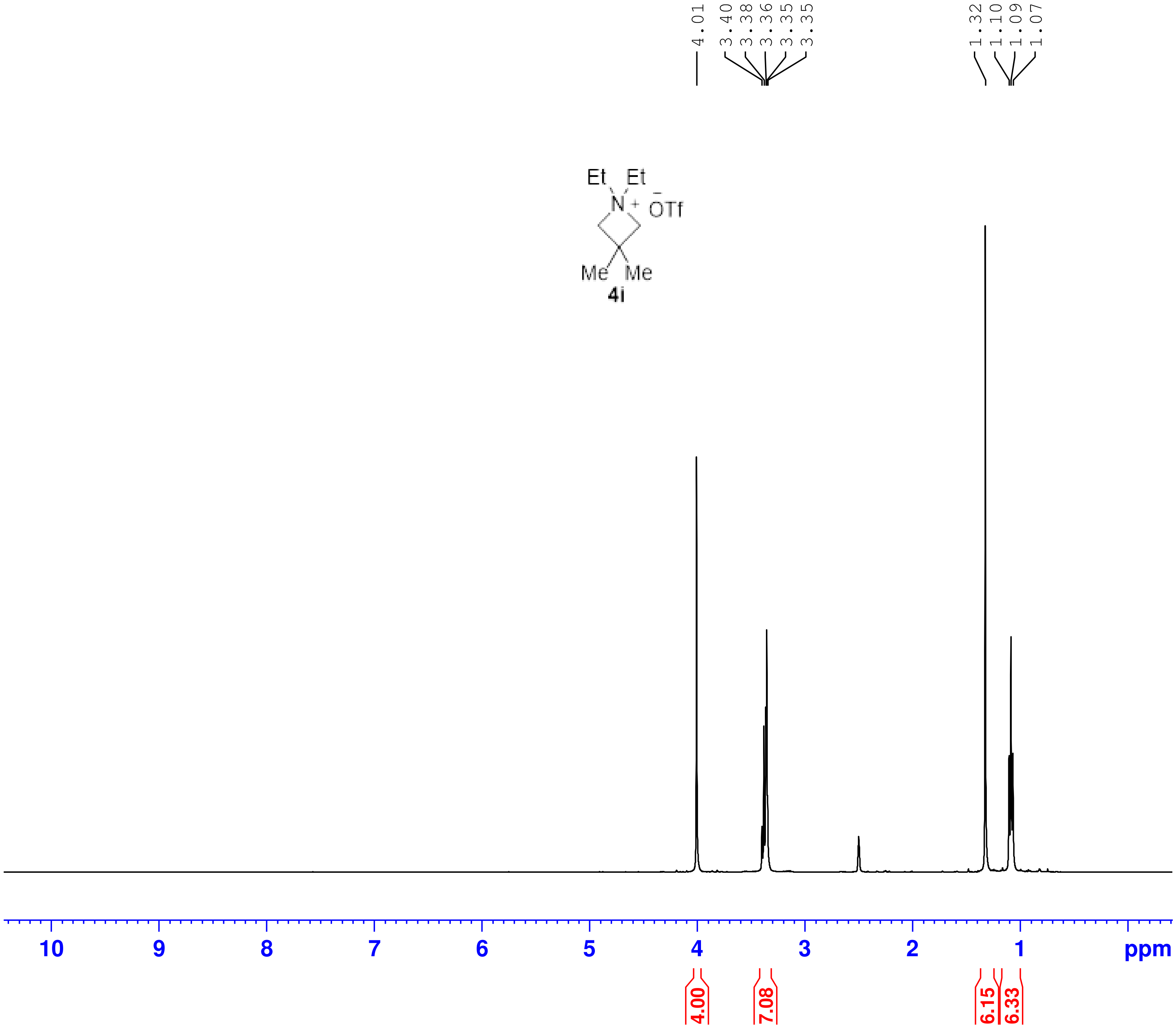

Current Data Parameters
NAME zmh-syh-7
EXPNO 2
PROCNO 1

F2 - Acquisition Parameters
Date_ 20230215
Time 13.47
INSTRUM spect
PROBHD 5 mm DUL 13C-1
PULPROG zg30
TD 65536
SOLVENT DMSO
NS 13
DS 2
SWH 8223.685 Hz
FIDRES 0.125483 Hz
AQ 3.9845889 sec
RG 161
DW 60.800 usec
DE 6.00 usec
TE 293.0 K
D1 1.00000000 sec
TD0 1

======== CHANNEL f1 ========
NUC1 1H
P1 15.80 usec
PL1 -1.00 dB
PL1W 12.17476940 W
SFO1 400.1324710 MHz

F2 - Processing parameters
SI 32768
SF 400.1300034 MHz
WDW EM
SSB 0
LB 0.30 Hz
GB 0
PC 1.00

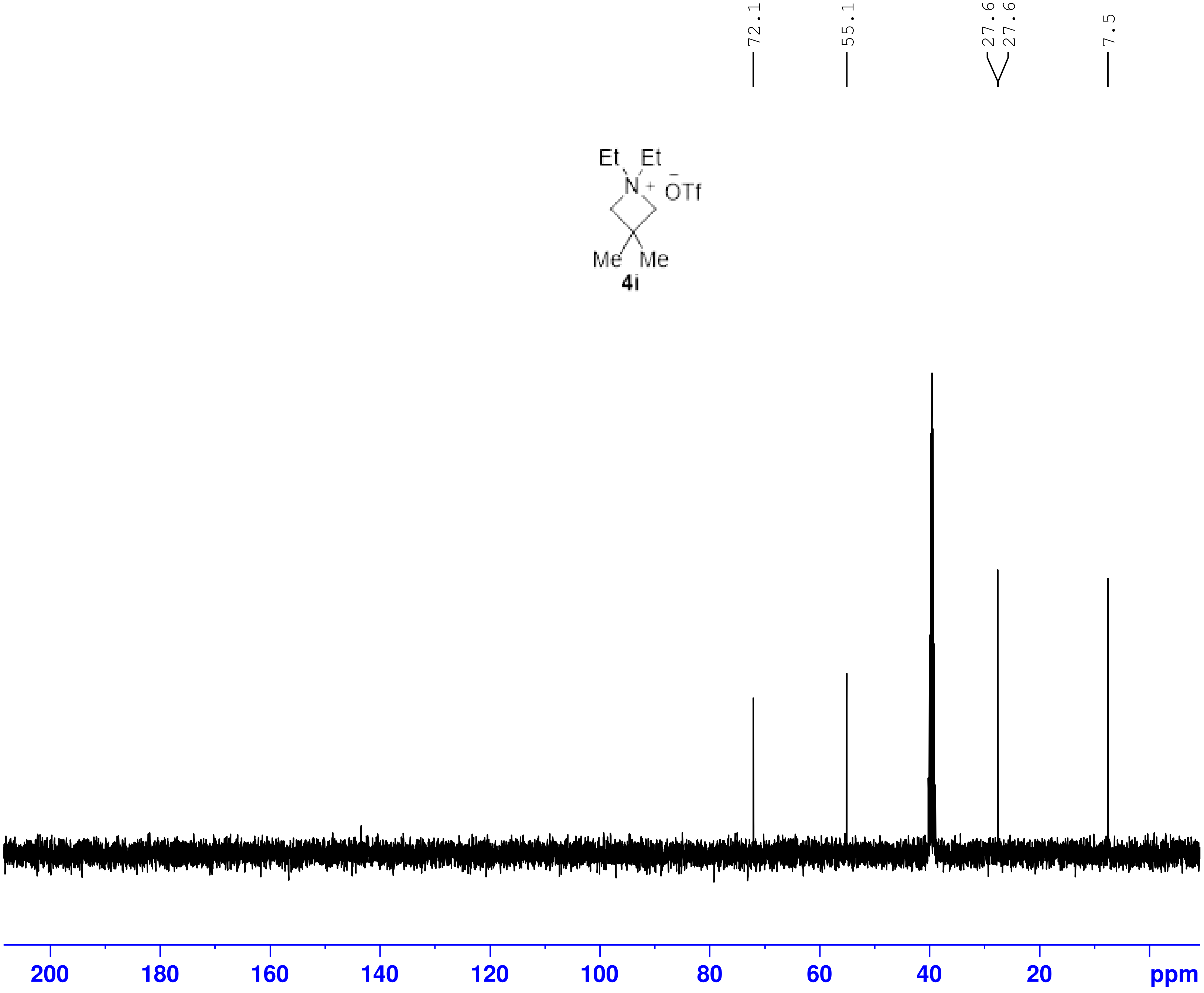


```
Current Data Parameters
NAME            zmh-syh-7
EXPNO                   1
PROCNO                  1

F2 - Acquisition Parameters
Date_           20230215
Time               13.50
INSTRUM            spect
PROBHD    5 mm DUL 13C-1
PULPROG          zgpg30
TD                 65536
SOLVENT             DMSO
NS                    63
DS                     1
SWH            24038.461 Hz
FIDRES          0.366798 Hz
AQ             1.3631488 sec
RG                  2050
DW                20.800 usec
DE                  6.00 usec
TE                 293.1 K
D1            2.00000000 sec
D11           0.03000000 sec
TD0                    1

======== CHANNEL f1 ========
NUC1                 13C
P1                 40.00 usec
PL1                -3.00 dB
PL1W         60.64365387 W
SFO1         100.6228298 MHz

======== CHANNEL f2 ========
CPDPRG[2         waltz16
NUC2                  1H
PCPD2              80.00 usec
PL2                -1.00 dB
PL12               14.39 dB
PL13               18.00 dB
PL2W         12.17476940 W
PL12W         0.35193357 W
PL13W         0.15327126 W
SFO2         400.1316005 MHz

F2 - Processing parameters
SI                 32768
SF           100.6128029 MHz
WDW                   EM
SSB                    0
LB                  1.00 Hz
GB                     0
PC                  1.40
```

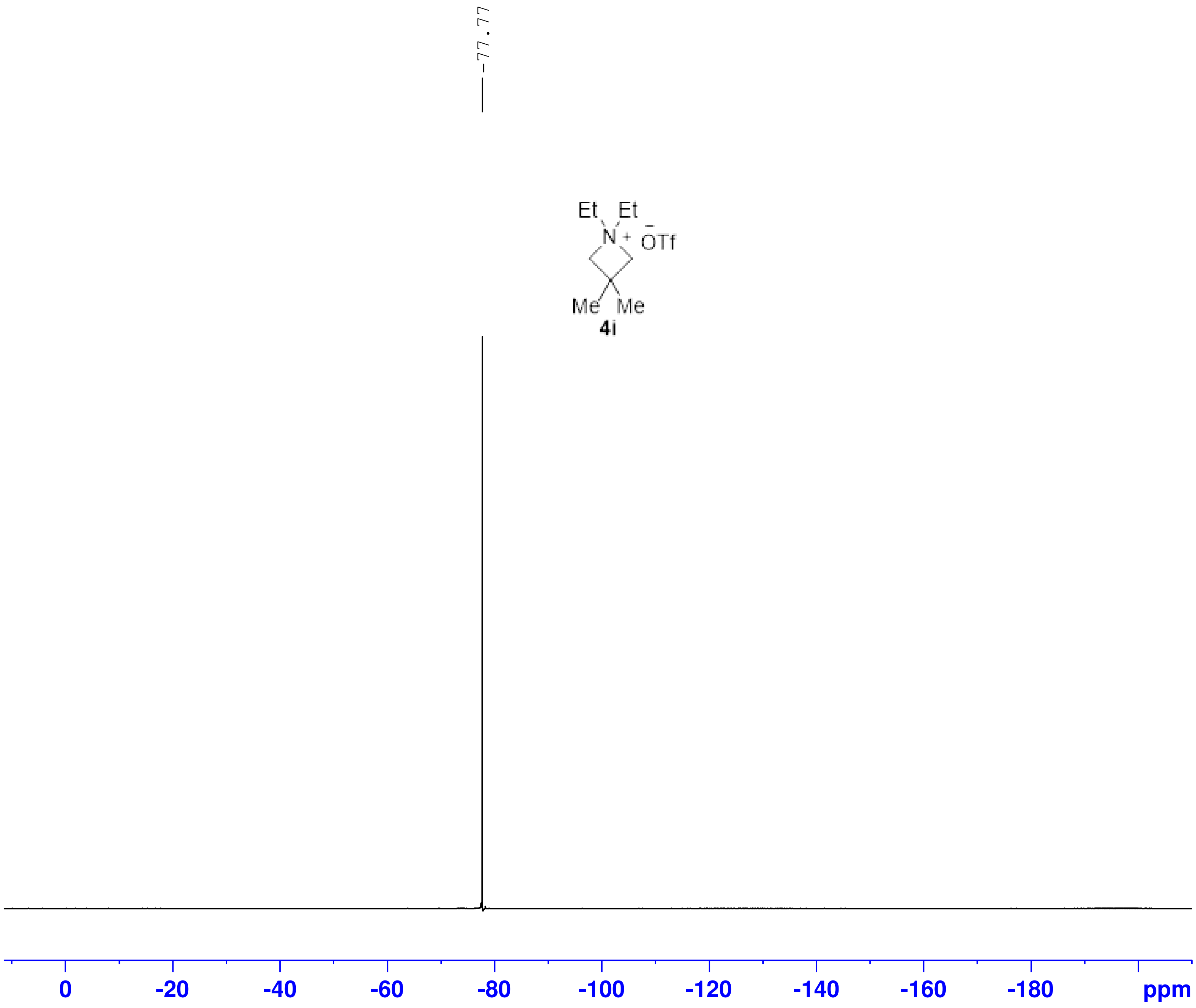

Current Data Parameters
NAME zmh-syh-7
EXPNO 1
PROCNO 1

F2 - Acquisition Parameters
Date_ 20230215
Time 18.46
INSTRUM spect
PROBHD 5 mm PABBO BB/
PULPROG zgpg30
TD 65536
SOLVENT DMSO
NS 16
DS 2
SWH 93750.000 Hz
FIDRES 1.430511 Hz
AQ 0.3495253 sec
RG 196.92
DW 5.333 usec
DE 6.50 usec
TE 294.4 K
D1 2.00000000 sec
D11 0.03000000 sec
TD0 1

======== CHANNEL f1 ========
SFO1 376.4607162 MHz
NUC1 19F
P1 14.70 usec
PLW1 15.99600029 W

======== CHANNEL f2 ========
SFO2 400.1316005 MHz
NUC2 1H
CPDPRG[2 waltz16
PCPD2 90.00 usec
PLW2 11.99499989 W
PLW12 0.34213999 W
PLW13 0.27713001 W

F2 - Processing parameters
SI 32768
SF 376.4983660 MHz
WDW EM
SSB 0
LB 1.00 Hz
GB 0
PC 1.40

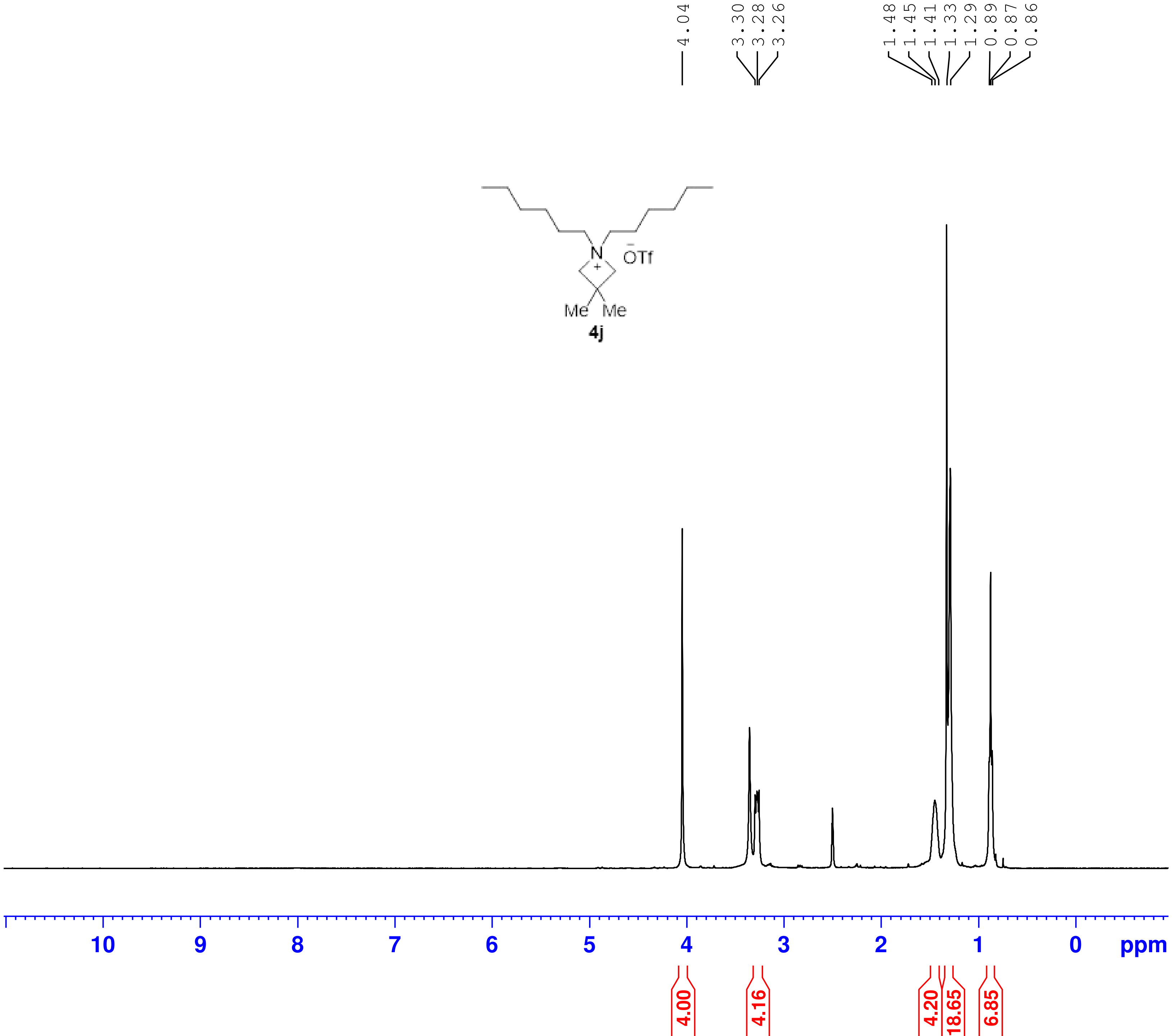

Current Data Parameters	
NAME	zmh-syh-1
EXPNO	1
PROCNO	1
F2 - Acquisition Parameters	
Date_	20230215
Time	12.55
INSTRUM	spect
PROBHD	5 mm DUL 13C-1
PULPROG	zg30
TD	65536
SOLVENT	DMSO
NS	16
DS	2
SWH	8223.685 Hz
FIDRES	0.125483 Hz
AQ	3.9845889 sec
RG	114
DW	60.800 usec
DE	6.00 usec
TE	292.9 K
D1	1.00000000 sec
TD0	1
======== CHANNEL f1 ========	
NUC1	1H
P1	15.80 usec
PL1	-1.00 dB
PL1W	12.17476940 W
SFO1	400.1324710 MHz
F2 - Processing parameters	
SI	32768
SF	400.1300036 MHz
WDW	EM
SSB	0
LB	0.30 Hz
GB	0
PC	1.00

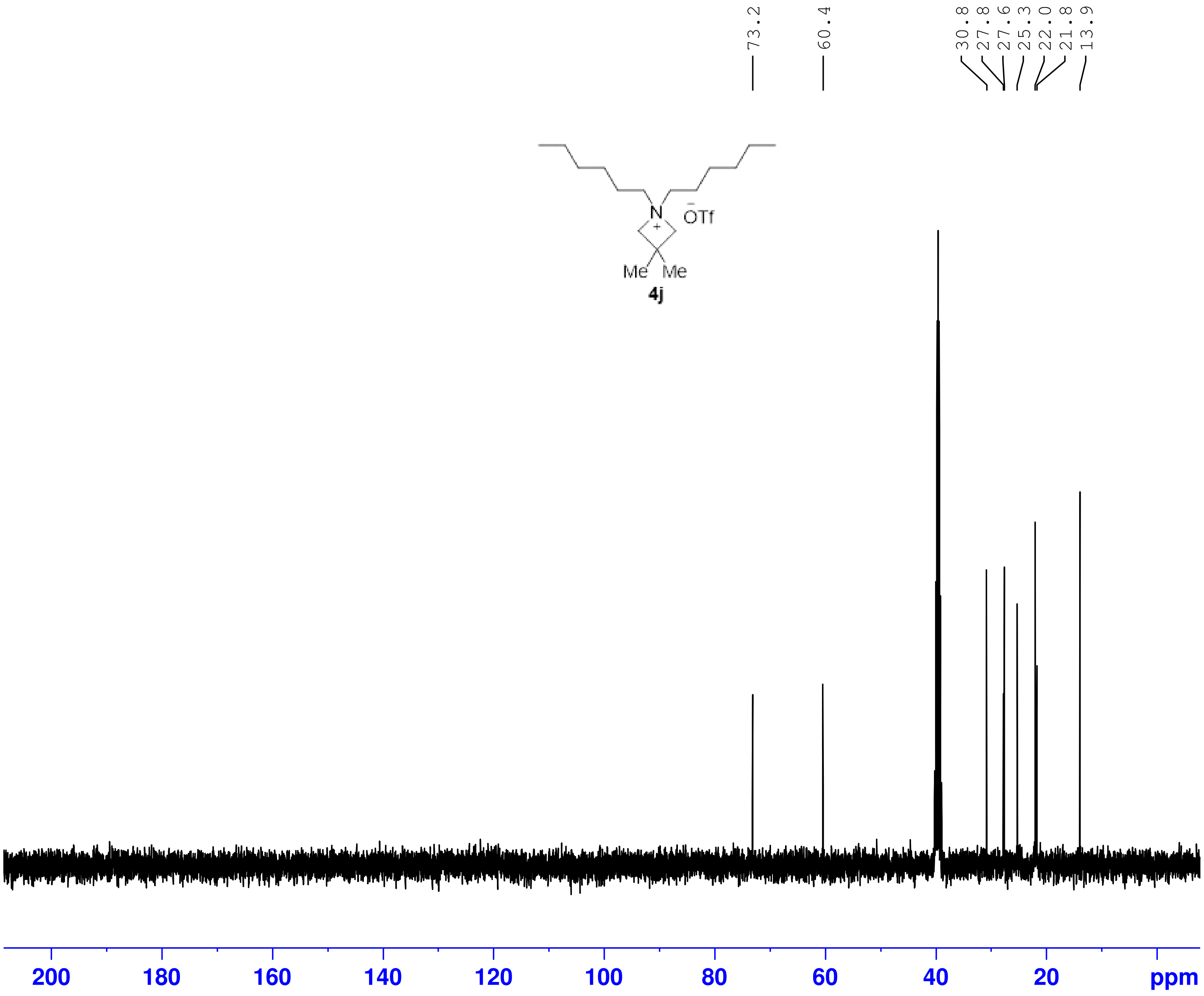

Current Data Parameters
NAME zmh-syh-1
EXPNO 2
PROCNO 1

F2 - Acquisition Parameters
Date_ 20230215
Time 12.57
INSTRUM spect
PROBHD 5 mm DUL 13C-1
PULPROG zgpg30
TD 65536
SOLVENT DMSO
NS 51
DS 1
SWH 24038.461 Hz
FIDRES 0.366798 Hz
AQ 1.3631488 sec
RG 2050
DW 20.800 usec
DE 6.00 usec
TE 292.9 K
D1 2.00000000 sec
D11 0.03000000 sec
TD0 1

======== CHANNEL f1 ========
NUC1 13C
P1 40.00 usec
PL1 -3.00 dB
PL1W 60.64365387 W
SFO1 100.6228298 MHz

======== CHANNEL f2 ========
CPDPRG[2 waltz16
NUC2 1H
PCPD2 80.00 usec
PL2 -1.00 dB
PL12 14.39 dB
PL13 18.00 dB
PL2W 12.17476940 W
PL12W 0.35193357 W
PL13W 0.15327126 W
SFO2 400.1316005 MHz

F2 - Processing parameters
SI 32768
SF 100.6128043 MHz
WDW EM
SSB 0
LB 1.00 Hz
GB 0
PC 1.40

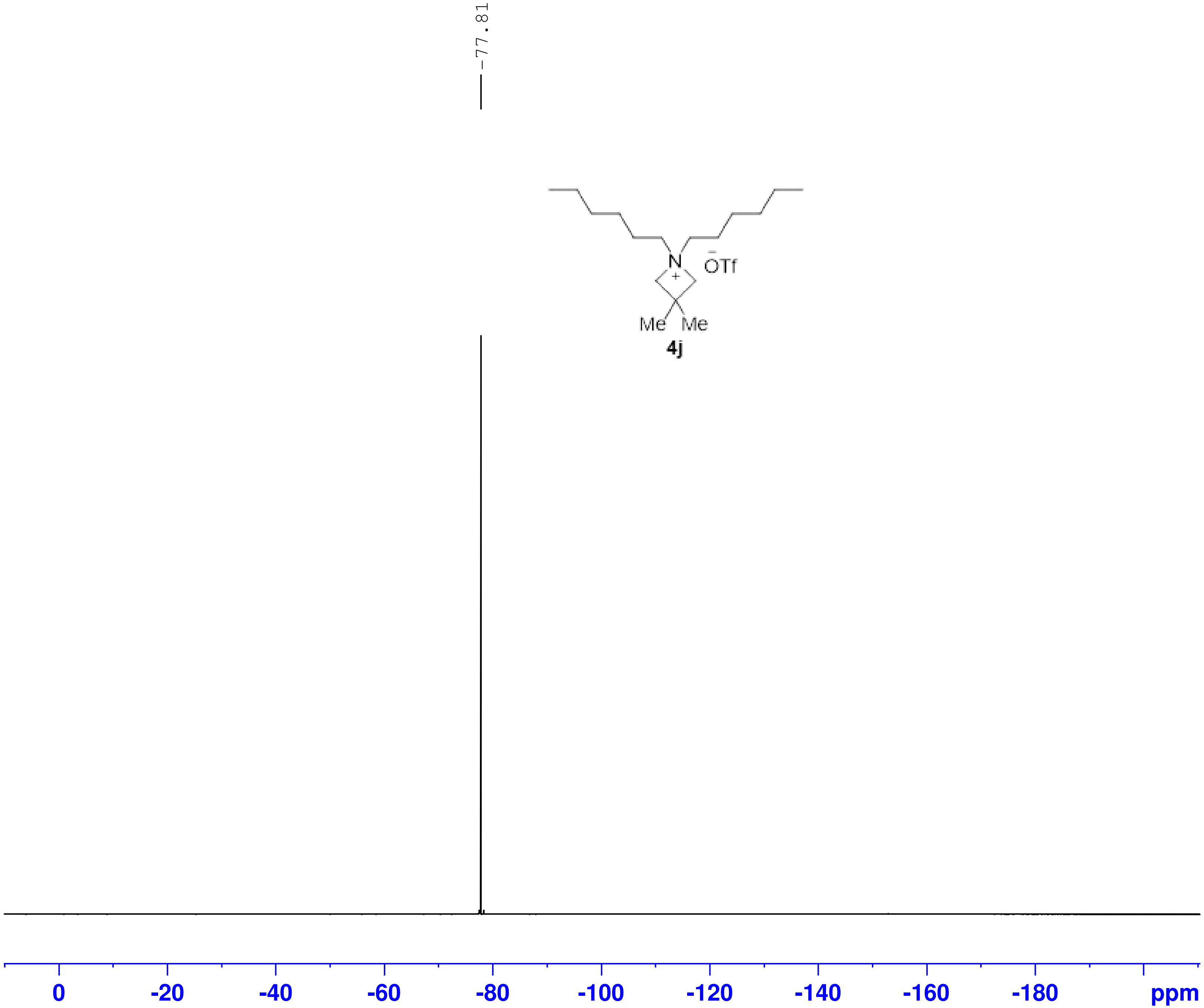

4j

Current Data Parameters
NAME zmh-syh-1
EXPNO 2
PROCNO 1

F2 - Acquisition Parameters
Date_ 20230215
Time 18.28
INSTRUM spect
PROBHD 5 mm PABBO BB/
PULPROG zgflqn
TD 131072
SOLVENT DMSO
NS 16
DS 4
SWH 89285.711 Hz
FIDRES 0.681196 Hz
AQ 0.7340032 sec
RG 196.92
DW 5.600 usec
DE 6.50 usec
TE 294.2 K
D1 1.00000000 sec
TD0 1

======== CHANNEL f1 ========
SFO1 376.4607164 MHz
NUC1 19F
P1 14.70 usec
PLW1 15.99600029 W

F2 - Processing parameters
SI 65536
SF 376.4983660 MHz
WDW EM
SSB 0
LB 0.30 Hz
GB 0
PC 1.00

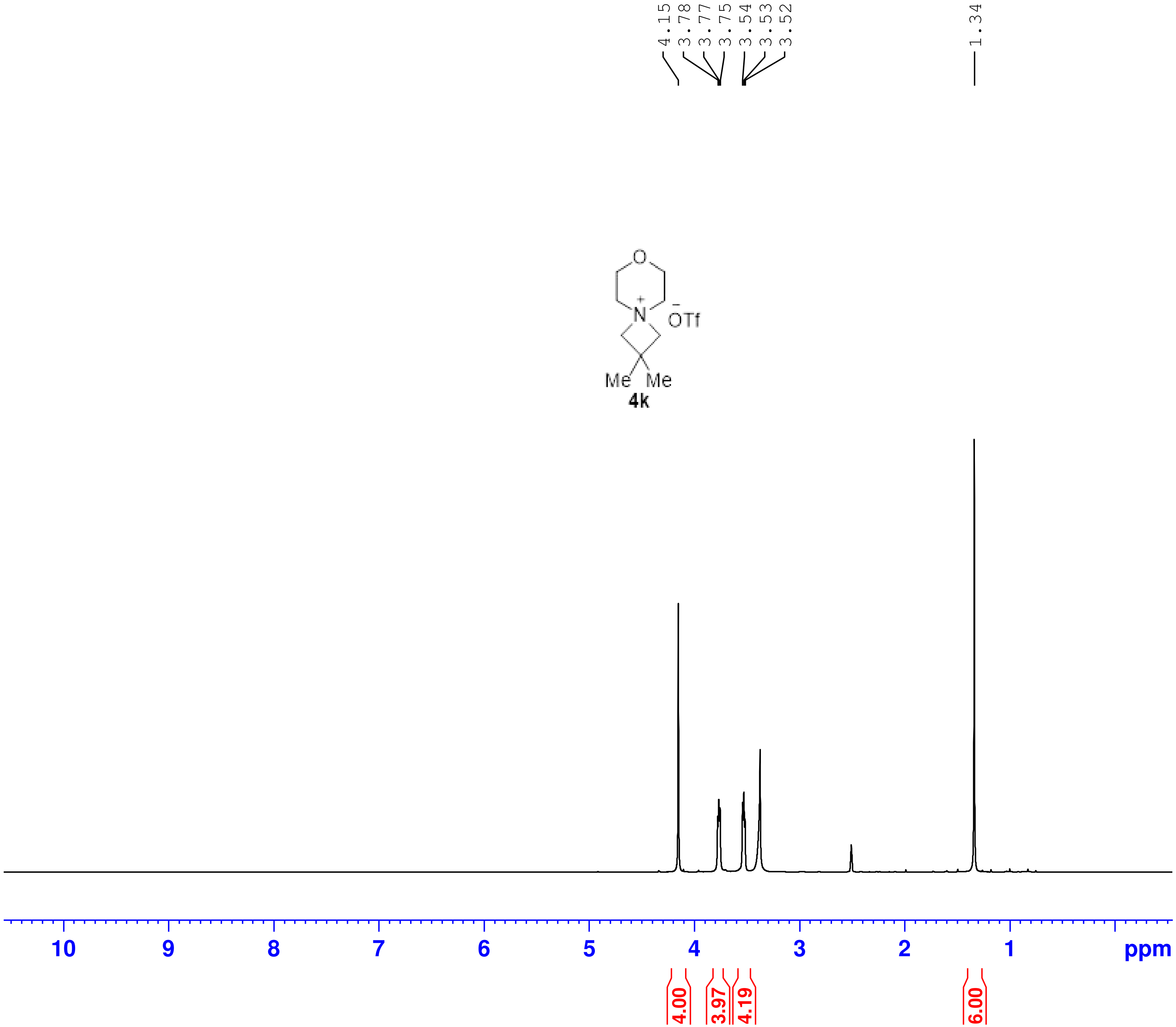

Current Data Parameters
NAME zmh-ml
EXPNO 1
PROCNO 1

F2 - Acquisition Parameters
Date_ 20230905
Time 15.13 h
INSTRUM AvanceNeo 400MHz
PROBHD Z163739_0629 (
PULPROG zg30
TD 65536
SOLVENT DMSO
NS 10
DS 2
SWH 8196.722 Hz
FIDRES 0.250144 Hz
AQ 3.9976959 sec
RG 71.8
DW 61.000 usec
DE 13.89 usec
TE 296.6 K
D1 1.00000000 sec
TD0 1
SFO1 400.1824711 MHz
NUC1 1H
P0 2.67 usec
P1 8.00 usec
PLW1 21.26700020 W

F2 - Processing parameters
SI 65536
SF 400.1800000 MHz
WDW EM
SSB 0
LB 0.30 Hz
GB 0
PC 1.00

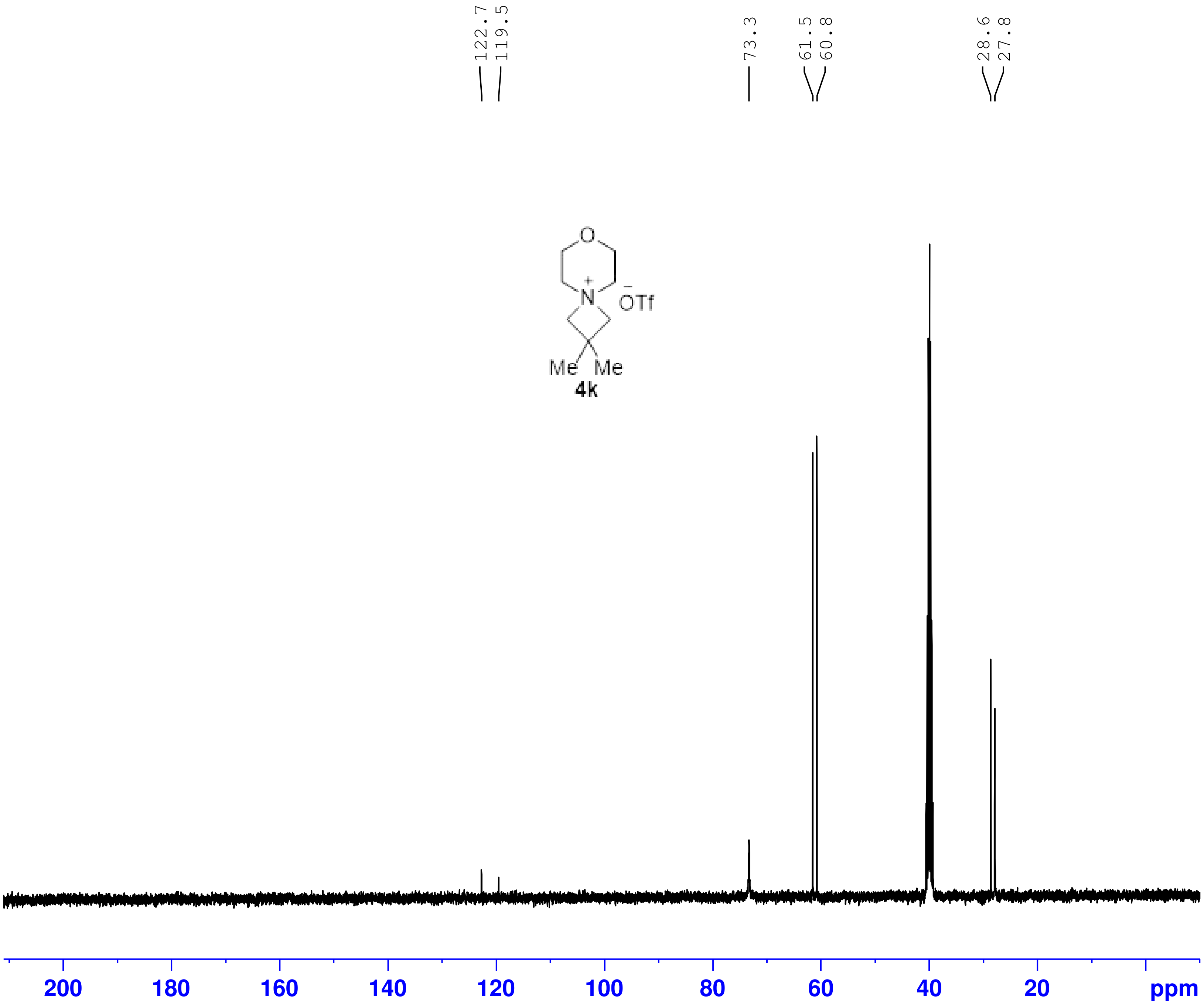

Current Data Parameters	
NAME	zmh-ml
EXPNO	3
PROCNO	1
F2 - Acquisition Parameters	
Date_	20230905
Time	15.27 h
INSTRUM	AvanceNeo 400MHz
PROBHD	Z163739_0629 (
PULPROG	zgpg30
TD	65536
SOLVENT	DMSO
NS	120
DS	4
SWH	23809.523 Hz
FIDRES	0.726609 Hz
AQ	1.3762560 sec
RG	20.2
DW	21.000 usec
DE	6.50 usec
TE	297.0 K
D1	2.00000000 sec
D11	0.03000000 sec
TD0	1
SFO1	100.6354036 MHz
NUC1	13C
P0	2.67 usec
P1	8.00 usec
PLW1	85.25399780 W
SFO2	400.1816007 MHz
NUC2	1H
CPDPRG[2	waltz65
PCPD2	90.00 usec
PLW2	21.26700020 W
PLW12	0.16802999 W
PLW13	0.08452000 W
F2 - Processing parameters	
SI	32768
SF	100.6253410 MHz
WDW	EM
SSB	0
LB	1.00 Hz
GB	0
PC	1.40

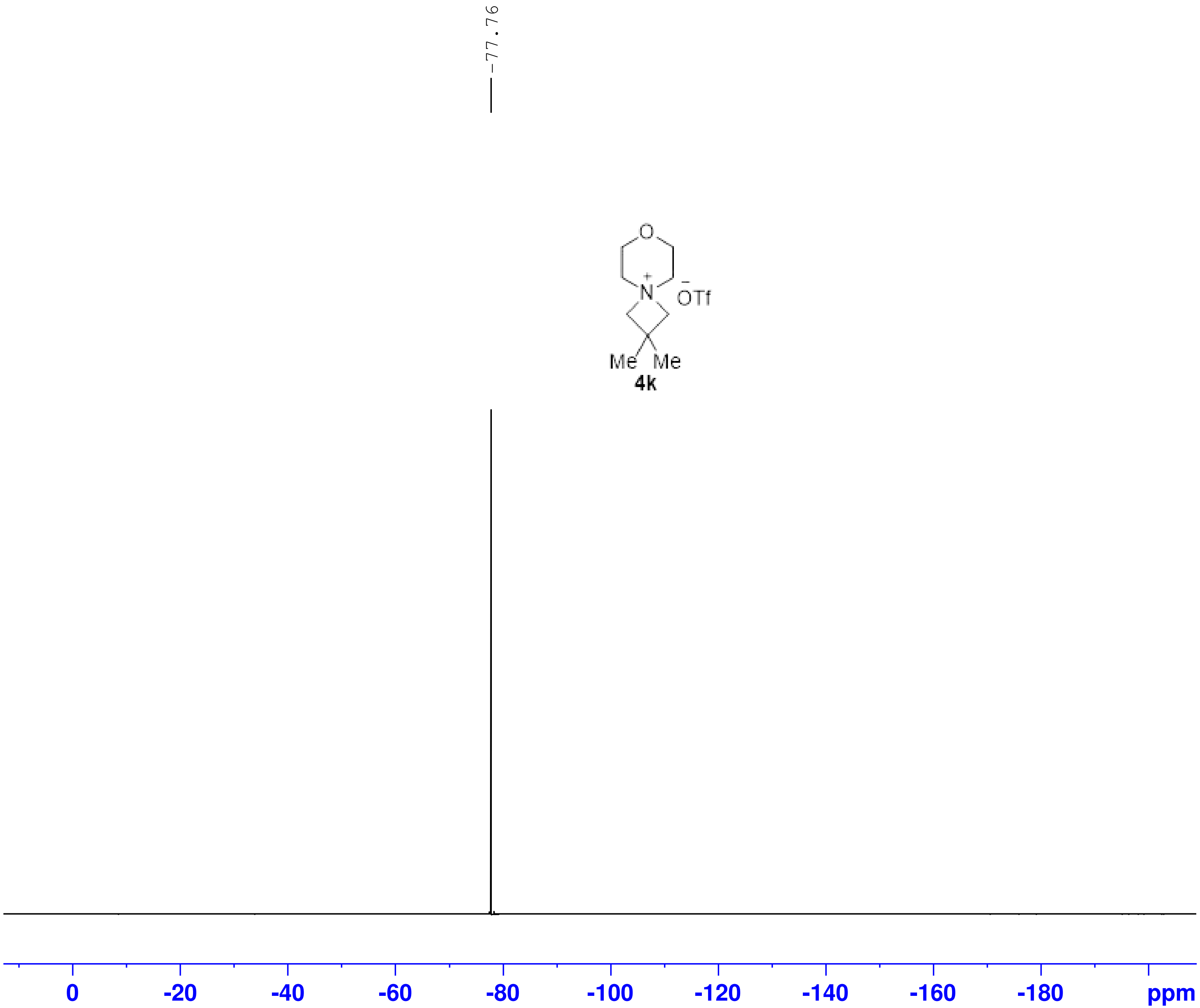

Current Data Parameters
NAME zmh-ml
EXPNO 2
PROCNO 1

F2 - Acquisition Parameters
Date_ 20230905
Time 15.16 h
INSTRUM AvanceNeo 400MHz
PROBHD Z163739_0629 (
PULPROG zgig
TD 131072
SOLVENT DMSO
NS 16
DS 4
SWH 90909.094 Hz
FIDRES 1.387163 Hz
AQ 0.7208960 sec
RG 101
DW 5.500 usec
DE 6.50 usec
TE 296.7 K
D1 1.00000000 sec
D11 0.03000000 sec
TD0 1
SFO1 376.5077587 MHz
NUC1 19F
P1 12.00 usec
PLW1 33.72800064 W
SFO2 400.1816007 MHz
NUC2 1H
CPDPRG[2 waltz16
PCPD2 90.00 usec
PLW2 21.26700020 W
PLW12 0.16802999 W

F2 - Processing parameters
SI 65536
SF 376.5454132 MHz
WDW EM
SSB 0
LB 0.30 Hz
GB 0
PC 1.00

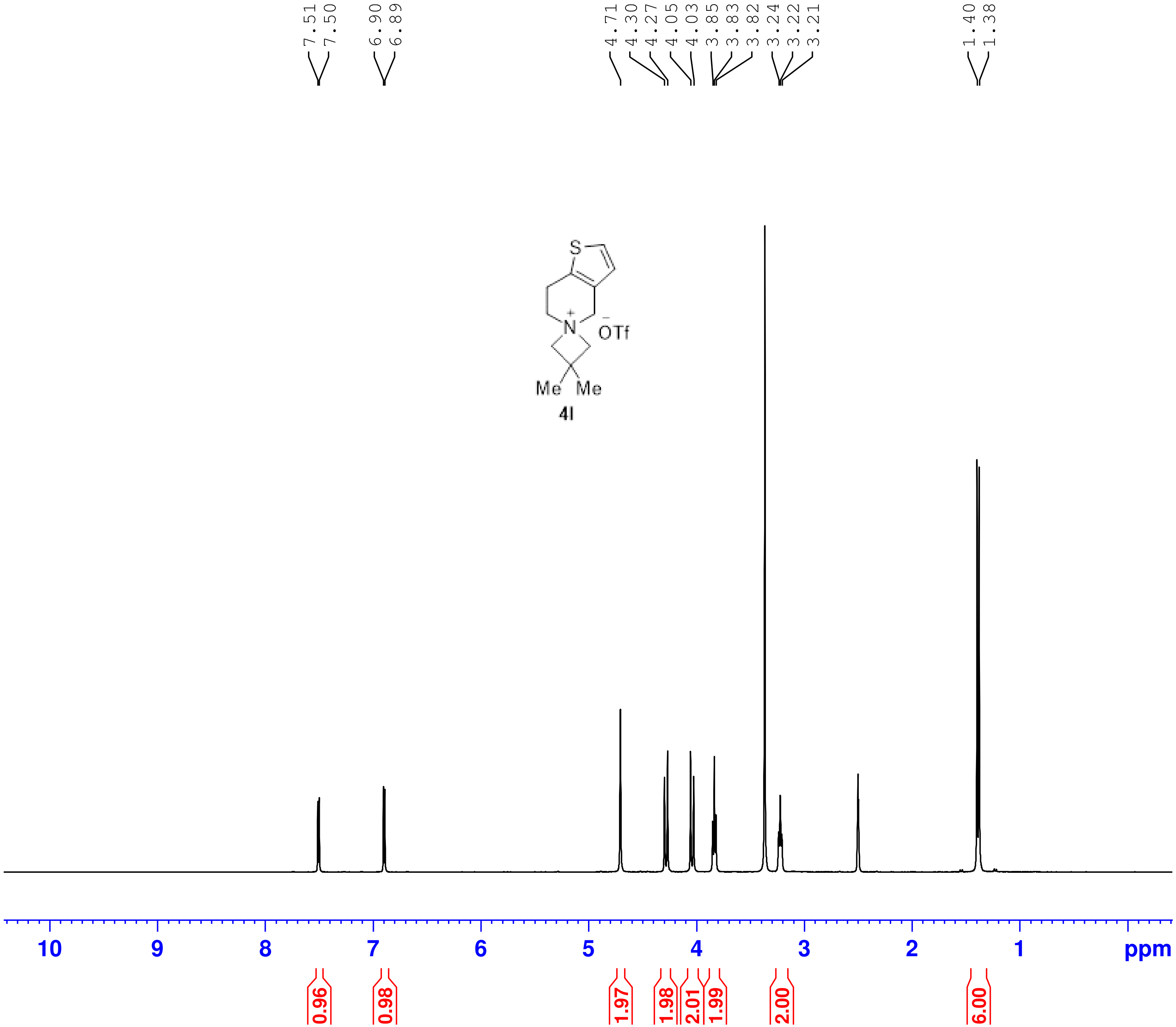


```
Current Data Parameters
NAME              zmh-syh-2
EXPNO                     1
PROCNO                    1

F2 - Acquisition Parameters
Date_              20230215
Time                  13.20
INSTRUM               spect
PROBHD    5 mm DUL 13C-1
PULPROG                zg30
TD                    65536
SOLVENT                DMSO
NS                        9
DS                        2
SWH                8223.685 Hz
FIDRES             0.125483 Hz
AQ                3.9845889 sec
RG                      256
DW                   60.800 usec
DE                     6.00 usec
TE                    293.0 K
D1               1.00000000 sec
TD0                       1

======== CHANNEL f1 ========
NUC1                     1H
P1                    15.80 usec
PL1                   -1.00 dB
PL1W            12.17476940 W
SFO1            400.1324710 MHz

F2 - Processing parameters
SI                    32768
SF              400.1300033 MHz
WDW                      EM
SSB                       0
LB                     0.30 Hz
GB                        0
PC                     1.00
```

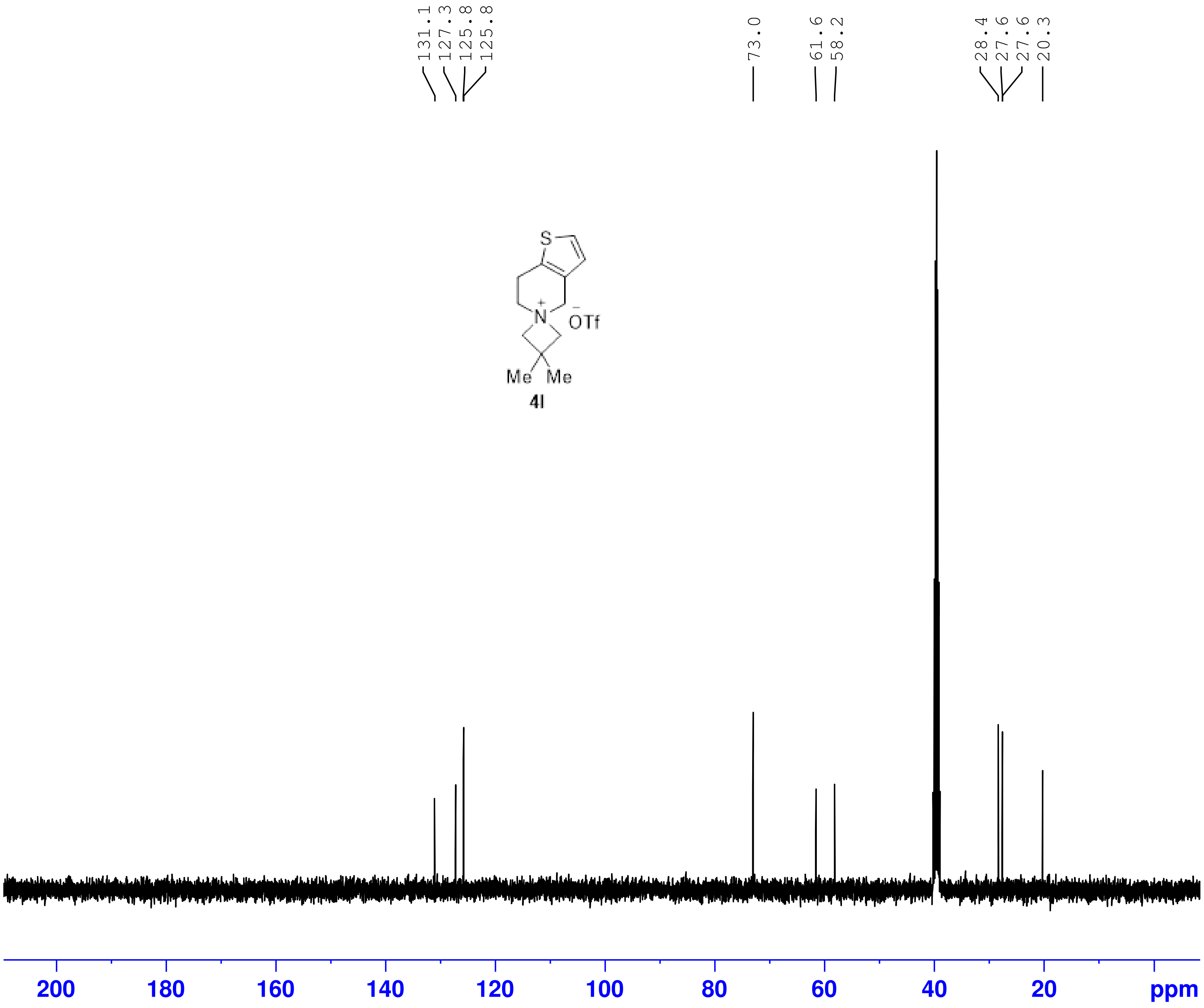


```
Current Data Parameters
NAME            zmh-syh-2
EXPNO                   2
PROCNO                  1

F2 - Acquisition Parameters
Date_            20230215
Time                13.22
INSTRUM             spect
PROBHD   5 mm DUL 13C-1
PULPROG           zgpg30
TD                  65536
SOLVENT              DMSO
NS                    134
DS                      1
SWH             24038.461 Hz
FIDRES           0.366798 Hz
AQ              1.3631488 sec
RG                   2050
DW                 20.800 usec
DE                   6.00 usec
TE                  293.1 K
D1             2.00000000 sec
D11            0.03000000 sec
TD0                     1

======== CHANNEL f1 ========
NUC1                  13C
P1                  40.00 usec
PL1                 -3.00 dB
PL1W          60.64365387 W
SFO1          100.6228298 MHz

======== CHANNEL f2 ========
CPDPRG[2          waltz16
NUC2                   1H
PCPD2               80.00 usec
PL2                 -1.00 dB
PL12                14.39 dB
PL13                18.00 dB
PL2W          12.17476940 W
PL12W          0.35193357 W
PL13W          0.15327126 W
SFO2          400.1316005 MHz

F2 - Processing parameters
SI                  32768
SF            100.6128035 MHz
WDW                    EM
SSB                     0
LB                   1.00 Hz
GB                      0
PC                   1.40
```

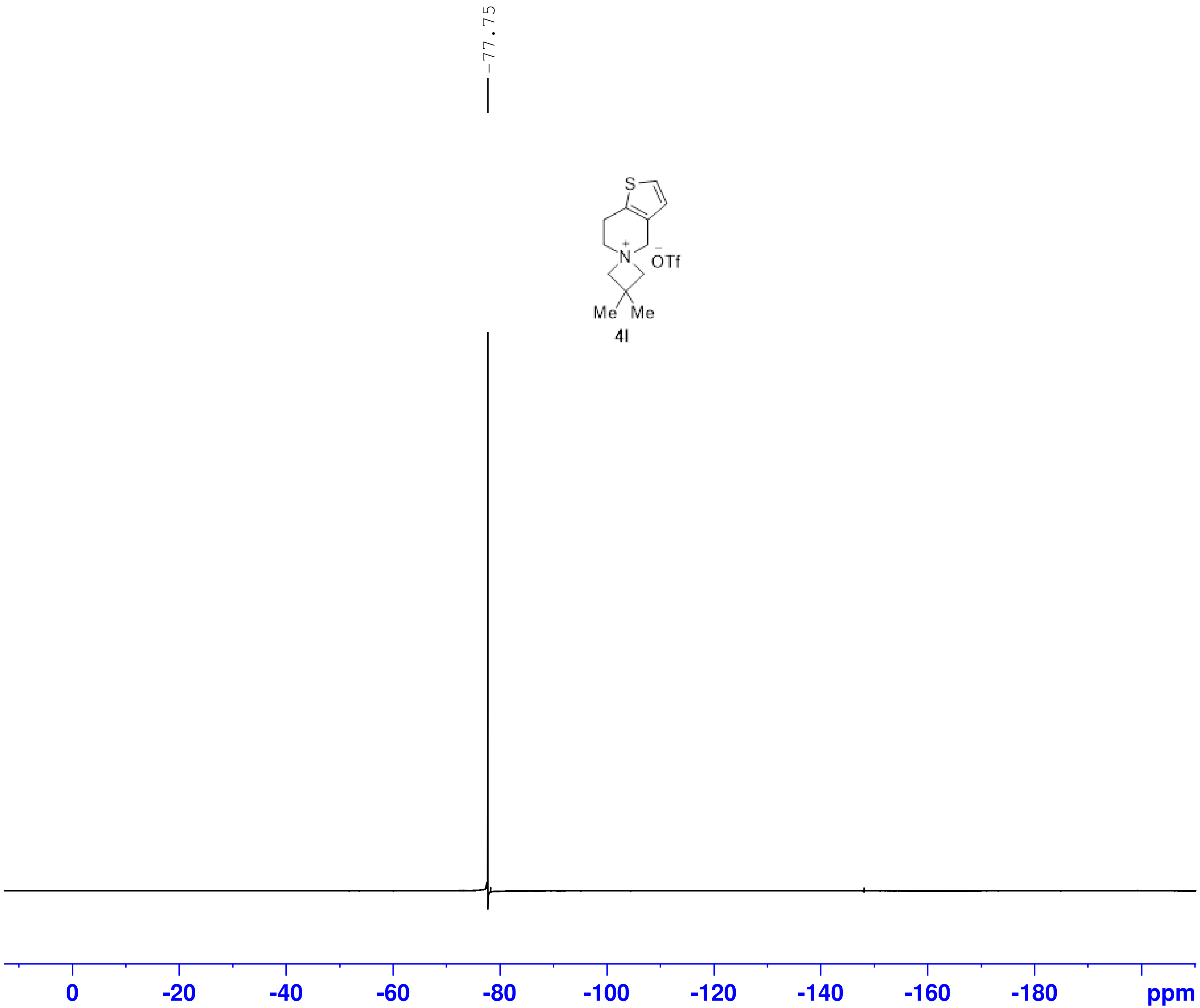

Current Data Parameters
NAME zmh-syh-2
EXPNO 1
PROCNO 1

F2 - Acquisition Parameters
Date_ 20230215
Time 18.32
INSTRUM spect
PROBHD 5 mm PABBO BB/
PULPROG zgpg30
TD 65536
SOLVENT DMSO
NS 16
DS 2
SWH 93750.000 Hz
FIDRES 1.430511 Hz
AQ 0.3495253 sec
RG 196.92
DW 5.333 usec
DE 6.50 usec
TE 294.4 K
D1 2.00000000 sec
D11 0.03000000 sec
TD0 1

======== CHANNEL f1 ========
SFO1 376.4607162 MHz
NUC1 19F
P1 14.70 usec
PLW1 15.99600029 W

======== CHANNEL f2 ========
SFO2 400.1316005 MHz
NUC2 1H
CPDPRG[2 waltz16
PCPD2 90.00 usec
PLW2 11.99499989 W
PLW12 0.34213999 W
PLW13 0.27713001 W

F2 - Processing parameters
SI 32768
SF 376.4983660 MHz
WDW EM
SSB 0
LB 1.00 Hz
GB 0
PC 1.40

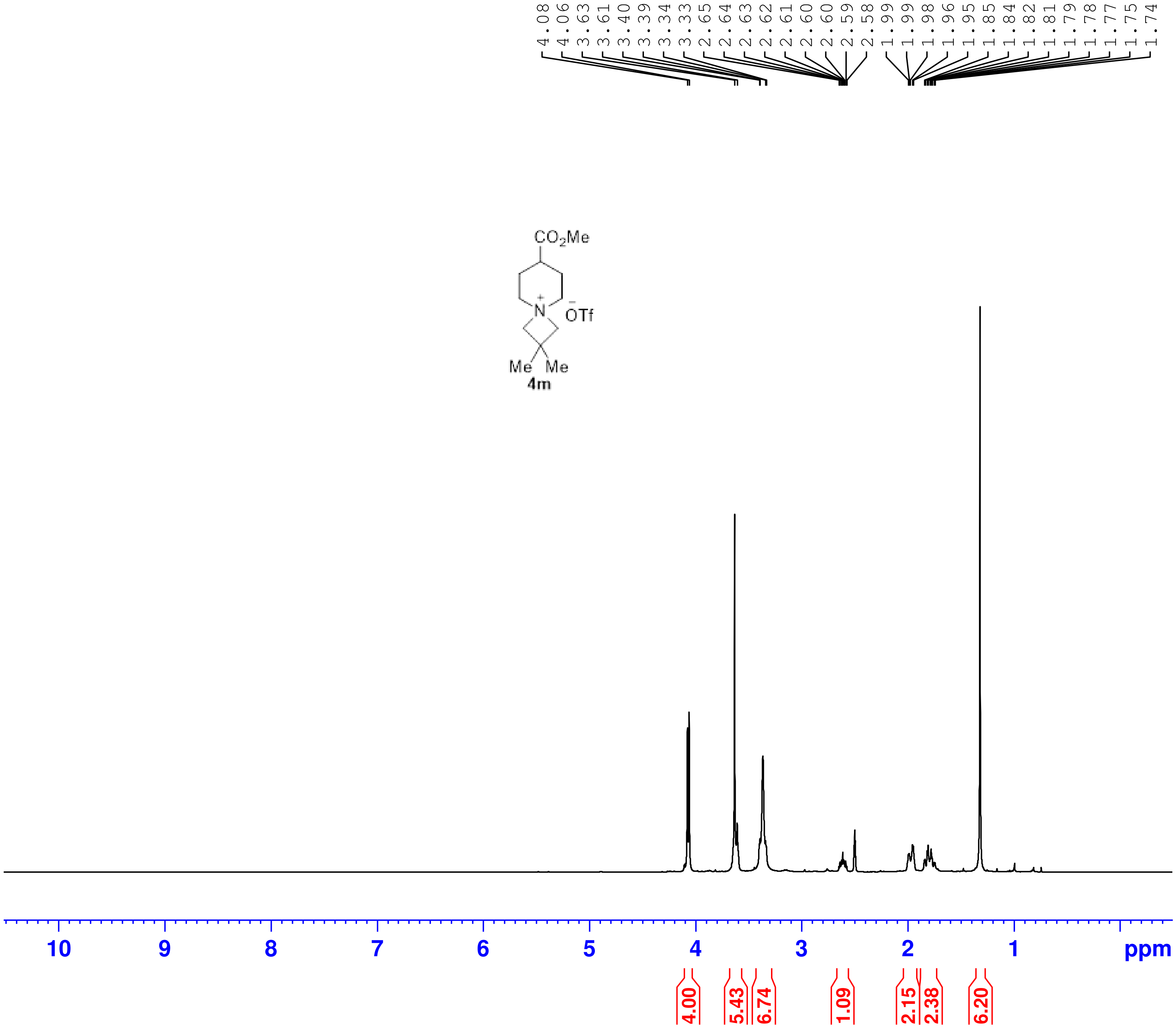

Current Data Parameters

NAME	zmh-syh-15
EXPNO	1
PROCNO	1

F2 - Acquisition Parameters

Date_	20230827
Time	20.28 h
INSTRUM	AvanceNeo 400MHz
PROBHD	Z163739_0629 (
PULPROG	zg30
TD	65536
SOLVENT	DMSO
NS	8
DS	2
SWH	8196.722 Hz
FIDRES	0.250144 Hz
AQ	3.9976959 sec
RG	101
DW	61.000 usec
DE	13.89 usec
TE	297.1 K
D1	1.00000000 sec
TD0	1
SFO1	400.1824711 MHz
NUC1	1H
P0	2.67 usec
P1	8.00 usec
PLW1	21.26700020 W

F2 - Processing parameters

SI	65536
SF	400.1800026 MHz
WDW	EM
SSB	0
LB	0.30 Hz
GB	0
PC	1.00

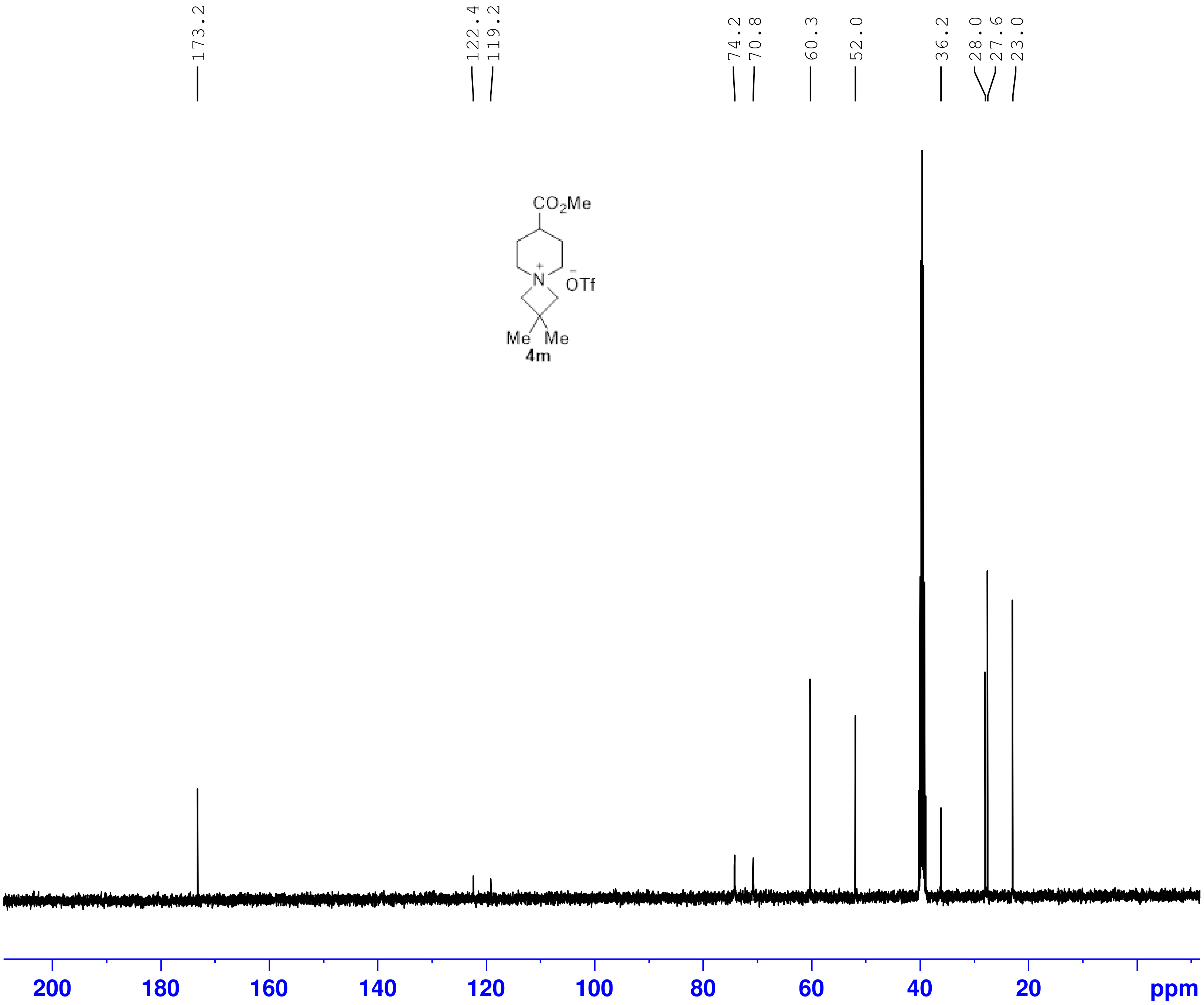

Current Data Parameters
NAME zmh-syh-15
EXPNO 2
PROCNO 1

F2 - Acquisition Parameters
Date_ 20230827
Time 20.36 h
INSTRUM AvanceNeo 400MHz
PROBHD Z163739_0629 (
PULPROG zgpg30
TD 65536
SOLVENT DMSO
NS 100
DS 4
SWH 23809.523 Hz
FIDRES 0.726609 Hz
AQ 1.3762560 sec
RG 10
DW 21.000 usec
DE 6.50 usec
TE 297.4 K
D1 2.00000000 sec
D11 0.03000000 sec
TD0 1
SFO1 100.6354036 MHz
NUC1 13C
P0 2.67 usec
P1 8.00 usec
PLW1 85.25399780 W
SFO2 400.1816007 MHz
NUC2 1H
CPDPRG[2 waltz65
PCPD2 90.00 usec
PLW2 21.26700020 W
PLW12 0.16802999 W
PLW13 0.08452000 W

F2 - Processing parameters
SI 32768
SF 100.6253764 MHz
WDW EM
SSB 0
LB 1.00 Hz
GB 0
PC 1.40

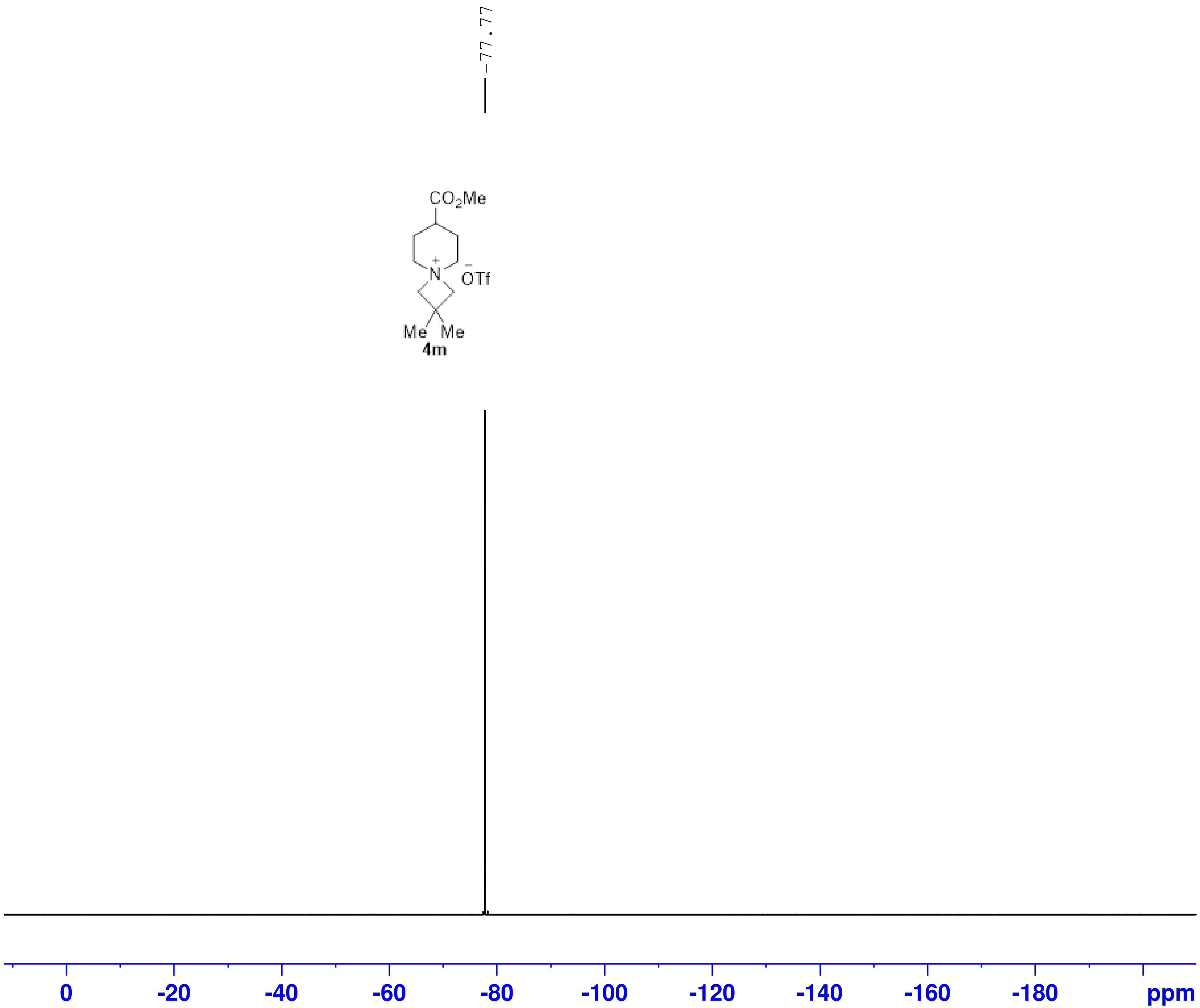

Current Data Parameters
NAME zmh-syh-15
EXPNO 3
PROCNO 1

F2 - Acquisition Parameters
Date_ 20230827
Time 20.38 h
INSTRUM AvanceNeo 400MHz
PROBHD Z163739_0629 (
PULPROG zgig
TD 131072
SOLVENT DMSO
NS 8
DS 4
SWH 90909.094 Hz
FIDRES 1.387163 Hz
AQ 0.7208960 sec
RG 101
DW 5.500 usec
DE 6.50 usec
TE 297.4 K
D1 1.00000000 sec
D11 0.03000000 sec
TD0 1
SFO1 376.5077587 MHz
NUC1 19F
P1 12.00 usec
PLW1 33.72800064 W
SFO2 400.1816007 MHz
NUC2 1H
CPDPRG[2 waltz16
PCPD2 90.00 usec
PLW2 21.26700020 W
PLW12 0.16802999 W

F2 - Processing parameters
SI 65536
SF 376.5454132 MHz
WDW EM
SSB 0
LB 0.30 Hz
GB 0
PC 1.00

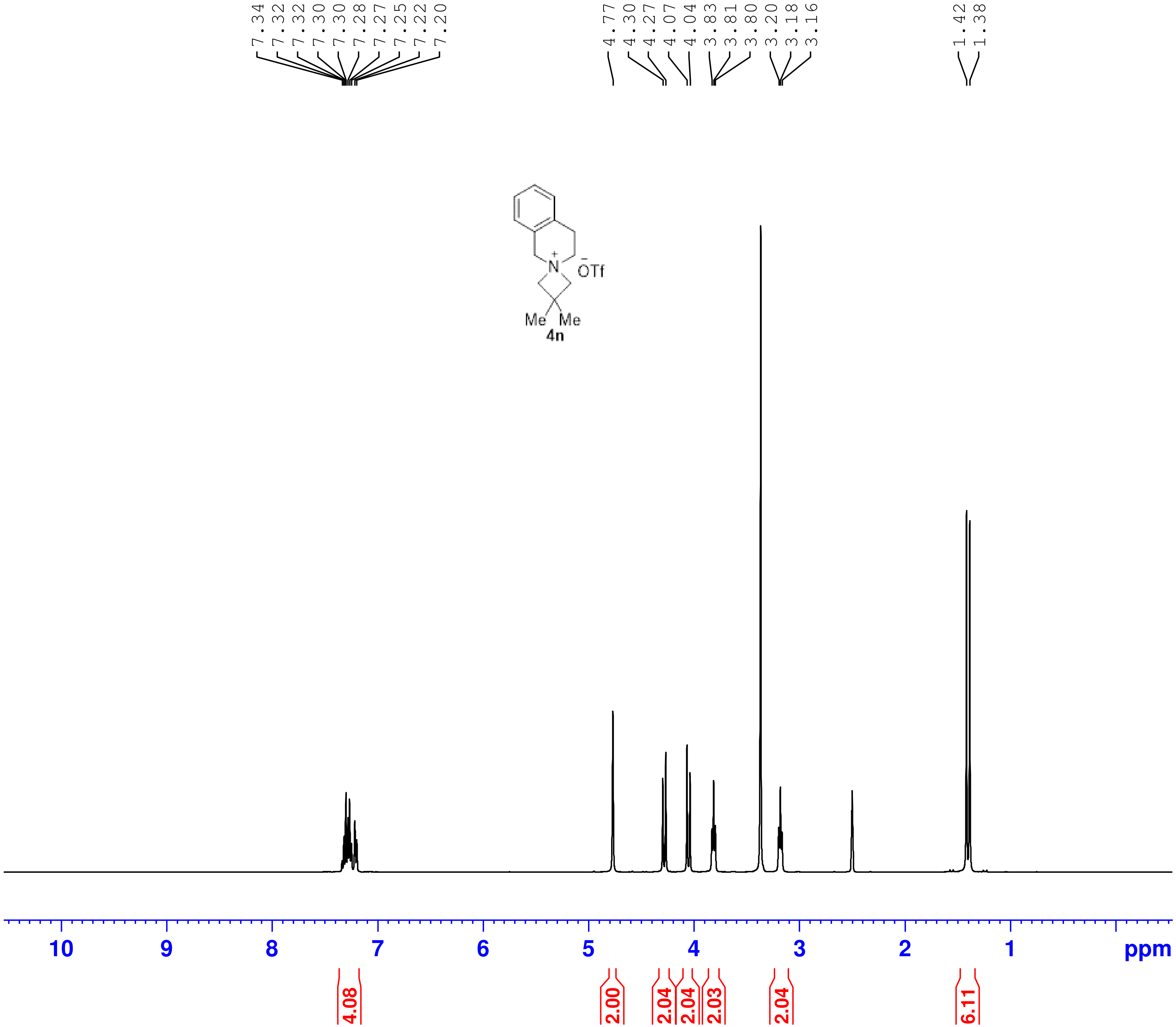

Current Data Parameters
NAME zmh-syh-13
EXPNO 10
PROCNO 1

F2 - Acquisition Parameters
Date_ 20230827
Time 20.03 h
INSTRUM AvanceNeo 400MHz
PROBHD Z163739_0629 (
PULPROG zg30
TD 65536
SOLVENT DMSO
NS 8
DS 2
SWH 8196.722 Hz
FIDRES 0.250144 Hz
AQ 3.9976959 sec
RG 101
DW 61.000 usec
DE 13.89 usec
TE 297.1 K
D1 1.00000000 sec
TD0 1
SFO1 400.1824711 MHz
NUC1 1H
P0 2.67 usec
P1 8.00 usec
PLW1 21.26700020 W

F2 - Processing parameters
SI 65536
SF 400.1800027 MHz
WDW EM
SSB 0
LB 0.30 Hz
GB 0
PC 1.00

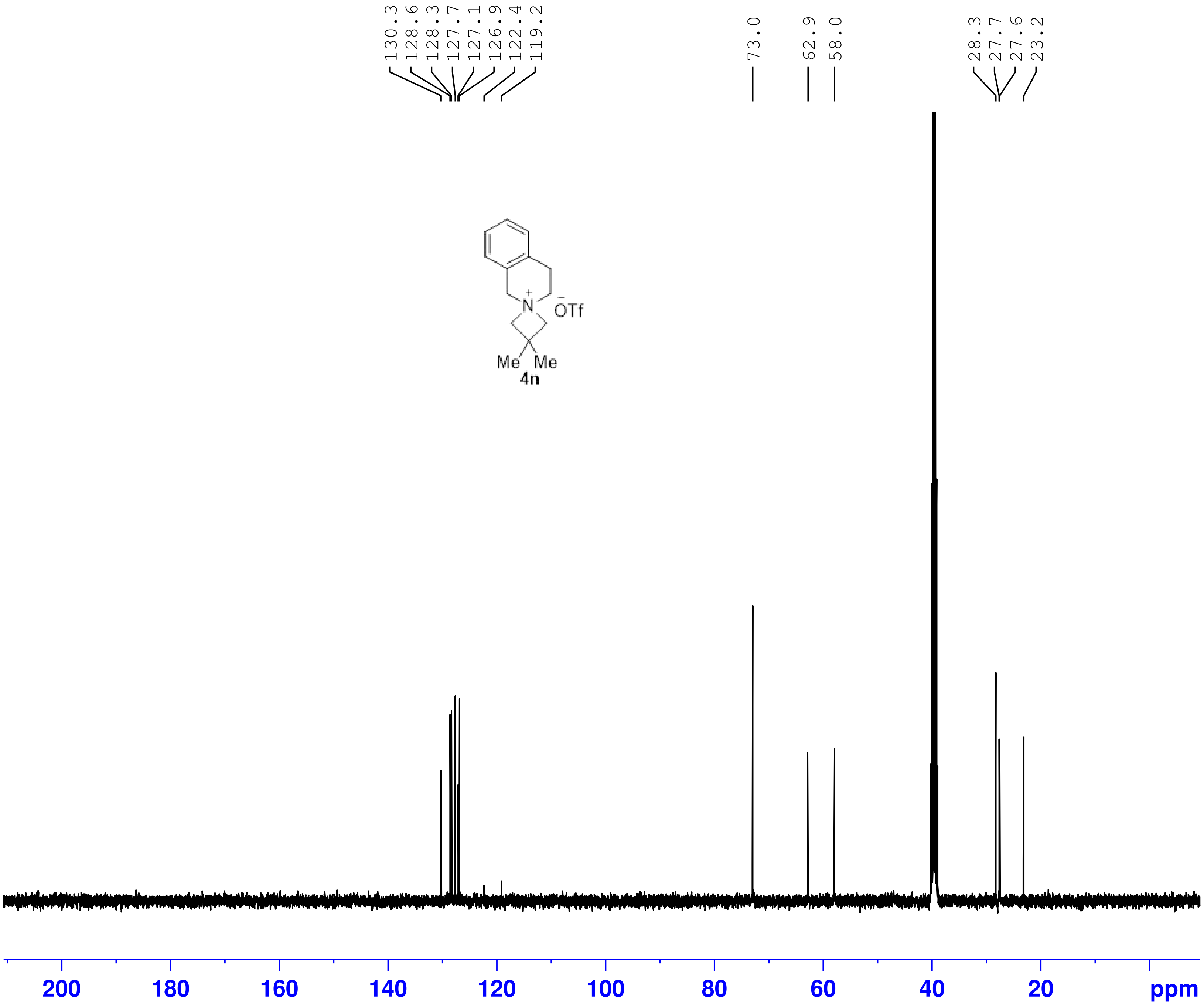

Current Data Parameters
NAME zmh-syh-13
EXPNO 11
PROCNO 1

F2 - Acquisition Parameters
Date_ 20230827
Time 20.11 h
INSTRUM AvanceNeo 400MHz
PROBHD Z163739_0629 (
PULPROG zgpg30
TD 65536
SOLVENT DMSO
NS 100
DS 4
SWH 23809.523 Hz
FIDRES 0.726609 Hz
AQ 1.3762560 sec
RG 10
DW 21.000 usec
DE 6.50 usec
TE 297.8 K
D1 2.00000000 sec
D11 0.03000000 sec
TD0 1
SFO1 100.6354036 MHz
NUC1 13C
P0 2.67 usec
P1 8.00 usec
PLW1 85.25399780 W
SFO2 400.1816007 MHz
NUC2 1H
CPDPRG[2 waltz65
PCPD2 90.00 usec
PLW2 21.26700020 W
PLW12 0.16802999 W
PLW13 0.08452000 W

F2 - Processing parameters
SI 32768
SF 100.6253777 MHz
WDW EM
SSB 0
LB 1.00 Hz
GB 0
PC 1.40

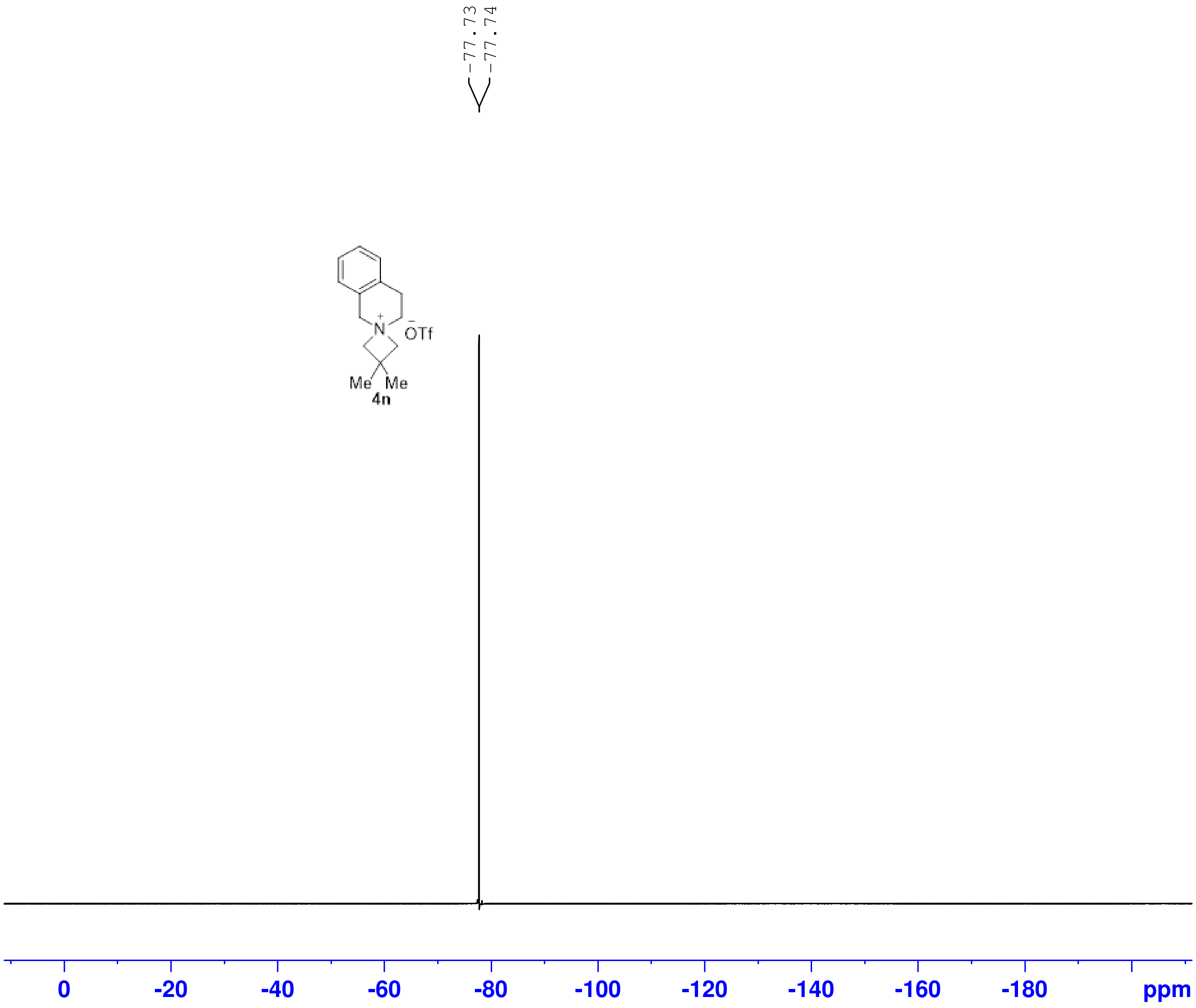

Current Data Parameters
NAME zmh-syh-13
EXPNO 12
PROCNO 1

F2 - Acquisition Parameters
Date_ 20230827
Time 20.13 h
INSTRUM AvanceNeo 400MHz
PROBHD Z163739_0629 (
PULPROG zgig
TD 131072
SOLVENT DMSO
NS 8
DS 4
SWH 90909.094 Hz
FIDRES 1.387163 Hz
AQ 0.7208960 sec
RG 101
DW 5.500 usec
DE 6.50 usec
TE 297.3 K
D1 1.00000000 sec
D11 0.03000000 sec
TD0 1
SFO1 376.5077587 MHz
NUC1 19F
P1 12.00 usec
PLW1 33.72800064 W
SFO2 400.1816007 MHz
NUC2 1H
CPDPRG[2 waltz16
PCPD2 90.00 usec
PLW2 21.26700020 W
PLW12 0.16802999 W

F2 - Processing parameters
SI 65536
SF 376.5454132 MHz
WDW EM
SSB 0
LB 0.30 Hz
GB 0
PC 1.00

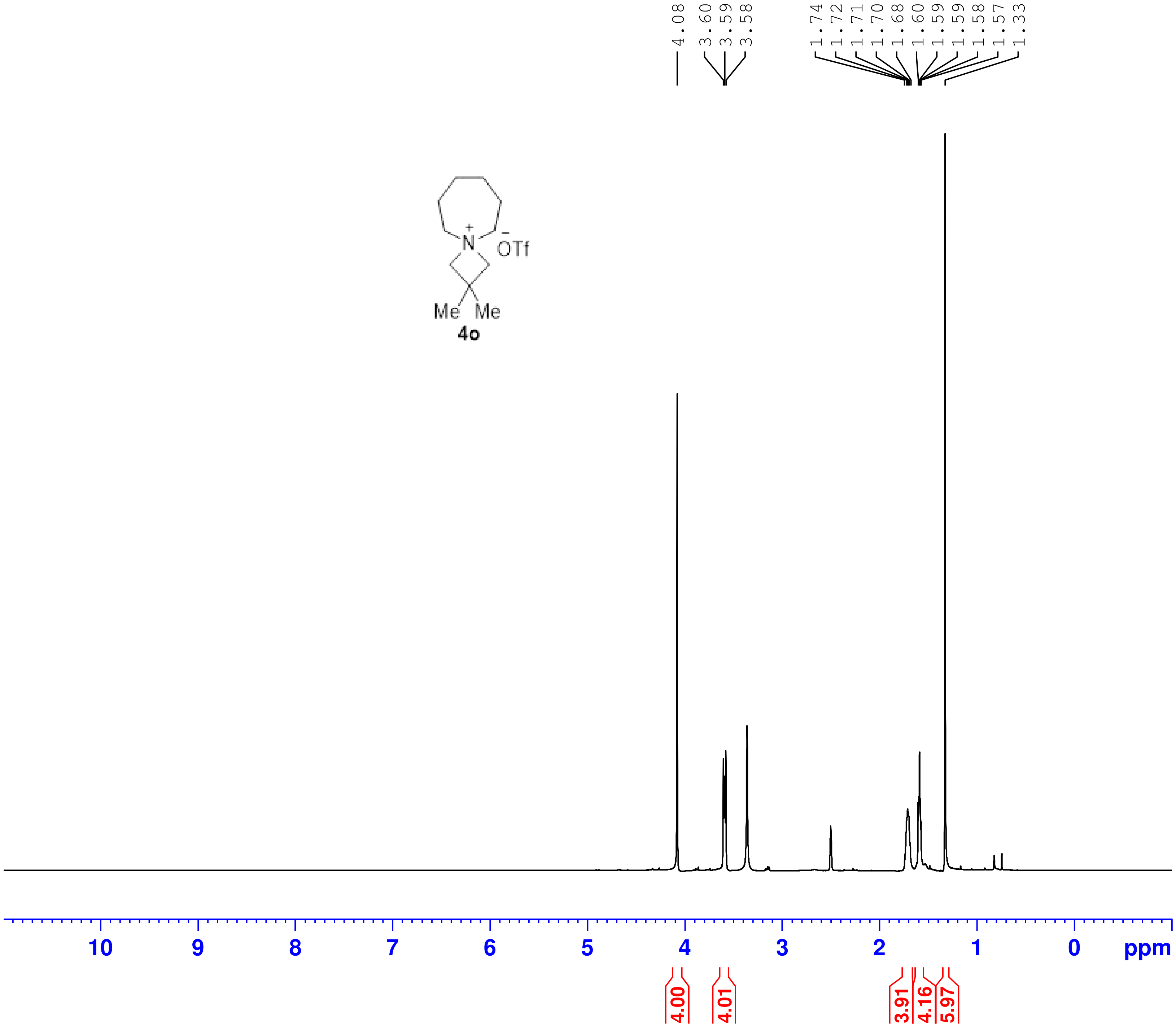

Current Data Parameters
NAME zmh-syh-3
EXPNO 1
PROCNO 1

F2 - Acquisition Parameters
Date_ 20230215
Time 13.08
INSTRUM spect
PROBHD 5 mm DUL 13C-1
PULPROG zg30
TD 65536
SOLVENT DMSO
NS 8
DS 2
SWH 8223.685 Hz
FIDRES 0.125483 Hz
AQ 3.9845889 sec
RG 161
DW 60.800 usec
DE 6.00 usec
TE 293.0 K
D1 1.00000000 sec
TD0 1

======== CHANNEL f1 ========
NUC1 1H
P1 15.80 usec
PL1 -1.00 dB
PL1W 12.17476940 W
SFO1 400.1324710 MHz

F2 - Processing parameters
SI 32768
SF 400.1300035 MHz
WDW EM
SSB 0
LB 0.30 Hz
GB 0
PC 1.00

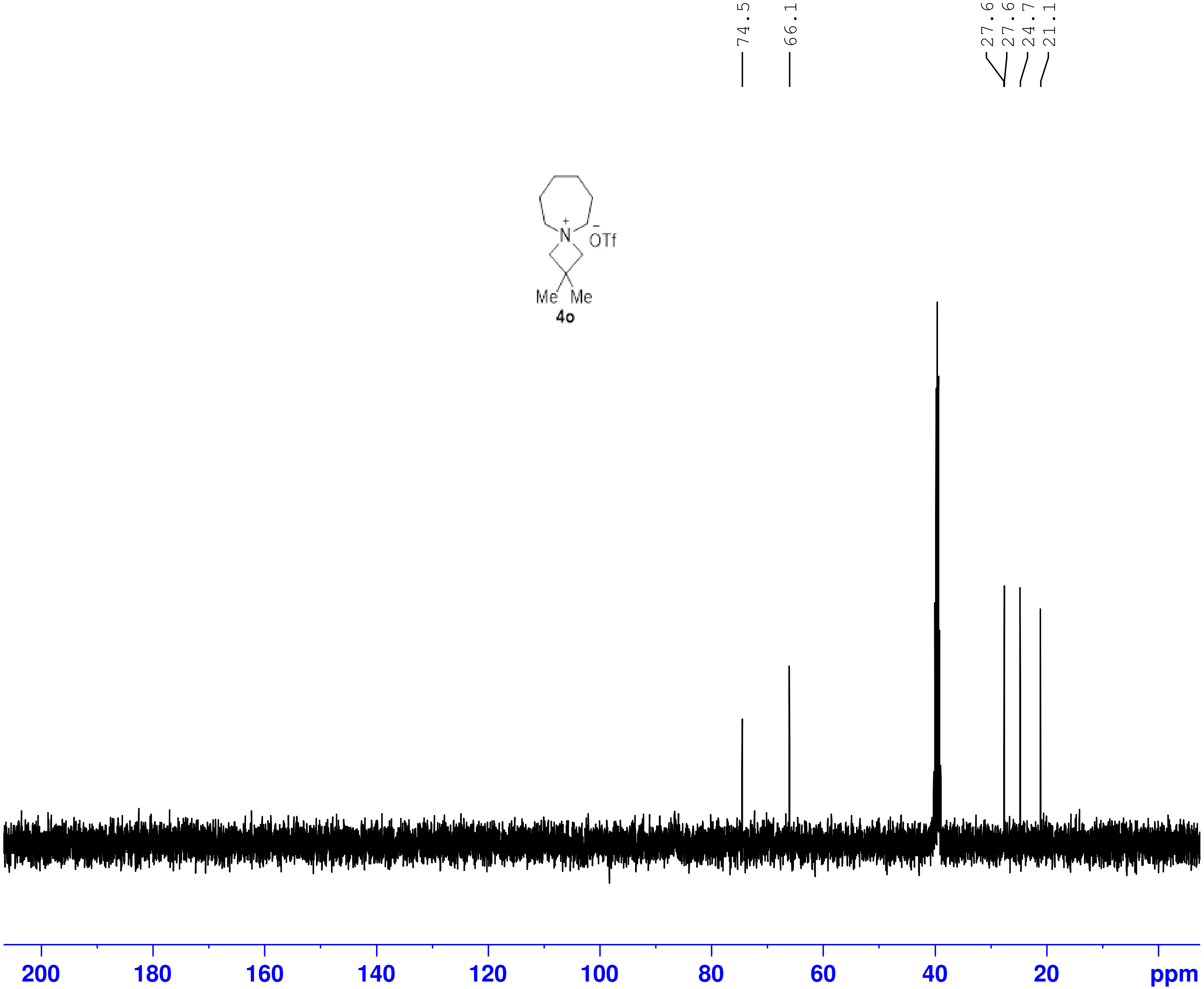

Current Data Parameters
NAME zmh-syh-3
EXPNO 2
PROCNO 1

F2 - Acquisition Parameters
Date_ 20230215
Time 13.10
INSTRUM spect
PROBHD 5 mm DUL 13C-1
PULPROG zgpg30
TD 65536
SOLVENT DMSO
NS 26
DS 1
SWH 24038.461 Hz
FIDRES 0.366798 Hz
AQ 1.3631488 sec
RG 2050
DW 20.800 usec
DE 6.00 usec
TE 293.1 K
D1 2.00000000 sec
D11 0.03000000 sec
TD0 1

======== CHANNEL f1 ========
NUC1 13C
P1 40.00 usec
PL1 -3.00 dB
PL1W 60.64365387 W
SFO1 100.6228298 MHz

======== CHANNEL f2 ========
CPDPRG[2 waltz16
NUC2 1H
PCPD2 80.00 usec
PL2 -1.00 dB
PL12 14.39 dB
PL13 18.00 dB
PL2W 12.17476940 W
PL12W 0.35193357 W
PL13W 0.15327126 W
SFO2 400.1316005 MHz

F2 - Processing parameters
SI 32768
SF 100.6128037 MHz
WDW EM
SSB 0
LB 1.00 Hz
GB 0
PC 1.40

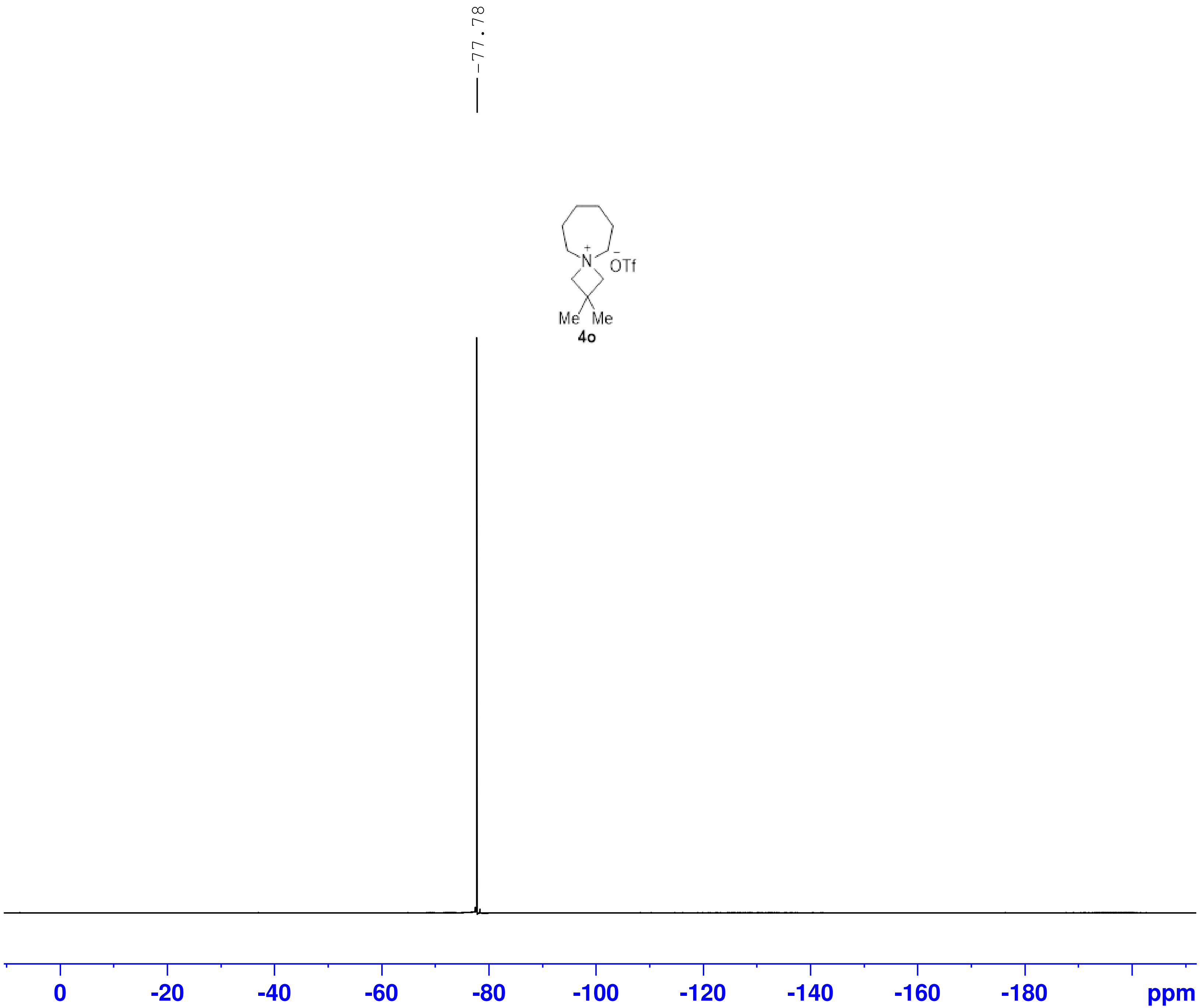

Current Data Parameters

NAME	zmh-syh-3
EXPNO	1
PROCNO	1

F2 - Acquisition Parameters

Date_	20230215
Time	18.35
INSTRUM	spect
PROBHD	5 mm PABBO BB/
PULPROG	zgpg30
TD	65536
SOLVENT	DMSO
NS	16
DS	2
SWH	93750.000 Hz
FIDRES	1.430511 Hz
AQ	0.3495253 sec
RG	196.92
DW	5.333 usec
DE	6.50 usec
TE	294.1 K
D1	2.00000000 sec
D11	0.03000000 sec
TD0	1

======== CHANNEL f1 ========

SFO1	376.4607162 MHz
NUC1	19F
P1	14.70 usec
PLW1	15.99600029 W

======== CHANNEL f2 ========

SFO2	400.1316005 MHz
NUC2	1H
CPDPRG[2	waltz16
PCPD2	90.00 usec
PLW2	11.99499989 W
PLW12	0.34213999 W
PLW13	0.27713001 W

F2 - Processing parameters

SI	32768
SF	376.4983660 MHz
WDW	EM
SSB	0
LB	1.00 Hz
GB	0
PC	1.40

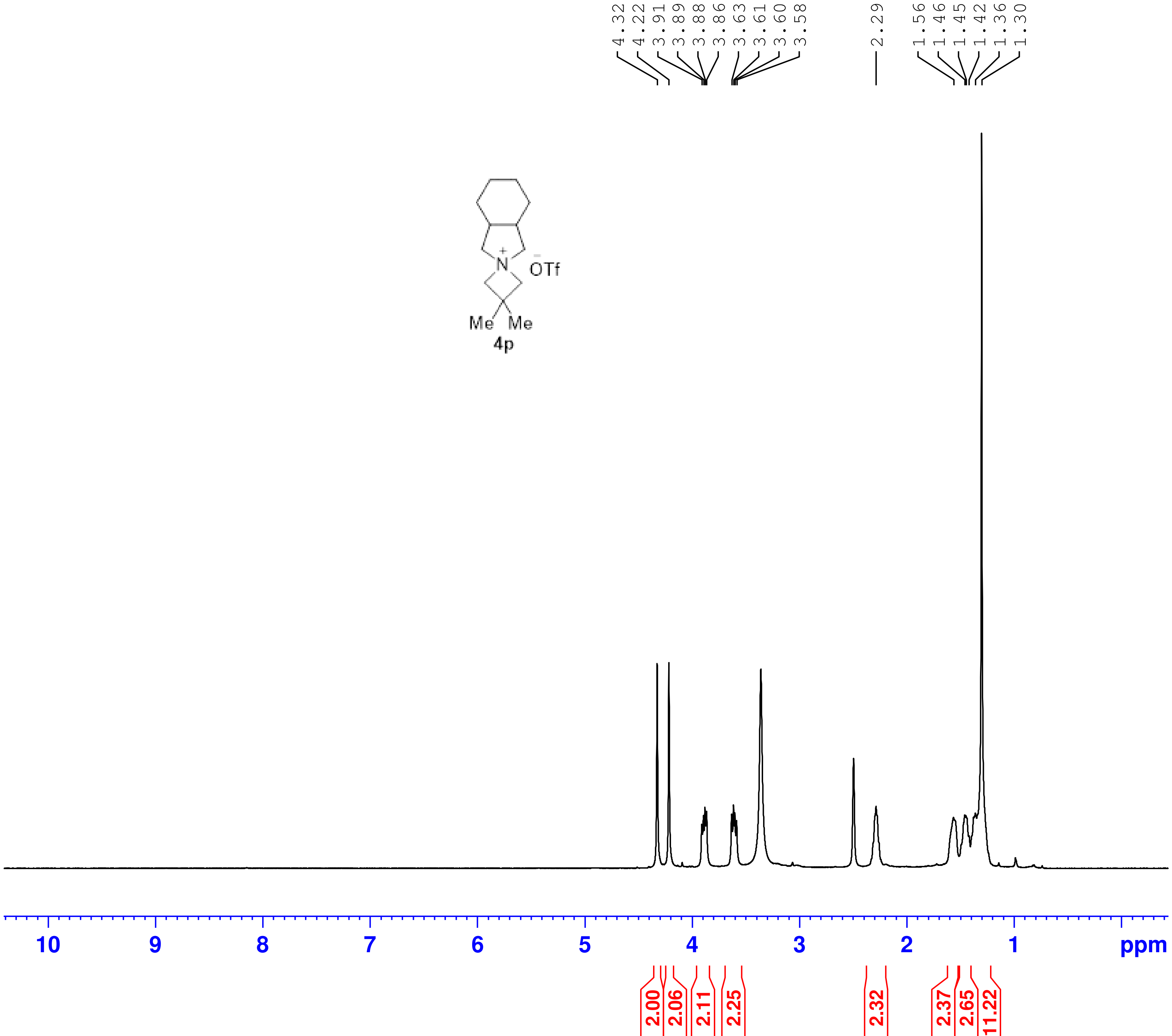

Current Data Parameters
NAME zmh-syh-8
EXPNO 1
PROCNO 1

F2 - Acquisition Parameters
Date_ 20230215
Time 13.37
INSTRUM spect
PROBHD 5 mm DUL 13C-1
PULPROG zg30
TD 65536
SOLVENT DMSO
NS 8
DS 2
SWH 8223.685 Hz
FIDRES 0.125483 Hz
AQ 3.9845889 sec
RG 203
DW 60.800 usec
DE 6.00 usec
TE 292.9 K
D1 1.00000000 sec
TD0 1

======== CHANNEL f1 ========
NUC1 1H
P1 15.80 usec
PL1 -1.00 dB
PL1W 12.17476940 W
SFO1 400.1324710 MHz

F2 - Processing parameters
SI 32768
SF 400.1300054 MHz
WDW EM
SSB 0
LB 0.30 Hz
GB 0
PC 1.00

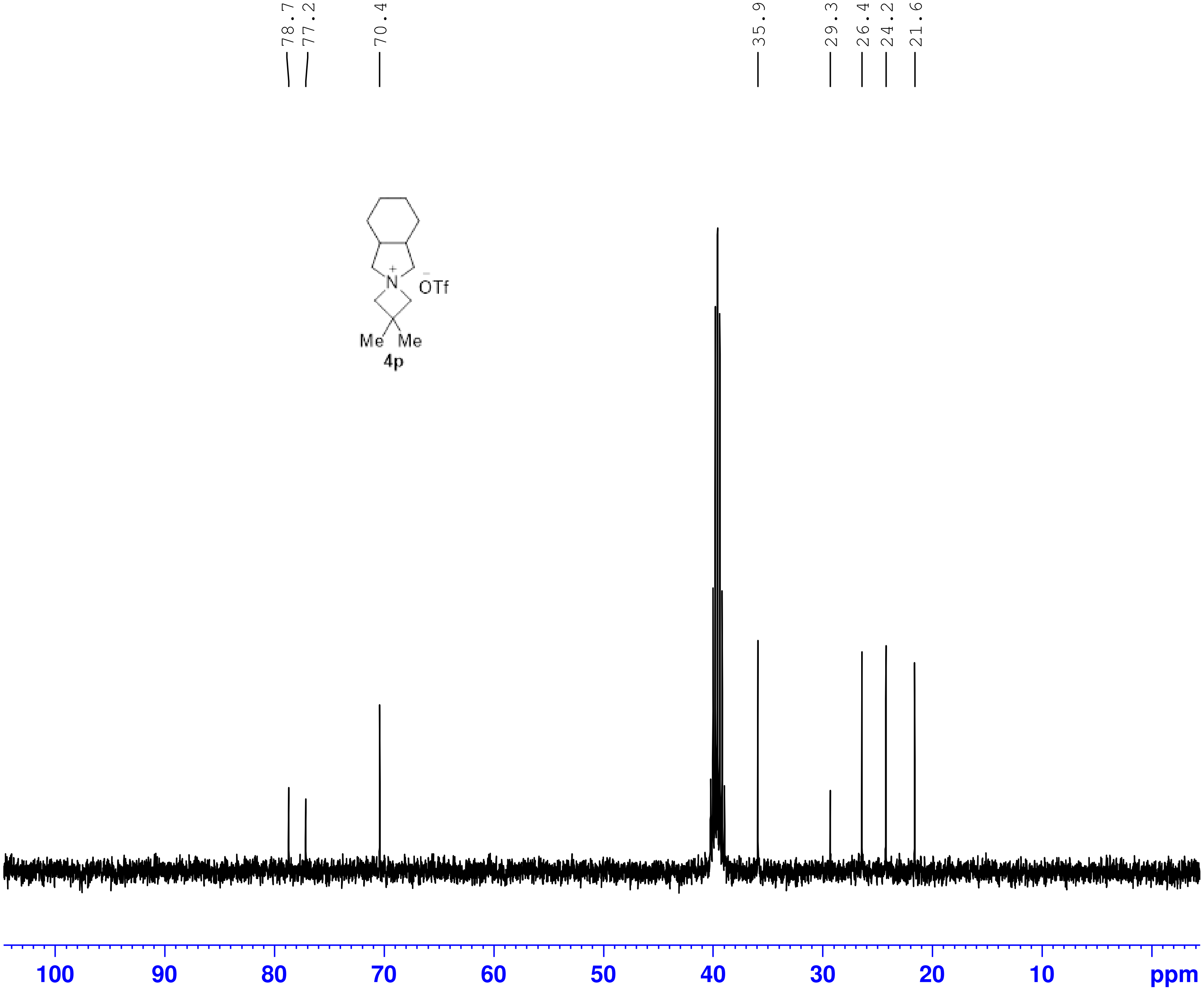

Current Data Parameters	
NAME	zmh-syh-8
EXPNO	2
PROCNO	1
**F2 - Acquisition Parameters**	
Date_	20230215
Time	13.39
INSTRUM	spect
PROBHD	5 mm DUL 13C-1
PULPROG	zgpg30
TD	65536
SOLVENT	DMSO
NS	125
DS	1
SWH	24038.461 Hz
FIDRES	0.366798 Hz
AQ	1.3631488 sec
RG	2050
DW	20.800 usec
DE	6.00 usec
TE	293.0 K
D1	2.00000000 sec
D11	0.03000000 sec
TD0	1
======== CHANNEL f1 ========	
NUC1	13C
P1	40.00 usec
PL1	-3.00 dB
PL1W	60.64365387 W
SFO1	100.6228298 MHz
======== CHANNEL f2 ========	
CPDPRG[2	waltz16
NUC2	1H
PCPD2	80.00 usec
PL2	-1.00 dB
PL12	14.39 dB
PL13	18.00 dB
PL2W	12.17476940 W
PL12W	0.35193357 W
PL13W	0.15327126 W
SFO2	400.1316005 MHz
**F2 - Processing parameters**	
SI	32768
SF	100.6128038 MHz
WDW	EM
SSB	0
LB	1.00 Hz
GB	0
PC	1.40

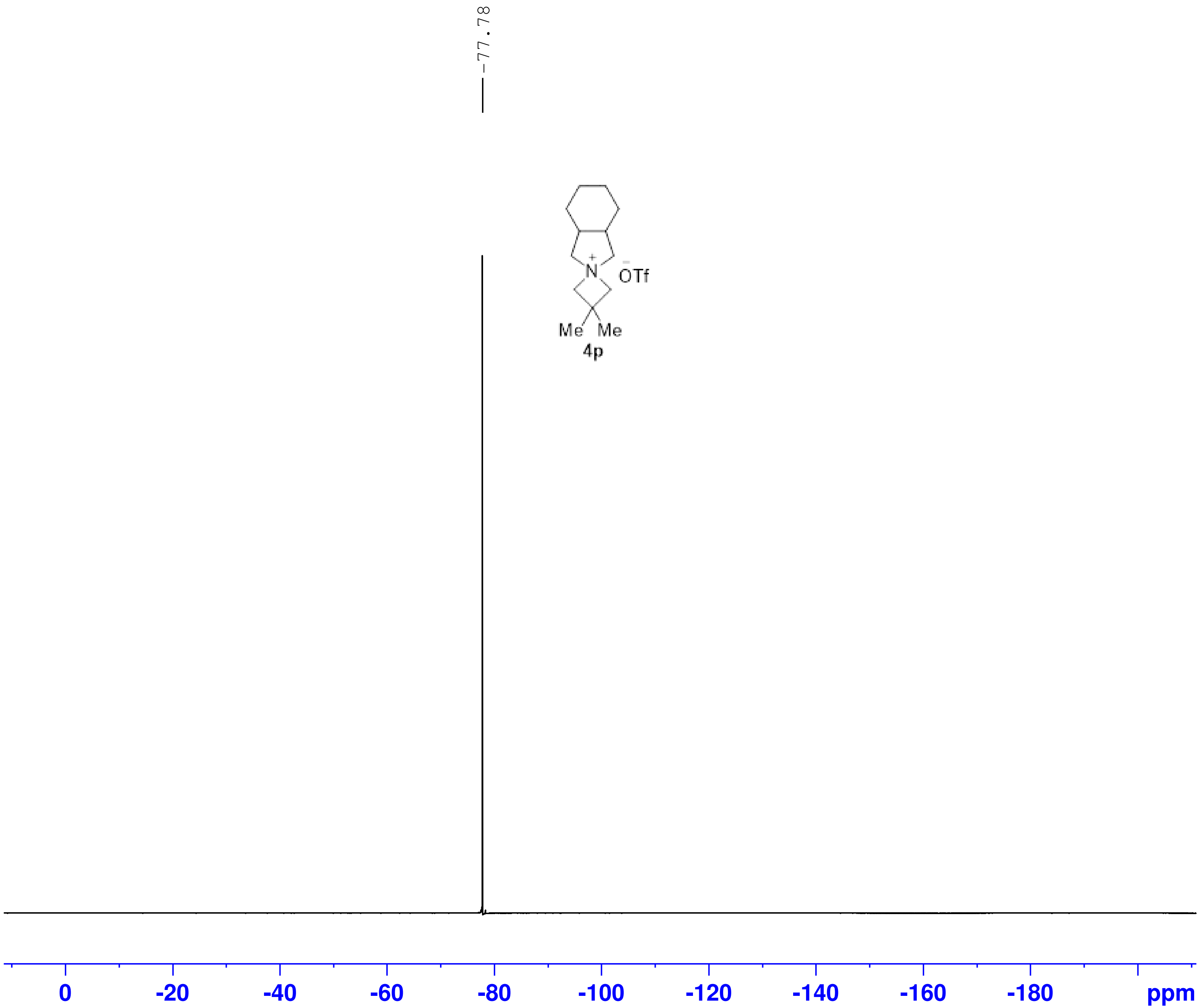

4p

Current Data Parameters
NAME zmh-syh-8
EXPNO 1
PROCNO 1

F2 - Acquisition Parameters
Date_ 20230215
Time 18.50
INSTRUM spect
PROBHD 5 mm PABBO BB/
PULPROG zgpg30
TD 65536
SOLVENT DMSO
NS 16
DS 2
SWH 93750.000 Hz
FIDRES 1.430511 Hz
AQ 0.3495253 sec
RG 196.92
DW 5.333 usec
DE 6.50 usec
TE 294.4 K
D1 2.00000000 sec
D11 0.03000000 sec
TD0 1

======== CHANNEL f1 ========
SFO1 376.4607162 MHz
NUC1 19F
P1 14.70 usec
PLW1 15.99600029 W

======== CHANNEL f2 ========
SFO2 400.1316005 MHz
NUC2 1H
CPDPRG[2 waltz16
PCPD2 90.00 usec
PLW2 11.99499989 W
PLW12 0.34213999 W
PLW13 0.27713001 W

F2 - Processing parameters
SI 32768
SF 376.4983660 MHz
WDW EM
SSB 0
LB 1.00 Hz
GB 0
PC 1.40

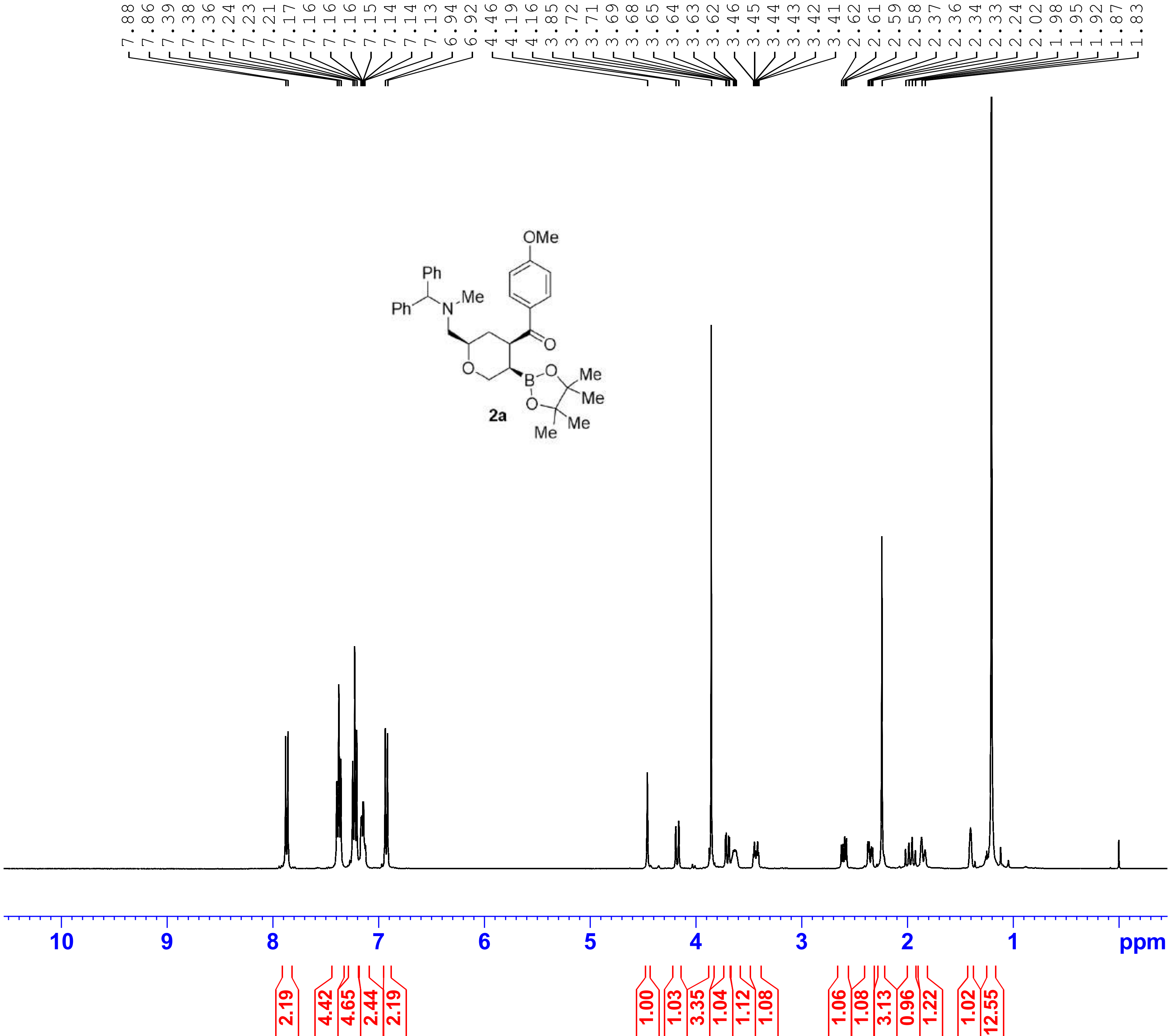

Current Data Parameters
NAME zmh-5-180j-chun
EXPNO 7
PROCNO 1

F2 - Acquisition Parameters
Date_ 20240518
Time 18.39 h
INSTRUM AvanceNeo 400MHz
PROBHD Z163739_0629 (
PULPROG zg30
TD 65536
SOLVENT CDCl3
NS 8
DS 2
SWH 8196.722 Hz
FIDRES 0.250144 Hz
AQ 3.9976959 sec
RG 45.2
DW 61.000 usec
DE 13.89 usec
TE 297.2 K
D1 1.00000000 sec
TD0 1
SFO1 400.1824711 MHz
NUC1 1H
P0 2.67 usec
P1 8.00 usec
PLW1 21.26700020 W

F2 - Processing parameters
SI 65536
SF 400.1800148 MHz
WDW EM
SSB 0
LB 0.30 Hz
GB 0
PC 1.00

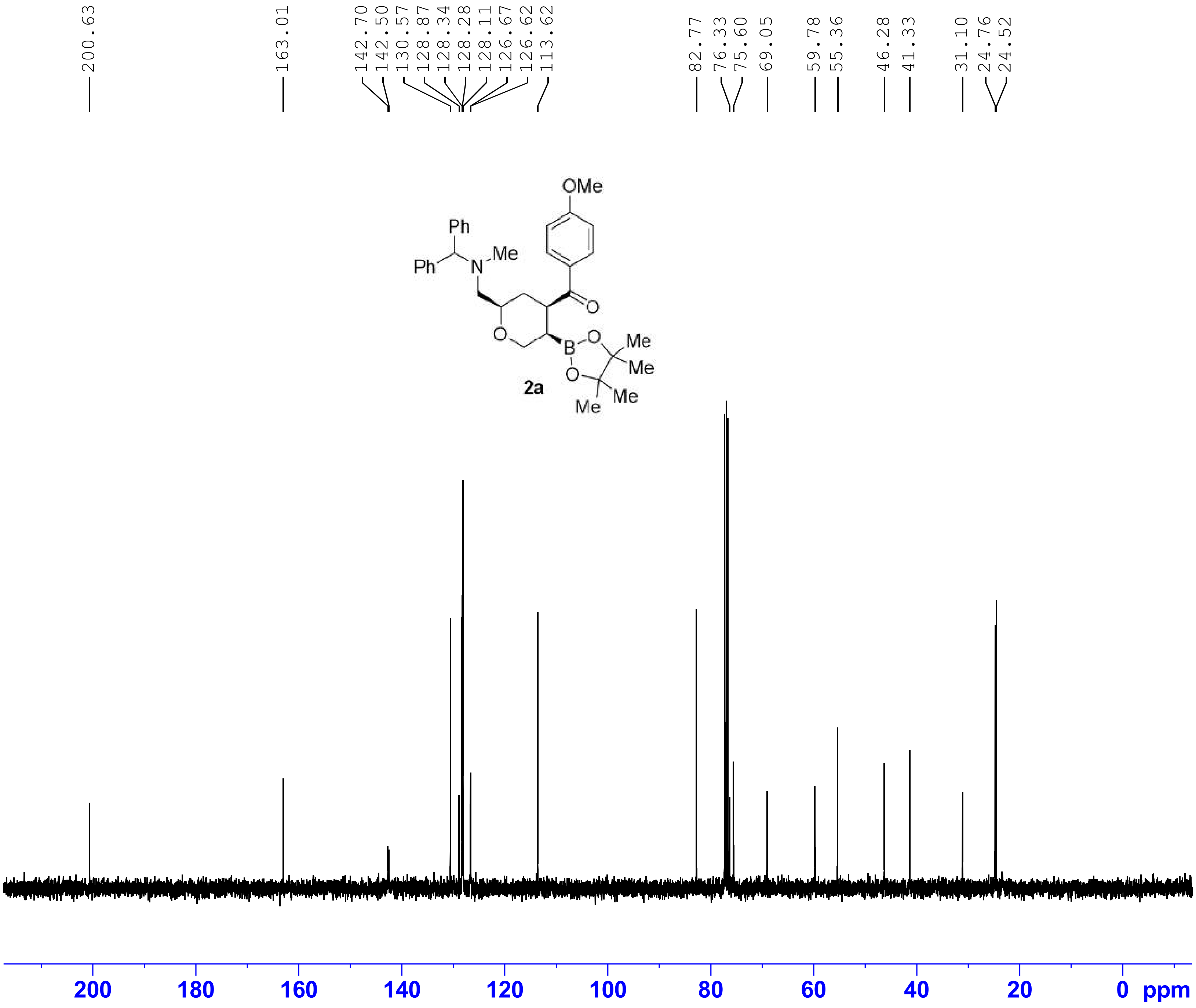

Current Data Parameters
NAME zmh-5-180j-chun
EXPNO 8
PROCNO 1

F2 - Acquisition Parameters
Date_ 20240518
Time 18.43 h
INSTRUM AvanceNeo 400MHz
PROBHD Z163739_0629 (
PULPROG zgpg30
TD 65536
SOLVENT CDCl3
NS 42
DS 4
SWH 23809.523 Hz
FIDRES 0.726609 Hz
AQ 1.3762560 sec
RG 10
DW 21.000 usec
DE 6.50 usec
TE 297.7 K
D1 2.00000000 sec
D11 0.03000000 sec
TD0 1
SFO1 100.6354036 MHz
NUC1 13C
P0 2.67 usec
P1 8.00 usec
PLW1 85.25399780 W
SFO2 400.1816007 MHz
NUC2 1H
CPDPRG[2 waltz65
PCPD2 90.00 usec
PLW2 21.26700020 W
PLW12 0.16802999 W
PLW13 0.08452000 W

F2 - Processing parameters
SI 32768
SF 100.6253506 MHz
WDW EM
SSB 0
LB 1.00 Hz
GB 0
PC 1.40

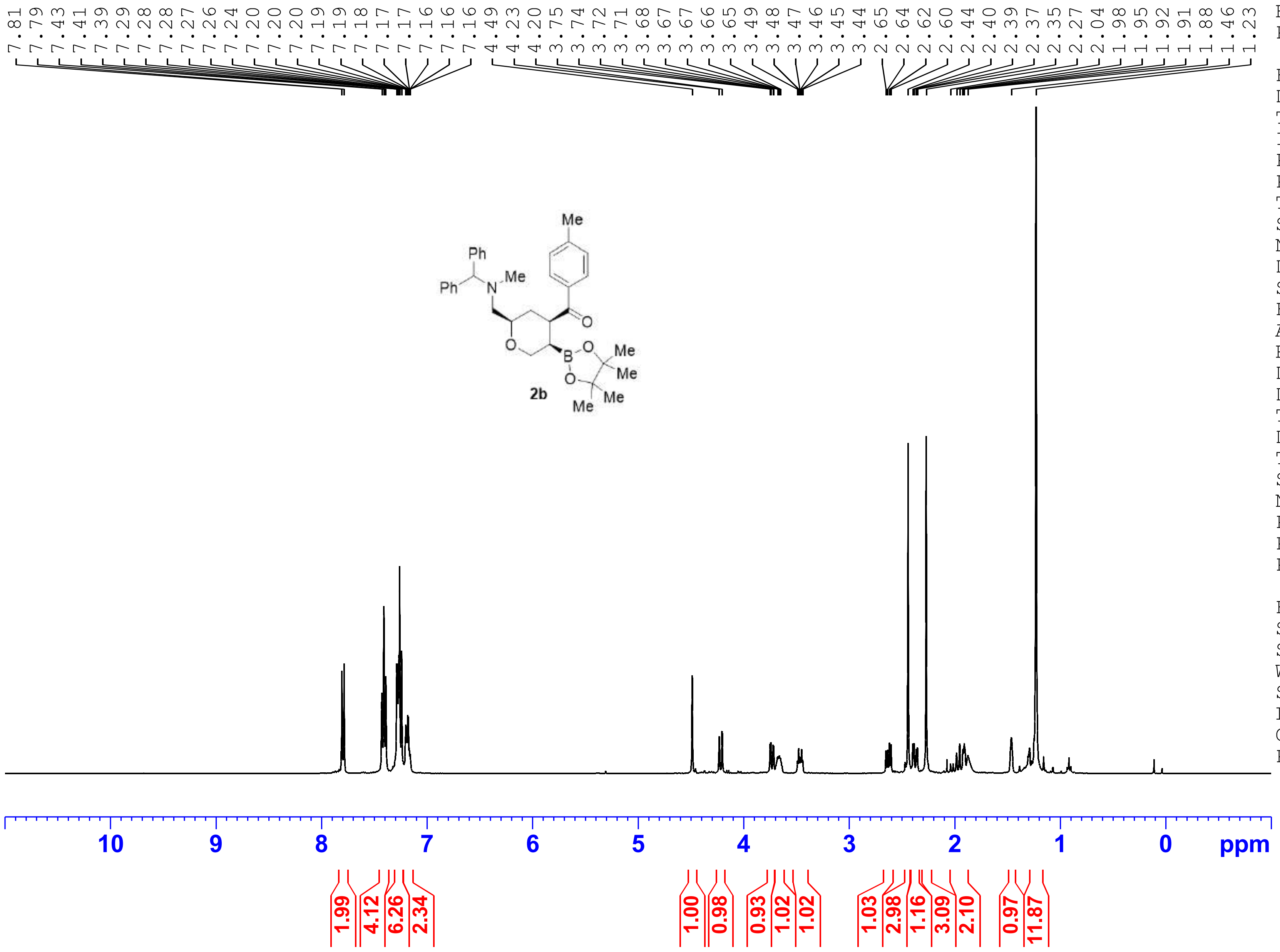

Current Data Parameters
NAME zmh-5-180a-chun
EXPNO 1
PROCNO 1

F2 - Acquisition Parameters
Date_ 20240515
Time 13.17 h
INSTRUM AvanceNeo 400MHz
PROBHD Z163739_0629 (
PULPROG zg30
TD 65536
SOLVENT CDCl3
NS 8
DS 2
SWH 8196.722 Hz
FIDRES 0.250144 Hz
AQ 3.9976959 sec
RG 101
DW 61.000 usec
DE 13.89 usec
TE 296.5 K
D1 1.00000000 sec
TD0 1
SFO1 400.1824711 MHz
NUC1 1H
P0 2.67 usec
P1 8.00 usec
PLW1 21.26700020 W

F2 - Processing parameters
SI 65536
SF 400.1800000 MHz
WDW EM
SSB 0
LB 0.30 Hz
GB 0
PC 1.00

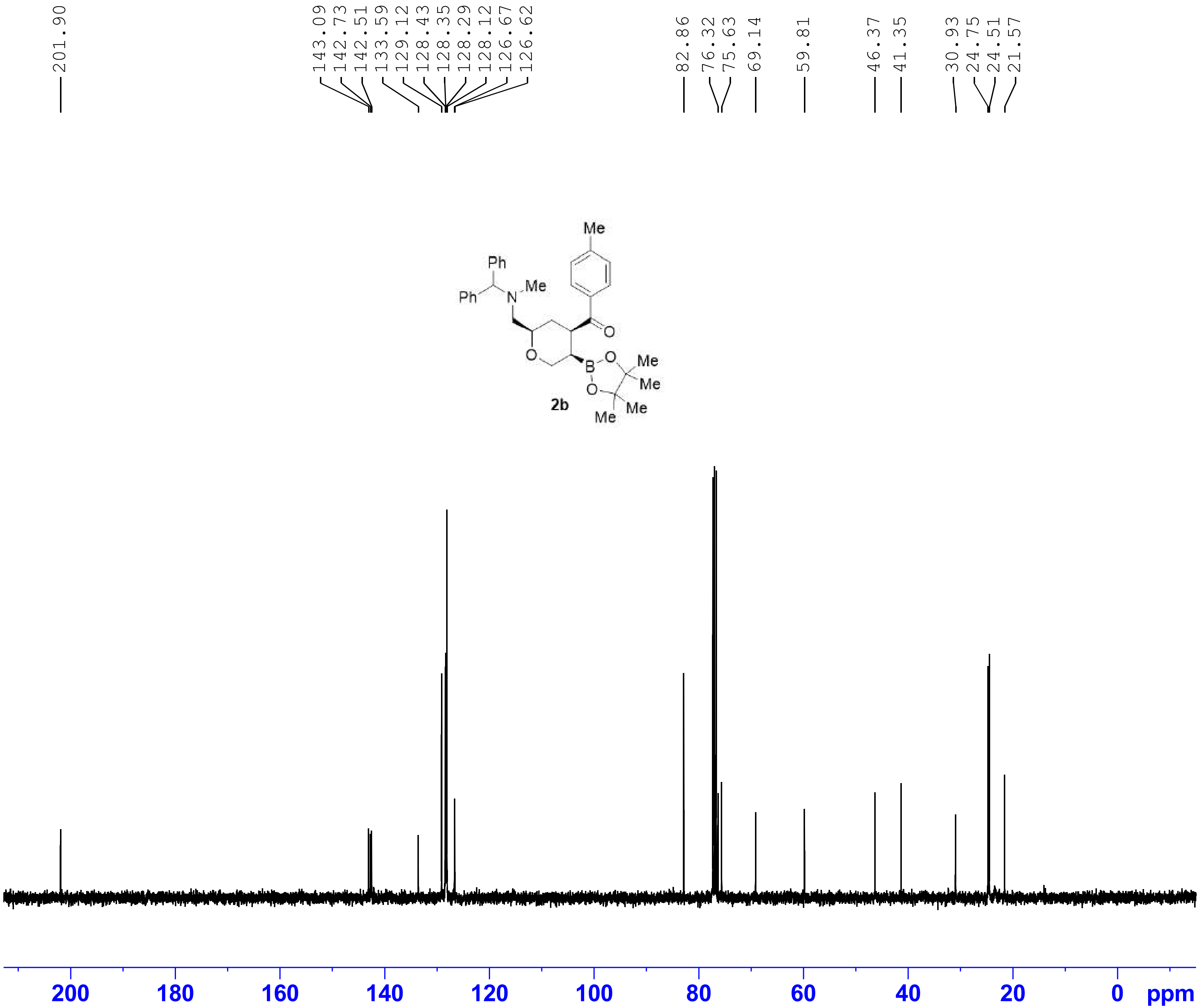

2b

```
Current Data Parameters
NAME          zmh-5-180a-chun
EXPNO                       2
PROCNO                      1

F2 - Acquisition Parameters
Date_                20240515
Time                  13.22 h
INSTRUM      AvanceNeo 400MHz
PROBHD       Z163739_0629 (
PULPROG               zgpg30
TD                      65536
SOLVENT                 CDCl3
NS                         75
DS                          4
SWH               23809.523 Hz
FIDRES             0.726609 Hz
AQ                1.3762560 sec
RG                         10
DW                   21.000 usec
DE                     6.50 usec
TE                    296.8 K
D1               2.00000000 sec
D11              0.03000000 sec
TD0                         1
SFO1            100.6354036 MHz
NUC1                      13C
P0                     2.67 usec
P1                     8.00 usec
PLW1            85.25399780 W
SFO2            400.1816007 MHz
NUC2                       1H
CPDPRG[2              waltz65
PCPD2                 90.00 usec
PLW2            21.26700020 W
PLW12            0.16802999 W
PLW13            0.08452000 W

F2 - Processing parameters
SI                      32768
SF              100.6253493 MHz
WDW                        EM
SSB                         0
LB                       1.00 Hz
GB                          0
PC                       1.40
```

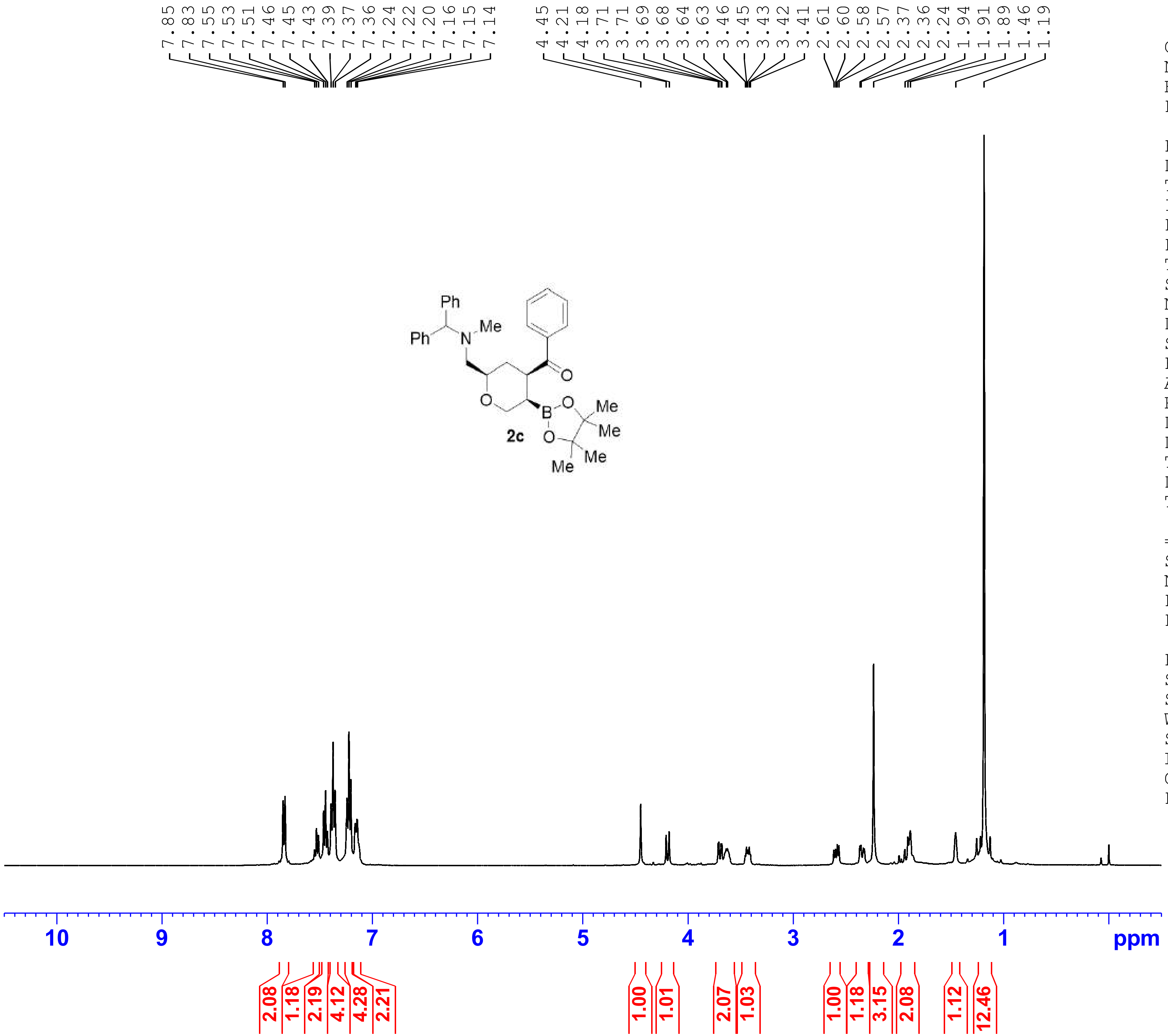

Current Data Parameters
NAME zmh-5-180q
EXPNO 1
PROCNO 1

F2 - Acquisition Parameters
Date_ 20240530
Time 14.04
INSTRUM spect
PROBHD 5 mm PABBO BB/
PULPROG zg30
TD 65536
SOLVENT CDCl3
NS 8
DS 2
SWH 8012.820 Hz
FIDRES 0.122266 Hz
AQ 4.0894465 sec
RG 31.55
DW 62.400 usec
DE 6.50 usec
TE 296.3 K
D1 1.00000000 sec
TD0 1

======== CHANNEL f1 ========
SFO1 400.1324710 MHz
NUC1 1H
P1 14.50 usec
PLW1 11.99499989 W

F2 - Processing parameters
SI 65536
SF 400.1300137 MHz
WDW EM
SSB 0
LB 0.30 Hz
GB 0
PC 1.00

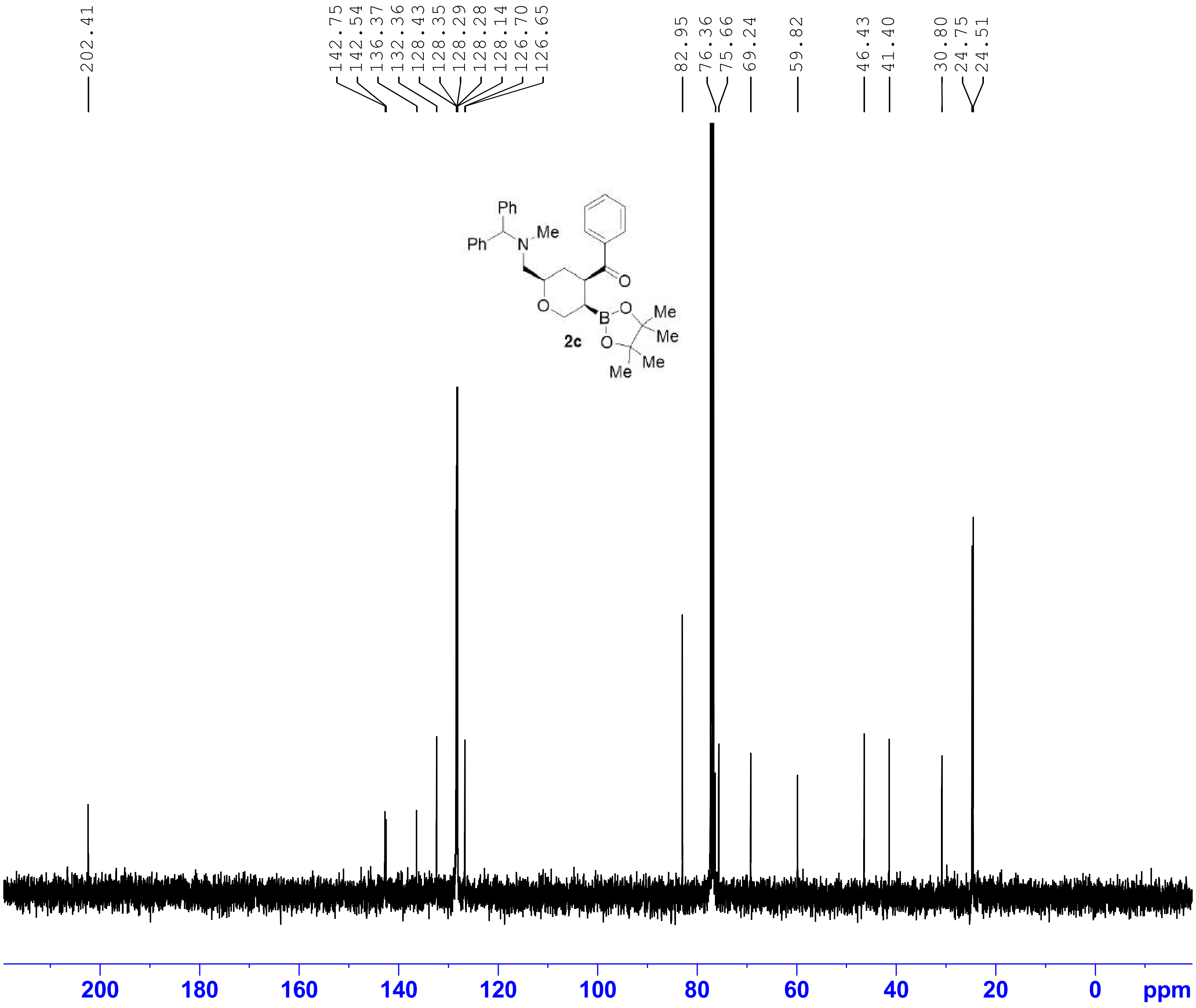

Current Data Parameters
NAME zmh-5-180q
EXPNO 2
PROCNO 1

F2 - Acquisition Parameters
Date_ 20240530
Time 14.05
INSTRUM spect
PROBHD 5 mm PABBO BB/
PULPROG zgpg30
TD 65536
SOLVENT CDCl3
NS 55
DS 2
SWH 24038.461 Hz
FIDRES 0.366798 Hz
AQ 1.3631488 sec
RG 196.92
DW 20.800 usec
DE 6.50 usec
TE 296.4 K
D1 2.00000000 sec
D11 0.03000000 sec
TD0 1

======== CHANNEL f1 ========
SFO1 100.6228298 MHz
NUC1 13C
P1 9.70 usec
PLW1 46.98899841 W

======== CHANNEL f2 ========
SFO2 400.1316005 MHz
NUC2 1H
CPDPRG[2 waltz16
PCPD2 90.00 usec
PLW2 11.99499989 W
PLW12 0.34213999 W
PLW13 0.27713001 W

F2 - Processing parameters
SI 32768
SF 100.6127750 MHz
WDW EM
SSB 0
LB 1.00 Hz
GB 0
PC 1.40

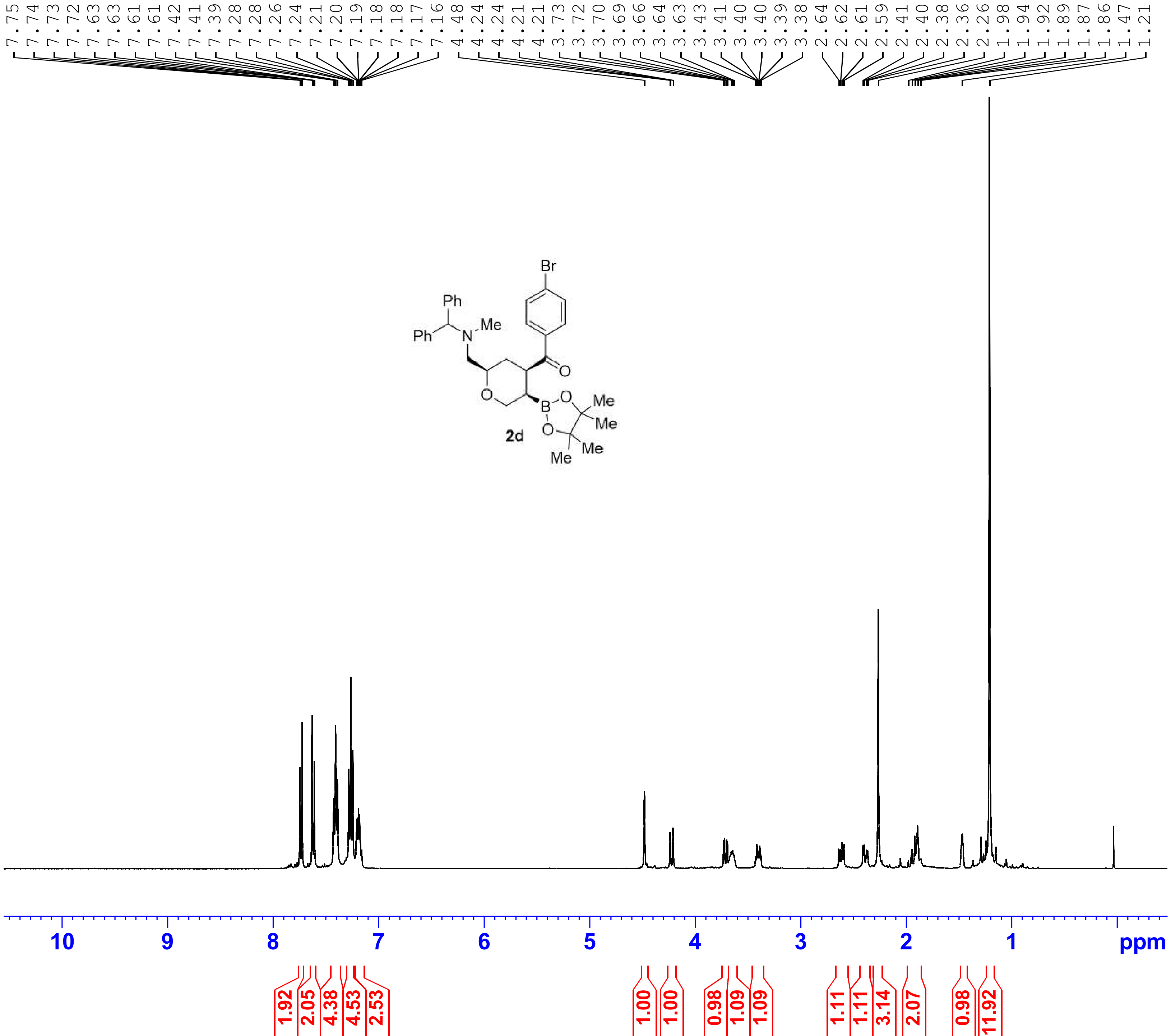

Current Data Parameters
NAME zmh-5-180k-chun
EXPNO 9
PROCNO 1

F2 - Acquisition Parameters
Date_ 20240518
Time 18.46 h
INSTRUM AvanceNeo 400MHz
PROBHD Z163739_0629 (
PULPROG zg30
TD 65536
SOLVENT CDCl3
NS 8
DS 2
SWH 8196.722 Hz
FIDRES 0.250144 Hz
AQ 3.9976959 sec
RG 101
DW 61.000 usec
DE 13.89 usec
TE 297.2 K
D1 1.00000000 sec
TD0 1
SFO1 400.1824711 MHz
NUC1 1H
P0 2.67 usec
P1 8.00 usec
PLW1 21.26700020 W

F2 - Processing parameters
SI 65536
SF 400.1800000 MHz
WDW EM
SSB 0
LB 0.30 Hz
GB 0
PC 1.00

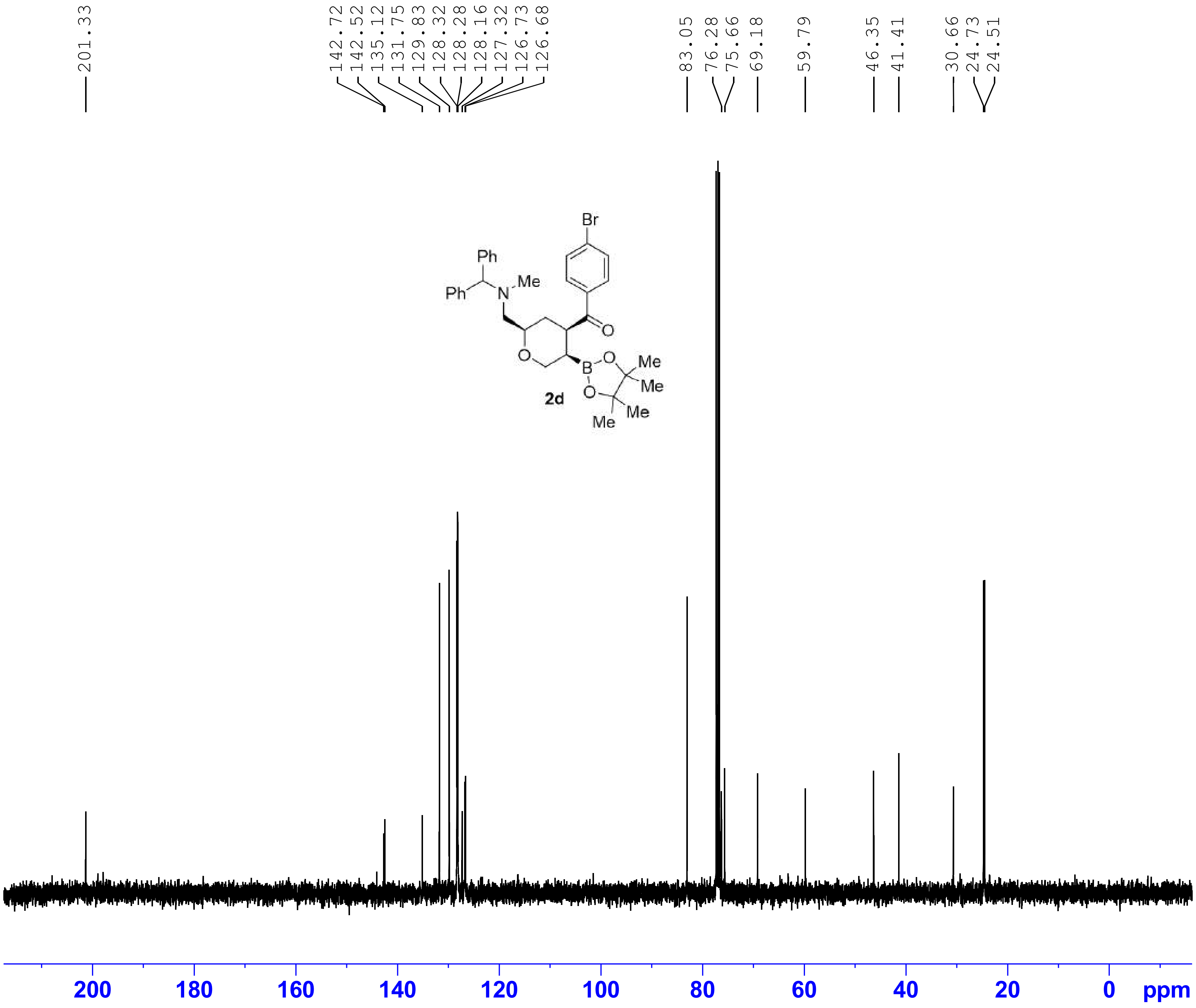

Current Data Parameters
NAME zmh-5-180k-chun
EXPNO 10
PROCNO 1

F2 - Acquisition Parameters
Date_ 20240518
Time_ 18.51 h
INSTRUM AvanceNeo 400MHz
PROBHD Z163739_0629 (
PULPROG zgpg30
TD 65536
SOLVENT CDCl3
NS 52
DS 4
SWH 23809.523 Hz
FIDRES 0.726609 Hz
AQ 1.3762560 sec
RG 10
DW 21.000 usec
DE 6.50 usec
TE 297.7 K
D1 2.00000000 sec
D11 0.03000000 sec
TD0 1
SFO1 100.6354036 MHz
NUC1 13C
P0 2.67 usec
P1 8.00 usec
PLW1 85.25399780 W
SFO2 400.1816007 MHz
NUC2 1H
CPDPRG[2 waltz65
PCPD2 90.00 usec
PLW2 21.26700020 W
PLW12 0.16802999 W
PLW13 0.08452000 W

F2 - Processing parameters
SI 32768
SF 100.6253477 MHz
WDW EM
SSB 0
LB 1.00 Hz
GB 0
PC 1.40

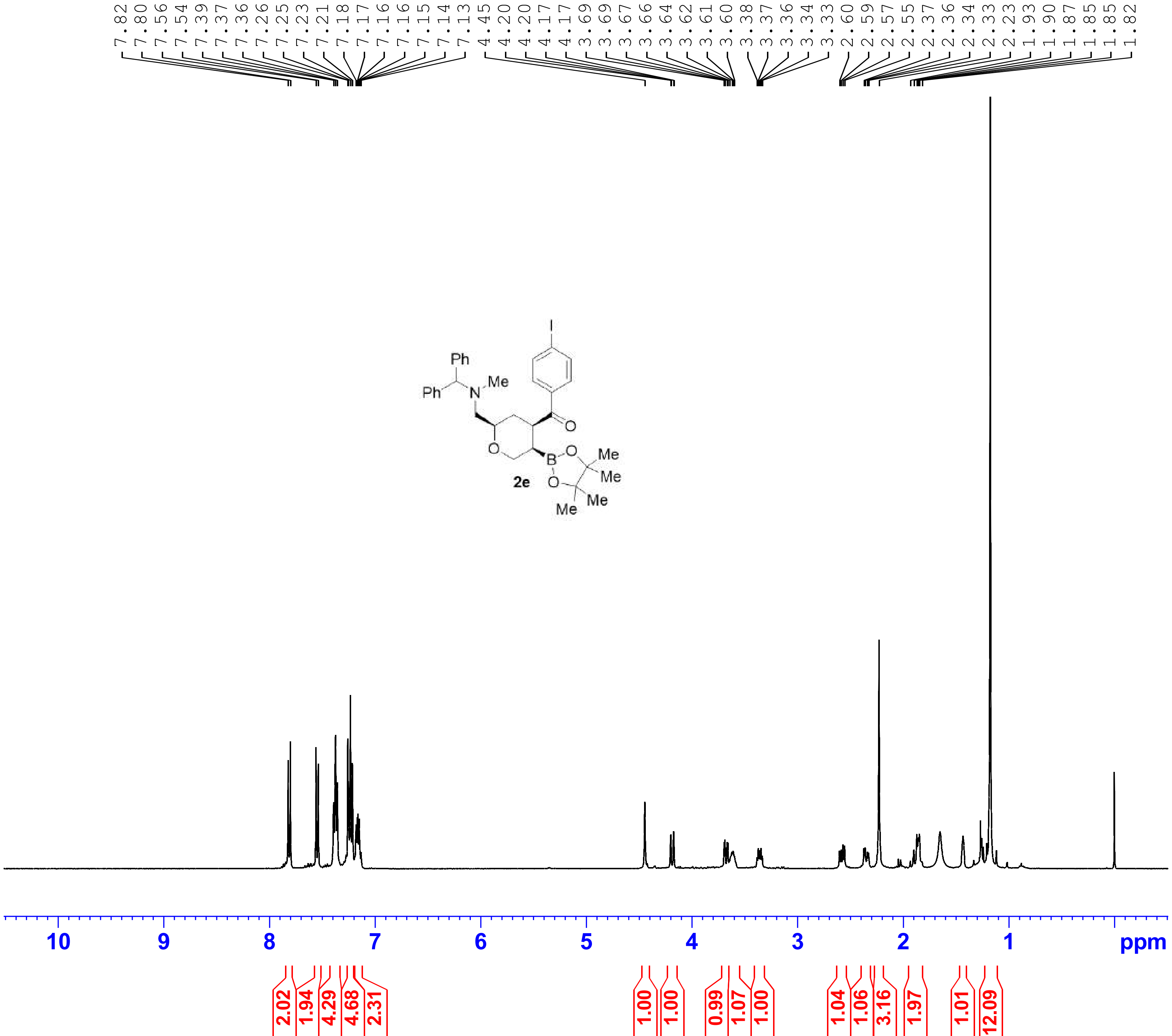

Current Data Parameters
NAME zmh-5-180p
EXPNO 9
PROCNO 1

F2 - Acquisition Parameters
Date_ 20240603
Time 19.09 h
INSTRUM AvanceNeo 400MHz
PROBHD Z163739_0629 (
PULPROG zg30
TD 65536
SOLVENT CDCl3
NS 8
DS 2
SWH 8196.722 Hz
FIDRES 0.250144 Hz
AQ 3.9976959 sec
RG 101
DW 61.000 usec
DE 13.89 usec
TE 296.9 K
D1 1.00000000 sec
TD0 1
SFO1 400.1824711 MHz
NUC1 1H
P0 2.67 usec
P1 8.00 usec
PLW1 21.26700020 W

F2 - Processing parameters
SI 65536
SF 400.1800101 MHz
WDW EM
SSB 0
LB 0.30 Hz
GB 0
PC 1.00

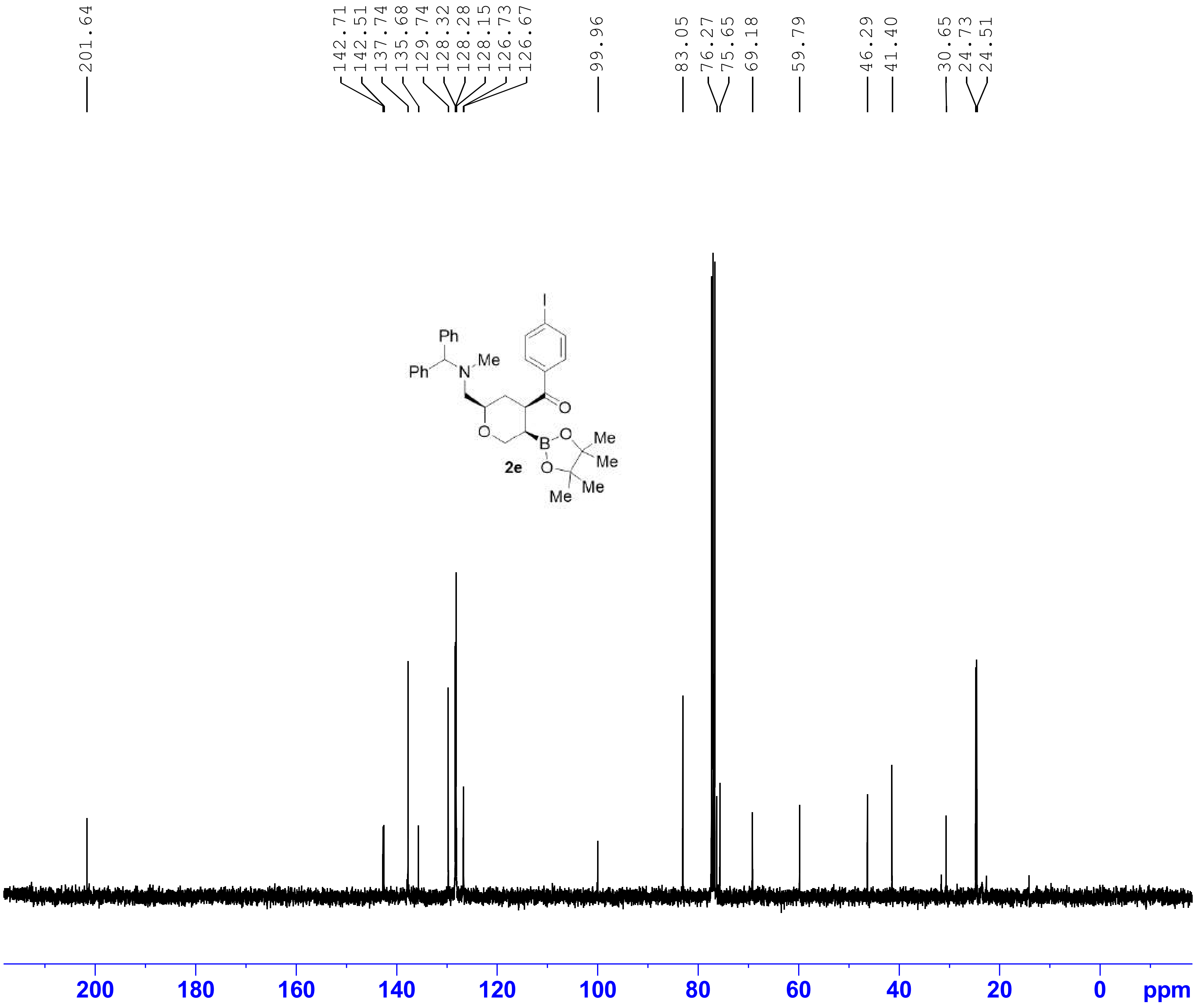

Current Data Parameters
NAME zmh-5-180p-chun
EXPNO 6
PROCNO 1

F2 - Acquisition Parameters
Date_ 20240520
Time 18.45 h
INSTRUM AvanceNeo 400MHz
PROBHD Z163739_0629 (
PULPROG zgpg30
TD 65536
SOLVENT CDCl3
NS 96
DS 4
SWH 23809.523 Hz
FIDRES 0.726609 Hz
AQ 1.3762560 sec
RG 10
DW 21.000 usec
DE 6.50 usec
TE 297.2 K
D1 2.00000000 sec
D11 0.03000000 sec
TD0 1
SFO1 100.6354036 MHz
NUC1 13C
P0 2.67 usec
P1 8.00 usec
PLW1 85.25399780 W
SFO2 400.1816007 MHz
NUC2 1H
CPDPRG[2 waltz65
PCPD2 90.00 usec
PLW2 21.26700020 W
PLW12 0.16802999 W
PLW13 0.08452000 W

F2 - Processing parameters
SI 32768
SF 100.6253477 MHz
WDW EM
SSB 0
LB 1.00 Hz
GB 0
PC 1.40

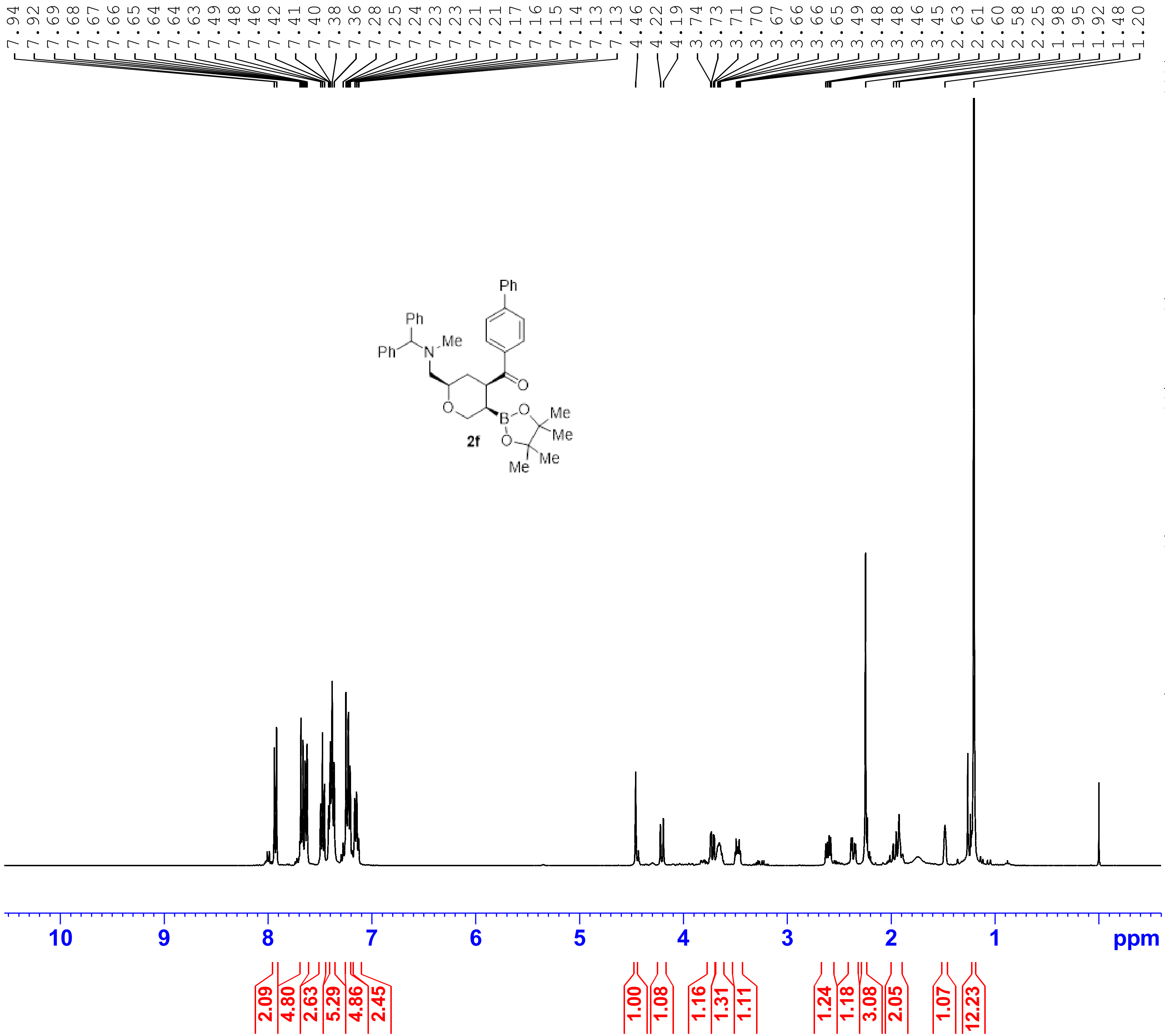

Current Data Parameters
NAME zmh-5-180n
EXPNO 3
PROCNO 1

F2 - Acquisition Parameters
Date_ 20240604
Time 18.57 h
INSTRUM AvanceNeo 400MHz
PROBHD Z163739_0629 (
PULPROG zg30
TD 65536
SOLVENT CDCl3
NS 8
DS 2
SWH 8196.722 Hz
FIDRES 0.250144 Hz
AQ 3.9976959 sec
RG 101
DW 61.000 usec
DE 13.89 usec
TE 296.7 K
D1 1.00000000 sec
TD0 1
SFO1 400.1824711 MHz
NUC1 1H
P0 2.67 usec
P1 8.00 usec
PLW1 21.26700020 W

F2 - Processing parameters
SI 65536
SF 400.1800129 MHz
WDW EM
SSB 0
LB 0.30 Hz
GB 0
PC 1.00

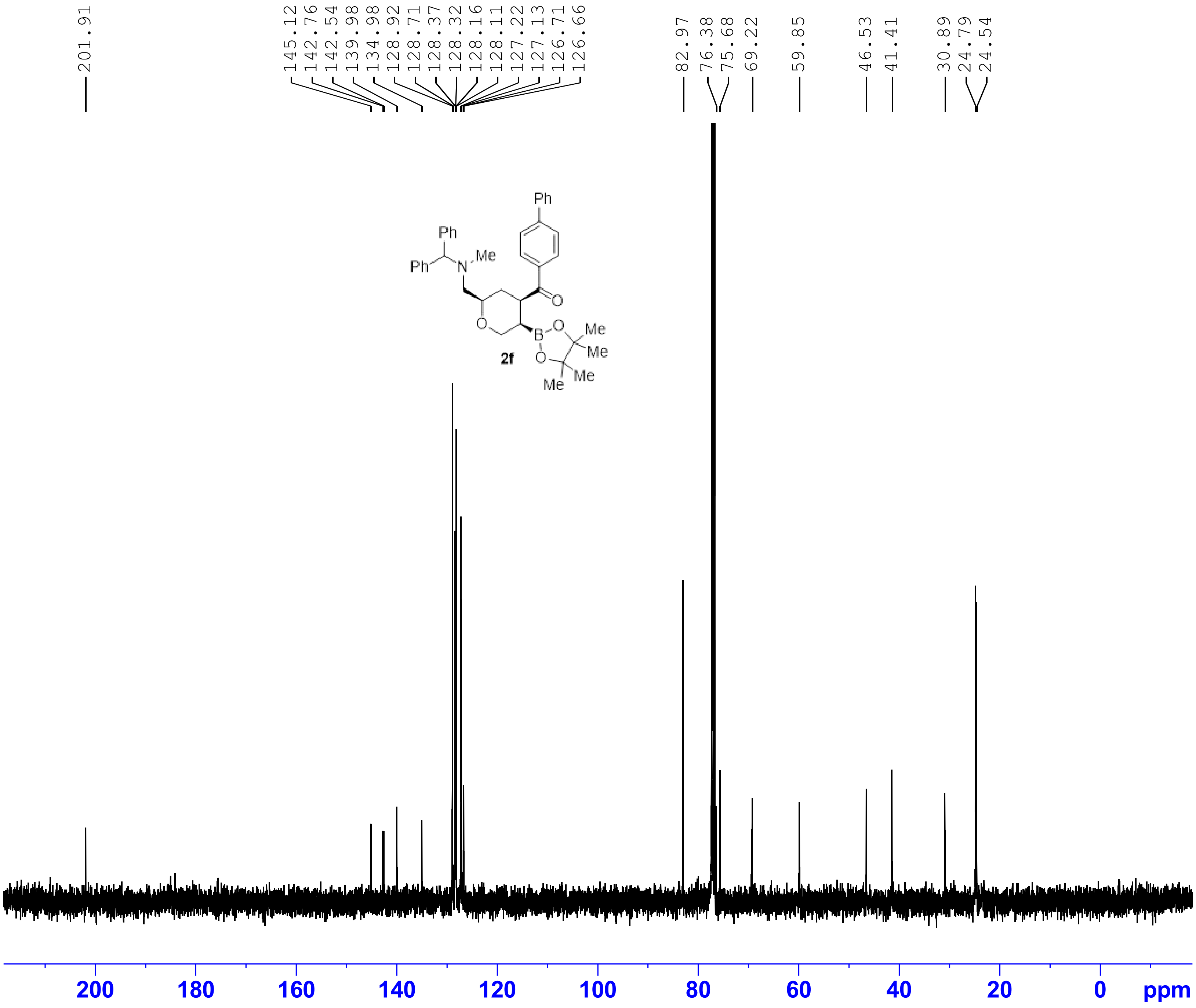


```
Current Data Parameters
NAME            zmh-5-180n
EXPNO                    4
PROCNO                   1

F2 - Acquisition Parameters
Date_             20240604
Time                 19.05 h
INSTRUM    AvanceNeo 400MHz
PROBHD     Z163739_0629 (
PULPROG            zgpg30
TD                  65536
SOLVENT             CDCl3
NS                    106
DS                      4
SWH             23809.523 Hz
FIDRES           0.726609 Hz
AQ              1.3762560 sec
RG                     10
DW                 21.000 usec
DE                   6.50 usec
TE                  297.0 K
D1             2.00000000 sec
D11            0.03000000 sec
TD0                     1
SFO1         100.6354036 MHz
NUC1                  13C
P0                   2.67 usec
P1                   8.00 usec
PLW1          85.25399780 W
SFO2         400.1816007 MHz
NUC2                   1H
CPDPRG[2           waltz65
PCPD2               90.00 usec
PLW2          21.26700020 W
PLW12          0.16802999 W
PLW13          0.08452000 W

F2 - Processing parameters
SI                  32768
SF           100.6253463 MHz
WDW                    EM
SSB                     0
LB                   1.00 Hz
GB                      0
PC                   1.40
```

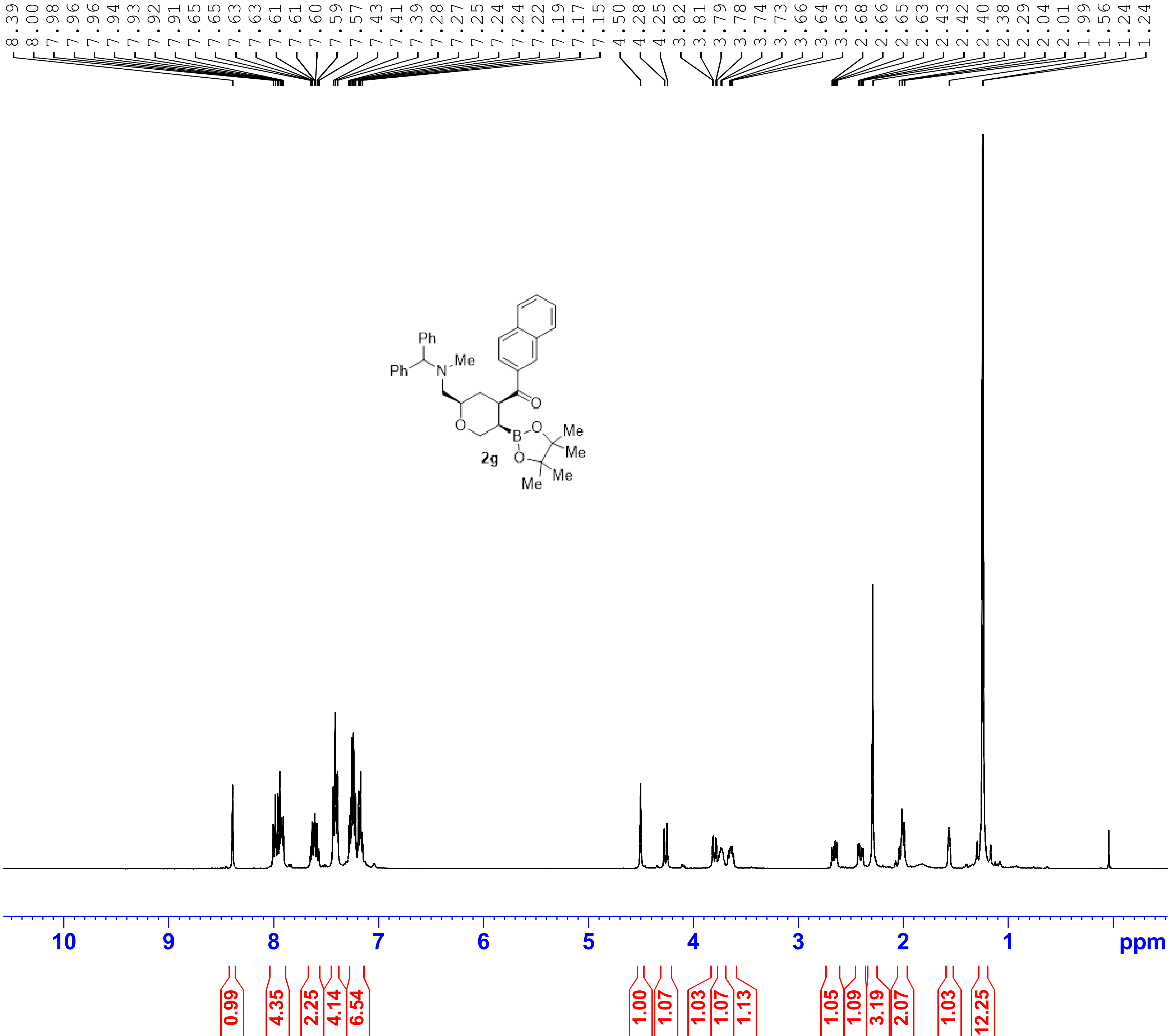

Current Data Parameters
NAME zmh-5-1801-chun
EXPNO 7
PROCNO 1

F2 - Acquisition Parameters
Date_ 20240520
Time 18.49 h
INSTRUM AvanceNeo 400MHz
PROBHD Z163739_0629 (
PULPROG zg30
TD 65536
SOLVENT CDCl3
NS 8
DS 2
SWH 8196.722 Hz
FIDRES 0.250144 Hz
AQ 3.9976959 sec
RG 101
DW 61.000 usec
DE 13.89 usec
TE 296.7 K
D1 1.00000000 sec
TD0 1
SFO1 400.1824711 MHz
NUC1 1H
P0 2.67 usec
P1 8.00 usec
PLW1 21.26700020 W

F2 - Processing parameters
SI 65536
SF 400.1800000 MHz
WDW EM
SSB 0
LB 0.30 Hz
GB 0
PC 1.00

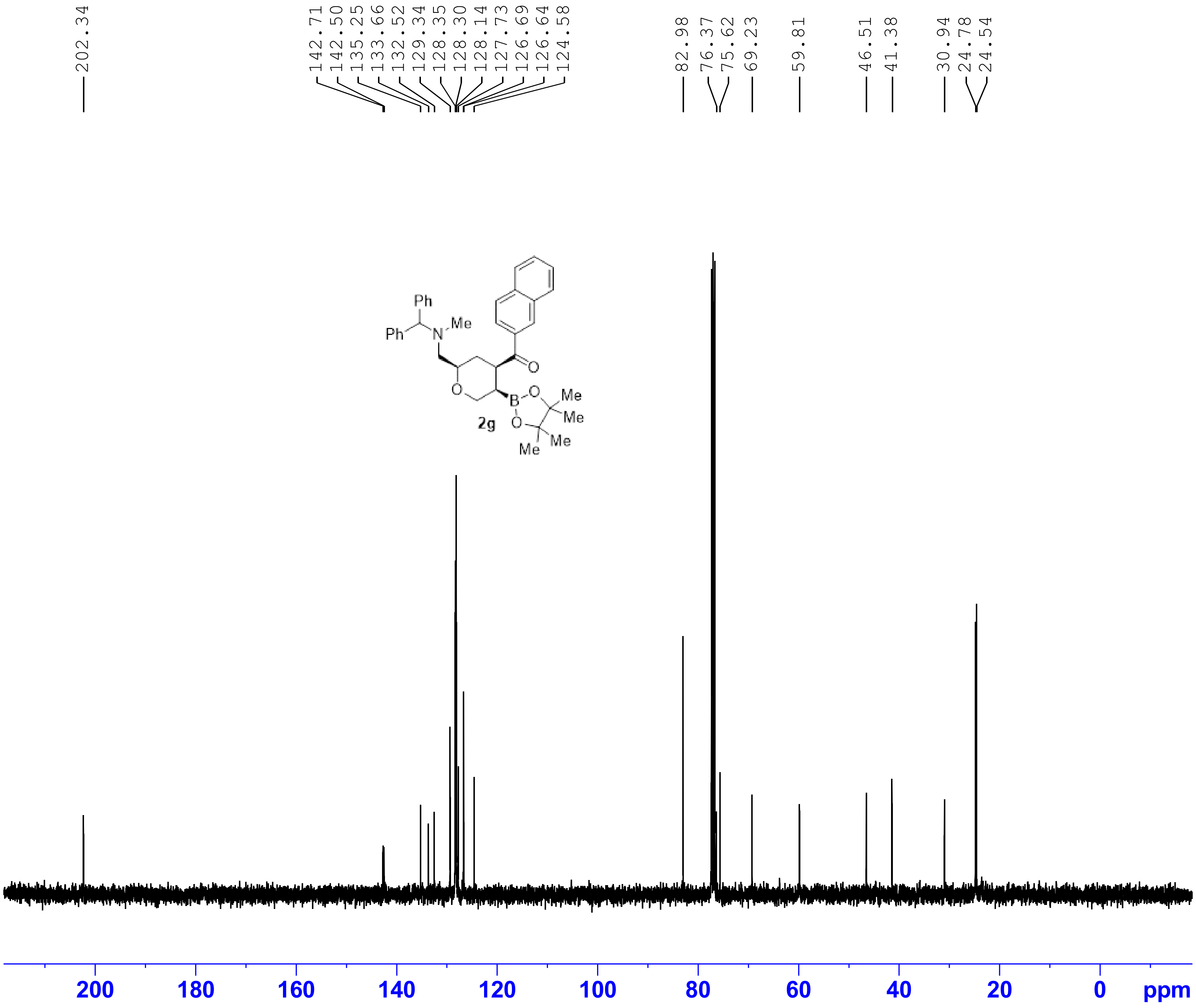

Current Data Parameters
NAME zmh-5-1801-chun
EXPNO 8
PROCNO 1

F2 - Acquisition Parameters
Date_ 20240520
Time 18.55 h
INSTRUM AvanceNeo 400MHz
PROBHD Z163739_0629 (
PULPROG zgpg30
TD 65536
SOLVENT CDCl3
NS 81
DS 4
SWH 23809.523 Hz
FIDRES 0.726609 Hz
AQ 1.3762560 sec
RG 10
DW 21.000 usec
DE 6.50 usec
TE 297.2 K
D1 2.00000000 sec
D11 0.03000000 sec
TD0 1
SFO1 100.6354036 MHz
NUC1 13C
P0 2.67 usec
P1 8.00 usec
PLW1 85.25399780 W
SFO2 400.1816007 MHz
NUC2 1H
CPDPRG[2 waltz65
PCPD2 90.00 usec
PLW2 21.26700020 W
PLW12 0.16802999 W
PLW13 0.08452000 W

F2 - Processing parameters
SI 32768
SF 100.6253484 MHz
WDW EM
SSB 0
LB 1.00 Hz
GB 0
PC 1.40

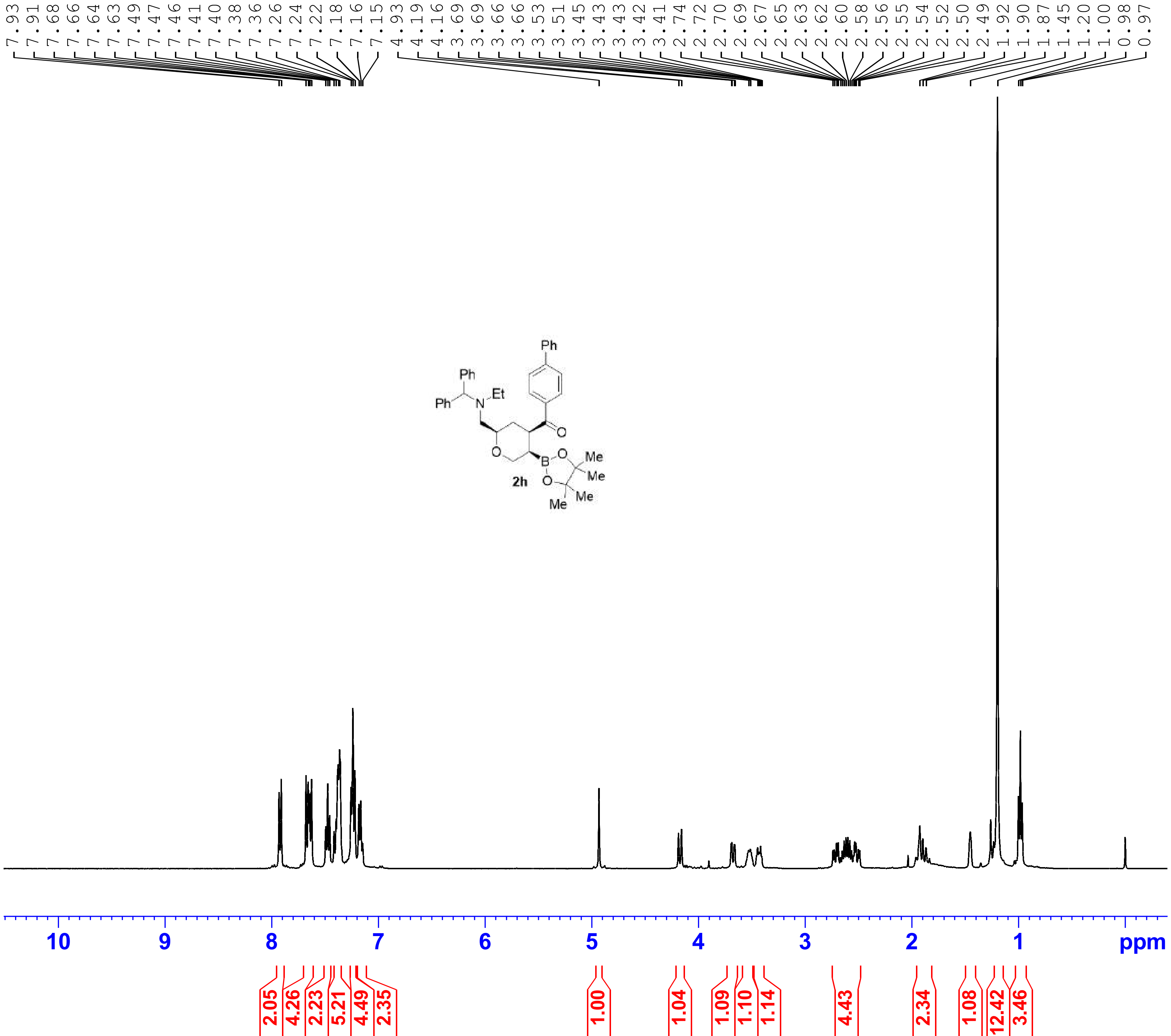

Current Data Parameters
NAME zmh-5-180o
EXPNO 1
PROCNO 1

F2 - Acquisition Parameters
Date_ 20240530
Time 13.33 h
INSTRUM AvanceNeo 400MHz
PROBHD Z163739_0629 (
PULPROG zg30
TD 65536
SOLVENT CDCl3
NS 8
DS 2
SWH 8196.722 Hz
FIDRES 0.250144 Hz
AQ 3.9976959 sec
RG 101
DW 61.000 usec
DE 13.89 usec
TE 304.1 K
D1 1.00000000 sec
TD0 1
SFO1 400.1824711 MHz
NUC1 1H
P0 2.67 usec
P1 8.00 usec
PLW1 21.26700020 W

F2 - Processing parameters
SI 65536
SF 400.1800152 MHz
WDW EM
SSB 0
LB 0.30 Hz
GB 0
PC 1.00

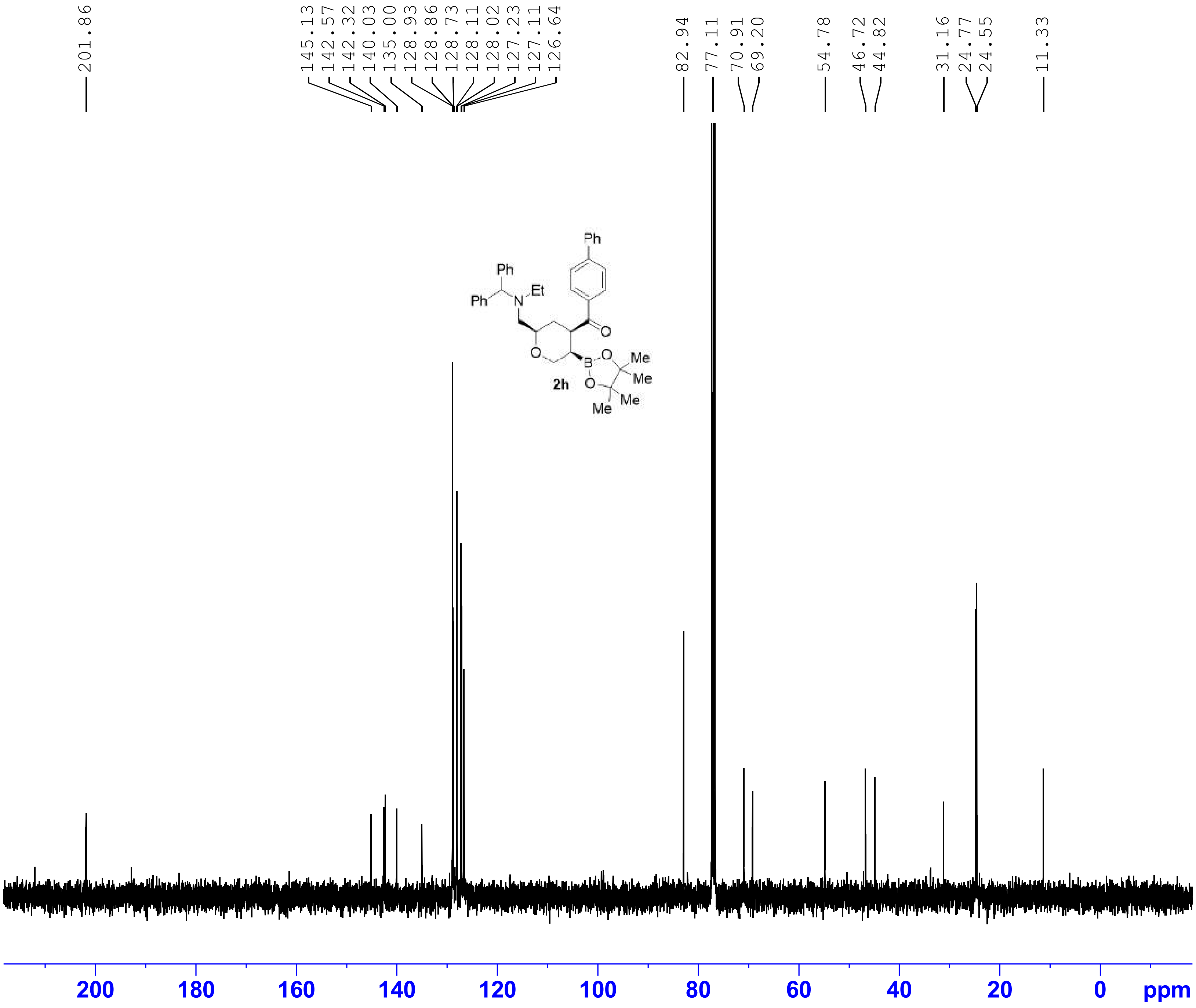
201.86
145.13
142.57
142.32
140.03
135.00
128.93
128.86
128.73
128.11
128.02
127.23
127.11
126.64
82.94
77.11
70.91
69.20
54.78
46.72
44.82
31.16
24.77
24.55
11.33
Ph
Ph
Ph
N
Et
O
O
B
O
Me
Me
Me
Me
2h
200
180
160
140
120
100
80
60
40
20
0
ppm

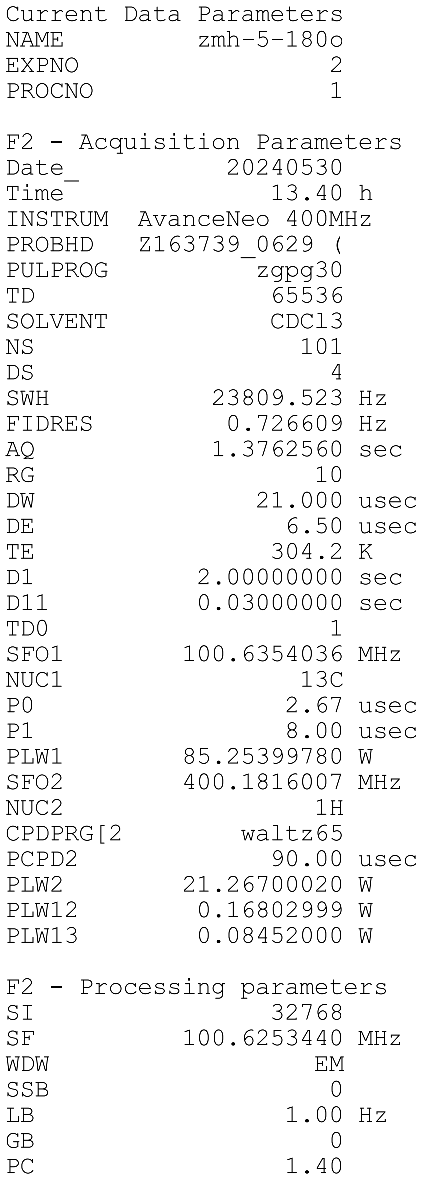
Current Data Parameters
NAME zmh-5-180o
EXPNO 2
PROCNO 1
F2 - Acquisition Parameters
Date_ 20240530
Time 13.40 h
INSTRUM AvanceNeo 400MHz
PROBHD Z163739_0629 (
PULPROG zgpg30
TD 65536
SOLVENT CDCl3
NS 101
DS 4
SWH 23809.523 Hz
FIDRES 0.726609 Hz
AQ 1.3762560 sec
RG 10
DW 21.000 usec
DE 6.50 usec
TE 304.2 K
D1 2.00000000 sec
D11 0.03000000 sec
TD0 1
SFO1 100.6354036 MHz
NUC1 13C
P0 2.67 usec
P1 8.00 usec
PLW1 85.25399780 W
SFO2 400.1816007 MHz
NUC2 1H
CPDPRG[2 waltz65
PCPD2 90.00 usec
PLW2 21.26700020 W
PLW12 0.16802999 W
PLW13 0.08452000 W
F2 - Processing parameters
SI 32768
SF 100.6253440 MHz
WDW EM
SSB 0
LB 1.00 Hz
GB 0
PC 1.40

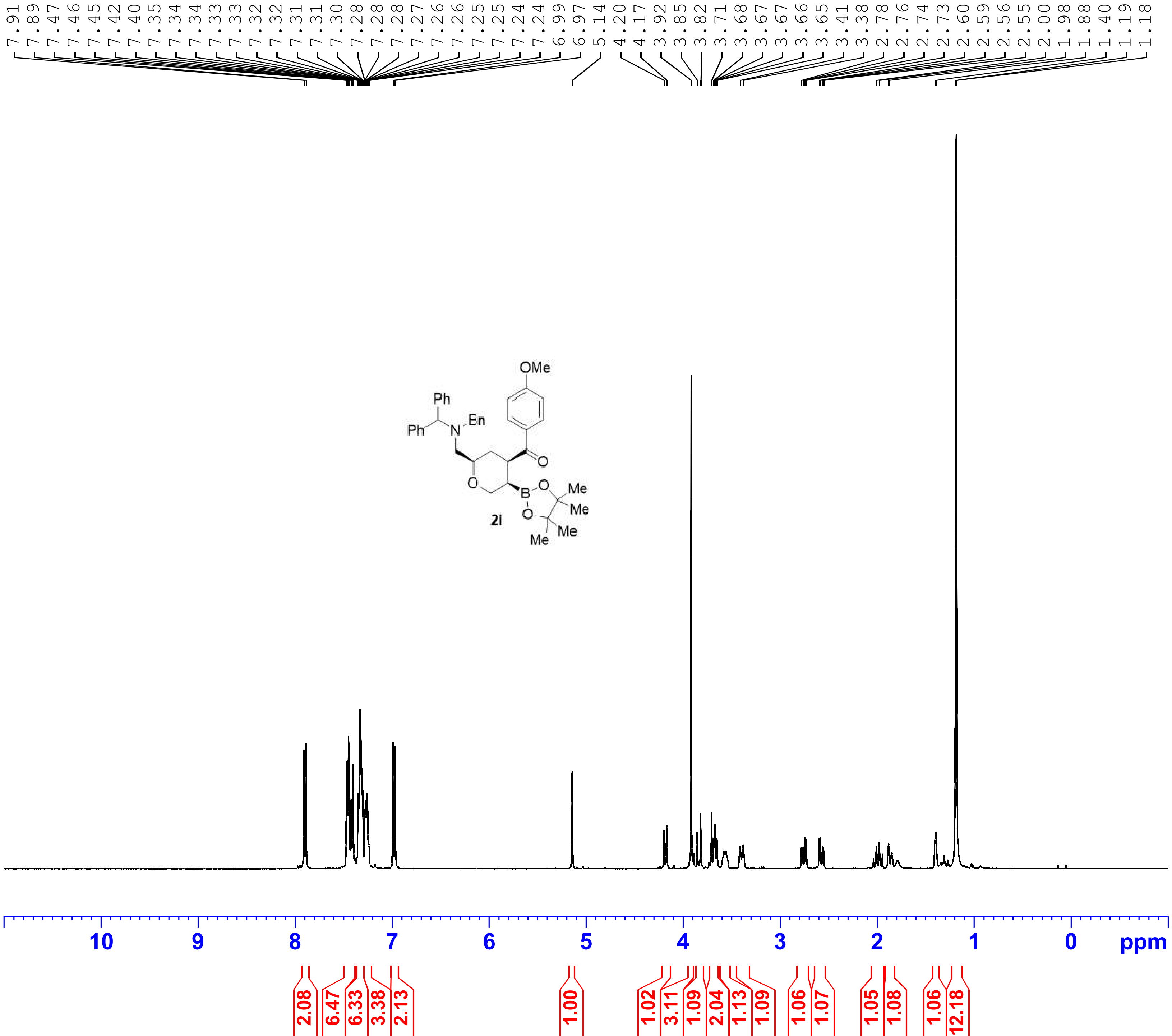

Current Data Parameters
NAME zmh-5-180b-chun-2
EXPNO 1
PROCNO 1

F2 - Acquisition Parameters
Date_ 20240516
Time 13.08 h
INSTRUM AvanceNeo 400MHz
PROBHD Z163739_0629 (
PULPROG zg30
TD 65536
SOLVENT CDCl3
NS 8
DS 2
SWH 8196.722 Hz
FIDRES 0.250144 Hz
AQ 3.9976959 sec
RG 45.2
DW 61.000 usec
DE 13.89 usec
TE 296.7 K
D1 1.00000000 sec
TD0 1
SFO1 400.1824711 MHz
NUC1 1H
P0 2.67 usec
P1 8.00 usec
PLW1 21.26700020 W

F2 - Processing parameters
SI 65536
SF 400.1800000 MHz
WDW EM
SSB 0
LB 0.30 Hz
GB 0
PC 1.00

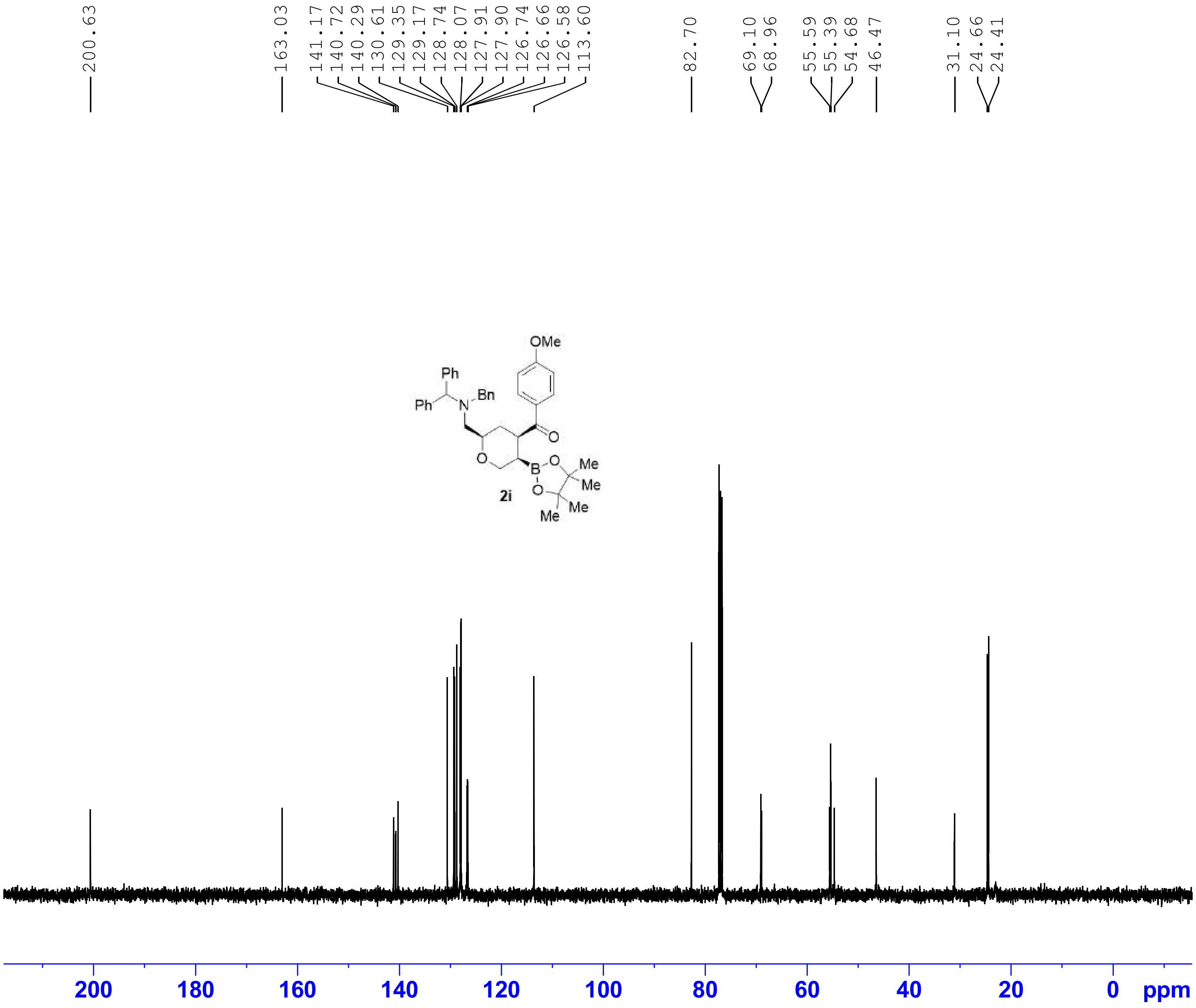

Current Data Parameters
NAME zmh-5-180b-chun-2
EXPNO 2
PROCNO 1

F2 - Acquisition Parameters
Date_ 20240516
Time 13.15 h
INSTRUM AvanceNeo 400MHz
PROBHD Z163739_0629 (
PULPROG zgpg30
TD 65536
SOLVENT CDCl3
NS 74
DS 4
SWH 23809.523 Hz
FIDRES 0.726609 Hz
AQ 1.3762560 sec
RG 10
DW 21.000 usec
DE 6.50 usec
TE 297.3 K
D1 2.00000000 sec
D11 0.03000000 sec
TD0 1
SFO1 100.6354036 MHz
NUC1 13C
P0 2.67 usec
P1 8.00 usec
PLW1 85.25399780 W
SFO2 400.1816007 MHz
NUC2 1H
CPDPRG[2 waltz65
PCPD2 90.00 usec
PLW2 21.26700020 W
PLW12 0.16802999 W
PLW13 0.08452000 W

F2 - Processing parameters
SI 32768
SF 100.6253512 MHz
WDW EM
SSB 0
LB 1.00 Hz
GB 0
PC 1.40

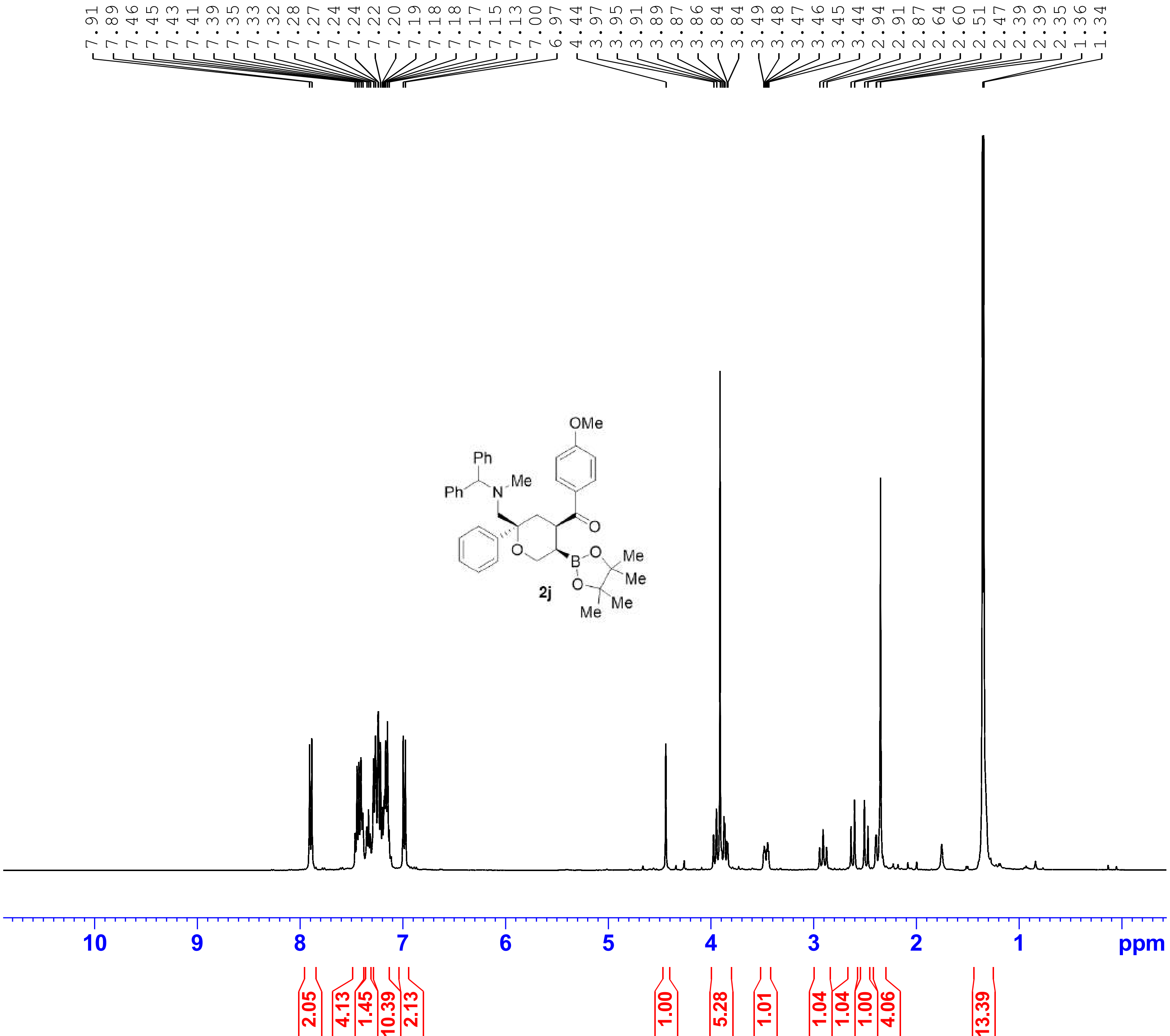

Current Data Parameters
NAME zmh-5-180d-chun-2
EXPNO 5
PROCNO 1

F2 - Acquisition Parameters
Date_ 20240516
Time 13.19 h
INSTRUM AvanceNeo 400MHz
PROBHD Z163739_0629 (
PULPROG zg30
TD 65536
SOLVENT CDCl3
NS 8
DS 2
SWH 8196.722 Hz
FIDRES 0.250144 Hz
AQ 3.9976959 sec
RG 45.2
DW 61.000 usec
DE 13.89 usec
TE 296.8 K
D1 1.00000000 sec
TD0 1
SFO1 400.1824711 MHz
NUC1 1H
P0 2.67 usec
P1 8.00 usec
PLW1 21.26700020 W

F2 - Processing parameters
SI 65536
SF 400.1800000 MHz
WDW EM
SSB 0
LB 0.30 Hz
GB 0
PC 1.00

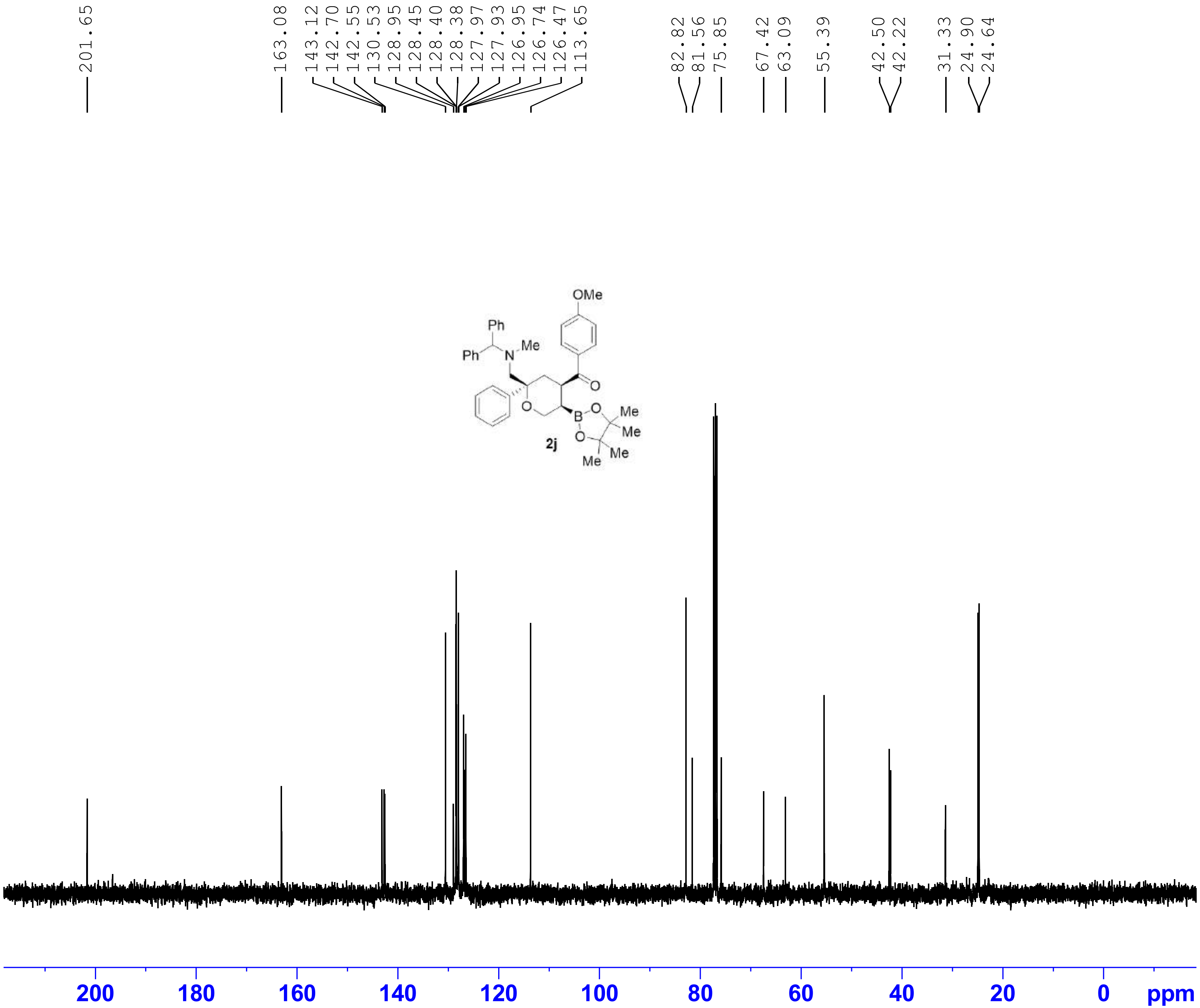

Current Data Parameters
NAME zmh-5-180d-chun-2
EXPNO 6
PROCNO 1

F2 - Acquisition Parameters
Date_ 20240516
Time 13.25 h
INSTRUM AvanceNeo 400MHz
PROBHD Z163739_0629 (
PULPROG zgpg30
TD 65536
SOLVENT CDCl3
NS 68
DS 4
SWH 23809.523 Hz
FIDRES 0.726609 Hz
AQ 1.3762560 sec
RG 10
DW 21.000 usec
DE 6.50 usec
TE 297.3 K
D1 2.00000000 sec
D11 0.03000000 sec
TD0 1
SFO1 100.6354036 MHz
NUC1 13C
P0 2.67 usec
P1 8.00 usec
PLW1 85.25399780 W
SFO2 400.1816007 MHz
NUC2 1H
CPDPRG[2 waltz65
PCPD2 90.00 usec
PLW2 21.26700020 W
PLW12 0.16802999 W
PLW13 0.08452000 W

F2 - Processing parameters
SI 32768
SF 100.6253515 MHz
WDW EM
SSB 0
LB 1.00 Hz
GB 0
PC 1.40

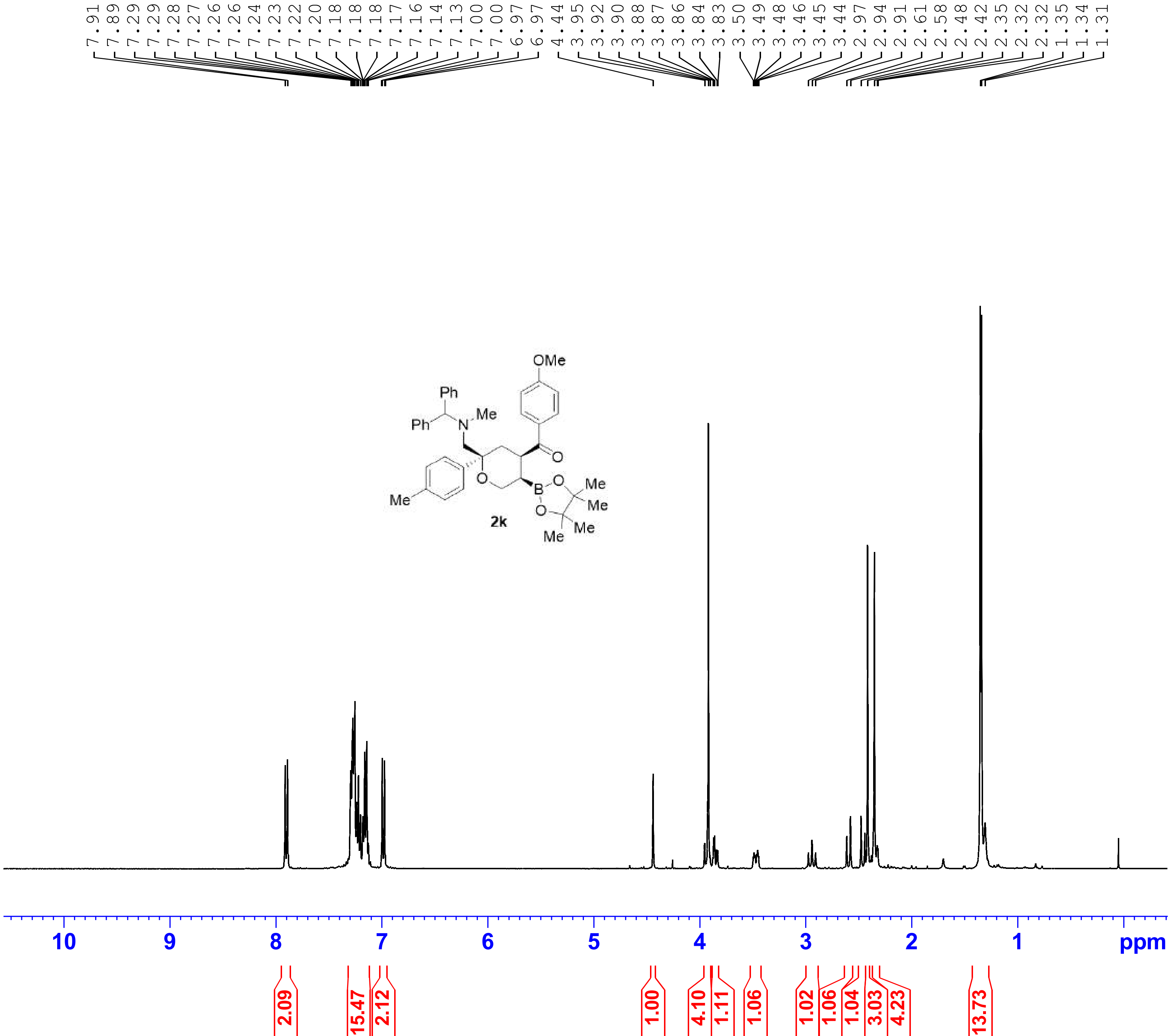

Current Data Parameters
NAME zmh-5-180h-chun
EXPNO 7
PROCNO 1

F2 - Acquisition Parameters
Date_ 20240518
Time 18.29 h
INSTRUM AvanceNeo 400MHz
PROBHD Z163739_0629 (
PULPROG zg30
TD 65536
SOLVENT CDCl3
NS 8
DS 2
SWH 8196.722 Hz
FIDRES 0.250144 Hz
AQ 3.9976959 sec
RG 45.2
DW 61.000 usec
DE 13.89 usec
TE 297.3 K
D1 1.00000000 sec
TD0 1
SFO1 400.1824711 MHz
NUC1 1H
P0 2.67 usec
P1 8.00 usec
PLW1 21.26700020 W

F2 - Processing parameters
SI 65536
SF 400.1800000 MHz
WDW EM
SSB 0
LB 0.30 Hz
GB 0
PC 1.00

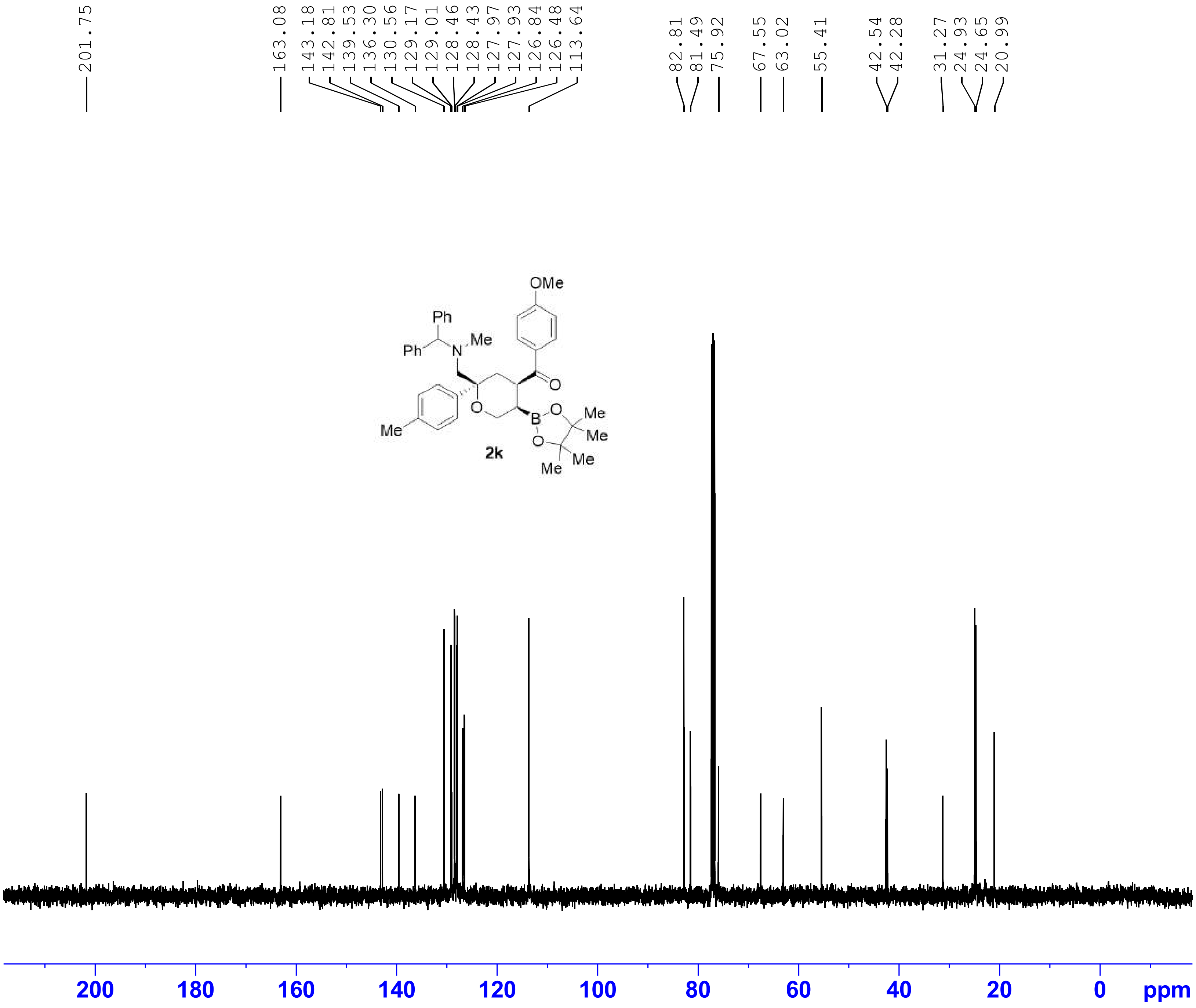

Current Data Parameters
NAME zmh-5-180h-chun
EXPNO 8
PROCNO 1

F2 - Acquisition Parameters
Date_ 20240518
Time 18.34 h
INSTRUM AvanceNeo 400MHz
PROBHD Z163739_0629 (
PULPROG zgpg30
TD 65536
SOLVENT CDCl3
NS 42
DS 4
SWH 23809.523 Hz
FIDRES 0.726609 Hz
AQ 1.3762560 sec
RG 10
DW 21.000 usec
DE 6.50 usec
TE 297.9 K
D1 2.00000000 sec
D11 0.03000000 sec
TD0 1
SFO1 100.6354036 MHz
NUC1 13C
P0 2.67 usec
P1 8.00 usec
PLW1 85.25399780 W
SFO2 400.1816007 MHz
NUC2 1H
CPDPRG[2 waltz65
PCPD2 90.00 usec
PLW2 21.26700020 W
PLW12 0.16802999 W
PLW13 0.08452000 W

F2 - Processing parameters
SI 32768
SF 100.6253493 MHz
WDW EM
SSB 0
LB 1.00 Hz
GB 0
PC 1.40

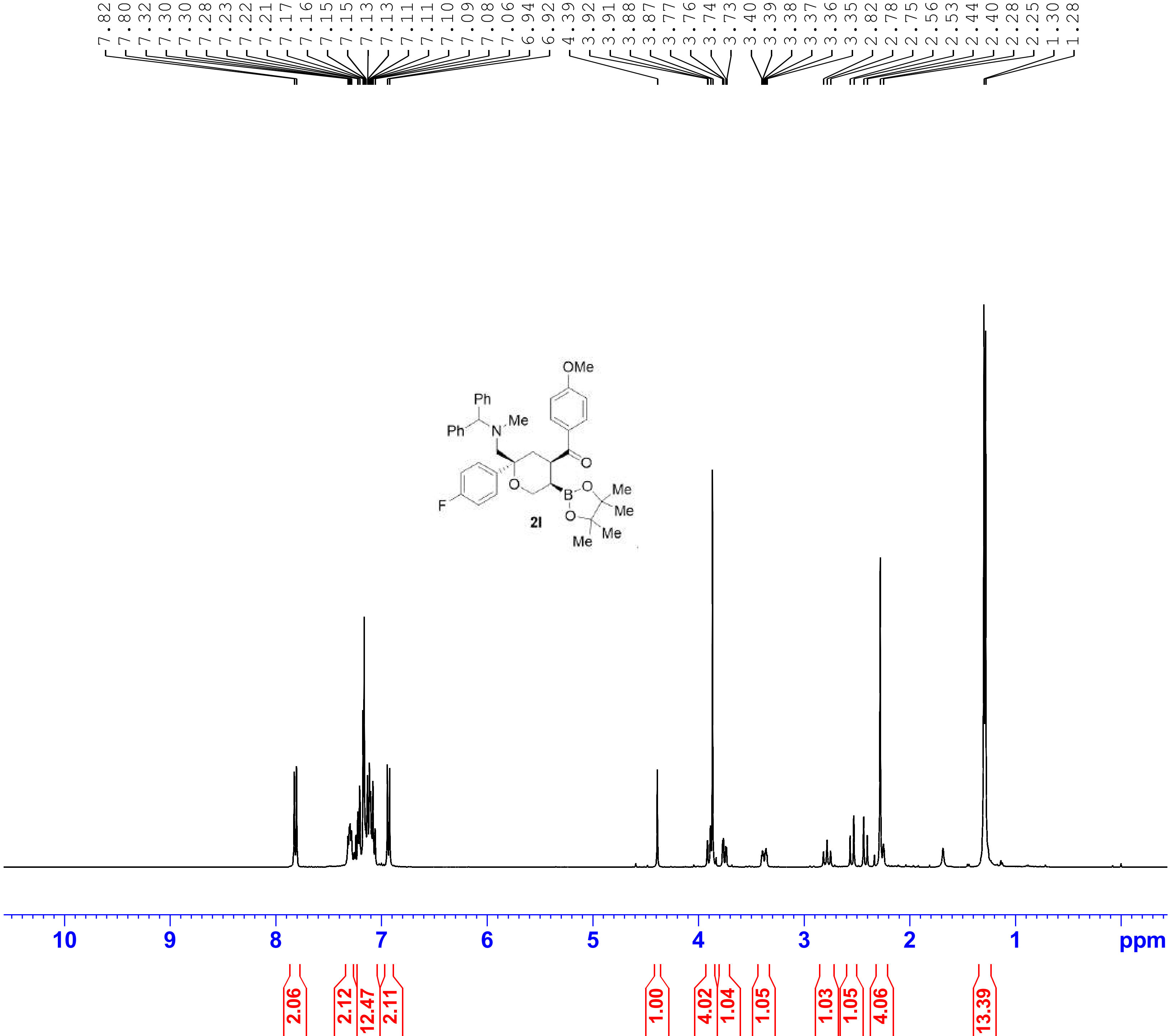

Current Data Parameters
NAME zmh-5-180e-chun-2
EXPNO 7
PROCNO 1

F2 - Acquisition Parameters
Date_ 20240516
Time 13.30 h
INSTRUM AvanceNeo 400MHz
PROBHD Z163739_0629 (
PULPROG zg30
TD 65536
SOLVENT CDCl3
NS 8
DS 2
SWH 8196.722 Hz
FIDRES 0.250144 Hz
AQ 3.9976959 sec
RG 45.2
DW 61.000 usec
DE 13.89 usec
TE 296.8 K
D1 1.00000000 sec
TD0 1
SFO1 400.1824711 MHz
NUC1 1H
P0 2.67 usec
P1 8.00 usec
PLW1 21.26700020 W

F2 - Processing parameters
SI 65536
SF 400.1800163 MHz
WDW EM
SSB 0
LB 0.30 Hz
GB 0
PC 1.00

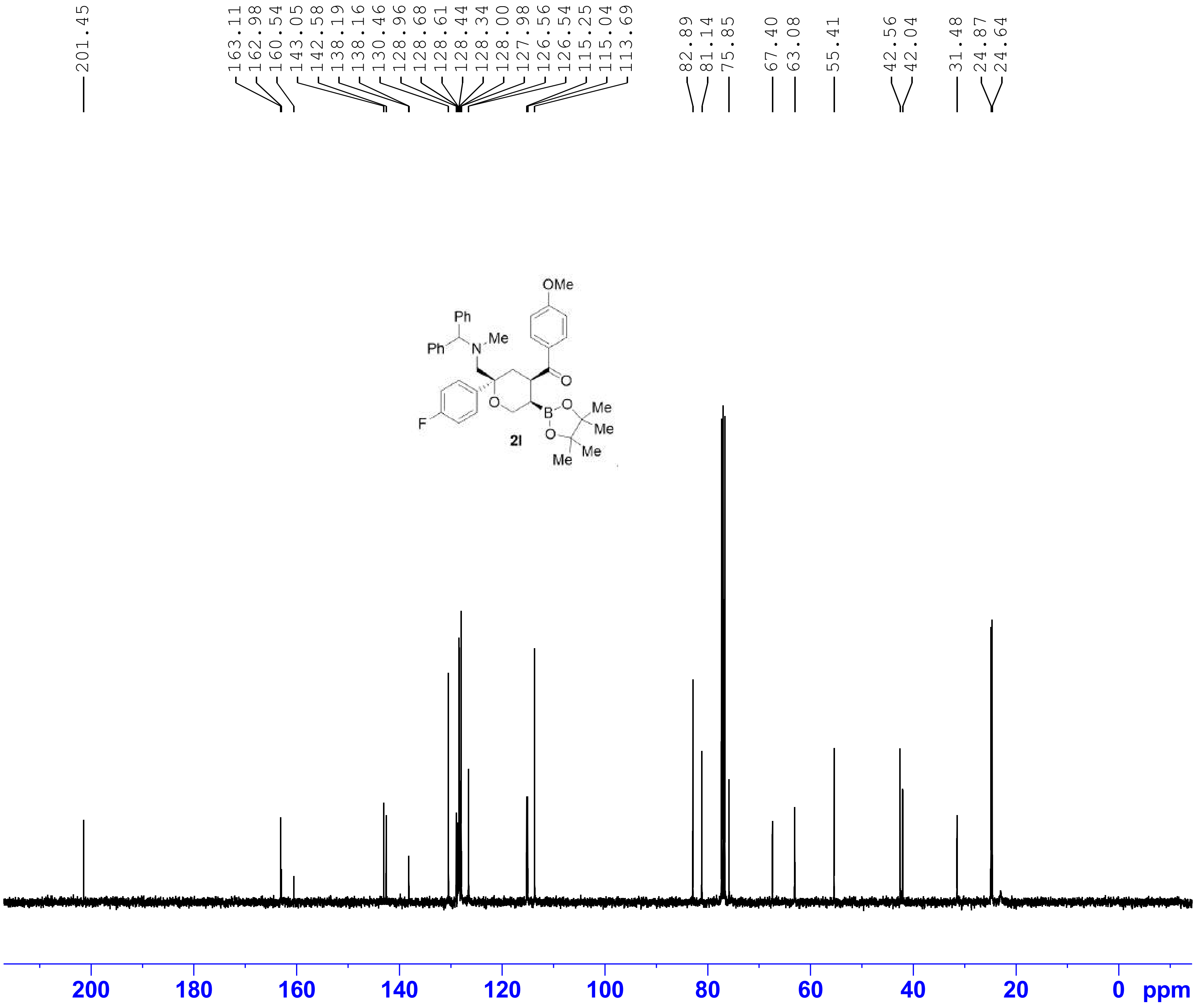

Current Data Parameters
NAME zmh-5-180e-chun-2
EXPNO 8
PROCNO 1

F2 - Acquisition Parameters
Date_ 20240516
Time 13.40 h
INSTRUM AvanceNeo 400MHz
PROBHD Z163739_0629 (
PULPROG zgpg30
TD 65536
SOLVENT CDCl3
NS 151
DS 4
SWH 23809.523 Hz
FIDRES 0.726609 Hz
AQ 1.3762560 sec
RG 10
DW 21.000 usec
DE 6.50 usec
TE 297.4 K
D1 2.00000000 sec
D11 0.03000000 sec
TD0 1
SFO1 100.6354036 MHz
NUC1 13C
P0 2.67 usec
P1 8.00 usec
PLW1 85.25399780 W
SFO2 400.1816007 MHz
NUC2 1H
CPDPRG[2 waltz65
PCPD2 90.00 usec
PLW2 21.26700020 W
PLW12 0.16802999 W
PLW13 0.08452000 W

F2 - Processing parameters
SI 32768
SF 100.6253492 MHz
WDW EM
SSB 0
LB 1.00 Hz
GB 0
PC 1.40

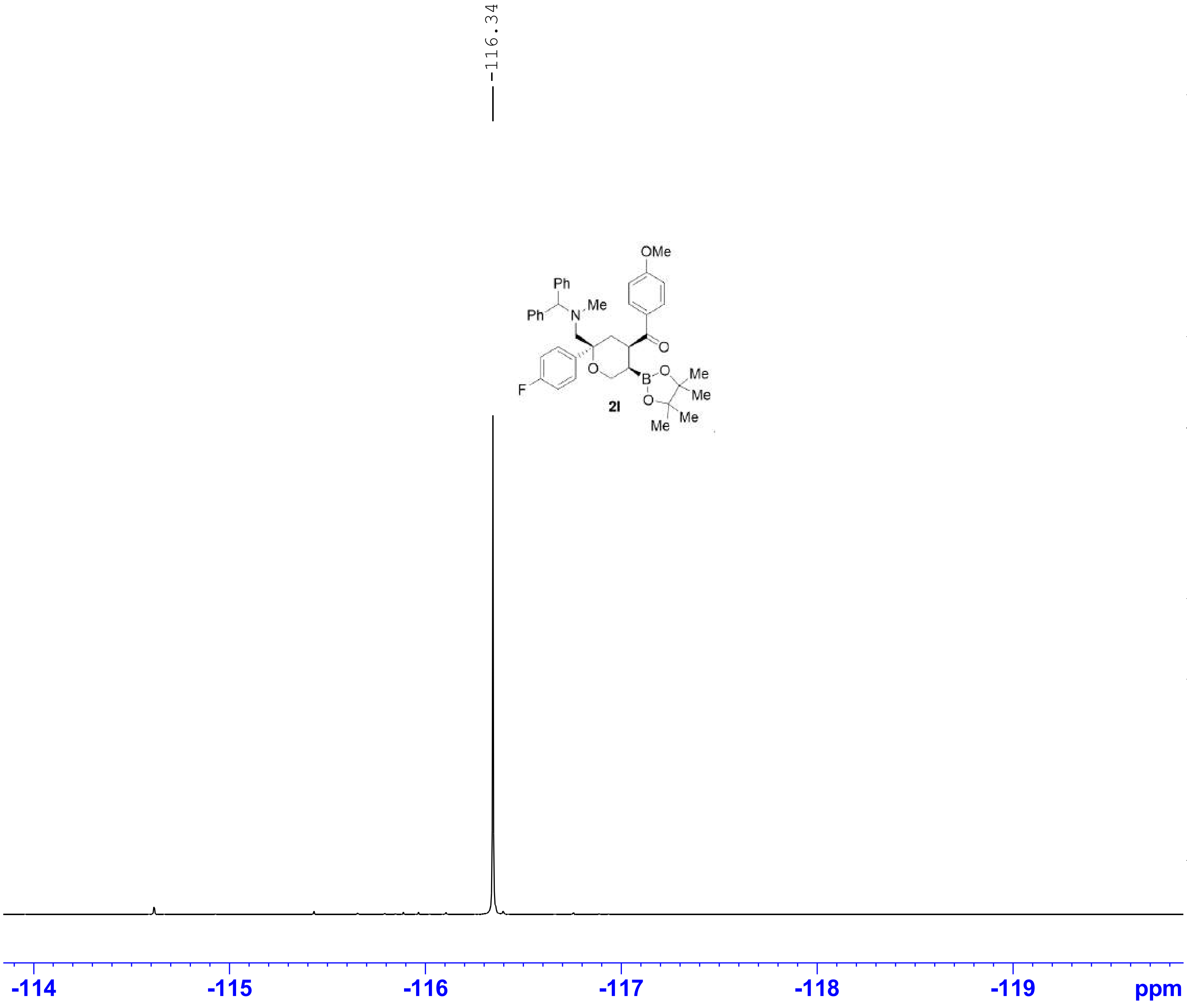

Current Data Parameters
NAME zmh-5-180e-chun
EXPNO 11
PROCNO 1

F2 - Acquisition Parameters
Date_ 20240515
Time 13.53 h
INSTRUM AvanceNeo 400MHz
PROBHD Z163739_0629 (
PULPROG zgig
TD 131072
SOLVENT CDCl3
NS 16
DS 4
SWH 90909.094 Hz
FIDRES 1.387163 Hz
AQ 0.7208960 sec
RG 101
DW 5.500 usec
DE 6.50 usec
TE 296.8 K
D1 1.00000000 sec
D11 0.03000000 sec
TD0 1
SFO1 376.5077587 MHz
NUC1 19F
P1 12.00 usec
PLW1 33.72800064 W
SFO2 400.1816007 MHz
NUC2 1H
CPDPRG[2 waltz16
PCPD2 90.00 usec
PLW2 21.26700020 W
PLW12 0.16802999 W

F2 - Processing parameters
SI 65536
SF 376.5454132 MHz
WDW EM
SSB 0
LB 0.30 Hz
GB 0
PC 1.00

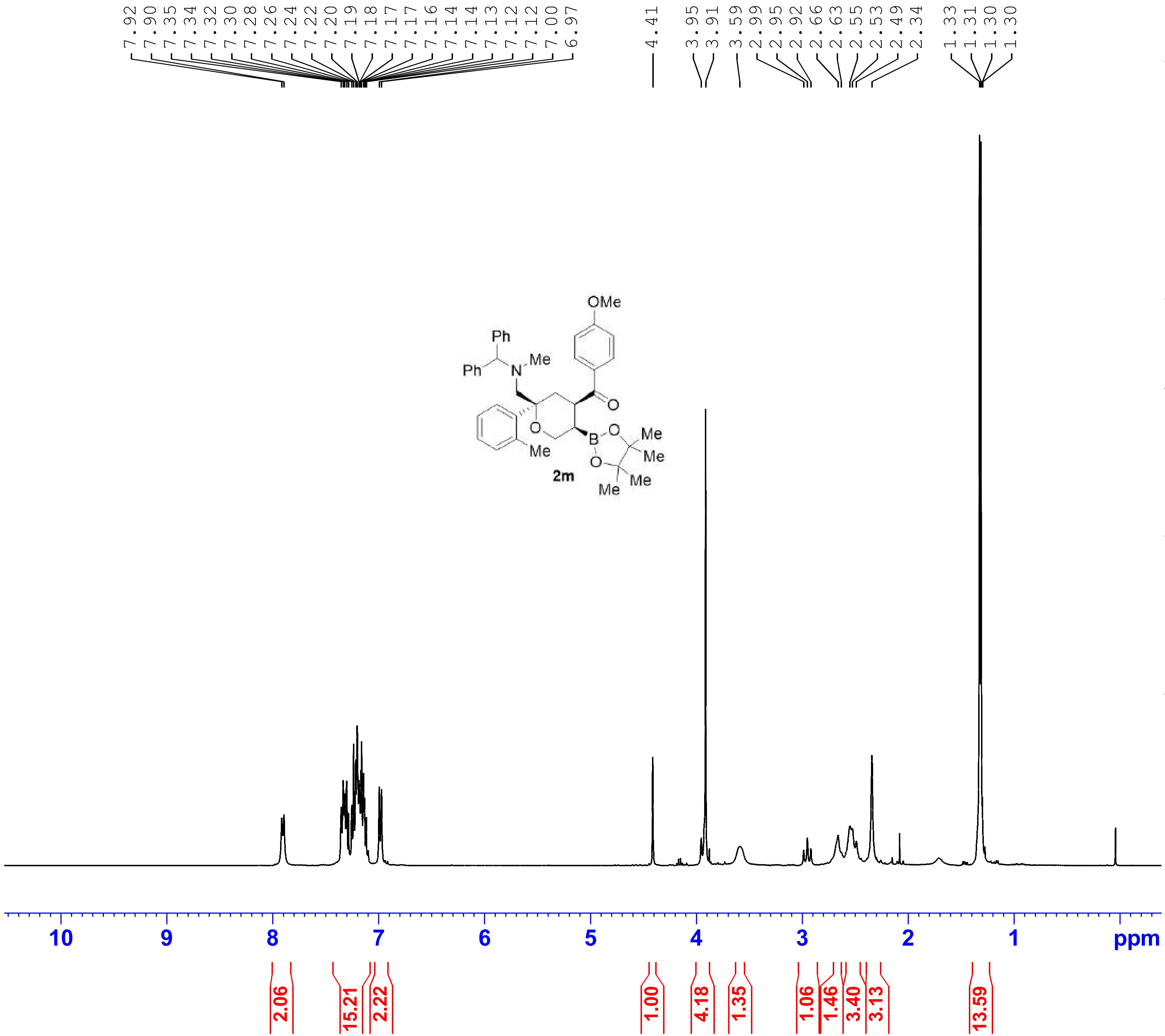

Current Data Parameters
NAME zmh-5-180f
EXPNO 5
PROCNO 1

F2 - Acquisition Parameters
Date_ 20240603
Time 18.58 h
INSTRUM AvanceNeo 400MHz
PROBHD Z163739_0629 (
PULPROG zg30
TD 65536
SOLVENT CDCl3
NS 8
DS 2
SWH 8196.722 Hz
FIDRES 0.250144 Hz
AQ 3.9976959 sec
RG 101
DW 61.000 usec
DE 13.89 usec
TE 296.8 K
D1 1.00000000 sec
TD0 1
SFO1 400.1824711 MHz
NUC1 1H
P0 2.67 usec
P1 8.00 usec
PLW1 21.26700020 W

F2 - Processing parameters
SI 65536
SF 400.1800000 MHz
WDW EM
SSB 0
LB 0.30 Hz
GB 0
PC 1.00

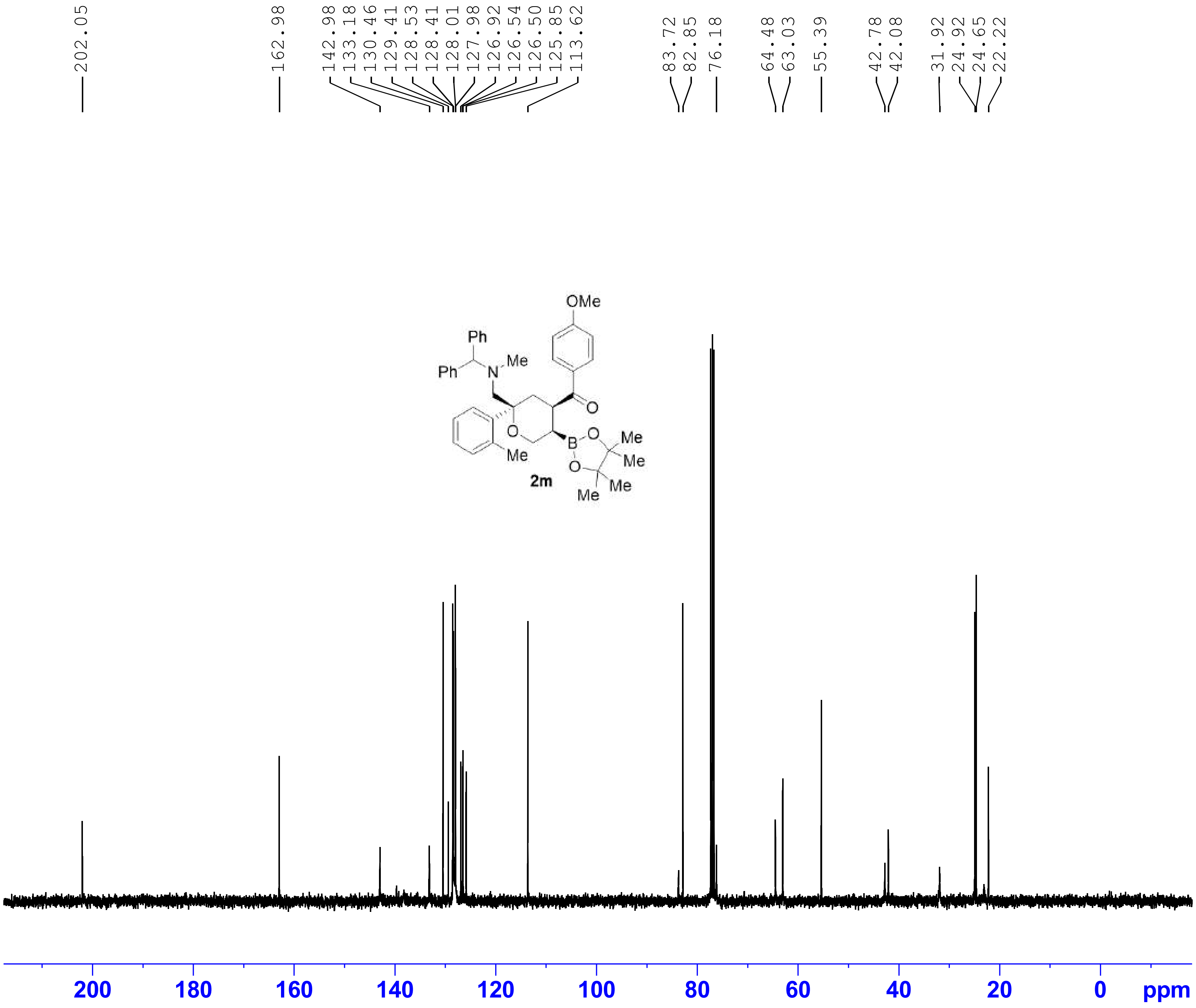

Current Data Parameters
NAME zmh-5-180f-chun-2
EXPNO 10
PROCNO 1

F2 - Acquisition Parameters
Date_ 20240516
Time 13.53 h
INSTRUM AvanceNeo 400MHz
PROBHD Z163739_0629 (
PULPROG zgpg30
TD 65536
SOLVENT CDCl3
NS 104
DS 4
SWH 23809.523 Hz
FIDRES 0.726609 Hz
AQ 1.3762560 sec
RG 10
DW 21.000 usec
DE 6.50 usec
TE 297.2 K
D1 2.00000000 sec
D11 0.03000000 sec
TD0 1
SFO1 100.6354036 MHz
NUC1 13C
P0 2.67 usec
P1 8.00 usec
PLW1 85.25399780 W
SFO2 400.1816007 MHz
NUC2 1H
CPDPRG[2 waltz65
PCPD2 90.00 usec
PLW2 21.26700020 W
PLW12 0.16802999 W
PLW13 0.08452000 W

F2 - Processing parameters
SI 32768
SF 100.6253506 MHz
WDW EM
SSB 0
LB 1.00 Hz
GB 0
PC 1.40

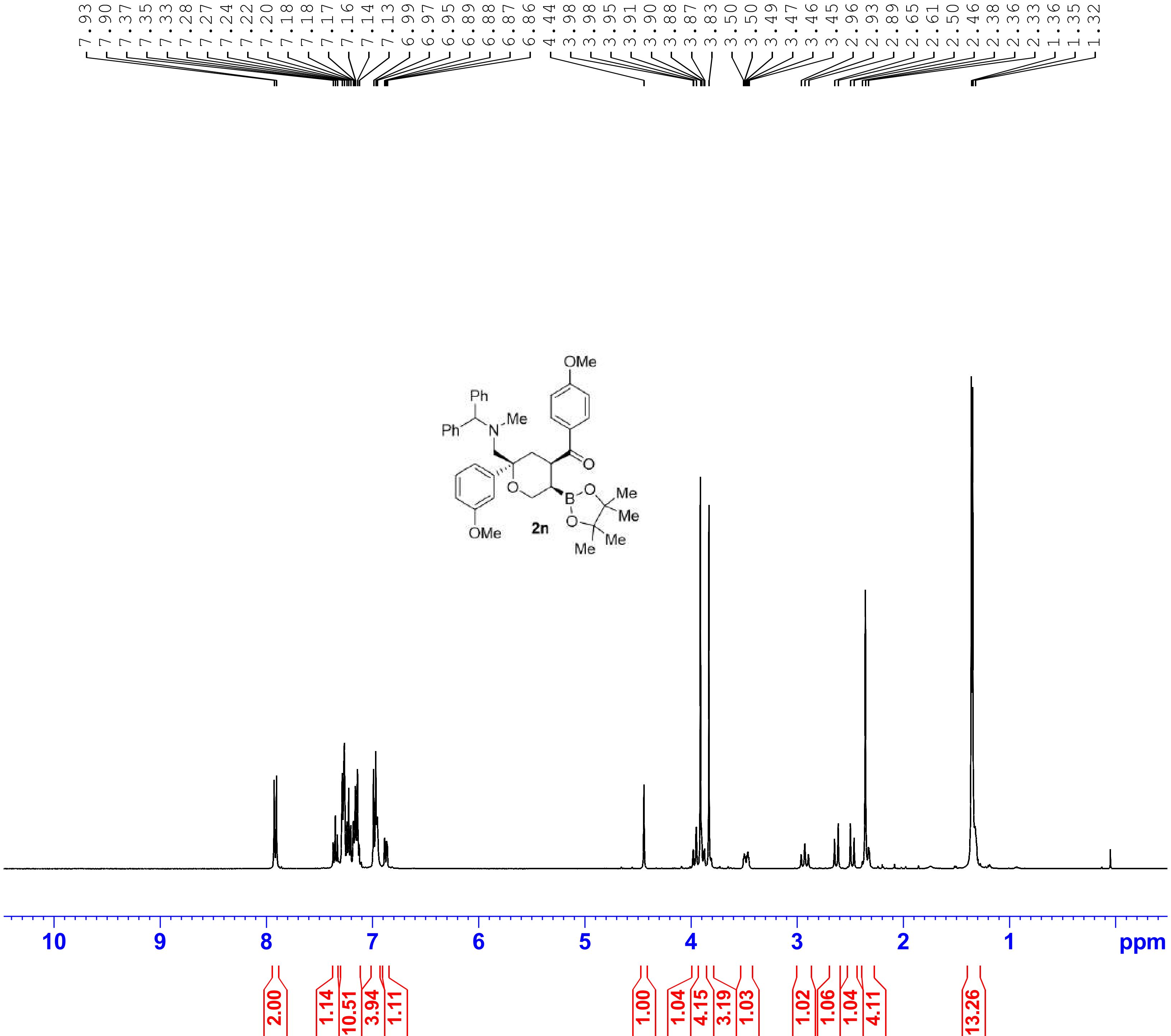

Current Data Parameters
NAME zmh-5-180g-chun
EXPNO 1
PROCNO 1

F2 - Acquisition Parameters
Date_ 20240517
Time 13.04 h
INSTRUM AvanceNeo 400MHz
PROBHD Z163739_0629 (
PULPROG zg30
TD 65536
SOLVENT CDCl3
NS 8
DS 2
SWH 8196.722 Hz
FIDRES 0.250144 Hz
AQ 3.9976959 sec
RG 45.2
DW 61.000 usec
DE 13.89 usec
TE 296.8 K
D1 1.00000000 sec
TD0 1
SFO1 400.1824711 MHz
NUC1 1H
P0 2.67 usec
P1 8.00 usec
PLW1 21.26700020 W

F2 - Processing parameters
SI 65536
SF 400.1800000 MHz
WDW EM
SSB 0
LB 0.30 Hz
GB 0
PC 1.00

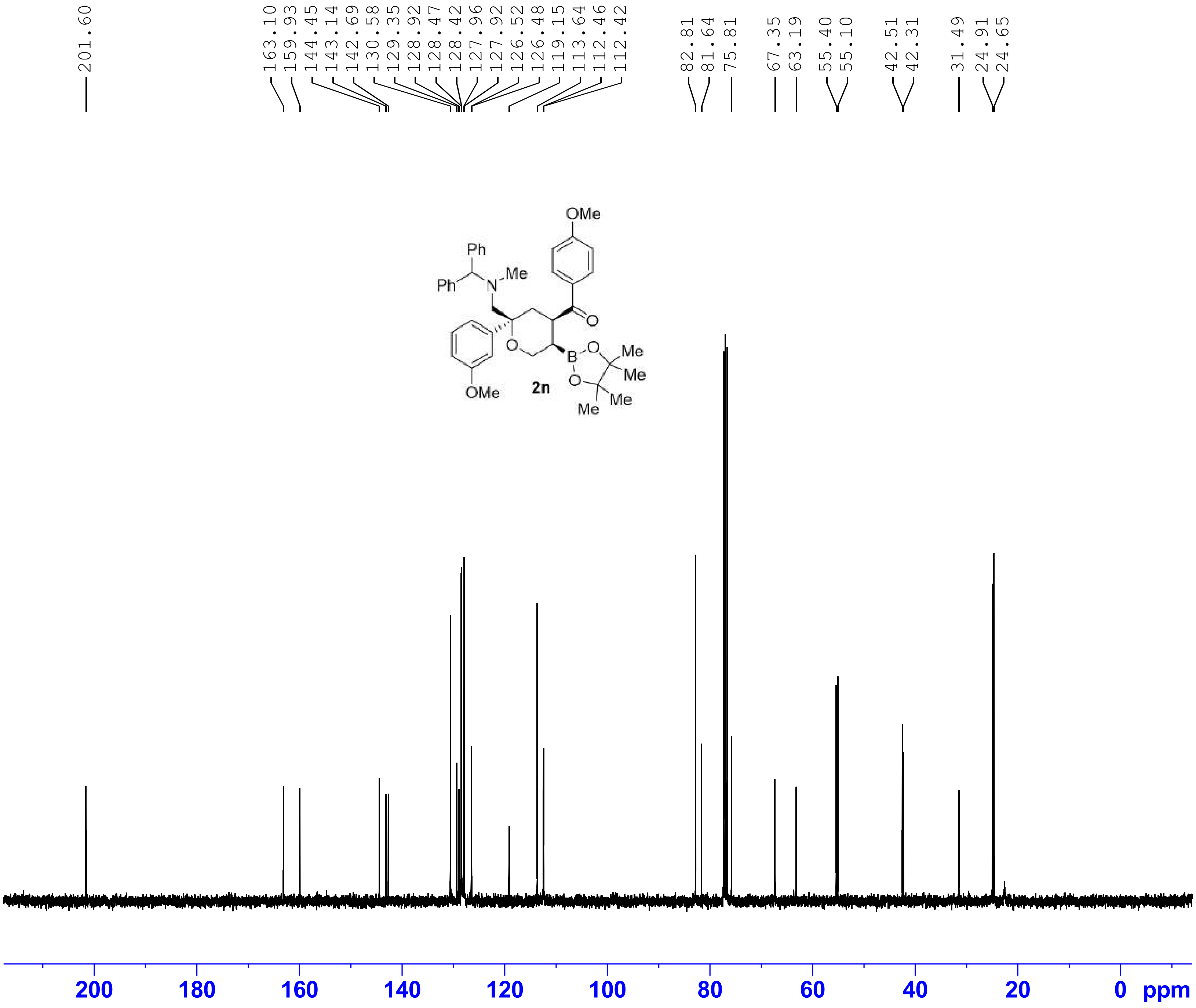

Current Data Parameters
NAME zmh-5-180g-chun
EXPNO 2
PROCNO 1

F2 - Acquisition Parameters
Date_ 20240517
Time 13.11 h
INSTRUM AvanceNeo 400MHz
PROBHD Z163739_0629 (
PULPROG zgpg30
TD 65536
SOLVENT CDCl3
NS 80
DS 4
SWH 23809.523 Hz
FIDRES 0.726609 Hz
AQ 1.3762560 sec
RG 10
DW 21.000 usec
DE 6.50 usec
TE 297.6 K
D1 2.00000000 sec
D11 0.03000000 sec
TD0 1
SFO1 100.6354036 MHz
NUC1 13C
P0 2.67 usec
P1 8.00 usec
PLW1 85.25399780 W
SFO2 400.1816007 MHz
NUC2 1H
CPDPRG[2 waltz65
PCPD2 90.00 usec
PLW2 21.26700020 W
PLW12 0.16802999 W
PLW13 0.08452000 W

F2 - Processing parameters
SI 32768
SF 100.6253506 MHz
WDW EM
SSB 0
LB 1.00 Hz
GB 0
PC 1.40

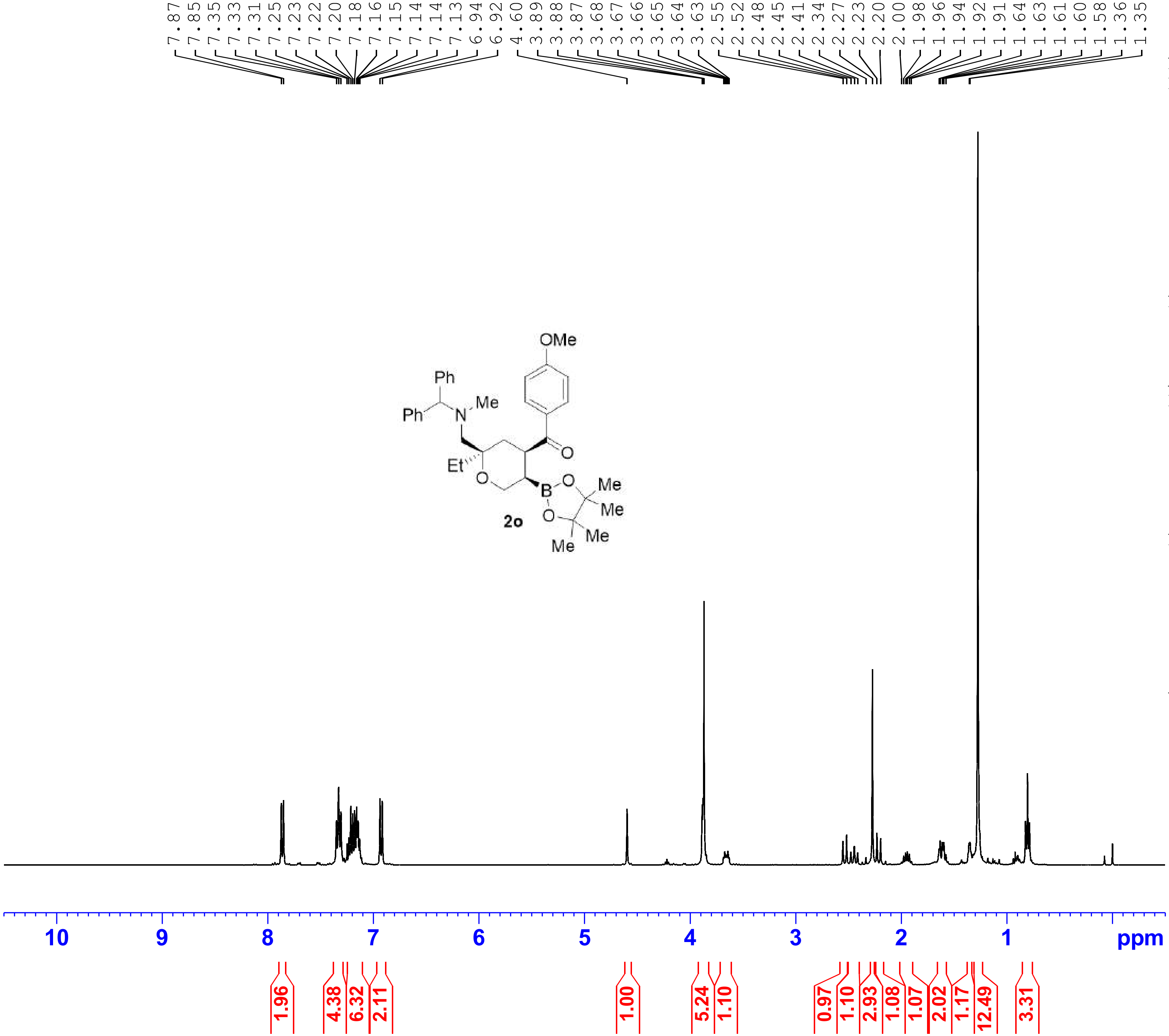

Current Data Parameters
NAME zmh-5-180i-chun
EXPNO 7
PROCNO 1

F2 - Acquisition Parameters
Date_ 20240517
Time 13.35 h
INSTRUM AvanceNeo 400MHz
PROBHD Z163739_0629 (
PULPROG zg30
TD 65536
SOLVENT CDCl3
NS 8
DS 2
SWH 8196.722 Hz
FIDRES 0.250144 Hz
AQ 3.9976959 sec
RG 101
DW 61.000 usec
DE 13.89 usec
TE 296.8 K
D1 1.00000000 sec
TD0 1
SFO1 400.1824711 MHz
NUC1 1H
P0 2.67 usec
P1 8.00 usec
PLW1 21.26700020 W

F2 - Processing parameters
SI 65536
SF 400.1800134 MHz
WDW EM
SSB 0
LB 0.30 Hz
GB 0
PC 1.00

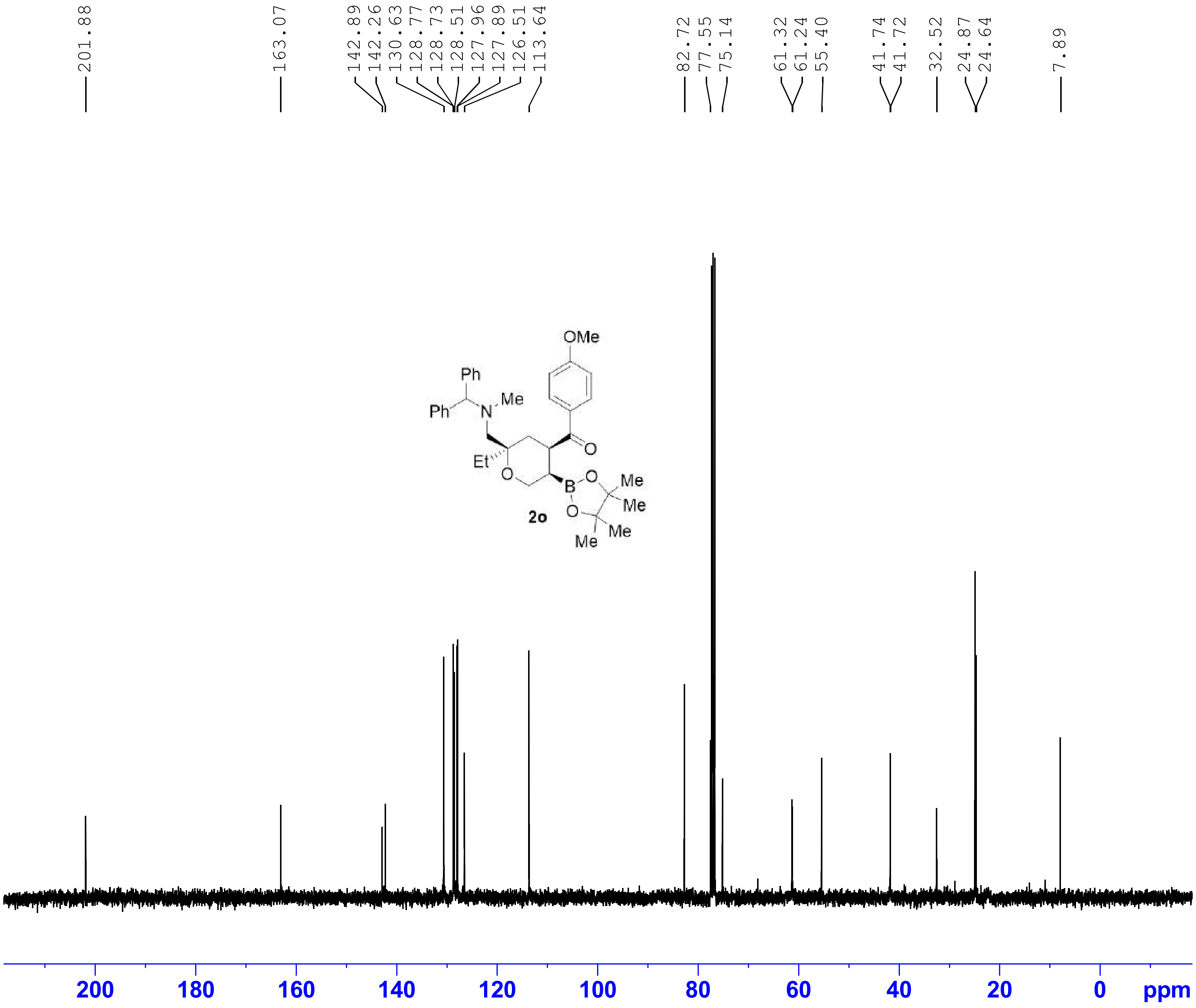

2o

Current Data Parameters
NAME zmh-5-180i-chun
EXPNO 8
PROCNO 1

F2 - Acquisition Parameters
Date_ 20240517
Time 13.41 h
INSTRUM AvanceNeo 400MHz
PROBHD Z163739_0629 (
PULPROG zgpg30
TD 65536
SOLVENT CDCl3
NS 80
DS 4
SWH 23809.523 Hz
FIDRES 0.726609 Hz
AQ 1.3762560 sec
RG 10
DW 21.000 usec
DE 6.50 usec
TE 297.5 K
D1 2.00000000 sec
D11 0.03000000 sec
TD0 1
SFO1 100.6354036 MHz
NUC1 13C
P0 2.67 usec
P1 8.00 usec
PLW1 85.25399780 W
SFO2 400.1816007 MHz
NUC2 1H
CPDPRG[2 waltz65
PCPD2 90.00 usec
PLW2 21.26700020 W
PLW12 0.16802999 W
PLW13 0.08452000 W

F2 - Processing parameters
SI 32768
SF 100.6253476 MHz
WDW EM
SSB 0
LB 1.00 Hz
GB 0
PC 1.40

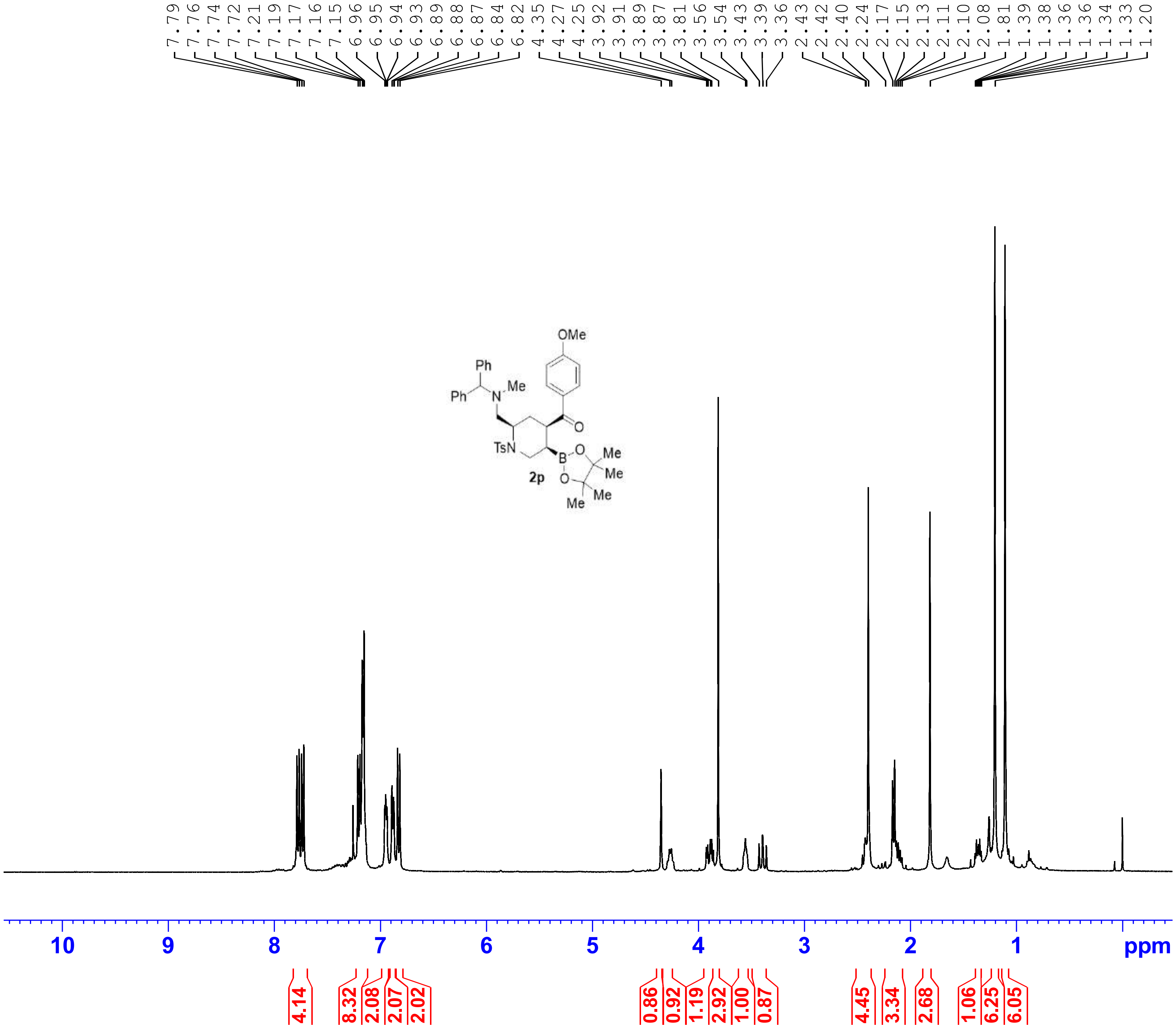

Current Data Parameters	
NAME	zmh-5-180m
EXPNO	1
PROCNO	1
F2 - Acquisition Parameters	
Date_	20240530
Time	14.11
INSTRUM	spect
PROBHD	5 mm PABBO BB/
PULPROG	zg30
TD	65536
SOLVENT	CDCl3
NS	8
DS	2
SWH	8012.820 Hz
FIDRES	0.122266 Hz
AQ	4.0894465 sec
RG	31.55
DW	62.400 usec
DE	6.50 usec
TE	296.3 K
D1	1.00000000 sec
TD0	1
======== CHANNEL f1 ========	
SFO1	400.1324710 MHz
NUC1	1H
P1	14.50 usec
PLW1	11.99499989 W
F2 - Processing parameters	
SI	65536
SF	400.1300109 MHz
WDW	EM
SSB	0
LB	0.30 Hz
GB	0
PC	1.00

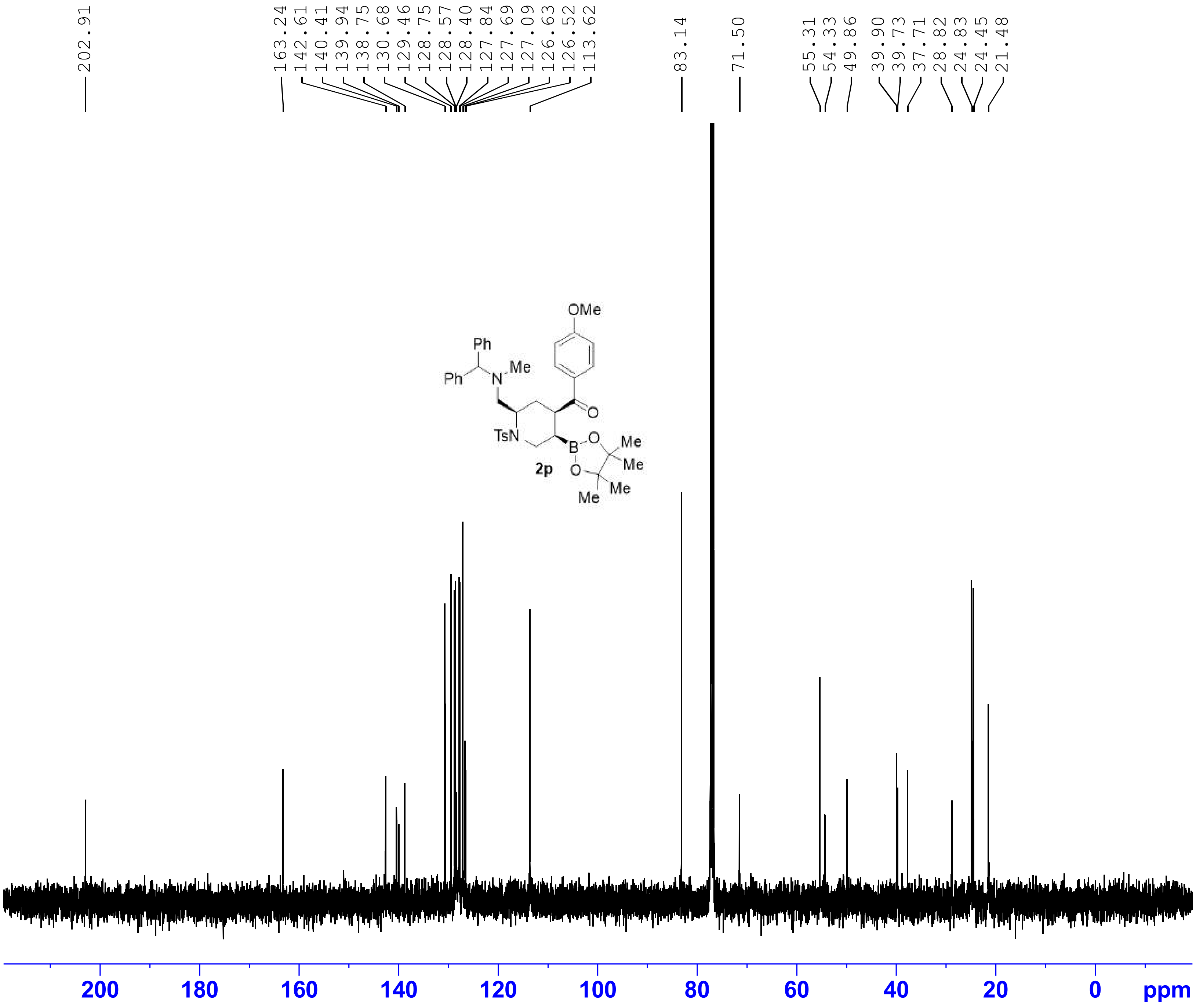


```
Current Data Parameters
NAME             zmh-5-180m
EXPNO                     2
PROCNO                    1

F2 - Acquisition Parameters
Date_              20240530
Time                  14.14
INSTRUM               spect
PROBHD   5 mm PABBO BB/
PULPROG             zgpg30
TD                    65536
SOLVENT               CDCl3
NS                      117
DS                        2
SWH            24038.461 Hz
FIDRES          0.366798 Hz
AQ             1.3631488 sec
RG                   196.92
DW               20.800 usec
DE                 6.50 usec
TE                  296.8 K
D1            2.00000000 sec
D11           0.03000000 sec
TD0                       1

======== CHANNEL f1 ========
SFO1        100.6228298 MHz
NUC1                    13C
P1                 9.70 usec
PLW1         46.98899841 W

======== CHANNEL f2 ========
SFO2        400.1316005 MHz
NUC2                     1H
CPDPRG[2            waltz16
PCPD2             90.00 usec
PLW2         11.99499989 W
PLW12         0.34213999 W
PLW13         0.27713001 W

F2 - Processing parameters
SI                    32768
SF          100.6127746 MHz
WDW                      EM
SSB                       0
LB                  1.00 Hz
GB                        0
PC                     1.40
```

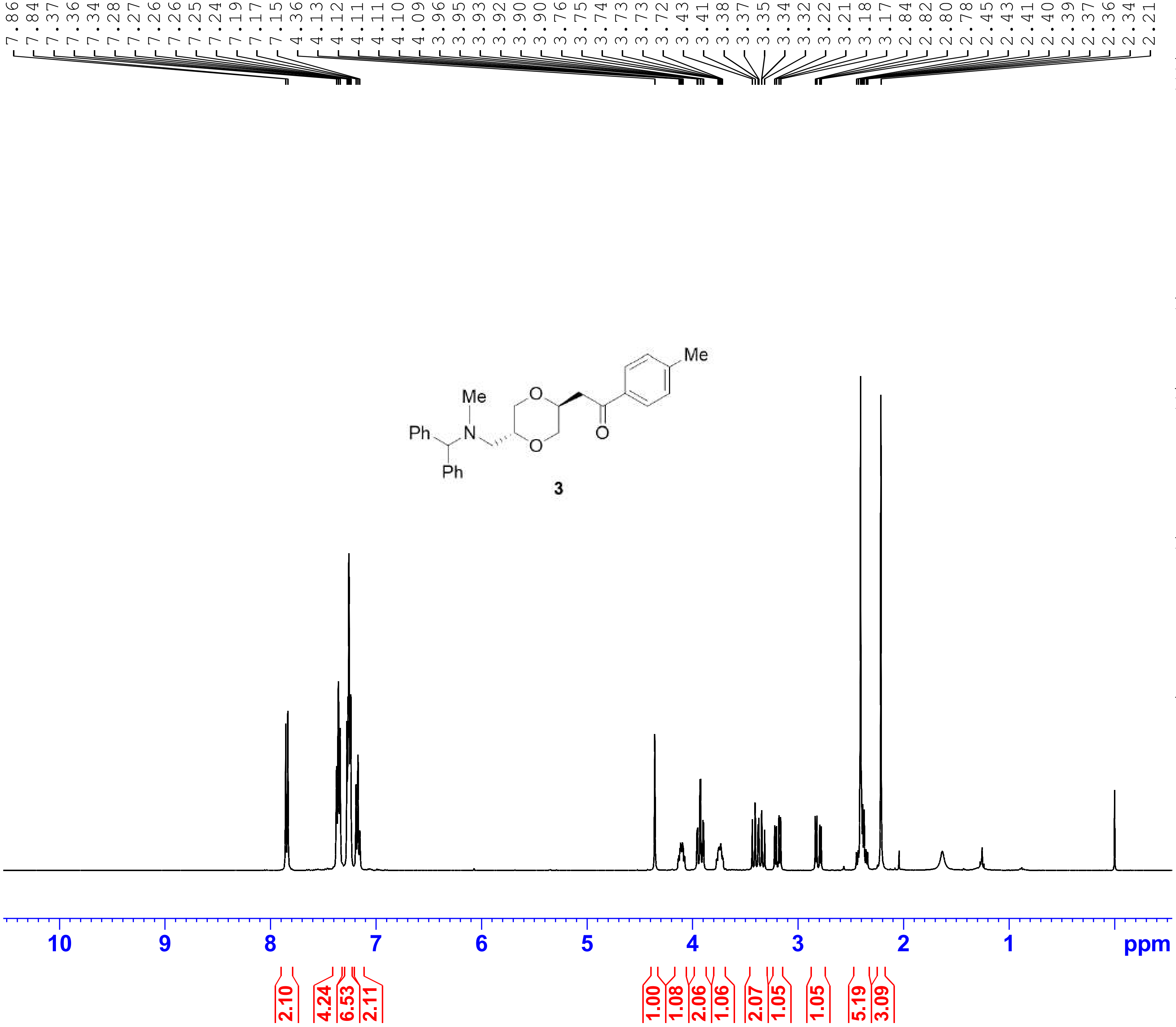

Current Data Parameters
NAME zmh-5-p17
EXPNO 1
PROCNO 1

F2 - Acquisition Parameters
Date_ 20240604
Time 19.09 h
INSTRUM AvanceNeo 400MHz
PROBHD Z163739_0629 (
PULPROG zg30
TD 65536
SOLVENT CDCl3
NS 8
DS 2
SWH 8196.722 Hz
FIDRES 0.250144 Hz
AQ 3.9976959 sec
RG 101
DW 61.000 usec
DE 13.89 usec
TE 296.8 K
D1 1.00000000 sec
TD0 1
SFO1 400.1824711 MHz
NUC1 1H
P0 2.67 usec
P1 8.00 usec
PLW1 21.26700020 W

F2 - Processing parameters
SI 65536
SF 400.1800118 MHz
WDW EM
SSB 0
LB 0.30 Hz
GB 0
PC 1.00

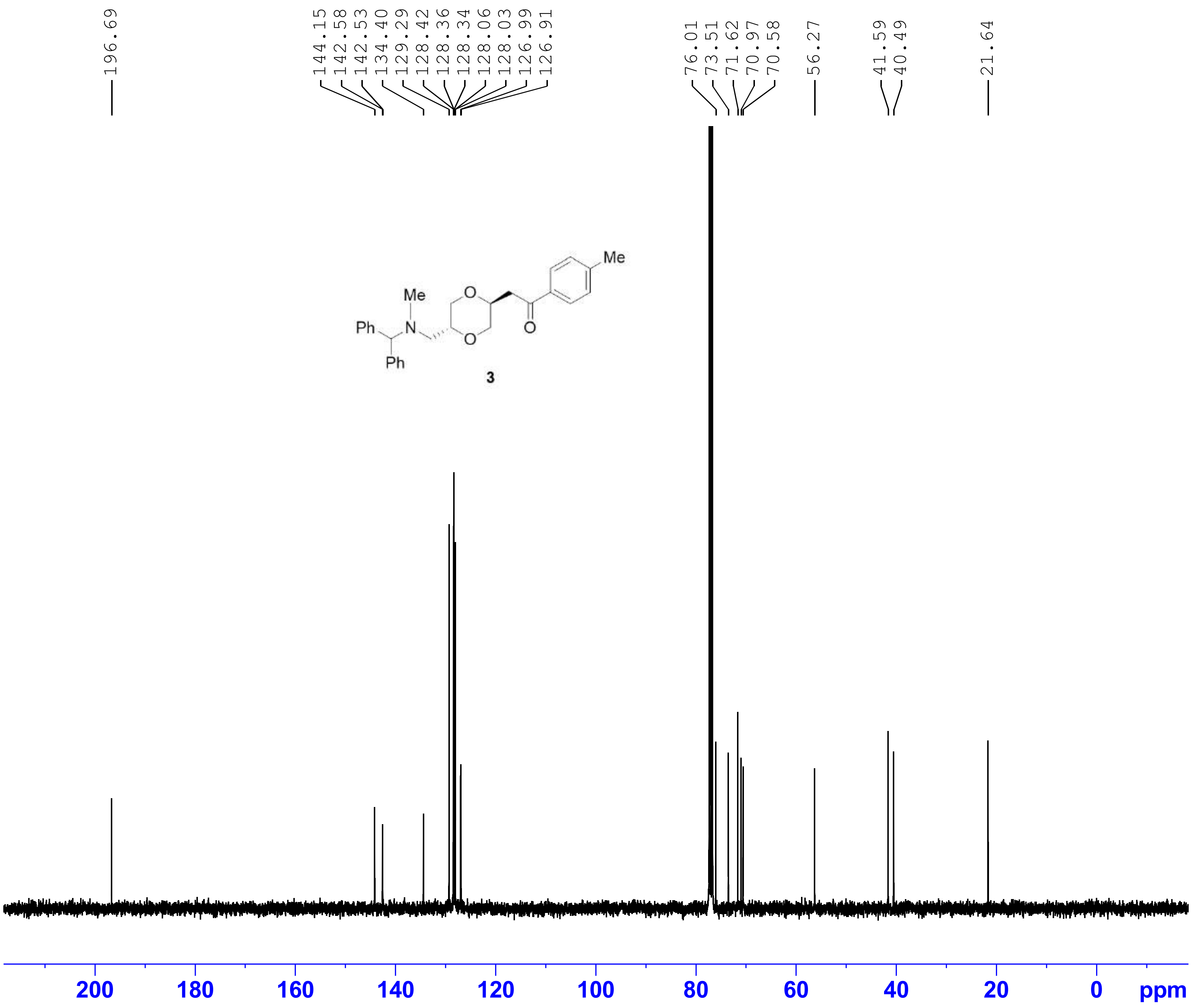

Current Data Parameters
NAME zmh-5-p17
EXPNO 2
PROCNO 1

F2 - Acquisition Parameters
Date_ 20240604
Time 19.33 h
INSTRUM AvanceNeo 400MHz
PROBHD Z163739_0629 (
PULPROG zgpg30
TD 65536
SOLVENT CDCl3
NS 400
DS 4
SWH 23809.523 Hz
FIDRES 0.726609 Hz
AQ 1.3762560 sec
RG 10
DW 21.000 usec
DE 6.50 usec
TE 297.5 K
D1 2.00000000 sec
D11 0.03000000 sec
TD0 1
SFO1 100.6354036 MHz
NUC1 13C
P0 2.67 usec
P1 8.00 usec
PLW1 85.25399780 W
SFO2 400.1816007 MHz
NUC2 1H
CPDPRG[2 waltz65
PCPD2 90.00 usec
PLW2 21.26700020 W
PLW12 0.16802999 W
PLW13 0.08452000 W

F2 - Processing parameters
SI 32768
SF 100.6253455 MHz
WDW EM
SSB 0
LB 1.00 Hz
GB 0
PC 1.40

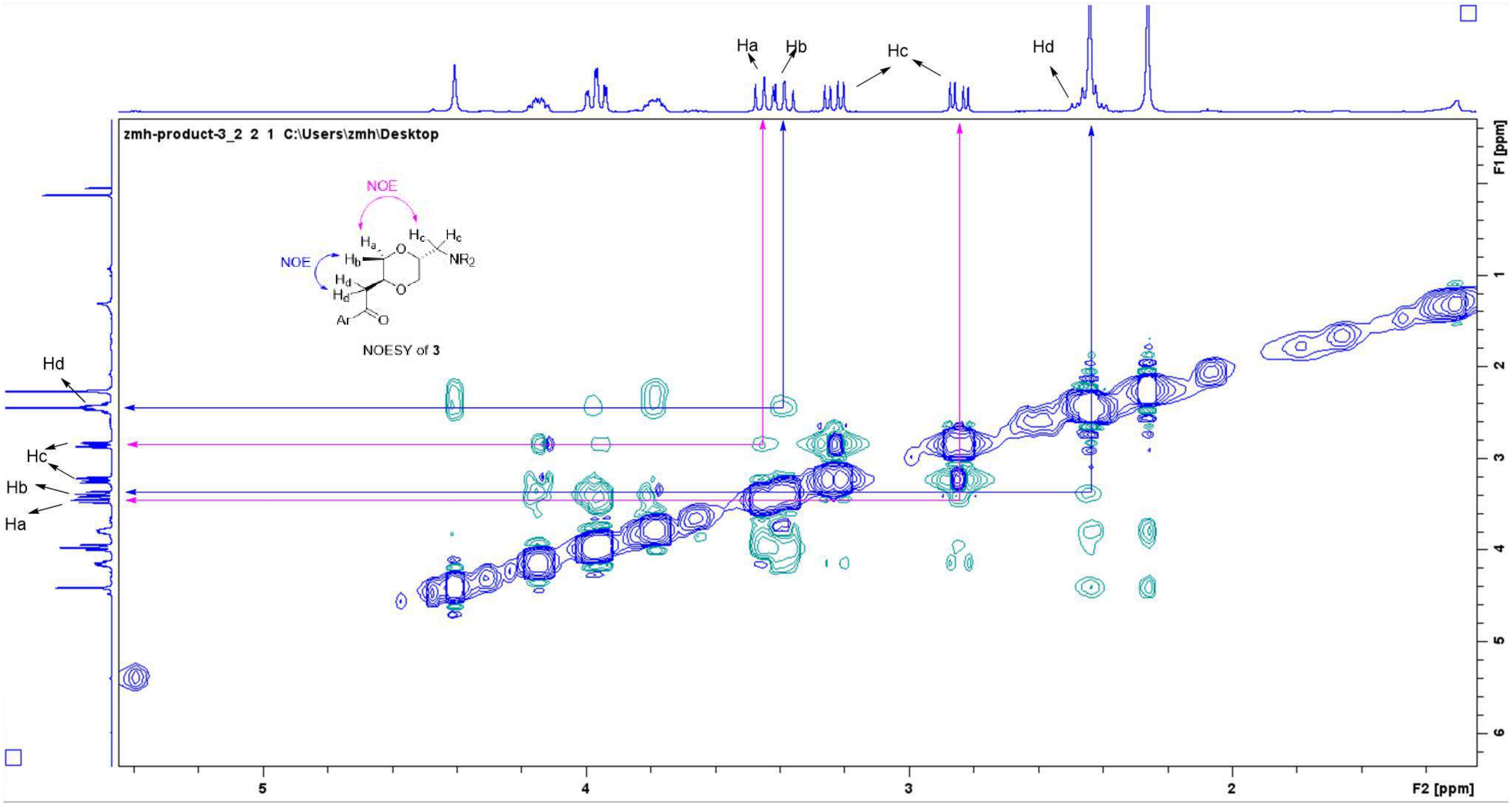

zmh-product-3_2 2 1 C:\Users\zmh\Desktop
NOE
NOE
NOESY of 3
Ha
Hb
Hc
Hd
F1 [ppm]
F2 [ppm]

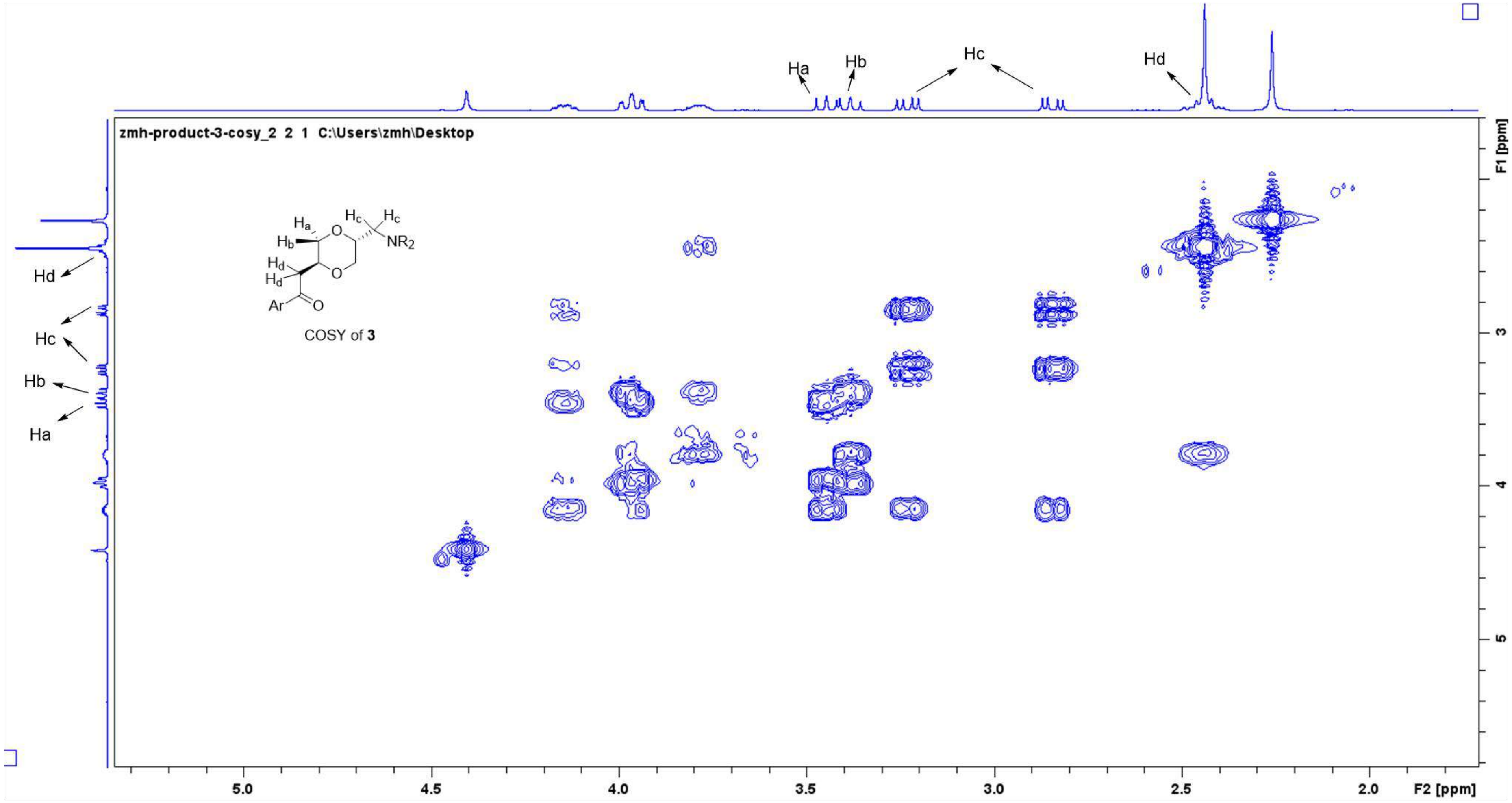

zmh-product-3-cosy_2 2 1 C:\Users\zmh\Desktop
Ha
Hb
Hc
Hd
F1 [ppm]
F2 [ppm]
5.0
4.5
4.0
3.5
3.0
2.5
2.0
3
4
5
Ha
Hb
Hc
Hc
Hd
Hd
Ar
O
NR2

COSY of **3**

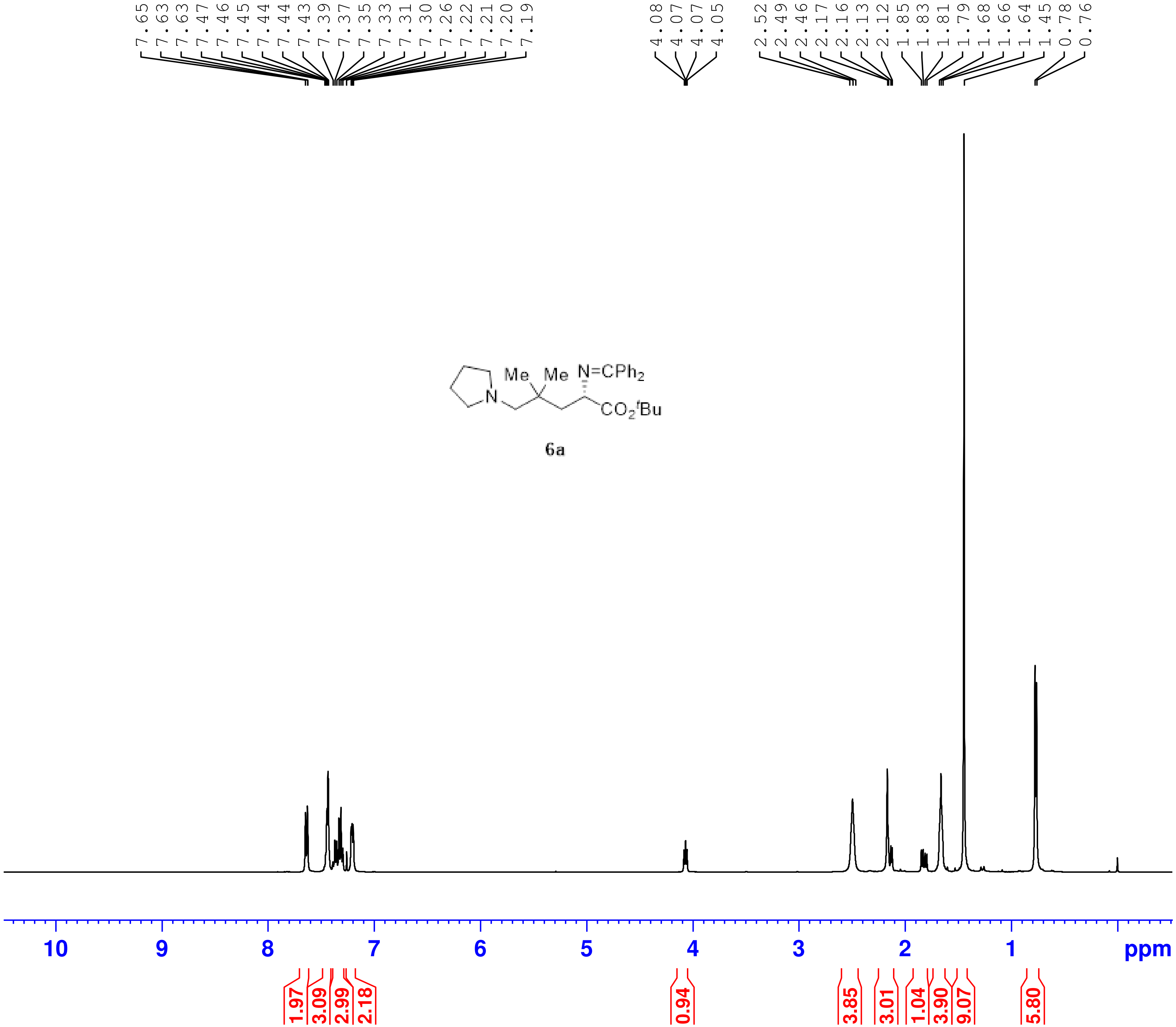

Current Data Parameters
NAME zmh-2-1b-chun-1212
EXPNO 1
PROCNO 1

F2 - Acquisition Parameters
Date_ 20221212
Time 18.44 h
INSTRUM AvanceNeo 400MHz
PROBHD Z163739_0629 (
PULPROG zg30
TD 65536
SOLVENT CDCl3
NS 16
DS 2
SWH 8196.722 Hz
FIDRES 0.250144 Hz
AQ 3.9976959 sec
RG 64
DW 61.000 usec
DE 13.89 usec
TE 293.8 K
D1 1.00000000 sec
TD0 1
SFO1 400.1824711 MHz
NUC1 1H
P0 2.67 usec
P1 8.00 usec
PLW1 21.26700020 W

F2 - Processing parameters
SI 65536
SF 400.1800095 MHz
WDW EM
SSB 0
LB 0.30 Hz
GB 0
PC 1.00

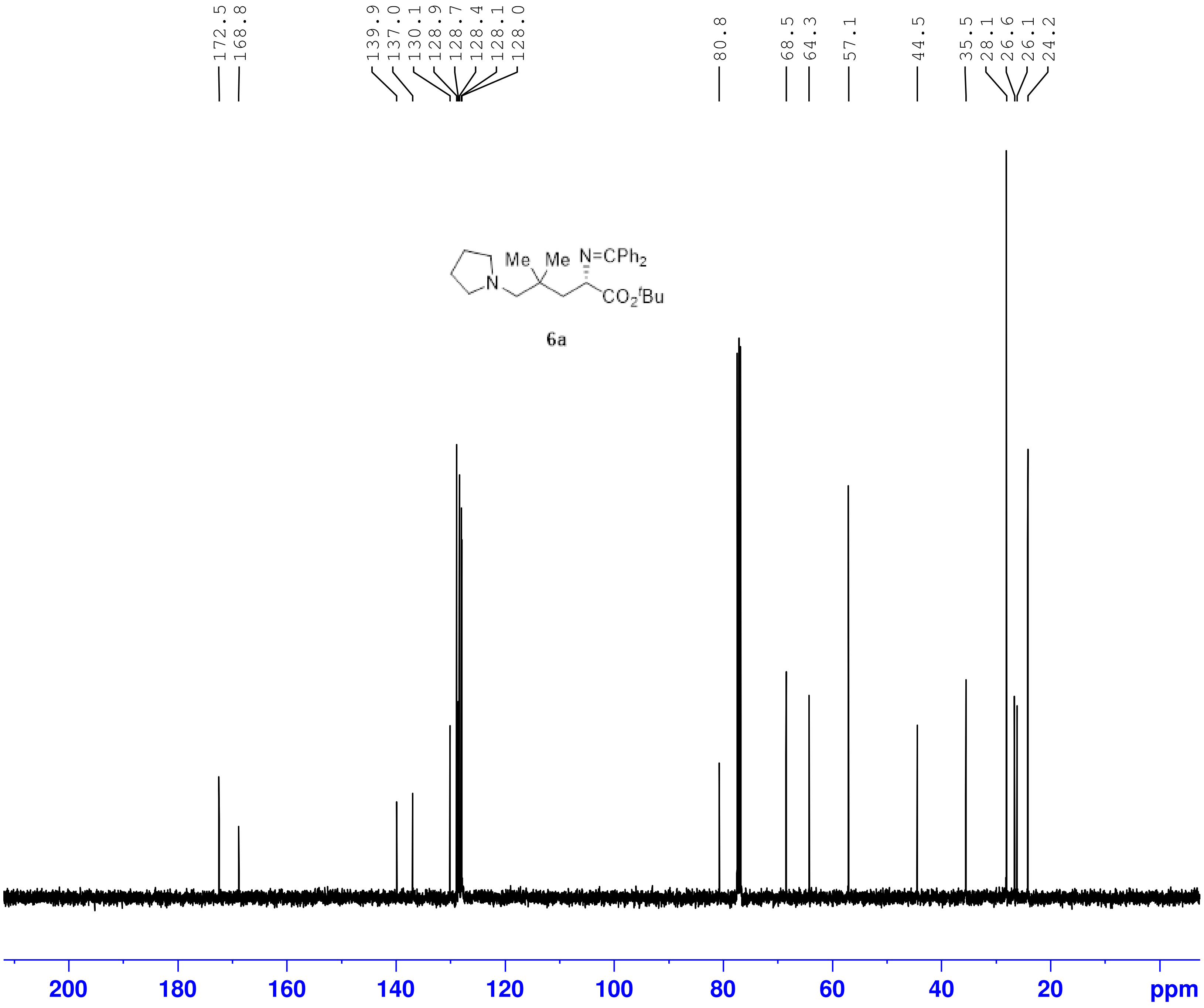

Current Data Parameters
NAME zmh-2-1b-chun-1212
EXPNO 2
PROCNO 1

F2 - Acquisition Parameters
Date_ 20221212
Time 18.52 h
INSTRUM AvanceNeo 400MHz
PROBHD Z163739_0629 (
PULPROG zgpg30
TD 65536
SOLVENT CDCl3
NS 94
DS 4
SWH 23809.523 Hz
FIDRES 0.726609 Hz
AQ 1.3762560 sec
RG 10
DW 21.000 usec
DE 6.50 usec
TE 294.0 K
D1 2.00000000 sec
D11 0.03000000 sec
TD0 1
SFO1 100.6354036 MHz
NUC1 13C
P0 2.67 usec
P1 8.00 usec
PLW1 85.25399780 W
SFO2 400.1816007 MHz
NUC2 1H
CPDPRG[2 waltz65
PCPD2 90.00 usec
PLW2 21.26700020 W
PLW12 0.16802999 W
PLW13 0.08452000 W

F2 - Processing parameters
SI 32768
SF 100.6253323 MHz
WDW EM
SSB 0
LB 1.00 Hz
GB 0
PC 1.40

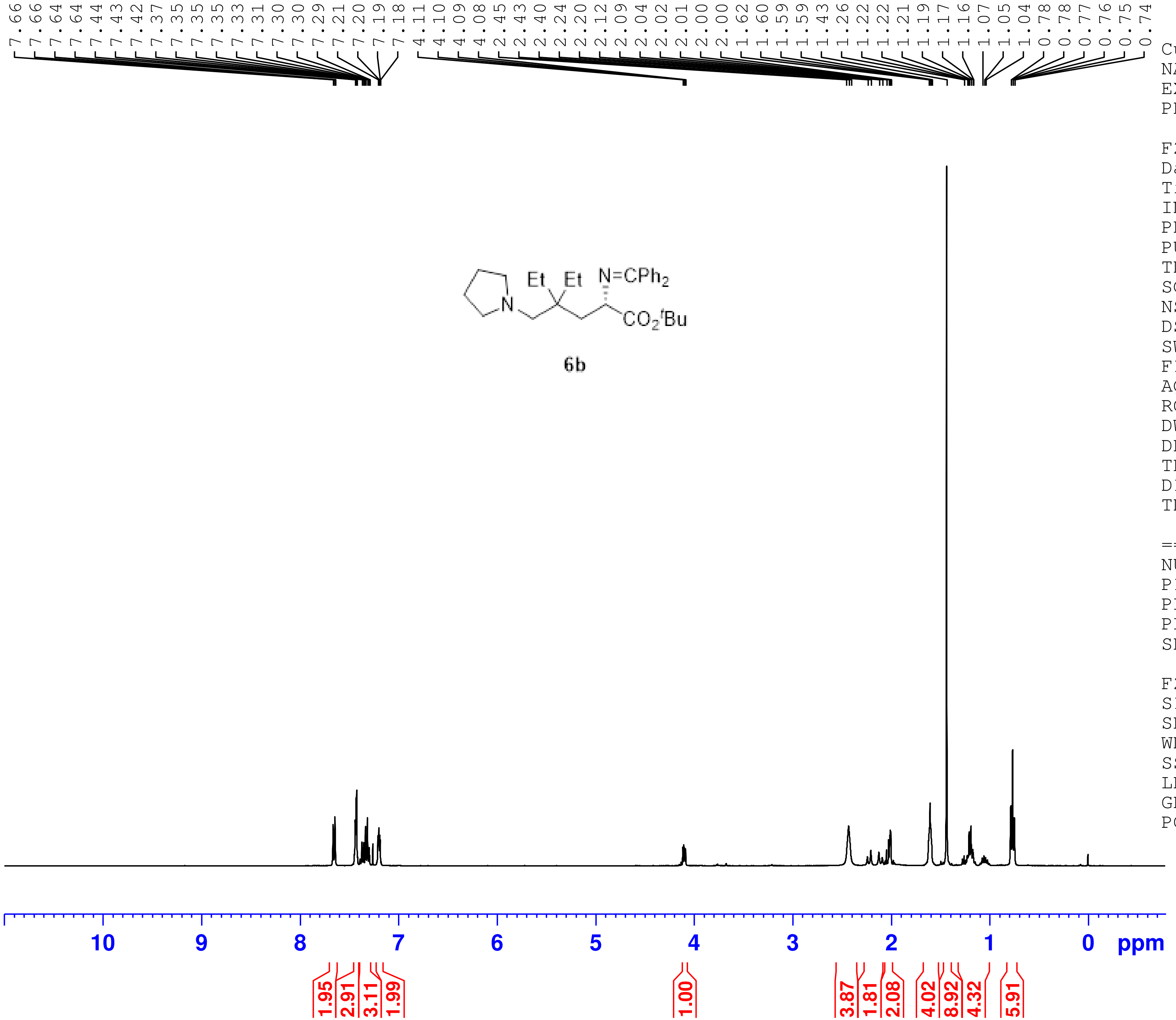

Current Data Parameters
NAME 3b
EXPNO 2
PROCNO 1

F2 - Acquisition Parameters
Date_ 20230116
Time 18.29
INSTRUM spect
PROBHD 5 mm DUL 13C-1
PULPROG zg30
TD 65536
SOLVENT CDCl3
NS 16
DS 2
SWH 8223.685 Hz
FIDRES 0.125483 Hz
AQ 3.9845889 sec
RG 144
DW 60.800 usec
DE 6.00 usec
TE 292.7 K
D1 1.00000000 sec
TD0 1

======== CHANNEL f1 ========
NUC1 1H
P1 15.80 usec
PL1 -1.00 dB
PL1W 12.17476940 W
SFO1 400.1324710 MHz

F2 - Processing parameters
SI 32768
SF 400.1300105 MHz
WDW EM
SSB 0
LB 0.30 Hz
GB 0
PC 1.00

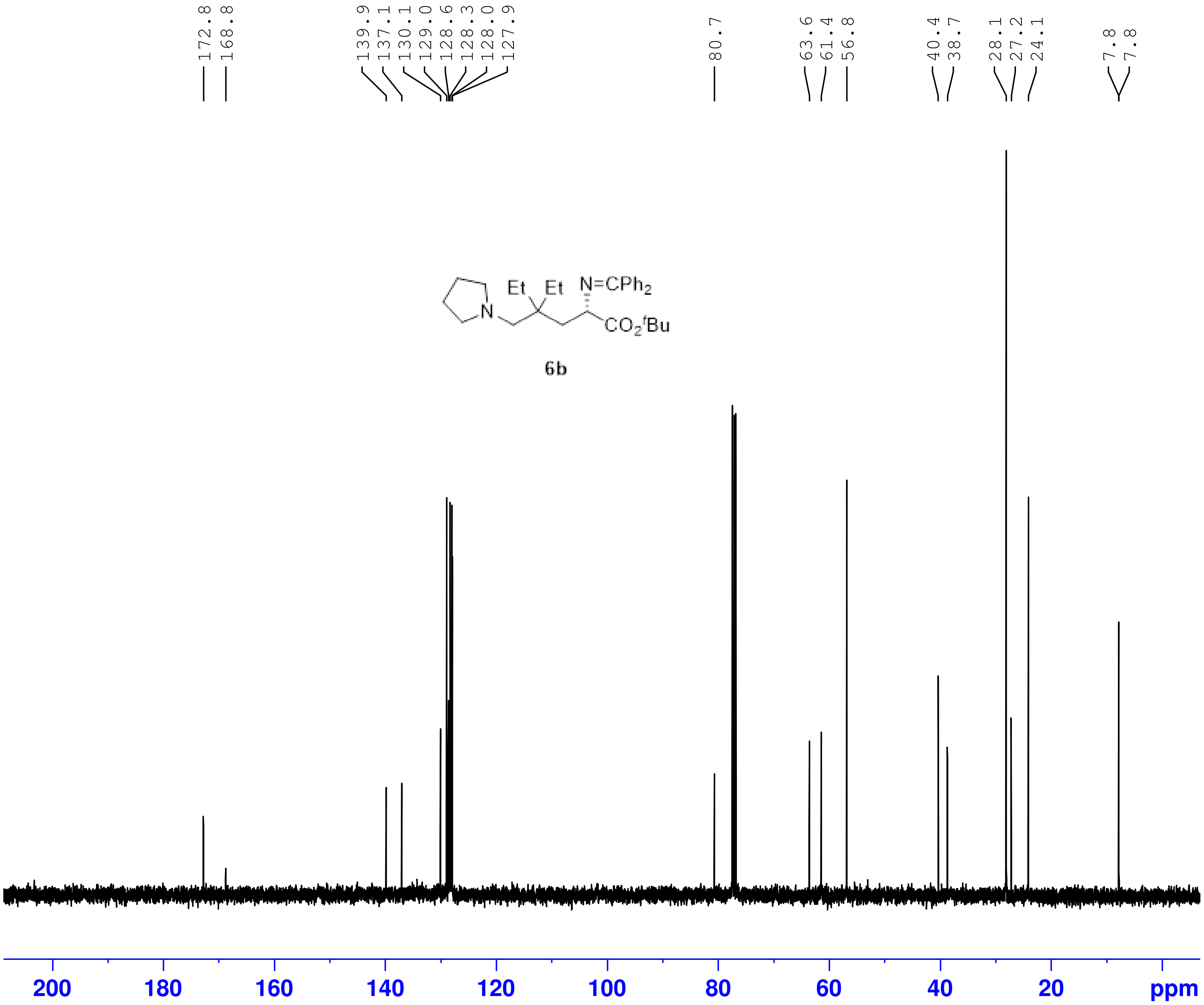


```
Current Data Parameters
NAME                 3b
EXPNO                 1
PROCNO                1

F2 - Acquisition Parameters
Date_          20230116
Time              18.32
INSTRUM           spect
PROBHD   5 mm DUL 13C-1
PULPROG         zgpg30
TD                65536
SOLVENT           CDCl3
NS                  340
DS                    1
SWH           24038.461 Hz
FIDRES         0.366798 Hz
AQ            1.3631488 sec
RG                 2050
DW               20.800 usec
DE                 6.00 usec
TE                292.7 K
D1           2.00000000 sec
D11          0.03000000 sec
TD0                   1

======== CHANNEL f1 ========
NUC1                13C
P1                40.00 usec
PL1               -3.00 dB
PL1W        60.64365387 W
SFO1        100.6228298 MHz

======== CHANNEL f2 ========
CPDPRG[2        waltz16
NUC2                 1H
PCPD2             80.00 usec
PL2               -1.00 dB
PL12              14.39 dB
PL13              18.00 dB
PL2W        12.17476940 W
PL12W        0.35193357 W
PL13W        0.15327126 W
SFO2        400.1316005 MHz

F2 - Processing parameters
SI                32768
SF          100.6127588 MHz
WDW                  EM
SSB                   0
LB                 1.00 Hz
GB                    0
PC                 1.40
```

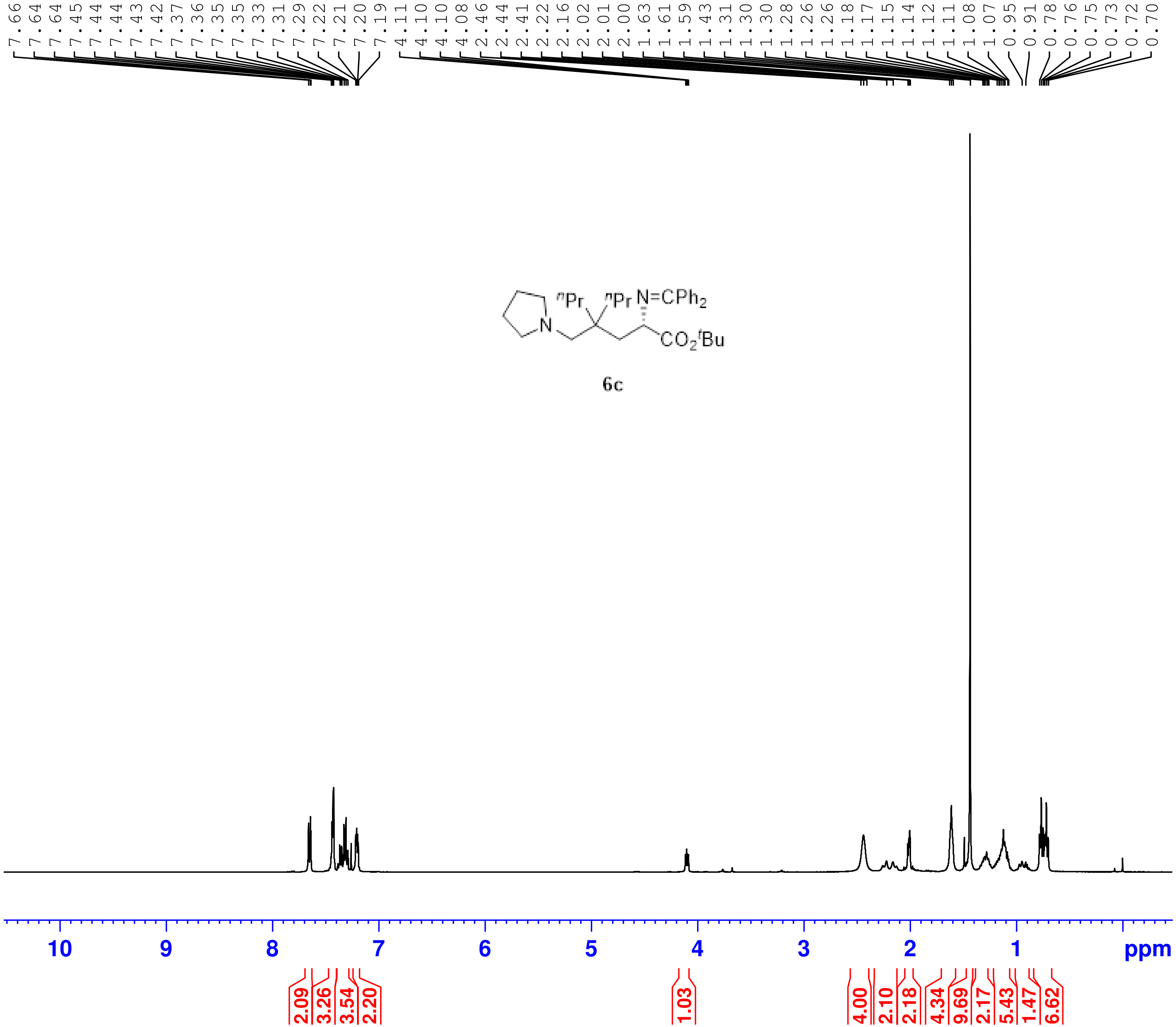

Current Data Parameters
NAME zmh-2-98b-chun
EXPNO 1
PROCNO 1

F2 - Acquisition Parameters
Date_ 20230117
Time 18.53
INSTRUM spect
PROBHD 5 mm PABBO BB/
PULPROG zg30
TD 65536
SOLVENT CDCl3
NS 16
DS 2
SWH 8012.820 Hz
FIDRES 0.122266 Hz
AQ 4.0894465 sec
RG 27.78
DW 62.400 usec
DE 6.50 usec
TE 293.0 K
D1 1.00000000 sec
TD0 1

======== CHANNEL f1 ========
SFO1 400.1324710 MHz
NUC1 1H
P1 14.50 usec
PLW1 11.99499989 W

F2 - Processing parameters
SI 65536
SF 400.1300098 MHz
WDW EM
SSB 0
LB 0.30 Hz
GB 0
PC 1.00

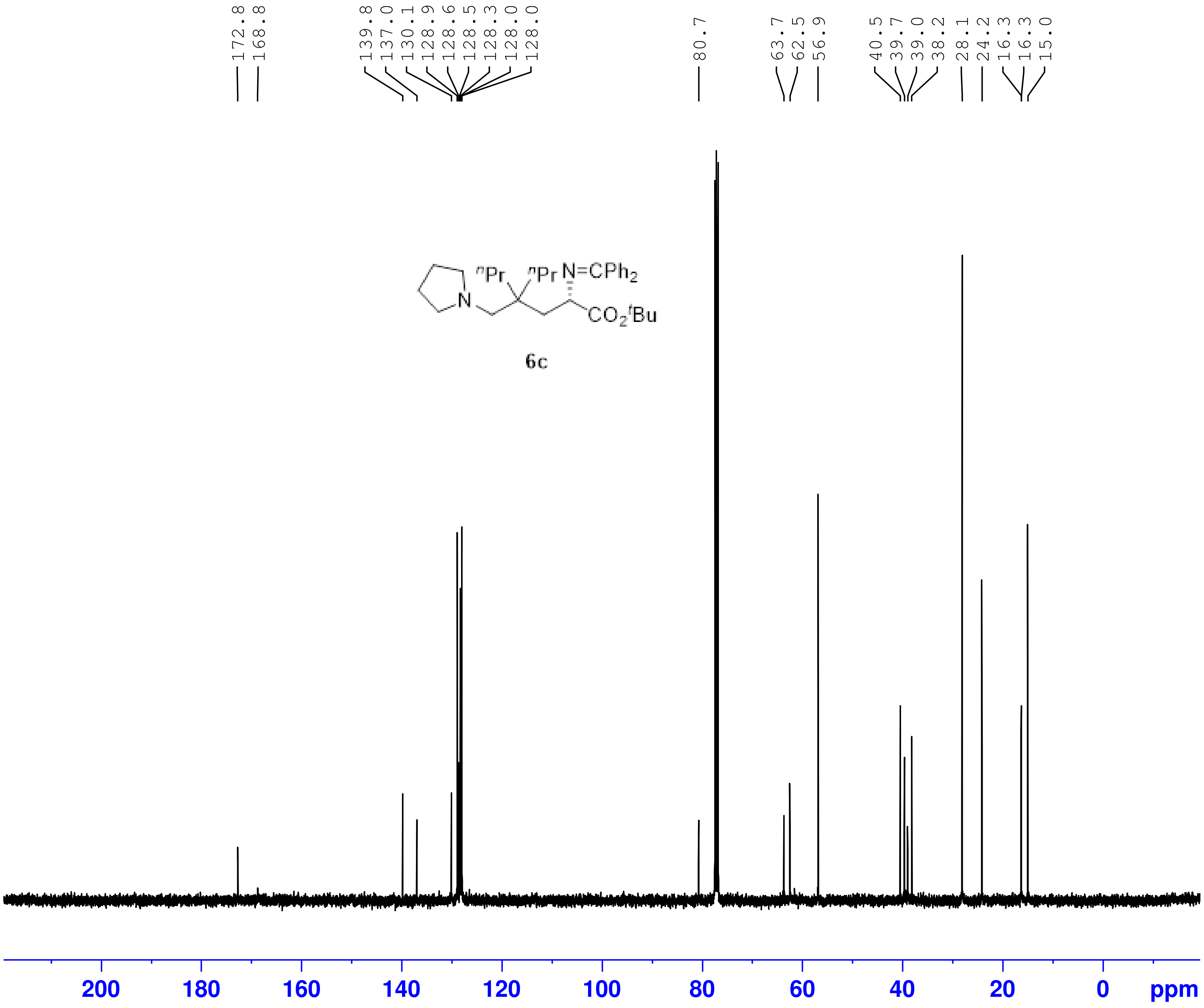

6c

Current Data Parameters
NAME zmh-2-98b-chun
EXPNO 2
PROCNO 1

F2 - Acquisition Parameters
Date_ 20230117
Time 18.55
INSTRUM spect
PROBHD 5 mm PABBO BB/
PULPROG zgpg30
TD 65536
SOLVENT CDCl3
NS 252
DS 2
SWH 24038.461 Hz
FIDRES 0.366798 Hz
AQ 1.3631488 sec
RG 196.92
DW 20.800 usec
DE 6.50 usec
TE 293.2 K
D1 2.00000000 sec
D11 0.03000000 sec
TD0 1

======== CHANNEL f1 ========
SFO1 100.6228298 MHz
NUC1 13C
P1 9.70 usec
PLW1 46.98899841 W

======== CHANNEL f2 ========
SFO2 400.1316005 MHz
NUC2 1H
CPDPRG[2 waltz16
PCPD2 90.00 usec
PLW2 11.99499989 W
PLW12 0.34213999 W
PLW13 0.27713001 W

F2 - Processing parameters
SI 32768
SF 100.6127587 MHz
WDW EM
SSB 0
LB 1.00 Hz
GB 0
PC 1.40

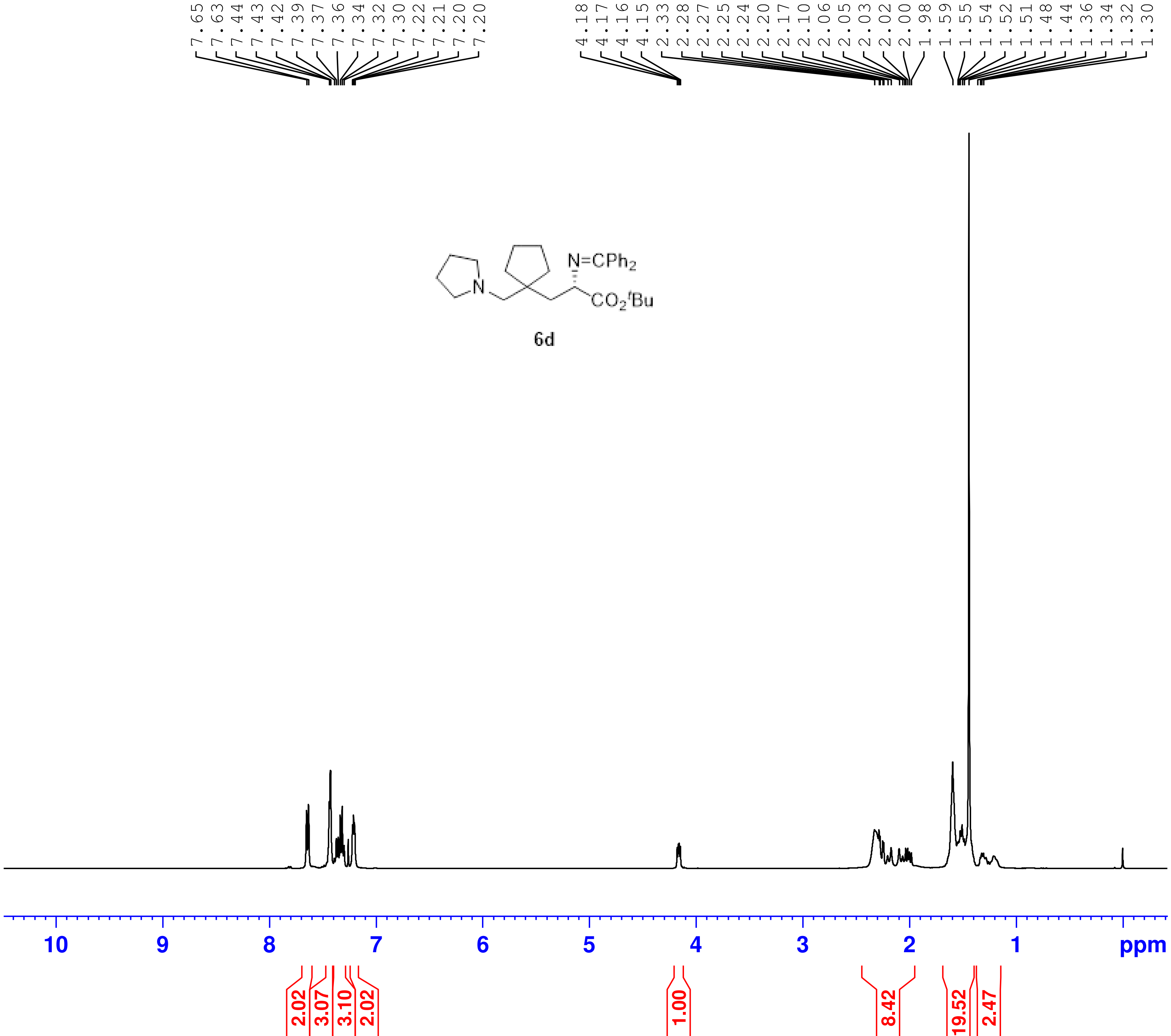


```
Current Data Parameters
NAME        zmh-2-4a-chun-1213
EXPNO                        1
PROCNO                       1

F2 - Acquisition Parameters
Date_             20221213
Time                 22.40 h
INSTRUM    AvanceNeo 400MHz
PROBHD     Z163739_0629 (
PULPROG               zg30
TD                   65536
SOLVENT              CDCl3
NS                       8
DS                       2
SWH               8196.722 Hz
FIDRES            0.250144 Hz
AQ               3.9976959 sec
RG                     101
DW                  61.000 usec
DE                   13.89 usec
TE                   293.8 K
D1              1.00000000 sec
TD0                      1
SFO1           400.1824711 MHz
NUC1                    1H
P0                    2.67 usec
P1                    8.00 usec
PLW1           21.26700020 W

F2 - Processing parameters
SI                   65536
SF             400.1800092 MHz
WDW                     EM
SSB                      0
LB                    0.30 Hz
GB                       0
PC                    1.00
```

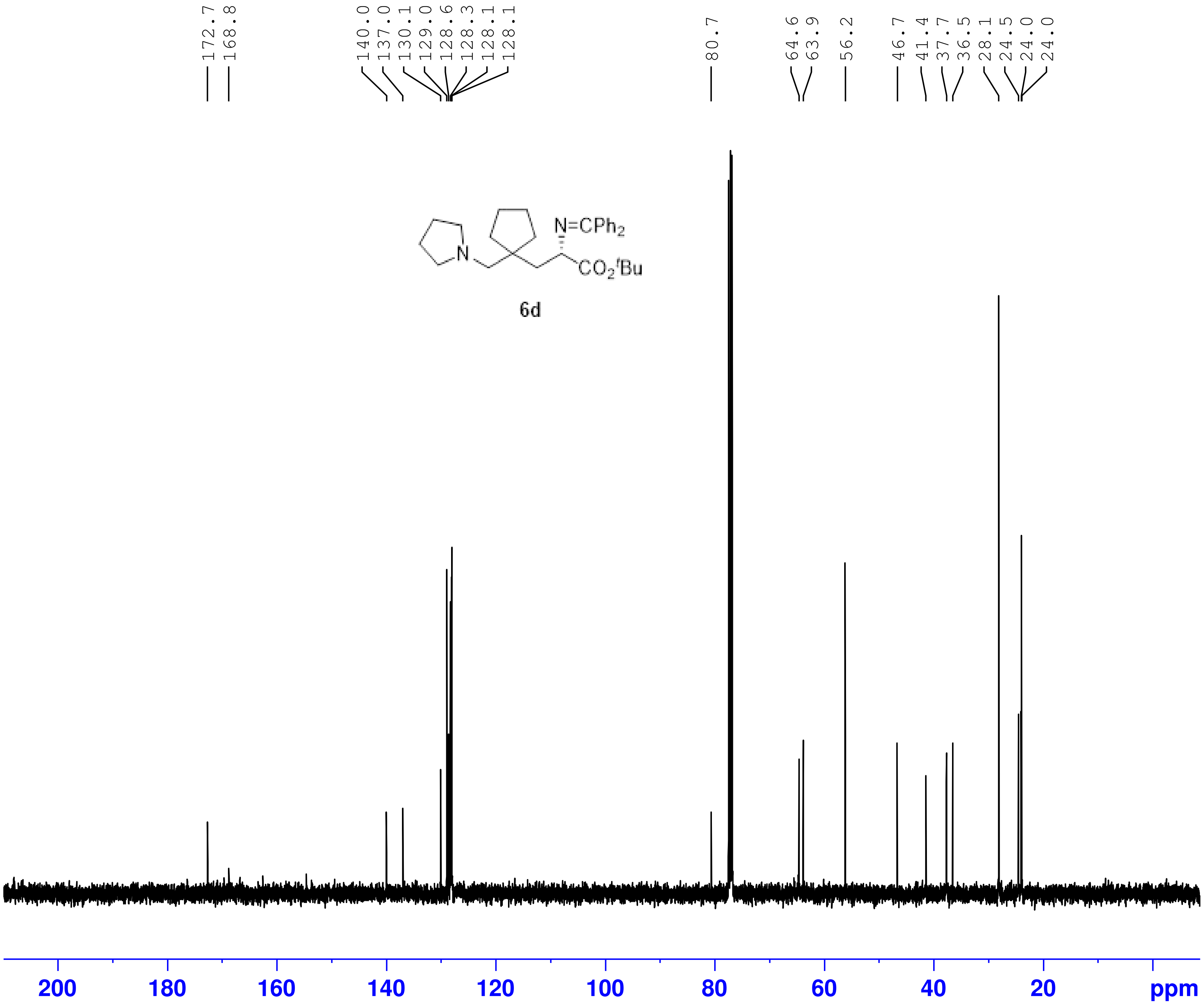

Current Data Parameters
NAME zmh-2-4a-chun-1213
EXPNO 2
PROCNO 1

F2 - Acquisition Parameters
Date_ 20221213
Time 22.47 h
INSTRUM AvanceNeo 400MHz
PROBHD Z163739_0629 (
PULPROG zgpg30
TD 65536
SOLVENT CDCl3
NS 98
DS 4
SWH 23809.523 Hz
FIDRES 0.726609 Hz
AQ 1.3762560 sec
RG 10
DW 21.000 usec
DE 6.50 usec
TE 294.0 K
D1 2.00000000 sec
D11 0.03000000 sec
TD0 1
SFO1 100.6354036 MHz
NUC1 13C
P0 2.67 usec
P1 8.00 usec
PLW1 85.25399780 W
SFO2 400.1816007 MHz
NUC2 1H
CPDPRG[2 waltz65
PCPD2 90.00 usec
PLW2 21.26700020 W
PLW12 0.16802999 W
PLW13 0.08452000 W

F2 - Processing parameters
SI 32768
SF 100.6253308 MHz
WDW EM
SSB 0
LB 1.00 Hz
GB 0
PC 1.40

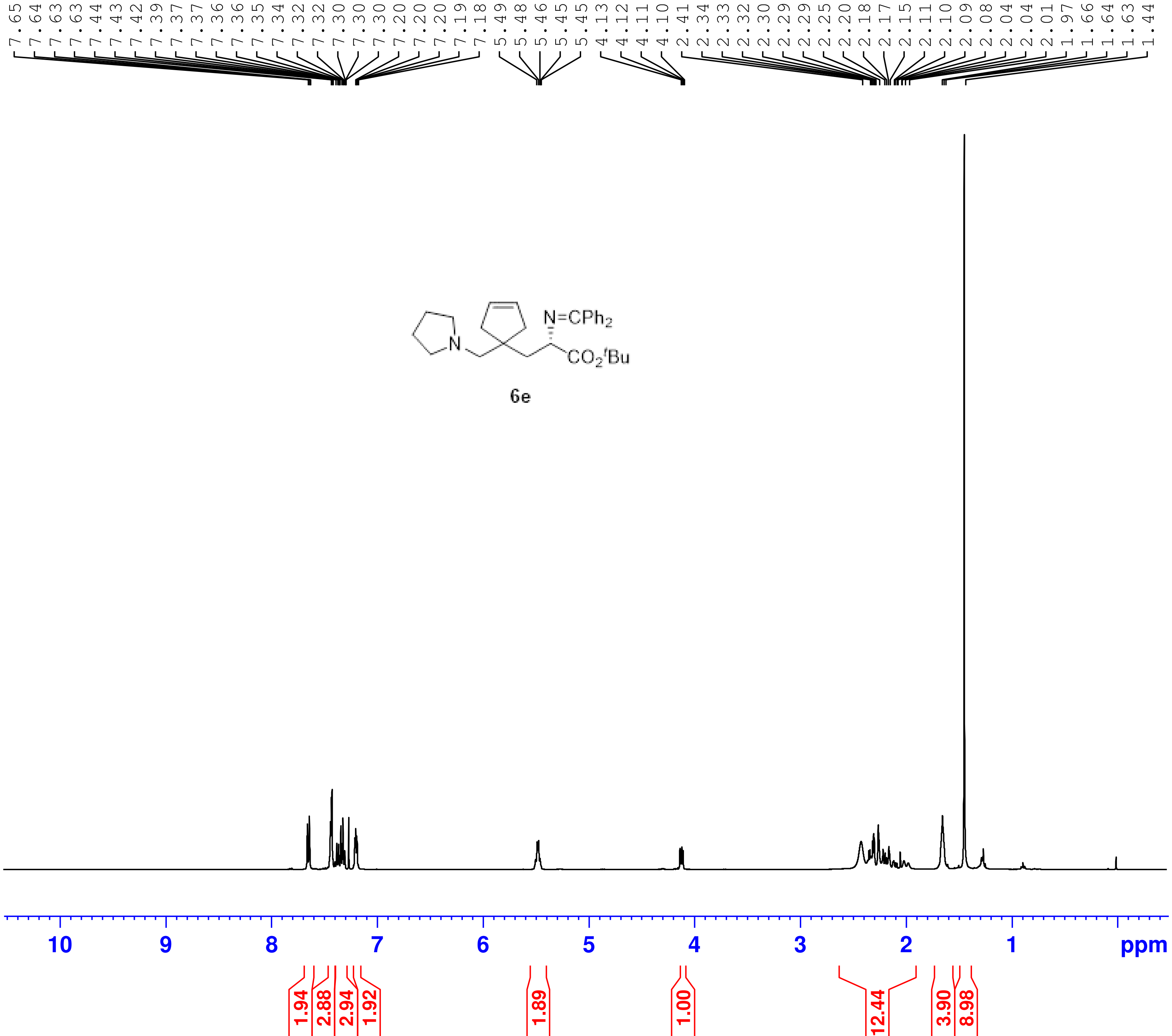

Current Data Parameters
NAME 3e
EXPNO 1
PROCNO 1

F2 - Acquisition Parameters
Date_ 20221216
Time 18.59
INSTRUM spect
PROBHD 5 mm PABBO BB/
PULPROG zg30
TD 65536
SOLVENT CDCl3
NS 8
DS 2
SWH 8012.820 Hz
FIDRES 0.122266 Hz
AQ 4.0894465 sec
RG 27.78
DW 62.400 usec
DE 6.50 usec
TE 294.5 K
D1 1.00000000 sec
TD0 1

======== CHANNEL f1 ========
SFO1 400.1324710 MHz
NUC1 1H
P1 14.50 usec
PLW1 11.99499989 W

F2 - Processing parameters
SI 65536
SF 400.1300098 MHz
WDW EM
SSB 0
LB 0.30 Hz
GB 0
PC 1.00

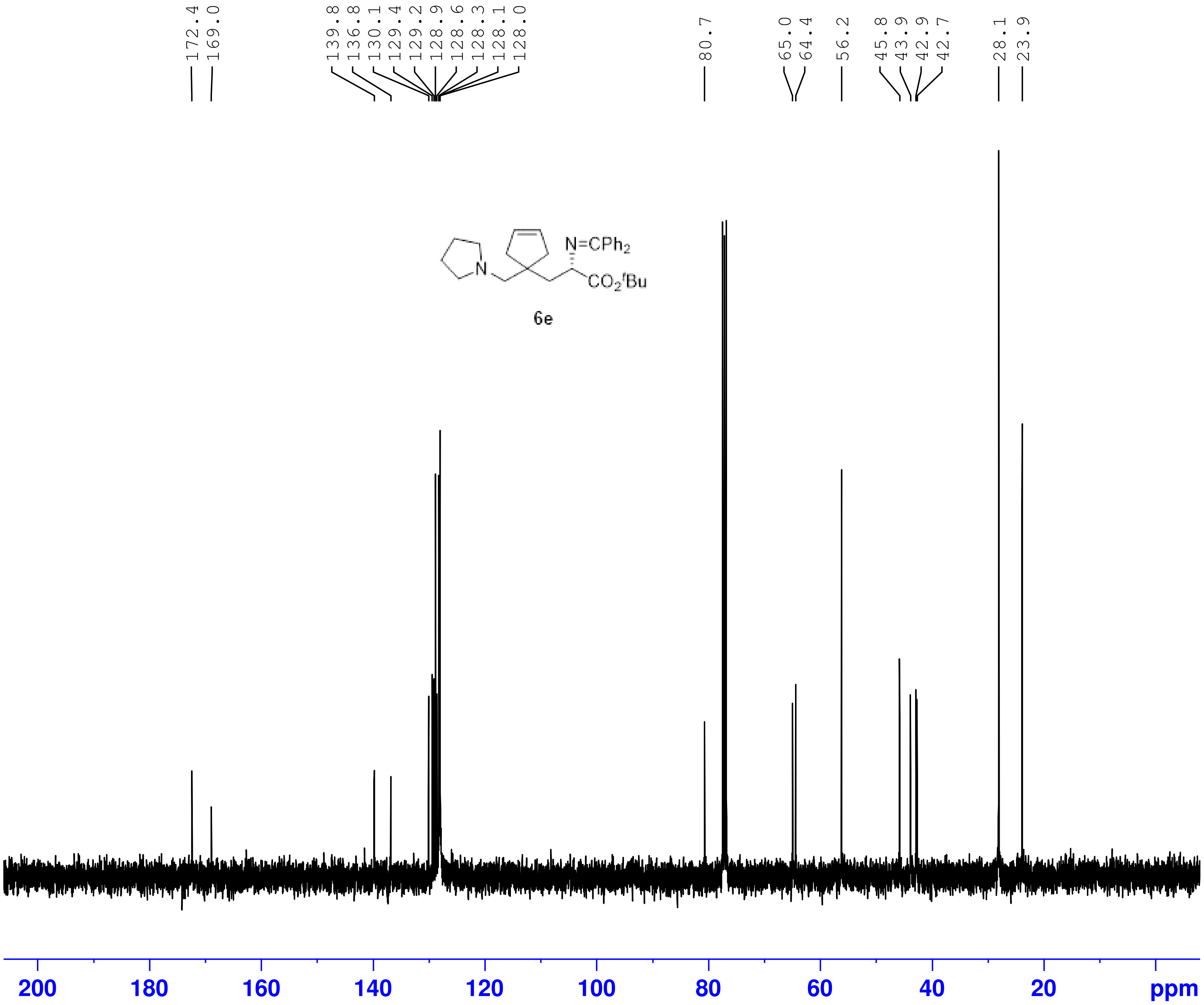

Current Data Parameters
NAME zmh-2-1c-chun-
EXPNO 2
PROCNO 1

F2 - Acquisition Parameters
Date_ 20221130
Time 18.34
INSTRUM spect
PROBHD 5 mm PABBO BB/
PULPROG zgpg30
TD 65536
SOLVENT CDCl3
NS 35
DS 2
SWH 24038.461 Hz
FIDRES 0.366798 Hz
AQ 1.3631488 sec
RG 196.92
DW 20.800 usec
DE 6.50 usec
TE 296.2 K
D1 2.00000000 sec
D11 0.03000000 sec
TD0 1

======== CHANNEL f1 ========
SFO1 100.6228298 MHz
NUC1 13C
P1 9.70 usec
PLW1 46.98899841 W

======== CHANNEL f2 ========
SFO2 400.1316005 MHz
NUC2 1H
CPDPRG[2 waltz16
PCPD2 90.00 usec
PLW2 11.99499989 W
PLW12 0.34213999 W
PLW13 0.27713001 W

F2 - Processing parameters
SI 32768
SF 100.6127638 MHz
WDW EM
SSB 0
LB 1.00 Hz
GB 0
PC 1.40

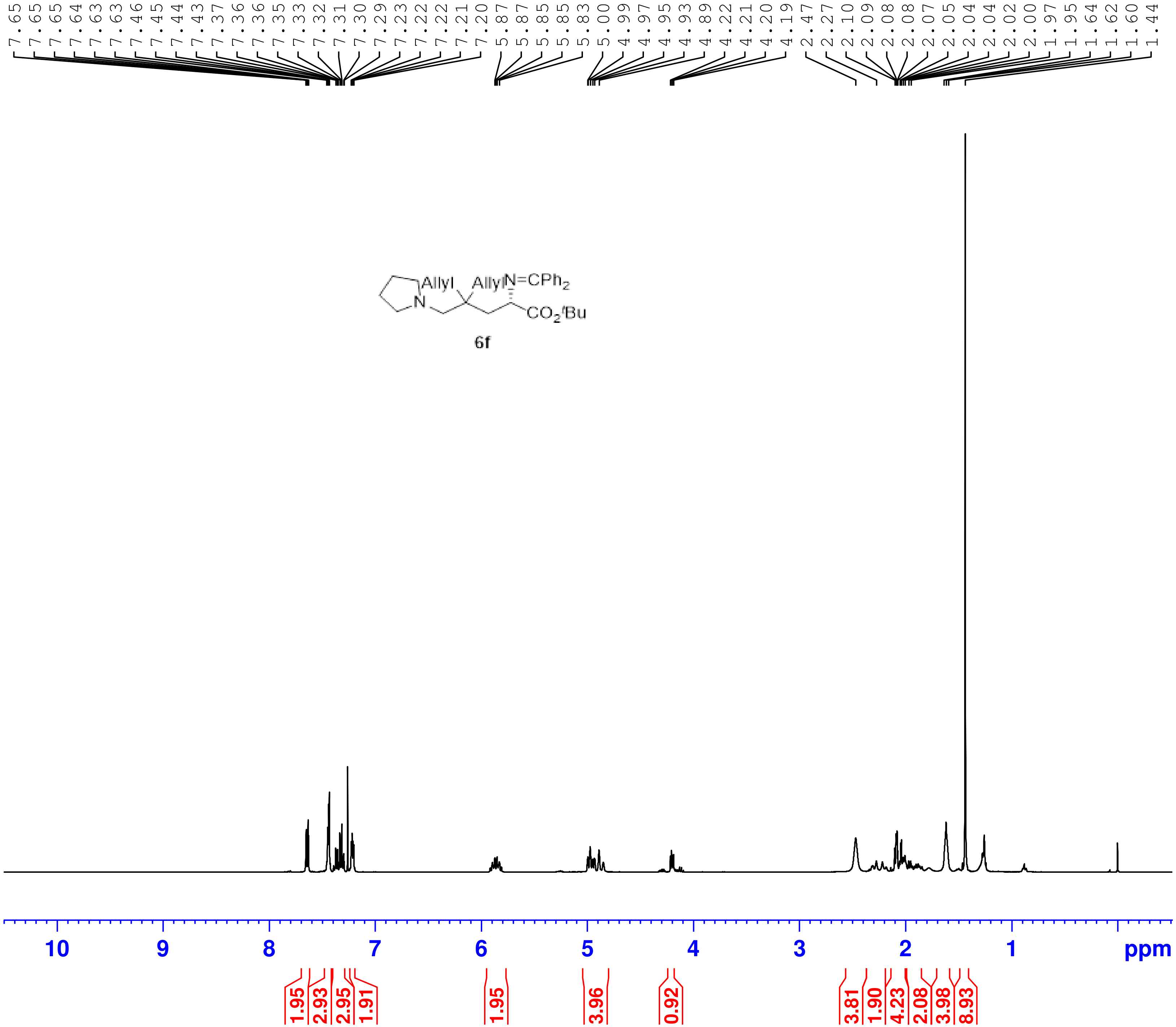

Current Data Parameters
NAME 3f
EXPNO 1
PROCNO 1

F2 - Acquisition Parameters
Date_ 20221216
Time 9.11
INSTRUM spect
PROBHD 5 mm DUL 13C-1
PULPROG zg30
TD 65536
SOLVENT CDCl3
NS 16
DS 2
SWH 8223.685 Hz
FIDRES 0.125483 Hz
AQ 3.9845889 sec
RG 203
DW 60.800 usec
DE 6.00 usec
TE 293.9 K
D1 1.00000000 sec
TD0 1

======== CHANNEL f1 ========
NUC1 1H
P1 15.80 usec
PL1 -1.00 dB
PL1W 12.17476940 W
SFO1 400.1324710 MHz

F2 - Processing parameters
SI 32768
SF 400.1300097 MHz
WDW EM
SSB 0
LB 0.30 Hz
GB 0
PC 1.00

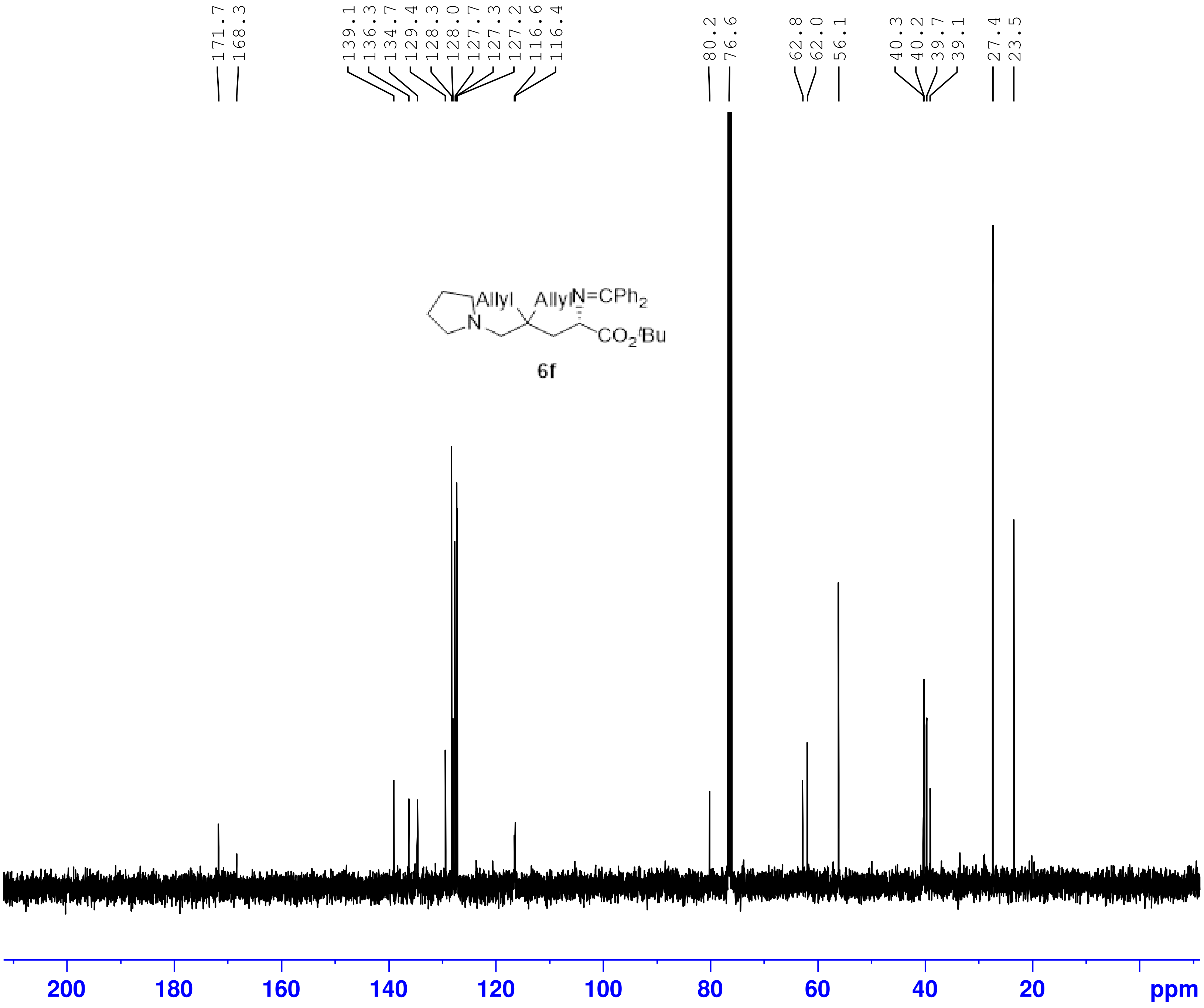

Current Data Parameters
NAME zmh-2-4b-chun-1216-C
EXPNO 1
PROCNO 1

F2 - Acquisition Parameters
Date_ 20221216
Time 9.14
INSTRUM spect
PROBHD 5 mm DUL 13C-1
PULPROG zgpg30
TD 65536
SOLVENT CDCl3
NS 260
DS 1
SWH 24038.461 Hz
FIDRES 0.366798 Hz
AQ 1.3631488 sec
RG 2050
DW 20.800 usec
DE 6.00 usec
TE 293.9 K
D1 2.00000000 sec
D11 0.03000000 sec
TD0 1

======== CHANNEL f1 ========
NUC1 13C
P1 40.00 usec
PL1 -3.00 dB
PL1W 60.64365387 W
SFO1 100.6228298 MHz

======== CHANNEL f2 ========
CPDPRG[2 waltz16
NUC2 1H
PCPD2 80.00 usec
PL2 -1.00 dB
PL12 14.39 dB
PL13 18.00 dB
PL2W 12.17476940 W
PL12W 0.35193357 W
PL13W 0.15327126 W
SFO2 400.1316005 MHz

F2 - Processing parameters
SI 32768
SF 100.6128330 MHz
WDW EM
SSB 0
LB 1.00 Hz
GB 0
PC 1.40

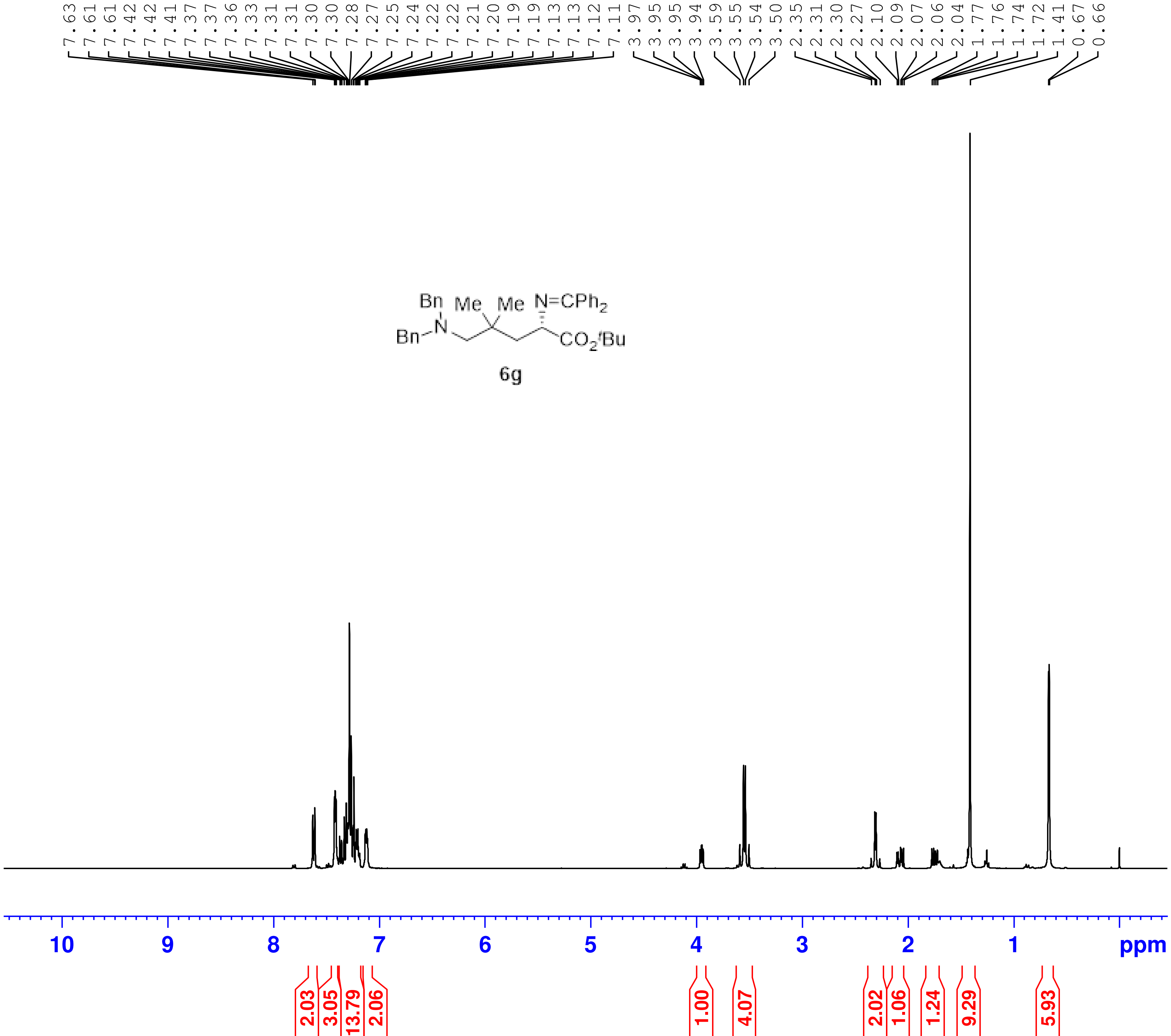

Current Data Parameters
NAME zmh-2-21g-chun-1216
EXPNO 1
PROCNO 1

F2 - Acquisition Parameters
Date_ 20221216
Time 9.41 h
INSTRUM AvanceNeo 400MHz
PROBHD Z163739_0629 (
PULPROG zg30
TD 65536
SOLVENT CDCl3
NS 8
DS 2
SWH 8196.722 Hz
FIDRES 0.250144 Hz
AQ 3.9976959 sec
RG 101
DW 61.000 usec
DE 13.89 usec
TE 294.7 K
D1 1.00000000 sec
TD0 1
SFO1 400.1824711 MHz
NUC1 1H
P0 2.67 usec
P1 8.00 usec
PLW1 21.26700020 W

F2 - Processing parameters
SI 65536
SF 400.1800174 MHz
WDW EM
SSB 0
LB 0.30 Hz
GB 0
PC 1.00

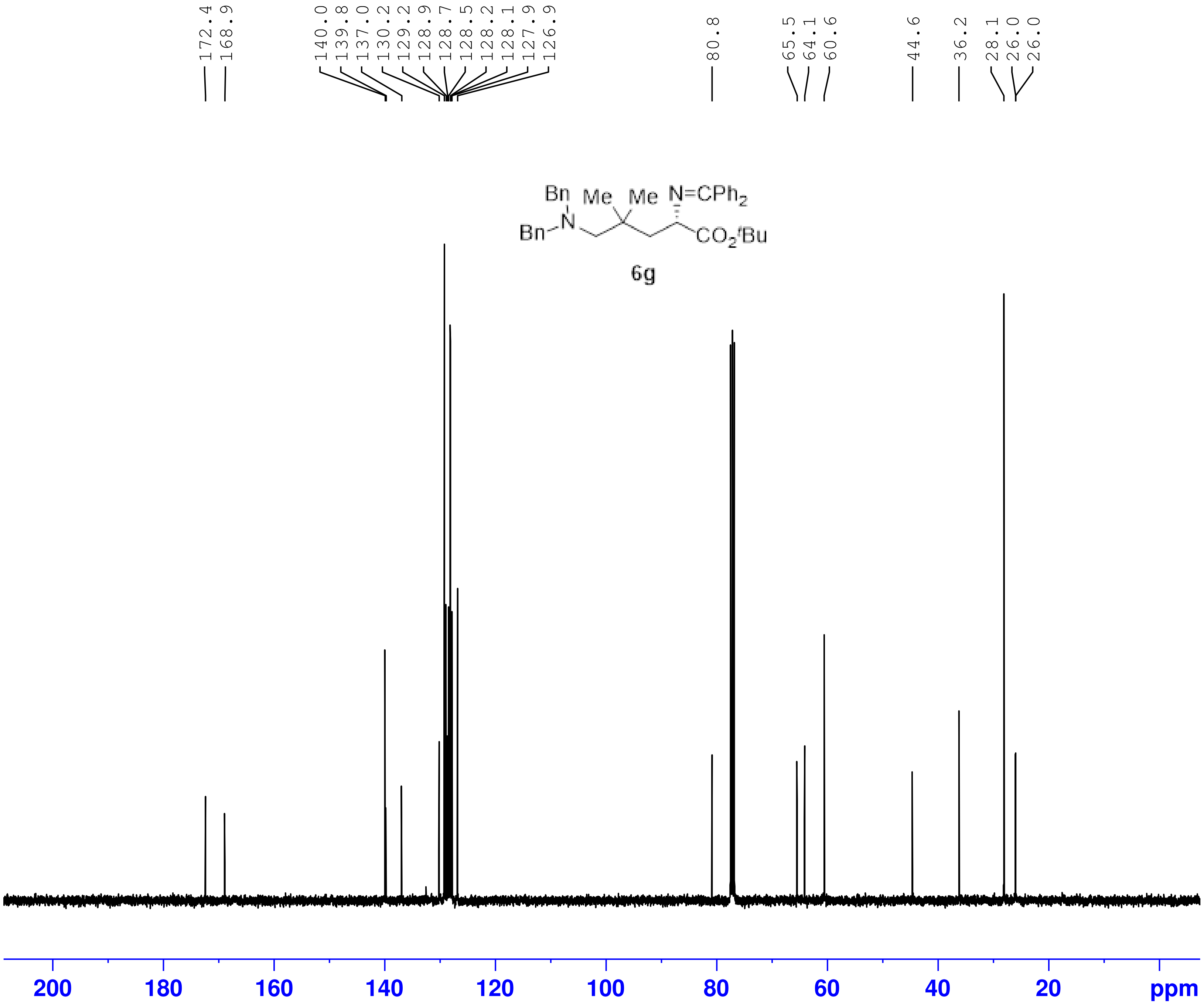

Current Data Parameters
NAME zmh-2-21g-chun-1216
EXPNO 2
PROCNO 1

F2 - Acquisition Parameters
Date_ 20221216
Time 9.54 h
INSTRUM AvanceNeo 400MHz
PROBHD Z163739_0629 (
PULPROG zgpg30
TD 65536
SOLVENT CDCl3
NS 200
DS 4
SWH 23809.523 Hz
FIDRES 0.726609 Hz
AQ 1.3762560 sec
RG 10
DW 21.000 usec
DE 6.50 usec
TE 295.4 K
D1 2.00000000 sec
D11 0.03000000 sec
TD0 1
SFO1 100.6354036 MHz
NUC1 13C
P0 2.67 usec
P1 8.00 usec
PLW1 85.25399780 W
SFO2 400.1816007 MHz
NUC2 1H
CPDPRG[2 waltz65
PCPD2 90.00 usec
PLW2 21.26700020 W
PLW12 0.16802999 W
PLW13 0.08452000 W

F2 - Processing parameters
SI 32768
SF 100.6253323 MHz
WDW EM
SSB 0
LB 1.00 Hz
GB 0
PC 1.40

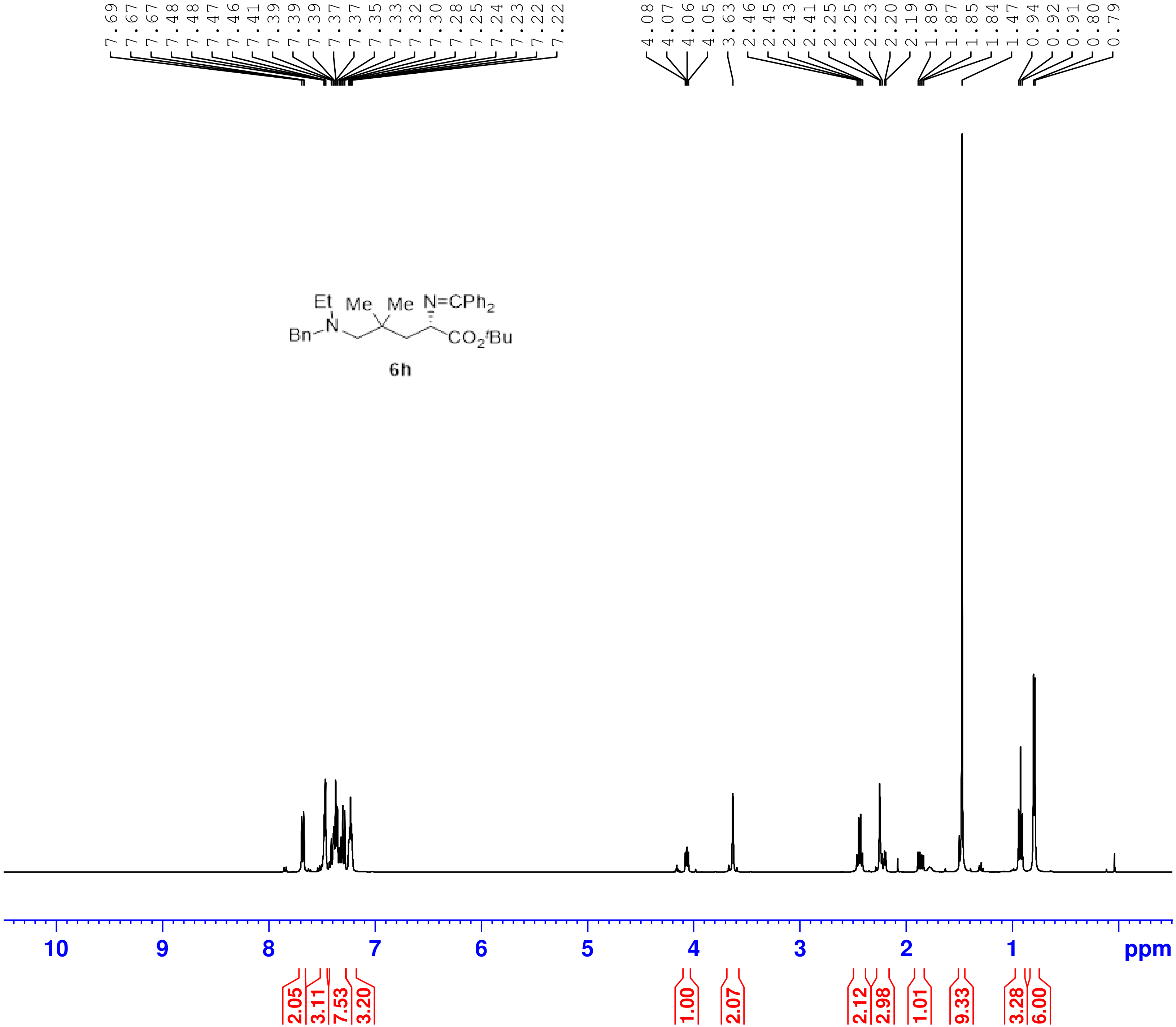

Current Data Parameters
NAME zmh-2-4e-chun
EXPNO 1
PROCNO 1

F2 - Acquisition Parameters
Date_ 20221129
Time 21.46 h
INSTRUM AvanceNeo 400MHz
PROBHD Z163739_0629 (
PULPROG zg30
TD 65536
SOLVENT CDCl3
NS 8
DS 2
SWH 8196.722 Hz
FIDRES 0.250144 Hz
AQ 3.9976959 sec
RG 101
DW 61.000 usec
DE 13.89 usec
TE 297.0 K
D1 1.00000000 sec
TD0 1
SFO1 400.1824711 MHz
NUC1 1H
P0 2.67 usec
P1 8.00 usec
PLW1 21.26700020 W

F2 - Processing parameters
SI 65536
SF 400.1800000 MHz
WDW EM
SSB 0
LB 0.30 Hz
GB 0
PC 1.00

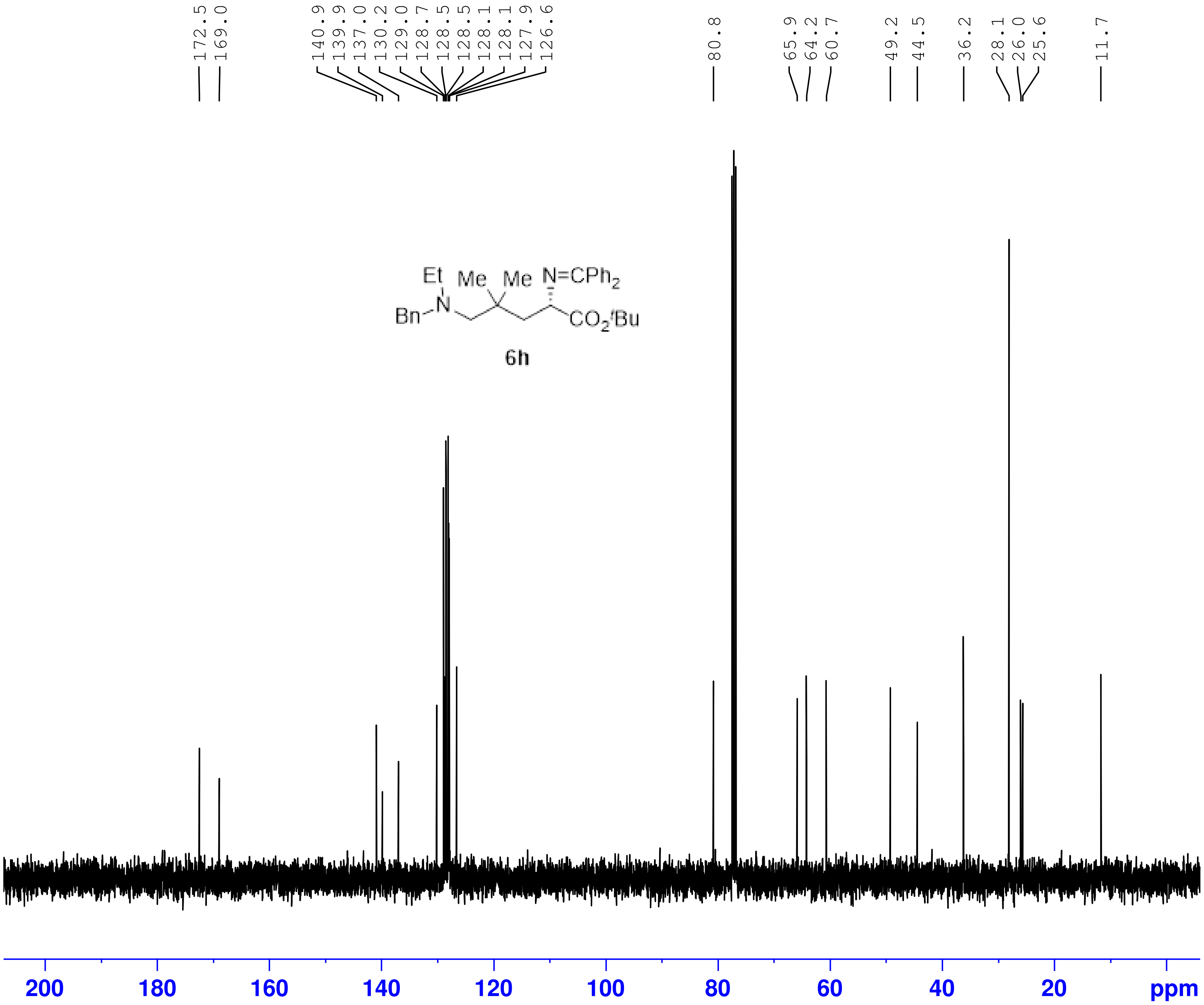

Current Data Parameters
NAME zmh-2-4e-chun
EXPNO 2
PROCNO 1

F2 - Acquisition Parameters
Date_ 20221129
Time 21.49 h
INSTRUM AvanceNeo 400MHz
PROBHD Z163739_0629 (
PULPROG zgpg30
TD 65536
SOLVENT CDCl3
NS 22
DS 4
SWH 23809.523 Hz
FIDRES 0.726609 Hz
AQ 1.3762560 sec
RG 10
DW 21.000 usec
DE 6.50 usec
TE 297.1 K
D1 2.00000000 sec
D11 0.03000000 sec
TD0 1
SFO1 100.6354036 MHz
NUC1 13C
P0 2.67 usec
P1 8.00 usec
PLW1 85.25399780 W
SFO2 400.1816007 MHz
NUC2 1H
CPDPRG[2 waltz65
PCPD2 90.00 usec
PLW2 21.26700020 W
PLW12 0.16802999 W
PLW13 0.08452000 W

F2 - Processing parameters
SI 32768
SF 100.6253308 MHz
WDW EM
SSB 0
LB 1.00 Hz
GB 0
PC 1.40

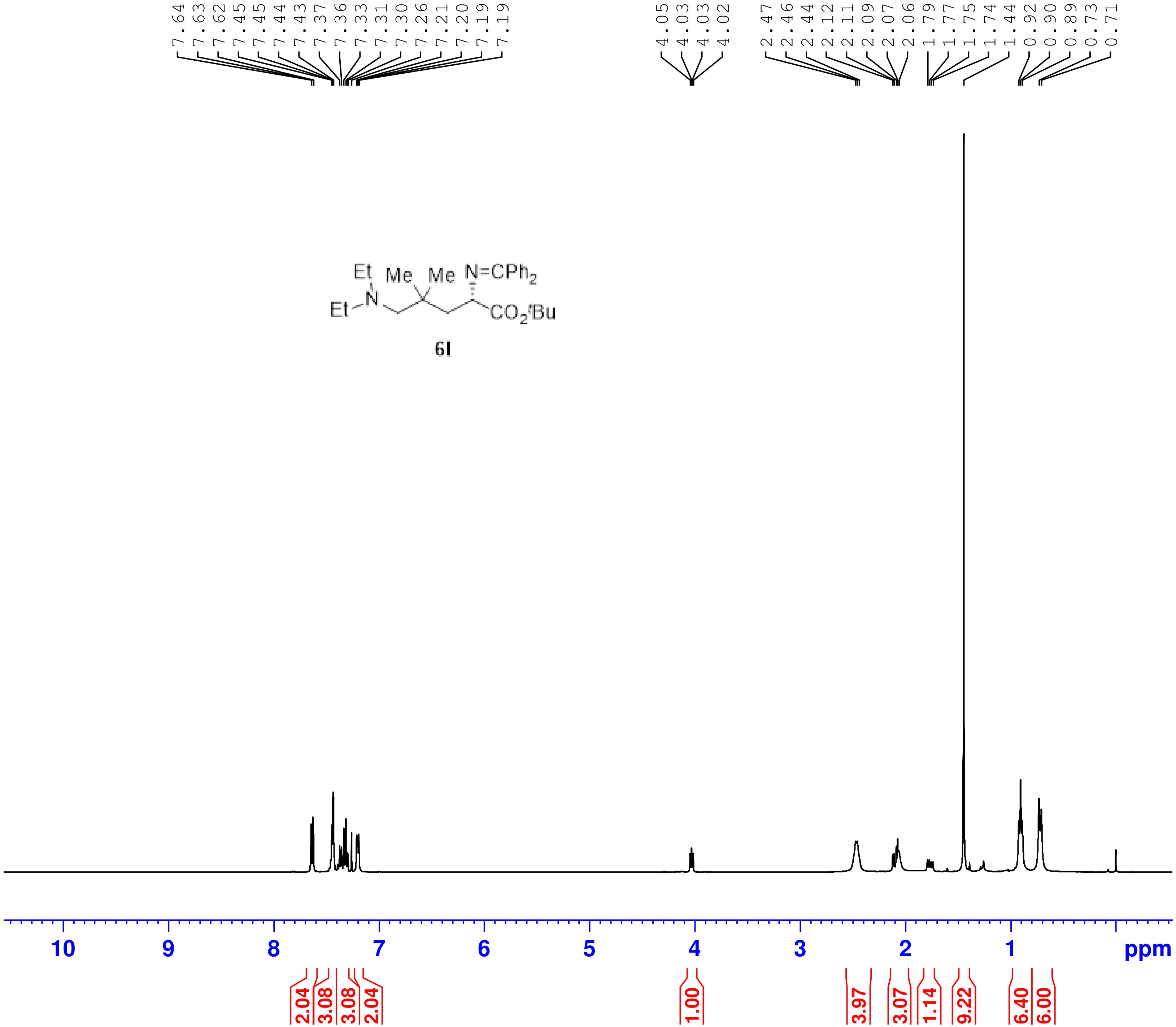

Current Data Parameters
NAME 3i
EXPNO 1
PROCNO 1

F2 - Acquisition Parameters
Date_ 20221216
Time 18.06
INSTRUM spect
PROBHD 5 mm PABBO BB/
PULPROG zg30
TD 65536
SOLVENT CDCl3
NS 8
DS 2
SWH 8012.820 Hz
FIDRES 0.122266 Hz
AQ 4.0894465 sec
RG 34.77
DW 62.400 usec
DE 6.50 usec
TE 294.3 K
D1 1.00000000 sec
TD0 1

======== CHANNEL f1 ========
SFO1 400.1324710 MHz
NUC1 1H
P1 14.50 usec
PLW1 11.99499989 W

F2 - Processing parameters
SI 65536
SF 400.1300099 MHz
WDW EM
SSB 0
LB 0.30 Hz
GB 0
PC 1.00

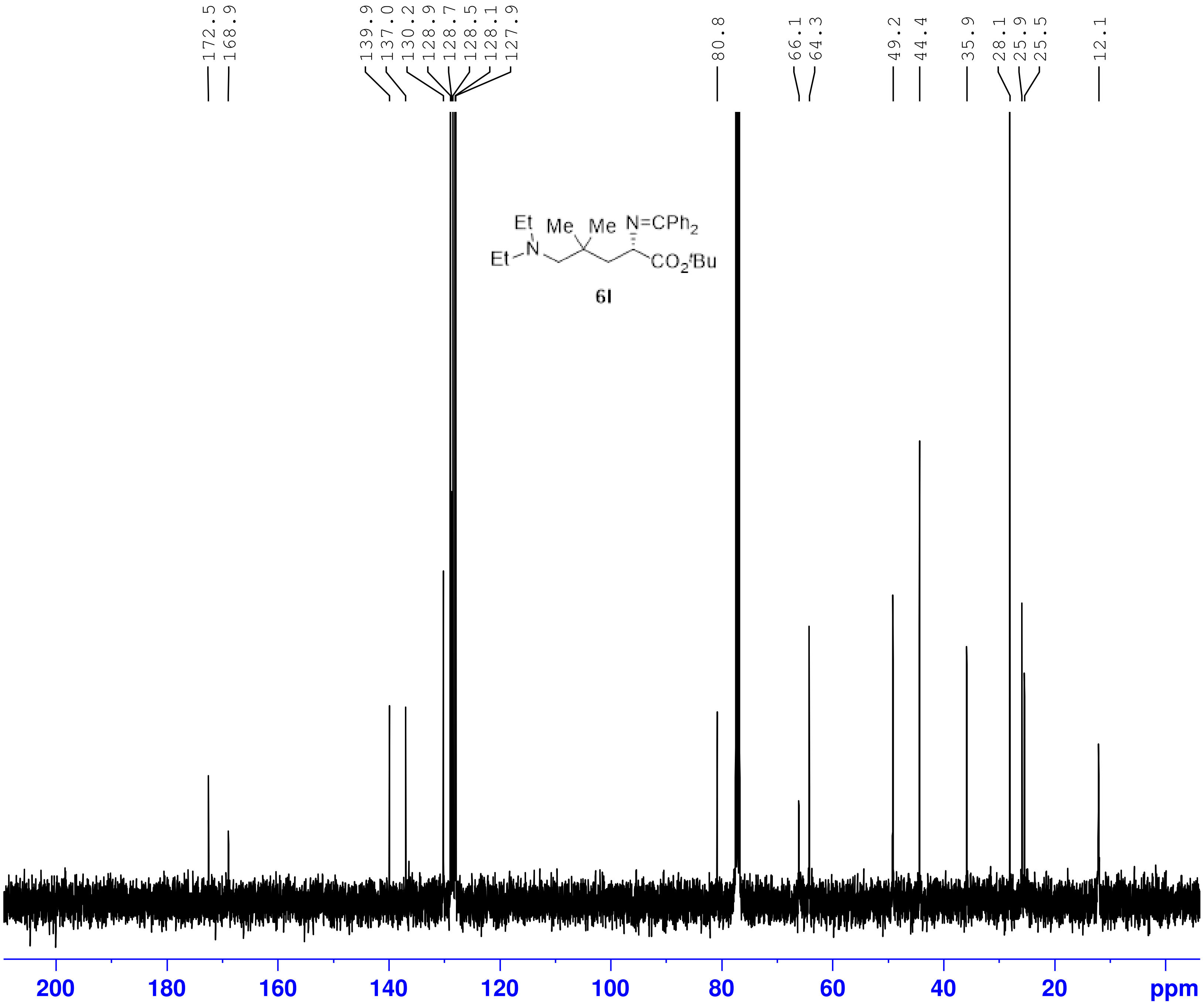


```
Current Data Parameters
NAME      zmh-2-4d-chun-1217
EXPNO                    2
PROCNO                   1

F2 - Acquisition Parameters
Date_             20221217
Time                 12.49
INSTRUM              spect
PROBHD    5 mm PABBO BB/
PULPROG            zgpg30
TD                  65536
SOLVENT             CDCl3
NS                    500
DS                      2
SWH            24038.461 Hz
FIDRES          0.366798 Hz
AQ             1.3631488 sec
RG                 196.92
DW                 20.800 usec
DE                   6.50 usec
TE                  293.1 K
D1             2.00000000 sec
D11            0.03000000 sec
TD0                     1

======== CHANNEL f1 ========
SFO1         100.6228298 MHz
NUC1                  13C
P1                   9.70 usec
PLW1          46.98899841 W

======== CHANNEL f2 ========
SFO2         400.1316005 MHz
NUC2                   1H
CPDPRG[2          waltz16
PCPD2               90.00 usec
PLW2          11.99499989 W
PLW12          0.34213999 W
PLW13          0.27713001 W

F2 - Processing parameters
SI                  32768
SF           100.6127575 MHz
WDW                    EM
SSB                     0
LB                   1.00 Hz
GB                      0
PC                   1.40
```

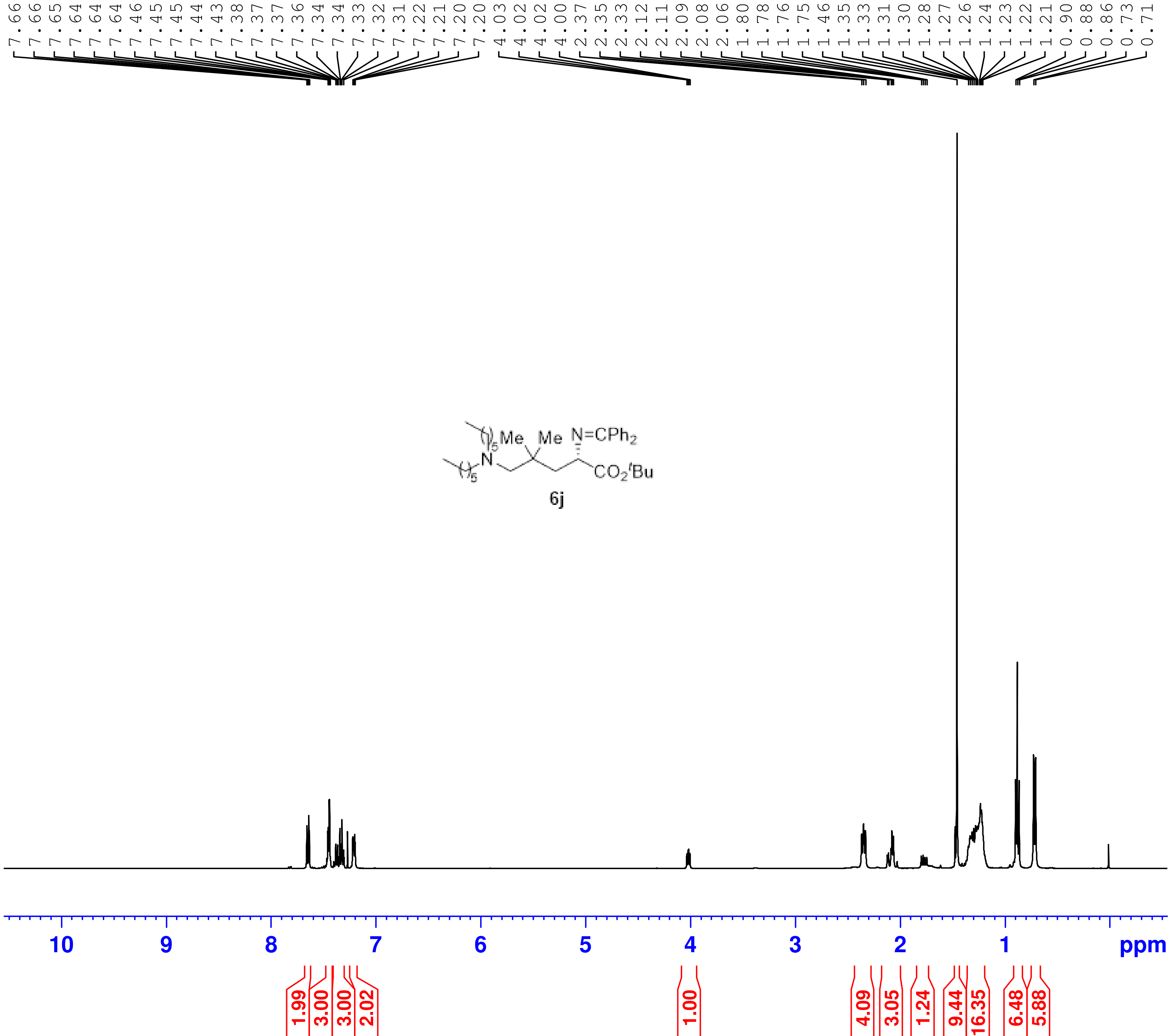

Current Data Parameters
NAME zmh-2-48c-chun-1231-
EXPNO 1
PROCNO 1

F2 - Acquisition Parameters
Date_ 20221231
Time 18.31
INSTRUM spect
PROBHD 5 mm DUL 13C-1
PULPROG zg30
TD 65536
SOLVENT CDCl3
NS 12
DS 2
SWH 8223.685 Hz
FIDRES 0.125483 Hz
AQ 3.9845889 sec
RG 181
DW 60.800 usec
DE 6.00 usec
TE 293.8 K
D1 1.00000000 sec
TD0 1

======== CHANNEL f1 ========
NUC1 1H
P1 15.80 usec
PL1 -1.00 dB
PL1W 12.17476940 W
SFO1 400.1324710 MHz

F2 - Processing parameters
SI 32768
SF 400.1300054 MHz
WDW EM
SSB 0
LB 0.30 Hz
GB 0
PC 1.00

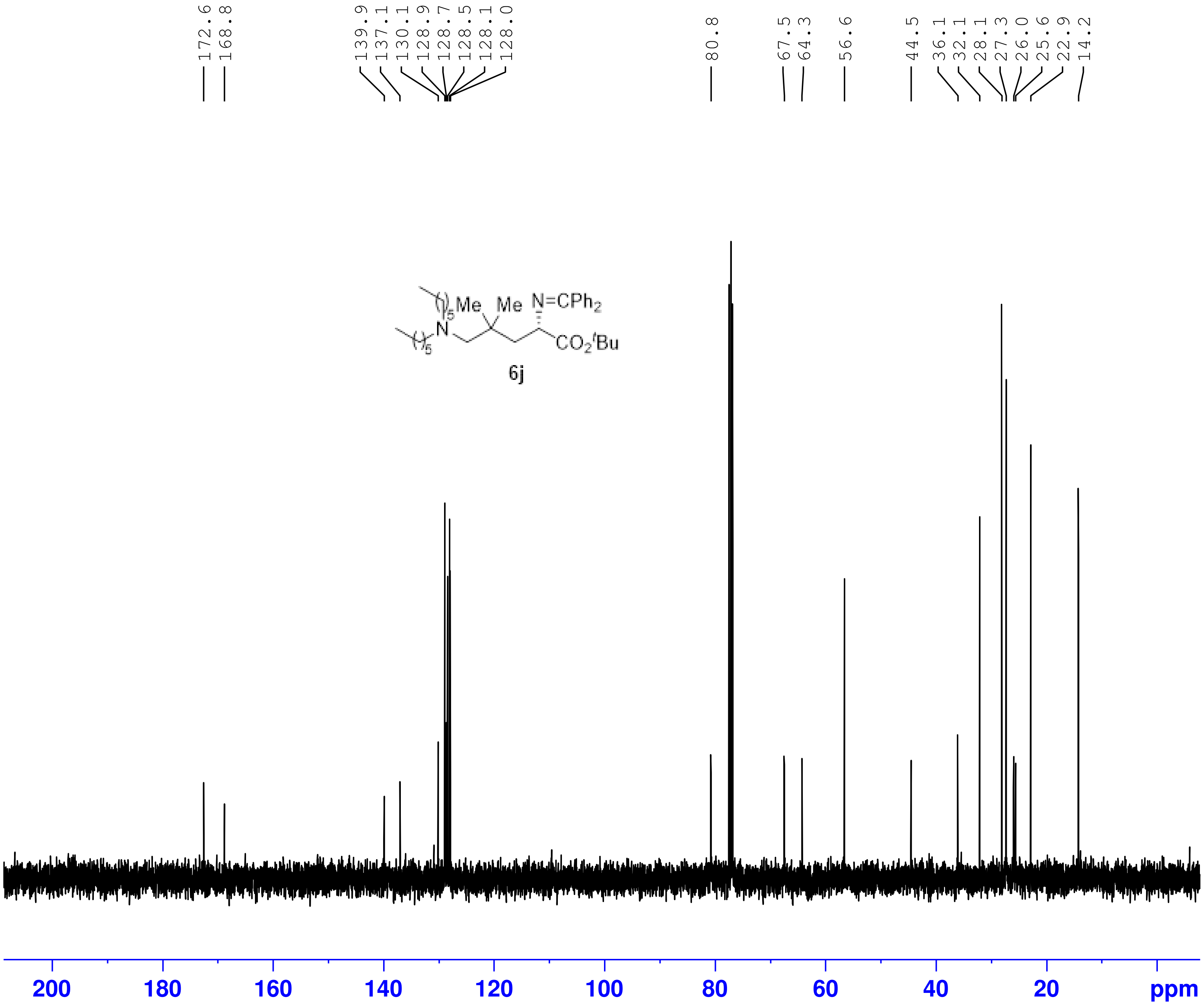

Current Data Parameters
NAME zmh-2-48c-chun-1231-c
EXPNO 1
PROCNO 1

F2 - Acquisition Parameters
Date_ 20221231
Time 18.33
INSTRUM spect
PROBHD 5 mm DUL 13C-1
PULPROG zgpg30
TD 65536
SOLVENT CDCl3
NS 200
DS 1
SWH 24038.461 Hz
FIDRES 0.366798 Hz
AQ 1.3631488 sec
RG 2050
DW 20.800 usec
DE 6.00 usec
TE 294.1 K
D1 2.00000000 sec
D11 0.03000000 sec
TD0 1

======== CHANNEL f1 ========
NUC1 13C
P1 40.00 usec
PL1 -3.00 dB
PL1W 60.64365387 W
SFO1 100.6228298 MHz

======== CHANNEL f2 ========
CPDPRG[2 waltz16
NUC2 1H
PCPD2 80.00 usec
PL2 -1.00 dB
PL12 14.39 dB
PL13 18.00 dB
PL2W 12.17476940 W
PL12W 0.35193357 W
PL13W 0.15327126 W
SFO2 400.1316005 MHz

F2 - Processing parameters
SI 32768
SF 100.6127564 MHz
WDW EM
SSB 0
LB 1.00 Hz
GB 0
PC 1.40

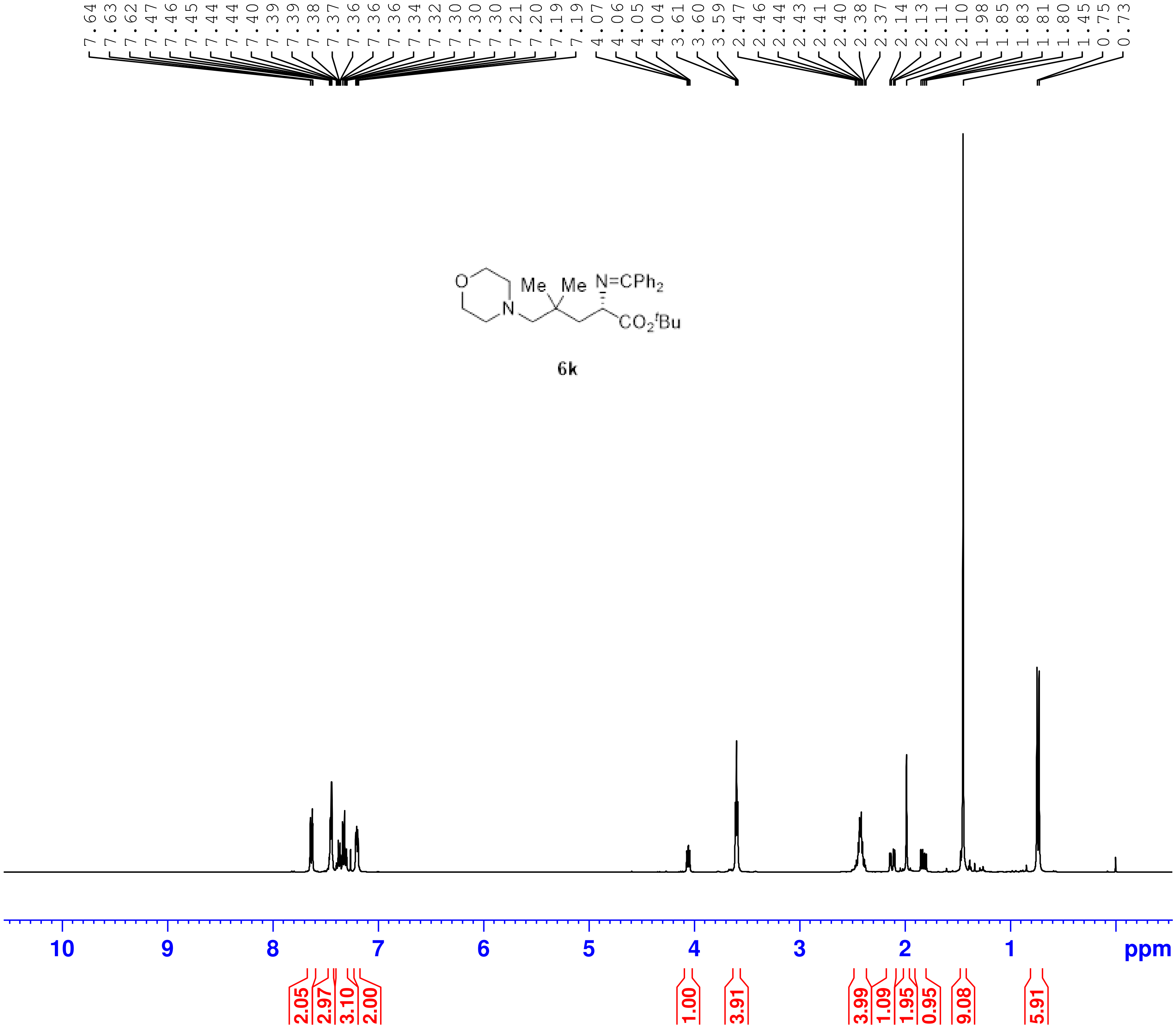

Current Data Parameters	
NAME	3k
EXPNO	1
PROCNO	1

F2 - Acquisition Parameters	
Date_	20221212
Time	18.58 h
INSTRUM	AvanceNeo 400MHz
PROBHD	Z163739_0629 (
PULPROG	zg30
TD	65536
SOLVENT	CDCl3
NS	16
DS	2
SWH	8196.722 Hz
FIDRES	0.250144 Hz
AQ	3.9976959 sec
RG	57
DW	61.000 usec
DE	13.89 usec
TE	293.8 K
D1	1.00000000 sec
TD0	1
SFO1	400.1824711 MHz
NUC1	1H
P0	2.67 usec
P1	8.00 usec
PLW1	21.26700020 W

F2 - Processing parameters	
SI	65536
SF	400.1800084 MHz
WDW	EM
SSB	0
LB	0.30 Hz
GB	0
PC	1.00

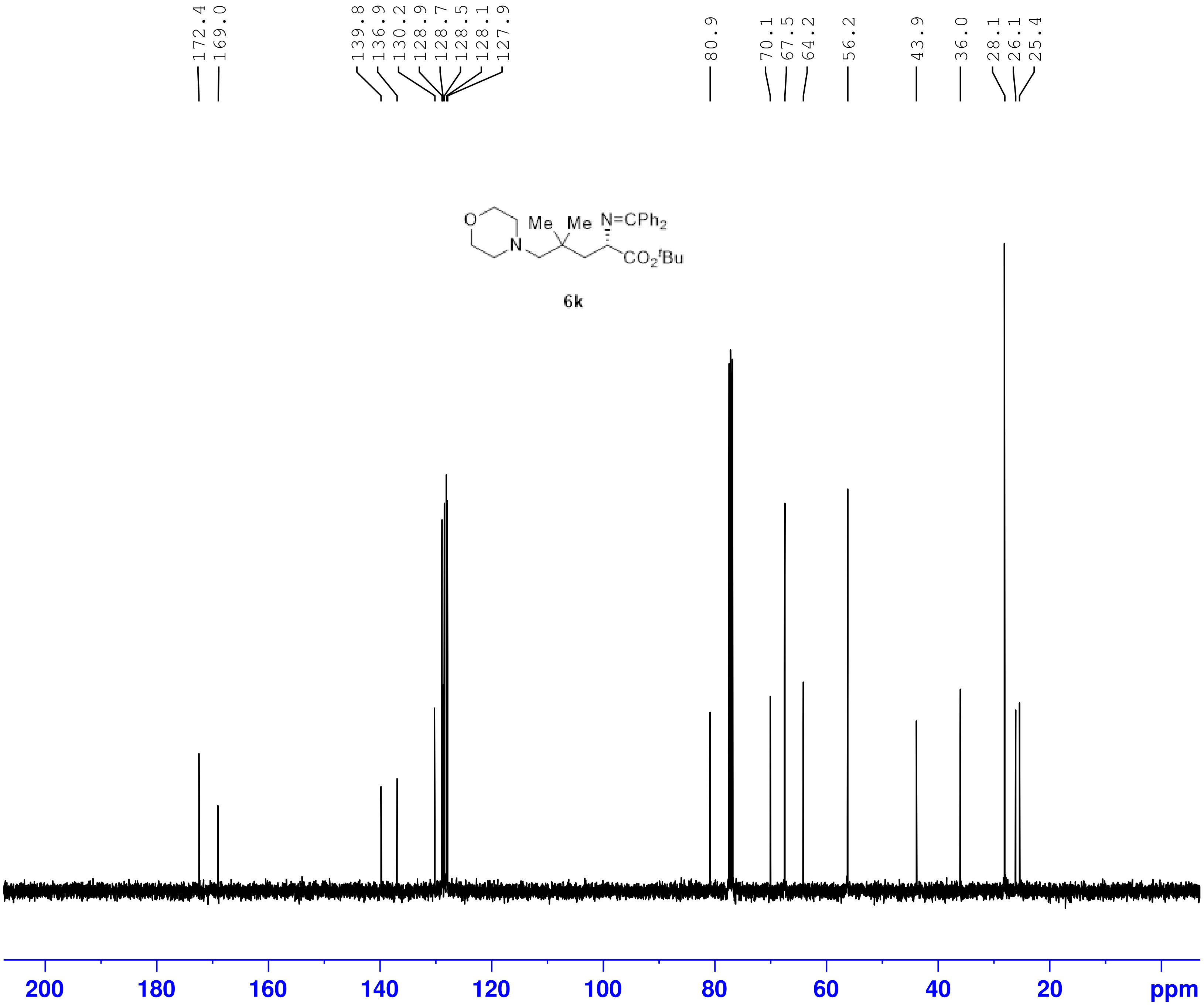

Current Data Parameters
NAME zmh-2-4g-chun-1212
EXPNO 2
PROCNO 1

F2 - Acquisition Parameters
Date_ 20221212
Time 19.03 h
INSTRUM AvanceNeo 400MHz
PROBHD Z163739_0629 (
PULPROG zgpg30
TD 65536
SOLVENT CDCl3
NS 64
DS 4
SWH 23809.523 Hz
FIDRES 0.726609 Hz
AQ 1.3762560 sec
RG 10
DW 21.000 usec
DE 6.50 usec
TE 294.2 K
D1 2.00000000 sec
D11 0.03000000 sec
TD0 1
SFO1 100.6354036 MHz
NUC1 13C
P0 2.67 usec
P1 8.00 usec
PLW1 85.25399780 W
SFO2 400.1816007 MHz
NUC2 1H
CPDPRG[2 waltz65
PCPD2 90.00 usec
PLW2 21.26700020 W
PLW12 0.16802999 W
PLW13 0.08452000 W

F2 - Processing parameters
SI 32768
SF 100.6253330 MHz
WDW EM
SSB 0
LB 1.00 Hz
GB 0
PC 1.40

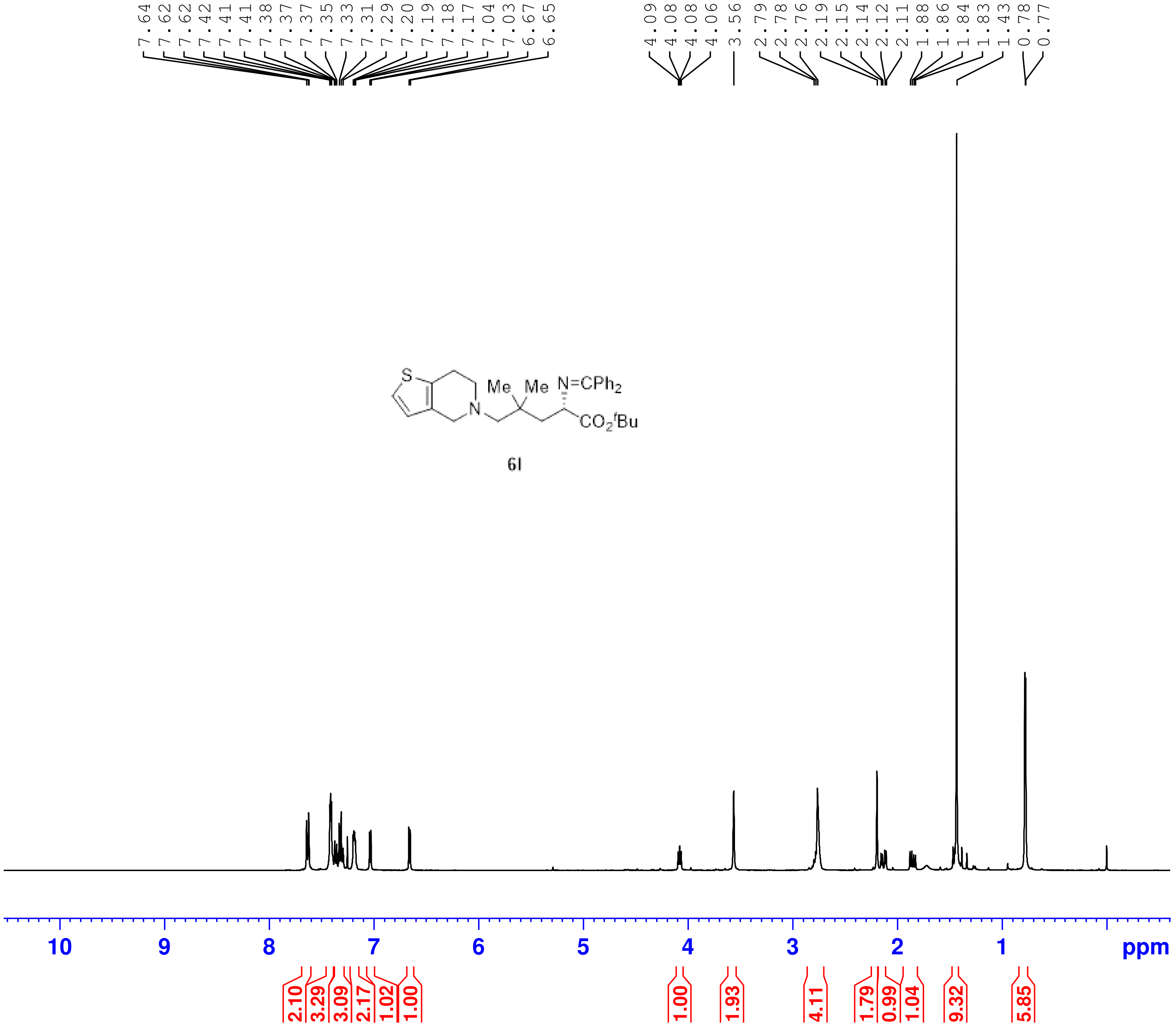

Current Data Parameters
NAME 31
EXPNO 1
PROCNO 1

F2 - Acquisition Parameters
Date_ 20221208
Time 21.49
INSTRUM spect
PROBHD 5 mm DUL 13C-1
PULPROG zg30
TD 65536
SOLVENT CDCl3
NS 9
DS 2
SWH 8223.685 Hz
FIDRES 0.125483 Hz
AQ 3.9845889 sec
RG 228
DW 60.800 usec
DE 6.00 usec
TE 293.3 K
D1 1.00000000 sec
TD0 1

======== CHANNEL f1 ========
NUC1 1H
P1 15.80 usec
PL1 -1.00 dB
PL1W 12.17476940 W
SFO1 400.1324710 MHz

F2 - Processing parameters
SI 32768
SF 400.1300125 MHz
WDW EM
SSB 0
LB 0.30 Hz
GB 0
PC 1.00

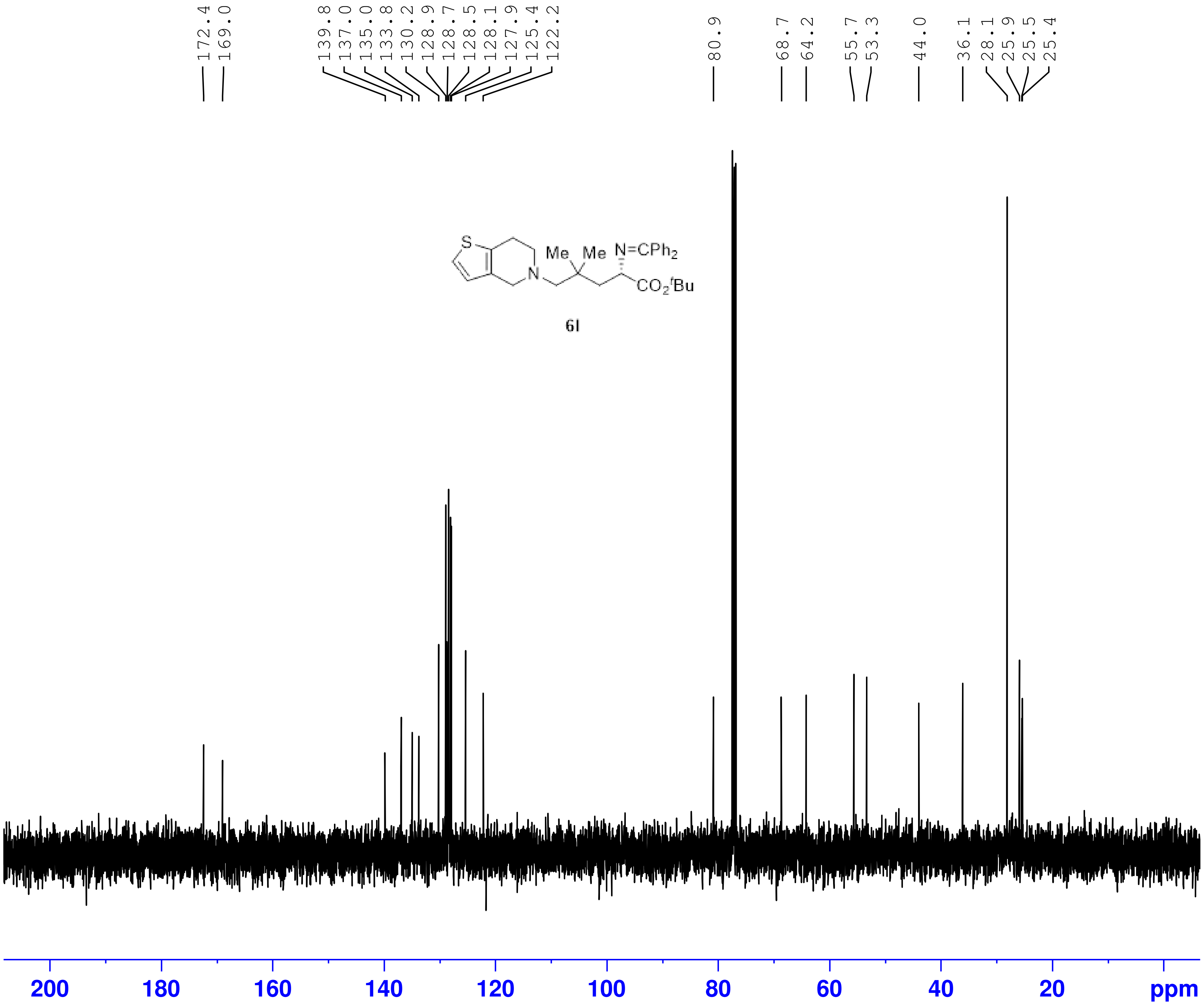


```
Current Data Parameters
NAME          zmh-2-53d-C
EXPNO                   1
PROCNO                  1

F2 - Acquisition Parameters
Date_            20221208
Time                21.50
INSTRUM             spect
PROBHD   5 mm DUL 13C-1
PULPROG           zgpg30
TD                 65536
SOLVENT            CDCl3
NS                    88
DS                     1
SWH            24038.461 Hz
FIDRES          0.366798 Hz
AQ             1.3631488 sec
RG                  2050
DW                20.800 usec
DE                  6.00 usec
TE                 293.4 K
D1            2.00000000 sec
D11           0.03000000 sec
TD0                    1

======== CHANNEL f1 ========
NUC1                 13C
P1                 40.00 usec
PL1                -3.00 dB
PL1W         60.64365387 W
SFO1         100.6228298 MHz

======== CHANNEL f2 ========
CPDPRG[2         waltz16
NUC2                  1H
PCPD2              80.00 usec
PL2                -1.00 dB
PL12               14.39 dB
PL13               18.00 dB
PL2W         12.17476940 W
PL12W         0.35193357 W
PL13W         0.15327126 W
SFO2         400.1316005 MHz

F2 - Processing parameters
SI                 32768
SF           100.6127578 MHz
WDW                   EM
SSB                    0
LB                  1.00 Hz
GB                     0
PC                  1.40
```

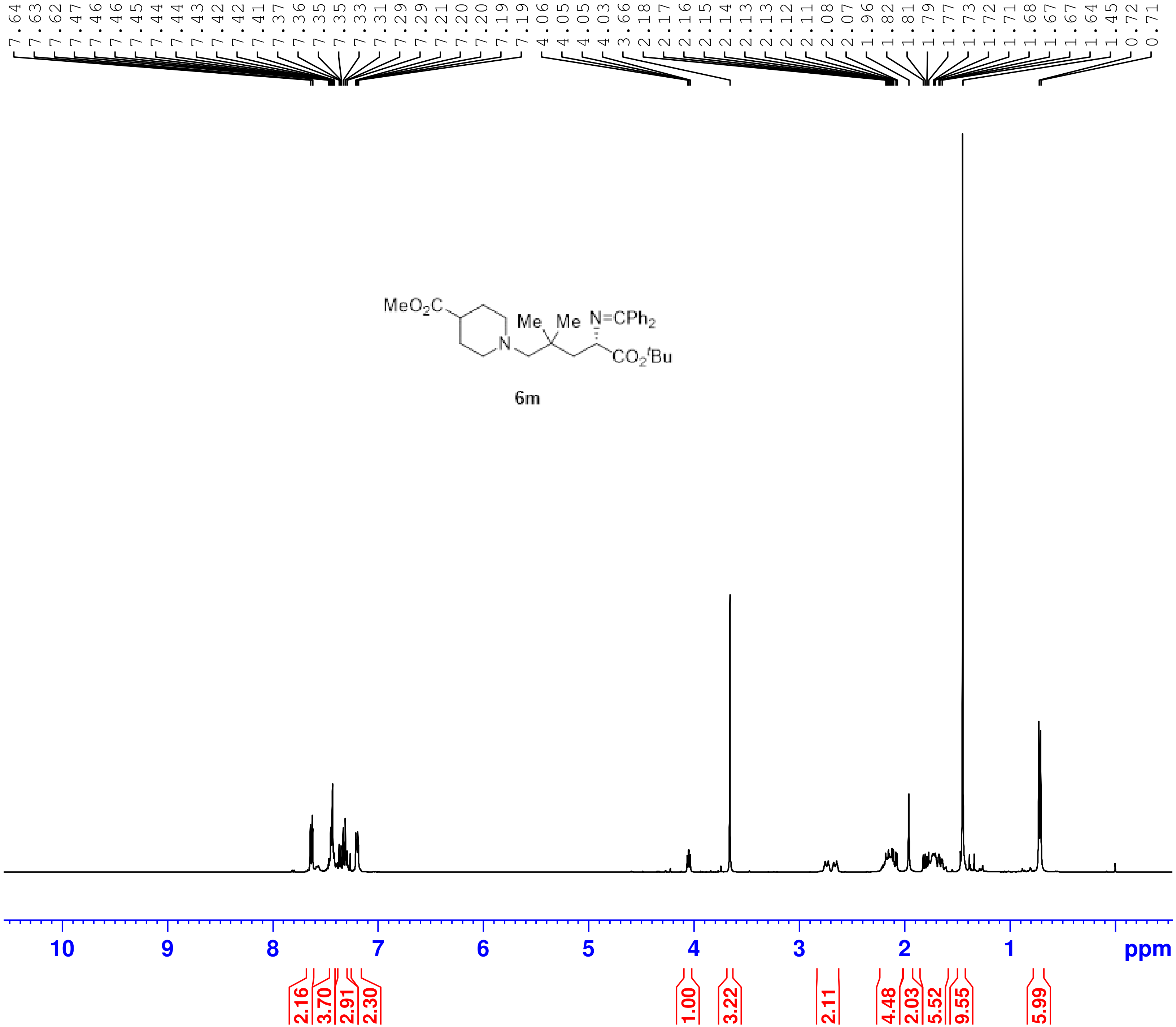

Current Data Parameters
NAME 3m
EXPNO 2
PROCNO 1

F2 - Acquisition Parameters
Date_ 20230116
Time 17.59
INSTRUM spect
PROBHD 5 mm DUL 13C-1
PULPROG zg30
TD 65536
SOLVENT CDCl3
NS 16
DS 2
SWH 8223.685 Hz
FIDRES 0.125483 Hz
AQ 3.9845889 sec
RG 64
DW 60.800 usec
DE 6.00 usec
TE 292.6 K
D1 1.00000000 sec
TD0 1

======== CHANNEL f1 ========
NUC1 1H
P1 15.80 usec
PL1 -1.00 dB
PL1W 12.17476940 W
SFO1 400.1324710 MHz

F2 - Processing parameters
SI 32768
SF 400.1300076 MHz
WDW EM
SSB 0
LB 0.30 Hz
GB 0
PC 1.00

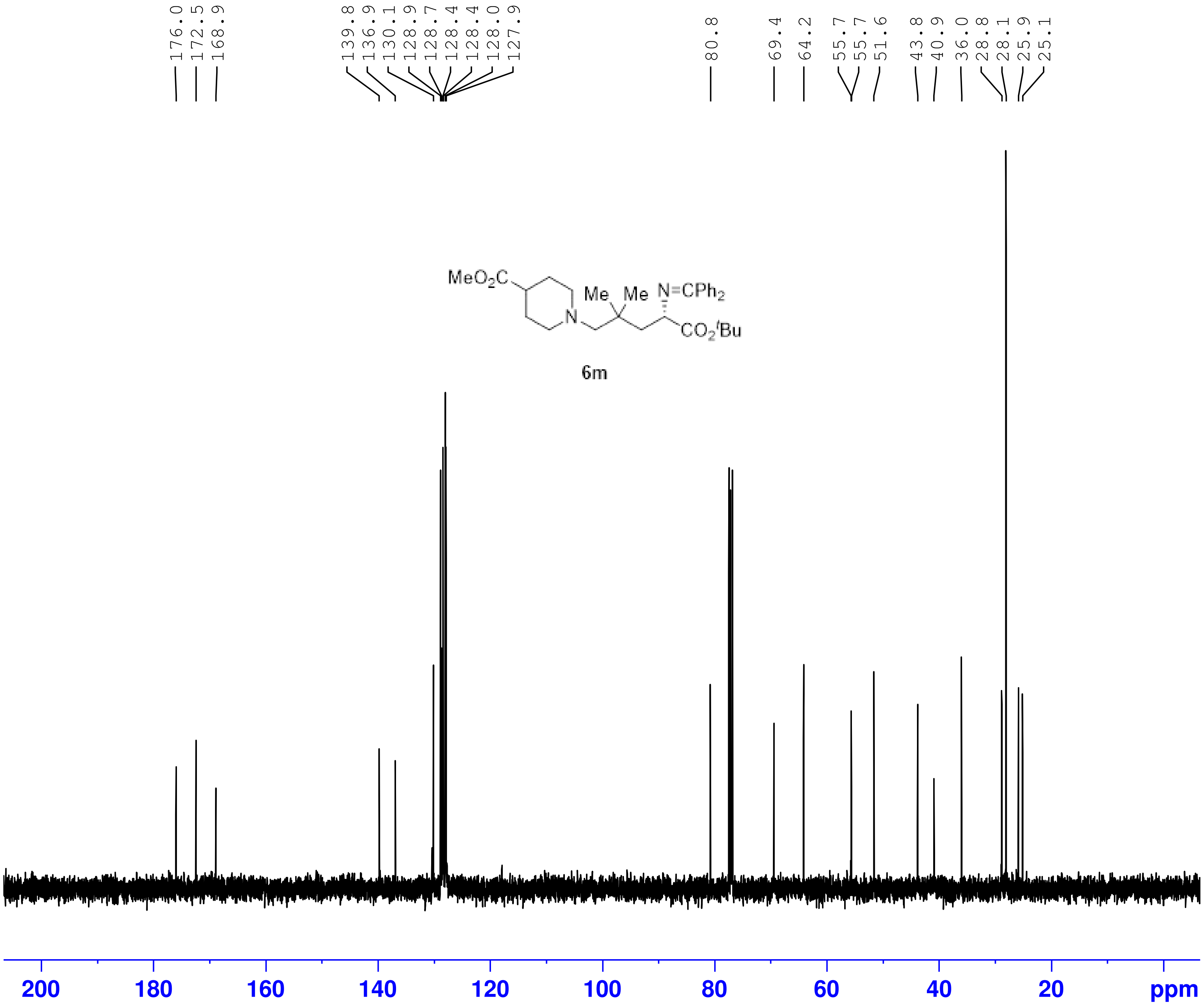

Current Data Parameters
NAME 3m
EXPNO 1
PROCNO 1

F2 - Acquisition Parameters
Date_ 20230116
Time 18.01
INSTRUM spect
PROBHD 5 mm DUL 13C-1
PULPROG zgpg30
TD 65536
SOLVENT CDCl3
NS 91
DS 1
SWH 24038.461 Hz
FIDRES 0.366798 Hz
AQ 1.3631488 sec
RG 2050
DW 20.800 usec
DE 6.00 usec
TE 292.7 K
D1 2.00000000 sec
D11 0.03000000 sec
TD0 1

======== CHANNEL f1 ========
NUC1 13C
P1 40.00 usec
PL1 -3.00 dB
PL1W 60.64365387 W
SFO1 100.6228298 MHz

======== CHANNEL f2 ========
CPDPRG[2 waltz16
NUC2 1H
PCPD2 80.00 usec
PL2 -1.00 dB
PL12 14.39 dB
PL13 18.00 dB
PL2W 12.17476940 W
PL12W 0.35193357 W
PL13W 0.15327126 W
SFO2 400.1316005 MHz

F2 - Processing parameters
SI 32768
SF 100.6127632 MHz
WDW EM
SSB 0
LB 1.00 Hz
GB 0
PC 1.40

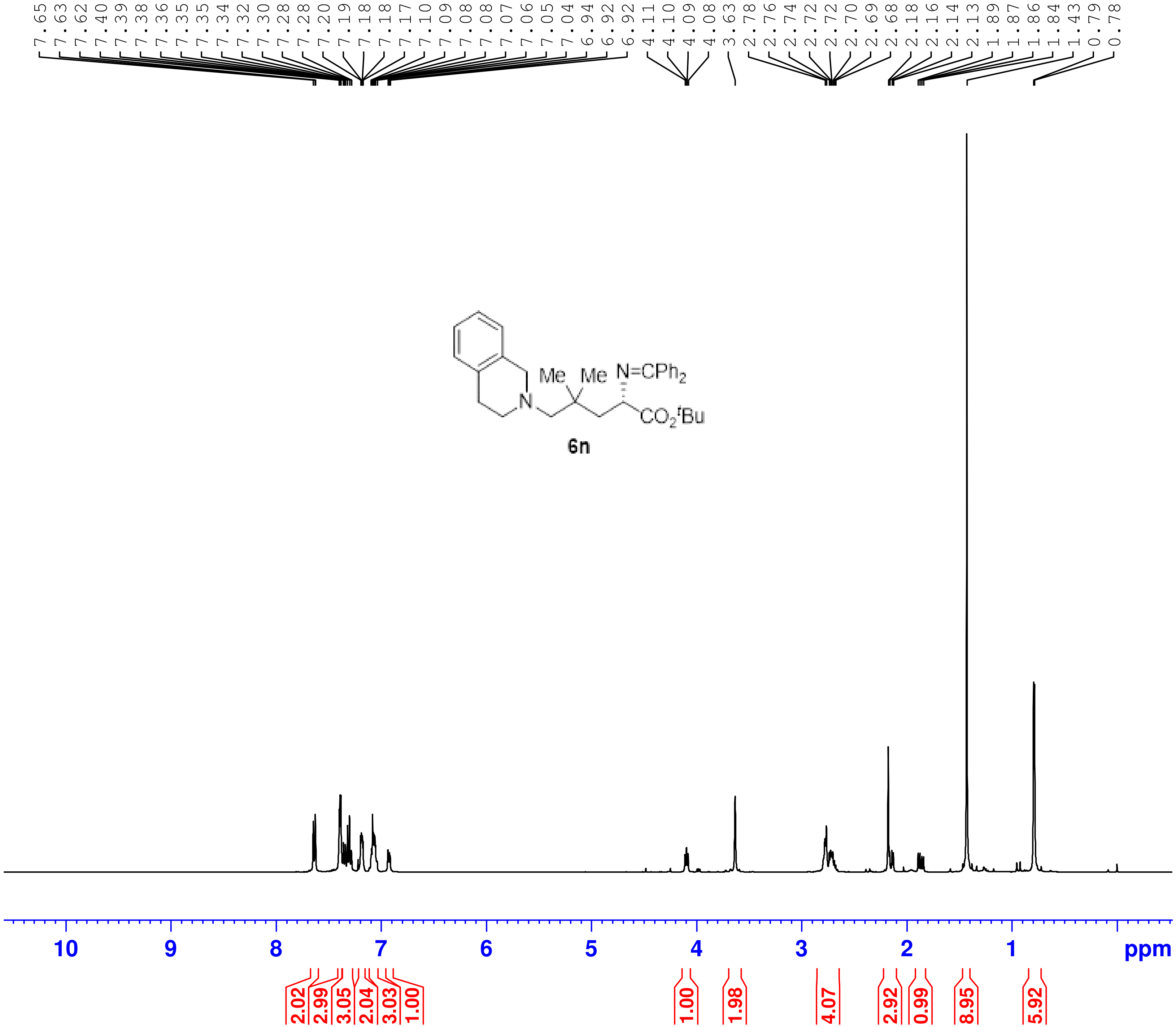

Current Data Parameters
NAME 3n
EXPNO 1
PROCNO 1

F2 - Acquisition Parameters
Date_ 20221208
Time 21.44
INSTRUM spect
PROBHD 5 mm DUL 13C-1
PULPROG zg30
TD 65536
SOLVENT CDCl3
NS 9
DS 2
SWH 8223.685 Hz
FIDRES 0.125483 Hz
AQ 3.9845889 sec
RG 64
DW 60.800 usec
DE 6.00 usec
TE 293.2 K
D1 1.00000000 sec
TD0 1

======== CHANNEL f1 ========
NUC1 1H
P1 15.80 usec
PL1 -1.00 dB
PL1W 12.17476940 W
SFO1 400.1324710 MHz

F2 - Processing parameters
SI 32768
SF 400.1300258 MHz
WDW EM
SSB 0
LB 0.30 Hz
GB 0
PC 1.00

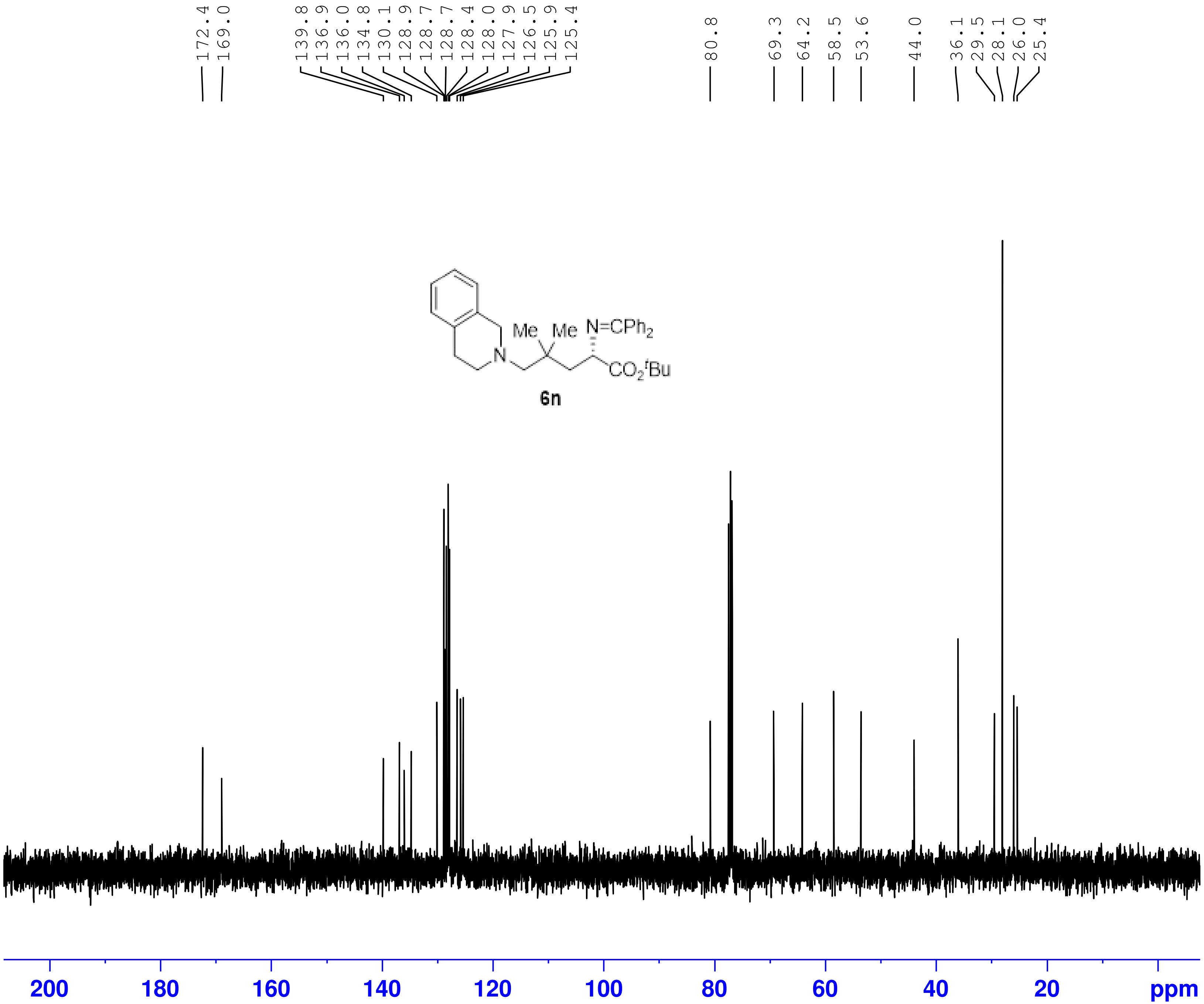

Current Data Parameters
NAME zmh-2-53c-C
EXPNO 1
PROCNO 1

F2 - Acquisition Parameters
Date_ 20221208
Time 21.45
INSTRUM spect
PROBHD 5 mm DUL 13C-1
PULPROG zgpg30
TD 65536
SOLVENT CDCl3
NS 31
DS 1
SWH 24038.461 Hz
FIDRES 0.366798 Hz
AQ 1.3631488 sec
RG 2050
DW 20.800 usec
DE 6.00 usec
TE 293.4 K
D1 2.00000000 sec
D11 0.03000000 sec
TD0 1

======== CHANNEL f1 ========
NUC1 13C
P1 40.00 usec
PL1 -3.00 dB
PL1W 60.64365387 W
SFO1 100.6228298 MHz

======== CHANNEL f2 ========
CPDPRG[2 waltz16
NUC2 1H
PCPD2 80.00 usec
PL2 -1.00 dB
PL12 14.39 dB
PL13 18.00 dB
PL2W 12.17476940 W
PL12W 0.35193357 W
PL13W 0.15327126 W
SFO2 400.1316005 MHz

F2 - Processing parameters
SI 32768
SF 100.6127666 MHz
WDW EM
SSB 0
LB 1.00 Hz
GB 0
PC 1.40

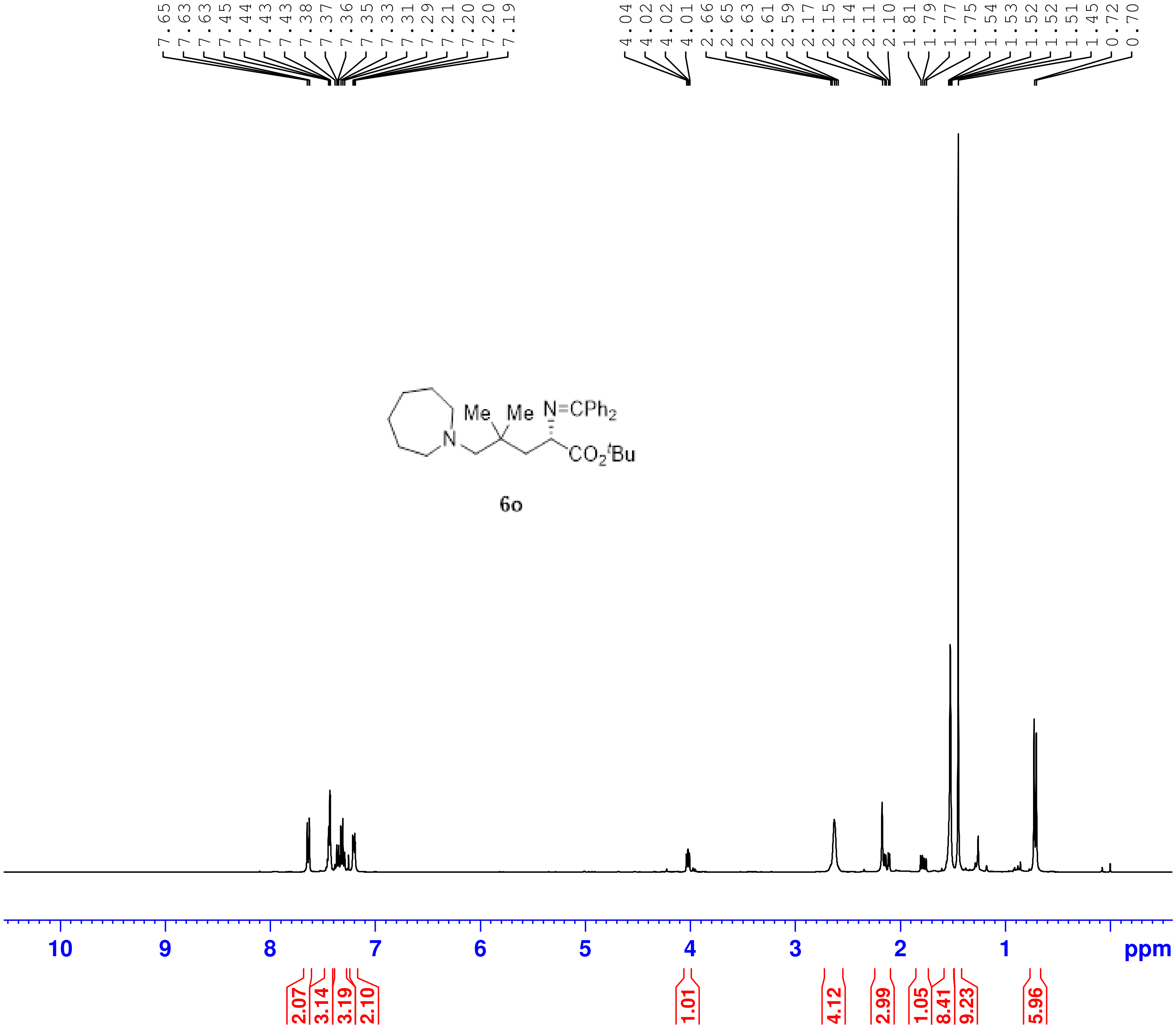

Current Data Parameters
NAME 3o
EXPNO 1
PROCNO 1

F2 - Acquisition Parameters
Date_ 20230107
Time 9.25
INSTRUM spect
PROBHD 5 mm DUL 13C-1
PULPROG zg30
TD 65536
SOLVENT CDCl3
NS 16
DS 2
SWH 8223.685 Hz
FIDRES 0.125483 Hz
AQ 3.9845889 sec
RG 114
DW 60.800 usec
DE 6.00 usec
TE 295.0 K
D1 1.00000000 sec
TD0 1

======== CHANNEL f1 ========
NUC1 1H
P1 15.80 usec
PL1 -1.00 dB
PL1W 12.17476940 W
SFO1 400.1324710 MHz

F2 - Processing parameters
SI 32768
SF 400.1300119 MHz
WDW EM
SSB 0
LB 0.30 Hz
GB 0
PC 1.00

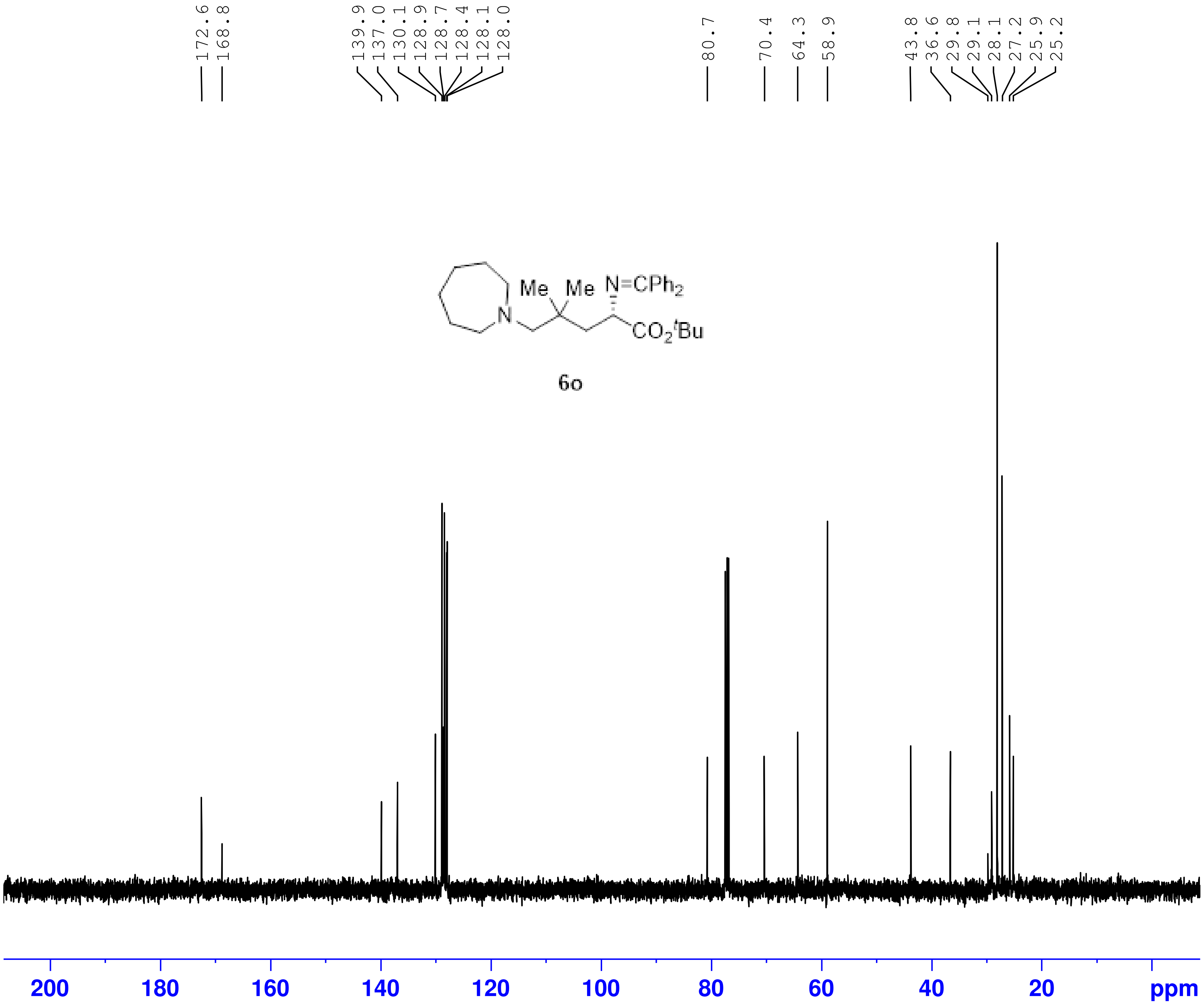

Current Data Parameters
NAME zmh-2-76-c
EXPNO 1
PROCNO 1

F2 - Acquisition Parameters
Date_ 20230107
Time 9.28
INSTRUM spect
PROBHD 5 mm DUL 13C-1
PULPROG zgpg30
TD 65536
SOLVENT CDCl3
NS 103
DS 1
SWH 24038.461 Hz
FIDRES 0.366798 Hz
AQ 1.3631488 sec
RG 2050
DW 20.800 usec
DE 6.00 usec
TE 295.1 K
D1 2.00000000 sec
D11 0.03000000 sec
TD0 1

======== CHANNEL f1 ========
NUC1 13C
P1 40.00 usec
PL1 -3.00 dB
PL1W 60.64365387 W
SFO1 100.6228298 MHz

======== CHANNEL f2 ========
CPDPRG[2 waltz16
NUC2 1H
PCPD2 80.00 usec
PL2 -1.00 dB
PL12 14.39 dB
PL13 18.00 dB
PL2W 12.17476940 W
PL12W 0.35193357 W
PL13W 0.15327126 W
SFO2 400.1316005 MHz

F2 - Processing parameters
SI 32768
SF 100.6127601 MHz
WDW EM
SSB 0
LB 1.00 Hz
GB 0
PC 1.40

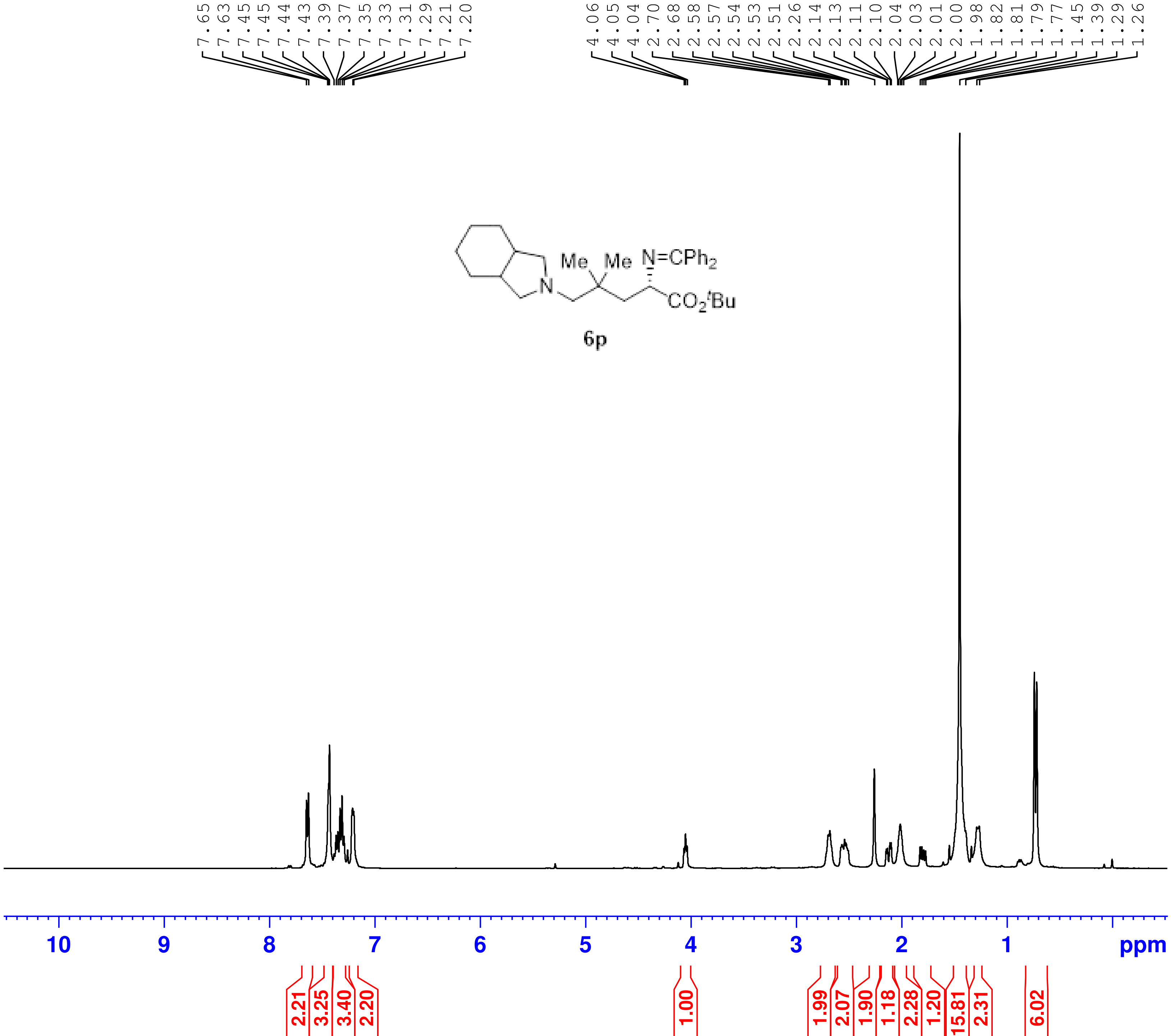

6p

Current Data Parameters

NAME	zmh-2-98d-chun
EXPNO	1
PROCNO	1

F2 - Acquisition Parameters

Date_	20230113
Time	18.21 h
INSTRUM	AvanceNeo 400MHz
PROBHD	Z163739_0629 (
PULPROG	zg30
TD	65536
SOLVENT	CDCl3
NS	8
DS	2
SWH	8196.722 Hz
FIDRES	0.250144 Hz
AQ	3.9976959 sec
RG	45.2
DW	61.000 usec
DE	13.89 usec
TE	296.7 K
D1	1.00000000 sec
TD0	1
SFO1	400.1824711 MHz
NUC1	1H
P0	2.67 usec
P1	8.00 usec
PLW1	21.26700020 W

F2 - Processing parameters

SI	65536
SF	400.1800100 MHz
WDW	EM
SSB	0
LB	0.30 Hz
GB	0
PC	1.00

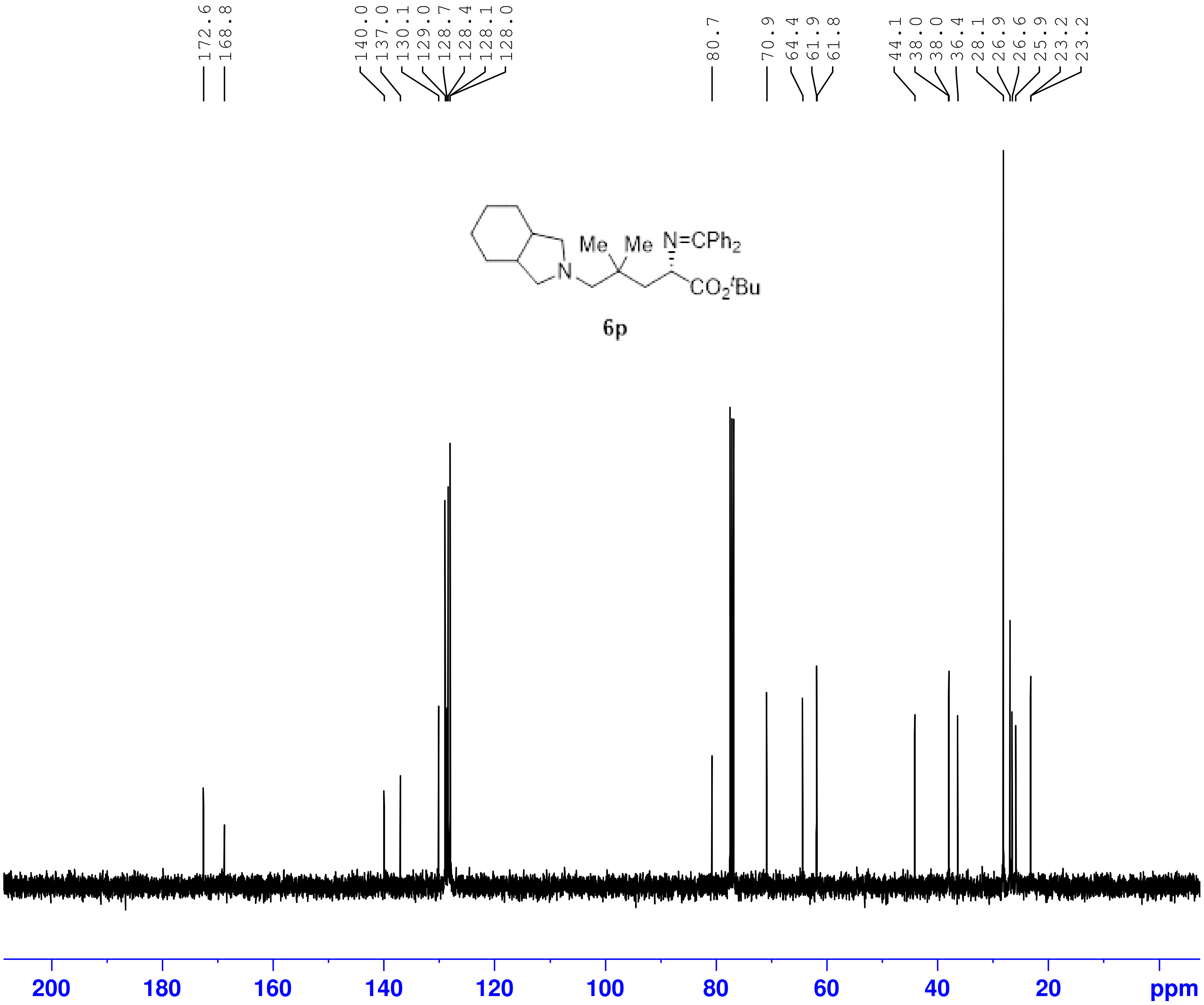


```
Current Data Parameters
NAME       zmh-2-98d-chun
EXPNO                   2
PROCNO                  1

F2 - Acquisition Parameters
Date_            20230113
Time                18.28 h
INSTRUM   AvanceNeo 400MHz
PROBHD    Z163739_0629 (
PULPROG           zgpg30
TD                 65536
SOLVENT            CDCl3
NS                   101
DS                     4
SWH            23809.523 Hz
FIDRES          0.726609 Hz
AQ             1.3762560 sec
RG                  11.3
DW                21.000 usec
DE                  6.50 usec
TE                 297.5 K
D1            2.00000000 sec
D11           0.03000000 sec
TD0                    1
SFO1         100.6354036 MHz
NUC1                 13C
P0                  2.67 usec
P1                  8.00 usec
PLW1         85.25399780 W
SFO2         400.1816007 MHz
NUC2                  1H
CPDPRG[2         waltz65
PCPD2              90.00 usec
PLW2         21.26700020 W
PLW12         0.16802999 W
PLW13         0.08452000 W

F2 - Processing parameters
SI                 32768
SF           100.6253295 MHz
WDW                   EM
SSB                    0
LB                  1.00 Hz
GB                     0
PC                  1.40
```

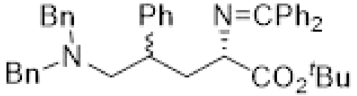

6q

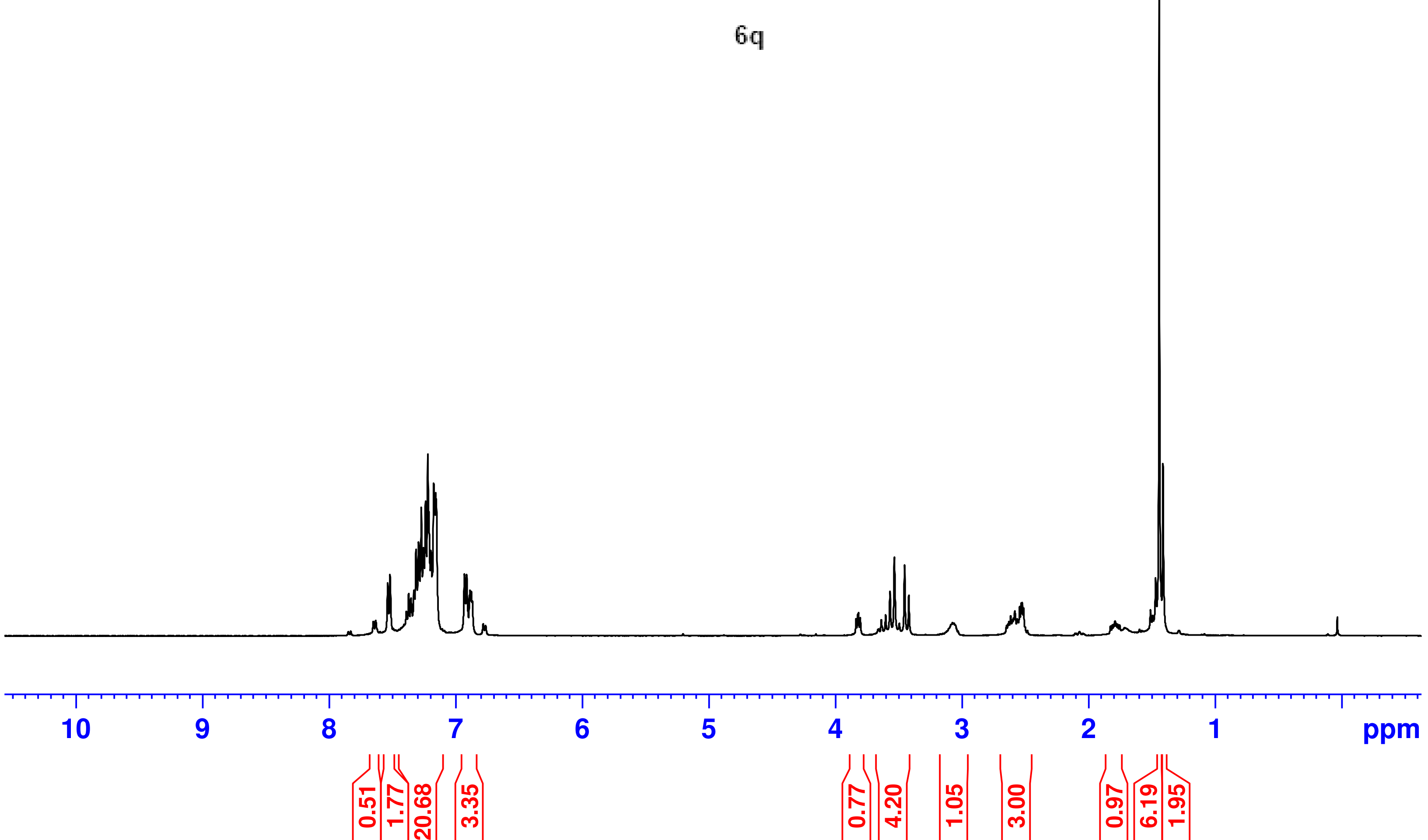

Current Data Parameters	
NAME	3q
EXPNO	2
PROCNO	1
F2 - Acquisition Parameters	
Date_	20230119
Time	9.41
INSTRUM	spect
PROBHD	5 mm DUL 13C-1
PULPROG	zg30
TD	65536
SOLVENT	CDCl3
NS	16
DS	2
SWH	8223.685 Hz
FIDRES	0.125483 Hz
AQ	3.9845889 sec
RG	203
DW	60.800 usec
DE	6.00 usec
TE	293.6 K
D1	1.00000000 sec
TD0	1
======== CHANNEL f1 ========	
NUC1	1H
P1	15.80 usec
PL1	-1.00 dB
PL1W	12.17476940 W
SFO1	400.1324710 MHz
F2 - Processing parameters	
SI	32768
SF	400.1300054 MHz
WDW	EM
SSB	0
LB	0.30 Hz
GB	0
PC	1.00

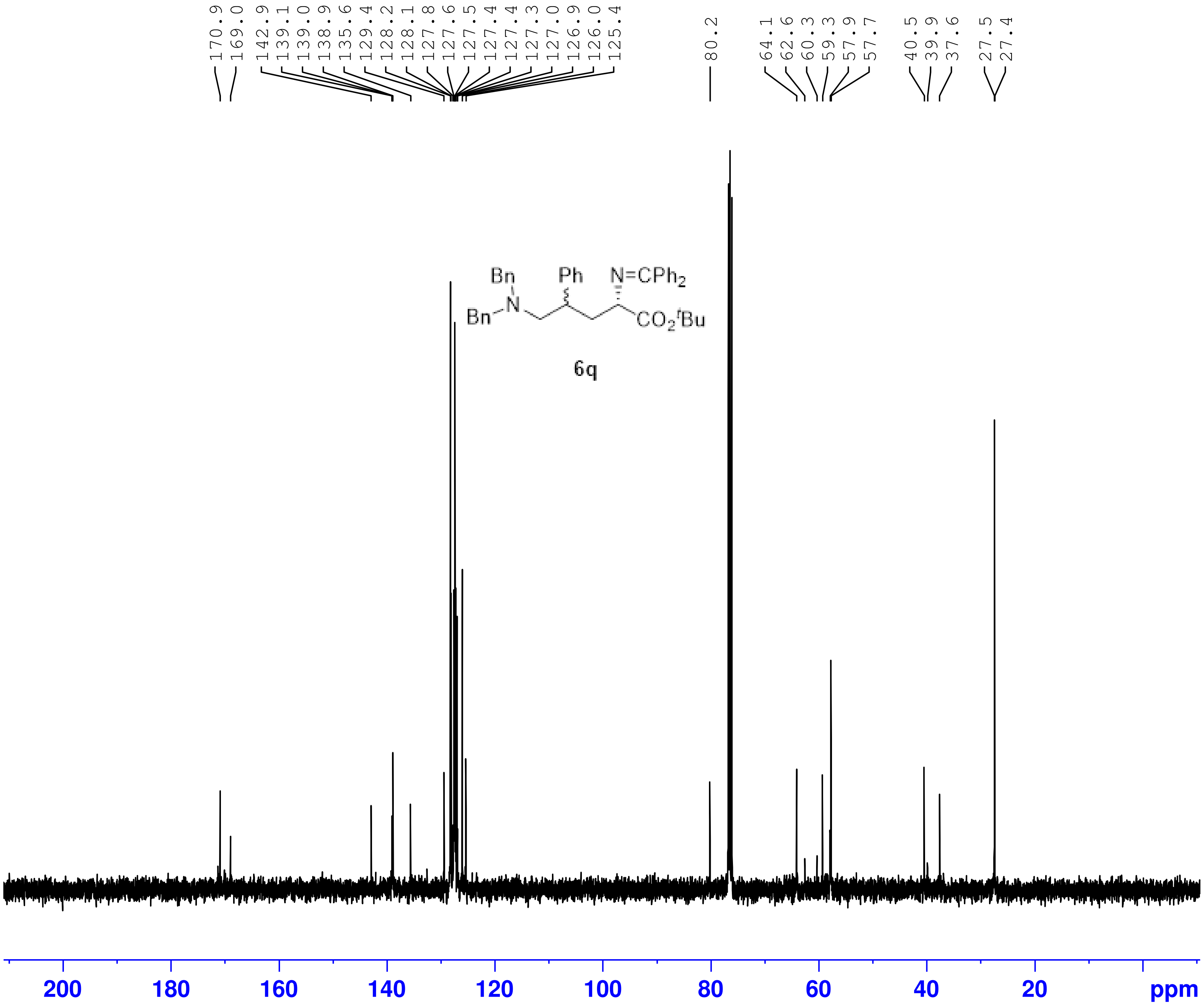

Current Data Parameters
NAME 3q
EXPNO 3
PROCNO 1

F2 - Acquisition Parameters
Date_ 20230119
Time 9.43
INSTRUM spect
PROBHD 5 mm DUL 13C-1
PULPROG zgpg30
TD 65536
SOLVENT CDCl3
NS 515
DS 1
SWH 24038.461 Hz
FIDRES 0.366798 Hz
AQ 1.3631488 sec
RG 2050
DW 20.800 usec
DE 6.00 usec
TE 293.6 K
D1 2.00000000 sec
D11 0.03000000 sec
TD0 1

======== CHANNEL f1 ========
NUC1 13C
P1 40.00 usec
PL1 -3.00 dB
PL1W 60.64365387 W
SFO1 100.6228298 MHz

======== CHANNEL f2 ========
CPDPRG[2 waltz16
NUC2 1H
PCPD2 80.00 usec
PL2 -1.00 dB
PL12 14.39 dB
PL13 18.00 dB
PL2W 12.17476940 W
PL12W 0.35193357 W
PL13W 0.15327126 W
SFO2 400.1316005 MHz

F2 - Processing parameters
SI 32768
SF 100.6128330 MHz
WDW EM
SSB 0
LB 1.00 Hz
GB 0
PC 1.40

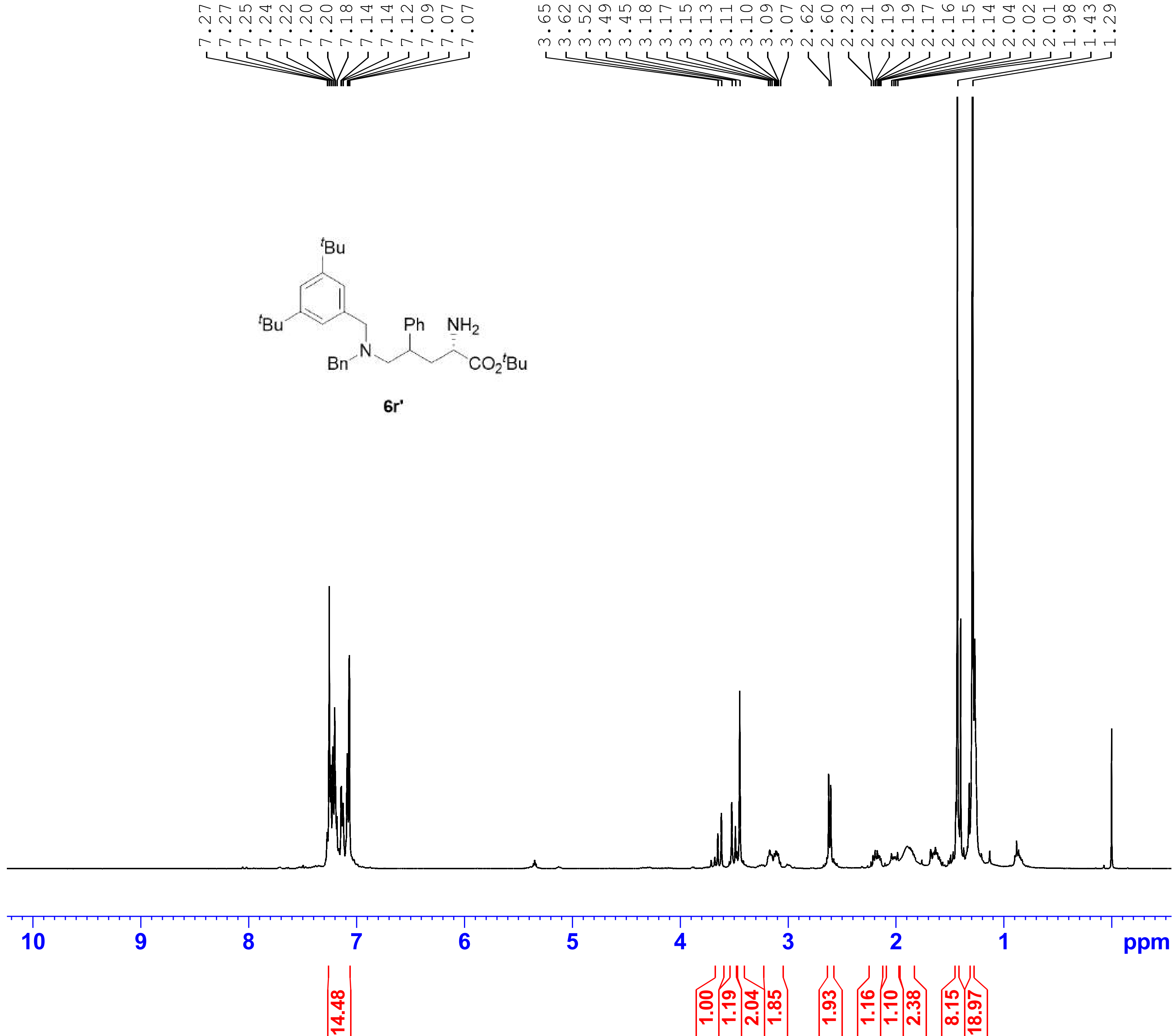

Current Data Parameters
NAME zmh-8-66b-shang-shou
EXPNO 5
PROCNO 1

F2 - Acquisition Parameters
Date_ 20250814
Time 19.32 h
INSTRUM AvanceNeo 400MHz
PROBHD Z163739_0629 (
PULPROG zg30
TD 65536
SOLVENT CDCl3
NS 16
DS 2
SWH 8196.722 Hz
FIDRES 0.250144 Hz
AQ 3.9976959 sec
RG 101
DW 61.000 usec
DE 13.89 usec
TE 297.8 K
D1 1.00000000 sec
TD0 1
SFO1 400.1824711 MHz
NUC1 1H
P0 2.67 usec
P1 8.00 usec
PLW1 21.26700020 W

F2 - Processing parameters
SI 65536
SF 400.1800125 MHz
WDW EM
SSB 0
LB 0.30 Hz
GB 0
PC 1.00

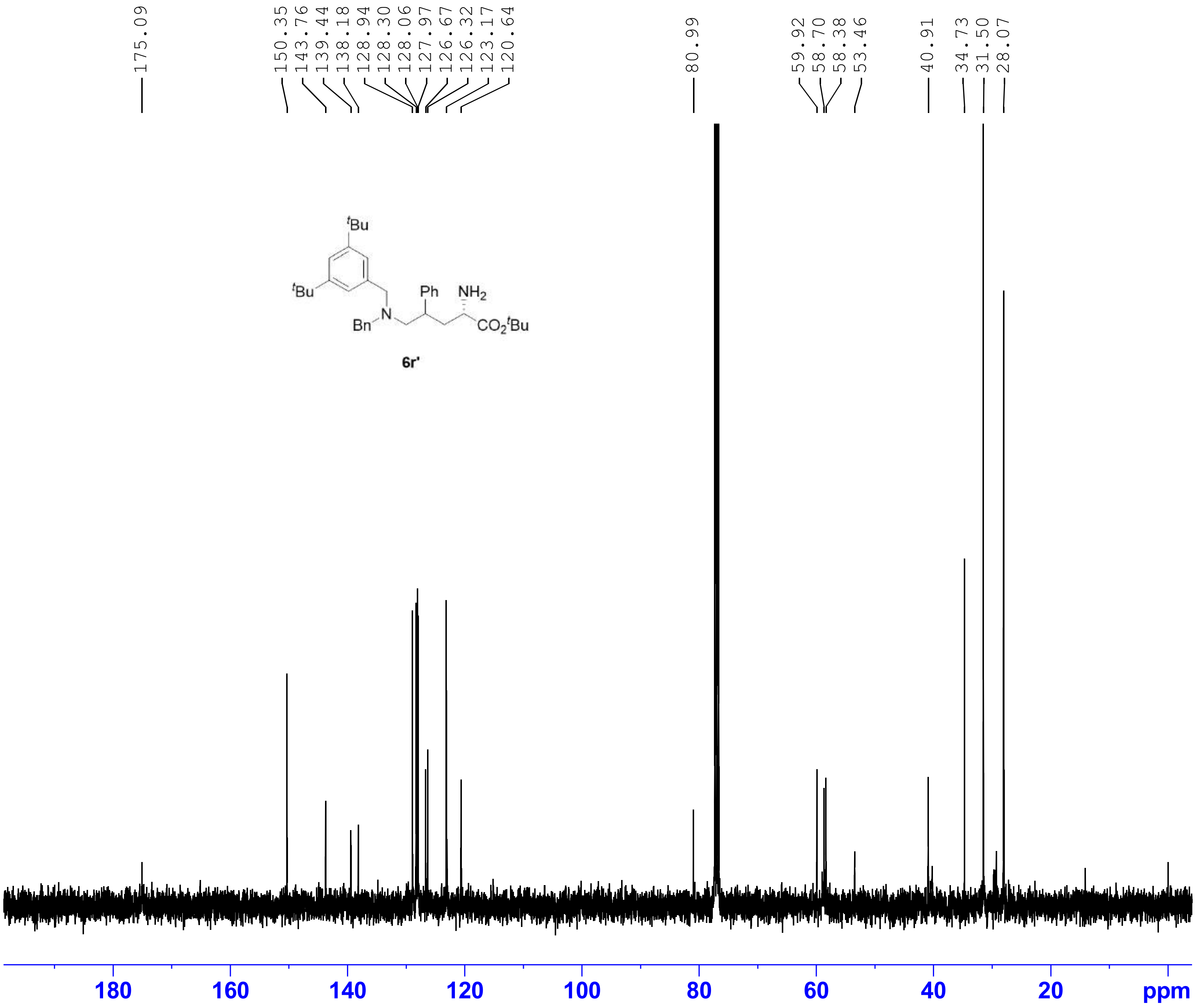

Current Data Parameters
NAME zmh-8-66a-shang-shou
EXPNO 6
PROCNO 1

F2 - Acquisition Parameters
Date_ 20250814
Time 19.45 h
INSTRUM AvanceNeo 400MHz
PROBHD Z163739_0629 (
PULPROG zgpg30
TD 65536
SOLVENT CDCl3
NS 209
DS 4
SWH 23809.523 Hz
FIDRES 0.726609 Hz
AQ 1.3762560 sec
RG 10
DW 21.000 usec
DE 6.50 usec
TE 298.2 K
D1 2.00000000 sec
D11 0.03000000 sec
TD0 1
SFO1 100.6354036 MHz
NUC1 13C
P0 2.67 usec
P1 8.00 usec
PLW1 85.25399780 W
SFO2 400.1816007 MHz
NUC2 1H
CPDPRG[2 waltz65
PCPD2 90.00 usec
PLW2 21.26700020 W
PLW12 0.16802999 W
PLW13 0.08452000 W

F2 - Processing parameters
SI 32768
SF 100.6253445 MHz
WDW EM
SSB 0
LB 1.00 Hz
GB 0
PC 1.40

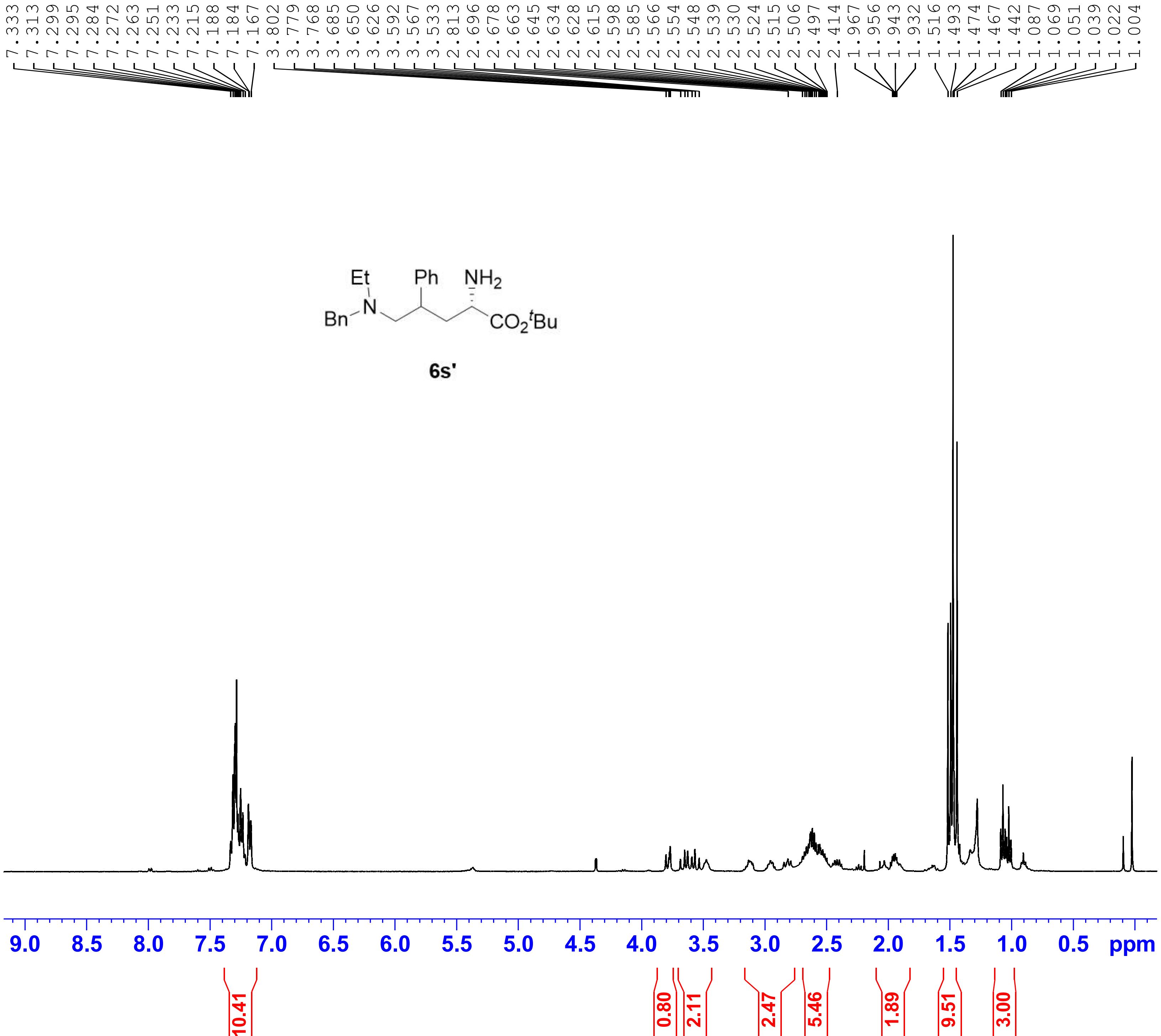

Current Data Parameters
NAME zmh-8-66a-re
EXPNO 1
PROCNO 1

F2 - Acquisition Parameters
Date_ 20250913
Time 20.11 h
INSTRUM AvanceNeo 400MHz
PROBHD Z163739_0629 (
PULPROG zg30
TD 65536
SOLVENT CDCl3
NS 11
DS 2
SWH 8196.722 Hz
FIDRES 0.250144 Hz
AQ 3.9976959 sec
RG 101
DW 61.000 usec
DE 13.89 usec
TE 297.9 K
D1 1.00000000 sec
TD0 1
SFO1 400.1824711 MHz
NUC1 1H
P0 2.67 usec
P1 8.00 usec
PLW1 21.26700020 W

F2 - Processing parameters
SI 65536
SF 400.1800000 MHz
WDW EM
SSB 0
LB 0.30 Hz
GB 0
PC 1.00

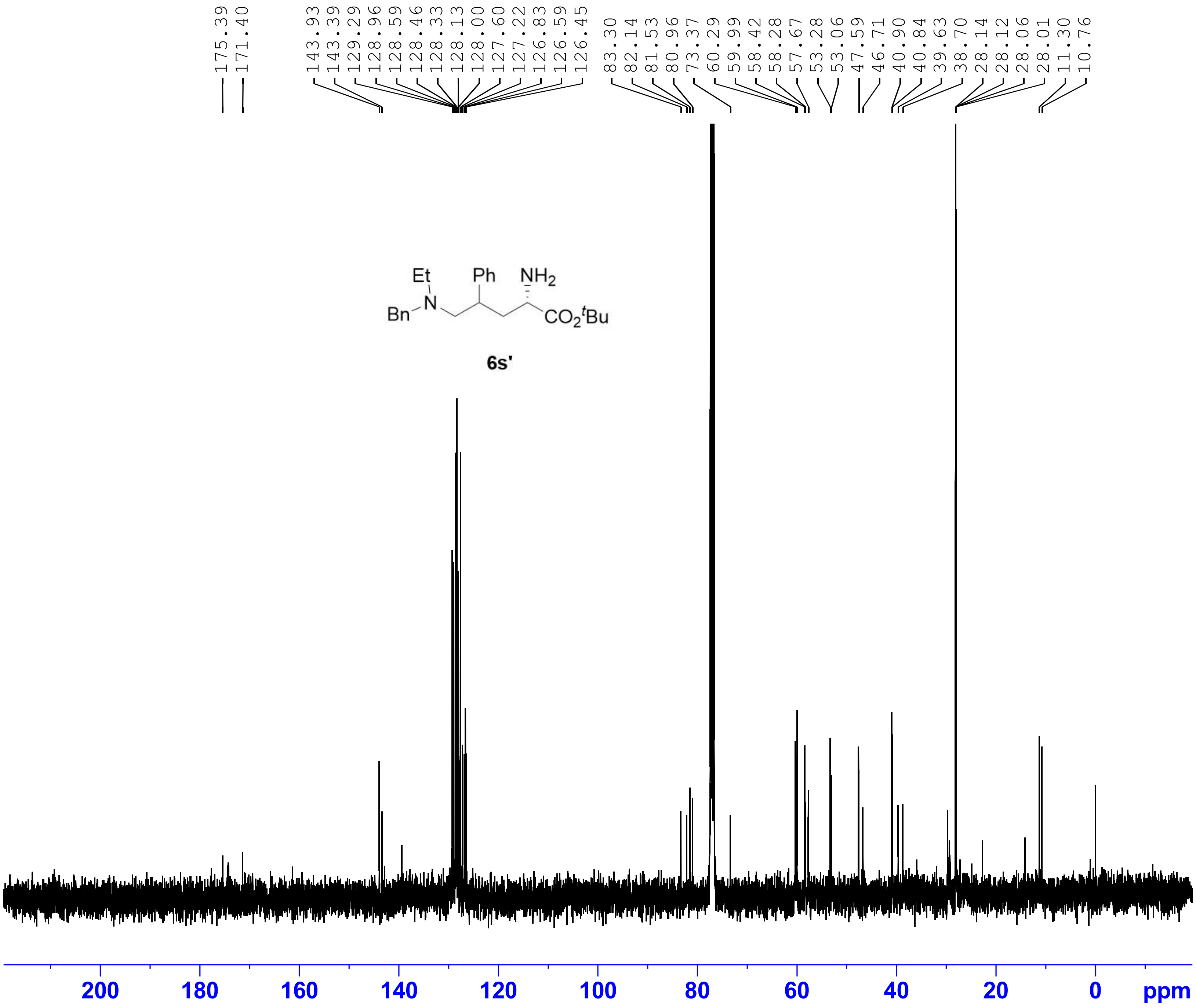

6s'

Current Data Parameters	
NAME	zmh-8-66a-rere
EXPNO	1
PROCNO	1
F2 - Acquisition Parameters	
Date_	20250913
Time	20.38
INSTRUM	spect
PROBHD	5 mm PABBO BB/
PULPROG	zgpg30
TD	65536
SOLVENT	CDCl3
NS	1405
DS	2
SWH	24038.461 Hz
FIDRES	0.366798 Hz
AQ	1.3631488 sec
RG	196.92
DW	20.800 usec
DE	6.50 usec
TE	297.4 K
D1	2.00000000 sec
D11	0.03000000 sec
TD0	1
======== CHANNEL f1 ========	
SFO1	100.6228298 MHz
NUC1	13C
P1	9.70 usec
PLW1	46.98899841 W
======== CHANNEL f2 ========	
SFO2	400.1316005 MHz
NUC2	1H
CPDPRG[2	waltz16
PCPD2	90.00 usec
PLW2	11.99499989 W
PLW12	0.34213999 W
PLW13	0.27713001 W
F2 - Processing parameters	
SI	32768
SF	100.6127690 MHz
WDW	EM
SSB	0
LB	1.00 Hz
GB	0
PC	1.40

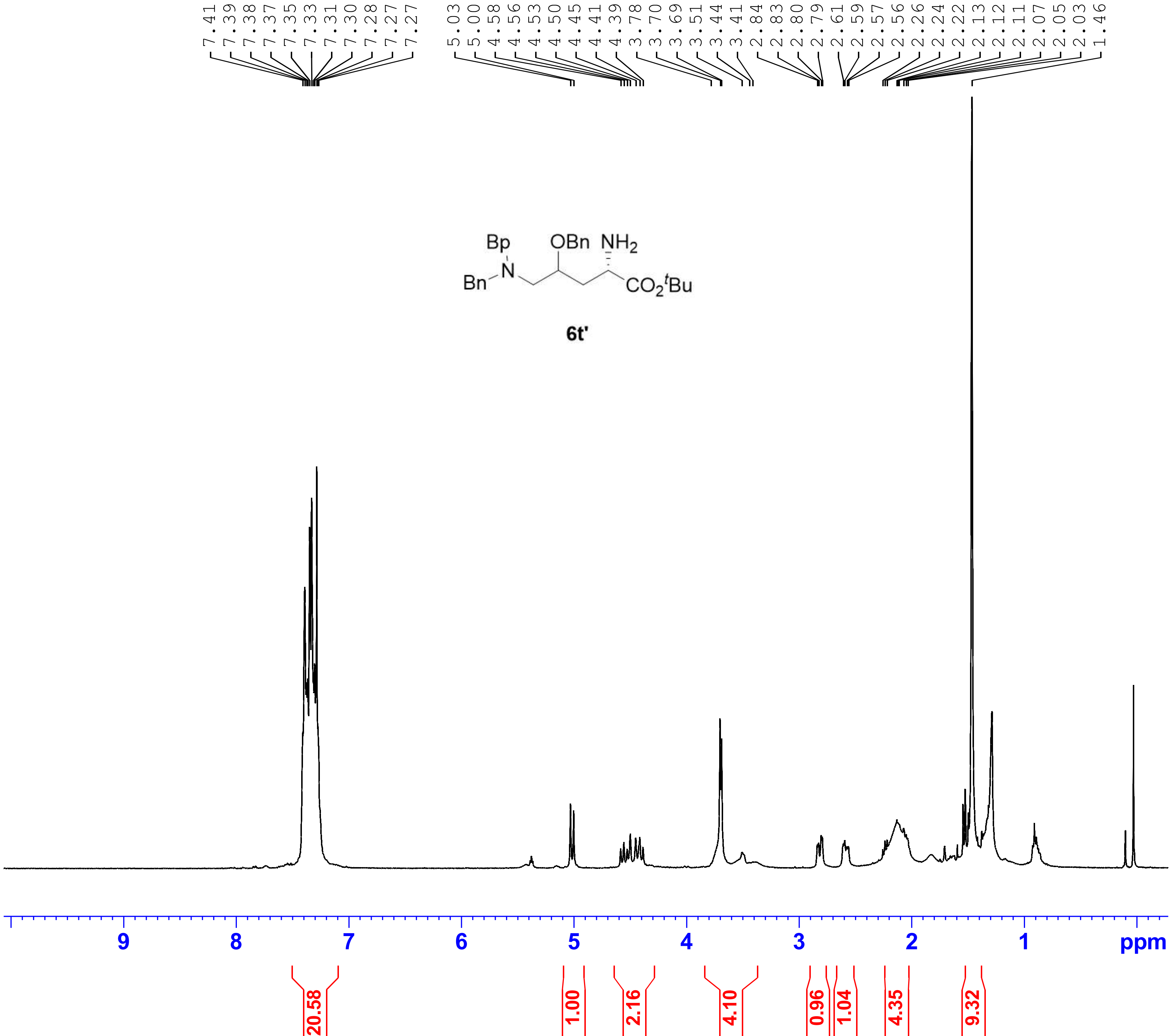

6t'

Current Data Parameters
NAME zmh-8-66c-shou
EXPNO 7
PROCNO 1

F2 - Acquisition Parameters
Date_ 20250814
Time 19.50 h
INSTRUM AvanceNeo 400MHz
PROBHD Z163739_0629 (
PULPROG zg30
TD 65536
SOLVENT CDCl3
NS 16
DS 2
SWH 8196.722 Hz
FIDRES 0.250144 Hz
AQ 3.9976959 sec
RG 101
DW 61.000 usec
DE 13.89 usec
TE 297.7 K
D1 1.00000000 sec
TD0 1
SFO1 400.1824711 MHz
NUC1 1H
P0 2.67 usec
P1 8.00 usec
PLW1 21.26700020 W

F2 - Processing parameters
SI 65536
SF 400.1800000 MHz
WDW EM
SSB 0
LB 0.30 Hz
GB 0
PC 1.00

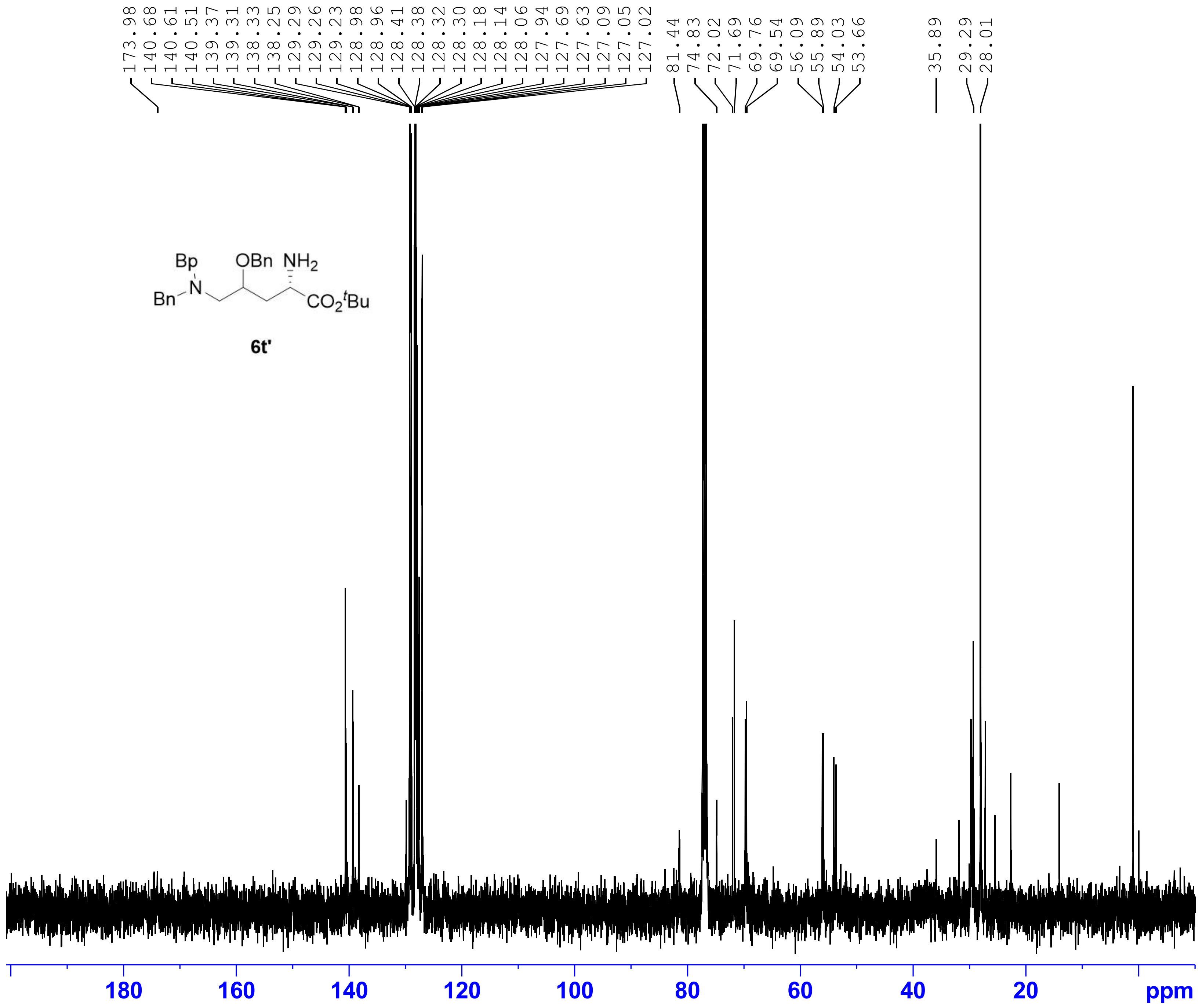

Current Data Parameters
NAME zmh-8-66c---
EXPNO 2
PROCNO 1

F2 - Acquisition Parameters
Date_ 20250816
Time 18.50 h
INSTRUM AvanceNeo 400MHz
PROBHD Z163739_0629 (
PULPROG zgpg30
TD 65536
SOLVENT CDCl3
NS 635
DS 4
SWH 23809.523 Hz
FIDRES 0.726609 Hz
AQ 1.3762560 sec
RG 10
DW 21.000 usec
DE 6.50 usec
TE 301.0 K
D1 2.00000000 sec
D11 0.03000000 sec
TD0 1
SFO1 100.6354036 MHz
NUC1 13C
P0 2.67 usec
P1 8.00 usec
PLW1 85.25399780 W
SFO2 400.1816007 MHz
NUC2 1H
CPDPRG[2 waltz65
PCPD2 90.00 usec
PLW2 21.26700020 W
PLW12 0.16802999 W
PLW13 0.08452000 W

F2 - Processing parameters
SI 32768
SF 100.6253454 MHz
WDW EM
SSB 0
LB 1.00 Hz
GB 0
PC 1.40

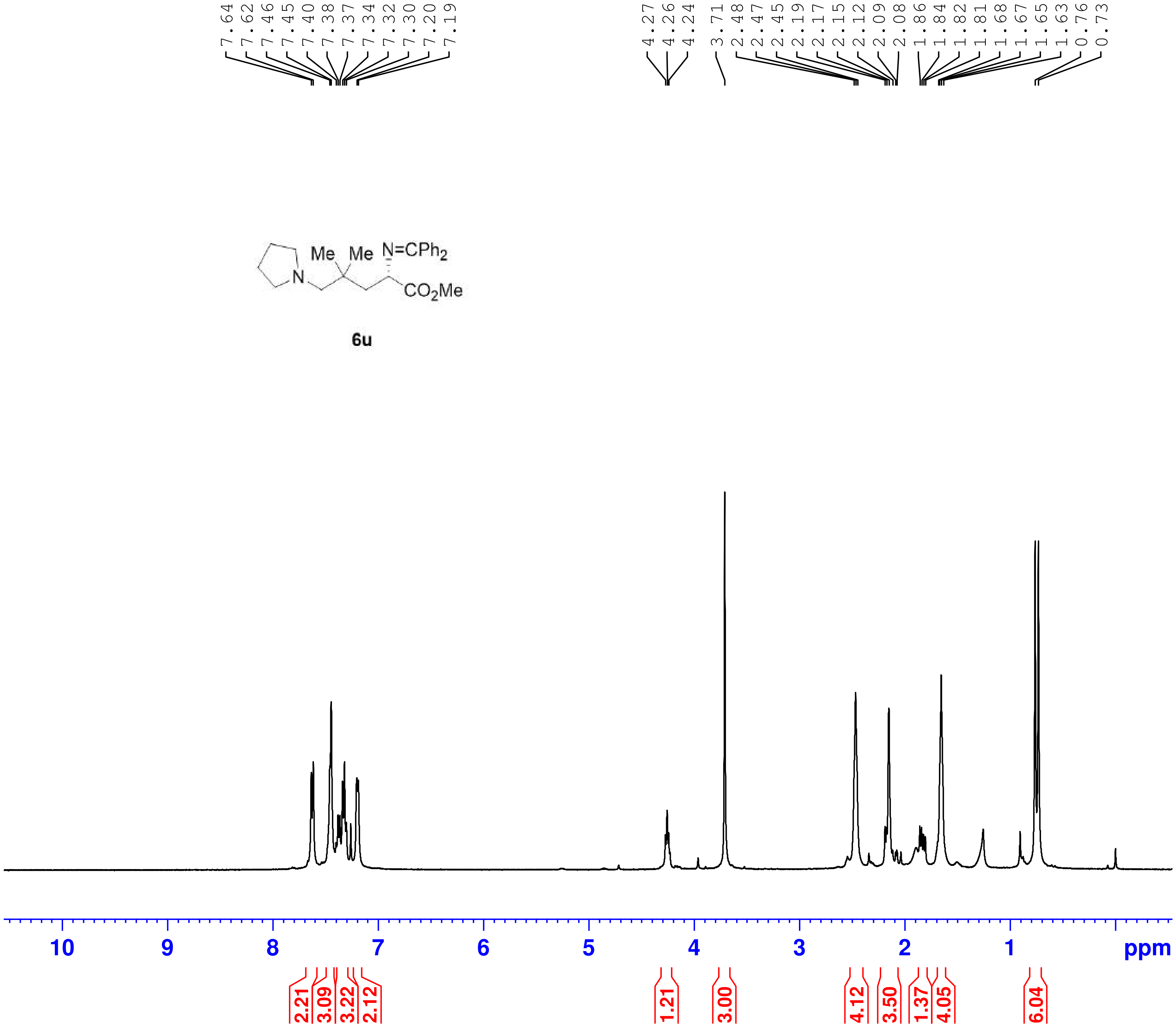

6u

Current Data Parameters
NAME zmh-2-116a-chun-
EXPNO 1
PROCNO 1

F2 - Acquisition Parameters
Date_ 20230113
Time 19.00
INSTRUM spect
PROBHD 5 mm PABBO BB/
PULPROG zg30
TD 65536
SOLVENT CDCl3
NS 16
DS 2
SWH 8012.820 Hz
FIDRES 0.122266 Hz
AQ 4.0894465 sec
RG 39.46
DW 62.400 usec
DE 6.50 usec
TE 296.2 K
D1 1.00000000 sec
TD0 1

======== CHANNEL f1 ========
SFO1 400.1324710 MHz
NUC1 1H
P1 14.50 usec
PLW1 11.99499989 W

F2 - Processing parameters
SI 65536
SF 400.1300097 MHz
WDW EM
SSB 0
LB 0.30 Hz
GB 0
PC 1.00

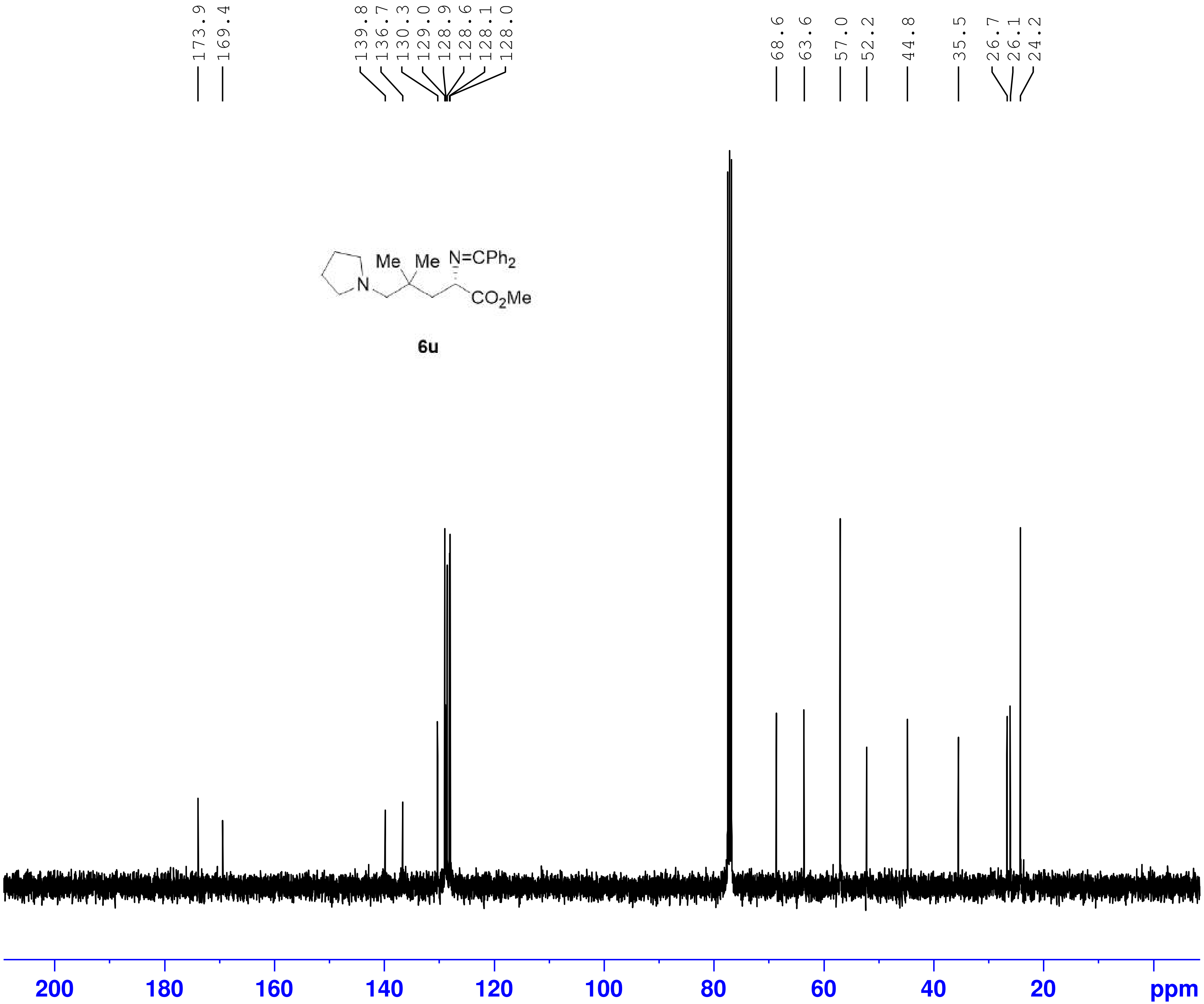

6u

```
Current Data Parameters
NAME        zmh-2-116a-chun
EXPNO                     2
PROCNO                    1

F2 - Acquisition Parameters
Date_              20230113
Time                  18.50 h
INSTRUM   AvanceNeo 400MHz
PROBHD    Z163739_0629 (
PULPROG             zgpg30
TD                    65536
SOLVENT               CDCl3
NS                      223
DS                        4
SWH               23809.523 Hz
FIDRES             0.726609 Hz
AQ                1.3762560 sec
RG                     11.3
DW                   21.000 usec
DE                     6.50 usec
TE                    300.1 K
D1               2.00000000 sec
D11              0.03000000 sec
TD0                       1
SFO1            100.6354036 MHz
NUC1                    13C
P0                     2.67 usec
P1                     8.00 usec
PLW1            85.25399780 W
SFO2            400.1816007 MHz
NUC2                     1H
CPDPRG[2            waltz65
PCPD2                 90.00 usec
PLW2            21.26700020 W
PLW12            0.16802999 W
PLW13            0.08452000 W

F2 - Processing parameters
SI                    32768
SF              100.6253288 MHz
WDW                      EM
SSB                       0
LB                     1.00 Hz
GB                        0
PC                     1.40
```

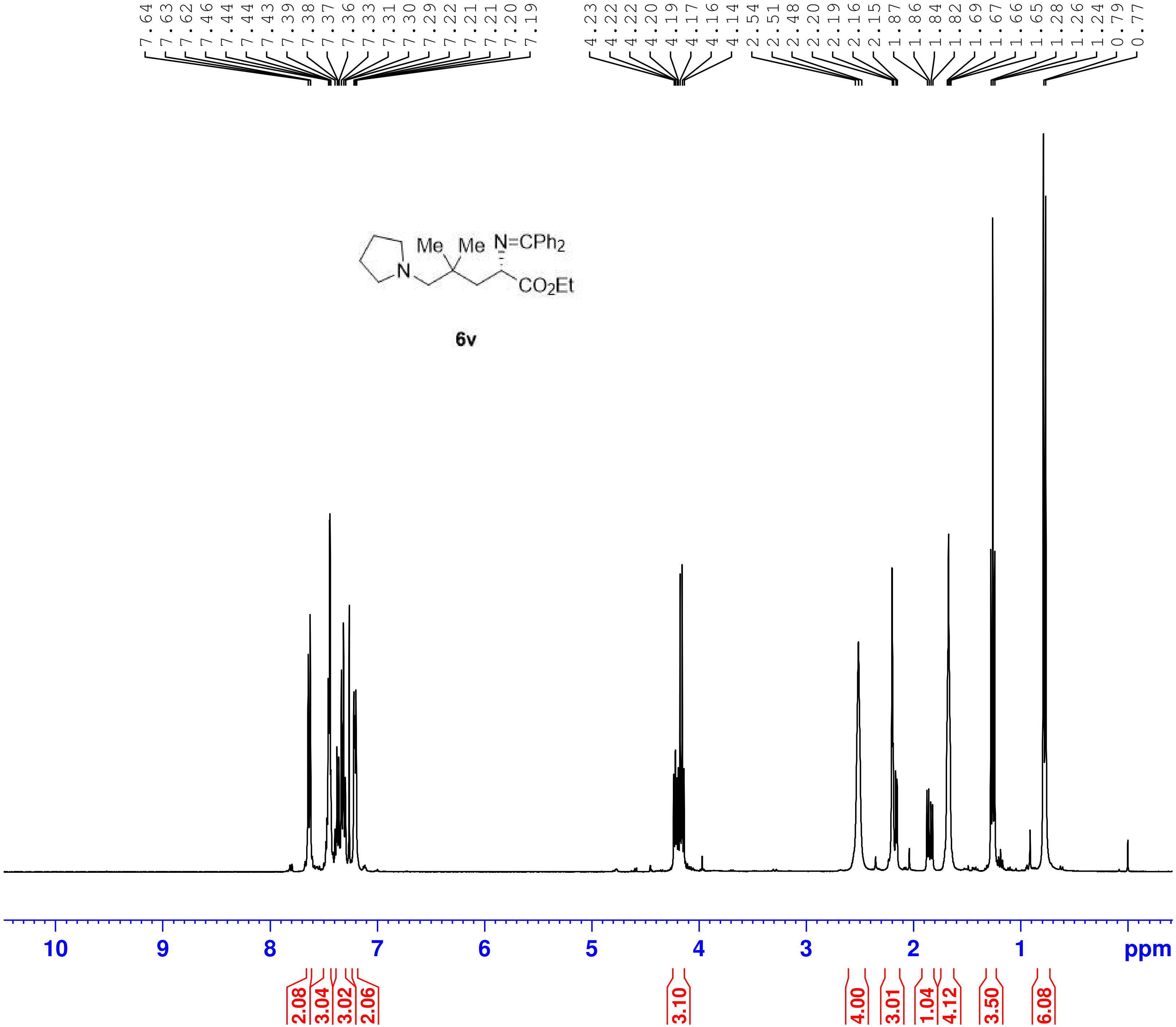

6v

Current Data Parameters
NAME 3s
EXPNO 1
PROCNO 1

F2 - Acquisition Parameters
Date_ 20221217
Time 9.07
INSTRUM spect
PROBHD 5 mm DUL 13C-1
PULPROG zg30
TD 65536
SOLVENT CDCl3
NS 16
DS 2
SWH 8223.685 Hz
FIDRES 0.125483 Hz
AQ 3.9845889 sec
RG 114
DW 60.800 usec
DE 6.00 usec
TE 294.6 K
D1 1.00000000 sec
TD0 1

======== CHANNEL f1 ========
NUC1 1H
P1 15.80 usec
PL1 -1.00 dB
PL1W 12.17476940 W
SFO1 400.1324710 MHz

F2 - Processing parameters
SI 32768
SF 400.1300093 MHz
WDW EM
SSB 0
LB 0.30 Hz
GB 0
PC 1.00

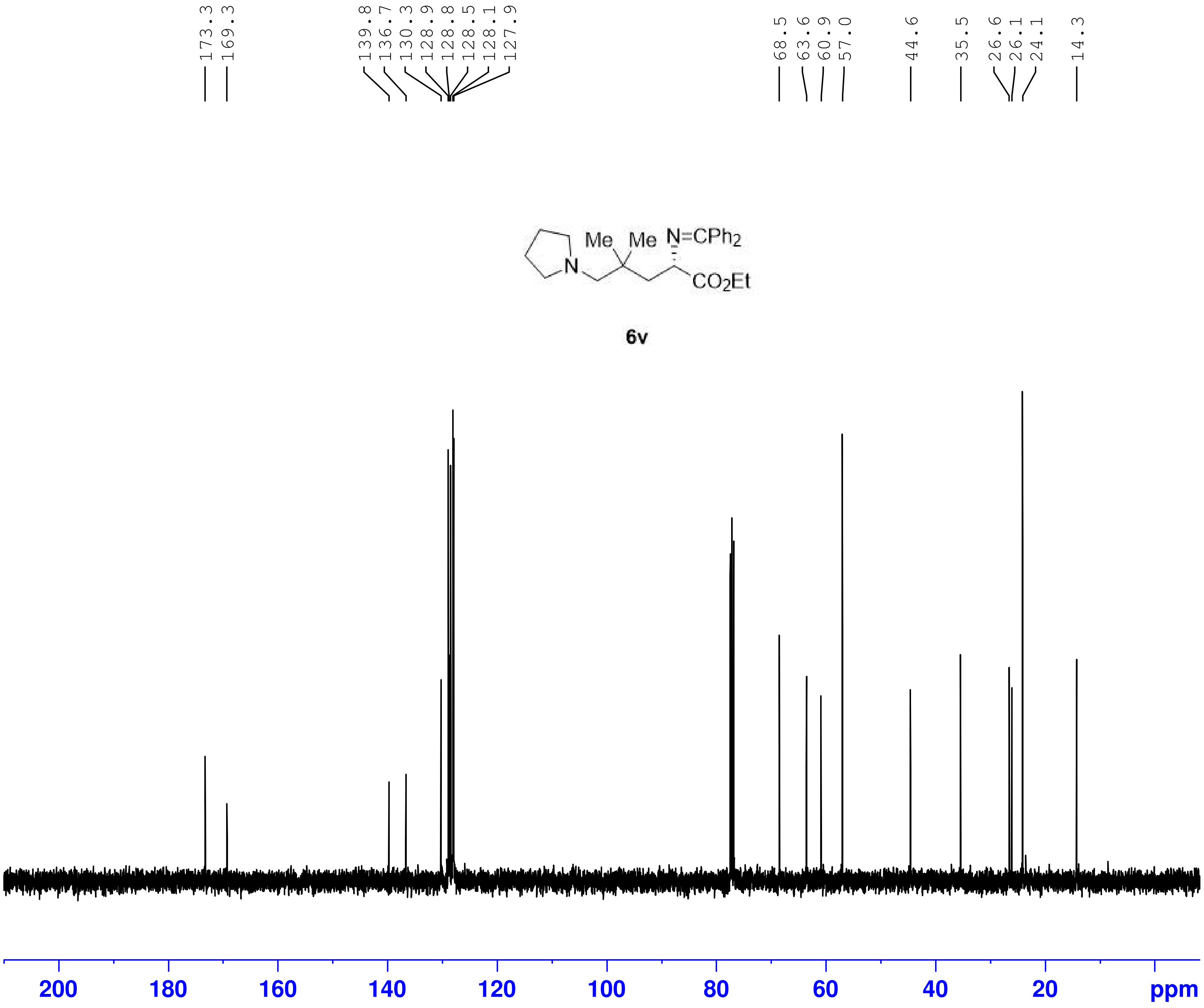

Current Data Parameters

NAME	zmh-2-64a-chun-1217-C
EXPNO	1
PROCNO	1

F2 - Acquisition Parameters

Date_	20221217
Time	9.11
INSTRUM	spect
PROBHD	5 mm DUL 13C-1
PULPROG	zgpg30
TD	65536
SOLVENT	CDCl3
NS	84
DS	1
SWH	24038.461 Hz
FIDRES	0.366798 Hz
AQ	1.3631488 sec
RG	2050
DW	20.800 usec
DE	6.00 usec
TE	294.7 K
D1	2.00000000 sec
D11	0.03000000 sec
TD0	1

======== CHANNEL f1 ========

NUC1	13C
P1	40.00 usec
PL1	-3.00 dB
PL1W	60.64365387 W
SFO1	100.6228298 MHz

======== CHANNEL f2 ========

CPDPRG[2	waltz16
NUC2	1H
PCPD2	80.00 usec
PL2	-1.00 dB
PL12	14.39 dB
PL13	18.00 dB
PL2W	12.17476940 W
PL12W	0.35193357 W
PL13W	0.15327126 W
SFO2	400.1316005 MHz

F2 - Processing parameters

SI	32768
SF	100.6127631 MHz
WDW	EM
SSB	0
LB	1.00 Hz
GB	0
PC	1.40

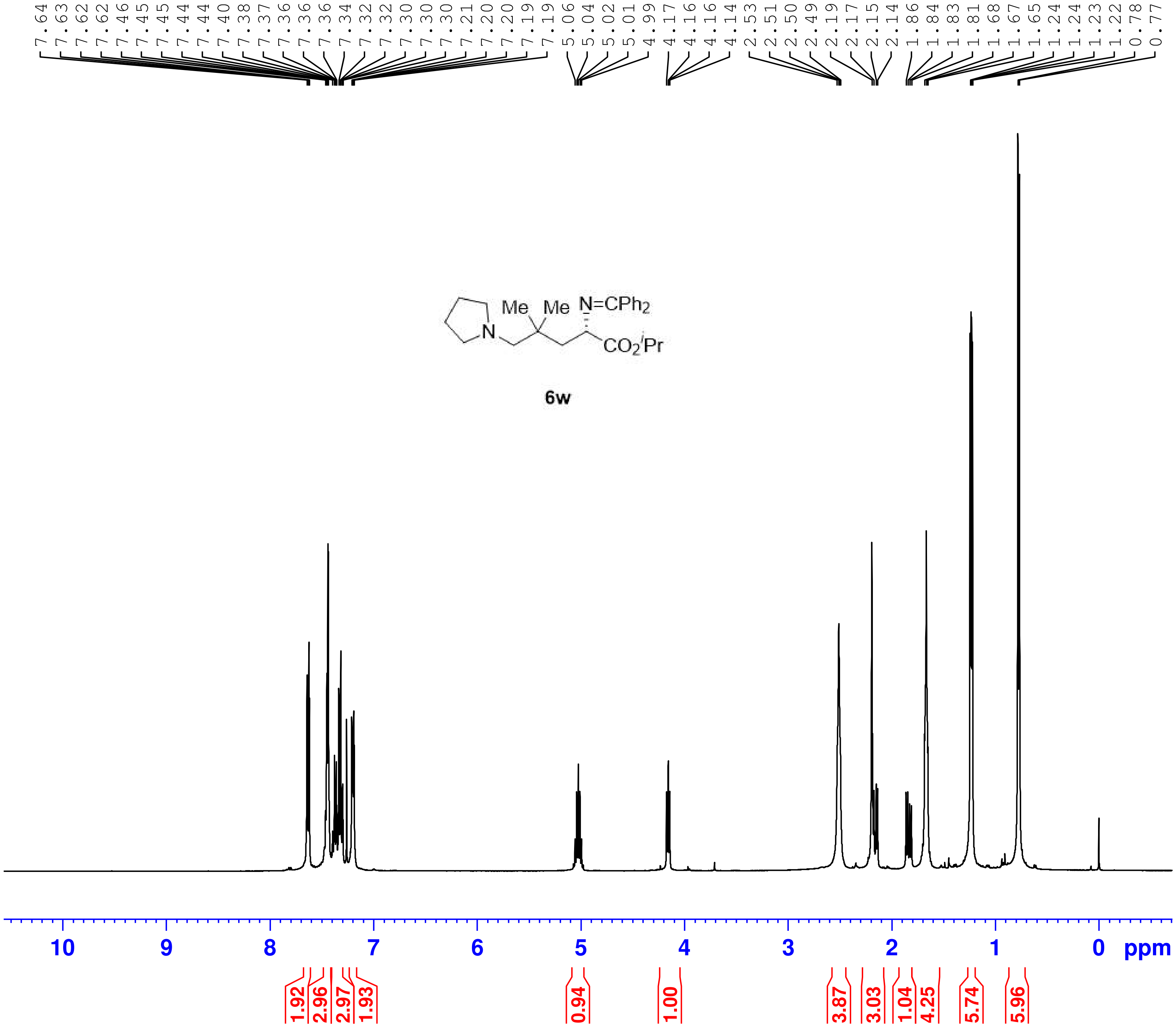

Current Data Parameters
NAME 3t
EXPNO 1
PROCNO 1

F2 - Acquisition Parameters
Date_ 20221216
Time 18.22
INSTRUM spect
PROBHD 5 mm PABBO BB/
PULPROG zg30
TD 65536
SOLVENT CDCl3
NS 8
DS 2
SWH 8012.820 Hz
FIDRES 0.122266 Hz
AQ 4.0894465 sec
RG 27.78
DW 62.400 usec
DE 6.50 usec
TE 294.5 K
D1 1.00000000 sec
TD0 1

======== CHANNEL f1 ========
SFO1 400.1324710 MHz
NUC1 1H
P1 14.50 usec
PLW1 11.99499989 W

F2 - Processing parameters
SI 65536
SF 400.1300098 MHz
WDW EM
SSB 0
LB 0.30 Hz
GB 0
PC 1.00

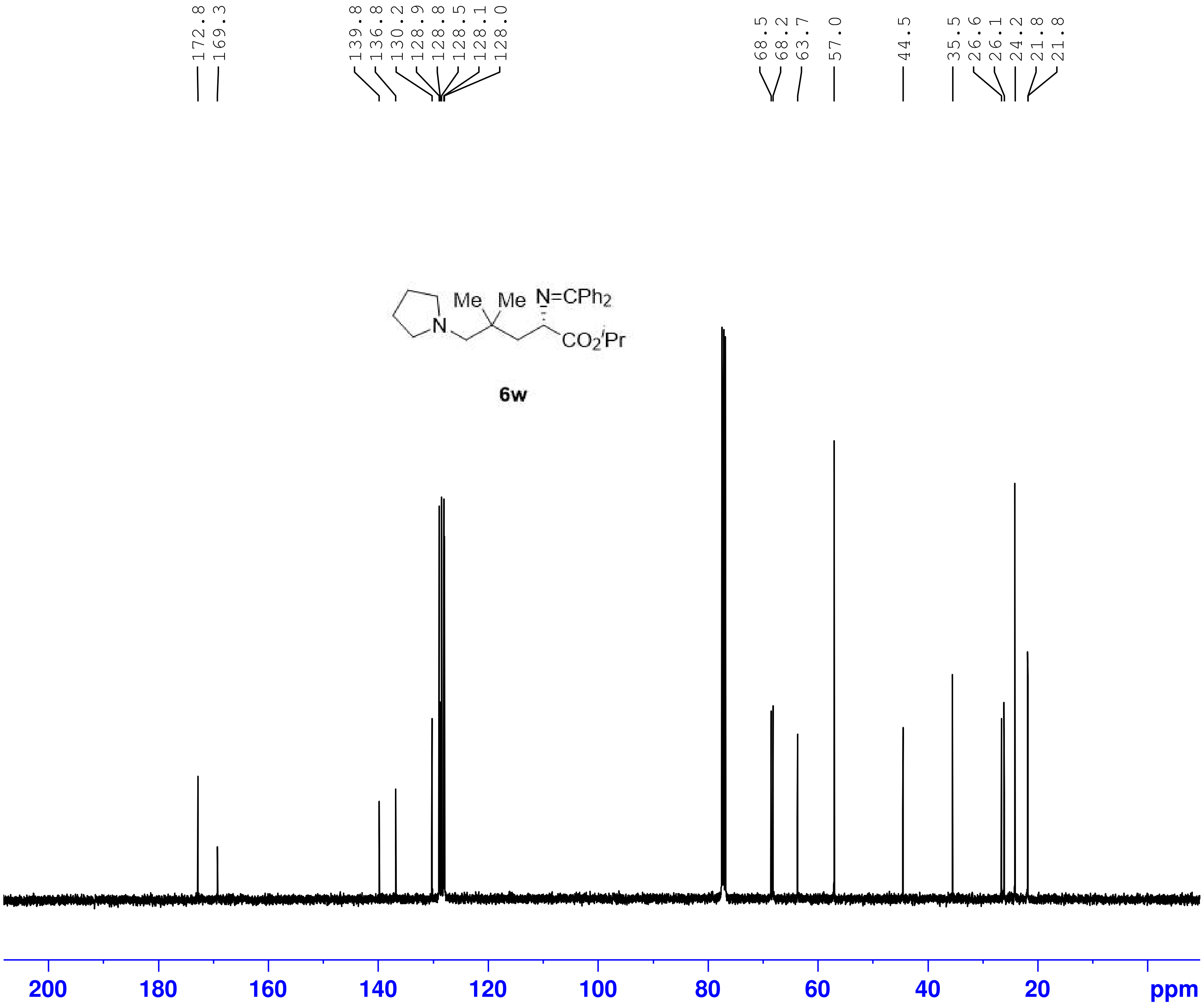

6w

Current Data Parameters
NAME 3t
EXPNO 2
PROCNO 1

F2 - Acquisition Parameters
Date_ 20221216
Time 18.36
INSTRUM spect
PROBHD 5 mm PABBO BB/
PULPROG zgpg30
TD 65536
SOLVENT CDCl3
NS 230
DS 2
SWH 24038.461 Hz
FIDRES 0.366798 Hz
AQ 1.3631488 sec
RG 196.92
DW 20.800 usec
DE 6.50 usec
TE 295.4 K
D1 2.00000000 sec
D11 0.03000000 sec
TD0 1

======== CHANNEL f1 ========
SFO1 100.6228298 MHz
NUC1 13C
P1 9.70 usec
PLW1 46.98899841 W

======== CHANNEL f2 ========
SFO2 400.1316005 MHz
NUC2 1H
CPDPRG[2 waltz16
PCPD2 90.00 usec
PLW2 11.99499989 W
PLW12 0.34213999 W
PLW13 0.27713001 W

F2 - Processing parameters
SI 32768
SF 100.6127593 MHz
WDW EM
SSB 0
LB 1.00 Hz
GB 0
PC 1.40

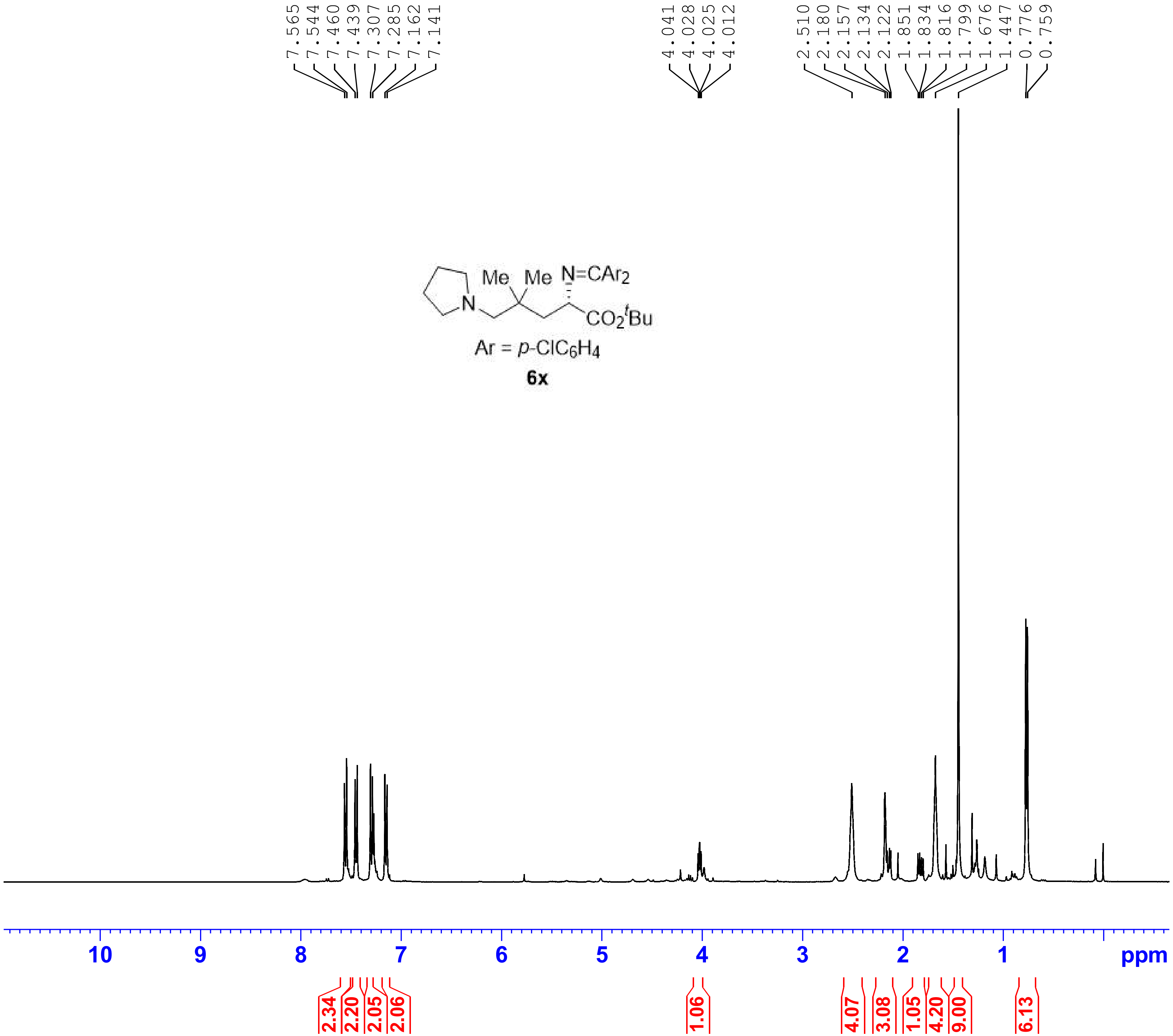

Current Data Parameters
NAME zmh-cl-p
EXPNO 1
PROCNO 1

F2 - Acquisition Parameters
Date_ 20250927
Time 19.01
INSTRUM spect
PROBHD 5 mm DUL 13C-1
PULPROG zg30
TD 65536
SOLVENT CDCl3
NS 16
DS 2
SWH 8223.685 Hz
FIDRES 0.125483 Hz
AQ 3.9845889 sec
RG 144
DW 60.800 usec
DE 6.00 usec
TE 294.0 K
D1 1.00000000 sec
TD0 1

======== CHANNEL f1 ========
NUC1 1H
P1 15.80 usec
PL1 -1.00 dB
PL1W 12.17476940 W
SFO1 400.1324710 MHz

F2 - Processing parameters
SI 32768
SF 400.1300054 MHz
WDW EM
SSB 0
LB 0.30 Hz
GB 0
PC 1.00

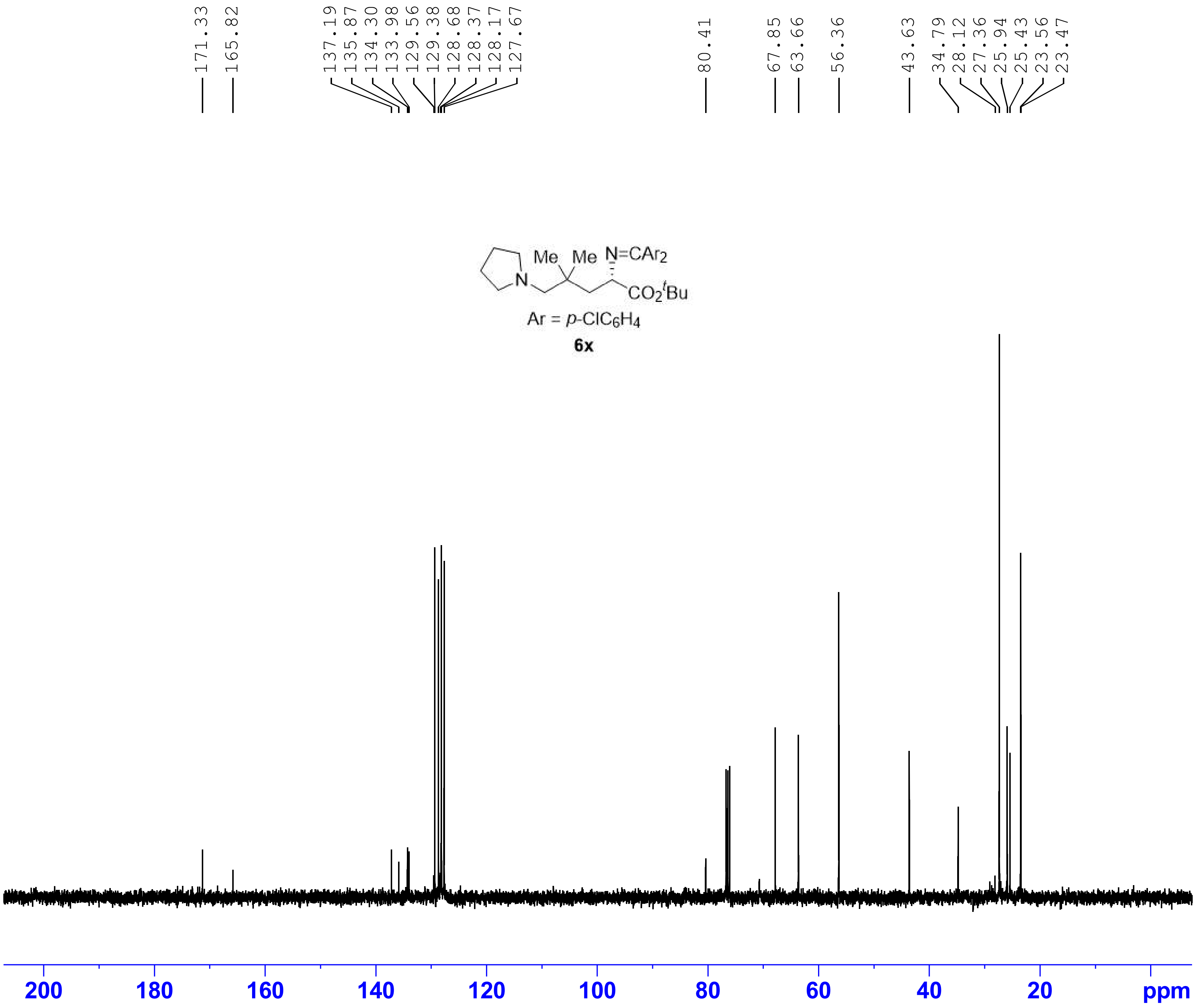

Ar = p-ClC$_6$H$_4$

6x

Current Data Parameters
NAME zmh-cl-p
EXPNO 2
PROCNO 1

F2 - Acquisition Parameters
Date_ 20250927
Time 19.03
INSTRUM spect
PROBHD 5 mm DUL 13C-1
PULPROG zgpg30
TD 65536
SOLVENT CDCl3
NS 60
DS 1
SWH 24038.461 Hz
FIDRES 0.366798 Hz
AQ 1.3631488 sec
RG 45.2
DW 20.800 usec
DE 6.00 usec
TE 294.1 K
D1 2.00000000 sec
D11 0.03000000 sec
TD0 1

======== CHANNEL f1 ========
NUC1 13C
P1 40.00 usec
PL1 -3.00 dB
PL1W 60.64365387 W
SFO1 100.6228298 MHz

======== CHANNEL f2 ========
CPDPRG[2 waltz16
NUC2 1H
PCPD2 80.00 usec
PL2 -1.00 dB
PL12 14.39 dB
PL13 18.00 dB
PL2W 12.17476940 W
PL12W 0.35193357 W
PL13W 0.15327126 W
SFO2 400.1316005 MHz

F2 - Processing parameters
SI 32768
SF 100.6128330 MHz
WDW EM
SSB 0
LB 1.00 Hz
GB 0
PC 1.40

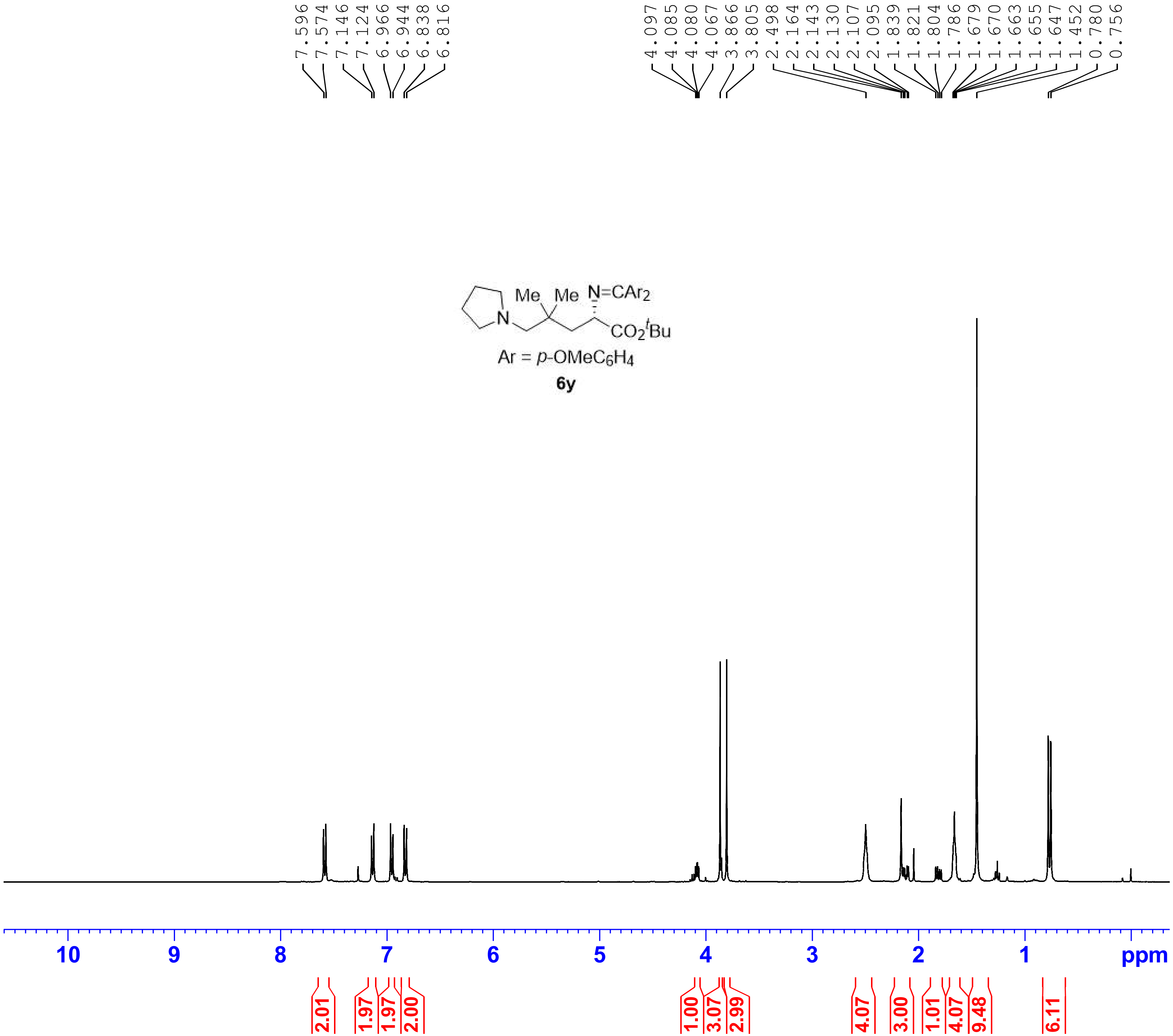

Ar = p-OMeC$_6$H$_4$

6y

Current Data Parameters
NAME zmh-ome-p
EXPNO 1
PROCNO 1

F2 - Acquisition Parameters
Date_ 20250928
Time 17.58
INSTRUM spect
PROBHD 5 mm DUL 13C-1
PULPROG zg30
TD 65536
SOLVENT CDCl3
NS 14
DS 2
SWH 8223.685 Hz
FIDRES 0.125483 Hz
AQ 3.9845889 sec
RG 64
DW 60.800 usec
DE 6.00 usec
TE 293.9 K
D1 1.00000000 sec
TD0 1

======== CHANNEL f1 ========
NUC1 1H
P1 15.80 usec
PL1 -1.00 dB
PL1W 12.17476940 W
SFO1 400.1324710 MHz

F2 - Processing parameters
SI 32768
SF 400.1300054 MHz
WDW EM
SSB 0
LB 0.30 Hz
GB 0
PC 1.00

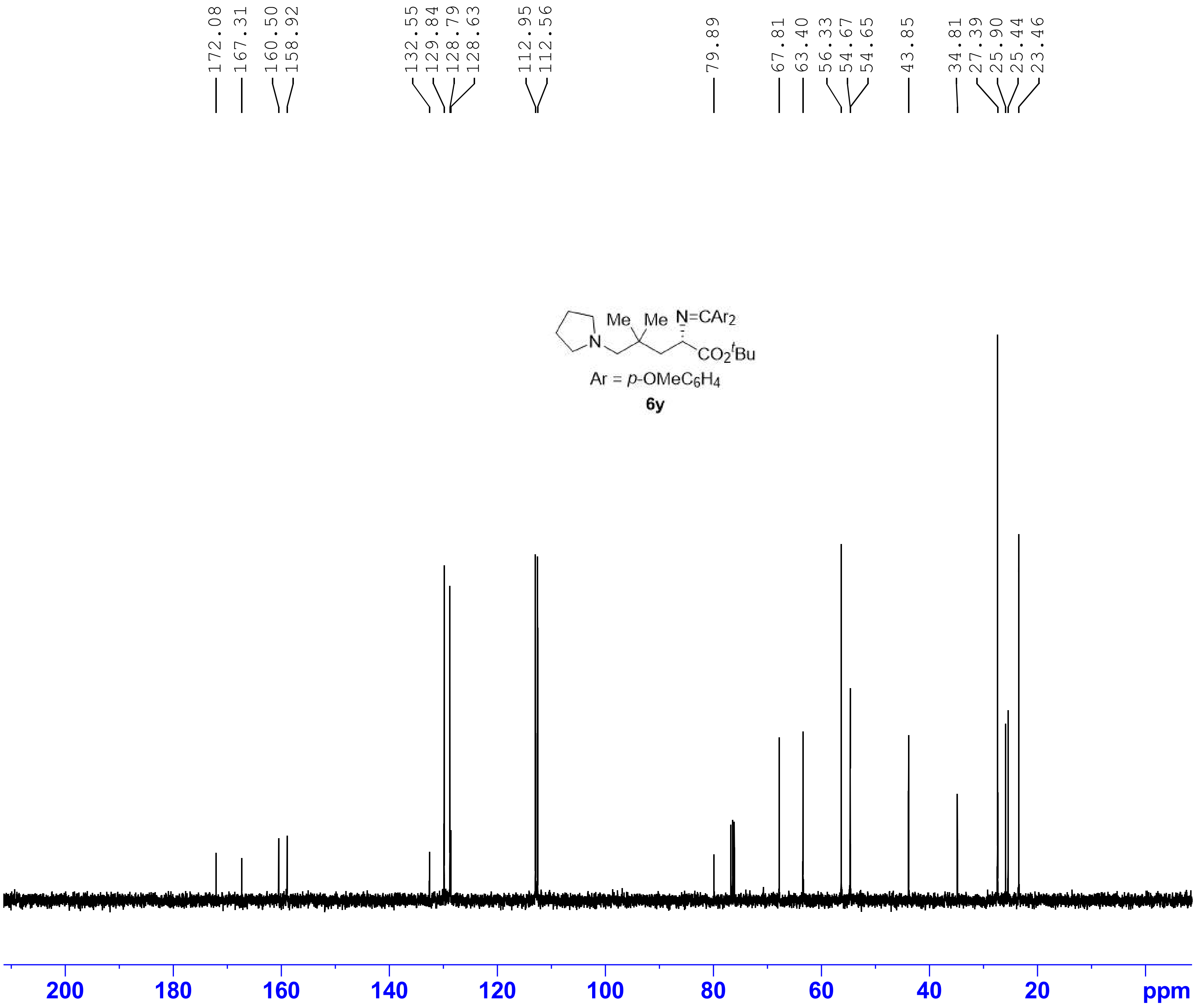


```
Current Data Parameters
NAME             zmh-ome-p
EXPNO                    2
PROCNO                   1

F2 - Acquisition Parameters
Date_             20250928
Time                 18.01
INSTRUM              spect
PROBHD   5 mm DUL 13C-1
PULPROG            zgpg30
TD                   65536
SOLVENT              CDCl3
NS                      27
DS                       1
SWH              24038.461 Hz
FIDRES            0.366798 Hz
AQ               1.3631488 sec
RG                    45.2
DW                  20.800 usec
DE                    6.00 usec
TE                   294.1 K
D1              2.00000000 sec
D11             0.03000000 sec
TD0                      1

======== CHANNEL f1 ========
NUC1                   13C
P1                   40.00 usec
PL1                  -3.00 dB
PL1W           60.64365387 W
SFO1           100.6228298 MHz

======== CHANNEL f2 ========
CPDPRG[2           waltz16
NUC2                    1H
PCPD2                80.00 usec
PL2                  -1.00 dB
PL12                 14.39 dB
PL13                 18.00 dB
PL2W           12.17476940 W
PL12W           0.35193357 W
PL13W           0.15327126 W
SFO2           400.1316005 MHz

F2 - Processing parameters
SI                   32768
SF             100.6128330 MHz
WDW                     EM
SSB                      0
LB                    1.00 Hz
GB                       0
PC                    1.40
```

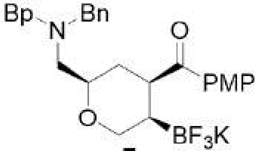

7

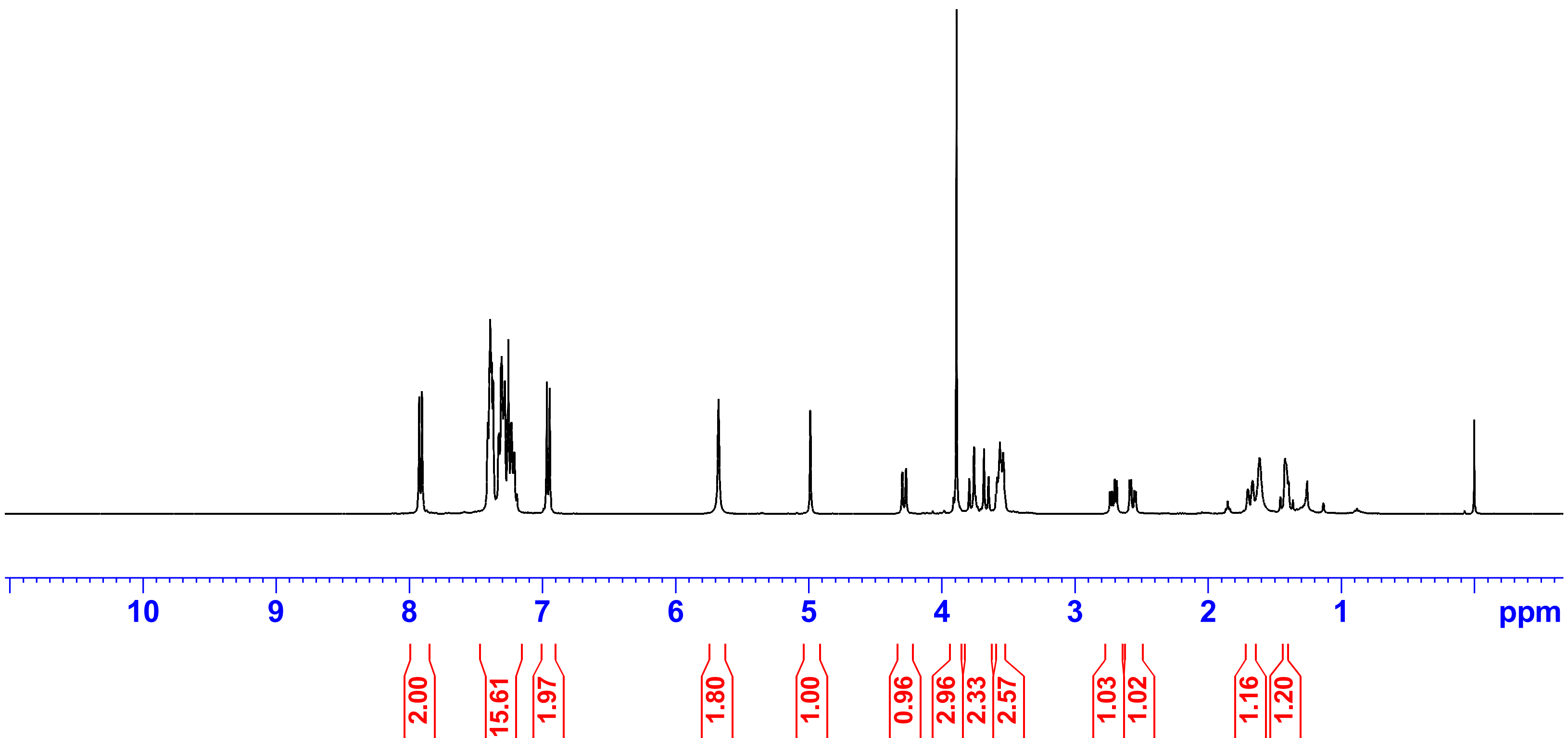

Current Data Parameters
NAME zmh-bf3k-chiral
EXPNO 1
PROCNO 1

F2 - Acquisition Parameters
Date_ 20240906
Time 11.20 h
INSTRUM AvanceNeo 400MHz
PROBHD Z163739_0629 (
PULPROG zg30
TD 65536
SOLVENT CDCl3
NS 8
DS 2
SWH 8196.722 Hz
FIDRES 0.250144 Hz
AQ 3.9976959 sec
RG 101
DW 61.000 usec
DE 13.89 usec
TE 296.8 K
D1 1.00000000 sec
TD0 1
SFO1 400.1824711 MHz
NUC1 1H
P0 2.67 usec
P1 8.00 usec
PLW1 21.26700020 W

F2 - Processing parameters
SI 65536
SF 400.1800114 MHz
WDW EM
SSB 0
LB 0.60 Hz
GB 0
PC 1.00

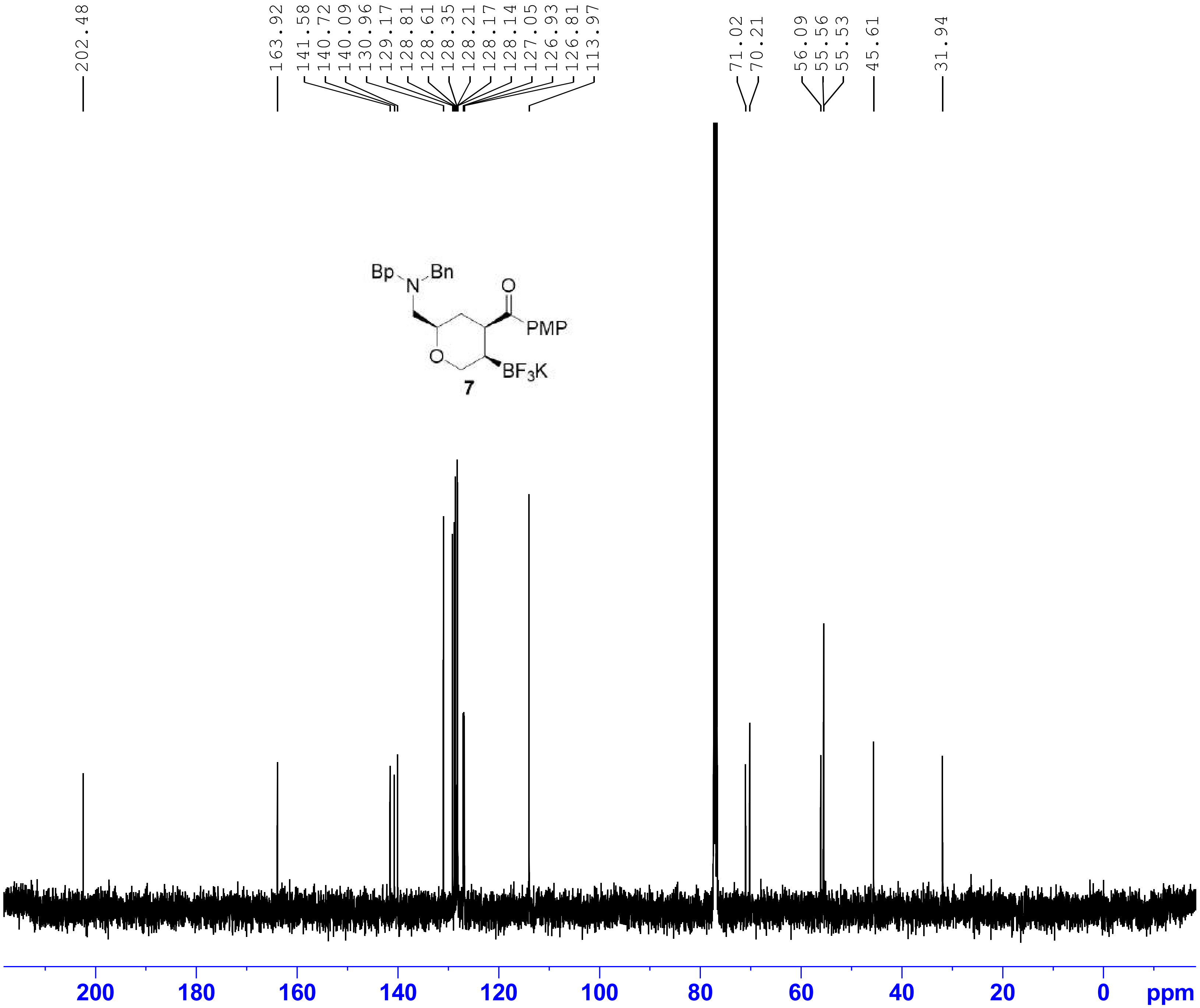

Current Data Parameters
NAME zmh-bf3k-chiral
EXPNO 2
PROCNO 1

F2 - Acquisition Parameters
Date_ 20240906
Time 11.40 h
INSTRUM AvanceNeo 400MHz
PROBHD Z163739_0629 (
PULPROG zgpg30
TD 65536
SOLVENT CDCl3
NS 325
DS 4
SWH 23809.523 Hz
FIDRES 0.726609 Hz
AQ 1.3762560 sec
RG 10
DW 21.000 usec
DE 6.50 usec
TE 297.2 K
D1 2.00000000 sec
D11 0.03000000 sec
TD0 1
SFO1 100.6354036 MHz
NUC1 13C
P0 2.67 usec
P1 8.00 usec
PLW1 85.25399780 W
SFO2 400.1816007 MHz
NUC2 1H
CPDPRG[2 waltz65
PCPD2 90.00 usec
PLW2 21.26700020 W
PLW12 0.16802999 W
PLW13 0.08452000 W

F2 - Processing parameters
SI 32768
SF 100.6253454 MHz
WDW EM
SSB 0
LB 1.00 Hz
GB 0
PC 1.40

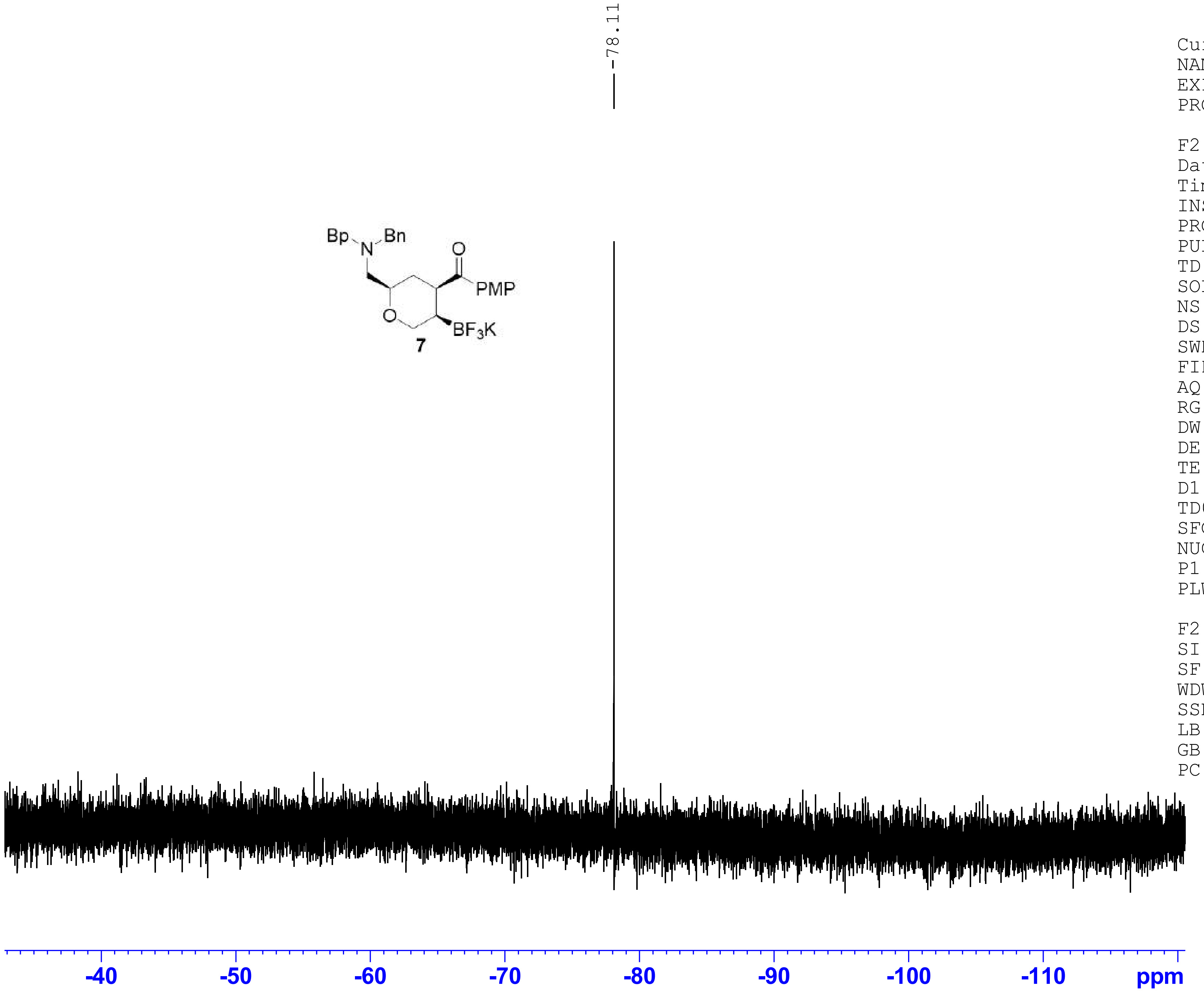

Current Data Parameters
NAME zmh-bf3k-chiral
EXPNO 3
PROCNO 1

F2 - Acquisition Parameters
Date_ 20240906
Time 11.42 h
INSTRUM AvanceNeo 400MHz
PROBHD Z163739_0629 (
PULPROG zg
TD 131072
SOLVENT CDCl3
NS 16
DS 4
SWH 90909.094 Hz
FIDRES 1.387163 Hz
AQ 0.7208960 sec
RG 101
DW 5.500 usec
DE 6.50 usec
TE 297.1 K
D1 1.00000000 sec
TD0 1
SFO1 376.5077587 MHz
NUC1 19F
P1 12.00 usec
PLW1 33.72800064 W

F2 - Processing parameters
SI 65536
SF 376.5454132 MHz
WDW EM
SSB 0
LB 0.30 Hz
GB 0
PC 1.00

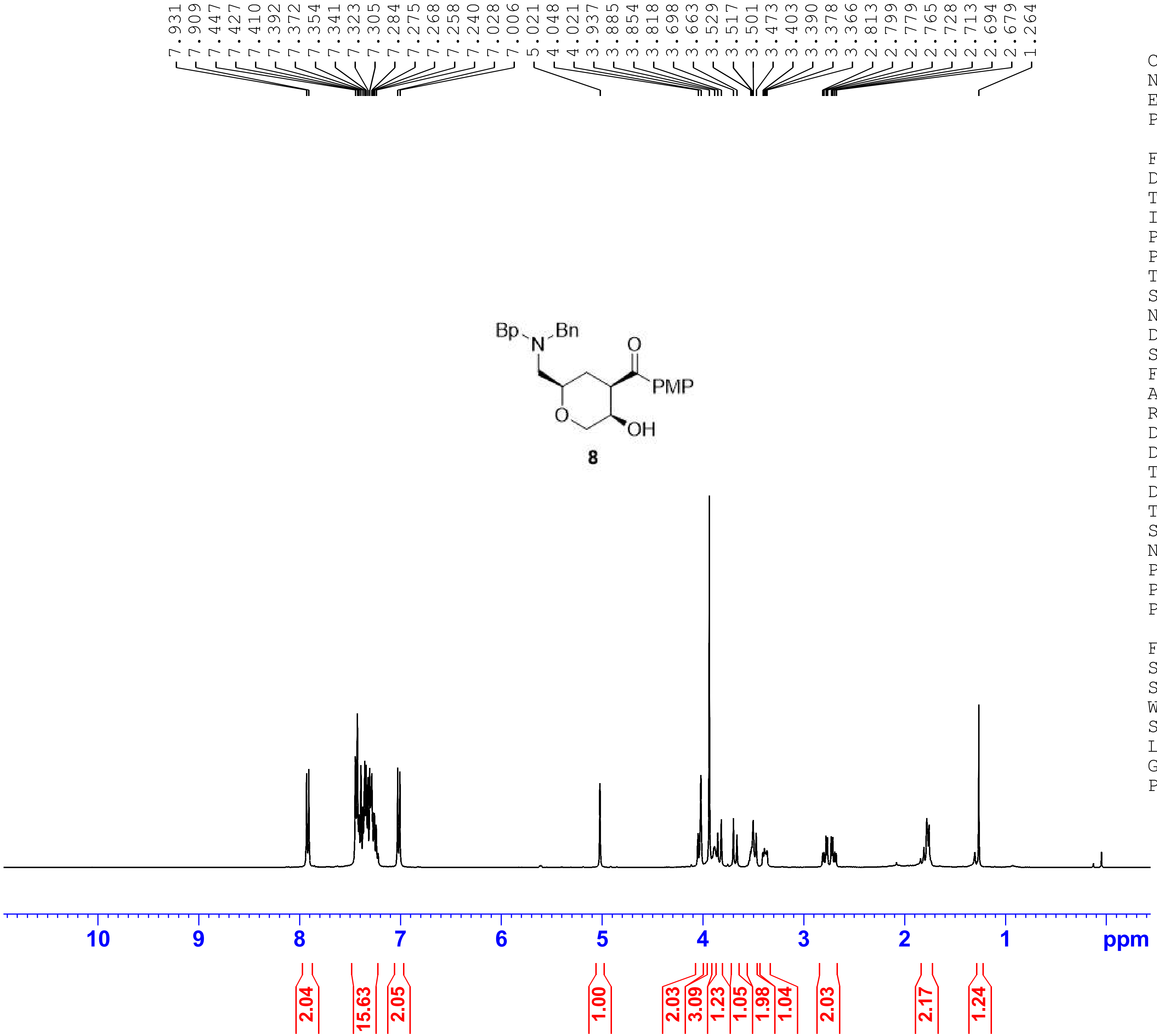

8

Current Data Parameters
NAME zmh-5-ys-oh
EXPNO 1
PROCNO 1

F2 - Acquisition Parameters
Date_ 20240604
Time 10.26 h
INSTRUM AvanceNeo 400MHz
PROBHD Z163739_0629 (
PULPROG zg30
TD 65536
SOLVENT CDCl3
NS 8
DS 2
SWH 8196.722 Hz
FIDRES 0.250144 Hz
AQ 3.9976959 sec
RG 45.2
DW 61.000 usec
DE 13.89 usec
TE 296.9 K
D1 1.00000000 sec
TD0 1
SFO1 400.1824711 MHz
NUC1 1H
P0 2.67 usec
P1 8.00 usec
PLW1 21.26700020 W

F2 - Processing parameters
SI 65536
SF 400.1800000 MHz
WDW EM
SSB 0
LB 0.30 Hz
GB 0
PC 1.00

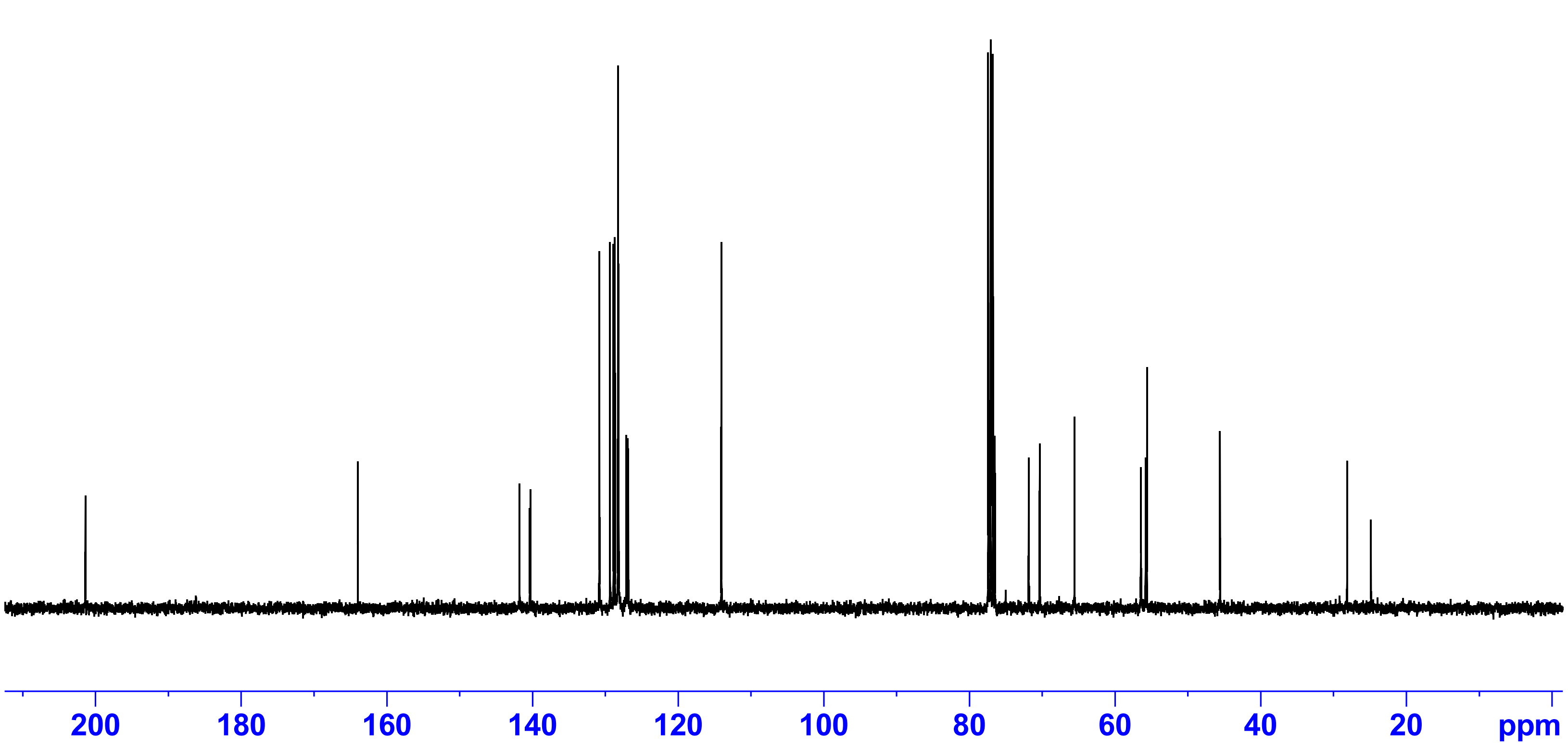

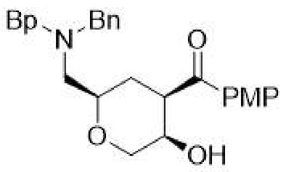

8

```
Current Data Parameters
NAME          zmh-5-ys-oh
EXPNO                   2
PROCNO                  1

F2 - Acquisition Parameters
Date_            20240604
Time              10.33 h
INSTRUM   AvanceNeo 400MHz
PROBHD    Z163739_0629 (
PULPROG           zgpg30
TD                 65536
SOLVENT            CDCl3
NS                   100
DS                     4
SWH            23809.523 Hz
FIDRES          0.726609 Hz
AQ             1.3762560 sec
RG                    10
DW                21.000 usec
DE                  6.50 usec
TE                 297.7 K
D1            2.00000000 sec
D11           0.03000000 sec
TD0                    1
SFO1         100.6354036 MHz
NUC1                 13C
P0                  2.67 usec
P1                  8.00 usec
PLW1         85.25399780 W
SFO2         400.1816007 MHz
NUC2                  1H
CPDPRG[2         waltz65
PCPD2              90.00 usec
PLW2         21.26700020 W
PLW12         0.16802999 W
PLW13         0.08452000 W

F2 - Processing parameters
SI                 32768
SF           100.6253410 MHz
WDW                   EM
SSB                    0
LB                  1.00 Hz
GB                     0
PC                  1.40
```

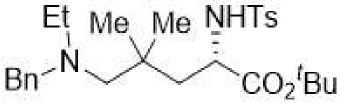

9

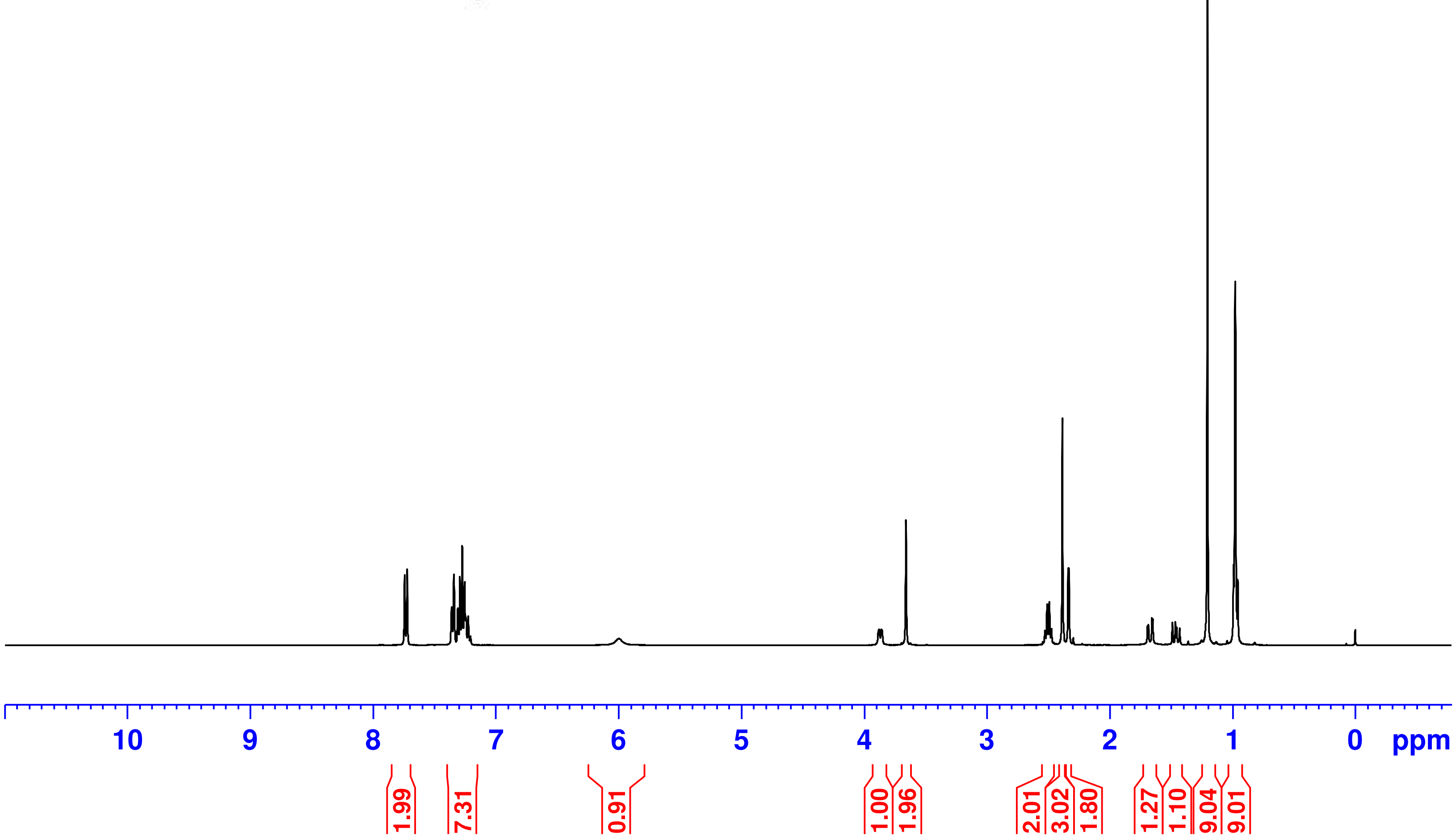

Current Data Parameters
NAME zmh-2-120-chun-
EXPNO 1
PROCNO 1

F2 - Acquisition Parameters
Date_ 20230118
Time 18.26
INSTRUM spect
PROBHD 5 mm DUL 13C-1
PULPROG zg30
TD 65536
SOLVENT CDCl3
NS 16
DS 2
SWH 8223.685 Hz
FIDRES 0.125483 Hz
AQ 3.9845889 sec
RG 181
DW 60.800 usec
DE 6.00 usec
TE 292.6 K
D1 1.00000000 sec
TD0 1

======== CHANNEL f1 ========
NUC1 1H
P1 15.80 usec
PL1 -1.00 dB
PL1W 12.17476940 W
SFO1 400.1324710 MHz

F2 - Processing parameters
SI 32768
SF 400.1300099 MHz
WDW EM
SSB 0
LB 0.30 Hz
GB 0
PC 1.00

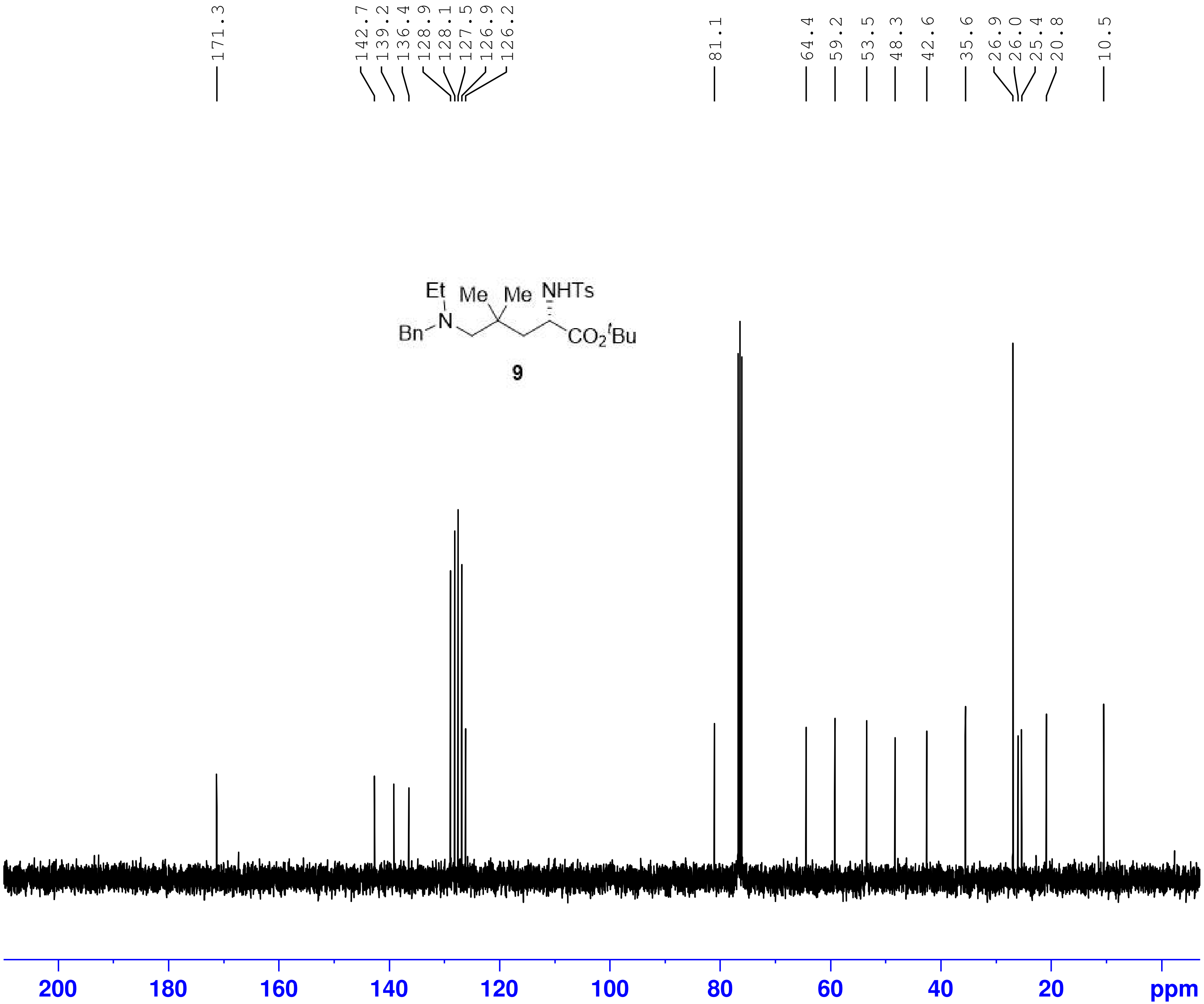


```
Current Data Parameters
NAME       zmh-2-120-chun-c
EXPNO                     2
PROCNO                    1

F2 - Acquisition Parameters
Date_              20230118
Time                  18.29
INSTRUM               spect
PROBHD    5 mm DUL 13C-1
PULPROG             zgpg30
TD                    65536
SOLVENT               CDCl3
NS                      101
DS                        1
SWH              24038.461 Hz
FIDRES            0.366798 Hz
AQ               1.3631488 sec
RG                     2050
DW                  20.800 usec
DE                    6.00 usec
TE                   292.7 K
D1              2.00000000 sec
D11             0.03000000 sec
TD0                       1

======== CHANNEL f1 ========
NUC1                    13C
P1                    40.00 usec
PL1                   -3.00 dB
PL1W           60.64365387 W
SFO1           100.6228298 MHz

======== CHANNEL f2 ========
CPDPRG[2            waltz16
NUC2                     1H
PCPD2                 80.00 usec
PL2                   -1.00 dB
PL12                  14.39 dB
PL13                  18.00 dB
PL2W           12.17476940 W
PL12W           0.35193357 W
PL13W           0.15327126 W
SFO2           400.1316005 MHz

F2 - Processing parameters
SI                    32768
SF             100.6128330 MHz
WDW                      EM
SSB                       0
LB                     1.00 Hz
GB                        0
PC                     1.40
```

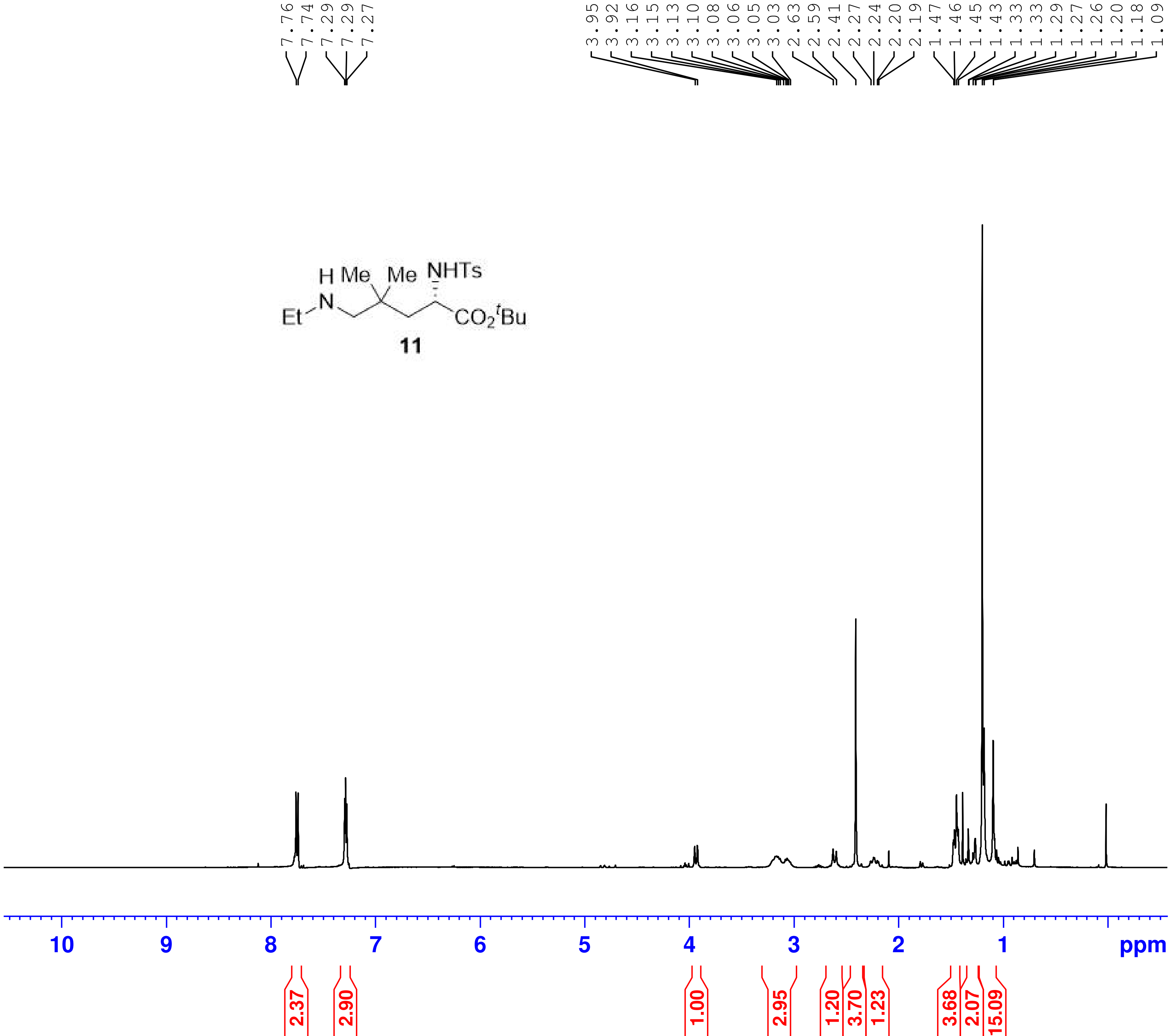

Current Data Parameters
NAME zmh-2-121
EXPNO 1
PROCNO 1

F2 - Acquisition Parameters
Date_ 20230120
Time 19.13
INSTRUM spect
PROBHD 5 mm PABBO BB/
PULPROG zg30
TD 65536
SOLVENT CDCl3
NS 8
DS 2
SWH 8012.820 Hz
FIDRES 0.122266 Hz
AQ 4.0894465 sec
RG 82.92
DW 62.400 usec
DE 6.50 usec
TE 293.6 K
D1 1.00000000 sec
TD0 1

======== CHANNEL f1 ========
SFO1 400.1324710 MHz
NUC1 1H
P1 14.50 usec
PLW1 11.99499989 W

F2 - Processing parameters
SI 65536
SF 400.1300000 MHz
WDW EM
SSB 0
LB 0.30 Hz
GB 0
PC 1.00

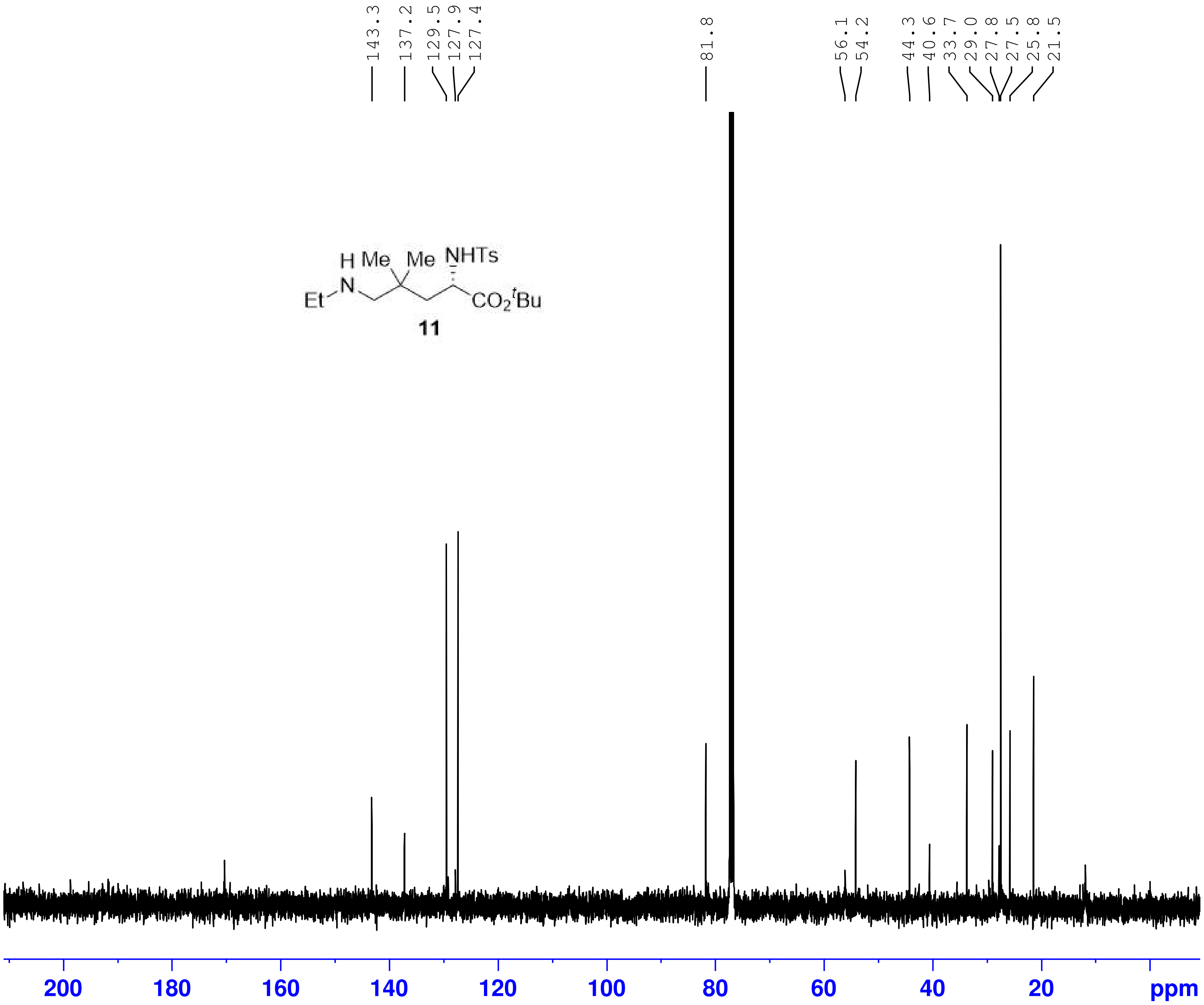

Current Data Parameters
NAME zmh-2-121
EXPNO 2
PROCNO 1

F2 - Acquisition Parameters
Date_ 20230120
Time 19.15
INSTRUM spect
PROBHD 5 mm PABBO BB/
PULPROG zgpg30
TD 65536
SOLVENT CDCl3
NS 493
DS 2
SWH 24038.461 Hz
FIDRES 0.366798 Hz
AQ 1.3631488 sec
RG 196.92
DW 20.800 usec
DE 6.50 usec
TE 294.3 K
D1 2.00000000 sec
D11 0.03000000 sec
TD0 1

======== CHANNEL f1 ========
SFO1 100.6228298 MHz
NUC1 13C
P1 9.70 usec
PLW1 46.98899841 W

======== CHANNEL f2 ========
SFO2 400.1316005 MHz
NUC2 1H
CPDPRG[2 waltz16
PCPD2 90.00 usec
PLW2 11.99499989 W
PLW12 0.34213999 W
PLW13 0.27713001 W

F2 - Processing parameters
SI 32768
SF 100.6127690 MHz
WDW EM
SSB 0
LB 1.00 Hz
GB 0
PC 1.40

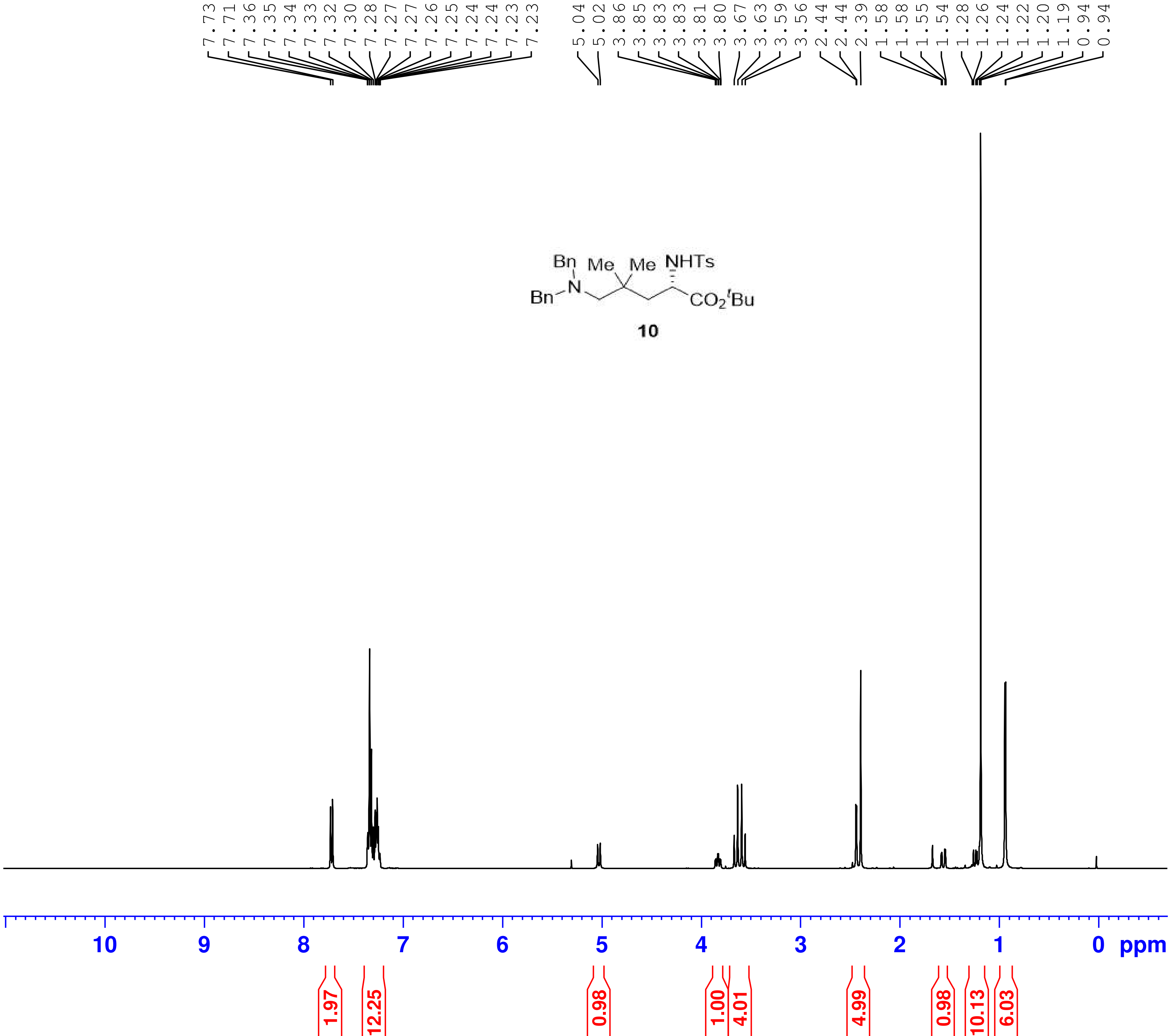

10

Current Data Parameters
NAME zmh-2-122
EXPNO 1
PROCNO 1

F2 - Acquisition Parameters
Date_ 20230119
Time 18.26
INSTRUM spect
PROBHD 5 mm DUL 13C-1
PULPROG zg30
TD 65536
SOLVENT CDCl3
NS 16
DS 2
SWH 8223.685 Hz
FIDRES 0.125483 Hz
AQ 3.9845889 sec
RG 144
DW 60.800 usec
DE 6.00 usec
TE 292.6 K
D1 1.00000000 sec
TD0 1

======== CHANNEL f1 ========
NUC1 1H
P1 15.80 usec
PL1 -1.00 dB
PL1W 12.17476940 W
SFO1 400.1324710 MHz

F2 - Processing parameters
SI 32768
SF 400.1300054 MHz
WDW EM
SSB 0
LB 0.30 Hz
GB 0
PC 1.00

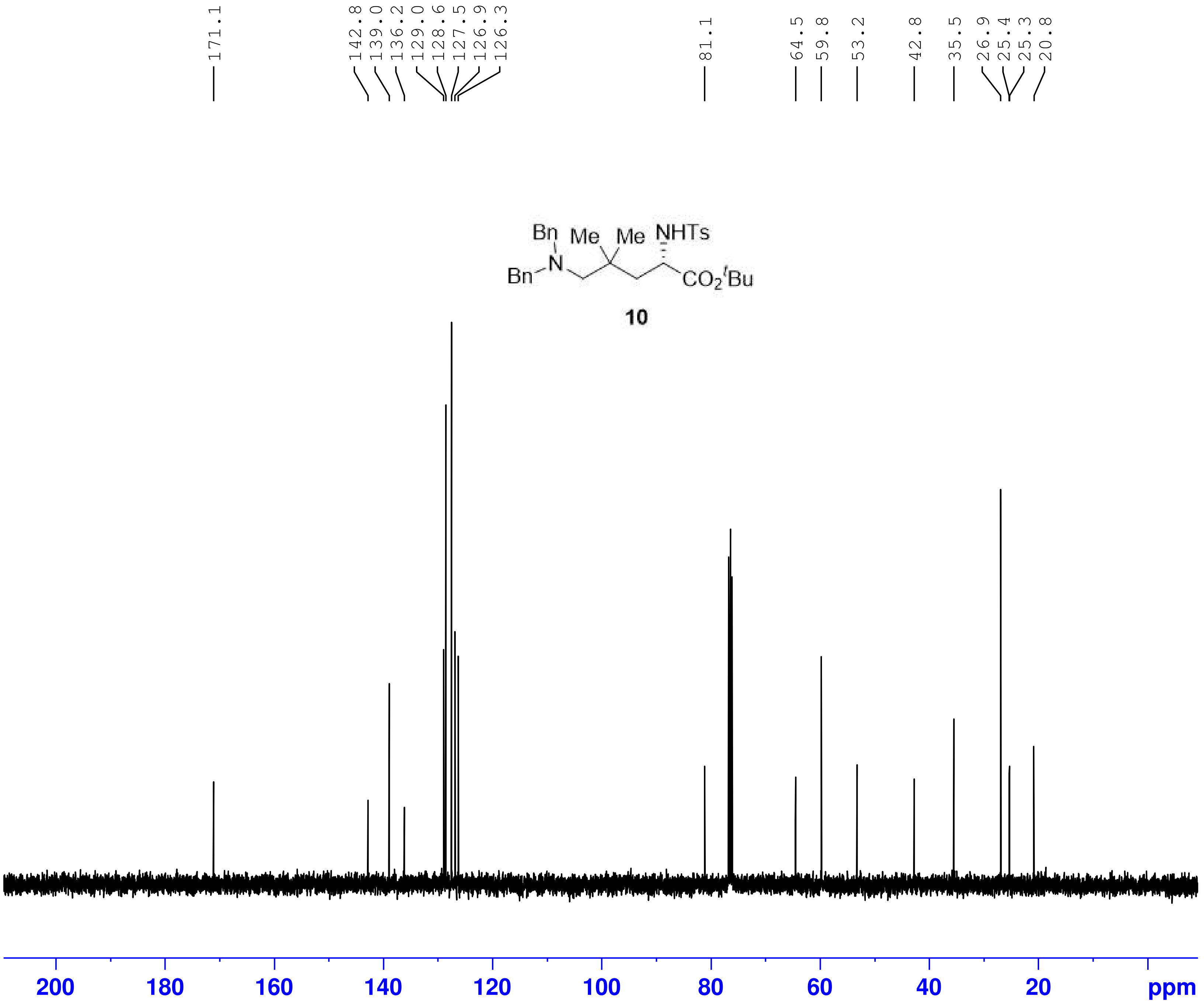


```
Current Data Parameters
NAME             zmh-2-122
EXPNO                    2
PROCNO                   1

F2 - Acquisition Parameters
Date_             20230119
Time                 18.29
INSTRUM              spect
PROBHD      5 mm DUL 13C-1
PULPROG            zgpg30
TD                   65536
SOLVENT              CDCl3
NS                     101
DS                       1
SWH              24038.461 Hz
FIDRES            0.366798 Hz
AQ               1.3631488 sec
RG                    2050
DW                  20.800 usec
DE                    6.00 usec
TE                   292.9 K
D1              2.00000000 sec
D11             0.03000000 sec
TD0                      1

======== CHANNEL f1 ========
NUC1                   13C
P1                   40.00 usec
PL1                  -3.00 dB
PL1W           60.64365387 W
SFO1           100.6228298 MHz

======== CHANNEL f2 ========
CPDPRG[2           waltz16
NUC2                    1H
PCPD2                80.00 usec
PL2                  -1.00 dB
PL12                 14.39 dB
PL13                 18.00 dB
PL2W           12.17476940 W
PL12W           0.35193357 W
PL13W           0.15327126 W
SFO2           400.1316005 MHz

F2 - Processing parameters
SI                   32768
SF             100.6128330 MHz
WDW                     EM
SSB                      0
LB                    1.00 Hz
GB                       0
PC                    1.40
```

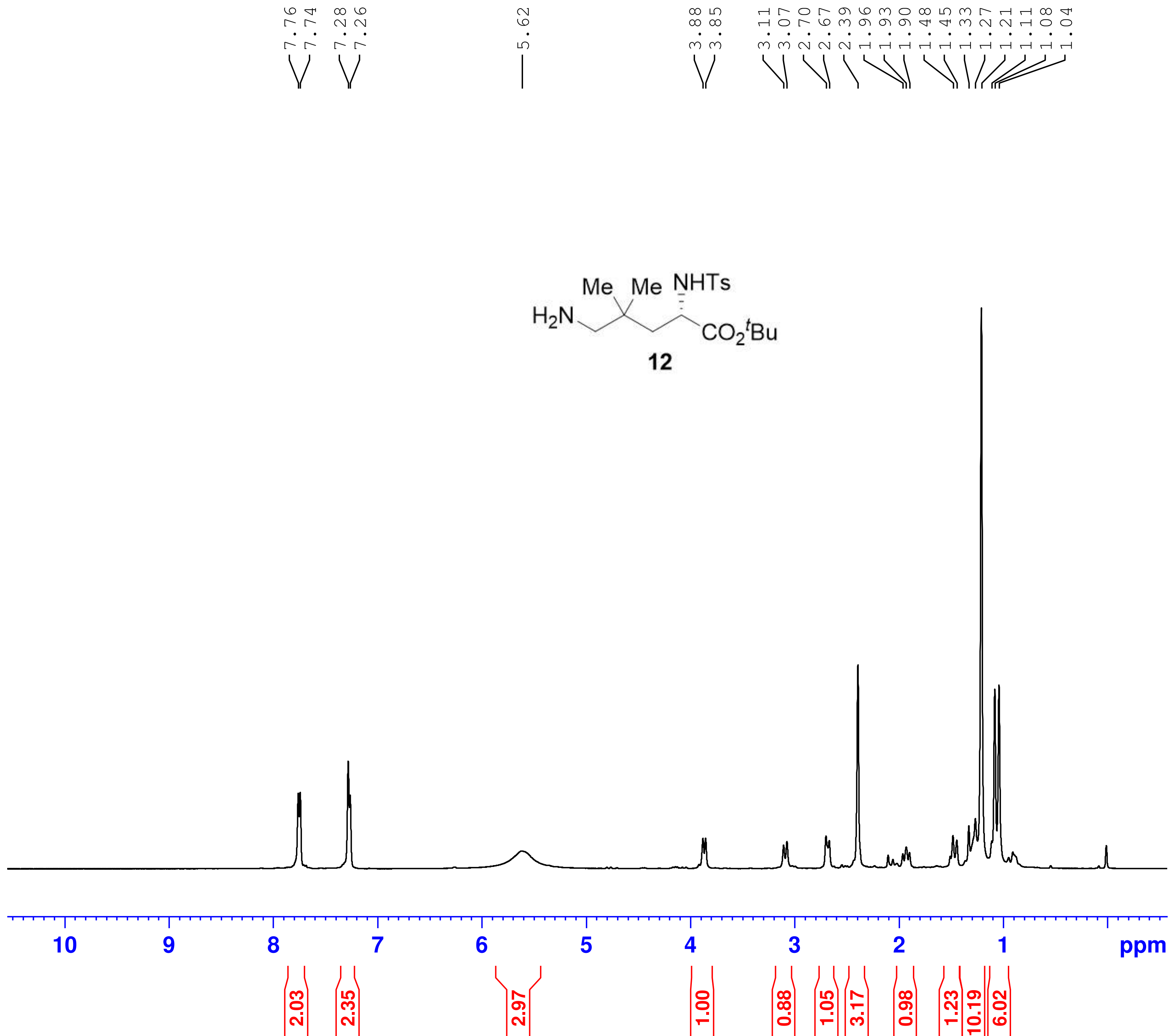

Current Data Parameters
NAME zmh-2-124
EXPNO 5
PROCNO 1

F2 - Acquisition Parameters
Date_ 20230120
Time 18.56 h
INSTRUM AvanceNeo 400MHz
PROBHD Z163739_0629 (
PULPROG zg30
TD 65536
SOLVENT CDCl3
NS 8
DS 2
SWH 8196.722 Hz
FIDRES 0.250144 Hz
AQ 3.9976959 sec
RG 101
DW 61.000 usec
DE 13.89 usec
TE 295.2 K
D1 1.00000000 sec
TD0 1
SFO1 400.1824711 MHz
NUC1 1H
P0 2.67 usec
P1 8.00 usec
PLW1 21.26700020 W

F2 - Processing parameters
SI 65536
SF 400.1800000 MHz
WDW EM
SSB 0
LB 0.30 Hz
GB 0
PC 1.00

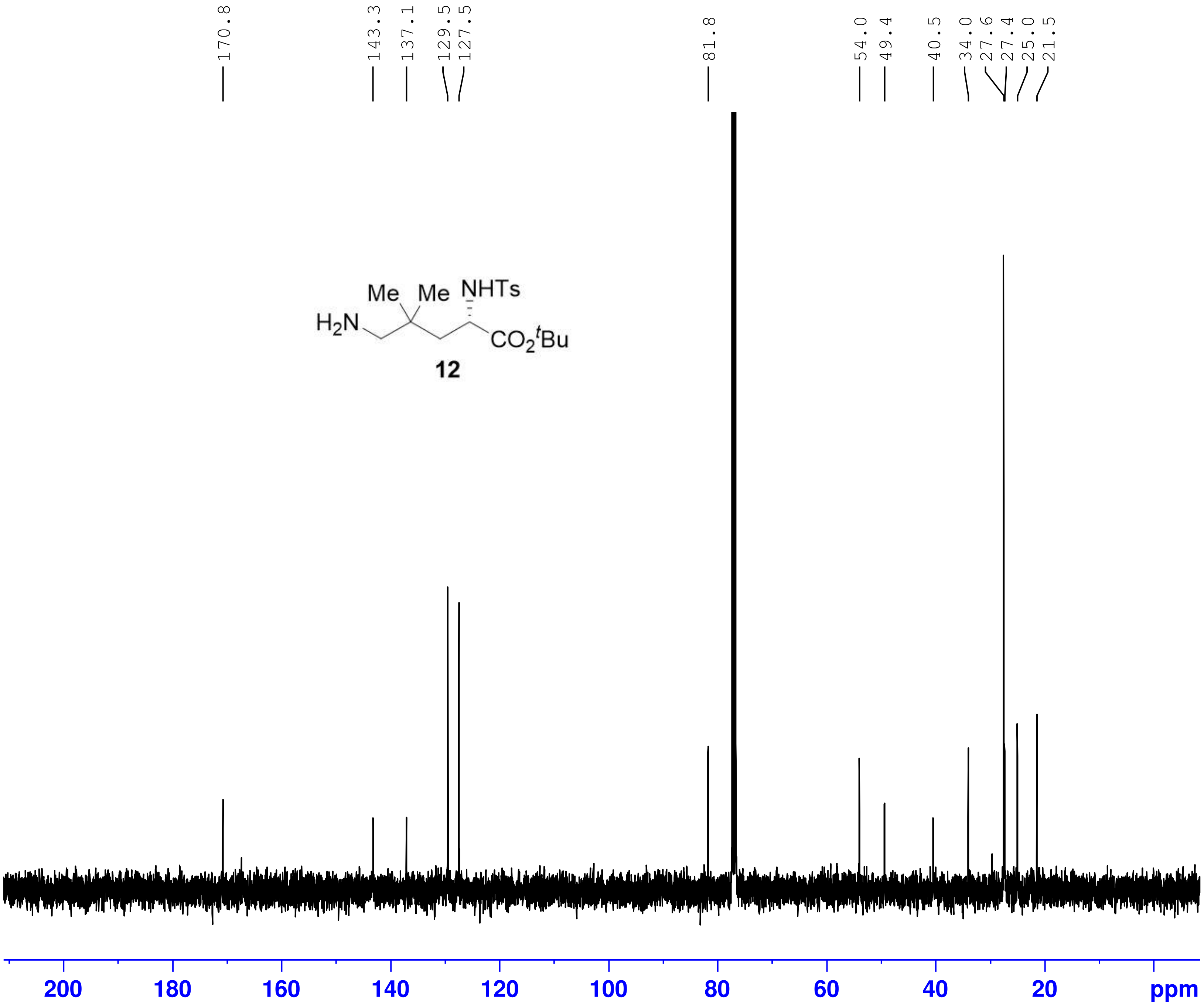

Current Data Parameters
NAME zmh-2-124
EXPNO 6
PROCNO 1

F2 - Acquisition Parameters
Date_ 20230120
Time 19.15 h
INSTRUM AvanceNeo 400MHz
PROBHD Z163739_0629 (
PULPROG zgpg30
TD 65536
SOLVENT CDCl3
NS 296
DS 4
SWH 23809.523 Hz
FIDRES 0.726609 Hz
AQ 1.3762560 sec
RG 10
DW 21.000 usec
DE 6.50 usec
TE 295.7 K
D1 2.00000000 sec
D11 0.03000000 sec
TD0 1
SFO1 100.6354036 MHz
NUC1 13C
P0 2.67 usec
P1 8.00 usec
PLW1 85.25399780 W
SFO2 400.1816007 MHz
NUC2 1H
CPDPRG[2 waltz65
PCPD2 90.00 usec
PLW2 21.26700020 W
PLW12 0.16802999 W
PLW13 0.08452000 W

F2 - Processing parameters
SI 32768
SF 100.6253410 MHz
WDW EM
SSB 0
LB 1.00 Hz
GB 0
PC 1.40

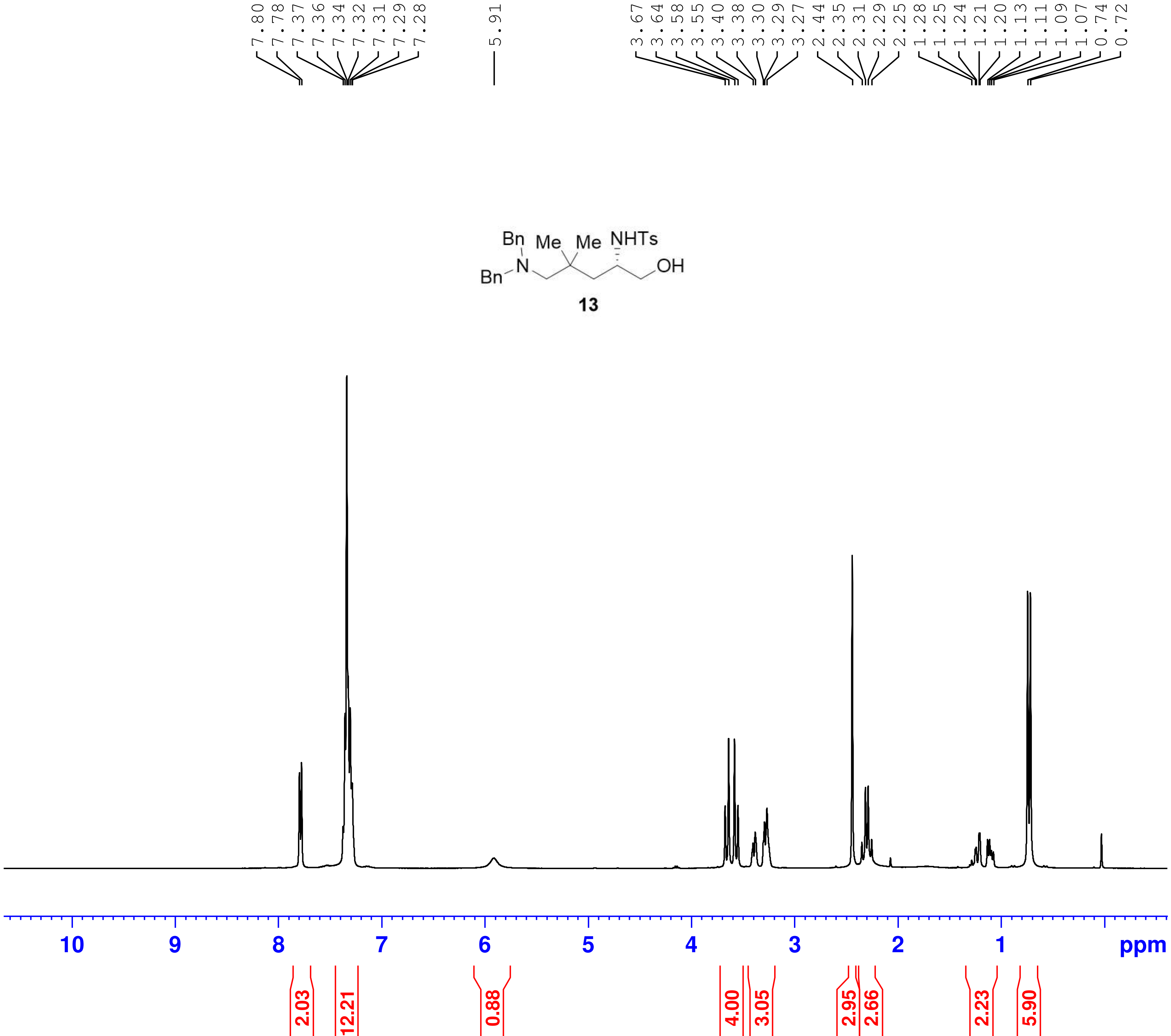

Current Data Parameters
NAME zmh-2-123
EXPNO 1
PROCNO 1

F2 - Acquisition Parameters
Date_ 20230120
Time 18.42 h
INSTRUM AvanceNeo 400MHz
PROBHD Z163739_0629 (
PULPROG zg30
TD 65536
SOLVENT CDCl3
NS 8
DS 2
SWH 8196.722 Hz
FIDRES 0.250144 Hz
AQ 3.9976959 sec
RG 101
DW 61.000 usec
DE 13.89 usec
TE 294.2 K
D1 1.00000000 sec
TD0 1
SFO1 400.1824711 MHz
NUC1 1H
P0 2.67 usec
P1 8.00 usec
PLW1 21.26700020 W

F2 - Processing parameters
SI 65536
SF 400.1800000 MHz
WDW EM
SSB 0
LB 0.30 Hz
GB 0
PC 1.00

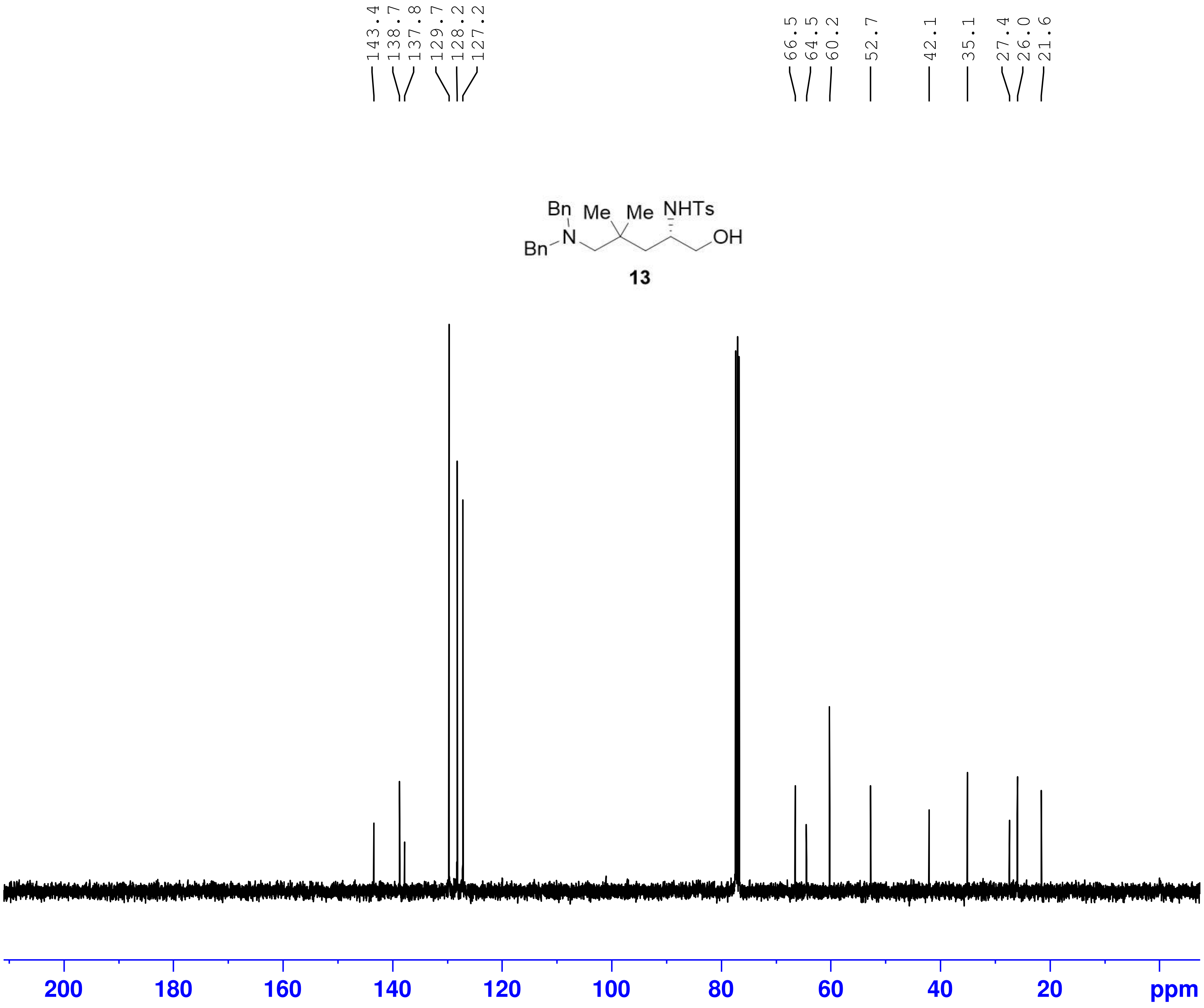

Current Data Parameters
NAME zmh-2-123
EXPNO 2
PROCNO 1

F2 - Acquisition Parameters
Date_ 20230120
Time 18.50 h
INSTRUM AvanceNeo 400MHz
PROBHD Z163739_0629 (
PULPROG zgpg30
TD 65536
SOLVENT CDCl3
NS 125
DS 4
SWH 23809.523 Hz
FIDRES 0.726609 Hz
AQ 1.3762560 sec
RG 10
DW 21.000 usec
DE 6.50 usec
TE 294.8 K
D1 2.00000000 sec
D11 0.03000000 sec
TD0 1
SFO1 100.6354036 MHz
NUC1 13C
P0 2.67 usec
P1 8.00 usec
PLW1 85.25399780 W
SFO2 400.1816007 MHz
NUC2 1H
CPDPRG[2 waltz65
PCPD2 90.00 usec
PLW2 21.26700020 W
PLW12 0.16802999 W
PLW13 0.08452000 W

F2 - Processing parameters
SI 32768
SF 100.6253410 MHz
WDW EM
SSB 0
LB 1.00 Hz
GB 0
PC 1.40

===

```
Acq. Operator   : SYSTEM                       Seq. Line :  63
Sample Operator : SYSTEM
Acq. Instrument : HPLC                          Location :   P1-F-04
Injection Date  : 18/4/2024 2:58:51 am               Inj :   1
                                              Inj Volume : 2.000 µl
Different Inj Volume from Sample Entry!  Actual Inj Volume : 10.000 µl
Acq. Method     : C:\Users\Public\Documents\ChemStation\1\Data\SUN\SUN 2024-04-17 10-37-28
                  \AD3-10-20.M
Last changed    : 15/8/2022 10:21:32 pm by SYSTEM
Analysis Method : C:\Users\Public\Documents\ChemStation\1\Data\SUN\SUN 2024-04-17 10-37-28
                  \AD3-10-20.M (Sequence Method)
Last changed    : 29/6/2024 3:52:03 pm by SYSTEM
                  (modified after loading)
Additional Info : Peak(s) manually integrated
```

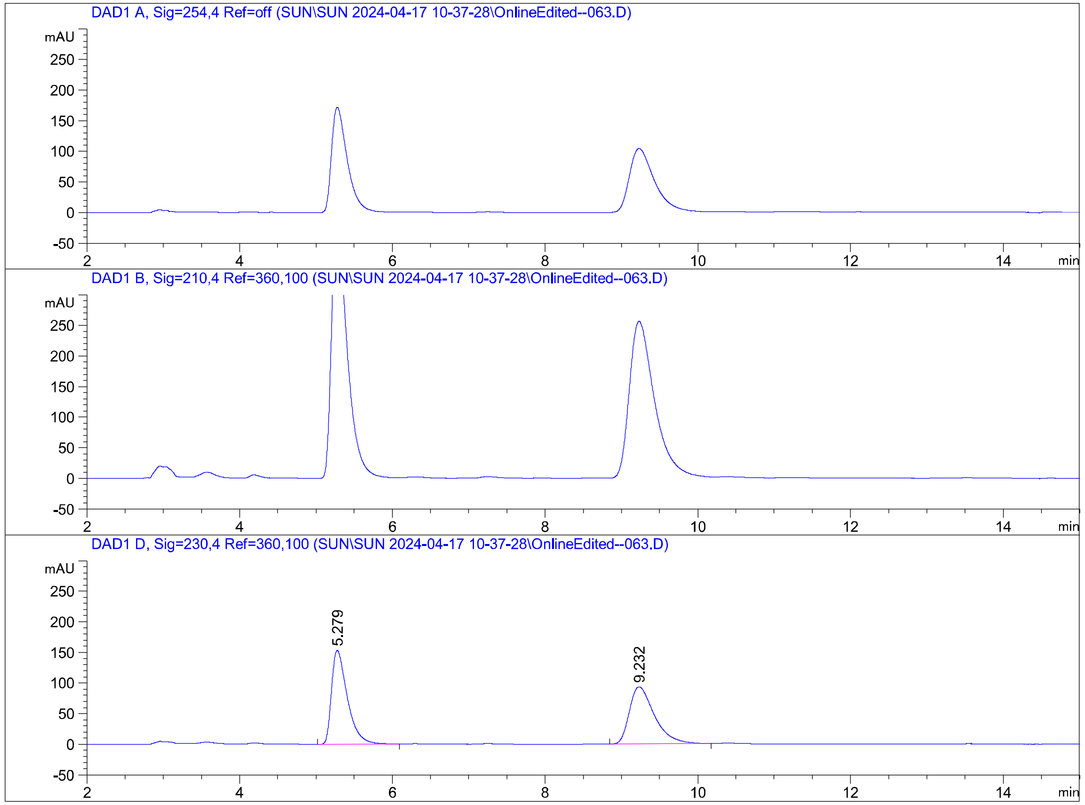

Data File C:\Users\P...\ChemStation\1\Data\SUN\SUN 2024-04-17 10-37-28\OnlineEdited--063.D
Sample Name: zmh-5-148d-rac-ad

===
Area Percent Report
===

```
Sorted By             :      Signal
Multiplier            :      1.0000
Dilution              :      1.0000
Use Multiplier & Dilution Factor with ISTDs
```

Signal 1: DAD1 A, Sig=254,4 Ref=off

Signal 2: DAD1 B, Sig=210,4 Ref=360,100

Signal 3: DAD1 D, Sig=230,4 Ref=360,100

Peak #	RetTime [min]	Type	Width [min]	Area [mAU*s]	Height [mAU]	Area %
1	5.279	BB	0.2164	2204.85718	153.24675	50.9442
2	9.232	BV R	0.3403	2123.12451	93.36269	49.0558

Totals : 4327.98169 246.60944

===
*** End of Report ***

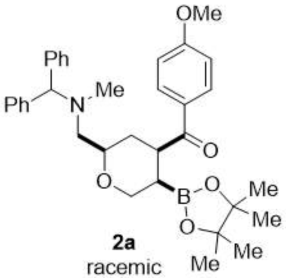

2a
racemic

===

Acq. Operator : SYSTEM Seq. Line : 2
Sample Operator : SYSTEM
Acq. Instrument : HPLC Location : P1-F-01
Injection Date : 14/5/2024 3:33:09 pm Inj : 1
Inj Volume : 2.000 µl
Different Inj Volume from Sample Entry! Actual Inj Volume : 10.000 µl
Acq. Method : C:\Users\Public\Documents\ChemStation\1\Data\SUN\SUN 2024-05-14 15-20-06\AD3-10-20.M
Last changed : 15/8/2022 10:21:32 pm by SYSTEM
Analysis Method : C:\Users\Public\Documents\ChemStation\1\Data\SUN\SUN 2024-05-14 15-20-06\AD3-10-20.M (Sequence Method)
Last changed : 29/6/2024 2:57:20 pm by SYSTEM
(modified after loading)
Additional Info : Peak(s) manually integrated

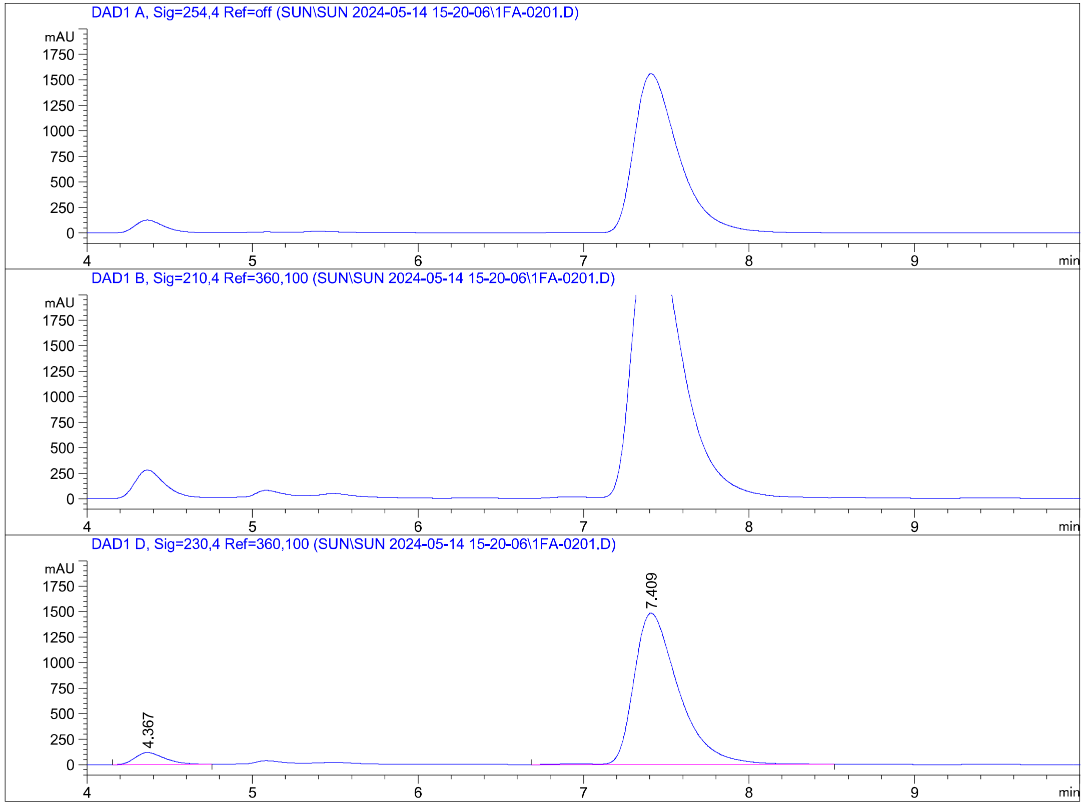

Data File C:\Users\P...Documents\ChemStation\1\Data\SUN\SUN 2024-05-14 15-20-06\1FA-0201.D
Sample Name: zmh-5-180b

===

Area Percent Report

===

Sorted By : Signal
Multiplier : 1.0000
Dilution : 1.0000
Use Multiplier & Dilution Factor with ISTDs

Signal 1: DAD1 A, Sig=254,4 Ref=off

Signal 2: DAD1 B, Sig=210,4 Ref=360,100

Signal 3: DAD1 D, Sig=230,4 Ref=360,100

Peak #	RetTime [min]	Type	Width [min]	Area [mAU*s]	Height [mAU]	Area %
1	4.367	BB	0.1857	1430.95459	117.02262	4.9063
2	7.409	VB R	0.2826	2.77345e4	1481.68115	95.0937

Totals : 2.91654e4 1598.70377

===

*** End of Report ***

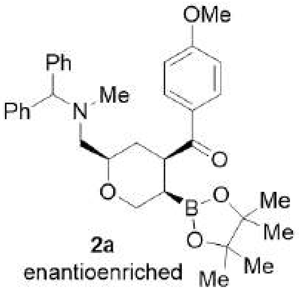

2a
enantioenriched

===

Acq. Operator : SYSTEM Seq. Line : 64
Sample Operator : SYSTEM
Acq. Instrument : HPLC Location : P1-F-05
Injection Date : 18/4/2024 3:20:05 am Inj : 1
Inj Volume : 2.000 µl
Different Inj Volume from Sample Entry! Actual Inj Volume : 10.000 µl
Acq. Method : C:\Users\Public\Documents\ChemStation\1\Data\SUN\SUN 2024-04-17 10-37-28\AD3-10-20.M
Last changed : 15/8/2022 10:21:32 pm by SYSTEM
Analysis Method : C:\Users\Public\Documents\ChemStation\1\Data\SUN\SUN 2024-04-17 10-37-28\AD3-10-20.M (Sequence Method)
Last changed : 29/6/2024 2:45:28 pm by SYSTEM
(modified after loading)
Additional Info : Peak(s) manually integrated

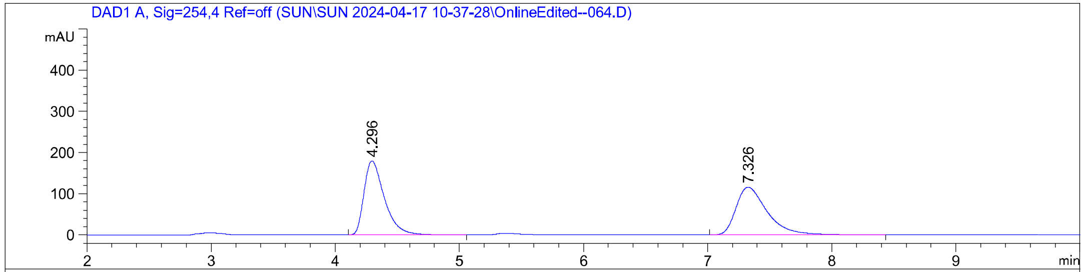

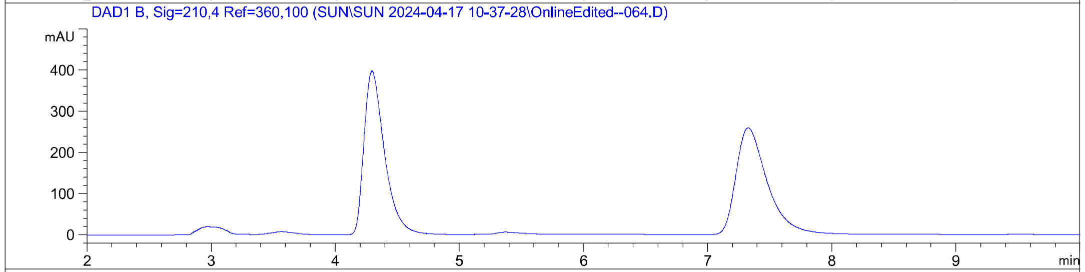

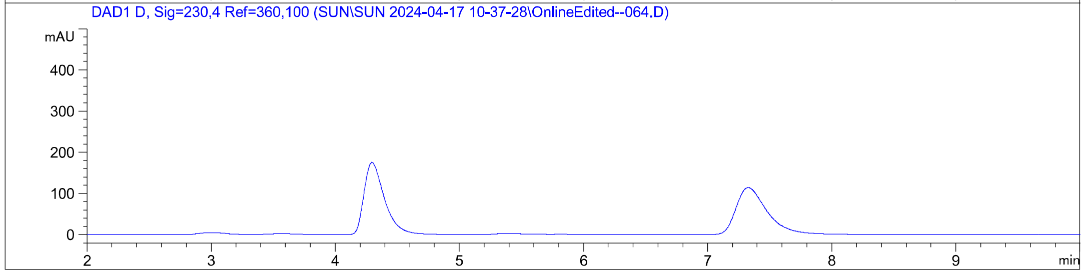

Data File C:\Users\P...\ChemStation\1\Data\SUN\SUN 2024-04-17 10-37-28\OnlineEdited--064.D
Sample Name: zmh-5-148e-rac-ad

===

Area Percent Report

===

Sorted By : Signal
Multiplier : 1.0000
Dilution : 1.0000
Use Multiplier & Dilution Factor with ISTDs

Signal 1: DAD1 A, Sig=254,4 Ref=off

Peak #	RetTime [min]	Type	Width [min]	Area [mAU*s]	Height [mAU]	Area %
1	4.296	BB	0.1729	2035.73035	179.37199	50.2246
2	7.326	BB	0.2624	2017.52344	115.69946	49.7754

Totals : 4053.25378 295.07145

Signal 2: DAD1 B, Sig=210,4 Ref=360,100

Signal 3: DAD1 D, Sig=230,4 Ref=360,100

===

*** End of Report ***

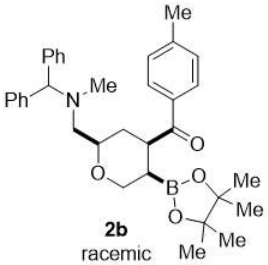

2b
racemic

```
=====================================================================
Acq. Operator   : SYSTEM                          Seq. Line :   2
Sample Operator : SYSTEM
Acq. Instrument : HPLC                             Location :   P1-F-01
Injection Date  : 14/5/2024 1:21:15 pm                  Inj :   1
                                                 Inj Volume : 2.000 µl
Different Inj Volume from Sample Entry!  Actual Inj Volume : 10.000 µl
Acq. Method     : C:\Users\Public\Documents\ChemStation\1\Data\SUN\SUN 2024-05-14 13-08-05
                  \AD3-10-20.M
Last changed    : 15/8/2022 10:21:32 pm by SYSTEM
Analysis Method : C:\Users\Public\Documents\ChemStation\1\Data\SUN\SUN 2024-05-14 13-08-05
                  \AD3-10-20.M (Sequence Method)
Last changed    : 29/6/2024 2:49:00 pm by SYSTEM
                  (modified after loading)
Additional Info : Peak(s) manually integrated
```

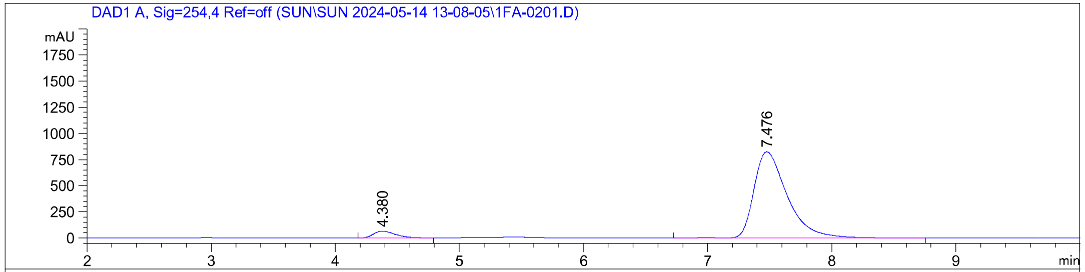

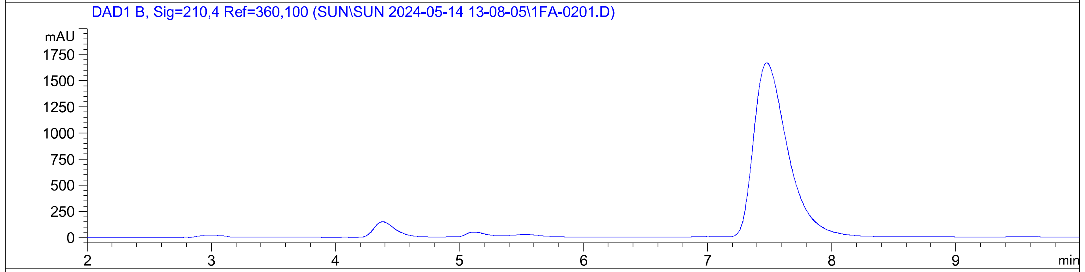

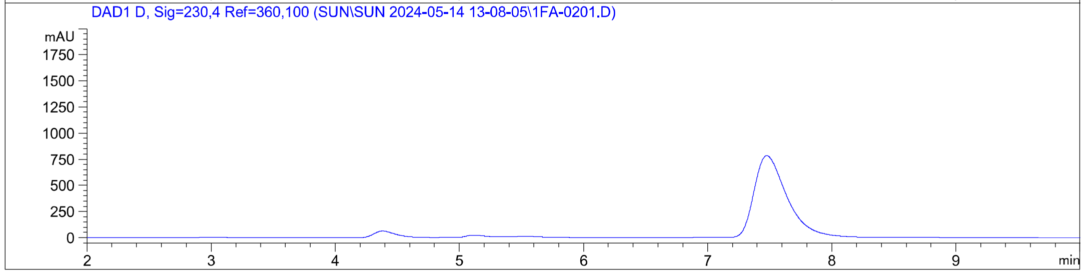

Data File C:\Users\P...Documents\ChemStation\1\Data\SUN\SUN 2024-05-14 13-08-05\1FA-0201.D
Sample Name: zmh-5-180a

```
=====================================================================
                         Area Percent Report
=====================================================================

Sorted By             :      Signal
Multiplier            :      1.0000
Dilution              :      1.0000
Use Multiplier & Dilution Factor with ISTDs


Signal 1: DAD1 A, Sig=254,4 Ref=off

Peak RetTime Type  Width     Area      Height     Area
  #   [min]        [min]   [mAU*s]     [mAU]        %
----|-------|----|-------|----------|----------|--------|
   1   4.380 BB    0.1801  776.68286   66.08646   4.8382
   2   7.476 VB R  0.2805 1.52764e4   824.59503  95.1618

Totals :                  1.60531e4   890.68149



Signal 2: DAD1 B, Sig=210,4 Ref=360,100


Signal 3: DAD1 D, Sig=230,4 Ref=360,100

=====================================================================
                          *** End of Report ***
```

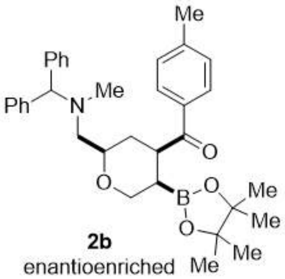

2b
enantioenriched

```
=====================================================================
Acq. Operator   : SYSTEM                        Seq. Line :  26
Sample Operator : SYSTEM
Acq. Instrument : HPLC                           Location :   P2-E-01
Injection Date  : 15/11/2023 8:00:03 pm               Inj :   1
                                               Inj Volume : 2.000 µl
Different Inj Volume from Sample Entry!  Actual Inj Volume : 10.000 µl
Acq. Method     : C:\Users\Public\Documents\ChemStation\1\Data\SUN\SUN 2023-11-15 10-00-07
                  \AD3-10-30.M
Last changed    : 24/10/2022 9:57:21 am by SYSTEM
Analysis Method : C:\Users\Public\Documents\ChemStation\1\Data\SUN\SUN 2023-11-15 10-00-07
                  \AD3-10-30.M (Sequence Method)
Last changed    : 29/6/2024 4:48:54 pm by SYSTEM
                  (modified after loading)
Additional Info : Peak(s) manually integrated
```

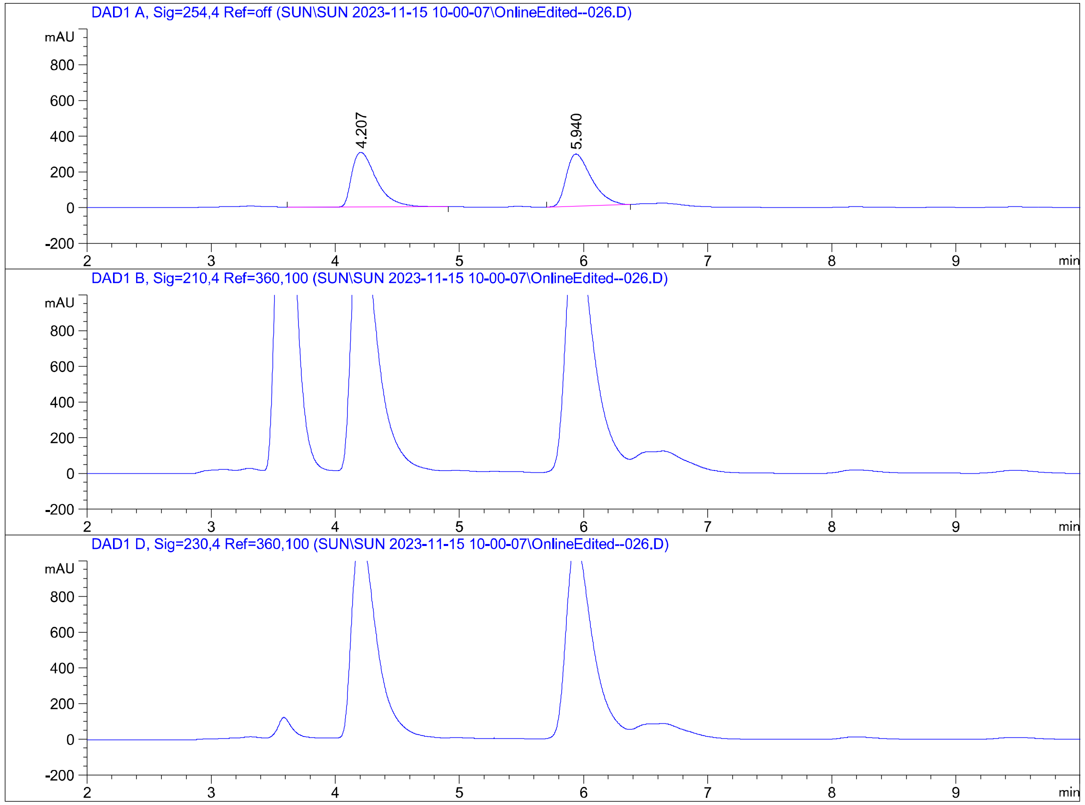

Data File C:\Users\P...\ChemStation\1\Data\SUN\SUN 2023-11-15 10-00-07\OnlineEdited--026.D
Sample Name: zmh-4-169a-ad

===
Area Percent Report
===

```
Sorted By             :      Signal
Multiplier            :      1.0000
Dilution              :      1.0000
Use Multiplier & Dilution Factor with ISTDs
```

Signal 1: DAD1 A, Sig=254,4 Ref=off

Peak #	RetTime [min]	Type	Width [min]	Area [mAU*s]	Height [mAU]	Area %
1	4.207	BB	0.2148	4302.51221	306.46497	50.8206
2	5.940	BB	0.2179	4163.56738	292.71170	49.1794

Totals : 8466.07959 599.17667

Signal 2: DAD1 B, Sig=210,4 Ref=360,100

Signal 3: DAD1 D, Sig=230,4 Ref=360,100

===
*** End of Report ***

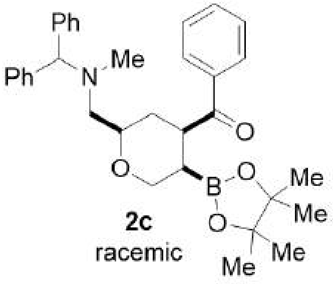

2c
racemic

===

Acq. Operator : SYSTEM　Seq. Line : 4
Sample Operator : SYSTEM
Acq. Instrument : HPLC　Location : P1-A-01
Injection Date : 18/5/2024 3:12:03 pm　Inj : 1
Inj Volume : 2.000 µl
Different Inj Volume from Sample Entry! Actual Inj Volume : 10.000 µl
Acq. Method : C:\Users\Public\Documents\ChemStation\1\Data\SUN\SUN 2024-05-18 14-43-37\AD3-10-20.M
Last changed : 15/8/2022 10:21:32 pm by SYSTEM
Analysis Method : C:\Users\Public\Documents\ChemStation\1\Data\SUN\SUN 2024-05-18 14-43-37\AD3-10-20.M (Sequence Method)
Last changed : 29/6/2024 4:47:04 pm by SYSTEM
(modified after loading)
Additional Info : Peak(s) manually integrated

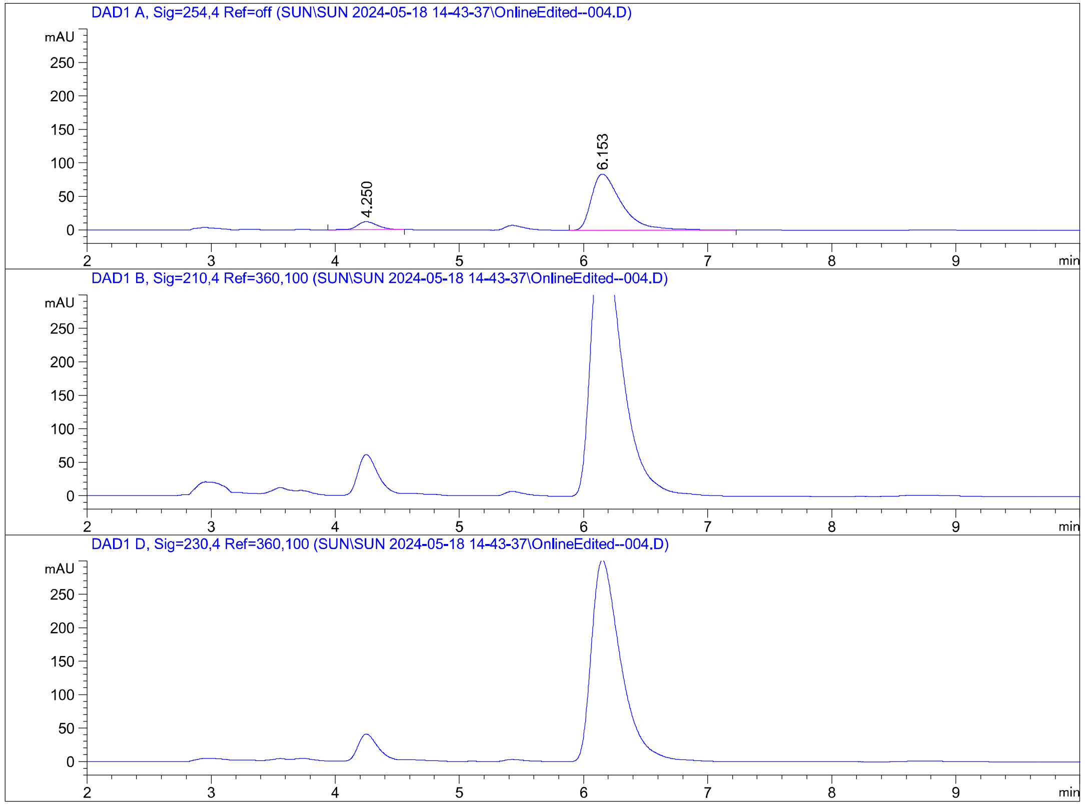

Data File C:\Users\P...\ChemStation\1\Data\SUN\SUN 2024-05-18 14-43-37\OnlineEdited--004.D
Sample Name: zmh-5-180q-re

===

Area Percent Report

===

Sorted By : Signal
Multiplier : 1.0000
Dilution : 1.0000
Use Multiplier & Dilution Factor with ISTDs

Signal 1: DAD1 A, Sig=254,4 Ref=off

Peak #	RetTime [min]	Type	Width [min]	Area [mAU*s]	Height [mAU]	Area %
1	4.250	BB	0.1681	134.81511	11.99860	8.7235
2	6.153	BB	0.2513	1410.60852	83.84942	91.2765

Totals : 1545.42363　95.84802

Signal 2: DAD1 B, Sig=210,4 Ref=360,100

Signal 3: DAD1 D, Sig=230,4 Ref=360,100

===

*** End of Report ***

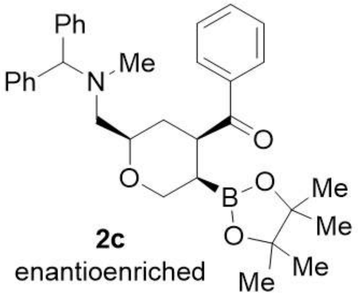

2c
enantioenriched

===

Acq. Operator : SYSTEM   Seq. Line : 2
Sample Operator : SYSTEM
Acq. Instrument : HPLC   Location : P1-F-01
Injection Date : 26/4/2024 9:41:45 pm   Inj : 1
Inj Volume : 2.000 µl
Different Inj Volume from Sample Entry! Actual Inj Volume : 10.000 µl
Acq. Method : C:\Users\Public\Documents\ChemStation\1\Data\SUN\SUN 2024-04-26 21-28-36\AD3-10-20.M
Last changed : 15/8/2022 10:21:32 pm by SYSTEM
Analysis Method : C:\Users\Public\Documents\ChemStation\1\Data\SUN\SUN 2024-04-26 21-28-36\AD3-10-20.M (Sequence Method)
Last changed : 29/6/2024 3:58:51 pm by SYSTEM
(modified after loading)
Additional Info : Peak(s) manually integrated

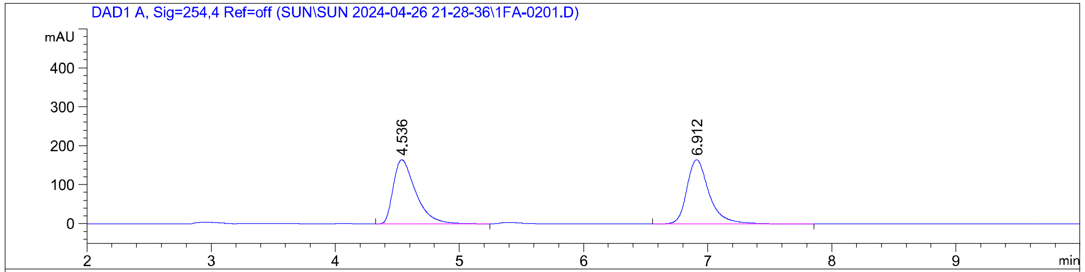

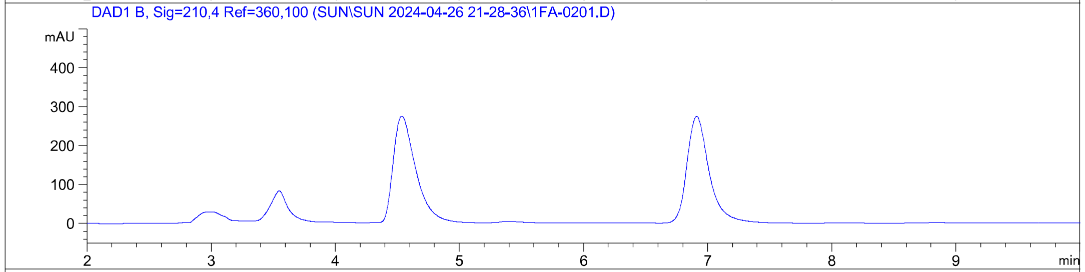

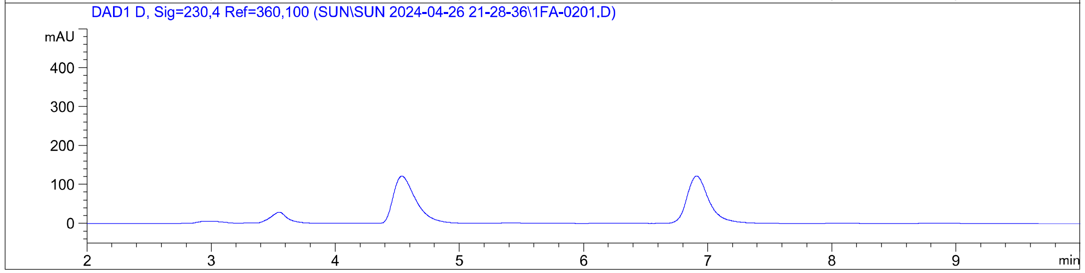

Data File C:\Users\P...Documents\ChemStation\1\Data\SUN\SUN 2024-04-26 21-28-36\1FA-0201.D
Sample Name: zmh-5-157c-rac

===

Area Percent Report

===

Sorted By : Signal
Multiplier : 1.0000
Dilution : 1.0000
Use Multiplier & Dilution Factor with ISTDs

Signal 1: DAD1 A, Sig=254,4 Ref=off

Peak #	RetTime [min]	Type	Width [min]	Area [mAU*s]	Height [mAU]	Area %
1	4.536	BB	0.1896	2058.08911	164.30684	49.9575
2	6.912	BB	0.1898	2061.59399	164.98383	50.0425

Totals : 4119.68311 329.29066

Signal 2: DAD1 B, Sig=210,4 Ref=360,100

Signal 3: DAD1 D, Sig=230,4 Ref=360,100

===

*** End of Report ***

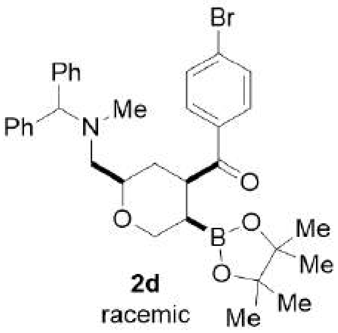

2d
racemic

===

Acq. Operator : SYSTEM   Seq. Line : 19
Sample Operator : SYSTEM
Acq. Instrument : HPLC   Location : P1-F-01
Injection Date : 16/5/2024 12:54:47 am   Inj : 1
Inj Volume : 2.000 µl
Different Inj Volume from Sample Entry! Actual Inj Volume : 10.000 µl
Acq. Method : C:\Users\Public\Documents\ChemStation\1\Data\SUN\SUN 2024-05-15 19-37-48\AD3-10-20.M
Last changed : 15/8/2022 10:21:32 pm by SYSTEM
Analysis Method : C:\Users\Public\Documents\ChemStation\1\Data\SUN\SUN 2024-05-15 19-37-48\AD3-10-20.M (Sequence Method)
Last changed : 29/6/2024 4:08:23 pm by SYSTEM
(modified after loading)
Additional Info : Peak(s) manually integrated

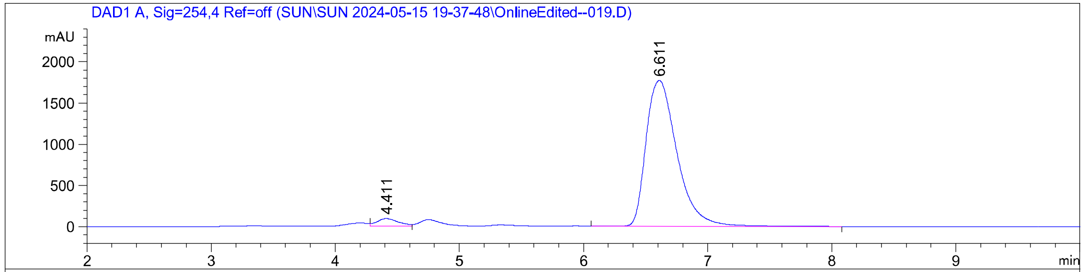

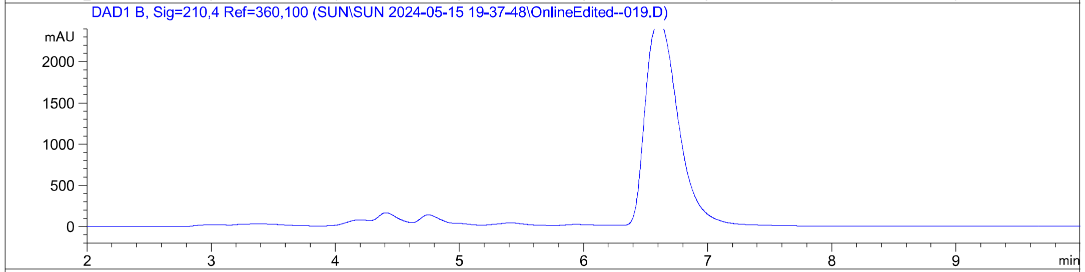

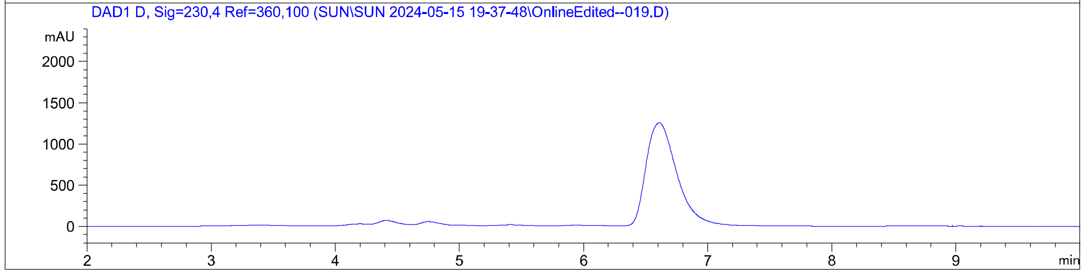

Data File C:\Users\P...\ChemStation\1\Data\SUN\SUN 2024-05-15 19-37-48\OnlineEdited--019.D
Sample Name: zmh-5-180k

===

Area Percent Report

===

Sorted By : Signal
Multiplier : 1.0000
Dilution : 1.0000
Use Multiplier & Dilution Factor with ISTDs

Signal 1: DAD1 A, Sig=254,4 Ref=off

Peak #	RetTime [min]	Type	Width [min]	Area [mAU*s]	Height [mAU]	Area %
1	4.411	VV	0.1816	1154.73474	93.49410	3.6530
2	6.611	VB R	0.2680	3.04560e4	1767.82983	96.3470

Totals : 3.16108e4 1861.32394

Signal 2: DAD1 B, Sig=210,4 Ref=360,100

Signal 3: DAD1 D, Sig=230,4 Ref=360,100

===

*** End of Report ***

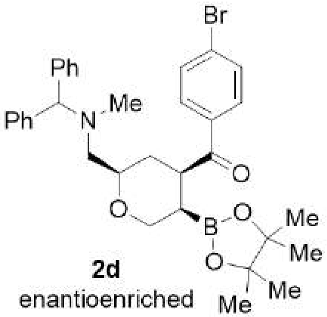

2d
enantioenriched

===

Acq. Operator : SYSTEM   Seq. Line : 3
Sample Operator : SYSTEM
Acq. Instrument : HPLC   Location : P1-F-02
Injection Date : 26/4/2024 10:02:51 pm   Inj : 1
Inj Volume : 2.000 µl
Different Inj Volume from Sample Entry! Actual Inj Volume : 10.000 µl
Acq. Method : C:\Users\Public\Documents\ChemStation\1\Data\SUN\SUN 2024-04-26 21-28-36\AD3-10-20.M
Last changed : 15/8/2022 10:21:32 pm by SYSTEM
Analysis Method : C:\Users\Public\Documents\ChemStation\1\Data\SUN\SUN 2024-04-26 21-28-36\AD3-10-20.M (Sequence Method)
Last changed : 29/6/2024 4:36:28 pm by SYSTEM
(modified after loading)
Additional Info : Peak(s) manually integrated

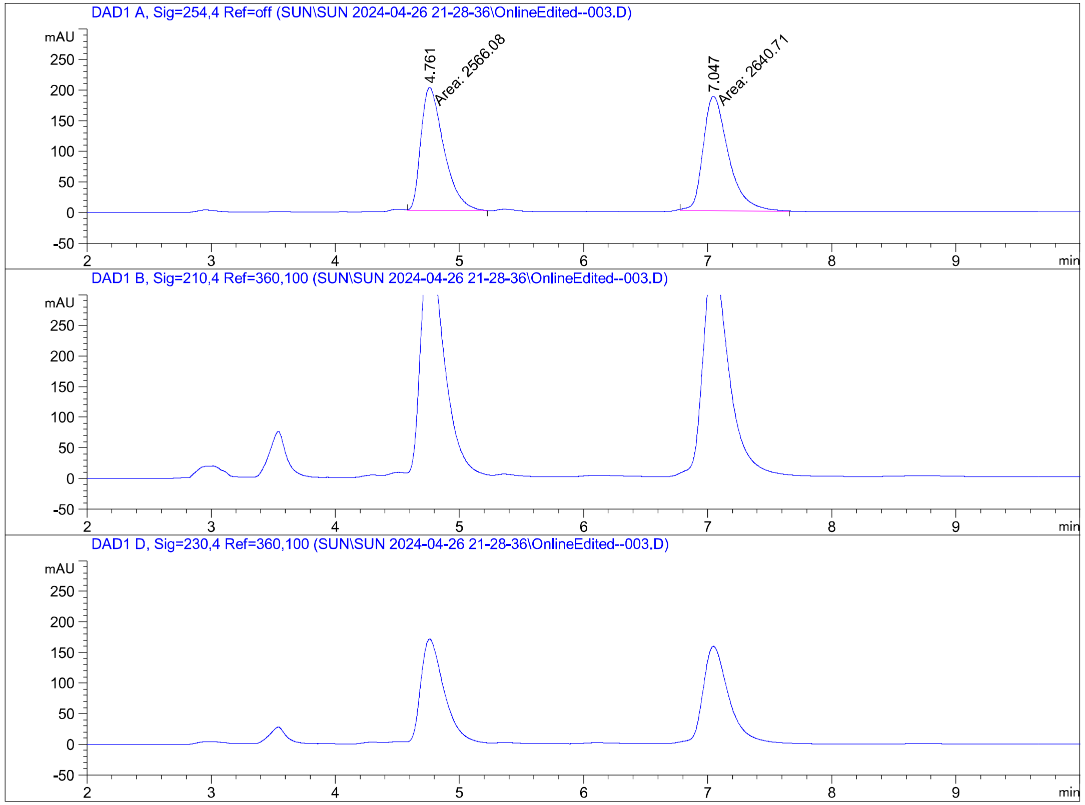

Data File C:\Users\P...\ChemStation\1\Data\SUN\SUN 2024-04-26 21-28-36\OnlineEdited--003.D
Sample Name: zmh-5-157d-rac

===
Area Percent Report
===

Sorted By : Signal
Multiplier : 1.0000
Dilution : 1.0000
Use Multiplier & Dilution Factor with ISTDs

Signal 1: DAD1 A, Sig=254,4 Ref=off

Peak #	RetTime [min]	Type	Width [min]	Area [mAU*s]	Height [mAU]	Area %
1	4.761	MM	0.2133	2566.08325	200.54152	49.2833
2	7.047	MM	0.2359	2640.71387	186.54597	50.7167
Totals :				5206.79712	387.08749	

Signal 2: DAD1 B, Sig=210,4 Ref=360,100

Signal 3: DAD1 D, Sig=230,4 Ref=360,100

===
*** End of Report ***

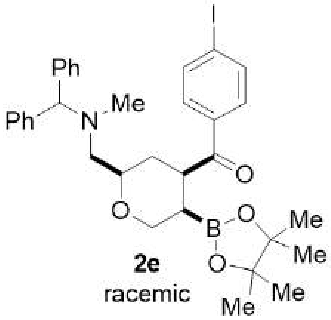

2e
racemic

===

Acq. Operator : SYSTEM   Seq. Line : 4
Sample Operator : SYSTEM
Acq. Instrument : HPLC   Location : P1-C-02
Injection Date : 16/5/2024 7:15:20 pm   Inj : 1
Inj Volume : 2.000 µl
Different Inj Volume from Sample Entry! Actual Inj Volume : 10.000 µl
Acq. Method : C:\Users\Public\Documents\ChemStation\1\Data\SUN\SUN 2024-05-16 18-00-07\AD3-10-20.M
Last changed : 15/8/2022 10:21:32 pm by SYSTEM
Analysis Method : C:\Users\Public\Documents\ChemStation\1\Data\SUN\SUN 2024-05-16 18-00-07\AD3-10-20.M (Sequence Method)
Last changed : 29/6/2024 4:39:49 pm by SYSTEM
(modified after loading)
Additional Info : Peak(s) manually integrated

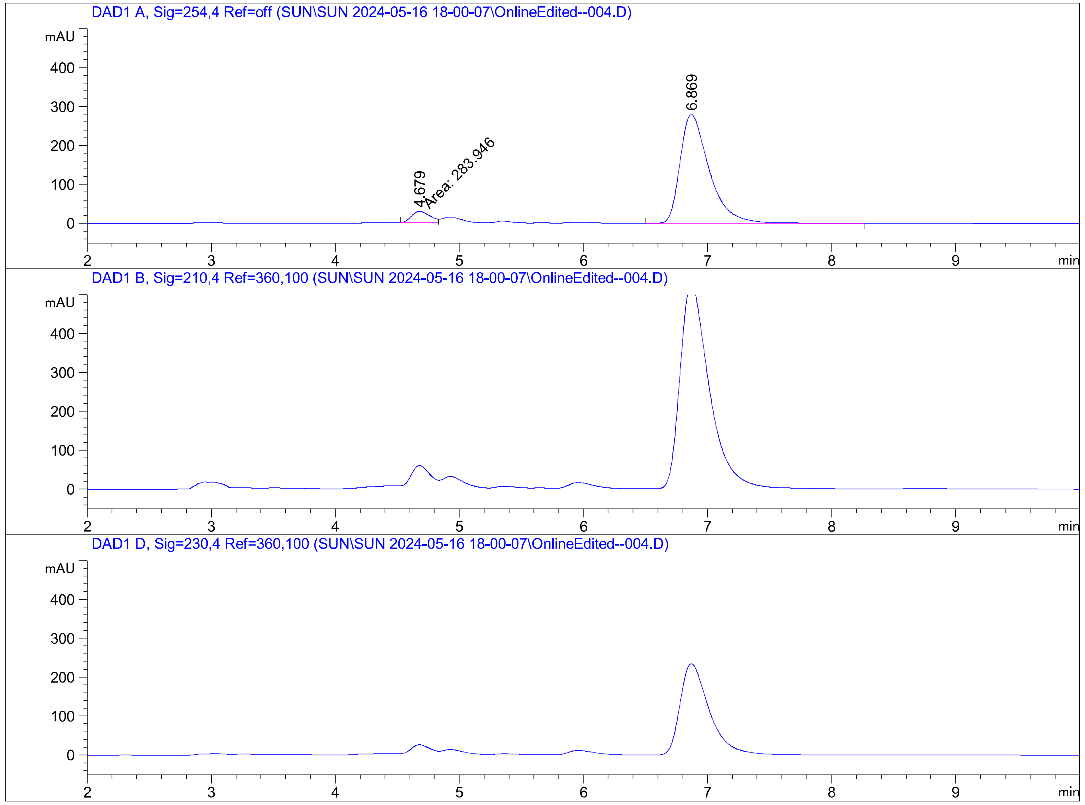

Data File C:\Users\P...\ChemStation\1\Data\SUN\SUN 2024-05-16 18-00-07\OnlineEdited--004.D
Sample Name: zmh-5-180p

===

Area Percent Report

===

Sorted By : Signal
Multiplier : 1.0000
Dilution : 1.0000
Use Multiplier & Dilution Factor with ISTDs

Signal 1: DAD1 A, Sig=254,4 Ref=off

Peak #	RetTime [min]	Type	Width [min]	Area [mAU*s]	Height [mAU]	Area %
1	4.679	MF	0.1697	283.94620	27.89430	5.8348
2	6.869	BB	0.2479	4582.44385	279.26749	94.1652

Totals : 4866.39005 307.16179

Signal 2: DAD1 B, Sig=210,4 Ref=360,100

Signal 3: DAD1 D, Sig=230,4 Ref=360,100

===

*** End of Report ***

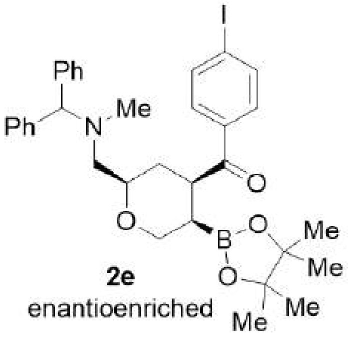

2e
enantioenriched

Data File C:\Users\P...\ChemStation\1\Data\SUN\SUN 2024-04-26 21-28-36\OnlineEdited--005.D
Sample Name: zmh-5-157f-rac

===

Acq. Operator : SYSTEM Seq. Line : 5
Sample Operator : SYSTEM
Acq. Instrument : HPLC Location : P1-F-04
Injection Date : 26/4/2024 10:45:13 pm Inj : 1
Inj Volume : 2.000 µl
Different Inj Volume from Sample Entry! Actual Inj Volume : 10.000 µl
Acq. Method : C:\Users\Public\Documents\ChemStation\1\Data\SUN\SUN 2024-04-26 21-28-36 \AD3-10-20.M
Last changed : 15/8/2022 10:21:32 pm by SYSTEM
Analysis Method : C:\Users\Public\Documents\ChemStation\1\Data\SUN\SUN 2024-04-26 21-28-36 \AD3-10-20.M (Sequence Method)
Last changed : 29/6/2024 4:25:20 pm by SYSTEM
(modified after loading)
Additional Info : Peak(s) manually integrated

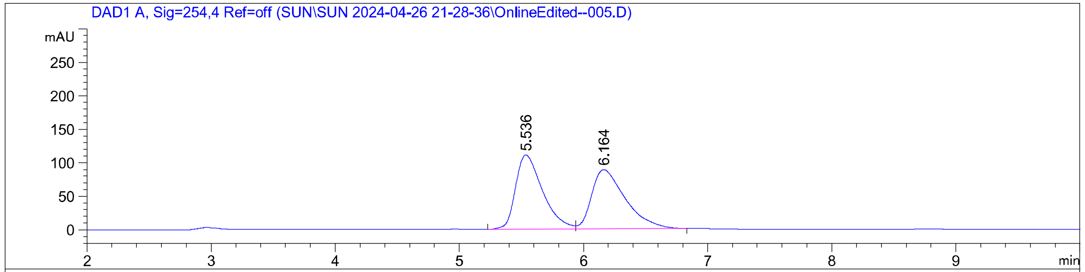

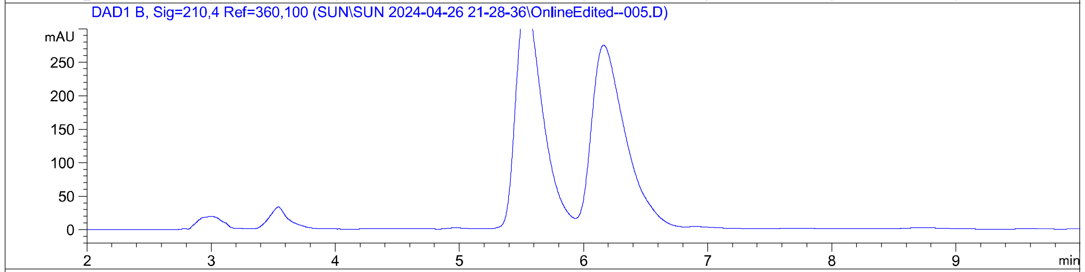

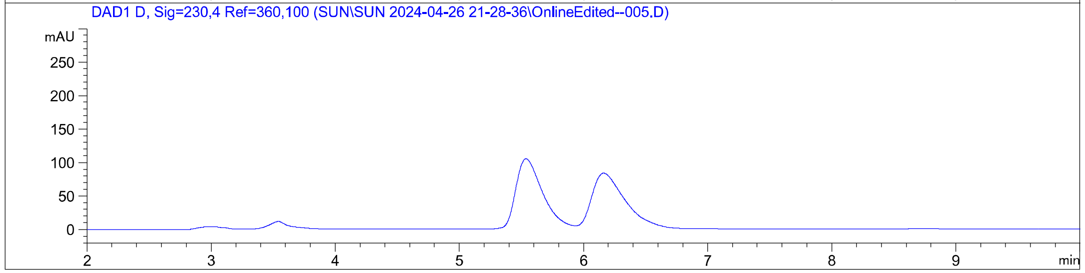

Data File C:\Users\P...\ChemStation\1\Data\SUN\SUN 2024-04-26 21-28-36\OnlineEdited--005.D
Sample Name: zmh-5-157f-rac

===

Area Percent Report

===

Sorted By : Signal
Multiplier : 1.0000
Dilution : 1.0000
Use Multiplier & Dilution Factor with ISTDs

Signal 1: DAD1 A, Sig=254,4 Ref=off

Peak #	RetTime [min]	Type	Width [min]	Area [mAU*s]	Height [mAU]	Area %
1	5.536	BV	0.2285	1681.40552	110.85321	50.2385
2	6.164	VB	0.2844	1665.44397	88.37688	49.7615

Totals : 3346.84949 199.23009

Signal 2: DAD1 B, Sig=210,4 Ref=360,100

Signal 3: DAD1 D, Sig=230,4 Ref=360,100

===

*** End of Report ***

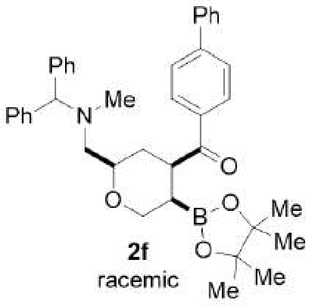

2f
racemic

===

Acq. Operator : SYSTEM Seq. Line : 10
Sample Operator : SYSTEM
Acq. Instrument : HPLC Location : P1-C-04
Injection Date : 16/5/2024 10:56:16 pm Inj : 1
Inj Volume : 2.000 µl
Different Inj Volume from Sample Entry! Actual Inj Volume : 10.000 µl
Acq. Method : C:\Users\Public\Documents\ChemStation\1\Data\SUN\SUN 2024-05-16 20-03-03\AD3-10-20.M
Last changed : 15/8/2022 10:21:32 pm by SYSTEM
Analysis Method : C:\Users\Public\Documents\ChemStation\1\Data\SUN\SUN 2024-05-16 20-03-03\AD3-10-20.M (Sequence Method)
Last changed : 29/6/2024 4:28:08 pm by SYSTEM
(modified after loading)
Additional Info : Peak(s) manually integrated

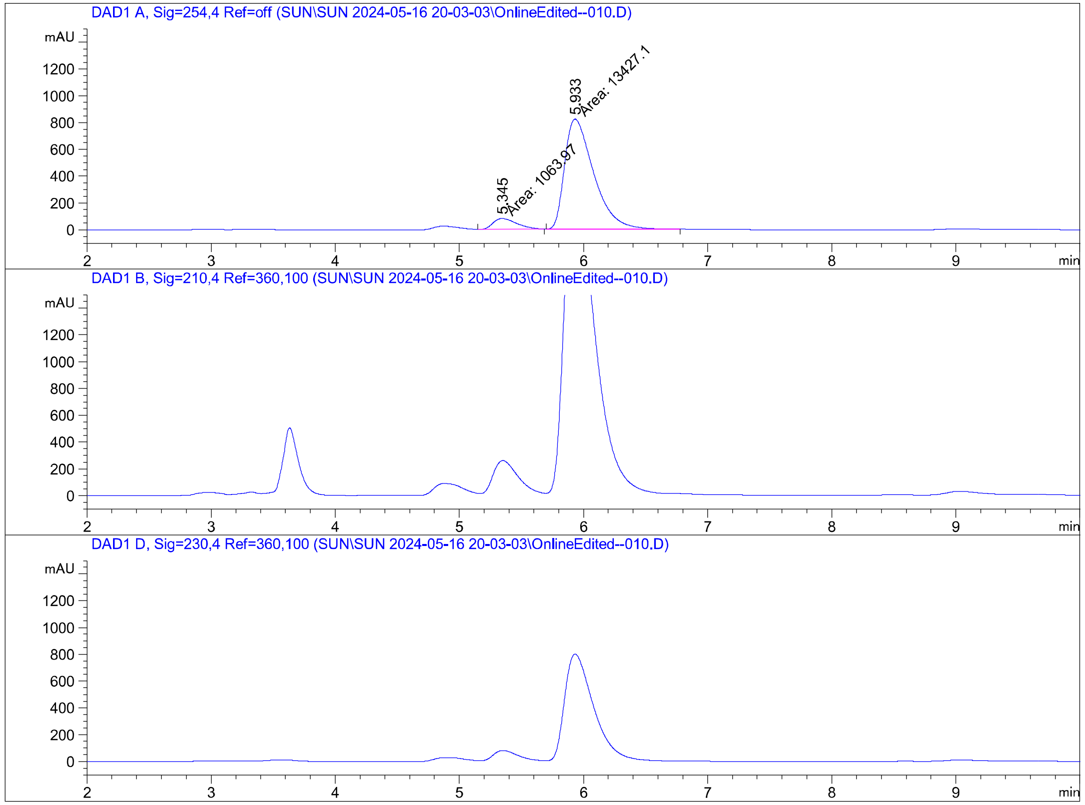

Data File C:\Users\P...\ChemStation\1\Data\SUN\SUN 2024-05-16 20-03-03\OnlineEdited--010.D
Sample Name: zmh-5-180n

===

Area Percent Report

===

Sorted By : Signal
Multiplier : 1.0000
Dilution : 1.0000
Use Multiplier & Dilution Factor with ISTDs

Signal 1: DAD1 A, Sig=254,4 Ref=off

Peak #	RetTime [min]	Type	Width [min]	Area [mAU*s]	Height [mAU]	Area %
1	5.345	MM	0.2194	1063.96948	80.82060	7.3422
2	5.933	MM	0.2725	1.34271e4	821.36426	92.6578

Totals : 1.44911e4 902.18486

Signal 2: DAD1 B, Sig=210,4 Ref=360,100

Signal 3: DAD1 D, Sig=230,4 Ref=360,100

===

*** End of Report ***

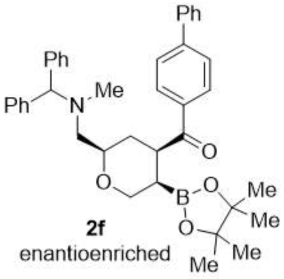

2f
enantioenriched

===

Acq. Operator : SYSTEM   Seq. Line : 4
Sample Operator : SYSTEM
Acq. Instrument : HPLC   Location : P1-F-03
Injection Date : 26/4/2024 10:23:55 pm   Inj : 1
Inj Volume : 2.000 µl
Different Inj Volume from Sample Entry! Actual Inj Volume : 10.000 µl
Acq. Method : C:\Users\Public\Documents\ChemStation\1\Data\SUN\SUN 2024-04-26 21-28-36\AD3-10-20.M
Last changed : 15/8/2022 10:21:32 pm by SYSTEM
Analysis Method : C:\Users\Public\Documents\ChemStation\1\Data\SUN\SUN 2024-04-26 21-28-36\AD3-10-20.M (Sequence Method)
Last changed : 29/6/2024 4:11:05 pm by SYSTEM
(modified after loading)
Additional Info : Peak(s) manually integrated

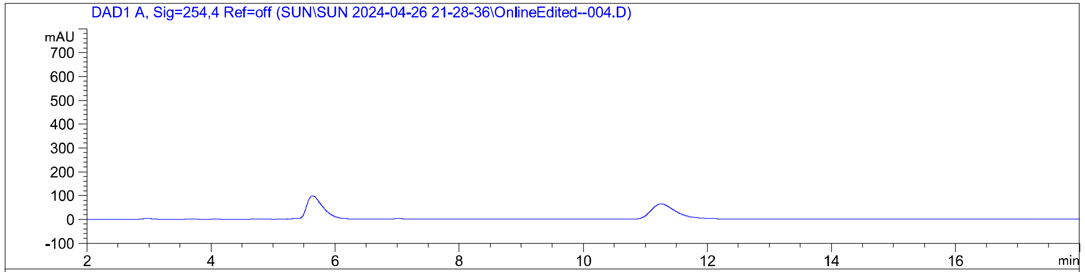

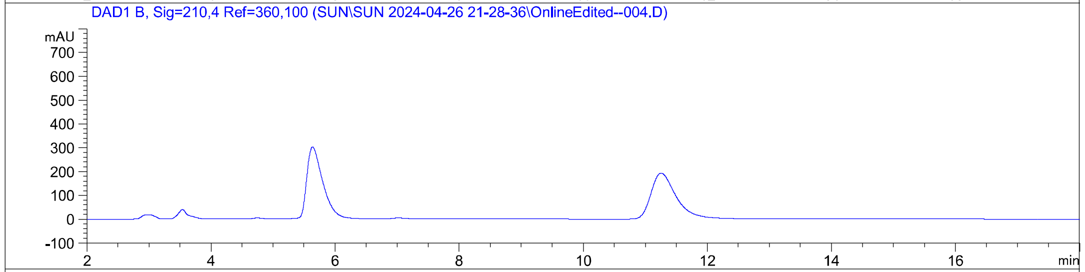

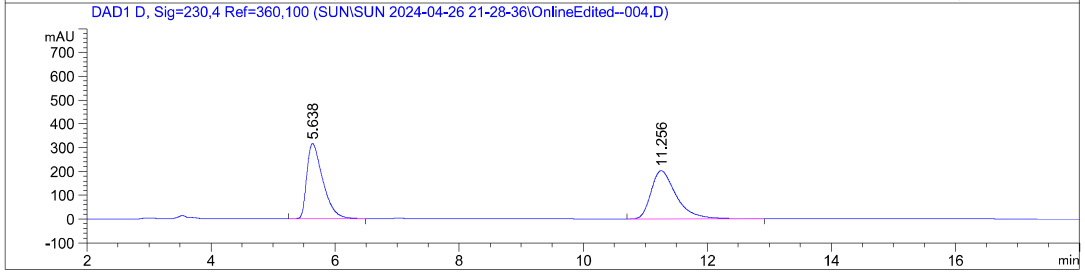

Data File C:\Users\P...\ChemStation\1\Data\SUN\SUN 2024-04-26 21-28-36\OnlineEdited--004.D
Sample Name: zmh-5-157e-rac

===

Area Percent Report

===

Sorted By : Signal
Multiplier : 1.0000
Dilution : 1.0000
Use Multiplier & Dilution Factor with ISTDs

Signal 1: DAD1 A, Sig=254,4 Ref=off

Signal 2: DAD1 B, Sig=210,4 Ref=360,100

Signal 3: DAD1 D, Sig=230,4 Ref=360,100

Peak #	RetTime [min]	Type	Width [min]	Area [mAU*s]	Height [mAU]	Area %
1	5.638	BB	0.2670	5636.18750	316.20459	49.7237
2	11.256	BB	0.4213	5698.83252	201.88258	50.2763

Totals : 1.13350e4 518.08717

===

*** End of Report ***

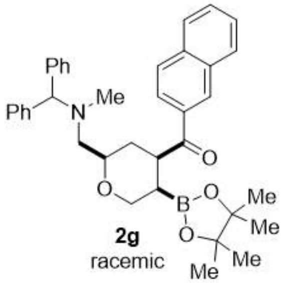

2g
racemic

===

Acq. Operator : SYSTEM Seq. Line : 20
Sample Operator : SYSTEM
Acq. Instrument : HPLC Location : P1-F-02
Injection Date : 16/5/2024 1:16:04 am Inj : 1
Inj Volume : 2.000 µl
Different Inj Volume from Sample Entry! Actual Inj Volume : 10.000 µl
Acq. Method : C:\Users\Public\Documents\ChemStation\1\Data\SUN\SUN 2024-05-15 19-37-48\AD3-10-20.M
Last changed : 15/8/2022 10:21:32 pm by SYSTEM
Analysis Method : C:\Users\Public\Documents\ChemStation\1\Data\SUN\SUN 2024-05-15 19-37-48\AD3-10-20.M (Sequence Method)
Last changed : 29/6/2024 4:13:25 pm by SYSTEM
(modified after loading)
Additional Info : Peak(s) manually integrated

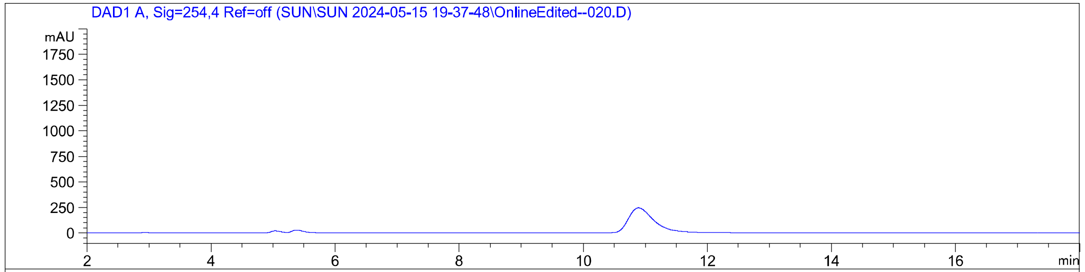

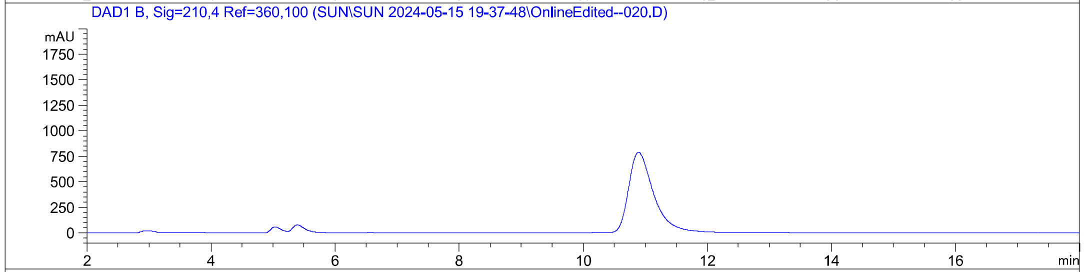

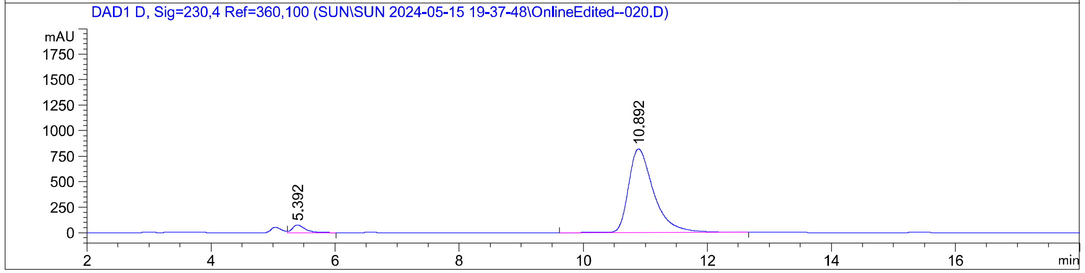

Data File C:\Users\P...\ChemStation\1\Data\SUN\SUN 2024-05-15 19-37-48\OnlineEdited--020.D
Sample Name: zmh-5-180l

===

Area Percent Report

===

Sorted By : Signal
Multiplier : 1.0000
Dilution : 1.0000
Use Multiplier & Dilution Factor with ISTDs

Signal 1: DAD1 A, Sig=254,4 Ref=off

Signal 2: DAD1 B, Sig=210,4 Ref=360,100

Signal 3: DAD1 D, Sig=230,4 Ref=360,100

Peak #	RetTime [min]	Type	Width [min]	Area [mAU*s]	Height [mAU]	Area %
1	5.392	VB	0.2096	1040.76221	74.89169	4.3337
2	10.892	BB	0.4275	2.29746e4	818.50403	95.6663

Totals : 2.40154e4 893.39572

===

*** End of Report ***

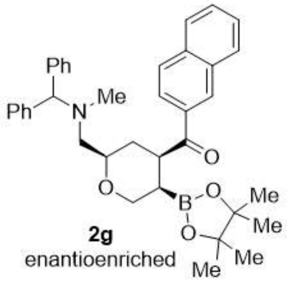

2g
enantioenriched

===

Acq. Operator : SYSTEM Seq. Line : 6
Sample Operator : SYSTEM
Acq. Instrument : HPLC Location : P1-F-05
Injection Date : 26/4/2024 11:06:18 pm Inj : 1
Inj Volume : 2.000 µl
Different Inj Volume from Sample Entry! Actual Inj Volume : 10.000 µl
Acq. Method : C:\Users\Public\Documents\ChemStation\1\Data\SUN\SUN 2024-04-26 21-28-36\AD3-10-20.M
Last changed : 15/8/2022 10:21:32 pm by SYSTEM
Analysis Method : C:\Users\Public\Documents\ChemStation\1\Data\SUN\SUN 2024-04-26 21-28-36\AD3-10-20.M (Sequence Method)
Last changed : 29/6/2024 4:31:41 pm by SYSTEM
(modified after loading)
Additional Info : Peak(s) manually integrated

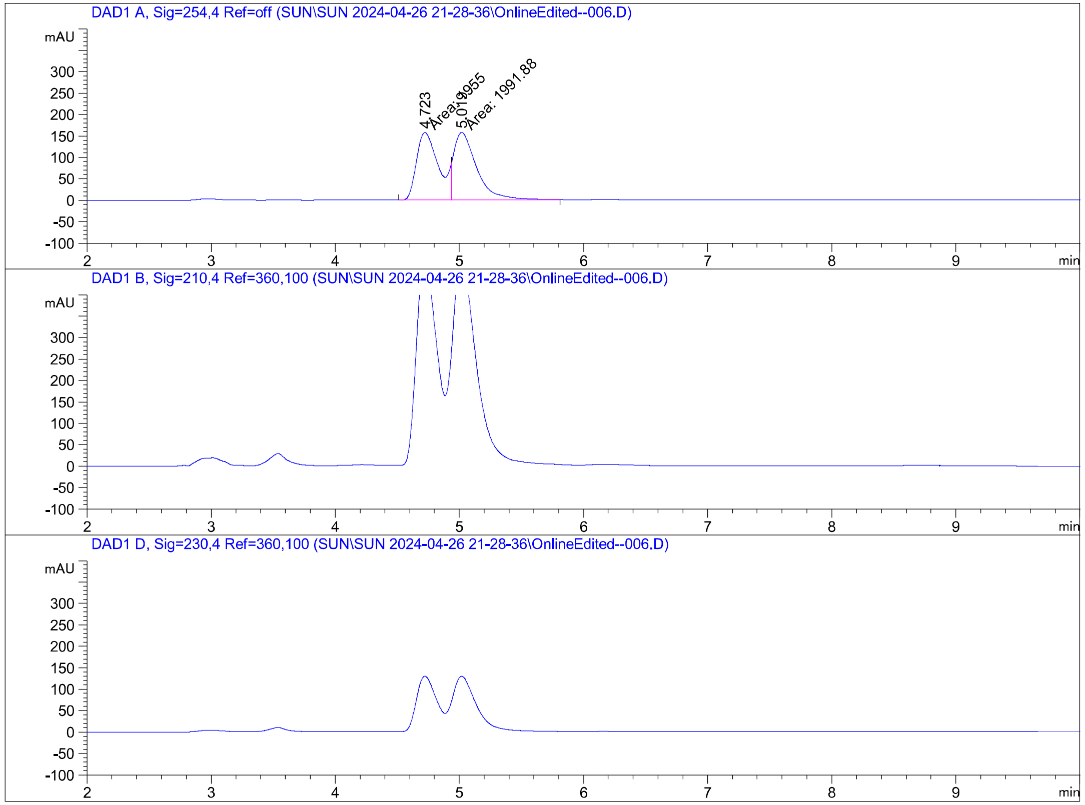

Data File C:\Users\P...\ChemStation\1\Data\SUN\SUN 2024-04-26 21-28-36\OnlineEdited--006.D
Sample Name: zmh-5-157g-rac

===

Area Percent Report

===

Sorted By : Signal
Multiplier : 1.0000
Dilution : 1.0000
Use Multiplier & Dilution Factor with ISTDs

Signal 1: DAD1 A, Sig=254,4 Ref=off

Peak #	RetTime [min]	Type	Width [min]	Area [mAU*s]	Height [mAU]	Area %
1	4.723	MF	0.2070	1954.99756	157.43814	49.5328
2	5.019	FM	0.2107	1991.87976	157.55223	50.4672
Totals :				3946.87732	314.99037	

Signal 2: DAD1 B, Sig=210,4 Ref=360,100

Signal 3: DAD1 D, Sig=230,4 Ref=360,100

===

*** End of Report ***

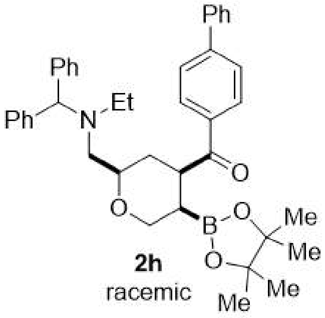

2h
racemic

===

Acq. Operator : SYSTEM Seq. Line : 5
Sample Operator : SYSTEM
Acq. Instrument : HPLC Location : P1-C-03
Injection Date : 16/5/2024 7:36:23 pm Inj : 1
Inj Volume : 2.000 µl
Different Inj Volume from Sample Entry! Actual Inj Volume : 10.000 µl
Acq. Method : C:\Users\Public\Documents\ChemStation\1\Data\SUN\SUN 2024-05-16 18-00-07 \AD3-10-20.M
Last changed : 15/8/2022 10:21:32 pm by SYSTEM
Analysis Method : C:\Users\Public\Documents\ChemStation\1\Data\SUN\SUN 2024-05-16 18-00-07 \AD3-10-20.M (Sequence Method)
Last changed : 29/6/2024 4:34:30 pm by SYSTEM
(modified after loading)
Additional Info : Peak(s) manually integrated

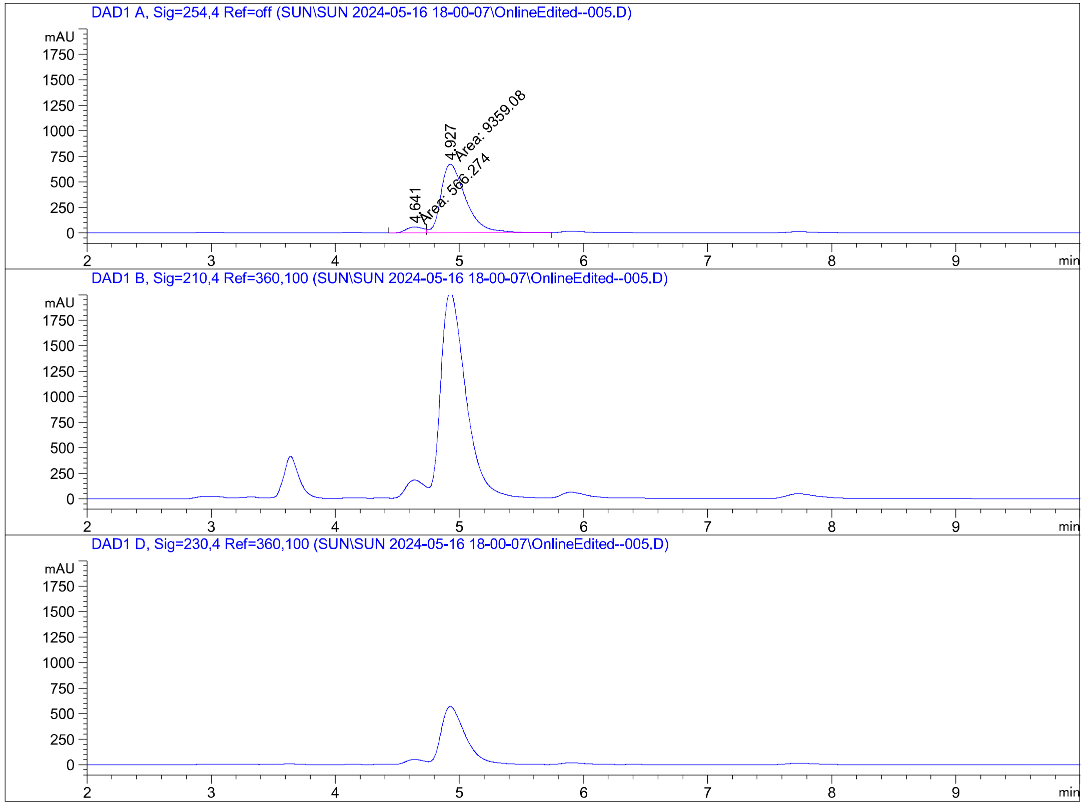

Data File C:\Users\P...\ChemStation\1\Data\SUN\SUN 2024-05-16 18-00-07\OnlineEdited--005.D
Sample Name: zmh-5-180o

===

Area Percent Report

===

Sorted By : Signal
Multiplier : 1.0000
Dilution : 1.0000
Use Multiplier & Dilution Factor with ISTDs

Signal 1: DAD1 A, Sig=254,4 Ref=off

Peak #	RetTime [min]	Type	Width [min]	Area [mAU*s]	Height [mAU]	Area %
1	4.641	MF	0.1632	566.27417	57.83654	5.7053
2	4.927	FM	0.2317	9359.08203	673.33551	94.2947

Totals : 9925.35620 731.17205

Signal 2: DAD1 B, Sig=210,4 Ref=360,100

Signal 3: DAD1 D, Sig=230,4 Ref=360,100

===

*** End of Report ***

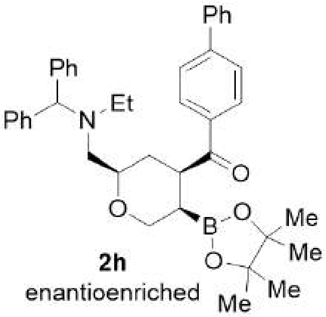

2h
enantioenriched

===

Acq. Operator : SYSTEM Seq. Line : 35
Sample Operator : SYSTEM
Acq. Instrument : HPLC Location : P1-E-09
Injection Date : 11/5/2024 6:49:33 pm Inj : 1
Inj Volume : 2.000 µl
Different Inj Volume from Sample Entry! Actual Inj Volume : 10.000 µl
Acq. Method : C:\Users\Public\Documents\ChemStation\1\Data\SUN\SUN 2024-05-11 09-32-52 \AD3-10-20.M
Last changed : 15/8/2022 10:21:32 pm by SYSTEM
Analysis Method : C:\Users\Public\Documents\ChemStation\1\Data\SUN\SUN 2024-05-11 09-32-52 \AD3-10-20.M (Sequence Method)
Last changed : 29/6/2024 2:55:03 pm by SYSTEM
(modified after loading)
Additional Info : Peak(s) manually integrated

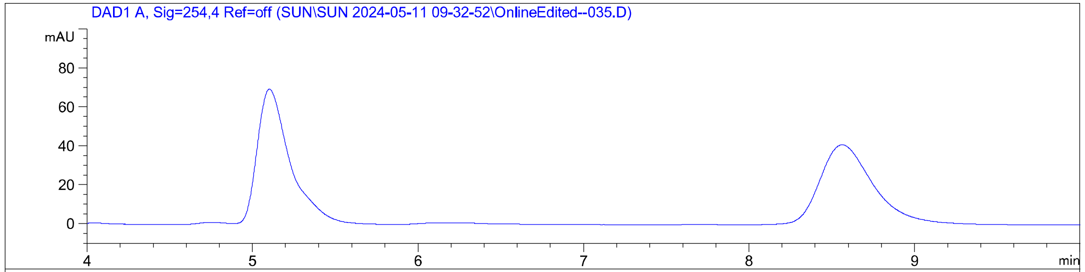

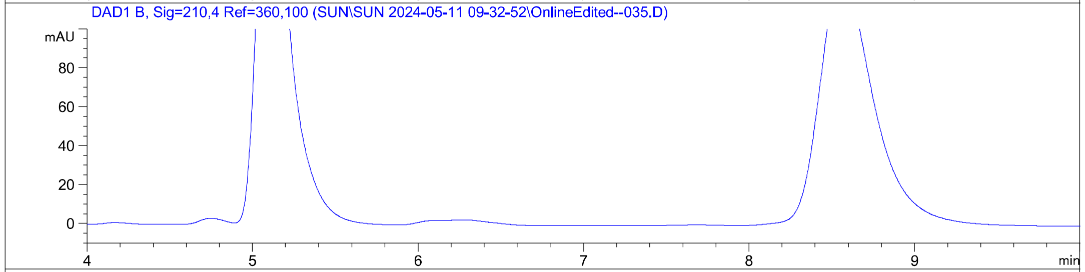

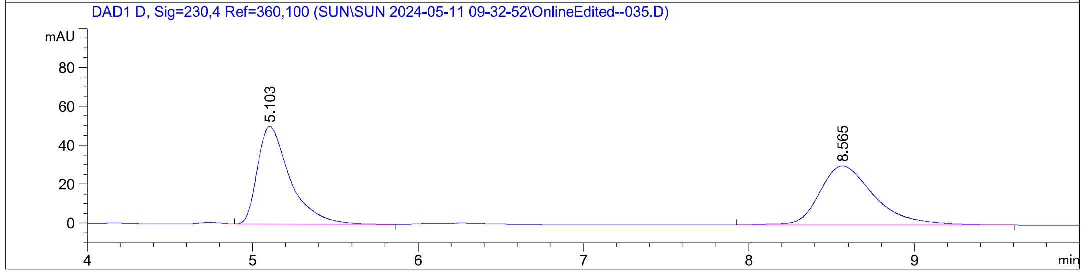

Data File C:\Users\P...\ChemStation\1\Data\SUN\SUN 2024-05-11 09-32-52\OnlineEdited--035.D
Sample Name: zmh-5-177e-rac

===

Area Percent Report

===

Sorted By : Signal
Multiplier : 1.0000
Dilution : 1.0000
Use Multiplier & Dilution Factor with ISTDs

Signal 1: DAD1 A, Sig=254,4 Ref=off

Signal 2: DAD1 B, Sig=210,4 Ref=360,100

Signal 3: DAD1 D, Sig=230,4 Ref=360,100

Peak #	RetTime [min]	Type	Width [min]	Area [mAU*s]	Height [mAU]	Area %
1	5.103	BB	0.2059	684.20807	50.06925	49.6969
2	8.565	BB	0.3339	692.55347	30.22166	50.3031

Totals : 1376.76154 80.29090

===

*** End of Report ***

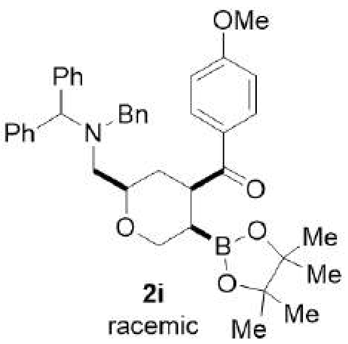

2i
racemic

===

Acq. Operator : SYSTEM Seq. Line : 2
Sample Operator : SYSTEM
Acq. Instrument : HPLC Location : P1-F-01
Injection Date : 14/5/2024 3:33:09 pm Inj : 1
Inj Volume : 2.000 µl
Different Inj Volume from Sample Entry! Actual Inj Volume : 10.000 µl
Acq. Method : C:\Users\Public\Documents\ChemStation\1\Data\SUN\SUN 2024-05-14 15-20-06 \AD3-10-20.M
Last changed : 15/8/2022 10:21:32 pm by SYSTEM
Analysis Method : C:\Users\Public\Documents\ChemStation\1\Data\SUN\SUN 2024-05-14 15-20-06 \AD3-10-20.M (Sequence Method)
Last changed : 29/6/2024 2:57:20 pm by SYSTEM
(modified after loading)
Additional Info : Peak(s) manually integrated

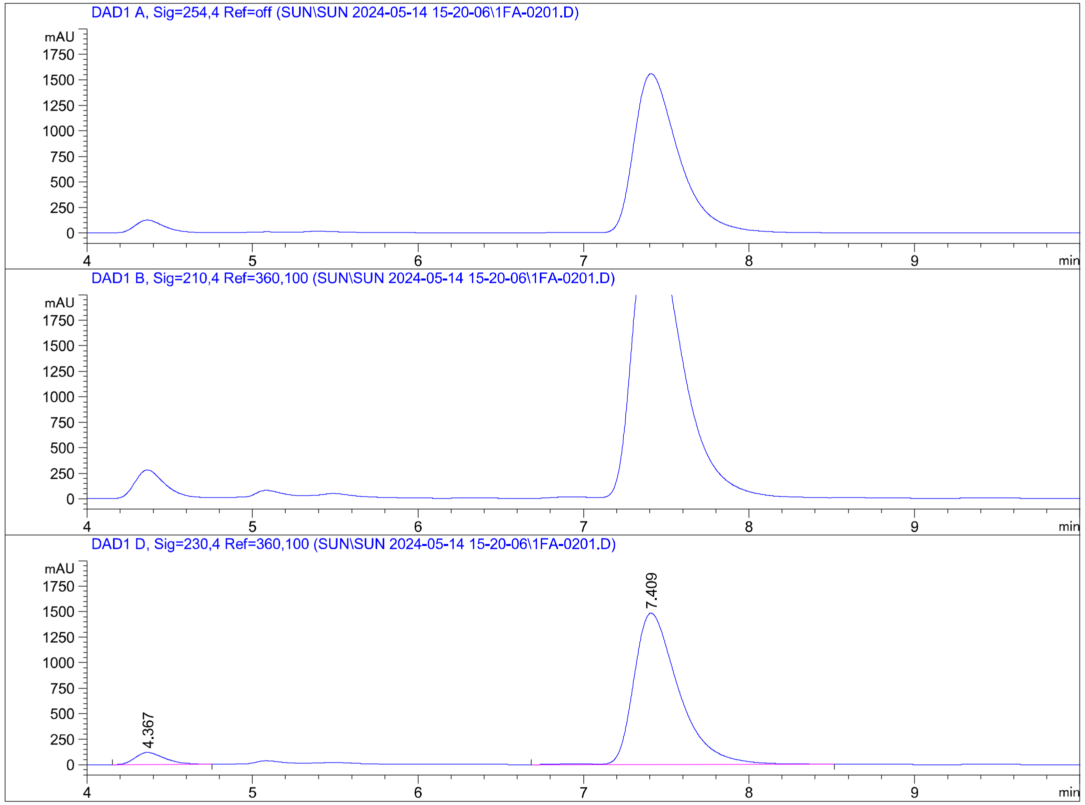

Data File C:\Users\P...Documents\ChemStation\1\Data\SUN\SUN 2024-05-14 15-20-06\1FA-0201.D
Sample Name: zmh-5-180b

===

Area Percent Report

===

Sorted By : Signal
Multiplier : 1.0000
Dilution : 1.0000
Use Multiplier & Dilution Factor with ISTDs

Signal 1: DAD1 A, Sig=254,4 Ref=off

Signal 2: DAD1 B, Sig=210,4 Ref=360,100

Signal 3: DAD1 D, Sig=230,4 Ref=360,100

Peak #	RetTime [min]	Type	Width [min]	Area [mAU*s]	Height [mAU]	Area %
1	4.367	BB	0.1857	1430.95459	117.02262	4.9063
2	7.409	VB R	0.2826	2.77345e4	1481.68115	95.0937

Totals : 2.91654e4 1598.70377

===

*** End of Report ***

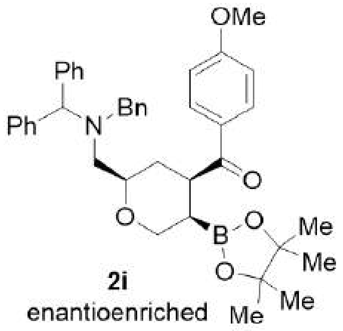

2i
enantioenriched

===

Acq. Operator : SYSTEM Seq. Line : 14
Sample Operator : SYSTEM
Acq. Instrument : HPLC Location : P1-E-01
Injection Date : 11/5/2024 12:10:41 pm Inj : 1
Inj Volume : 2.000 µl
Different Inj Volume from Sample Entry! Actual Inj Volume : 10.000 µl
Acq. Method : C:\Users\Public\Documents\ChemStation\1\Data\SUN\SUN 2024-05-11 09-32-52\AD3-10-20.M
Last changed : 15/8/2022 10:21:32 pm by SYSTEM
Analysis Method : C:\Users\Public\Documents\ChemStation\1\Data\SUN\SUN 2024-05-11 09-32-52\AD3-10-20.M (Sequence Method)
Last changed : 29/6/2024 3:03:34 pm by SYSTEM
(modified after loading)
Additional Info : Peak(s) manually integrated

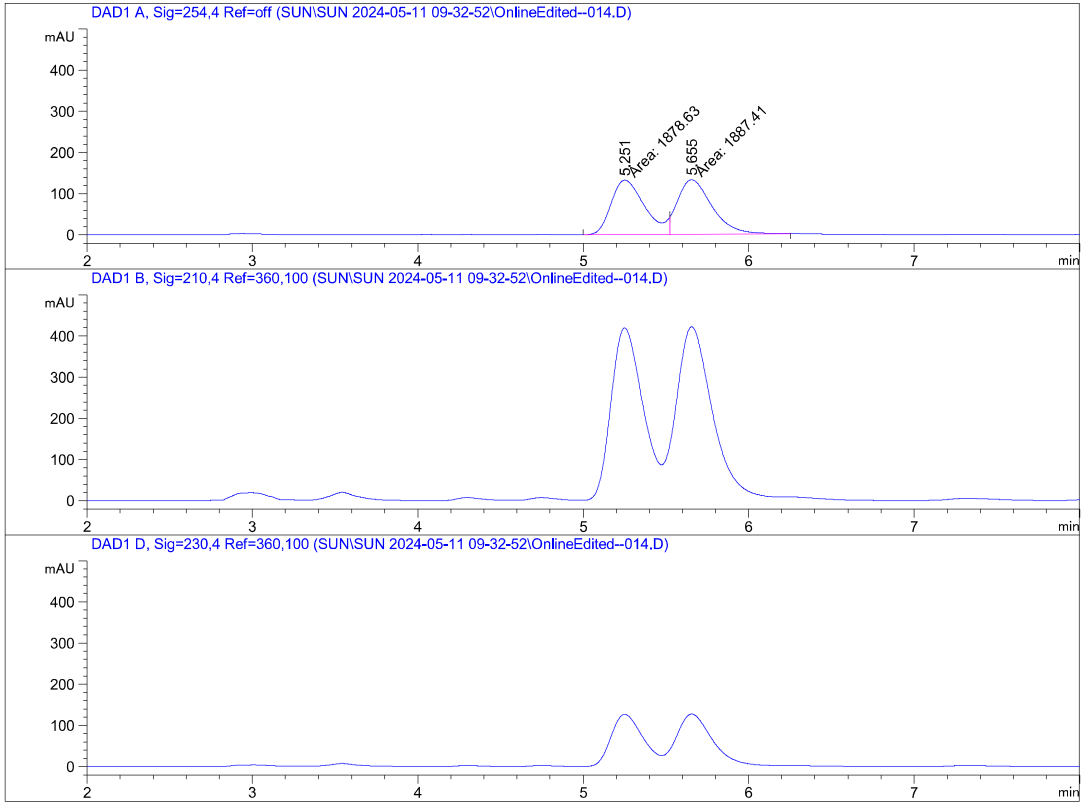

Data File C:\Users\P...\ChemStation\1\Data\SUN\SUN 2024-05-11 09-32-52\OnlineEdited--014.D
Sample Name: zmh-5-177a-rac

===

Area Percent Report

===

Sorted By : Signal
Multiplier : 1.0000
Dilution : 1.0000
Use Multiplier & Dilution Factor with ISTDs

Signal 1: DAD1 A, Sig=254,4 Ref=off

Peak #	RetTime [min]	Type	Width [min]	Area [mAU*s]	Height [mAU]	Area %
1	5.251	MF	0.2373	1878.62708	131.92404	49.8833
2	5.655	FM	0.2382	1887.41455	132.05179	50.1167

Totals : 3766.04163 263.97583

Signal 2: DAD1 B, Sig=210,4 Ref=360,100

Signal 3: DAD1 D, Sig=230,4 Ref=360,100

===

*** End of Report ***

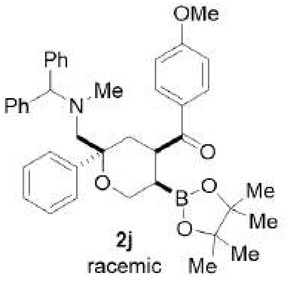

2j
racemic

===

Acq. Operator : SYSTEM Seq. Line : 2
Sample Operator : SYSTEM
Acq. Instrument : HPLC Location : P1-A-01
Injection Date : 14/5/2024 8:32:00 pm Inj : 1
Inj Volume : 2.000 µl
Different Inj Volume from Sample Entry! Actual Inj Volume : 10.000 µl
Acq. Method : C:\Users\Public\Documents\ChemStation\1\Data\SUN\SUN 2024-05-14 20-18-51\AD3-10-20.M
Last changed : 15/8/2022 10:21:32 pm by SYSTEM
Analysis Method : C:\Users\Public\Documents\ChemStation\1\Data\SUN\SUN 2024-05-14 20-18-51\AD3-10-20.M (Sequence Method)
Last changed : 29/6/2024 3:08:07 pm by SYSTEM
(modified after loading)
Additional Info : Peak(s) manually integrated

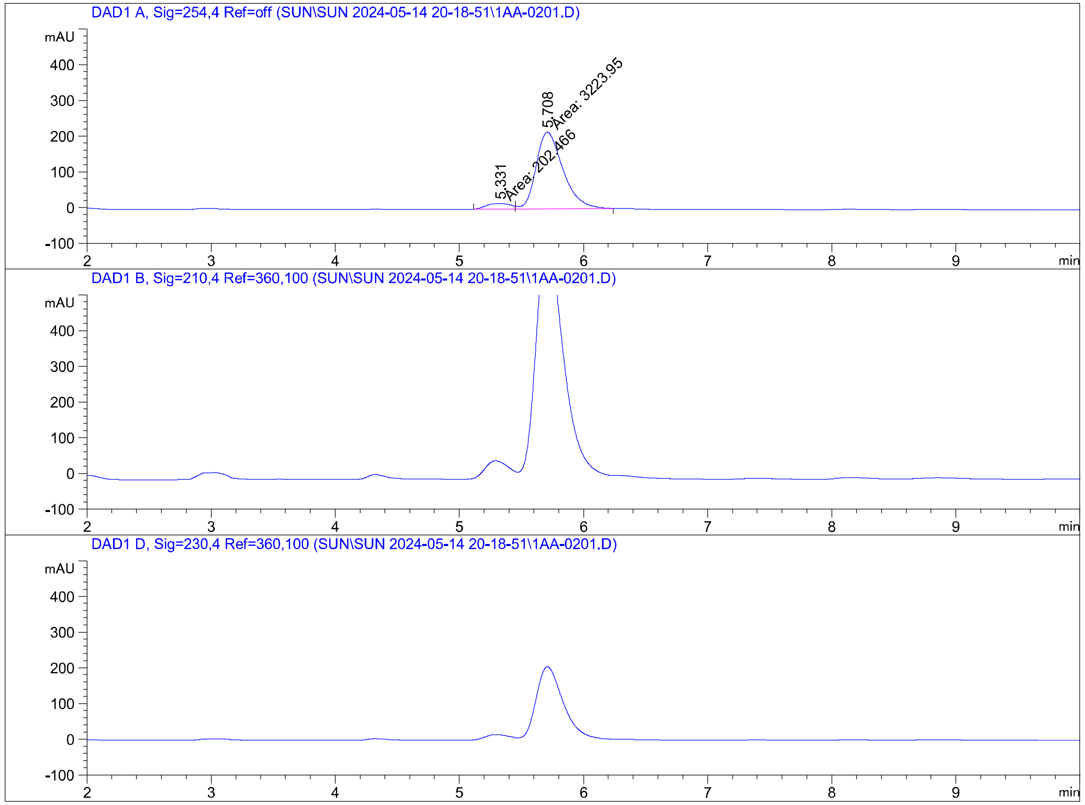

Data File C:\Users\P...Documents\ChemStation\1\Data\SUN\SUN 2024-05-14 20-18-51\1AA-0201.D
Sample Name: zmh-5-180d

===

Area Percent Report

===

Sorted By : Signal
Multiplier : 1.0000
Dilution : 1.0000
Use Multiplier & Dilution Factor with ISTDs

Signal 1: DAD1 A, Sig=254,4 Ref=off

Peak #	RetTime [min]	Type	Width [min]	Area [mAU*s]	Height [mAU]	Area %
1	5.331	MF	0.2274	202.46555	14.83965	5.9090
2	5.708	FM	0.2508	3223.94922	214.20454	94.0910

Totals : 3426.41476 229.04420

Signal 2: DAD1 B, Sig=210,4 Ref=360,100

Signal 3: DAD1 D, Sig=230,4 Ref=360,100

===

*** End of Report ***

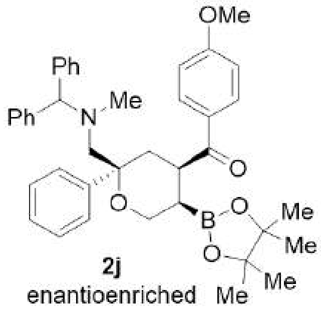

2j
enantioenriched

===

```
Acq. Operator   : SYSTEM                        Seq. Line :   2
Sample Operator : SYSTEM
Acq. Instrument : HPLC                           Location :   P1-F-01
Injection Date  : 29/4/2024 12:32:31 pm               Inj :   1
                                               Inj Volume : 2.000 µl
Different Inj Volume from Sample Entry!  Actual Inj Volume : 10.000 µl
Acq. Method     : C:\Users\Public\Documents\ChemStation\1\Data\SUN\SUN 2024-04-29 12-19-26
                  \AD3-10-20.M
Last changed    : 15/8/2022 10:21:32 pm by SYSTEM
Analysis Method : C:\Users\Public\Documents\ChemStation\1\Data\SUN\SUN 2024-04-29 12-19-26
                  \AD3-10-20.M (Sequence Method)
Last changed    : 29/6/2024 3:37:35 pm by SYSTEM
                  (modified after loading)
Additional Info : Peak(s) manually integrated
```

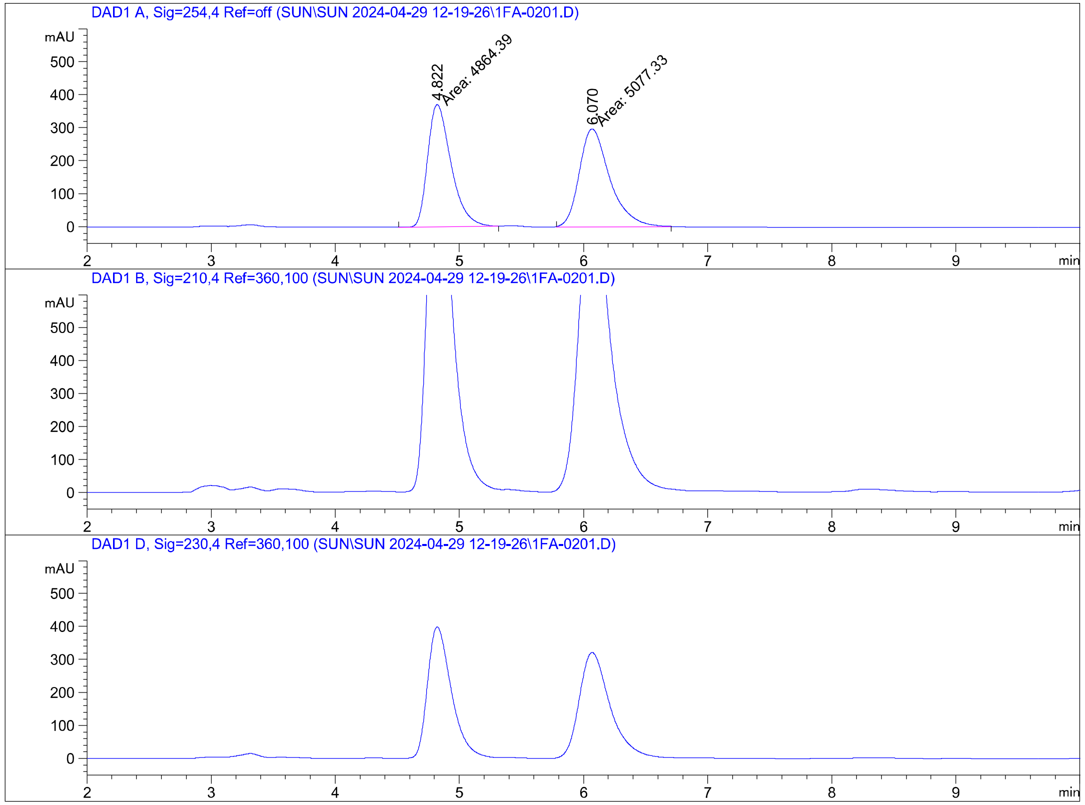

Data File C:\Users\P...Documents\ChemStation\1\Data\SUN\SUN 2024-04-29 12-19-26\1FA-0201.D
Sample Name: zmh-5-163a-rac

===

Area Percent Report

===

```
Sorted By             :      Signal
Multiplier            :      1.0000
Dilution              :      1.0000
Use Multiplier & Dilution Factor with ISTDs
```

Signal 1: DAD1 A, Sig=254,4 Ref=off

Peak #	RetTime [min]	Type	Width [min]	Area [mAU*s]	Height [mAU]	Area %
1	4.822	MM	0.2186	4864.38818	370.94012	48.9291
2	6.070	MM	0.2849	5077.32520	297.03305	51.0709
Totals :				9941.71338	667.97318	

Signal 2: DAD1 B, Sig=210,4 Ref=360,100

Signal 3: DAD1 D, Sig=230,4 Ref=360,100

===

*** End of Report ***

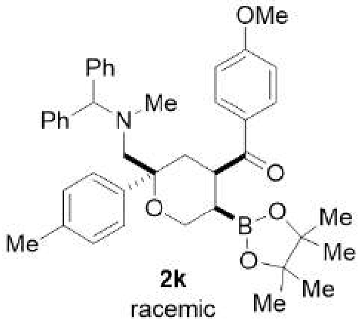

2k
racemic

===

Acq. Operator : SYSTEM Seq. Line : 3
Sample Operator : SYSTEM
Acq. Instrument : HPLC Location : P1-F-02
Injection Date : 15/5/2024 6:57:06 pm Inj : 1
Inj Volume : 2.000 µl
Different Inj Volume from Sample Entry! Actual Inj Volume : 10.000 µl
Acq. Method : C:\Users\Public\Documents\ChemStation\1\Data\SUN\SUN 2024-05-15 18-23-09\AD3-10-20.M
Last changed : 15/8/2022 10:21:32 pm by SYSTEM
Analysis Method : C:\Users\Public\Documents\ChemStation\1\Data\SUN\SUN 2024-05-15 18-23-09\AD3-10-20.M (Sequence Method)
Last changed : 29/6/2024 3:40:09 pm by SYSTEM
(modified after loading)
Additional Info : Peak(s) manually integrated

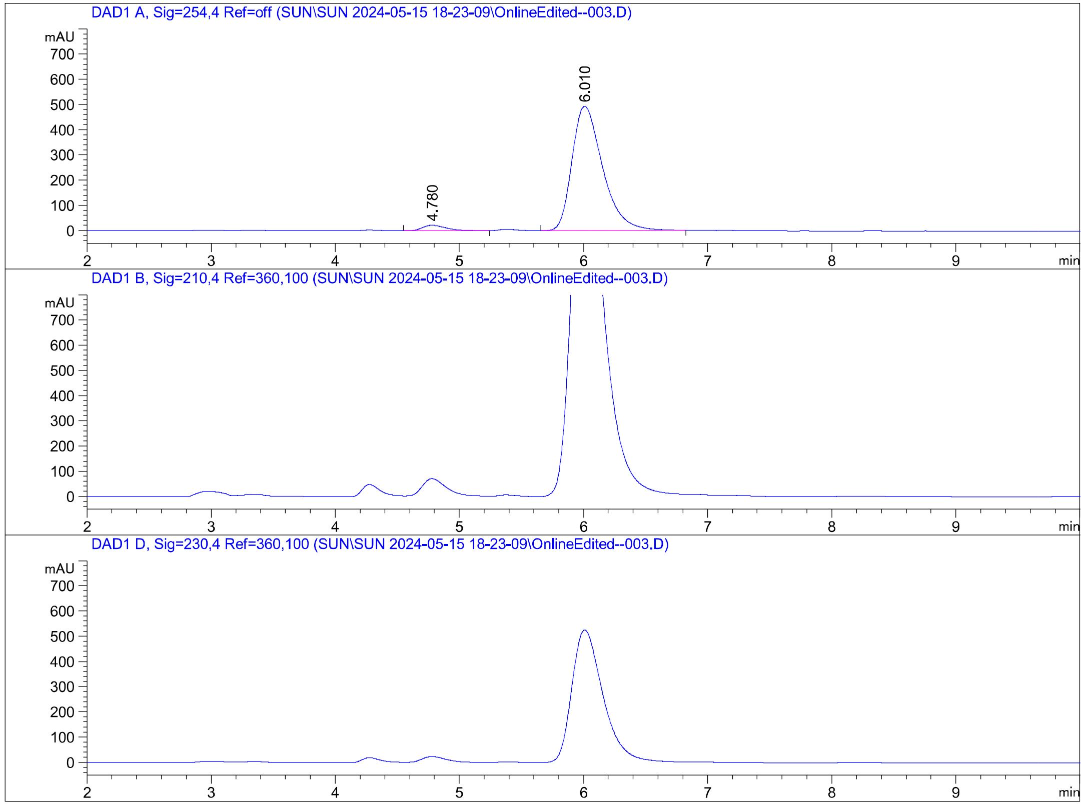

Data File C:\Users\P...\ChemStation\1\Data\SUN\SUN 2024-05-15 18-23-09\OnlineEdited--003.D
Sample Name: zmh-5-180h

===

Area Percent Report

===

Sorted By : Signal
Multiplier : 1.0000
Dilution : 1.0000
Use Multiplier & Dilution Factor with ISTDs

Signal 1: DAD1 A, Sig=254,4 Ref=off

Peak #	RetTime [min]	Type	Width [min]	Area [mAU*s]	Height [mAU]	Area %
1	4.780	BV	0.2057	295.27682	21.64379	3.3707
2	6.010	BB	0.2627	8464.94629	493.18549	96.6293

Totals : 8760.22311 514.82927

Signal 2: DAD1 B, Sig=210,4 Ref=360,100

Signal 3: DAD1 D, Sig=230,4 Ref=360,100

===

*** End of Report ***

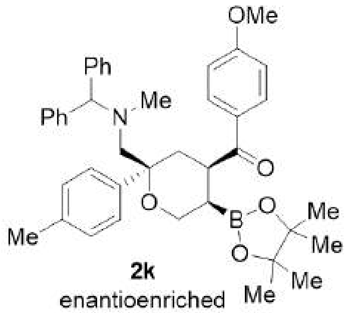

2k
enantioenriched

===

Acq. Operator : SYSTEM Seq. Line : 3
Sample Operator : SYSTEM
Acq. Instrument : HPLC Location : P1-F-02
Injection Date : 29/4/2024 12:53:34 pm Inj : 1
Inj Volume : 2.000 µl
Different Inj Volume from Sample Entry! Actual Inj Volume : 10.000 µl
Acq. Method : C:\Users\Public\Documents\ChemStation\1\Data\SUN\SUN 2024-04-29 12-19-26 \AD3-10-20.M
Last changed : 15/8/2022 10:21:32 pm by SYSTEM
Analysis Method : C:\Users\Public\Documents\ChemStation\1\Data\SUN\SUN 2024-04-29 12-19-26 \AD3-10-20.M (Sequence Method)
Last changed : 29/6/2024 3:11:04 pm by SYSTEM
(modified after loading)
Additional Info : Peak(s) manually integrated

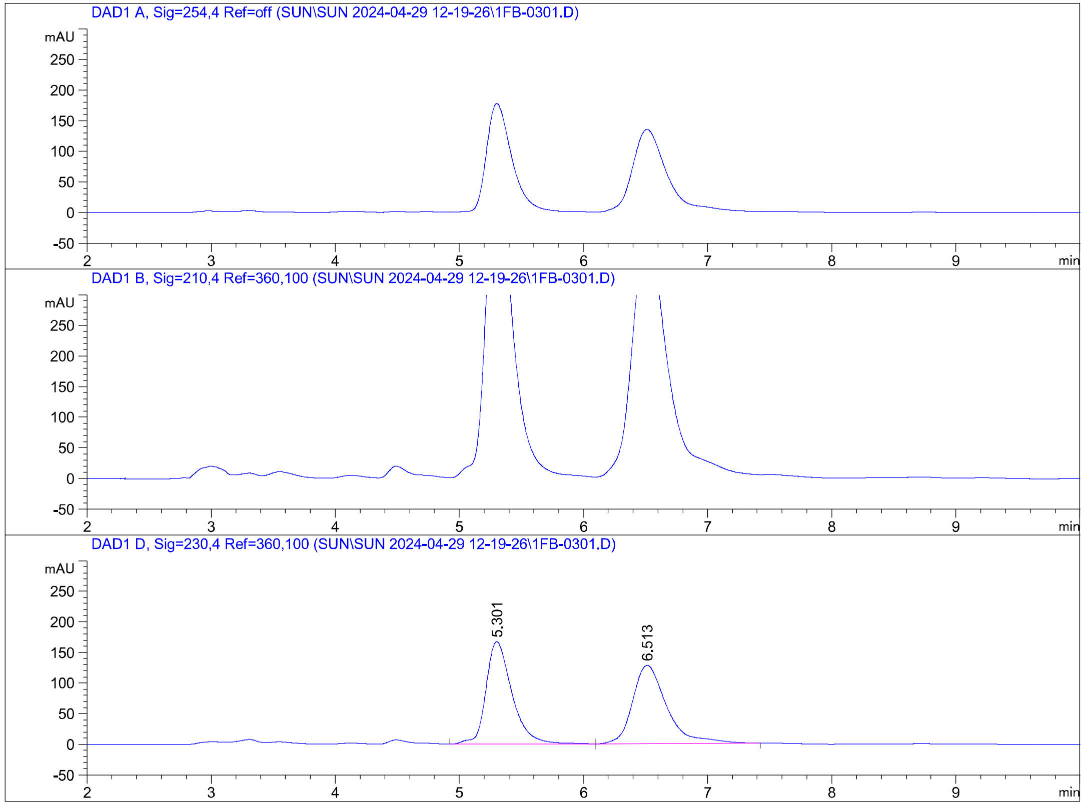

Data File C:\Users\P...Documents\ChemStation\1\Data\SUN\SUN 2024-04-29 12-19-26\1FB-0301.D
Sample Name: zmh-5-163b-rac

===

Area Percent Report

===

Sorted By : Signal
Multiplier : 1.0000
Dilution : 1.0000
Use Multiplier & Dilution Factor with ISTDs

Signal 1: DAD1 A, Sig=254,4 Ref=off

Signal 2: DAD1 B, Sig=210,4 Ref=360,100

Signal 3: DAD1 D, Sig=230,4 Ref=360,100

Peak #	RetTime [min]	Type	Width [min]	Area [mAU*s]	Height [mAU]	Area %
1	5.301	BB	0.2197	2431.29272	167.10831	49.9718
2	6.513	BB	0.2891	2434.03906	127.88009	50.0282

Totals : 4865.33179 294.98840

===

*** End of Report ***

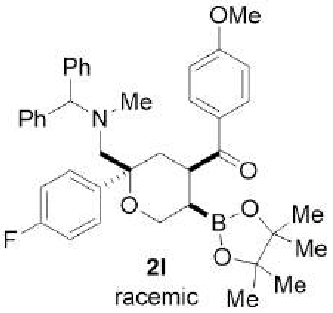

2l
racemic

===

```
Acq. Operator   : SYSTEM                        Seq. Line :  12
Sample Operator : SYSTEM
Acq. Instrument : HPLC                           Location :   P1-F-02
Injection Date  : 15/5/2024 12:30:46 am                Inj :   1
                                               Inj Volume : 2.000 µl
Different Inj Volume from Sample Entry!  Actual Inj Volume : 10.000 µl
Acq. Method     : C:\Users\Public\Documents\ChemStation\1\Data\SUN\SUN 2024-05-14 20-56-06
                  \AD3-10-20.M
Last changed    : 15/8/2022 10:21:32 pm by SYSTEM
Analysis Method : C:\Users\Public\Documents\ChemStation\1\Data\SUN\SUN 2024-05-14 20-56-06
                  \AD3-10-20.M (Sequence Method)
Last changed    : 29/6/2024 3:15:12 pm by SYSTEM
                  (modified after loading)
Additional Info : Peak(s) manually integrated
```

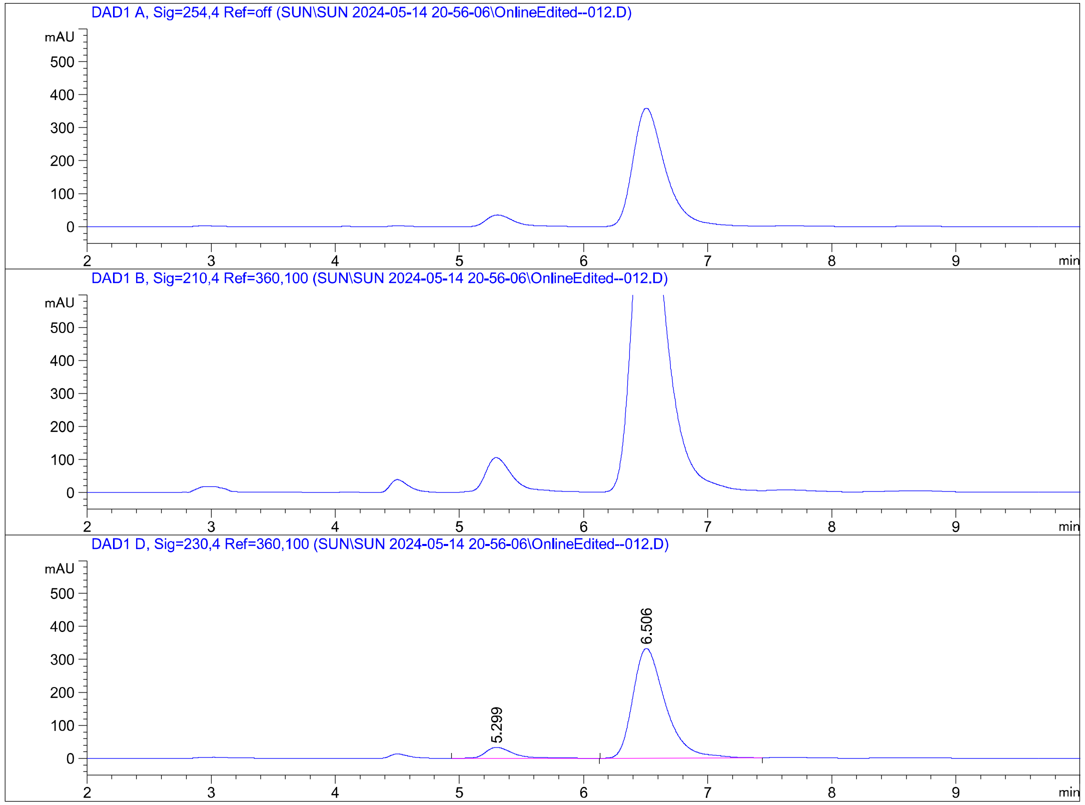

===

Area Percent Report

===

```
Sorted By             :      Signal
Multiplier            :      1.0000
Dilution              :      1.0000
Use Multiplier & Dilution Factor with ISTDs
```

Signal 1: DAD1 A, Sig=254,4 Ref=off

Signal 2: DAD1 B, Sig=210,4 Ref=360,100

Signal 3: DAD1 D, Sig=230,4 Ref=360,100

Peak #	RetTime [min]	Type	Width [min]	Area [mAU*s]	Height [mAU]	Area %
1	5.299	BB	0.2349	519.22345	33.11973	7.9027
2	6.506	BB	0.2768	6050.95996	332.45712	92.0973

Totals : 6570.18341 365.57685

===

*** End of Report ***

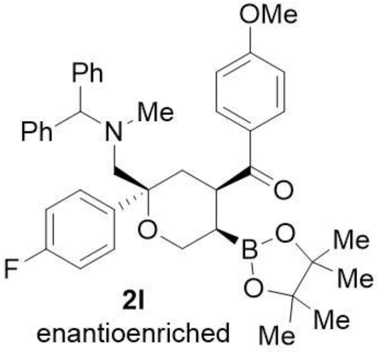

2l
enantioenriched

===

Acq. Operator : SYSTEM Seq. Line : 39
Sample Operator : SYSTEM
Acq. Instrument : HPLC Location : P1-E-02
Injection Date : 11/5/2024 7:53:55 pm Inj : 1
Inj Volume : 2.000 µl
Different Inj Volume from Sample Entry! Actual Inj Volume : 10.000 µl
Acq. Method : C:\Users\Public\Documents\ChemStation\1\Data\SUN\SUN 2024-05-11 09-32-52 \IE3-10-20.M
Last changed : 15/8/2022 10:27:27 pm by SYSTEM
Analysis Method : C:\Users\Public\Documents\ChemStation\1\Data\SUN\SUN 2024-05-11 09-32-52 \IE3-10-20.M (Sequence Method)
Last changed : 29/6/2024 3:20:06 pm by SYSTEM
(modified after loading)
Additional Info : Peak(s) manually integrated

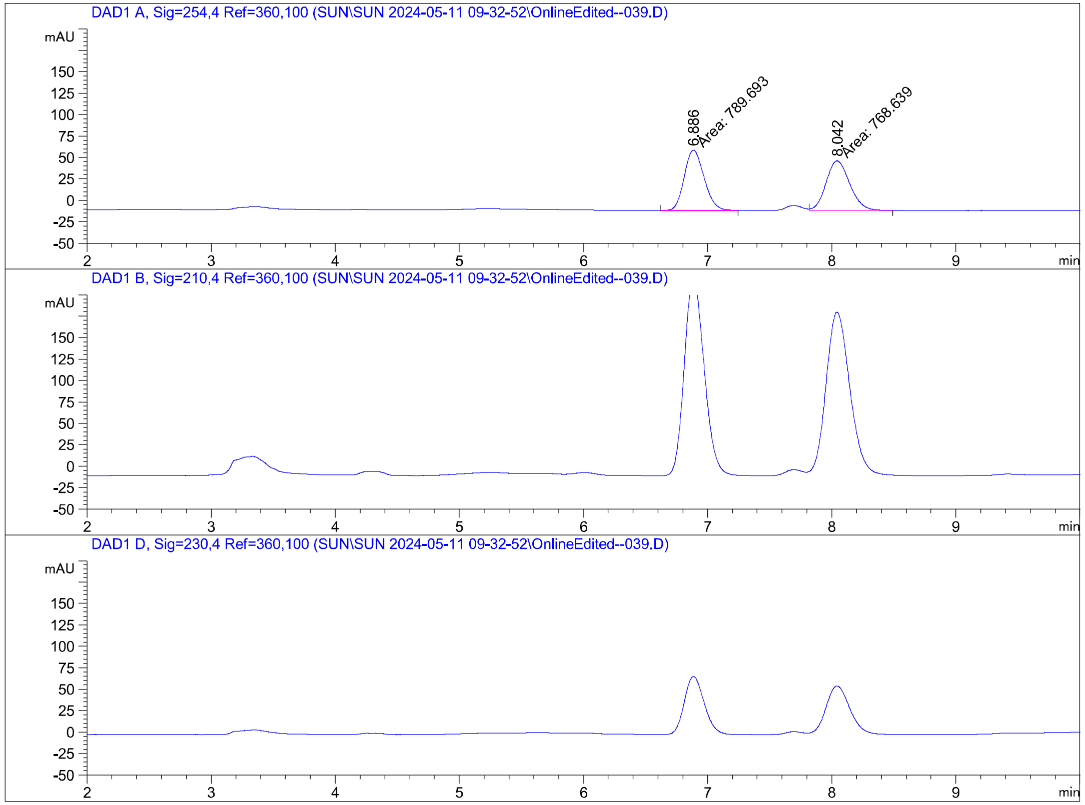

Data File C:\Users\P...\ChemStation\1\Data\SUN\SUN 2024-05-11 09-32-52\OnlineEdited--039.D
Sample Name: zmh-5-177b-rac-ie

===

Area Percent Report

===

Sorted By : Signal
Multiplier : 1.0000
Dilution : 1.0000
Use Multiplier & Dilution Factor with ISTDs

Signal 1: DAD1 A, Sig=254,4 Ref=360,100

Peak #	RetTime [min]	Type	Width [min]	Area [mAU*s]	Height [mAU]	Area %
1	6.886	MM	0.1865	789.69281	70.56861	50.6755
2	8.042	FM	0.2210	768.63879	57.96238	49.3245

Totals : 1558.33160 128.53099

Signal 2: DAD1 B, Sig=210,4 Ref=360,100

Signal 3: DAD1 D, Sig=230,4 Ref=360,100

===
*** End of Report ***

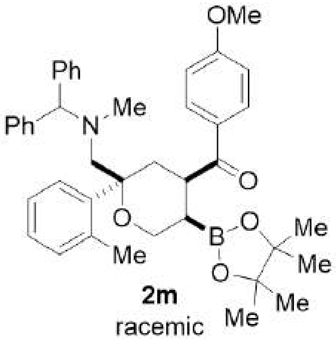

2m
racemic

===
Acq. Operator : SYSTEM   Seq. Line : 14
Sample Operator : SYSTEM
Acq. Instrument : HPLC   Location : P1-F-01
Injection Date : 15/5/2024 1:03:02 am   Inj : 1
Inj Volume : 2.000 µl
Different Inj Volume from Sample Entry! Actual Inj Volume : 10.000 µl
Acq. Method : C:\Users\Public\Documents\ChemStation\1\Data\SUN\SUN 2024-05-14 20-56-06\IE3-10-20.M
Last changed : 15/8/2022 10:27:27 pm by SYSTEM
Analysis Method : C:\Users\Public\Documents\ChemStation\1\Data\SUN\SUN 2024-05-14 20-56-06\IE3-10-20.M (Sequence Method)
Last changed : 29/6/2024 3:23:50 pm by SYSTEM
(modified after loading)
Additional Info : Peak(s) manually integrated

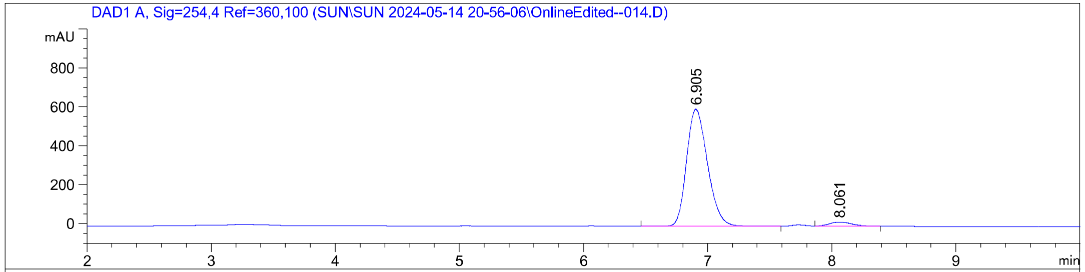

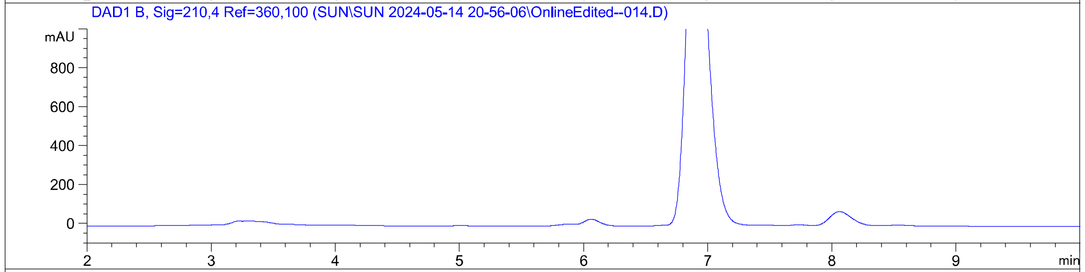

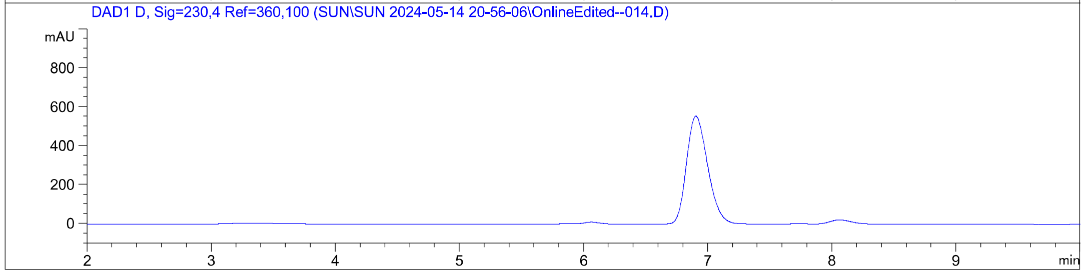

Data File C:\Users\P...\ChemStation\1\Data\SUN\SUN 2024-05-14 20-56-06\OnlineEdited--014.D
Sample Name: zmh-5-180f

===
Area Percent Report
===

Sorted By : Signal
Multiplier : 1.0000
Dilution : 1.0000
Use Multiplier & Dilution Factor with ISTDs

Signal 1: DAD1 A, Sig=254,4 Ref=360,100

Peak #	RetTime [min]	Type	Width [min]	Area [mAU*s]	Height [mAU]	Area %
1	6.905	BV	0.1821	7060.33594	600.99030	96.1832
2	8.061	VB	0.1935	280.17535	22.24009	3.8168
Totals :				7340.51129	623.23038	

Signal 2: DAD1 B, Sig=210,4 Ref=360,100

Signal 3: DAD1 D, Sig=230,4 Ref=360,100

===
*** End of Report ***

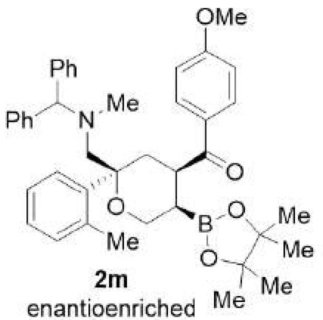

2m
enantioenriched

Data File C:\Users\P...\ChemStation\1\Data\SUN\SUN 2024-04-17 10-37-28\OnlineEdited--015.D
Sample Name: zmh-5-144-rac-ad

===

Acq. Operator : SYSTEM Seq. Line : 15
Sample Operator : SYSTEM
Acq. Instrument : HPLC Location : P1-F-01
Injection Date : 17/4/2024 3:14:47 pm Inj : 1
Inj Volume : 2.000 µl
Different Inj Volume from Sample Entry! Actual Inj Volume : 10.000 µl
Acq. Method : C:\Users\Public\Documents\ChemStation\1\Data\SUN\SUN 2024-04-17 10-37-28\AD3-10-20.M
Last changed : 15/8/2022 10:21:32 pm by SYSTEM
Analysis Method : C:\Users\Public\Documents\ChemStation\1\Data\SUN\SUN 2024-04-17 10-37-28\AD3-10-20.M (Sequence Method)
Last changed : 29/6/2024 3:29:28 pm by SYSTEM
(modified after loading)
Additional Info : Peak(s) manually integrated

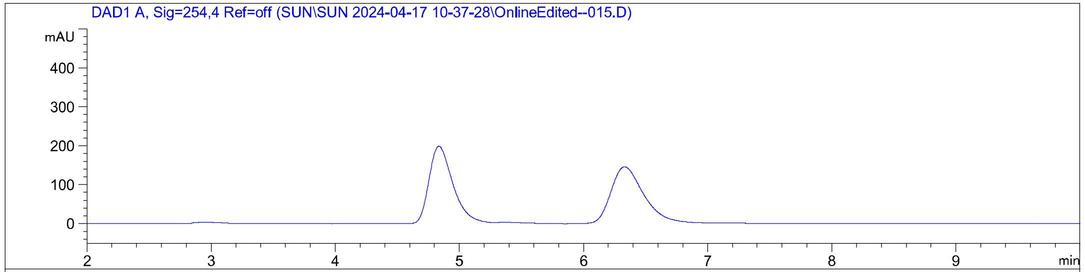

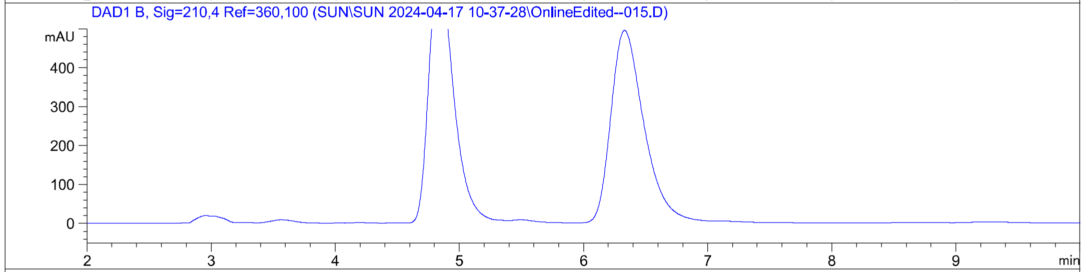

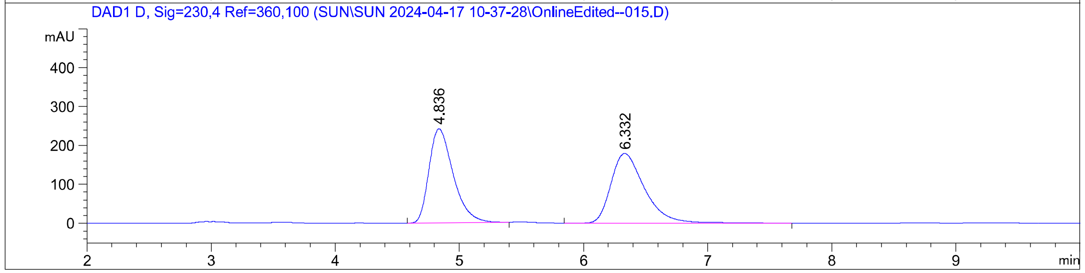

Data File C:\Users\P...\ChemStation\1\Data\SUN\SUN 2024-04-17 10-37-28\OnlineEdited--015.D
Sample Name: zmh-5-144-rac-ad

===

Area Percent Report

===

Sorted By : Signal
Multiplier : 1.0000
Dilution : 1.0000
Use Multiplier & Dilution Factor with ISTDs

Signal 1: DAD1 A, Sig=254,4 Ref=off

Signal 2: DAD1 B, Sig=210,4 Ref=360,100

Signal 3: DAD1 D, Sig=230,4 Ref=360,100

Peak #	RetTime [min]	Type	Width [min]	Area [mAU*s]	Height [mAU]	Area %
1	4.836	BB	0.2074	3267.57495	241.41077	49.1375
2	6.332	BB	0.2861	3382.28662	178.90895	50.8625
Totals :				6649.86157	420.31972	

===

*** End of Report ***

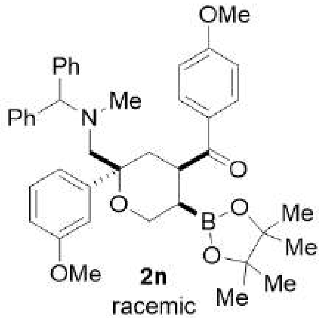

2n
racemic

===

Acq. Operator : SYSTEM Seq. Line : 2
Sample Operator : SYSTEM
Acq. Instrument : HPLC Location : P1-F-01
Injection Date : 15/5/2024 6:36:02 pm Inj : 1
Inj Volume : 2.000 µl
Different Inj Volume from Sample Entry! Actual Inj Volume : 10.000 µl
Acq. Method : C:\Users\Public\Documents\ChemStation\1\Data\SUN\SUN 2024-05-15 18-23-09\AD3-10-20.M
Last changed : 15/8/2022 10:21:32 pm by SYSTEM
Analysis Method : C:\Users\Public\Documents\ChemStation\1\Data\SUN\SUN 2024-05-15 18-23-09\AD3-10-20.M (Sequence Method)
Last changed : 29/6/2024 3:33:48 pm by SYSTEM
(modified after loading)
Additional Info : Peak(s) manually integrated

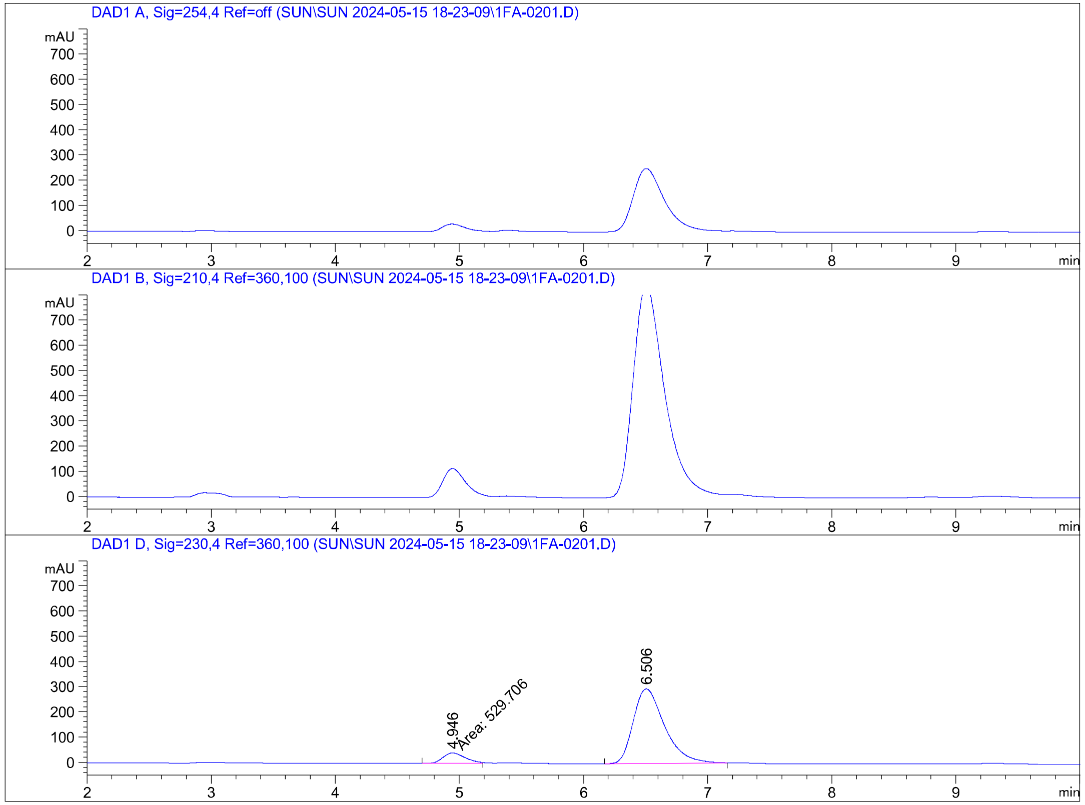

Data File C:\Users\P...Documents\ChemStation\1\Data\SUN\SUN 2024-05-15 18-23-09\1FA-0201.D
Sample Name: zmh-5-180g

===

Area Percent Report

===

Sorted By : Signal
Multiplier : 1.0000
Dilution : 1.0000
Use Multiplier & Dilution Factor with ISTDs

Signal 1: DAD1 A, Sig=254,4 Ref=off

Signal 2: DAD1 B, Sig=210,4 Ref=360,100

Signal 3: DAD1 D, Sig=230,4 Ref=360,100

Peak #	RetTime [min]	Type	Width [min]	Area [mAU*s]	Height [mAU]	Area %
1	4.946	MF	0.2103	529.70605	41.97714	9.3382
2	6.506	BB	0.2651	5142.74756	295.36874	90.6618

Totals : 5672.45361 337.34588

===

*** End of Report ***

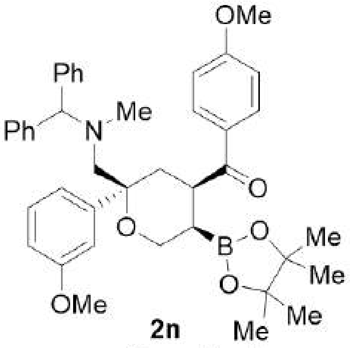

2n
enantioenriched

===

Acq. Operator : SYSTEM Seq. Line : 16
Sample Operator : SYSTEM
Acq. Instrument : HPLC Location : P1-E-03
Injection Date : 11/5/2024 12:53:02 pm Inj : 1
Inj Volume : 2.000 µl
Different Inj Volume from Sample Entry! Actual Inj Volume : 10.000 µl
Acq. Method : C:\Users\Public\Documents\ChemStation\1\Data\SUN\SUN 2024-05-11 09-32-52 \AD3-10-20.M
Last changed : 15/8/2022 10:21:32 pm by SYSTEM
Analysis Method : C:\Users\Public\Documents\ChemStation\1\Data\SUN\SUN 2024-05-11 09-32-52 \AD3-10-20.M (Sequence Method)
Last changed : 29/6/2024 3:43:43 pm by SYSTEM
(modified after loading)
Additional Info : Peak(s) manually integrated

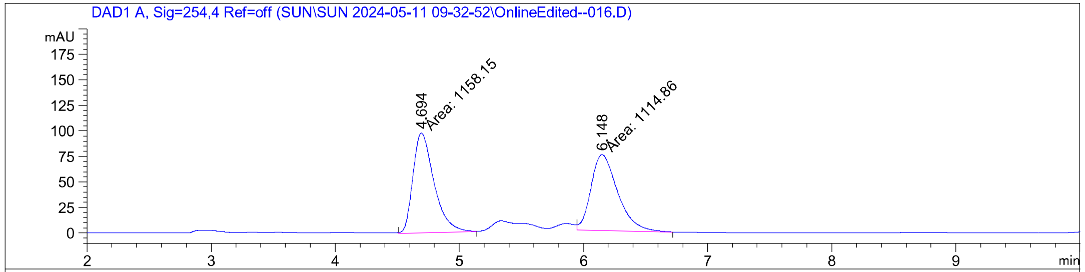

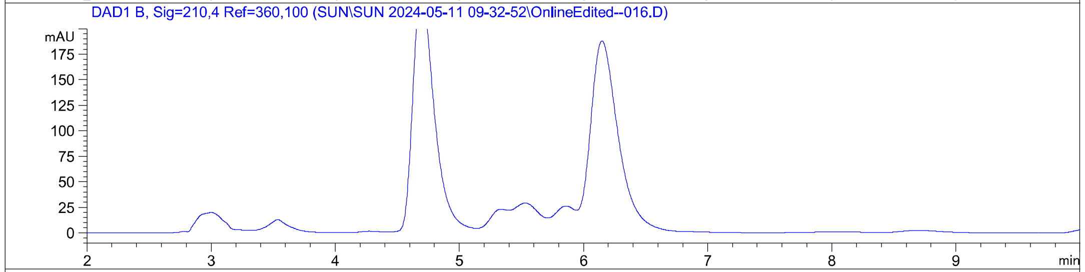

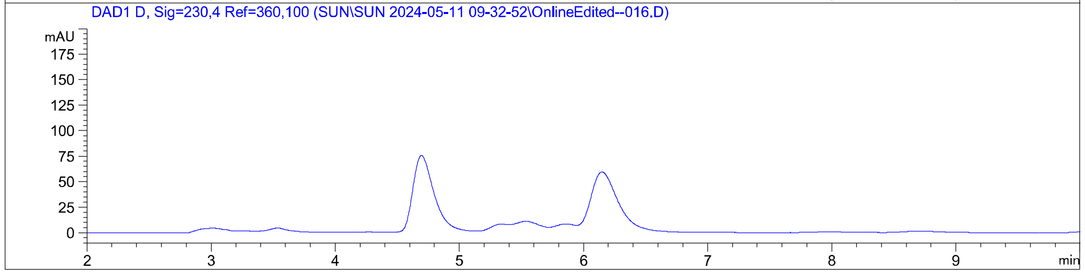

Data File C:\Users\P...\ChemStation\1\Data\SUN\SUN 2024-05-11 09-32-52\OnlineEdited--016.D
Sample Name: zmh-5-177a-rac

===

Area Percent Report

===

Sorted By : Signal
Multiplier : 1.0000
Dilution : 1.0000
Use Multiplier & Dilution Factor with ISTDs

Signal 1: DAD1 A, Sig=254,4 Ref=off

Peak #	RetTime [min]	Type	Width [min]	Area [mAU*s]	Height [mAU]	Area %
1	4.694	MM	0.1974	1158.14661	97.77737	50.9521
2	6.148	FM	0.2507	1114.86304	74.12889	49.0479
Totals :				2273.00964	171.90627	

Signal 2: DAD1 B, Sig=210,4 Ref=360,100

Signal 3: DAD1 D, Sig=230,4 Ref=360,100

===

*** End of Report ***

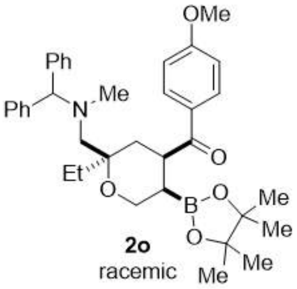

2o
racemic

===

```
Acq. Operator   : SYSTEM                         Seq. Line :   6
Sample Operator : SYSTEM
Acq. Instrument : HPLC                            Location :   P1-F-01
Injection Date  : 15/5/2024 8:39:57 pm                 Inj :   1
                                                Inj Volume : 2.000 µl
Different Inj Volume from Sample Entry!  Actual Inj Volume : 10.000 µl
Acq. Method     : C:\Users\Public\Documents\ChemStation\1\Data\SUN\SUN 2024-05-15 19-37-48
                  \AD3-10-20.M
Last changed    : 15/8/2022 10:21:32 pm by SYSTEM
Analysis Method : C:\Users\Public\Documents\ChemStation\1\Data\SUN\SUN 2024-05-15 19-37-48
                  \AD3-10-20.M (Sequence Method)
Last changed    : 29/6/2024 3:46:36 pm by SYSTEM
                  (modified after loading)
Additional Info : Peak(s) manually integrated
```

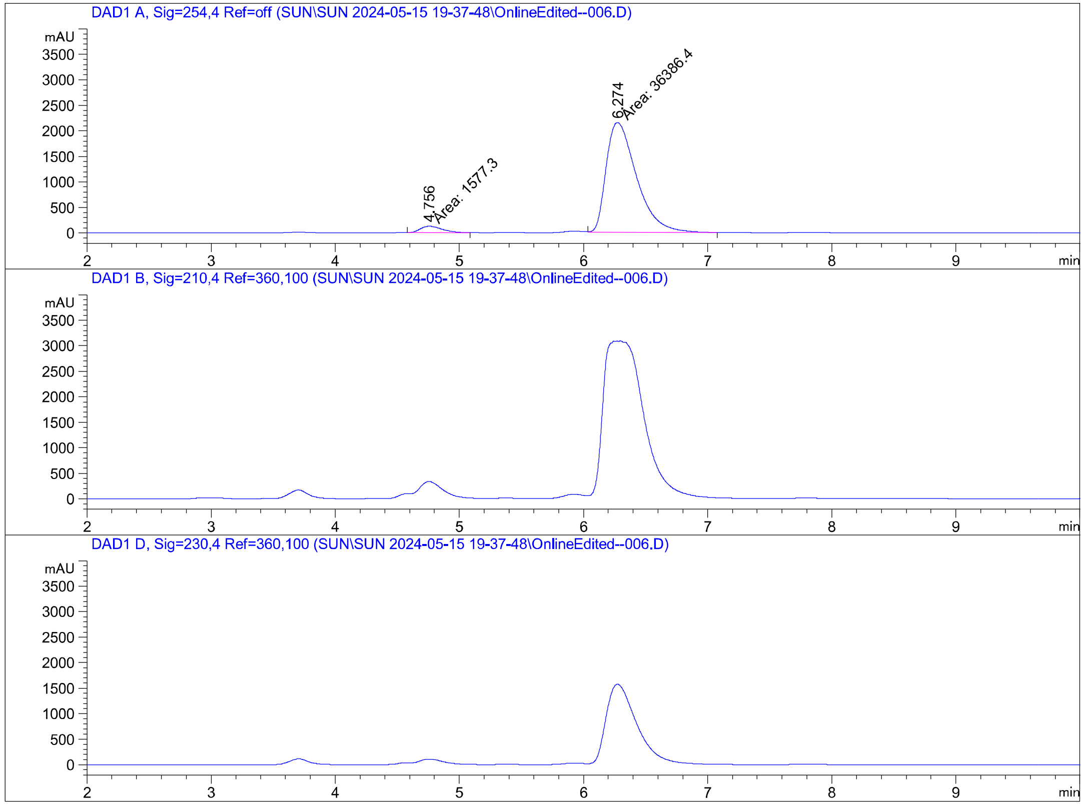

Data File C:\Users\P...\ChemStation\1\Data\SUN\SUN 2024-05-15 19-37-48\OnlineEdited--006.D
Sample Name: zmh-5-180i

===

Area Percent Report

===

```
Sorted By             :      Signal
Multiplier            :      1.0000
Dilution              :      1.0000
Use Multiplier & Dilution Factor with ISTDs
```

Signal 1: DAD1 A, Sig=254,4 Ref=off

Peak #	RetTime [min]	Type	Width [min]	Area [mAU*s]	Height [mAU]	Area %
1	4.756	MM	0.2086	1577.30164	126.01213	4.1548
2	6.274	MM	0.2825	3.63864e4	2146.96875	95.8452

Totals : 3.79637e4 2272.98088

Signal 2: DAD1 B, Sig=210,4 Ref=360,100

Signal 3: DAD1 D, Sig=230,4 Ref=360,100

===

*** End of Report ***

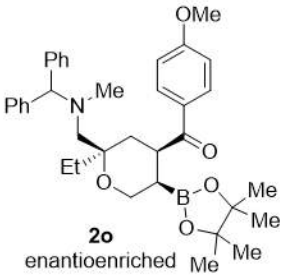

2o
enantioenriched

===

```
Acq. Operator   : SYSTEM                      Seq. Line :  17
Sample Operator : SYSTEM
Acq. Instrument : HPLC                         Location :   P1-E-04
Injection Date  : 11/5/2024 1:14:07 pm              Inj :   1
                                             Inj Volume : 2.000 µl
Different Inj Volume from Sample Entry!  Actual Inj Volume : 10.000 µl
Acq. Method     : C:\Users\Public\Documents\ChemStation\1\Data\SUN\SUN 2024-05-11 09-32-52
                  \AD3-10-20.M
Last changed    : 15/8/2022 10:21:32 pm by SYSTEM
Analysis Method : C:\Users\Public\Documents\ChemStation\1\Data\SUN\SUN 2024-05-11 09-32-52
                  \AD3-10-20.M (Sequence Method)
Last changed    : 29/6/2024 4:19:28 pm by SYSTEM
                  (modified after loading)
Additional Info : Peak(s) manually integrated
```

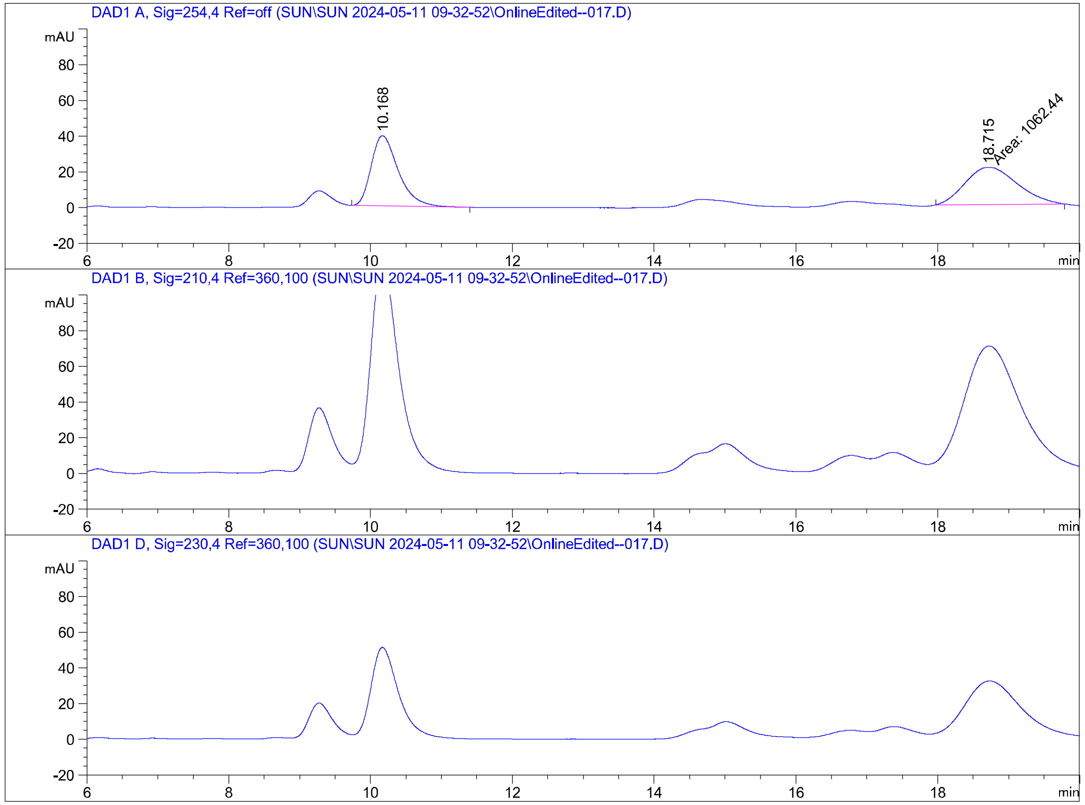

Data File C:\Users\P...\ChemStation\1\Data\SUN\SUN 2024-05-11 09-32-52\OnlineEdited--017.D
Sample Name: zmh-5-177d-rac

===

Area Percent Report

===

```
Sorted By             :      Signal
Multiplier            :      1.0000
Dilution              :      1.0000
Use Multiplier & Dilution Factor with ISTDs
```

Signal 1: DAD1 A, Sig=254,4 Ref=off

Peak #	RetTime [min]	Type	Width [min]	Area [mAU*s]	Height [mAU]	Area %
1	10.168	BB	0.3894	1047.36975	39.36895	49.6428
2	18.715	PP	0.8479	1062.44336	20.88366	50.3572

Totals : 2109.81311   60.25261

Signal 2: DAD1 B, Sig=210,4 Ref=360,100

Signal 3: DAD1 D, Sig=230,4 Ref=360,100

===

*** End of Report ***

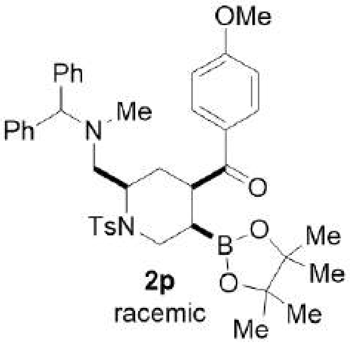

2p
racemic

===

Acq. Operator : SYSTEM Seq. Line : 2
Sample Operator : SYSTEM
Acq. Instrument : HPLC Location : P1-A-01
Injection Date : 18/5/2024 7:50:09 pm Inj : 1
Inj Volume : 2.000 µl
Different Inj Volume from Sample Entry! Actual Inj Volume : 10.000 µl
Acq. Method : C:\Users\Public\Documents\ChemStation\1\Data\SUN\SUN 2024-05-18 19-27-07\AD3-10-30.M
Last changed : 24/10/2022 9:57:21 am by SYSTEM
Analysis Method : C:\Users\Public\Documents\ChemStation\1\Data\SUN\SUN 2024-05-18 19-27-07\AD3-10-30.M (Sequence Method)
Last changed : 29/6/2024 4:22:41 pm by SYSTEM
(modified after loading)
Additional Info : Peak(s) manually integrated

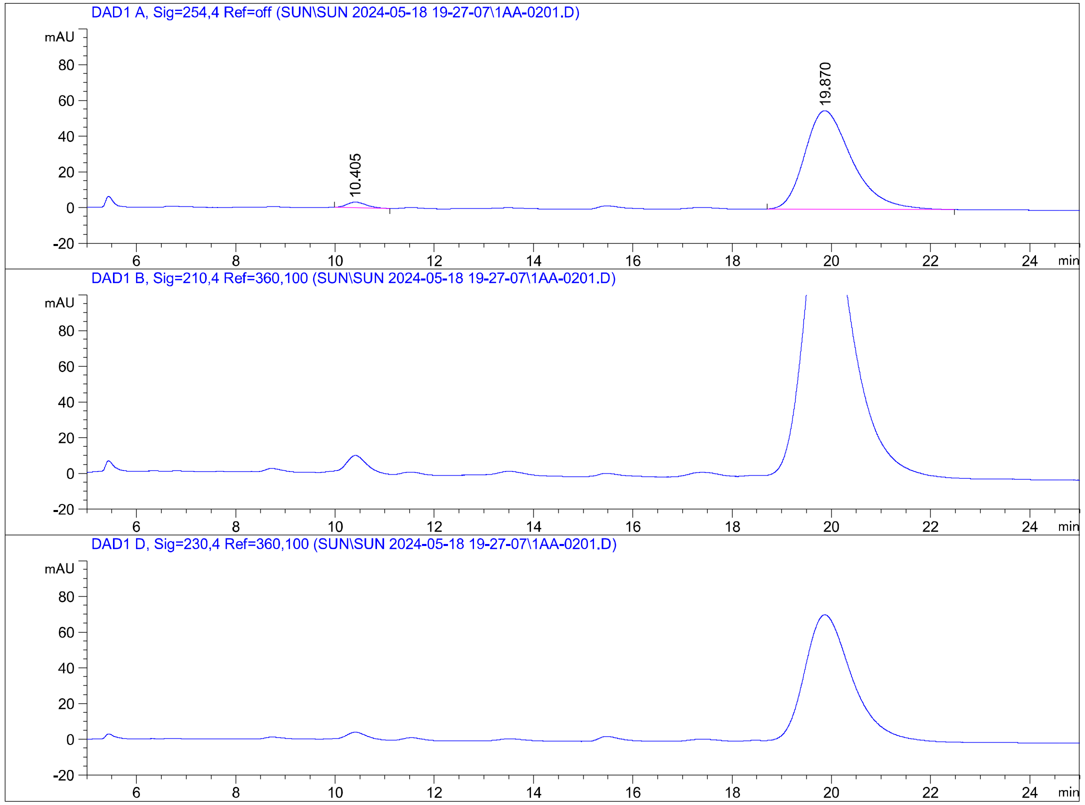

Data File C:\Users\P...Documents\ChemStation\1\Data\SUN\SUN 2024-05-18 19-27-07\1AA-0201.D
Sample Name: zmh-5-180m-re-20

===

Area Percent Report

===

Sorted By : Signal
Multiplier : 1.0000
Dilution : 1.0000
Use Multiplier & Dilution Factor with ISTDs

Signal 1: DAD1 A, Sig=254,4 Ref=off

Peak #	RetTime [min]	Type	Width [min]	Area [mAU*s]	Height [mAU]	Area %
1	10.405	BB	0.3189	84.49731	3.12573	2.2696
2	19.870	BB	0.7825	3638.55176	55.13100	97.7304

Totals : 3723.04906 58.25673

Signal 2: DAD1 B, Sig=210,4 Ref=360,100

Signal 3: DAD1 D, Sig=230,4 Ref=360,100

===

*** End of Report ***

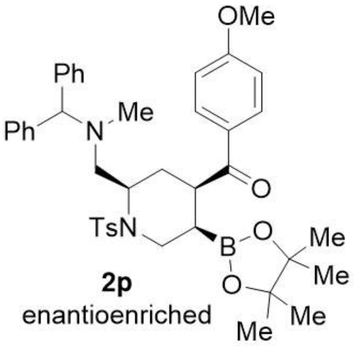

2p
enantioenriched

===

Acq. Operator : SYSTEM Seq. Line : 69
Sample Operator : SYSTEM
Acq. Instrument : HPLC Location : P1-F-06
Injection Date : 18/4/2024 4:45:50 am Inj : 1
Inj Volume : 2.000 µl
Different Inj Volume from Sample Entry! Actual Inj Volume : 10.000 µl
Acq. Method : C:\Users\Public\Documents\ChemStation\1\Data\SUN\SUN 2024-04-17 10-37-28\IBN3-10-20.M
Last changed : 30/7/2023 1:22:19 pm by SYSTEM
Analysis Method : C:\Users\Public\Documents\ChemStation\1\Data\SUN\SUN 2024-04-17 10-37-28\IBN3-10-20.M (Sequence Method)
Last changed : 10/10/2024 9:20:29 pm by SYSTEM
(modified after loading)
Additional Info : Peak(s) manually integrated

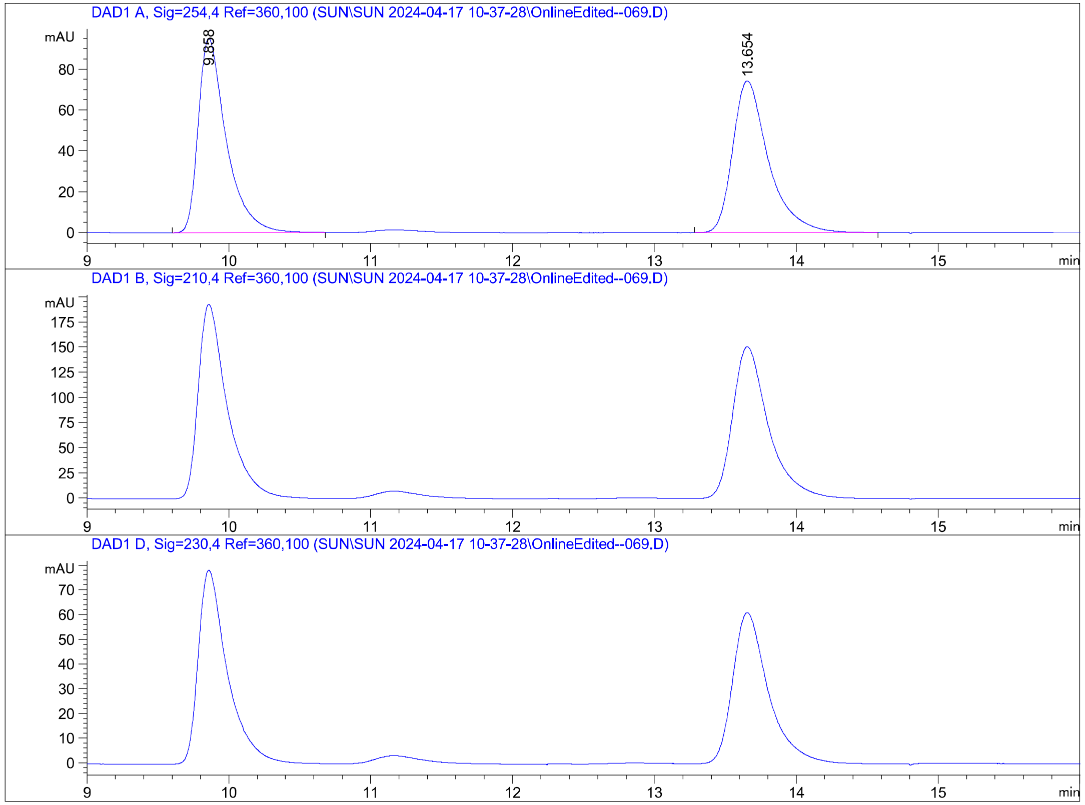

Data File C:\Users\P...\ChemStation\1\Data\SUN\SUN 2024-04-17 10-37-28\OnlineEdited--069.D
Sample Name: zmh-5-149-rac-ib

===

Area Percent Report

===

Sorted By : Signal
Multiplier : 1.0000
Dilution : 1.0000
Use Multiplier & Dilution Factor with ISTDs

Signal 1: DAD1 A, Sig=254,4 Ref=360,100

Peak #	RetTime [min]	Type	Width [min]	Area [mAU*s]	Height [mAU]	Area %
1	9.858	BB	0.2037	1305.13306	95.04007	50.0268
2	13.654	BB	0.2632	1303.73523	74.29480	49.9732

Totals : 2608.86829 169.33487

Signal 2: DAD1 B, Sig=210,4 Ref=360,100

Signal 3: DAD1 D, Sig=230,4 Ref=360,100

===

*** End of Report ***

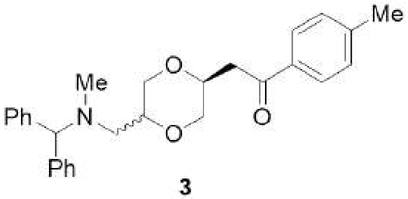

3
racemic

```
=====================================================================
Acq. Operator   : SYSTEM                        Seq. Line :  21
Sample Operator : SYSTEM
Acq. Instrument : HPLC                           Location :   P1-F-02
Injection Date  : 21/5/2024 9:45:35 pm                 Inj :   1
                                               Inj Volume : 2.000 µl
Different Inj Volume from Sample Entry!  Actual Inj Volume : 20.000 µl
Acq. Method     : C:\Users\Public\Documents\ChemStation\1\Data\SUN\SUN 2024-05-21 15-25-06
                  \IBN3-10-30.M
Last changed    : 29/8/2022 6:09:10 pm by SYSTEM
Analysis Method : C:\Users\Public\Documents\ChemStation\1\Data\SUN\SUN 2024-05-21 15-25-06
                  \IBN3-10-30.M (Sequence Method)
Last changed    : 10/10/2024 9:16:48 pm by SYSTEM
                  (modified after loading)
Additional Info : Peak(s) manually integrated
```

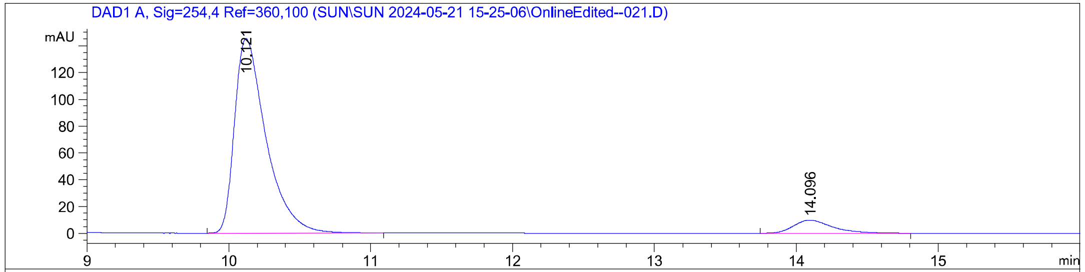

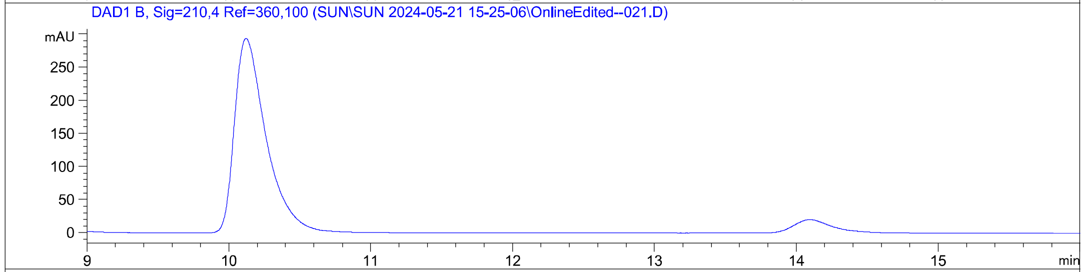

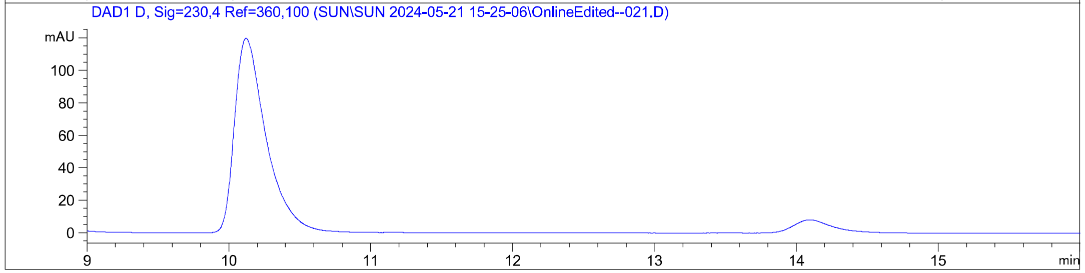

Data File C:\Users\P...\ChemStation\1\Data\SUN\SUN 2024-05-21 15-25-06\OnlineEdited--021.D
Sample Name: zmh-5-180r-20

```
=====================================================================
                         Area Percent Report
=====================================================================

Sorted By             :      Signal
Multiplier            :      1.0000
Dilution              :      1.0000
Use Multiplier & Dilution Factor with ISTDs


Signal 1: DAD1 A, Sig=254,4 Ref=360,100
```

Peak #	RetTime [min]	Type	Width [min]	Area [mAU*s]	Height [mAU]	Area %
1	10.121	BB	0.2298	2238.91333	145.28279	92.2938
2	14.096	BB	0.2599	186.94212	9.80846	7.7062

Totals : 2425.85545 155.09126

Signal 2: DAD1 B, Sig=210,4 Ref=360,100

Signal 3: DAD1 D, Sig=230,4 Ref=360,100

```
=====================================================================
                        *** End of Report ***
```

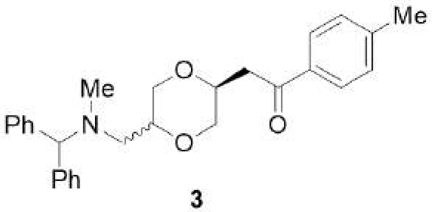

3
enantioenriched

===

Acq. Operator : SYSTEM   Seq. Line : 13
Sample Operator : SYSTEM
Acq. Instrument : HPLC   Location : P2-F-04
Injection Date : 10/10/2022 1:06:51 pm   Inj : 1
Inj Volume : 2.000 µl
Different Inj Volume from Sample Entry! Actual Inj Volume : 5.000 µl
Acq. Method : C:\Users\Public\Documents\ChemStation\1\Data\SUN\SUN 2022-10-10 10-03-12 \AY3-10-20.M
Last changed : 15/8/2022 10:25:04 pm by SYSTEM
Analysis Method : C:\Users\Public\Documents\ChemStation\1\Data\SUN\SUN 2022-10-10 10-03-12 \AY3-10-20.M (Sequence Method)
Last changed : 31/8/2023 9:33:58 pm by SYSTEM
(modified after loading)
Additional Info : Peak(s) manually integrated

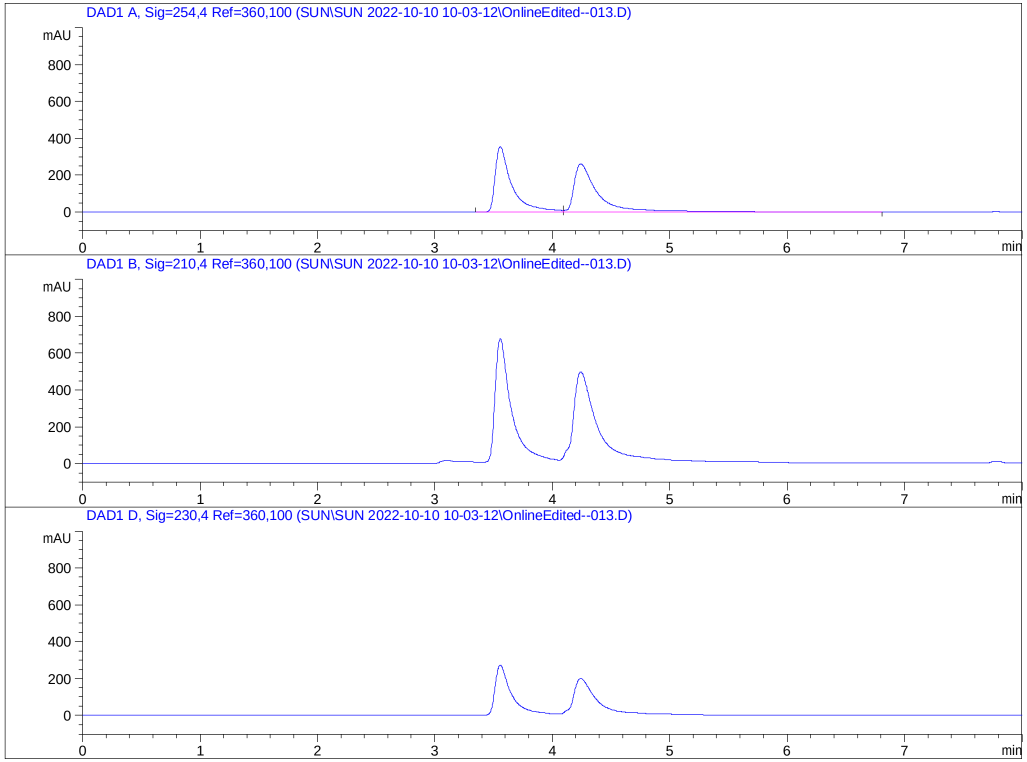

Data File C:\Users\P...\ChemStation\1\Data\SUN\SUN 2022-10-10 10-03-12\OnlineEdited--013.D
Sample Name: zmh-1-146h-rac-ay

===

Area Percent Report

===

Sorted By : Signal
Multiplier : 1.0000
Dilution : 1.0000
Use Multiplier & Dilution Factor with ISTDs

Signal 1: DAD1 A, Sig=254,4 Ref=360,100

Peak #	RetTime [min]	Type	Width [min]	Area [mAU*s]	Height [mAU]	Area %
1	3.558	BV	0.1424	3518.53076	354.23157	48.8530
2	4.243	VB	0.2014	3683.74658	260.64261	51.1470
Totals :				7202.27734	614.87418	

Signal 2: DAD1 B, Sig=210,4 Ref=360,100

Signal 3: DAD1 D, Sig=230,4 Ref=360,100

===

*** End of Report ***

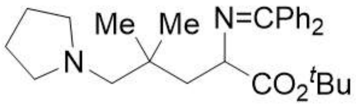

6a
racemic

Data File C:\Users\P...Documents\ChemStation\1\Data\SUN\SUN 2022-10-30 21-52-33\2AF-0201.D
Sample Name: zmh-2-1b

===

```
Acq. Operator   : SYSTEM                        Seq. Line :    2
Sample Operator : SYSTEM
Acq. Instrument : HPLC                           Location :   P2-A-06
Injection Date  : 30/10/2022 10:05:09 pm              Inj :    1
                                               Inj Volume : 2.000 µl
Different Inj Volume from Sample Entry!  Actual Inj Volume : 5.000 µl
Acq. Method     : C:\Users\Public\Documents\ChemStation\1\Data\SUN\SUN 2022-10-30 21-52-33
                  \AY3-10-20.M
Last changed    : 15/8/2022 10:25:04 pm by SYSTEM
Analysis Method : C:\Users\Public\Documents\ChemStation\1\Data\SUN\SUN 2022-10-30 21-52-33
                  \AY3-10-20.M (Sequence Method)
Last changed    : 26/1/2023 3:17:00 pm by SYSTEM
                  (modified after loading)
Additional Info : Peak(s) manually integrated
```

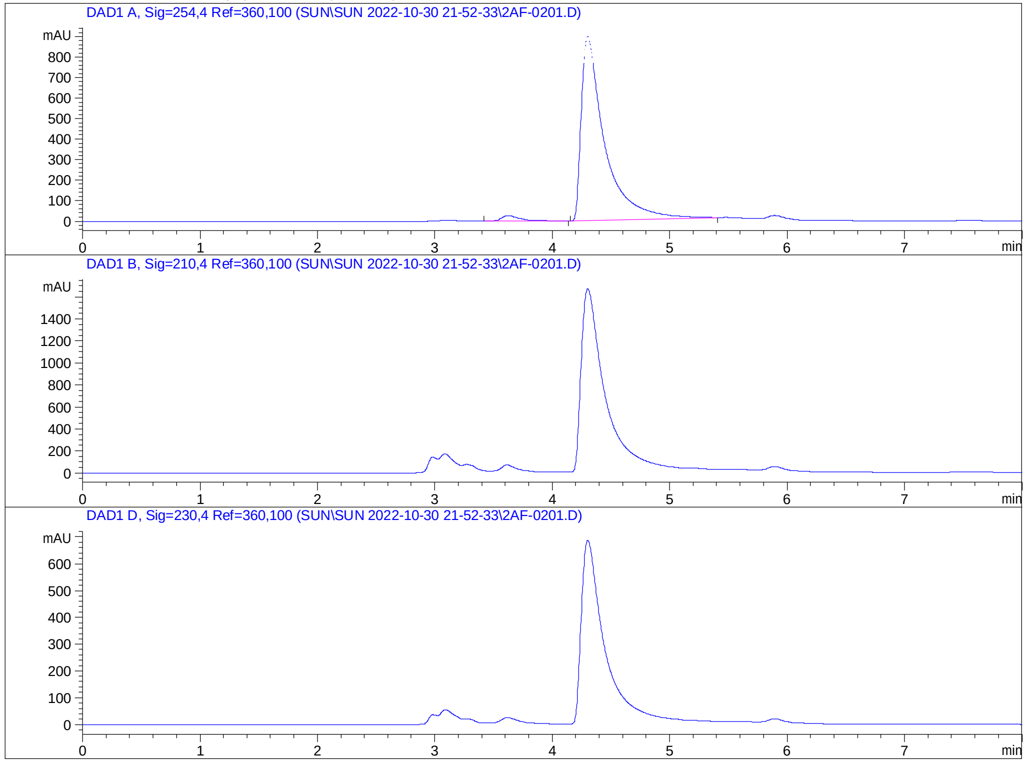

Data File C:\Users\P...Documents\ChemStation\1\Data\SUN\SUN 2022-10-30 21-52-33\2AF-0201.D
Sample Name: zmh-2-1b

===
Area Percent Report
===

```
Sorted By             :      Signal
Multiplier            :      1.0000
Dilution              :      1.0000
Use Multiplier & Dilution Factor with ISTDs
```

Signal 1: DAD1 A, Sig=254,4 Ref=360,100

Peak #	RetTime [min]	Type	Width [min]	Area [mAU*s]	Height [mAU]	Area %
1	3.623	BB	0.1632	283.46161	25.39827	2.2426
2	4.302	BB	0.1978	1.23566e4	896.51727	97.7574

Totals : 1.26400e4 921.91555

Signal 2: DAD1 B, Sig=210,4 Ref=360,100

Signal 3: DAD1 D, Sig=230,4 Ref=360,100

===
*** End of Report ***

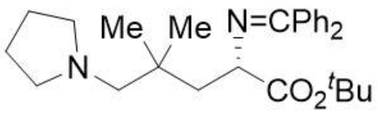

6a
enantioenriched

===

Acq. Operator : SYSTEM   Seq. Line : 8
Sample Operator : SYSTEM
Acq. Instrument : HPLC   Location : P2-A-10
Injection Date : 16/12/2022 8:04:02 pm   Inj : 1
Inj Volume : 2.000 µl
Different Inj Volume from Sample Entry! Actual Inj Volume : 5.000 µl
Acq. Method : C:\Users\Public\Documents\ChemStation\1\Data\SUN\SUN 2022-12-16 16-59-59\AY3-10-20.M
Last changed : 15/8/2022 10:25:04 pm by SYSTEM
Analysis Method : C:\Users\Public\Documents\ChemStation\1\Data\SUN\SUN 2022-12-16 16-59-59\AY3-10-20.M (Sequence Method)
Last changed : 30/1/2023 3:51:50 pm by SYSTEM
(modified after loading)
Additional Info : Peak(s) manually integrated

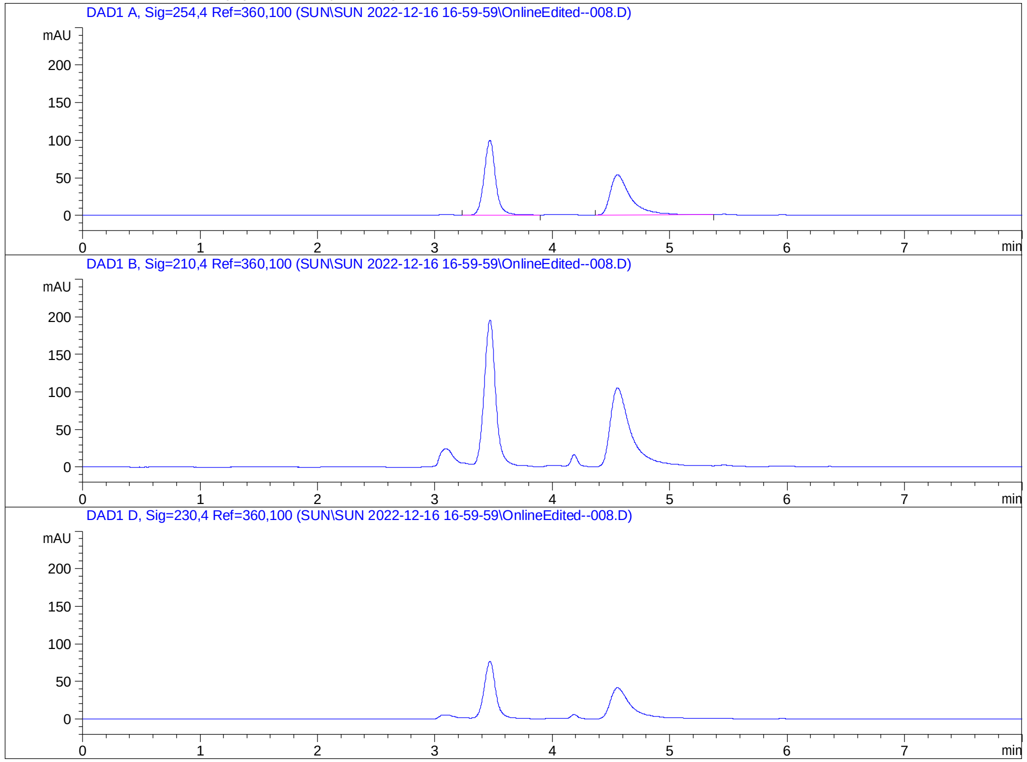

Data File C:\Users\P...\ChemStation\1\Data\SUN\SUN 2022-12-16 16-59-59\OnlineEdited--008.D
Sample Name: zmh-2-37g-rac

===

Area Percent Report

===

Sorted By : Signal
Multiplier : 1.0000
Dilution : 1.0000
Use Multiplier & Dilution Factor with ISTDs

Signal 1: DAD1 A, Sig=254,4 Ref=360,100

Peak #	RetTime [min]	Type	Width [min]	Area [mAU*s]	Height [mAU]	Area %
1	3.470	BB	0.1041	678.62183	99.44582	50.8725
2	4.557	BB	0.1789	655.34302	53.70074	49.1275

Totals : 1333.96484 153.14655

Signal 2: DAD1 B, Sig=210,4 Ref=360,100

Signal 3: DAD1 D, Sig=230,4 Ref=360,100

===

*** End of Report ***

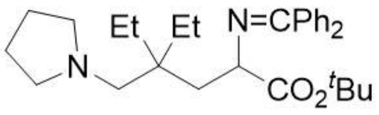

6b
racemic

===

Acq. Operator : SYSTEM Seq. Line : 45
Sample Operator : SYSTEM
Acq. Instrument : HPLC Location : P1-A-02
Injection Date : 13/1/2023 11:11:25 pm Inj : 1
Inj Volume : 2.000 µl
Different Inj Volume from Sample Entry! Actual Inj Volume : 5.000 µl
Acq. Method : C:\Users\Public\Documents\ChemStation\1\Data\SUN\SUN 2023-01-13 09-11-35 \AY3-10-20.M
Last changed : 15/8/2022 10:25:04 pm by SYSTEM
Analysis Method : C:\Users\Public\Documents\ChemStation\1\Data\SUN\SUN 2023-01-13 09-11-35 \AY3-10-20.M (Sequence Method)
Last changed : 30/1/2023 4:38:37 pm by SYSTEM
(modified after loading)
Additional Info : Peak(s) manually integrated

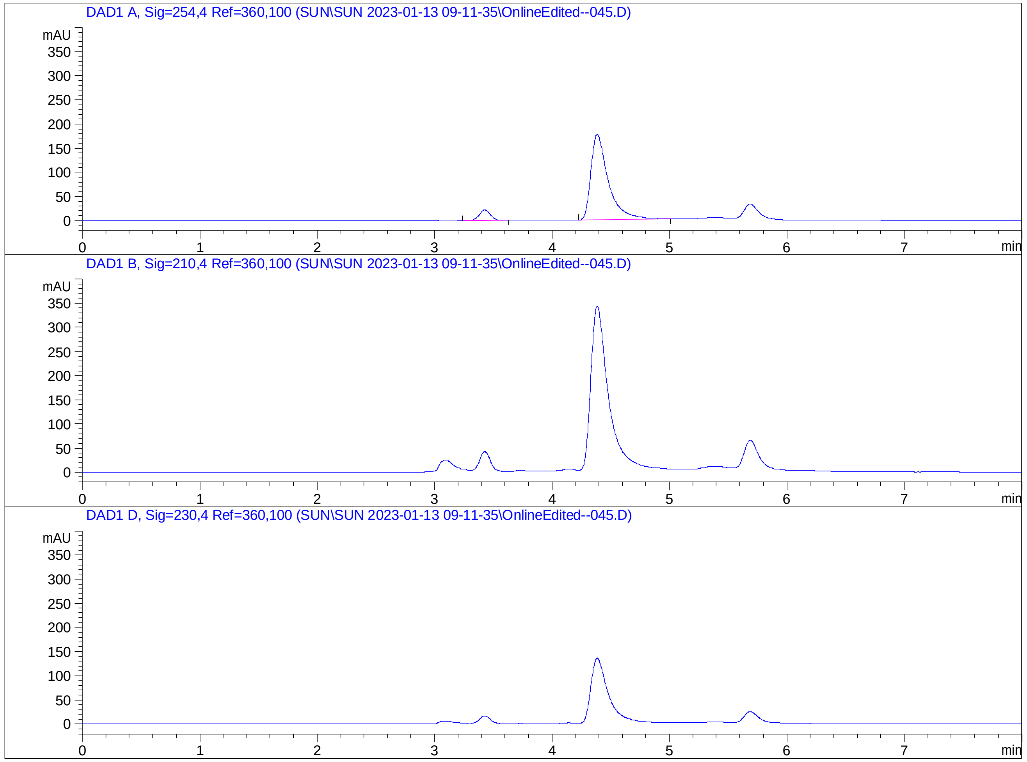

Data File C:\Users\P...\ChemStation\1\Data\SUN\SUN 2023-01-13 09-11-35\OnlineEdited--045.D
Sample Name: zmh-2-98a

===

Area Percent Report

===

Sorted By : Signal
Multiplier : 1.0000
Dilution : 1.0000
Use Multiplier & Dilution Factor with ISTDs

Signal 1: DAD1 A, Sig=254,4 Ref=360,100

Peak #	RetTime [min]	Type	Width [min]	Area [mAU*s]	Height [mAU]	Area %
1	3.428	BB	0.1009	140.97758	21.66383	7.1948
2	4.386	BB	0.1529	1818.45227	176.22015	92.8052

Totals : 1959.42986 197.88399

Signal 2: DAD1 B, Sig=210,4 Ref=360,100

Signal 3: DAD1 D, Sig=230,4 Ref=360,100

===

*** End of Report ***

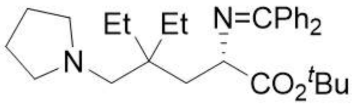

6b
enantioenriched

===

Acq. Operator : SYSTEM Seq. Line : 4
Sample Operator : SYSTEM
Acq. Instrument : HPLC Location : P2-A-11
Injection Date : 12/12/2022 9:36:48 am Inj : 1
Inj Volume : 2.000 µl
Different Inj Volume from Sample Entry! Actual Inj Volume : 5.000 µl
Acq. Method : C:\Users\Public\Documents\ChemStation\1\Data\SUN\SUN 2022-12-12 08-52-11\AY3-10-20.M
Last changed : 15/8/2022 10:25:04 pm by SYSTEM
Analysis Method : C:\Users\Public\Documents\ChemStation\1\Data\SUN\SUN 2022-12-12 08-52-11\AY3-10-20.M (Sequence Method)
Last changed : 5/9/2023 6:57:28 pm by SYSTEM
(modified after loading)
Additional Info : Peak(s) manually integrated

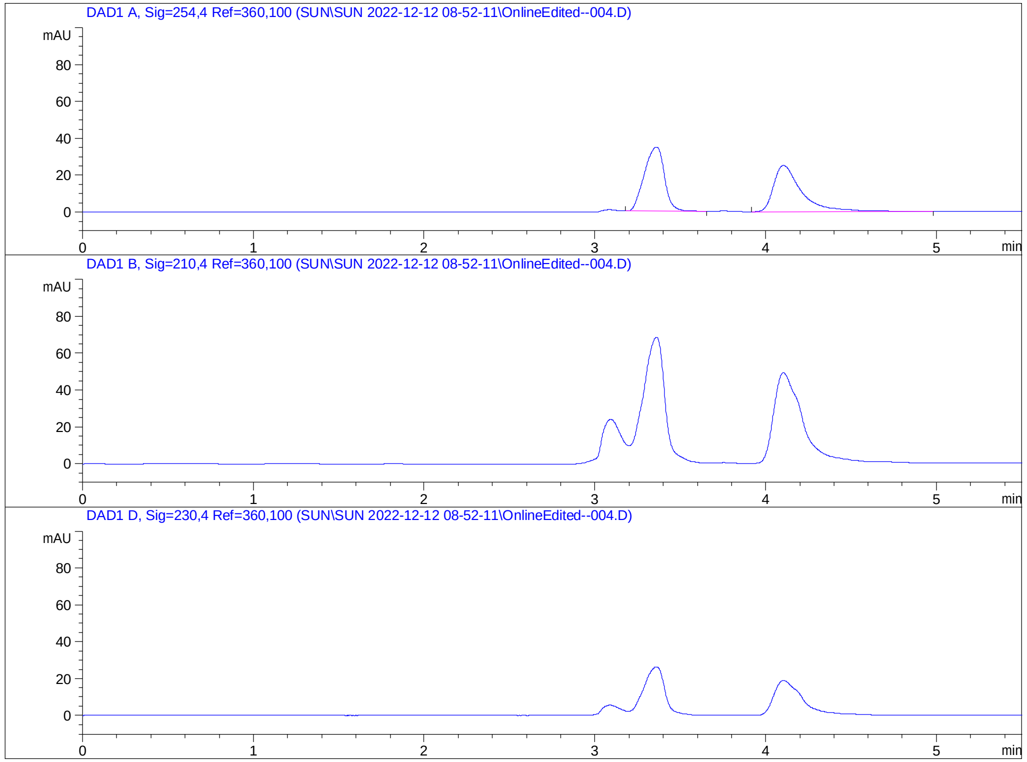

===

Area Percent Report

===

Sorted By : Signal
Multiplier : 1.0000
Dilution : 1.0000
Use Multiplier & Dilution Factor with ISTDs

Signal 1: DAD1 A, Sig=254,4 Ref=360,100

Peak #	RetTime [min]	Type	Width [min]	Area [mAU*s]	Height [mAU]	Area %
1	3.360	MM R	0.1362	284.66388	34.82896	50.0212
2	4.104	BB	0.1652	284.42261	25.19388	49.9788

Totals : 569.08649 60.02283

Signal 2: DAD1 B, Sig=210,4 Ref=360,100

Signal 3: DAD1 D, Sig=230,4 Ref=360,100

===

*** End of Report ***

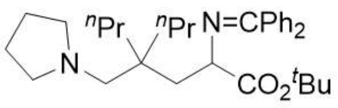

6c
racemic

===

Acq. Operator : SYSTEM Seq. Line : 20
Sample Operator : SYSTEM
Acq. Instrument : HPLC Location : P1-A-08
Injection Date : 14/1/2023 3:32:18 pm Inj : 1
Inj Volume : 2.000 µl
Different Inj Volume from Sample Entry! Actual Inj Volume : 5.000 µl
Acq. Method : C:\Users\Public\Documents\ChemStation\1\Data\SUN\SUN 2023-01-14 09-27-03 \AY3-10-20.M
Last changed : 15/8/2022 10:25:04 pm by SYSTEM
Analysis Method : C:\Users\Public\Documents\ChemStation\1\Data\SUN\SUN 2023-01-14 09-27-03 \AY3-10-20.M (Sequence Method)
Last changed : 30/1/2023 4:42:18 pm by SYSTEM
(modified after loading)
Additional Info : Peak(s) manually integrated

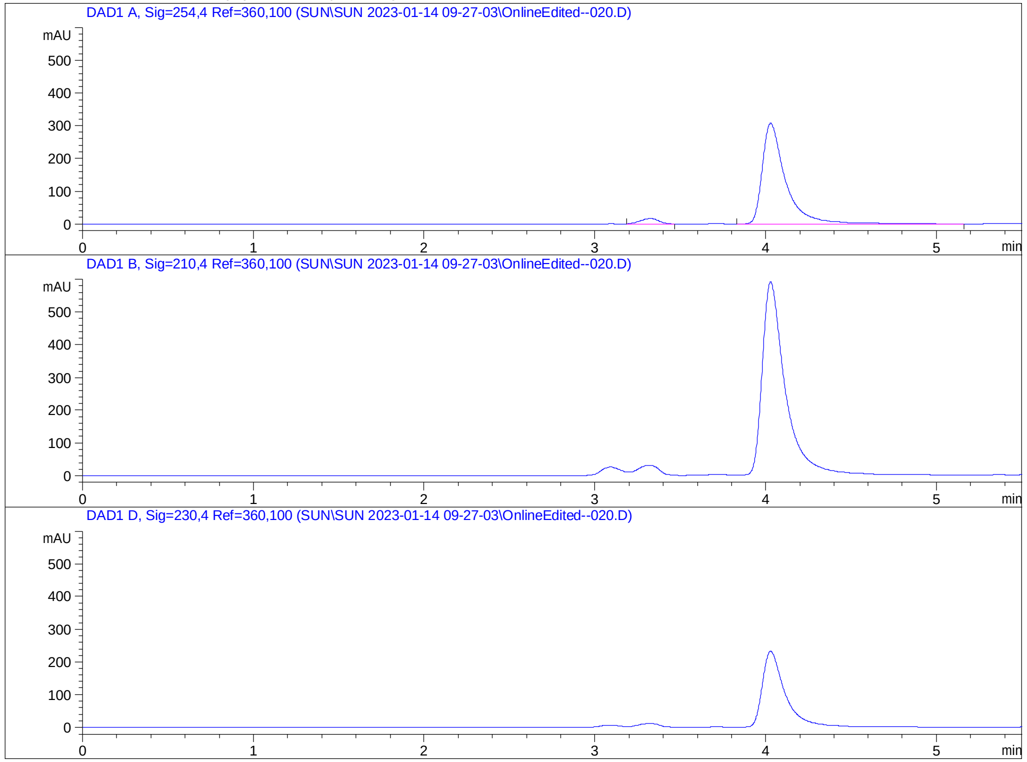

Data File C:\Users\P...\ChemStation\1\Data\SUN\SUN 2023-01-14 09-27-03\OnlineEdited--020.D
Sample Name: zmh-2-98b

===

Area Percent Report

===

Sorted By : Signal
Multiplier : 1.0000
Dilution : 1.0000
Use Multiplier & Dilution Factor with ISTDs

Signal 1: DAD1 A, Sig=254,4 Ref=360,100

Peak #	RetTime [min]	Type	Width [min]	Area [mAU*s]	Height [mAU]	Area %
1	3.325	MM R	0.1315	120.40055	15.26284	3.9934
2	4.029	BB	0.1390	2894.60645	307.11246	96.0066

Totals : 3015.00700 322.37530

Signal 2: DAD1 B, Sig=210,4 Ref=360,100

Signal 3: DAD1 D, Sig=230,4 Ref=360,100

===

*** End of Report ***

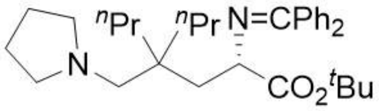

6c
enantioenriched

===

Acq. Operator : SYSTEM Seq. Line : 12
Sample Operator : SYSTEM
Acq. Instrument : HPLC Location : P2-A-08
Injection Date : 16/12/2022 9:08:00 pm Inj : 1
Inj Volume : 2.000 µl
Different Inj Volume from Sample Entry! Actual Inj Volume : 5.000 µl
Acq. Method : C:\Users\Public\Documents\ChemStation\1\Data\SUN\SUN 2022-12-16 16-59-59 \AY3-10-20.M
Last changed : 15/8/2022 10:25:04 pm by SYSTEM
Analysis Method : C:\Users\Public\Documents\ChemStation\1\Data\SUN\SUN 2022-12-16 16-59-59 \AY3-10-20.M (Sequence Method)
Last changed : 30/1/2023 3:58:13 pm by SYSTEM
(modified after loading)
Additional Info : Peak(s) manually integrated

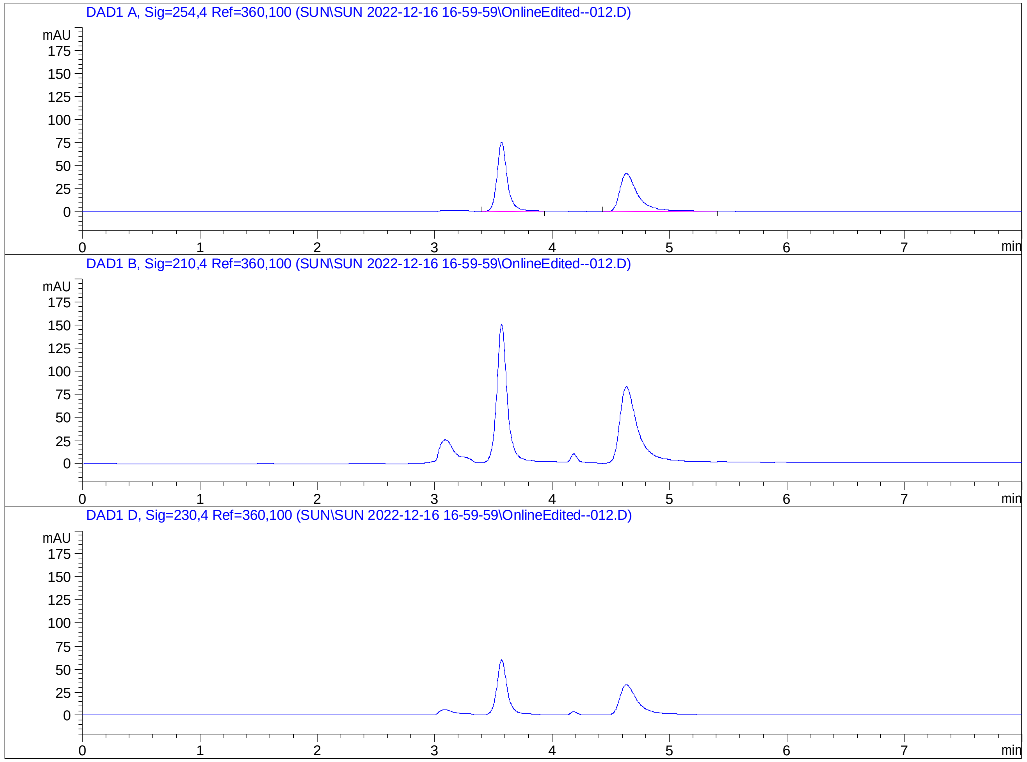

===

Area Percent Report

===

Sorted By : Signal
Multiplier : 1.0000
Dilution : 1.0000
Use Multiplier & Dilution Factor with ISTDs

Signal 1: DAD1 A, Sig=254,4 Ref=360,100

Peak #	RetTime [min]	Type	Width [min]	Area [mAU*s]	Height [mAU]	Area %
1	3.572	BB	0.0918	456.98499	74.80676	50.6011
2	4.634	BB	0.1594	446.12698	41.33090	49.3989

Totals : 903.11197 116.13765

Signal 2: DAD1 B, Sig=210,4 Ref=360,100

Signal 3: DAD1 D, Sig=230,4 Ref=360,100

===

*** End of Report ***

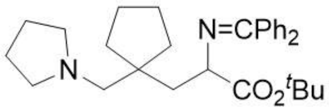

6d
racemic

===

Acq. Operator : SYSTEM Seq. Line : 24
Sample Operator : SYSTEM
Acq. Instrument : HPLC Location : P2-E-11
Injection Date : 17/12/2022 2:20:26 am Inj : 1
Inj Volume : 2.000 µl
Different Inj Volume from Sample Entry! Actual Inj Volume : 5.000 µl
Acq. Method : C:\Users\Public\Documents\ChemStation\1\Data\SUN\SUN 2022-12-16 16-59-59\AY3-10-20.M
Last changed : 15/8/2022 10:25:04 pm by SYSTEM
Analysis Method : C:\Users\Public\Documents\ChemStation\1\Data\SUN\SUN 2022-12-16 16-59-59\AY3-10-20.M (Sequence Method)
Last changed : 30/1/2023 4:10:45 pm by SYSTEM
(modified after loading)
Additional Info : Peak(s) manually integrated

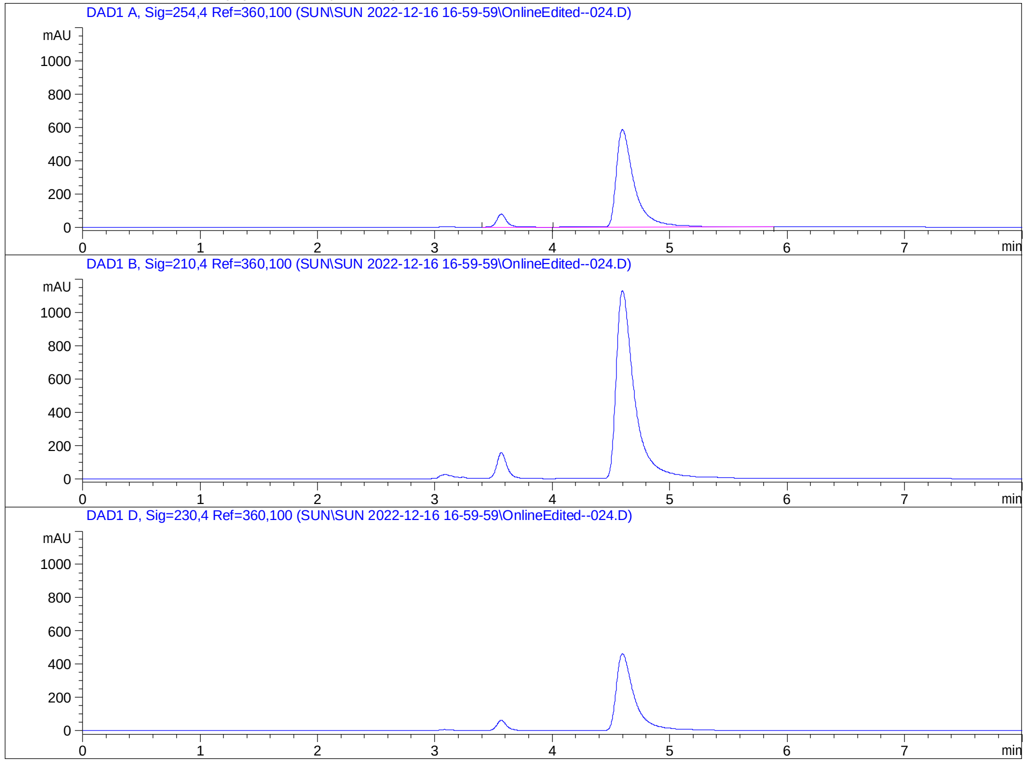

Data File C:\Users\P...\ChemStation\1\Data\SUN\SUN 2022-12-16 16-59-59\OnlineEdited--024.D
Sample Name: zmh-2-4a-chiral

===

Area Percent Report

===

Sorted By : Signal
Multiplier : 1.0000
Dilution : 1.0000
Use Multiplier & Dilution Factor with ISTDs

Signal 1: DAD1 A, Sig=254,4 Ref=360,100

Peak #	RetTime [min]	Type	Width [min]	Area [mAU*s]	Height [mAU]	Area %
1	3.567	BB	0.0899	468.85132	78.25301	6.9080
2	4.599	VB R	0.1586	6318.24561	585.23700	93.0920

Totals : 6787.09692 663.49001

Signal 2: DAD1 B, Sig=210,4 Ref=360,100

Signal 3: DAD1 D, Sig=230,4 Ref=360,100

===

*** End of Report ***

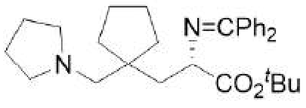

6d
enantioenriched

===

Acq. Operator : SYSTEM Seq. Line : 2
Sample Operator : SYSTEM
Acq. Instrument : HPLC Location : P2-B-04
Injection Date : 3/11/2022 9:42:09 am Inj : 1
Inj Volume : 2.000 µl
Different Inj Volume from Sample Entry! Actual Inj Volume : 20.000 µl
Acq. Method : C:\Users\Public\Documents\ChemStation\1\Data\SUN\SUN 2022-11-03 09-29-26\AY3-10-20.M
Last changed : 15/8/2022 10:25:04 pm by SYSTEM
Analysis Method : C:\Users\Public\Documents\ChemStation\1\Data\SUN\SUN 2022-11-03 09-29-26\AY3-10-20.M (Sequence Method)
Last changed : 5/9/2023 7:07:46 pm by SYSTEM
(modified after loading)
Additional Info : Peak(s) manually integrated

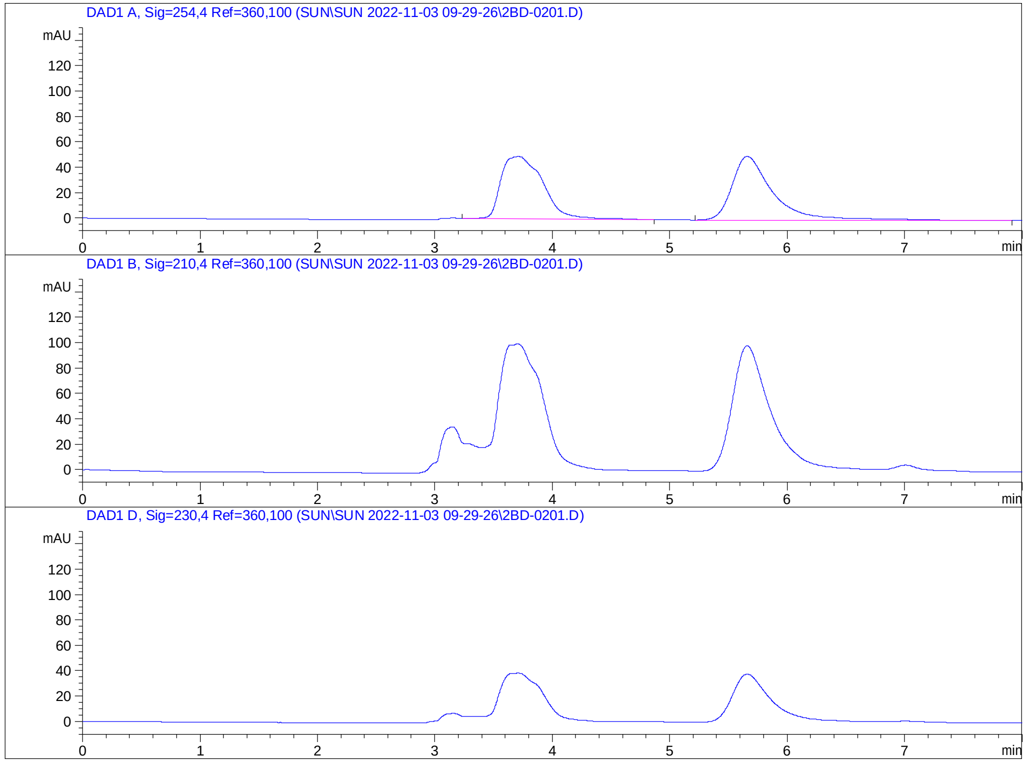

Data File C:\Users\P...Documents\ChemStation\1\Data\SUN\SUN 2022-11-03 09-29-26\2BD-0201.D
Sample Name: zmh-2-1c-rac

===

Area Percent Report

===

Sorted By : Signal
Multiplier : 1.0000
Dilution : 1.0000
Use Multiplier & Dilution Factor with ISTDs

Signal 1: DAD1 A, Sig=254,4 Ref=360,100

Peak #	RetTime [min]	Type	Width [min]	Area [mAU*s]	Height [mAU]	Area %
1	3.712	BB	0.3205	1209.46619	49.32903	50.0926
2	5.661	BB	0.3488	1204.99304	50.15105	49.9074

Totals : 2414.45923 99.48008

Signal 2: DAD1 B, Sig=210,4 Ref=360,100

Signal 3: DAD1 D, Sig=230,4 Ref=360,100

===

*** End of Report ***

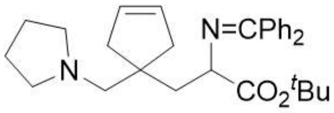

6e
racemic

===

Acq. Operator : SYSTEM Seq. Line : 4
Sample Operator : SYSTEM
Acq. Instrument : HPLC Location : P2-B-05
Injection Date : 3/11/2022 10:14:15 am Inj : 1
Inj Volume : 2.000 µl
Different Inj Volume from Sample Entry! Actual Inj Volume : 10.000 µl
Acq. Method : C:\Users\Public\Documents\ChemStation\1\Data\SUN\SUN 2022-11-03 09-29-26\AY3-10-20.M
Last changed : 15/8/2022 10:25:04 pm by SYSTEM
Analysis Method : C:\Users\Public\Documents\ChemStation\1\Data\SUN\SUN 2022-11-03 09-29-26\AY3-10-20.M (Sequence Method)
Last changed : 31/8/2023 9:41:12 pm by SYSTEM
(modified after loading)
Additional Info : Peak(s) manually integrated

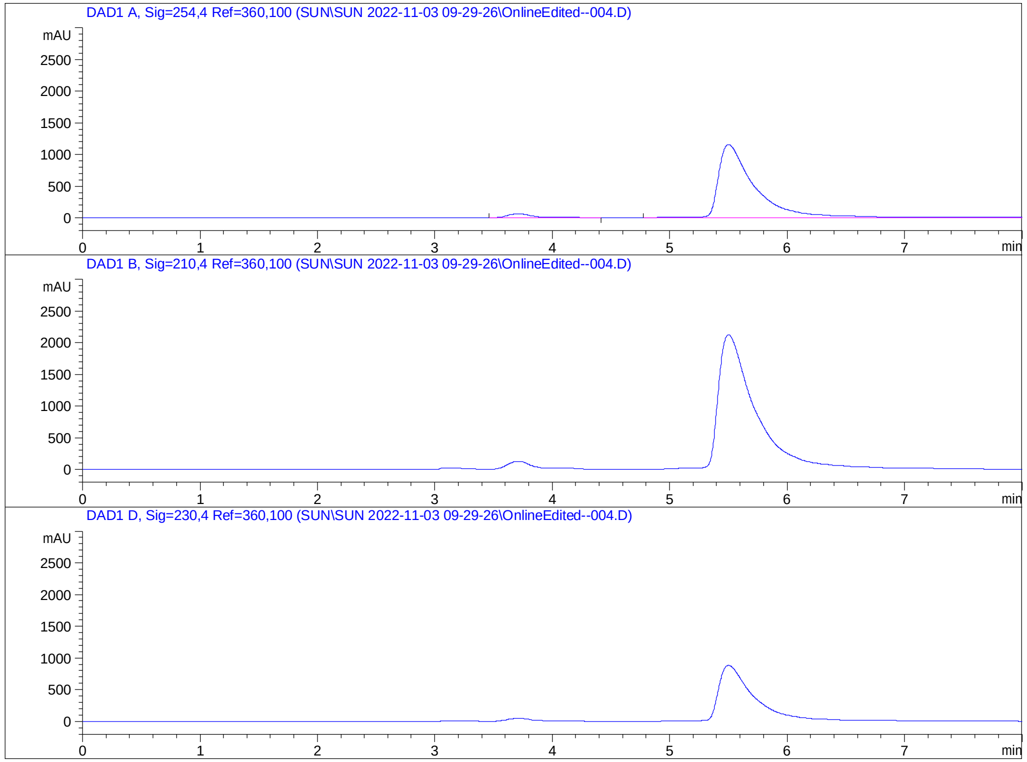

Data File C:\Users\P...\ChemStation\1\Data\SUN\SUN 2022-11-03 09-29-26\OnlineEdited--004.D
Sample Name: zmh-2-1c-chiral

===
Area Percent Report
===

Sorted By : Signal
Multiplier : 1.0000
Dilution : 1.0000
Use Multiplier & Dilution Factor with ISTDs

Signal 1: DAD1 A, Sig=254,4 Ref=360,100

Peak #	RetTime [min]	Type	Width [min]	Area [mAU*s]	Height [mAU]	Area %
1	3.714	BB	0.2203	899.00372	62.31496	3.5039
2	5.501	BB	0.3093	2.47581e4	1151.64990	96.4961

Totals : 2.56571e4 1213.96486

Signal 2: DAD1 B, Sig=210,4 Ref=360,100

Signal 3: DAD1 D, Sig=230,4 Ref=360,100

===
*** End of Report ***

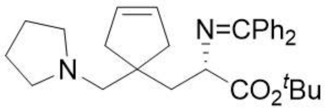

6e
enantioenriched

===

Acq. Operator : SYSTEM Seq. Line : 10
Sample Operator : SYSTEM
Acq. Instrument : HPLC Location : P2-B-05
Injection Date : 19/11/2022 9:10:41 pm Inj : 1
Inj Volume : 2.000 µl
Different Inj Volume from Sample Entry! Actual Inj Volume : 20.000 µl
Acq. Method : C:\Users\Public\Documents\ChemStation\1\Data\SUN\SUN 2022-11-19 18-49-23\IE3-10-20.M
Last changed : 15/8/2022 10:27:27 pm by SYSTEM
Analysis Method : C:\Users\Public\Documents\ChemStation\1\Data\SUN\SUN 2022-11-19 18-49-23\IE3-10-20.M (Sequence Method)
Last changed : 26/1/2023 3:49:34 pm by SYSTEM
(modified after loading)
Additional Info : Peak(s) manually integrated

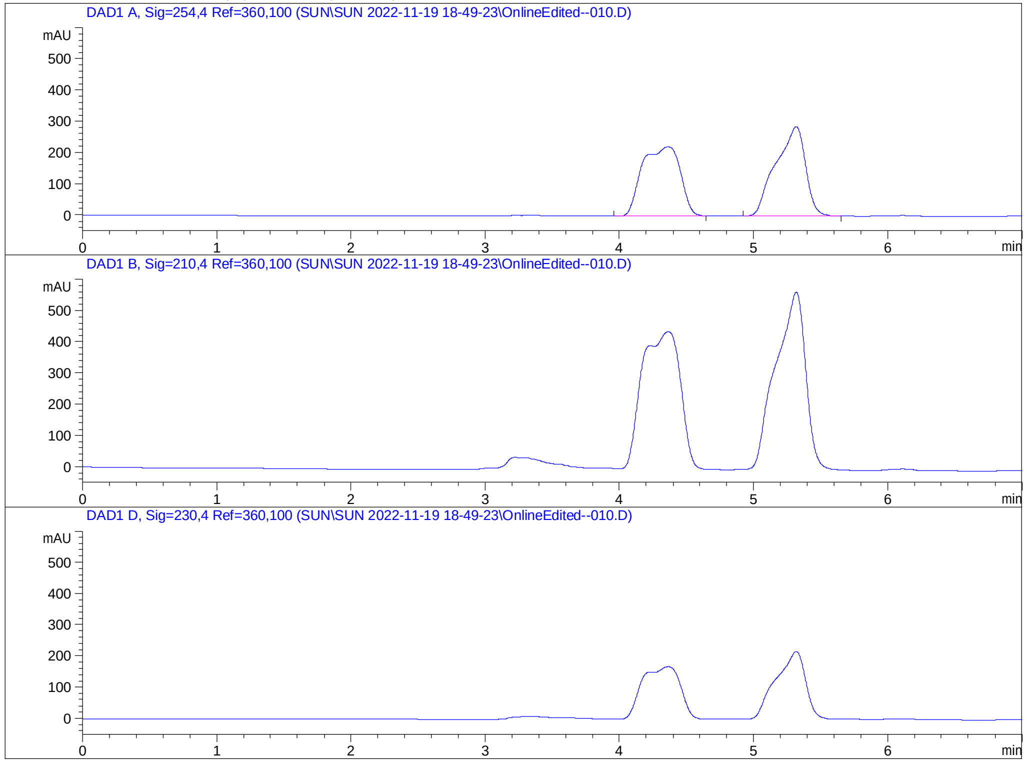

Data File C:\Users\P...\ChemStation\1\Data\SUN\SUN 2022-11-19 18-49-23\OnlineEdited--010.D
Sample Name: zmh-2-28a-rac-ie

===

Area Percent Report

===

Sorted By : Signal
Multiplier : 1.0000
Dilution : 1.0000
Use Multiplier & Dilution Factor with ISTDs

Signal 1: DAD1 A, Sig=254,4 Ref=360,100

Peak #	RetTime [min]	Type	Width [min]	Area [mAU*s]	Height [mAU]	Area %
1	4.364	MM	0.3276	4366.63770	222.15614	49.8871
2	5.318	MM	0.2553	4386.39502	286.32452	50.1129
Totals :				8753.03271	508.48067	

Signal 2: DAD1 B, Sig=210,4 Ref=360,100

Signal 3: DAD1 D, Sig=230,4 Ref=360,100

===

*** End of Report ***

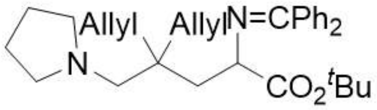

6f

racemic

Data File C:\Users\P...\ChemStation\1\Data\SUN\SUN 2022-11-19 18-49-23\OnlineEdited--014.D
Sample Name: zmh-2-4b-chiral

===

Acq. Operator : SYSTEM Seq. Line : 14
Sample Operator : SYSTEM
Acq. Instrument : HPLC Location : P2-A-06
Injection Date : 19/11/2022 10:24:57 pm Inj : 1
Inj Volume : 2.000 µl
Different Inj Volume from Sample Entry! Actual Inj Volume : 10.000 µl
Acq. Method : C:\Users\Public\Documents\ChemStation\1\Data\SUN\SUN 2022-11-19 18-49-23 \IE3-10-20.M
Last changed : 15/8/2022 10:27:27 pm by SYSTEM
Analysis Method : C:\Users\Public\Documents\ChemStation\1\Data\SUN\SUN 2022-11-19 18-49-23 \IE3-10-20.M (Sequence Method)
Last changed : 26/1/2023 3:52:59 pm by SYSTEM
(modified after loading)
Additional Info : Peak(s) manually integrated

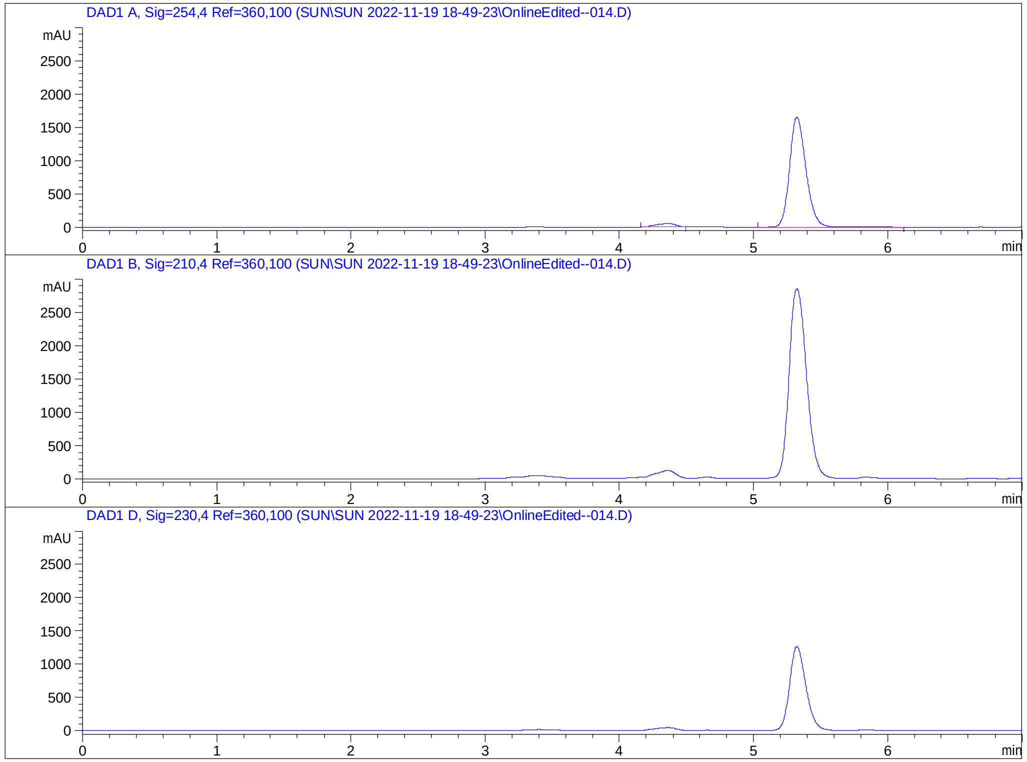

Data File C:\Users\P...\ChemStation\1\Data\SUN\SUN 2022-11-19 18-49-23\OnlineEdited--014.D
Sample Name: zmh-2-4b-chiral

===
Area Percent Report
===

Sorted By : Signal
Multiplier : 1.0000
Dilution : 1.0000
Use Multiplier & Dilution Factor with ISTDs

Signal 1: DAD1 A, Sig=254,4 Ref=360,100

Peak #	RetTime [min]	Type	Width [min]	Area [mAU*s]	Height [mAU]	Area %
1	4.362	MM	0.1670	565.91101	56.47709	3.8714
2	5.322	BV R	0.1308	1.40518e4	1652.16797	96.1286

Totals : 1.46177e4 1708.64505

Signal 2: DAD1 B, Sig=210,4 Ref=360,100

Signal 3: DAD1 D, Sig=230,4 Ref=360,100

===
*** End of Report ***

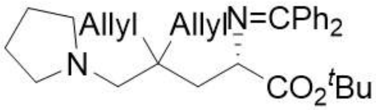

6f
enantioenriched

Data File C:\Users\P...ents\ChemStation\1\Data\SUN\SUN 2022-11-20 13-46-25\2AJ-0401--005.D
Sample Name: zmh-2-37d-rac

===

Acq. Operator : SYSTEM Seq. Line : 5
Sample Operator : SYSTEM
Acq. Instrument : HPLC Location : P2-A-10
Injection Date : 20/11/2022 2:54:02 pm Inj : 1
Inj Volume : 2.000 µl
Different Inj Volume from Sample Entry! Actual Inj Volume : 20.000 µl
Acq. Method : C:\Users\Public\Documents\ChemStation\1\Data\SUN\SUN 2022-11-20 13-46-25 \AY3-10-20.M
Last changed : 15/8/2022 10:25:04 pm by SYSTEM
Analysis Method : C:\Users\Public\Documents\ChemStation\1\Data\SUN\SUN 2022-11-20 13-46-25 \AY3-10-20.M (Sequence Method)
Last changed : 26/1/2023 3:57:07 pm by SYSTEM
(modified after loading)
Additional Info : Peak(s) manually integrated

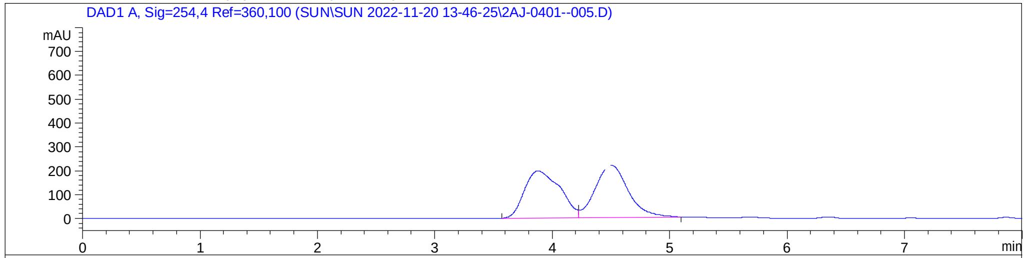

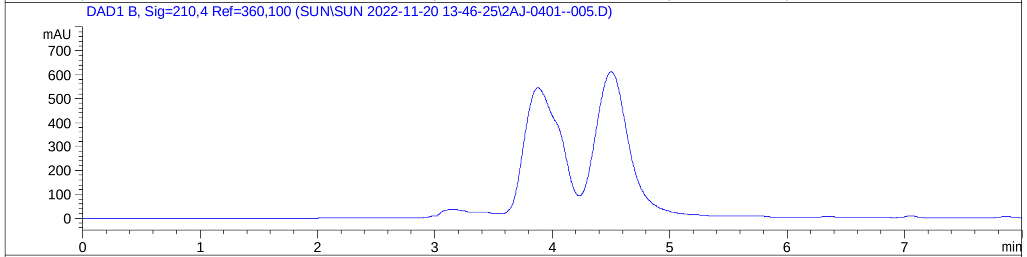

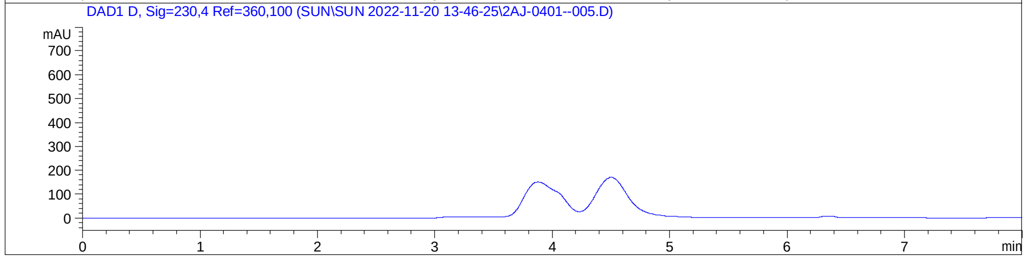

Data File C:\Users\P...ents\ChemStation\1\Data\SUN\SUN 2022-11-20 13-46-25\2AJ-0401--005.D
Sample Name: zmh-2-37d-rac

===

Area Percent Report

===

Sorted By : Signal
Multiplier : 1.0000
Dilution : 1.0000
Use Multiplier & Dilution Factor with ISTDs

Signal 1: DAD1 A, Sig=254,4 Ref=360,100

Peak #	RetTime [min]	Type	Width [min]	Area [mAU*s]	Height [mAU]	Area %
1	3.878	MM	0.3508	4158.44043	197.59575	49.0825
2	4.502	MM	0.3258	4313.90283	220.66156	50.9175

Totals : 8472.34326 418.25731

Signal 2: DAD1 B, Sig=210,4 Ref=360,100

Signal 3: DAD1 D, Sig=230,4 Ref=360,100

===

*** End of Report ***

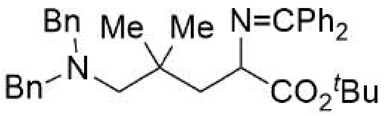

6g
racemic

===

Acq. Operator : SYSTEM   Seq. Line : 12
Sample Operator : SYSTEM
Acq. Instrument : HPLC   Location : P2-A-06
Injection Date : 20/11/2022 9:25:25 pm   Inj : 1
Inj Volume : 2.000 µl
Different Inj Volume from Sample Entry! Actual Inj Volume : 10.000 µl
Acq. Method : C:\Users\Public\Documents\ChemStation\1\Data\SUN\SUN 2022-11-20 16-01-48 \AY3-10-20.M
Last changed : 15/8/2022 10:25:04 pm by SYSTEM
Analysis Method : C:\Users\Public\Documents\ChemStation\1\Data\SUN\SUN 2022-11-20 16-01-48 \AY3-10-20.M (Sequence Method)
Last changed : 26/1/2023 4:15:22 pm by SYSTEM
(modified after loading)
Additional Info : Peak(s) manually integrated

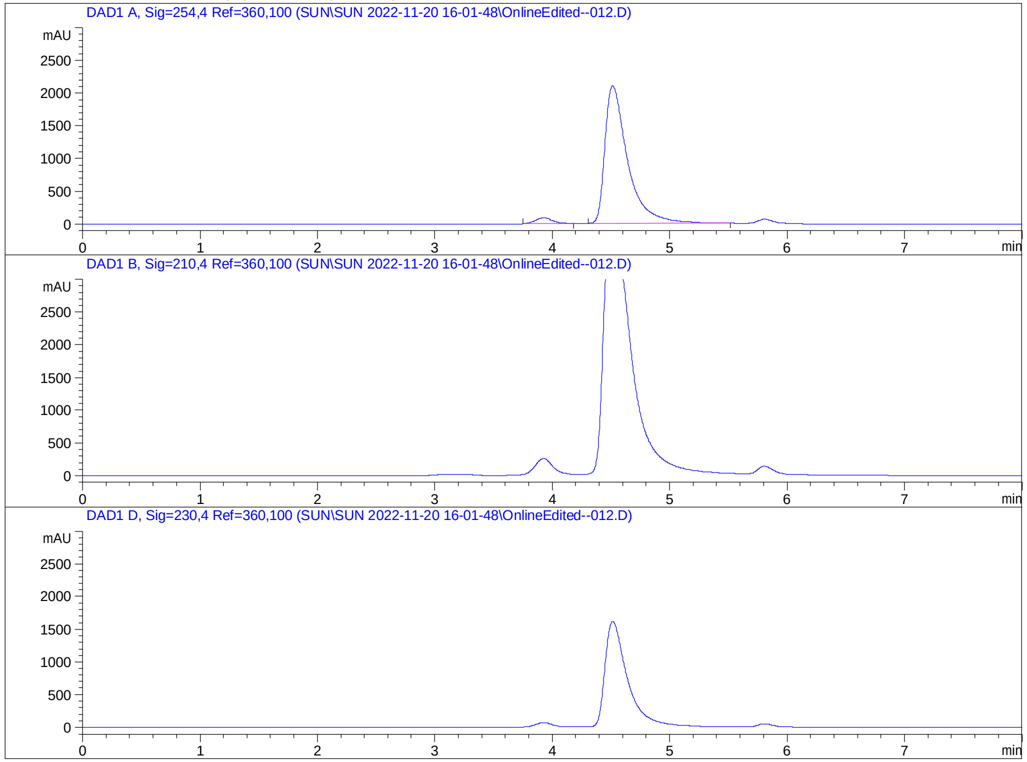

Data File C:\Users\P...\ChemStation\1\Data\SUN\SUN 2022-11-20 16-01-48\OnlineEdited--012.D
Sample Name: zmh-2-21g-chiral

===

Area Percent Report

===

Sorted By : Signal
Multiplier : 1.0000
Dilution : 1.0000
Use Multiplier & Dilution Factor with ISTDs

Signal 1: DAD1 A, Sig=254,4 Ref=360,100

Peak #	RetTime [min]	Type	Width [min]	Area [mAU*s]	Height [mAU]	Area %
1	3.927	MM R	0.1707	915.96759	89.40687	3.1420
2	4.515	MM R	0.2240	2.82361e4	2101.01001	96.8580

Totals : 2.91521e4 2190.41688

Signal 2: DAD1 B, Sig=210,4 Ref=360,100

Signal 3: DAD1 D, Sig=230,4 Ref=360,100

===

*** End of Report ***

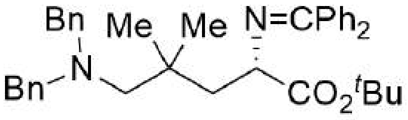

6g
enantioenriched

===

Acq. Operator : SYSTEM Seq. Line : 10
Sample Operator : SYSTEM
Acq. Instrument : HPLC Location : P2-B-02
Injection Date : 10/11/2022 1:31:17 pm Inj : 1
Inj Volume : 2.000 µl
Different Inj Volume from Sample Entry! Actual Inj Volume : 5.000 µl
Acq. Method : C:\Users\Public\Documents\ChemStation\1\Data\SUN\SUN 2022-11-10 09-46-15 \OD3-10-20.M
Last changed : 15/8/2022 10:27:52 pm by SYSTEM
Analysis Method : C:\Users\Public\Documents\ChemStation\1\Data\SUN\SUN 2022-11-10 09-46-15 \OD3-10-20.M (Sequence Method)
Last changed : 5/9/2023 7:19:52 pm by SYSTEM
(modified after loading)
Additional Info : Peak(s) manually integrated

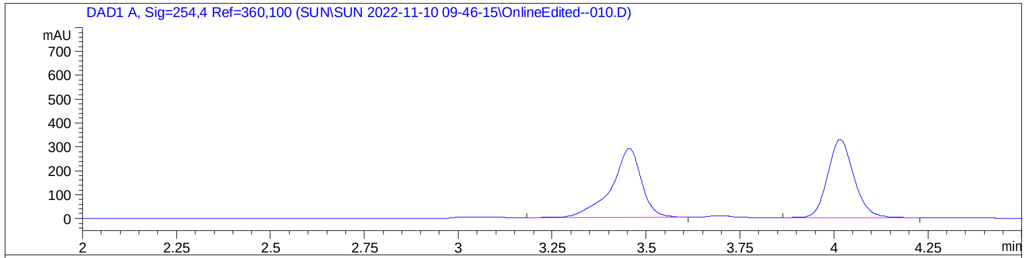

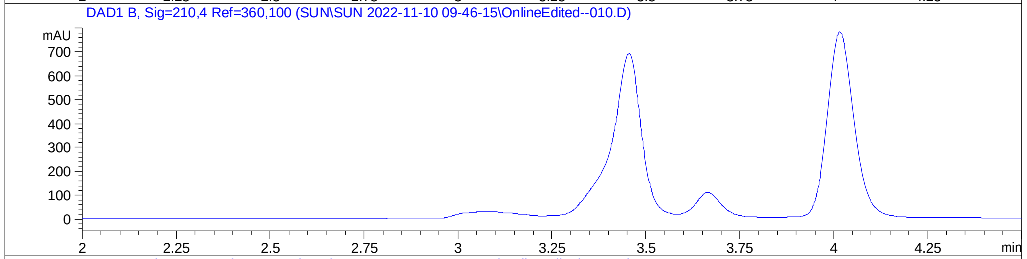

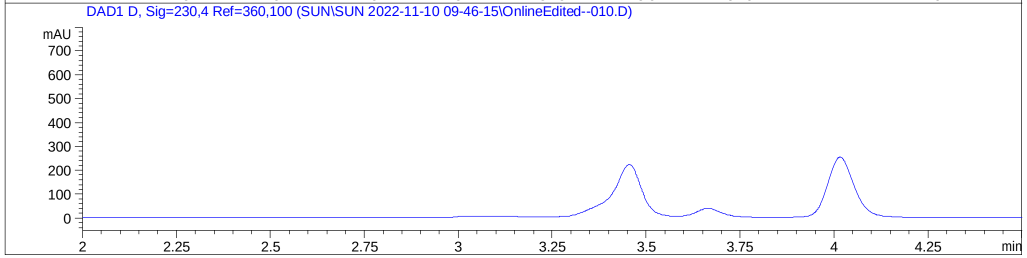

Data File C:\Users\P...\ChemStation\1\Data\SUN\SUN 2022-11-10 09-46-15\OnlineEdited--010.D
Sample Name: zmh-2-20c-rac-od

===

Area Percent Report

===

Sorted By : Signal
Multiplier : 1.0000
Dilution : 1.0000
Use Multiplier & Dilution Factor with ISTDs

Signal 1: DAD1 A, Sig=254,4 Ref=360,100

Peak #	RetTime [min]	Type	Width [min]	Area [mAU*s]	Height [mAU]	Area %
1	3.455	MM	0.0934	1621.80139	289.42026	50.1712
2	4.016	MM	0.0817	1610.73145	328.49796	49.8288

Totals : 3232.53284 617.91821

Signal 2: DAD1 B, Sig=210,4 Ref=360,100

Signal 3: DAD1 D, Sig=230,4 Ref=360,100

===

*** End of Report ***

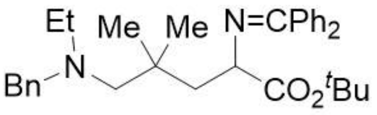

6h
racemic

Data File C:\Users\P...\ChemStation\1\Data\SUN\SUN 2022-11-10 09-46-15\OnlineEdited--013.D
Sample Name: zmh-2-4e-chiral

===

Acq. Operator : SYSTEM Seq. Line : 13
Sample Operator : SYSTEM
Acq. Instrument : HPLC Location : P2-B-03
Injection Date : 10/11/2022 2:14:30 pm Inj : 1
Inj Volume : 2.000 µl
Different Inj Volume from Sample Entry! Actual Inj Volume : 5.000 µl
Acq. Method : C:\Users\Public\Documents\ChemStation\1\Data\SUN\SUN 2022-11-10 09-46-15 \OD3-10-20.M
Last changed : 15/8/2022 10:27:52 pm by SYSTEM
Analysis Method : C:\Users\Public\Documents\ChemStation\1\Data\SUN\SUN 2022-11-10 09-46-15 \OD3-10-20.M (Sequence Method)
Last changed : 5/9/2023 7:22:12 pm by SYSTEM
(modified after loading)
Additional Info : Peak(s) manually integrated

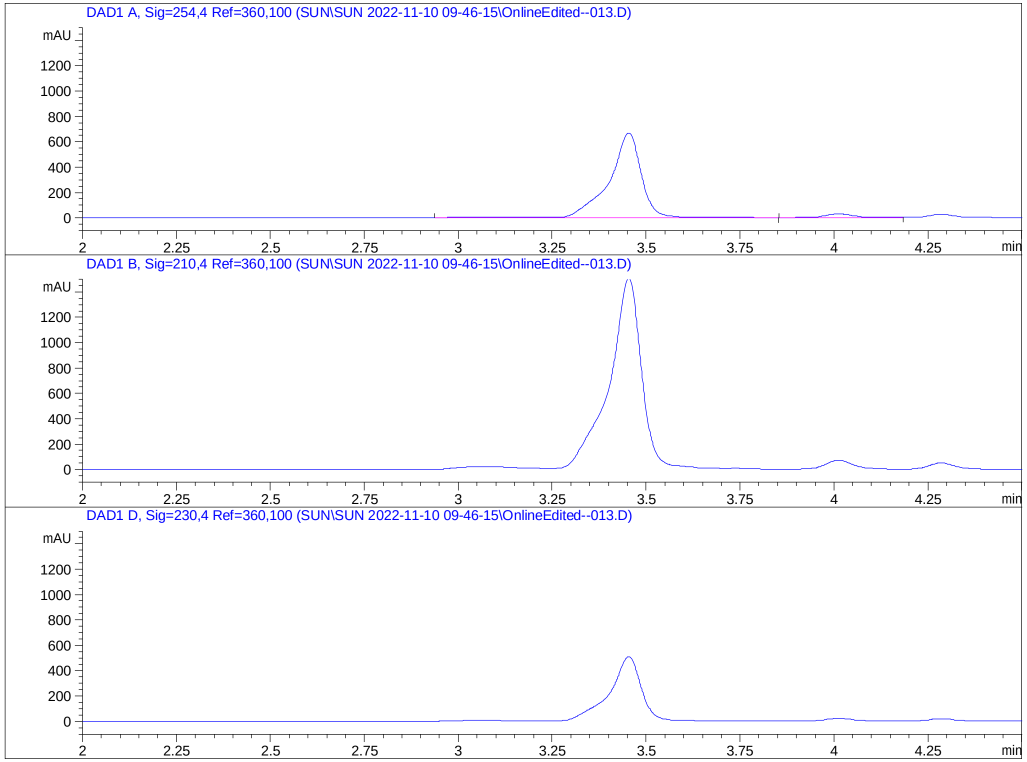

Data File C:\Users\P...\ChemStation\1\Data\SUN\SUN 2022-11-10 09-46-15\OnlineEdited--013.D
Sample Name: zmh-2-4e-chiral

===

Area Percent Report

===

Sorted By : Signal
Multiplier : 1.0000
Dilution : 1.0000
Use Multiplier & Dilution Factor with ISTDs

Signal 1: DAD1 A, Sig=254,4 Ref=360,100

Peak #	RetTime [min]	Type	Width [min]	Area [mAU*s]	Height [mAU]	Area %
1	3.454	VB R	0.0839	3944.50732	667.93805	96.3220
2	4.013	BV	0.0774	150.61684	29.85502	3.6780

Totals : 4095.12416 697.79307

Signal 2: DAD1 B, Sig=210,4 Ref=360,100

Signal 3: DAD1 D, Sig=230,4 Ref=360,100

===

*** End of Report ***

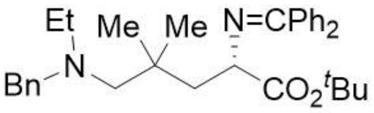

6h
enantioenriched

===

Acq. Operator : SYSTEM Seq. Line : 6
Sample Operator : SYSTEM
Acq. Instrument : HPLC Location : P2-A-11
Injection Date : 16/12/2022 7:32:04 pm Inj : 1
Inj Volume : 2.000 µl
Different Inj Volume from Sample Entry! Actual Inj Volume : 5.000 µl
Acq. Method : C:\Users\Public\Documents\ChemStation\1\Data\SUN\SUN 2022-12-16 16-59-59\AY3-10-20.M
Last changed : 15/8/2022 10:25:04 pm by SYSTEM
Analysis Method : C:\Users\Public\Documents\ChemStation\1\Data\SUN\SUN 2022-12-16 16-59-59\AY3-10-20.M (Sequence Method)
Last changed : 30/1/2023 3:51:50 pm by SYSTEM
(modified after loading)
Additional Info : Peak(s) manually integrated

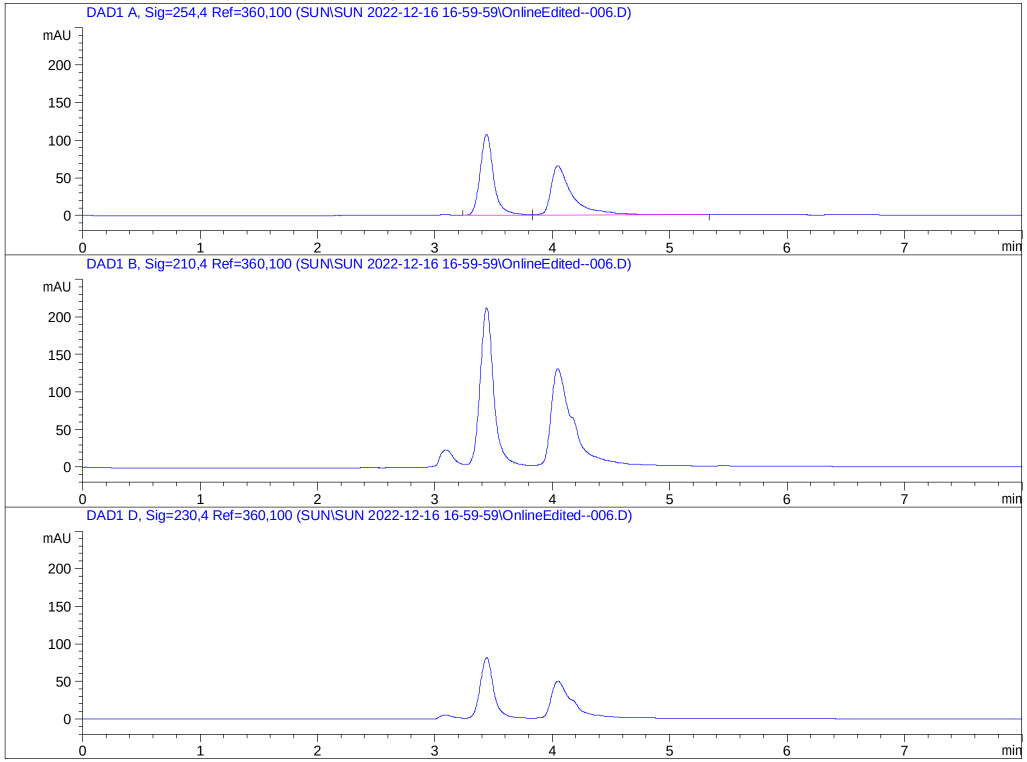

Data File C:\Users\P...\ChemStation\1\Data\SUN\SUN 2022-12-16 16-59-59\OnlineEdited--006.D
Sample Name: zmh-2-37e-rac

===
Area Percent Report
===

Sorted By : Signal
Multiplier : 1.0000
Dilution : 1.0000
Use Multiplier & Dilution Factor with ISTDs

Signal 1: DAD1 A, Sig=254,4 Ref=360,100

Peak #	RetTime [min]	Type	Width [min]	Area [mAU*s]	Height [mAU]	Area %
1	3.442	BV	0.1223	869.45850	107.52095	50.4630
2	4.048	VB	0.1842	853.50360	66.07687	49.5370

Totals : 1722.96210 173.59782

Signal 2: DAD1 B, Sig=210,4 Ref=360,100

Signal 3: DAD1 D, Sig=230,4 Ref=360,100

===
*** End of Report ***

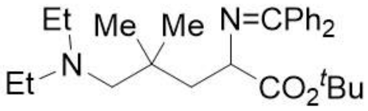

6i
racemic

```
=====================================================================
Acq. Operator   : SYSTEM                        Seq. Line :  22
Sample Operator : SYSTEM
Acq. Instrument : HPLC                           Location :   P2-D-11
Injection Date  : 17/12/2022 1:48:24 am               Inj :   1
                                               Inj Volume : 2.000 µl
Different Inj Volume from Sample Entry!  Actual Inj Volume : 5.000 µl
Acq. Method     : C:\Users\Public\Documents\ChemStation\1\Data\SUN\SUN 2022-12-16 16-59-59
                  \AY3-10-20.M
Last changed    : 15/8/2022 10:25:04 pm by SYSTEM
Analysis Method : C:\Users\Public\Documents\ChemStation\1\Data\SUN\SUN 2022-12-16 16-59-59
                  \AY3-10-20.M (Sequence Method)
Last changed    : 30/1/2023 4:01:21 pm by SYSTEM
                  (modified after loading)
Additional Info : Peak(s) manually integrated
```

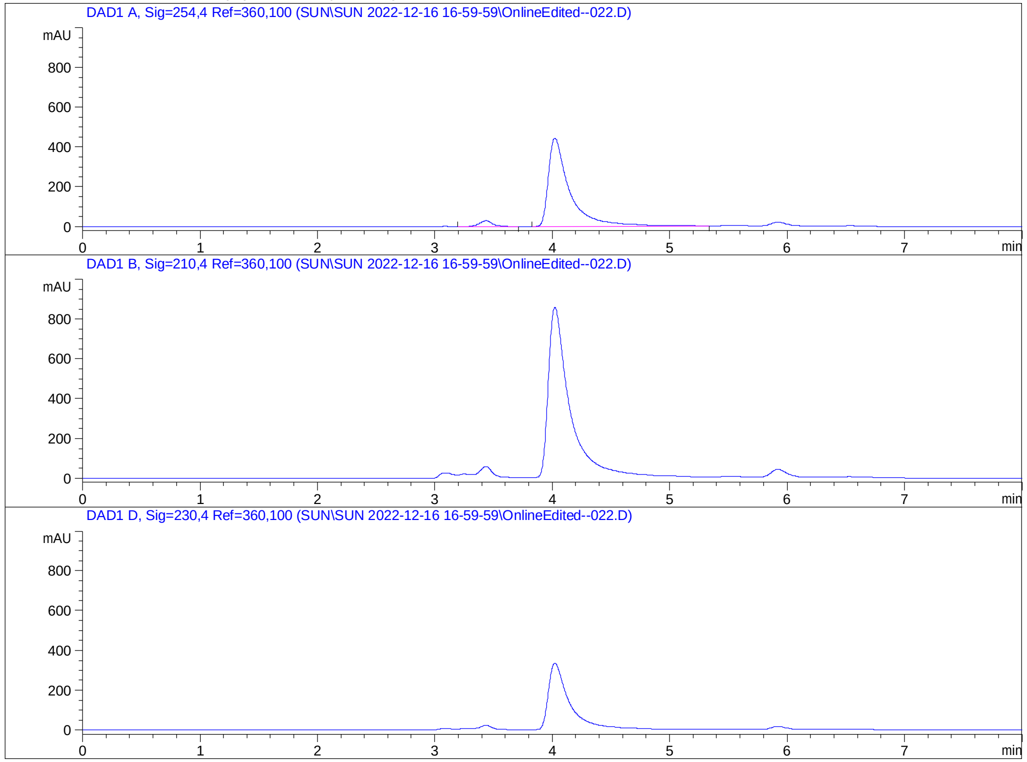

Data File C:\Users\P...\ChemStation\1\Data\SUN\SUN 2022-12-16 16-59-59\OnlineEdited--022.D
Sample Name: zmh-2-4d-chiral

```
=====================================================================
                         Area Percent Report
=====================================================================

Sorted By             :      Signal
Multiplier            :      1.0000
Dilution              :      1.0000
Use Multiplier & Dilution Factor with ISTDs


Signal 1: DAD1 A, Sig=254,4 Ref=360,100
```

Peak #	RetTime [min]	Type	Width [min]	Area [mAU*s]	Height [mAU]	Area %
1	3.437	VB R	0.1144	218.13112	28.79519	3.7623
2	4.023	BB	0.1804	5579.71387	443.09842	96.2377

```
Totals :                  5797.84499  471.89361



Signal 2: DAD1 B, Sig=210,4 Ref=360,100


Signal 3: DAD1 D, Sig=230,4 Ref=360,100

=====================================================================
                          *** End of Report ***
```

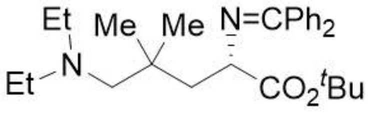

6i
enantioenriched

===

Acq. Operator : SYSTEM Seq. Line : 30
Sample Operator : SYSTEM
Acq. Instrument : HPLC Location : P2-B-11
Injection Date : 12/12/2022 7:06:49 pm Inj : 1
Inj Volume : 2.000 µl
Different Inj Volume from Sample Entry! Actual Inj Volume : 5.000 µl
Acq. Method : C:\Users\Public\Documents\ChemStation\1\Data\SUN\SUN 2022-12-12 10-36-15\IE3-10-20.M
Last changed : 15/8/2022 10:27:27 pm by SYSTEM
Analysis Method : C:\Users\Public\Documents\ChemStation\1\Data\SUN\SUN 2022-12-12 10-36-15\IE3-10-20.M (Sequence Method)
Last changed : 30/1/2023 3:14:49 pm by SYSTEM
(modified after loading)
Additional Info : Peak(s) manually integrated

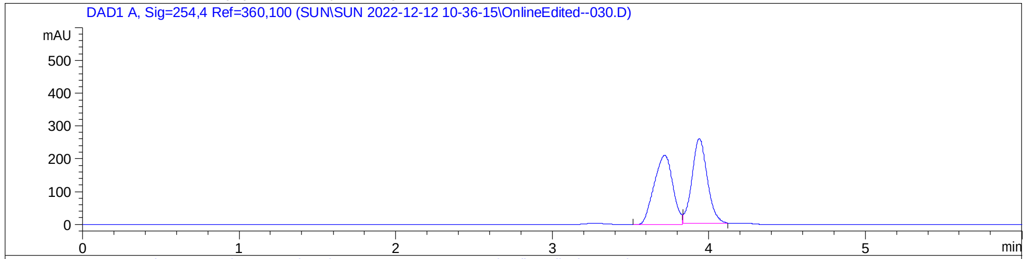

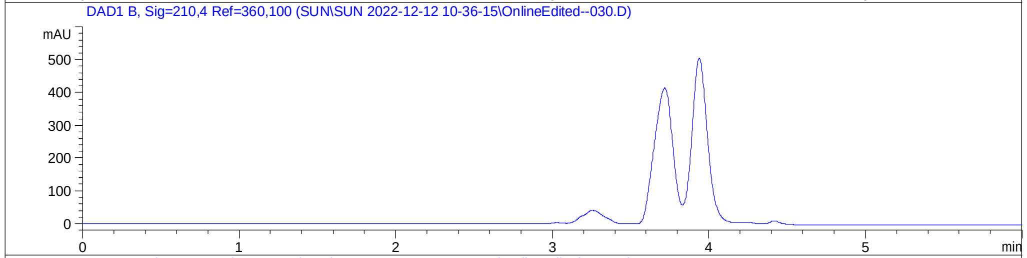

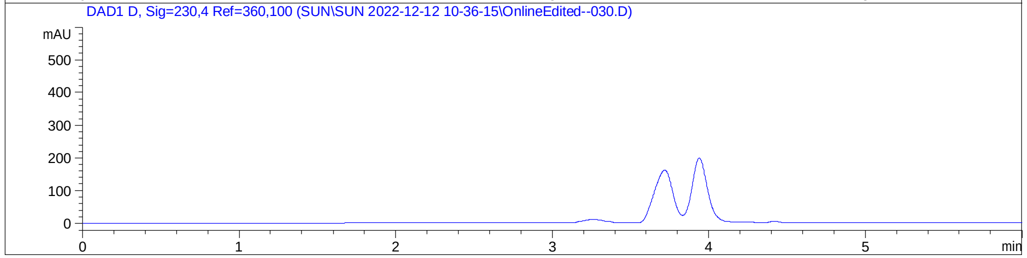

Data File C:\Users\P...\ChemStation\1\Data\SUN\SUN 2022-12-12 10-36-15\OnlineEdited--030.D
Sample Name: zmh-2-67c-rac-ie

===

Area Percent Report

===

Sorted By : Signal
Multiplier : 1.0000
Dilution : 1.0000
Use Multiplier & Dilution Factor with ISTDs

Signal 1: DAD1 A, Sig=254,4 Ref=360,100

Peak #	RetTime [min]	Type	Width [min]	Area [mAU*s]	Height [mAU]	Area %
1	3.718	VV	0.1344	1755.26257	211.42949	50.1998
2	3.940	MM	0.1128	1741.28821	257.26193	49.8002
Totals :				3496.55078	468.69142	

Signal 2: DAD1 B, Sig=210,4 Ref=360,100

Signal 3: DAD1 D, Sig=230,4 Ref=360,100

===

*** End of Report ***

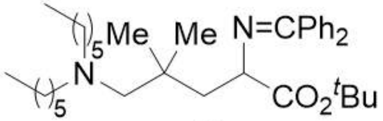

6j
racemic

===

Acq. Operator : SYSTEM　　Seq. Line : 41
Sample Operator : SYSTEM
Acq. Instrument : HPLC　　Location : P2-B-11
Injection Date : 13/12/2022 12:02:50 am　　Inj : 1
Inj Volume : 2.000 µl
Different Inj Volume from Sample Entry! Actual Inj Volume : 5.000 µl
Acq. Method : C:\Users\Public\Documents\ChemStation\1\Data\SUN\SUN 2022-12-12 10-36-15 \IE3-10-20.M
Last changed : 15/8/2022 10:27:27 pm by SYSTEM
Analysis Method : C:\Users\Public\Documents\ChemStation\1\Data\SUN\SUN 2022-12-12 10-36-15 \IE3-10-20.M (Sequence Method)
Last changed : 30/1/2023 3:48:00 pm by SYSTEM (modified after loading)
Additional Info : Peak(s) manually integrated

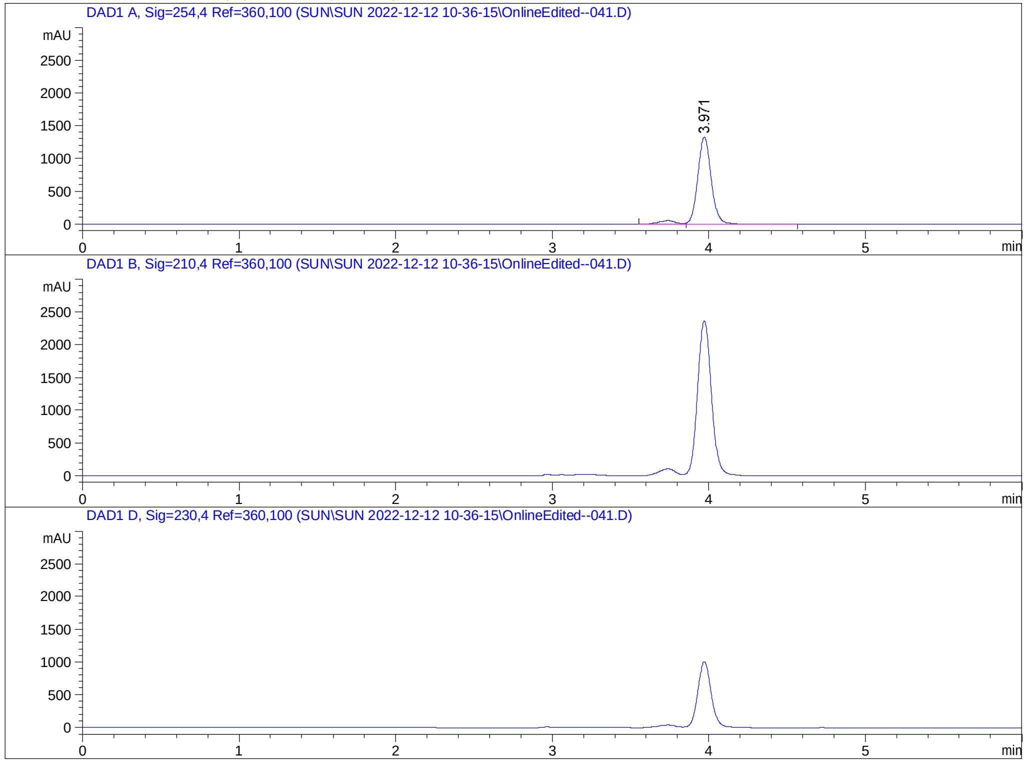

===

Area Percent Report

===

Sorted By : Signal
Multiplier : 1.0000
Dilution : 1.0000
Use Multiplier & Dilution Factor with ISTDs

Signal 1: DAD1 A, Sig=254,4 Ref=360,100

Peak #	RetTime [min]	Type	Width [min]	Area [mAU*s]	Height [mAU]	Area %
1	3.740	BV E	0.1142	376.29568	52.11237	4.5747
2	3.971	VB R	0.0916	7849.33008	1326.03027	95.4253

Totals : 8225.62576 1378.14265

Signal 2: DAD1 B, Sig=210,4 Ref=360,100

Signal 3: DAD1 D, Sig=230,4 Ref=360,100

===

*** End of Report ***

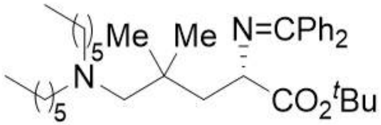

6j
enantioenriched

Data File C:\Users\P...Documents\ChemStation\1\Data\SUN\SUN 2022-11-19 15-57-14\2BD-0401.D
Sample Name: zmh-2-28b-rac

===

Acq. Operator : SYSTEM Seq. Line : 4
Sample Operator : SYSTEM
Acq. Instrument : HPLC Location : P2-B-04
Injection Date : 19/11/2022 4:41:18 pm Inj : 1
Inj Volume : 2.000 µl
Different Inj Volume from Sample Entry! Actual Inj Volume : 20.000 µl
Acq. Method : C:\Users\Public\Documents\ChemStation\1\Data\SUN\SUN 2022-11-19 15-57-14 \AY3-10-20.M
Last changed : 15/8/2022 10:25:04 pm by SYSTEM
Analysis Method : C:\Users\Public\Documents\ChemStation\1\Data\SUN\SUN 2022-11-19 15-57-14 \AY3-10-20.M (Sequence Method)
Last changed : 26/1/2023 3:42:25 pm by SYSTEM
(modified after loading)
Additional Info : Peak(s) manually integrated

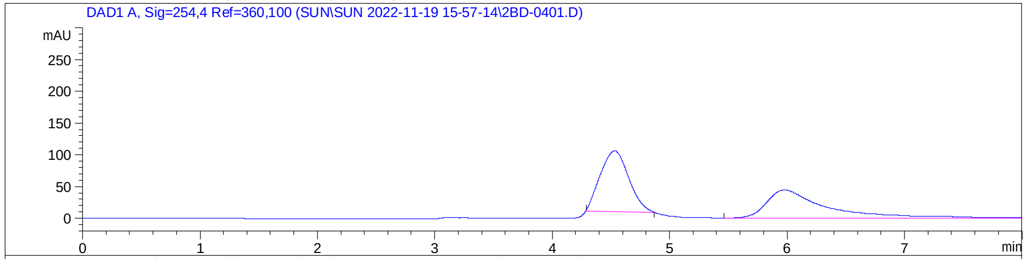

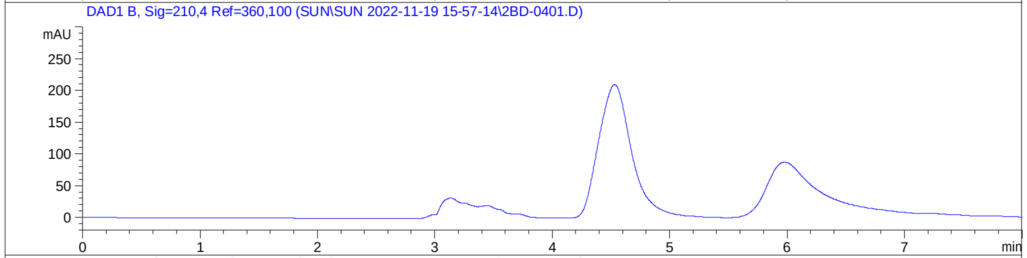

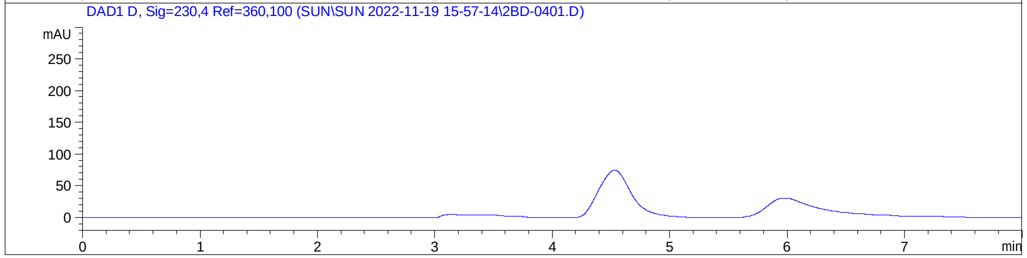

Data File C:\Users\P...Documents\ChemStation\1\Data\SUN\SUN 2022-11-19 15-57-14\2BD-0401.D
Sample Name: zmh-2-28b-rac

===

Area Percent Report

===

Sorted By : Signal
Multiplier : 1.0000
Dilution : 1.0000
Use Multiplier & Dilution Factor with ISTDs

Signal 1: DAD1 A, Sig=254,4 Ref=360,100

Peak #	RetTime [min]	Type	Width [min]	Area [mAU*s]	Height [mAU]	Area %
1	4.533	MM	0.2820	1620.83838	95.79667	50.2316
2	5.979	BB	0.4995	1605.89417	44.60292	49.7684

Totals : 3226.73254 140.39959

Signal 2: DAD1 B, Sig=210,4 Ref=360,100

Signal 3: DAD1 D, Sig=230,4 Ref=360,100

===

*** End of Report ***

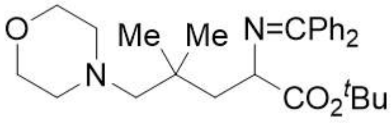

6k
racemic

===
Acq. Operator : SYSTEM Seq. Line : 2
Sample Operator : SYSTEM
Acq. Instrument : HPLC Location : P2-A-11
Injection Date : 19/11/2022 7:02:05 pm Inj : 1
Inj Volume : 2.000 µl
Different Inj Volume from Sample Entry! Actual Inj Volume : 10.000 µl
Acq. Method : C:\Users\Public\Documents\ChemStation\1\Data\SUN\SUN 2022-11-19 18-49-23 \AY3-10-20.M
Last changed : 15/8/2022 10:25:04 pm by SYSTEM
Analysis Method : C:\Users\Public\Documents\ChemStation\1\Data\SUN\SUN 2022-11-19 18-49-23 \AY3-10-20.M (Sequence Method)
Last changed : 26/1/2023 3:45:55 pm by SYSTEM
(modified after loading)
Additional Info : Peak(s) manually integrated

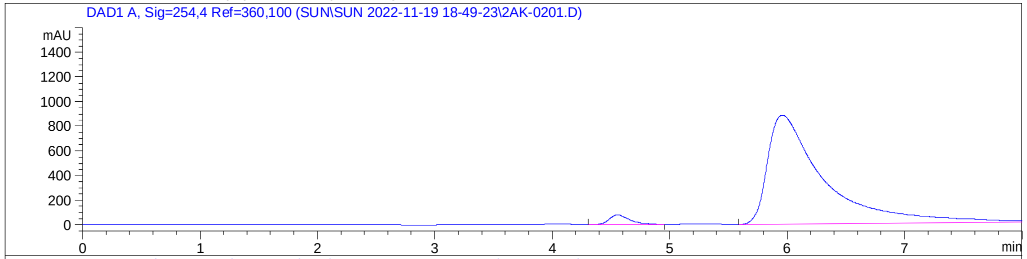

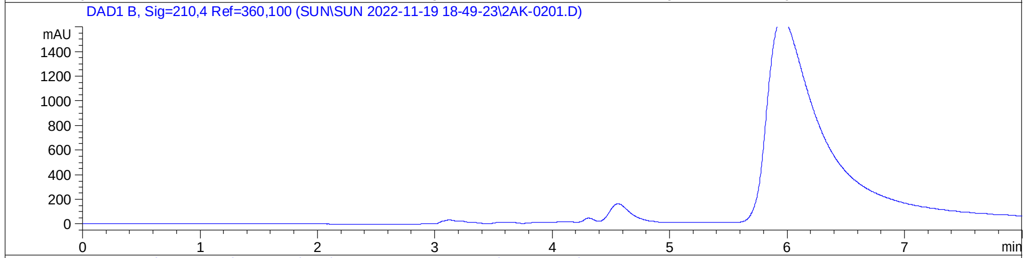

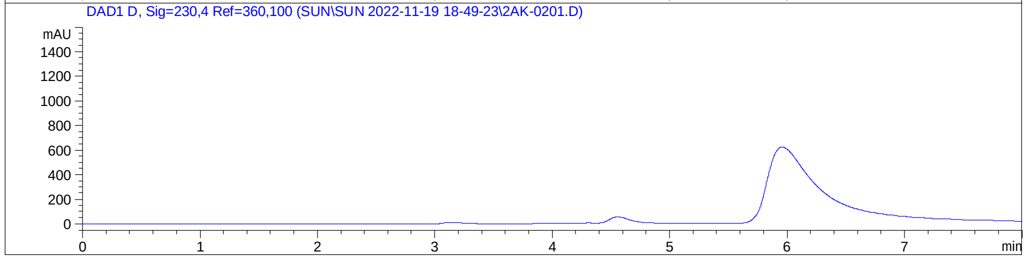

Data File C:\Users\P...Documents\ChemStation\1\Data\SUN\SUN 2022-11-19 18-49-23\2AK-0201.D
Sample Name: zmh-2-4g-chiral

===
Area Percent Report
===

Sorted By : Signal
Multiplier : 1.0000
Dilution : 1.0000
Use Multiplier & Dilution Factor with ISTDs

Signal 1: DAD1 A, Sig=254,4 Ref=360,100

Peak #	RetTime [min]	Type	Width [min]	Area [mAU*s]	Height [mAU]	Area %
1	4.556	BB	0.1824	895.53204	74.15740	2.9279
2	5.959	BB	0.4742	2.96902e4	883.29089	97.0721

Totals : 3.05857e4 957.44830

Signal 2: DAD1 B, Sig=210,4 Ref=360,100

Signal 3: DAD1 D, Sig=230,4 Ref=360,100

===
*** End of Report ***

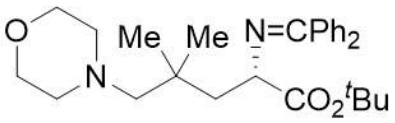

6k
enantioenriched

===

Acq. Operator : SYSTEM Seq. Line : 6
Sample Operator : SYSTEM
Acq. Instrument : HPLC Location : P2-A-11
Injection Date : 9/12/2022 9:43:57 pm Inj : 1
Inj Volume : 2.000 µl
Different Inj Volume from Sample Entry! Actual Inj Volume : 10.000 µl
Acq. Method : C:\Users\Public\Documents\ChemStation\1\Data\SUN\SUN 2022-12-09 19-39-15\AY3-10-20.M
Last changed : 15/8/2022 10:25:04 pm by SYSTEM
Analysis Method : C:\Users\Public\Documents\ChemStation\1\Data\SUN\SUN 2022-12-09 19-39-15\AY3-10-20.M (Sequence Method)
Last changed : 26/1/2023 4:30:42 pm by SYSTEM
(modified after loading)
Additional Info : Peak(s) manually integrated

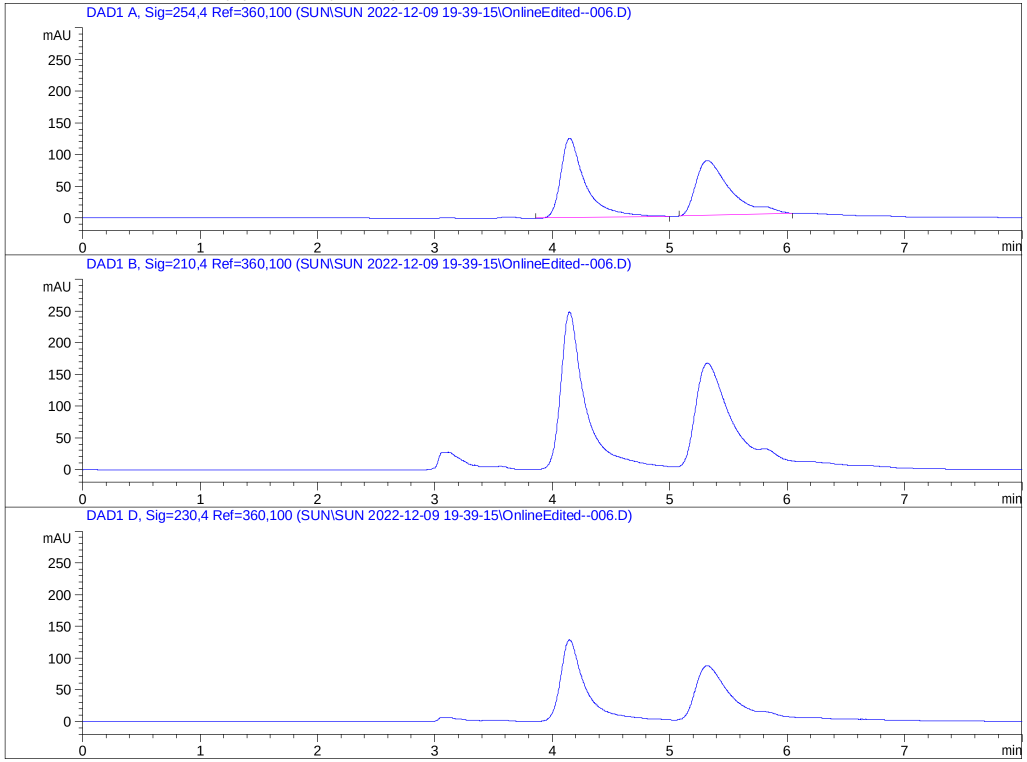

Data File C:\Users\P...\ChemStation\1\Data\SUN\SUN 2022-12-09 19-39-15\OnlineEdited--006.D
Sample Name: zmh-2-37c-rac

===
Area Percent Report
===

Sorted By : Signal
Multiplier : 1.0000
Dilution : 1.0000
Use Multiplier & Dilution Factor with ISTDs

Signal 1: DAD1 A, Sig=254,4 Ref=360,100

Peak #	RetTime [min]	Type	Width [min]	Area [mAU*s]	Height [mAU]	Area %
1	4.148	MM R	0.2434	1824.52576	124.94902	50.6997
2	5.322	PM R	0.3402	1774.16760	86.92776	49.3003

Totals : 3598.69336 211.87678

Signal 2: DAD1 B, Sig=210,4 Ref=360,100

Signal 3: DAD1 D, Sig=230,4 Ref=360,100

===
*** End of Report ***

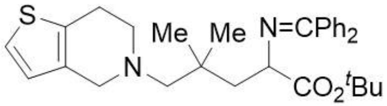

6l
racemic

===

Acq. Operator : SYSTEM   Seq. Line : 2
Sample Operator : SYSTEM
Acq. Instrument : HPLC   Location : P2-A-10
Injection Date : 10/12/2022 8:57:06 am   Inj : 1
Inj Volume : 2.000 µl
Different Inj Volume from Sample Entry! Actual Inj Volume : 10.000 µl
Acq. Method : C:\Users\Public\Documents\ChemStation\1\Data\SUN\SUN 2022-12-10 08-44-26\AY3-10-20.M
Last changed : 15/8/2022 10:25:04 pm by SYSTEM
Analysis Method : C:\Users\Public\Documents\ChemStation\1\Data\SUN\SUN 2022-12-10 08-44-26\AY3-10-20.M (Sequence Method)
Last changed : 26/1/2023 4:32:39 pm by SYSTEM
(modified after loading)
Additional Info : Peak(s) manually integrated

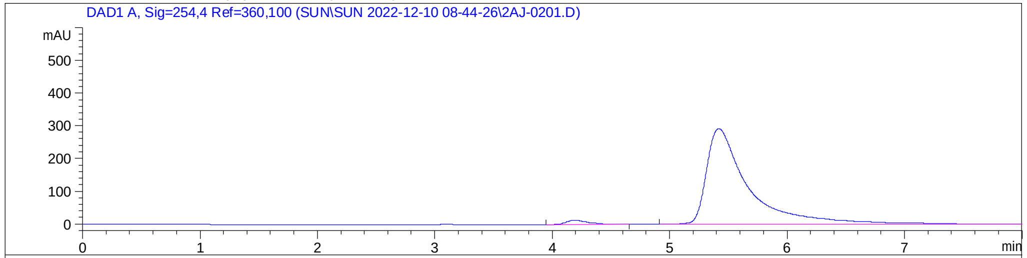

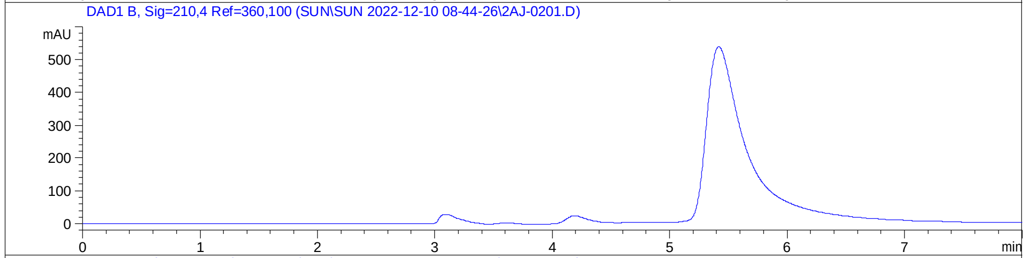

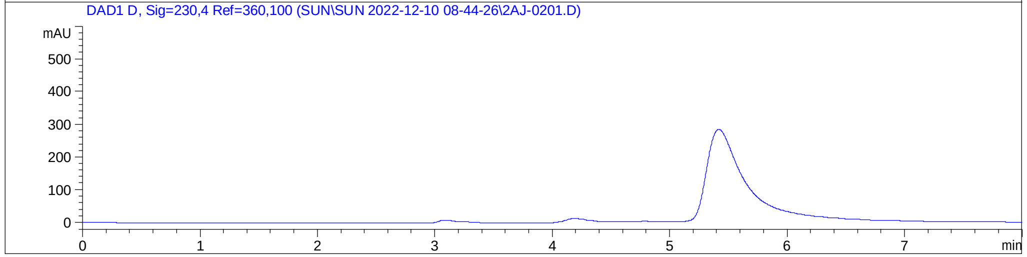

Data File C:\Users\P...Documents\ChemStation\1\Data\SUN\SUN 2022-12-10 08-44-26\2AJ-0201.D
Sample Name: zmh-2-53d-chiral

===

Area Percent Report

===

Sorted By : Signal
Multiplier : 1.0000
Dilution : 1.0000
Use Multiplier & Dilution Factor with ISTDs

Signal 1: DAD1 A, Sig=254,4 Ref=360,100

Peak #	RetTime [min]	Type	Width [min]	Area [mAU*s]	Height [mAU]	Area %
1	4.192	BB	0.2021	182.80055	13.32250	2.4208
2	5.418	BB	0.3584	7368.41602	292.03226	97.5792

Totals : 7551.21657 305.35475

Signal 2: DAD1 B, Sig=210,4 Ref=360,100

Signal 3: DAD1 D, Sig=230,4 Ref=360,100

===

*** End of Report ***

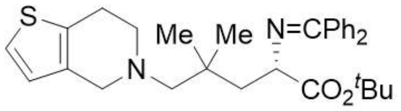

6l
enantioenriched

===

Acq. Operator : SYSTEM Seq. Line : 27
Sample Operator : SYSTEM
Acq. Instrument : HPLC Location : P1-D-11
Injection Date : 10/2/2023 4:43:03 am Inj : 1
Inj Volume : 2.000 µl
Different Inj Volume from Sample Entry! Actual Inj Volume : 10.000 µl
Acq. Method : C:\Users\Public\Documents\ChemStation\1\Data\SUN\SUN 2023-02-09 17-24-43\IC3-10-20.M
Last changed : 15/8/2022 10:26:28 pm by SYSTEM
Analysis Method : C:\Users\Public\Documents\ChemStation\1\Data\SUN\SUN 2023-02-09 17-24-43\IC3-10-20.M (Sequence Method)
Last changed : 5/9/2023 7:35:15 pm by SYSTEM
(modified after loading)
Additional Info : Peak(s) manually integrated

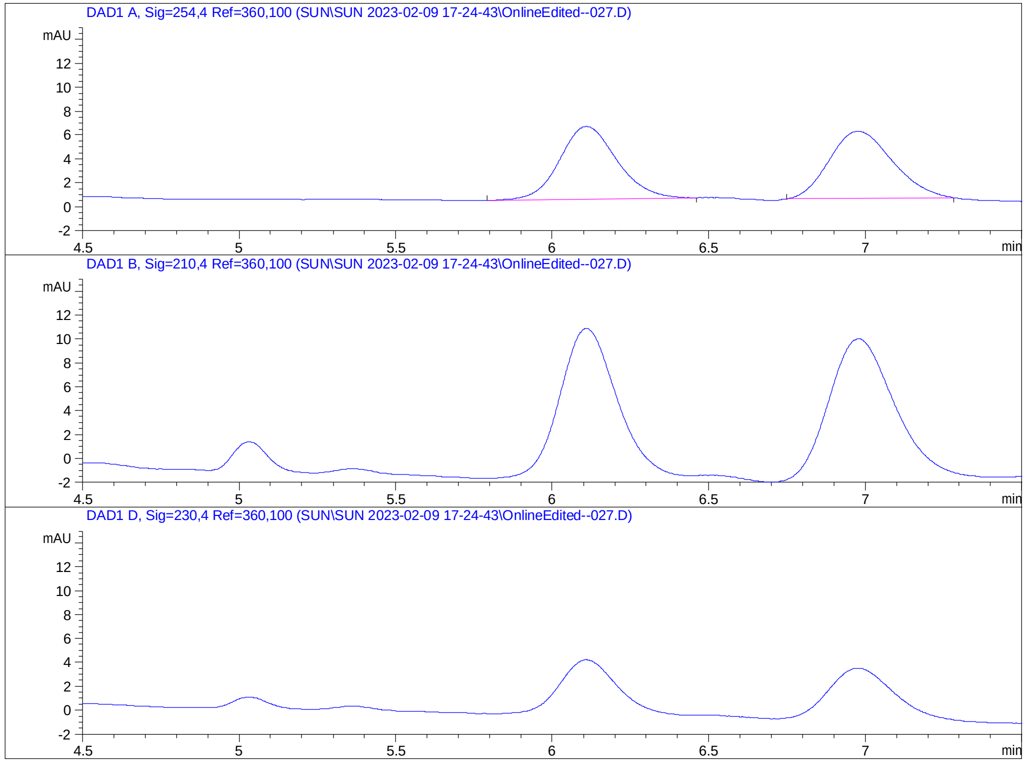

Data File C:\Users\P...\ChemStation\1\Data\SUN\SUN 2023-02-09 17-24-43\OnlineEdited--027.D
Sample Name: zmh-2-67d-ic

===

Area Percent Report

===

Sorted By : Signal
Multiplier : 1.0000
Dilution : 1.0000
Use Multiplier & Dilution Factor with ISTDs

Signal 1: DAD1 A, Sig=254,4 Ref=360,100

Peak #	RetTime [min]	Type	Width [min]	Area [mAU*s]	Height [mAU]	Area %
1	6.109	BB	0.1849	77.19479	6.08984	49.4549
2	6.978	MM	0.2339	78.89661	5.62110	50.5451

Totals : 156.09141 11.71094

Signal 2: DAD1 B, Sig=210,4 Ref=360,100

Signal 3: DAD1 D, Sig=230,4 Ref=360,100

===

*** End of Report ***

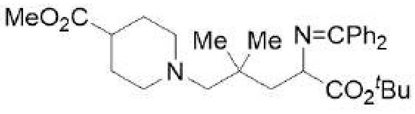

6m
racemic

===

Acq. Operator : SYSTEM Seq. Line : 51
Sample Operator : SYSTEM
Acq. Instrument : HPLC Location : P1-D-10
Injection Date : 10/2/2023 3:28:00 pm Inj : 1
Inj Volume : 2.000 µl
Different Inj Volume from Sample Entry! Actual Inj Volume : 10.000 µl
Acq. Method : C:\Users\Public\Documents\ChemStation\1\Data\SUN\SUN 2023-02-09 17-24-43 \IC3-10-20.M
Last changed : 15/8/2022 10:26:28 pm by SYSTEM
Analysis Method : C:\Users\Public\Documents\ChemStation\1\Data\SUN\SUN 2023-02-09 17-24-43 \IC3-10-20.M (Sequence Method)
Last changed : 5/9/2023 7:39:12 pm by SYSTEM
(modified after loading)
Additional Info : Peak(s) manually integrated

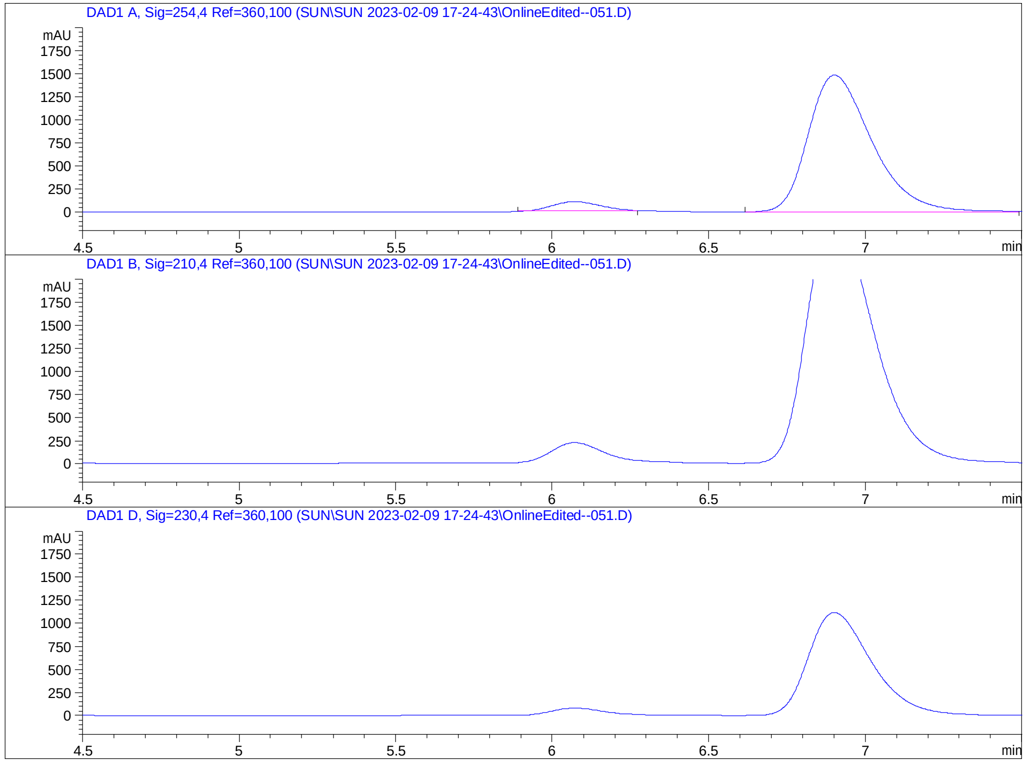

Data File C:\Users\P...\ChemStation\1\Data\SUN\SUN 2023-02-09 17-24-43\OnlineEdited--051.D
Sample Name: zmh-2-67d-chiral

===

Area Percent Report

===

Sorted By : Signal
Multiplier : 1.0000
Dilution : 1.0000
Use Multiplier & Dilution Factor with ISTDs

Signal 1: DAD1 A, Sig=254,4 Ref=360,100

Peak #	RetTime [min]	Type	Width [min]	Area [mAU*s]	Height [mAU]	Area %
1	6.071	MM R	0.1812	1112.48645	102.32578	4.9932
2	6.901	MM R	0.2376	2.11673e4	1484.82397	95.0068

Totals : 2.22798e4 1587.14975

Signal 2: DAD1 B, Sig=210,4 Ref=360,100

Signal 3: DAD1 D, Sig=230,4 Ref=360,100

===

*** End of Report ***

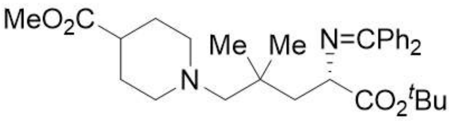

6m
enantioenriched

===

```
Acq. Operator   : SYSTEM                        Seq. Line :   2
Sample Operator : SYSTEM
Acq. Instrument : HPLC                           Location :   P2-A-11
Injection Date  : 20/11/2022 2:00:51 pm               Inj :   1
                                               Inj Volume : 2.000 µl
Different Inj Volume from Sample Entry!  Actual Inj Volume : 20.000 µl
Acq. Method     : C:\Users\Public\Documents\ChemStation\1\Data\SUN\SUN 2022-11-20 13-46-25
                  \AY3-10-20.M
Last changed    : 15/8/2022 10:25:04 pm by SYSTEM
Analysis Method : C:\Users\Public\Documents\ChemStation\1\Data\SUN\SUN 2022-11-20 13-46-25
                  \AY3-10-20.M (Sequence Method)
Last changed    : 26/1/2023 4:00:20 pm by SYSTEM
                  (modified after loading)
Additional Info : Peak(s) manually integrated
```

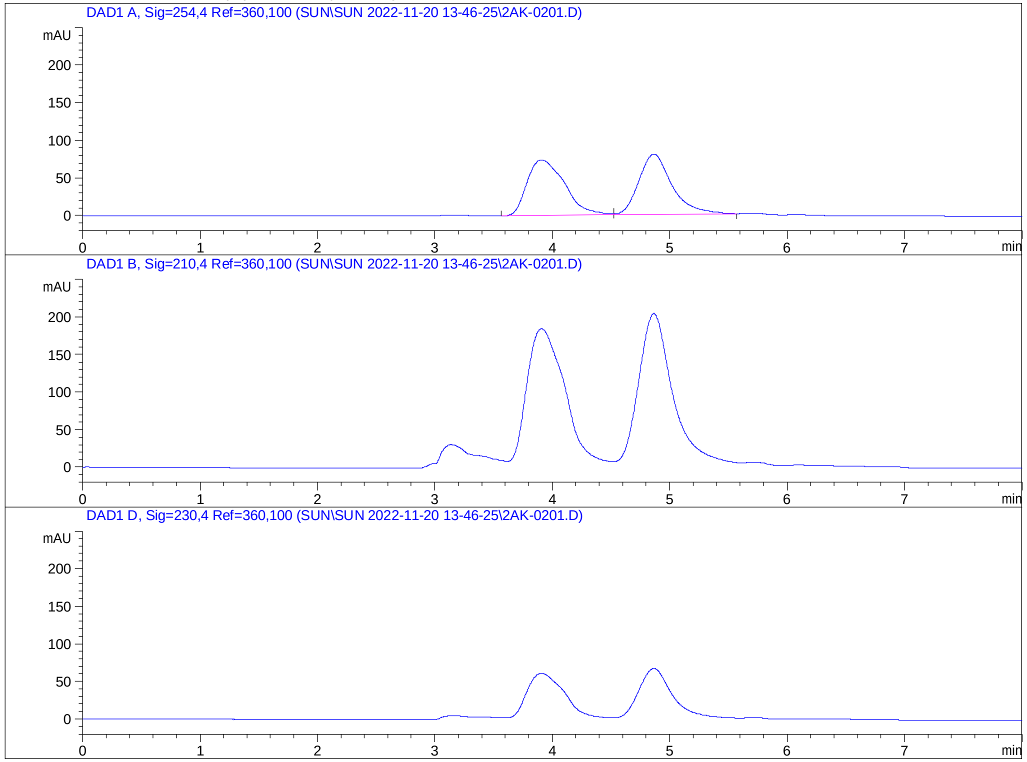

Data File C:\Users\P...Documents\ChemStation\1\Data\SUN\SUN 2022-11-20 13-46-25\2AK-0201.D
Sample Name: zmh-2-37b-rac

===

Area Percent Report

===

```
Sorted By             :      Signal
Multiplier            :      1.0000
Dilution              :      1.0000
Use Multiplier & Dilution Factor with ISTDs
```

Signal 1: DAD1 A, Sig=254,4 Ref=360,100

Peak #	RetTime [min]	Type	Width [min]	Area [mAU*s]	Height [mAU]	Area %
1	3.909	BV	0.3277	1637.58057	73.86097	50.9252
2	4.865	VB	0.2946	1578.07532	80.54662	49.0748

Totals : 3215.65588 154.40759

Signal 2: DAD1 B, Sig=210,4 Ref=360,100

Signal 3: DAD1 D, Sig=230,4 Ref=360,100

===

*** End of Report ***

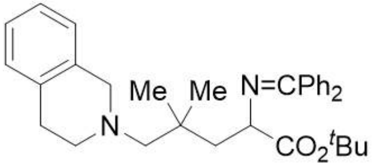

6n
racemic

===

```
Acq. Operator   : SYSTEM                        Seq. Line :   3
Sample Operator : SYSTEM
Acq. Instrument : HPLC                           Location :   P2-A-09
Injection Date  : 20/11/2022 2:21:55 pm              Inj :   1
                                              Inj Volume : 2.000 µl
Different Inj Volume from Sample Entry!  Actual Inj Volume : 10.000 µl
Acq. Method     : C:\Users\Public\Documents\ChemStation\1\Data\SUN\SUN 2022-11-20 13-46-25
                  \AY3-10-20.M
Last changed    : 15/8/2022 10:25:04 pm by SYSTEM
Analysis Method : C:\Users\Public\Documents\ChemStation\1\Data\SUN\SUN 2022-11-20 13-46-25
                  \AY3-10-20.M (Sequence Method)
Last changed    : 26/1/2023 4:09:24 pm by SYSTEM
                  (modified after loading)
Additional Info : Peak(s) manually integrated
```

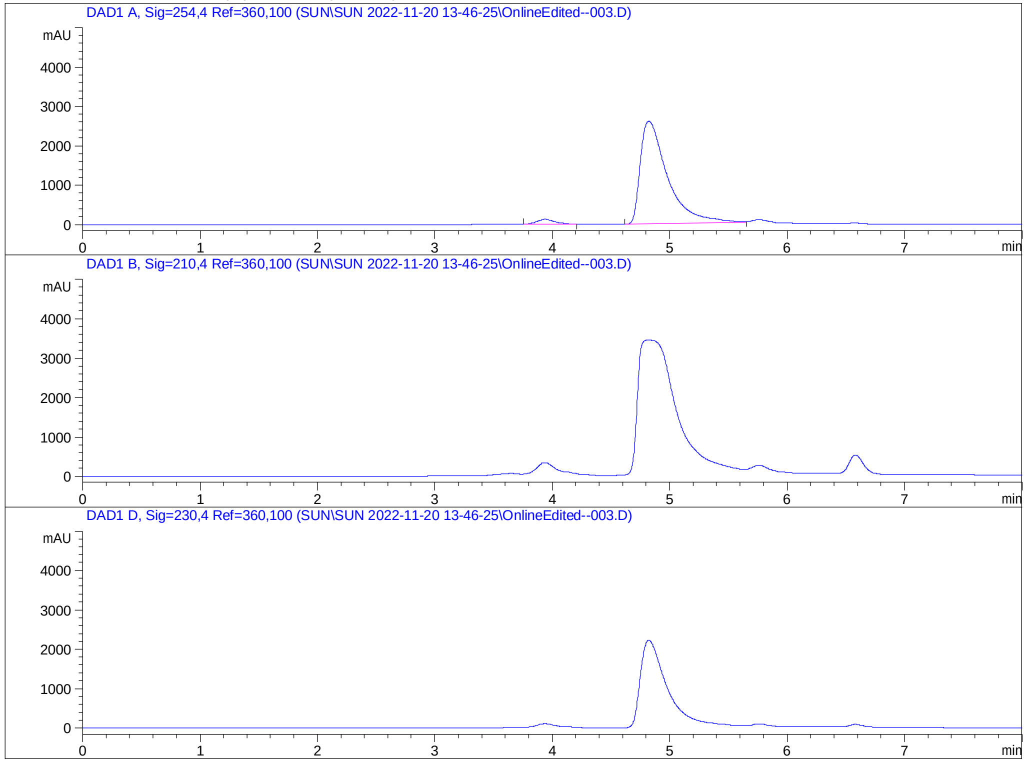

Data File C:\Users\P...\ChemStation\1\Data\SUN\SUN 2022-11-20 13-46-25\OnlineEdited--003.D
Sample Name: zmh-2-21b-chiral

===

Area Percent Report

===

```
Sorted By            :      Signal
Multiplier           :      1.0000
Dilution             :      1.0000
Use Multiplier & Dilution Factor with ISTDs
```

Signal 1: DAD1 A, Sig=254,4 Ref=360,100

Peak #	RetTime [min]	Type	Width [min]	Area [mAU*s]	Height [mAU]	Area %
1	3.938	MM R	0.1828	1323.14954	120.64146	3.0646
2	4.823	MM R	0.2671	4.18519e4	2611.23096	96.9354

Totals : 4.31750e4 2731.87241

Signal 2: DAD1 B, Sig=210,4 Ref=360,100

Signal 3: DAD1 D, Sig=230,4 Ref=360,100

===

*** End of Report ***

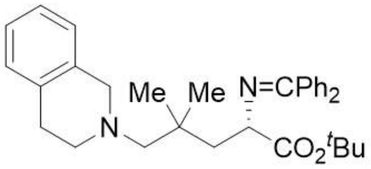

6n
enantioenriched

===

Acq. Operator : SYSTEM   Seq. Line : 8
Sample Operator : SYSTEM
Acq. Instrument : HPLC   Location : P2-A-08
Injection Date : 11/12/2022 10:04:41 pm   Inj : 1
Inj Volume : 2.000 µl
Different Inj Volume from Sample Entry! Actual Inj Volume : 10.000 µl
Acq. Method : C:\Users\Public\Documents\ChemStation\1\Data\SUN\SUN 2022-12-11 20-15-03 \AY3-10-20.M
Last changed : 15/8/2022 10:25:04 pm by SYSTEM
Analysis Method : C:\Users\Public\Documents\ChemStation\1\Data\SUN\SUN 2022-12-11 20-15-03 \AY3-10-20.M (Sequence Method)
Last changed : 26/1/2023 4:39:48 pm by SYSTEM
(modified after loading)
Additional Info : Peak(s) manually integrated

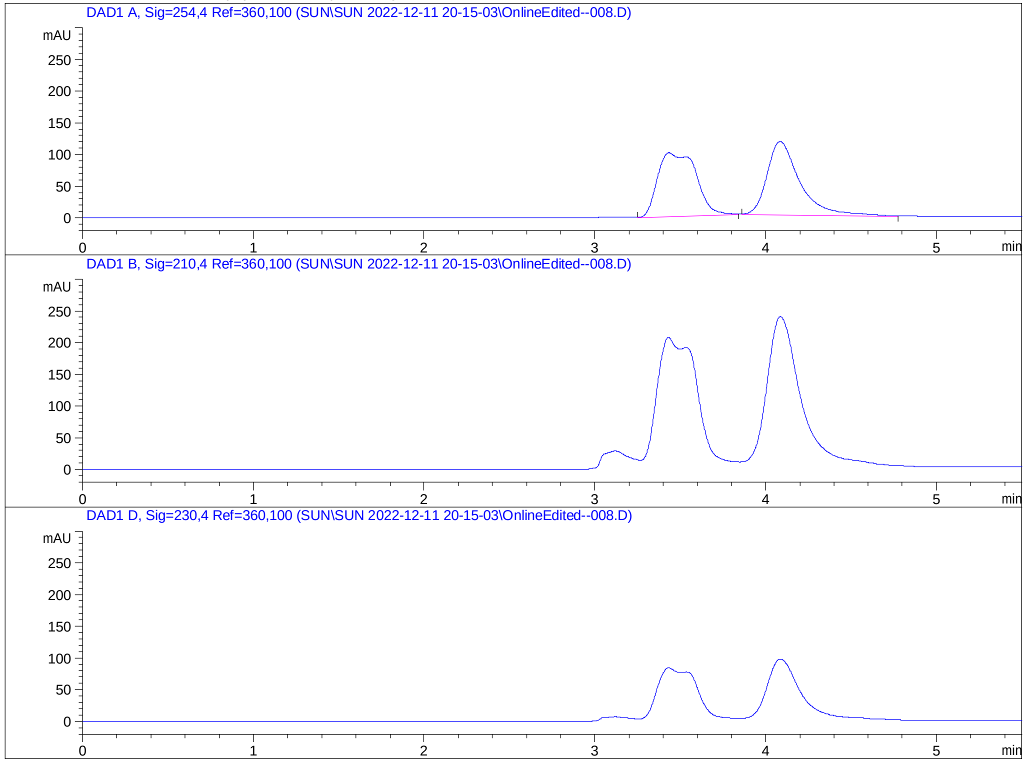

Data File C:\Users\P...\ChemStation\1\Data\SUN\SUN 2022-12-11 20-15-03\OnlineEdited--008.D
Sample Name: zmh-2-67e-rac

===

Area Percent Report

===

Sorted By : Signal
Multiplier : 1.0000
Dilution : 1.0000
Use Multiplier & Dilution Factor with ISTDs

Signal 1: DAD1 A, Sig=254,4 Ref=360,100

Peak #	RetTime [min]	Type	Width [min]	Area [mAU*s]	Height [mAU]	Area %
1	3.432	MM R	0.2601	1582.35632	101.41196	50.4968
2	4.084	MM R	0.2230	1551.22144	115.92220	49.5032

Totals : 3133.57776 217.33415

Signal 2: DAD1 B, Sig=210,4 Ref=360,100

Signal 3: DAD1 D, Sig=230,4 Ref=360,100

===

*** End of Report ***

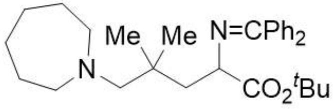

6o
racemic

===

Acq. Operator : SYSTEM Seq. Line : 141
Sample Operator : SYSTEM
Acq. Instrument : HPLC Location : P1-F-01
Injection Date : 19/1/2023 2:02:55 am Inj : 1
Inj Volume : 2.000 µl
Different Inj Volume from Sample Entry! Actual Inj Volume : 5.000 µl
Acq. Method : C:\Users\Public\Documents\ChemStation\1\Data\SUN\SUN 2023-01-17 11-01-44\AY3-10-20.M
Last changed : 15/8/2022 10:25:04 pm by SYSTEM
Analysis Method : C:\Users\Public\Documents\ChemStation\1\Data\SUN\SUN 2023-01-17 11-01-44\AY3-10-20.M (Sequence Method)
Last changed : 5/9/2023 7:46:51 pm by SYSTEM
(modified after loading)
Additional Info : Peak(s) manually integrated

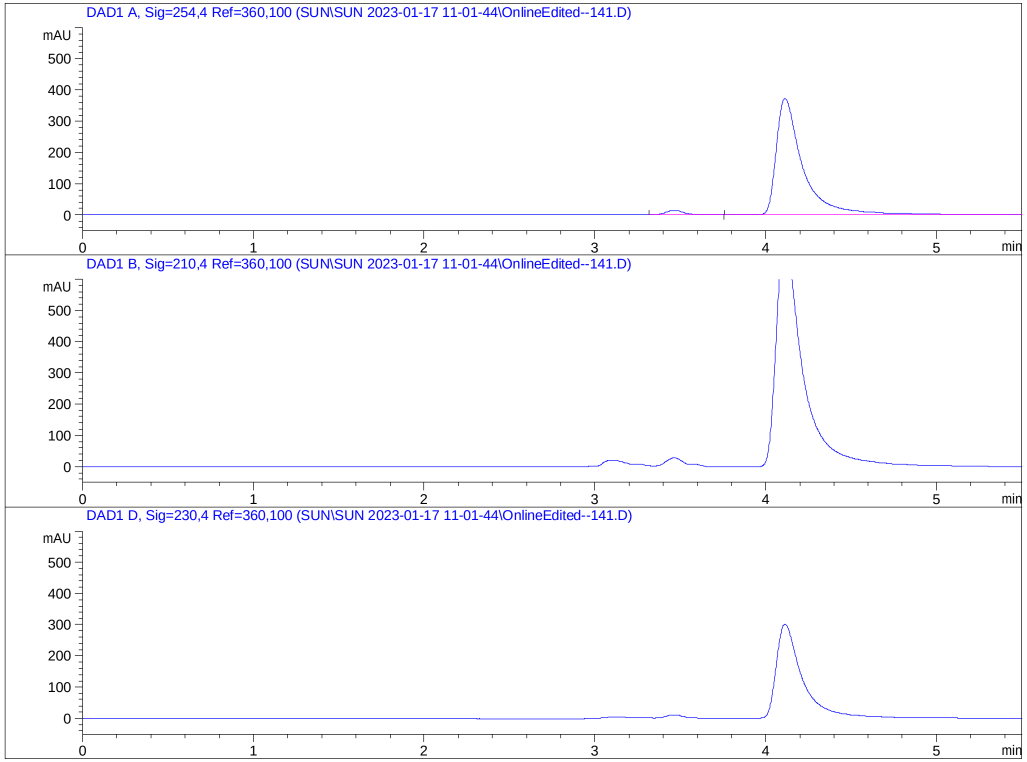

Data File C:\Users\P...\ChemStation\1\Data\SUN\SUN 2023-01-17 11-01-44\OnlineEdited--141.D
Sample Name: zmh-2-76

===
Area Percent Report
===

Sorted By : Signal
Multiplier : 1.0000
Dilution : 1.0000
Use Multiplier & Dilution Factor with ISTDs

Signal 1: DAD1 A, Sig=254,4 Ref=360,100

Peak #	RetTime [min]	Type	Width [min]	Area [mAU*s]	Height [mAU]	Area %
1	3.464	BB	0.1145	105.31068	14.19681	2.5502
2	4.113	BB	0.1564	4024.13721	372.76596	97.4498
Totals :				4129.44789	386.96277	

Signal 2: DAD1 B, Sig=210,4 Ref=360,100

Signal 3: DAD1 D, Sig=230,4 Ref=360,100

===
*** End of Report ***

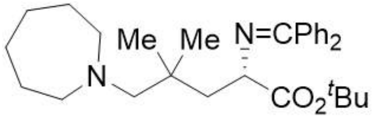

6o
enantioenriched

===

Acq. Operator : SYSTEM Seq. Line : 2
Sample Operator : SYSTEM
Acq. Instrument : HPLC Location : P2-A-07
Injection Date : 12/12/2022 9:04:49 am Inj : 1
Inj Volume : 2.000 µl
Different Inj Volume from Sample Entry! Actual Inj Volume : 5.000 µl
Acq. Method : C:\Users\Public\Documents\ChemStation\1\Data\SUN\SUN 2022-12-12 08-52-11\AY3-10-20.M
Last changed : 15/8/2022 10:25:04 pm by SYSTEM
Analysis Method : C:\Users\Public\Documents\ChemStation\1\Data\SUN\SUN 2022-12-12 08-52-11\AY3-10-20.M (Sequence Method)
Last changed : 26/1/2023 4:46:26 pm by SYSTEM
(modified after loading)
Additional Info : Peak(s) manually integrated

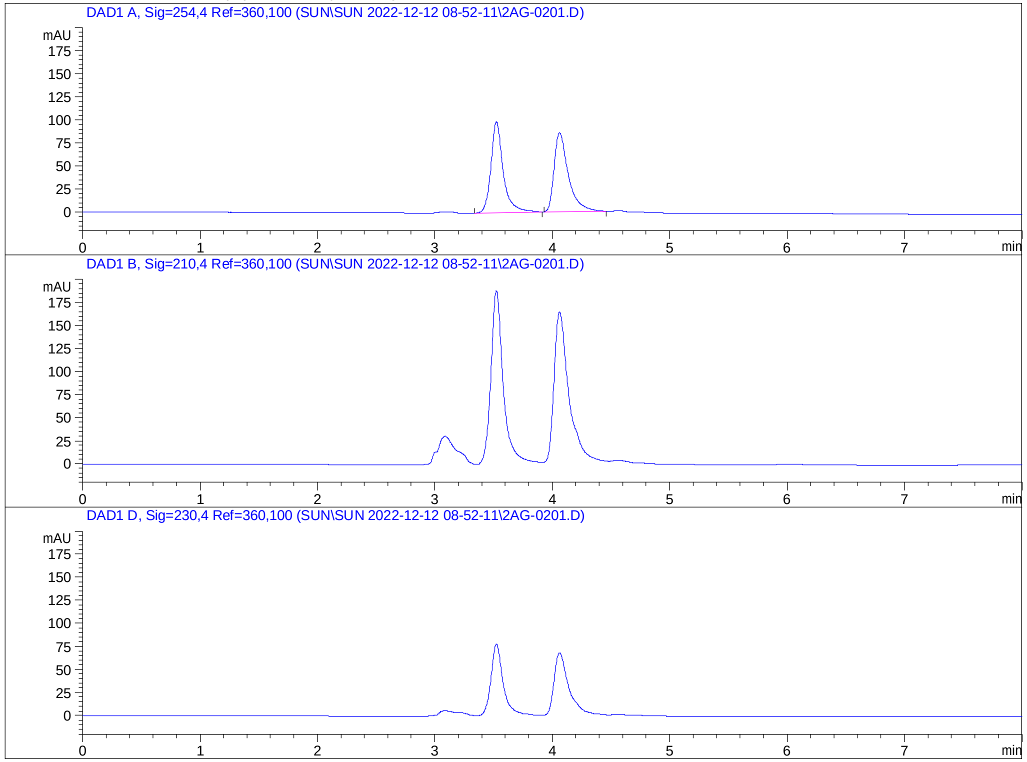

Data File C:\Users\P...Documents\ChemStation\1\Data\SUN\SUN 2022-12-12 08-52-11\2AG-0201.D
Sample Name: zmh-2-67f-rac-

===

Area Percent Report

===

Sorted By : Signal
Multiplier : 1.0000
Dilution : 1.0000
Use Multiplier & Dilution Factor with ISTDs

Signal 1: DAD1 A, Sig=254,4 Ref=360,100

Peak #	RetTime [min]	Type	Width [min]	Area [mAU*s]	Height [mAU]	Area %
1	3.525	MM R	0.1224	723.73267	98.56277	49.8667
2	4.062	MM R	0.1413	727.60327	85.80049	50.1333

Totals : 1451.33594 184.36327

Signal 2: DAD1 B, Sig=210,4 Ref=360,100

Signal 3: DAD1 D, Sig=230,4 Ref=360,100

===

*** End of Report ***

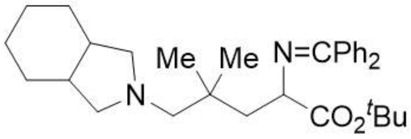

6p
racemic

===

Acq. Operator : SYSTEM Seq. Line : 2
Sample Operator : SYSTEM
Acq. Instrument : HPLC Location : P1-A-03
Injection Date : 13/1/2023 9:24:15 am Inj : 1
Inj Volume : 2.000 µl
Different Inj Volume from Sample Entry! Actual Inj Volume : 5.000 µl
Acq. Method : C:\Users\Public\Documents\ChemStation\1\Data\SUN\SUN 2023-01-13 09-11-35 \AY3-10-20.M
Last changed : 15/8/2022 10:25:04 pm by SYSTEM
Analysis Method : C:\Users\Public\Documents\ChemStation\1\Data\SUN\SUN 2023-01-13 09-11-35 \AY3-10-20.M (Sequence Method)
Last changed : 10/2/2023 4:50:51 pm by SYSTEM
(modified after loading)
Additional Info : Peak(s) manually integrated

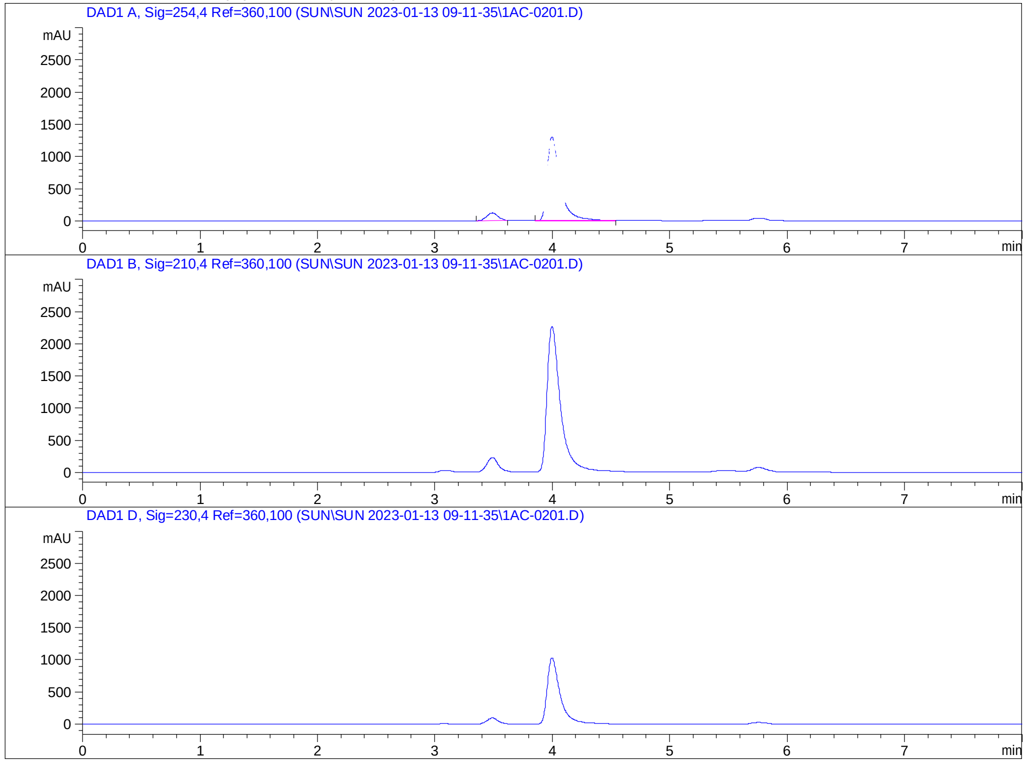

Data File C:\Users\P...Documents\ChemStation\1\Data\SUN\SUN 2023-01-13 09-11-35\1AC-0201.D
Sample Name: zmh-2-98d

===

Area Percent Report

===

Sorted By : Signal
Multiplier : 1.0000
Dilution : 1.0000
Use Multiplier & Dilution Factor with ISTDs

Signal 1: DAD1 A, Sig=254,4 Ref=360,100

Peak #	RetTime [min]	Type	Width [min]	Area [mAU*s]	Height [mAU]	Area %
1	3.491	MM	0.1113	797.40479	119.45081	7.2793
2	3.998	MM	0.1298	1.01570e4	1304.48572	92.7207

Totals : 1.09544e4 1423.93652

Signal 2: DAD1 B, Sig=210,4 Ref=360,100

Signal 3: DAD1 D, Sig=230,4 Ref=360,100

===

*** End of Report ***

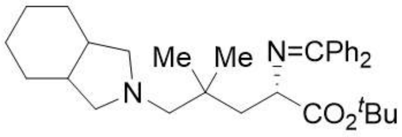

6p
enantioenriched

===

Acq. Operator : SYSTEM Seq. Line : 24
Sample Operator : SYSTEM
Acq. Instrument : HPLC Location : P1-E-10
Injection Date : 10/1/2023 4:22:52 am Inj : 1
Inj Volume : 2.000 µl
Different Inj Volume from Sample Entry! Actual Inj Volume : 5.000 µl
Acq. Method : C:\Users\Public\Documents\ChemStation\1\Data\SUN\SUN 2023-01-09 21-57-28\IE3-10-20.M
Last changed : 15/8/2022 10:27:27 pm by SYSTEM
Analysis Method : C:\Users\Public\Documents\ChemStation\1\Data\SUN\SUN 2023-01-09 21-57-28\IE3-10-20.M (Sequence Method)
Last changed : 30/1/2023 4:30:13 pm by SYSTEM
(modified after loading)
Additional Info : Peak(s) manually integrated

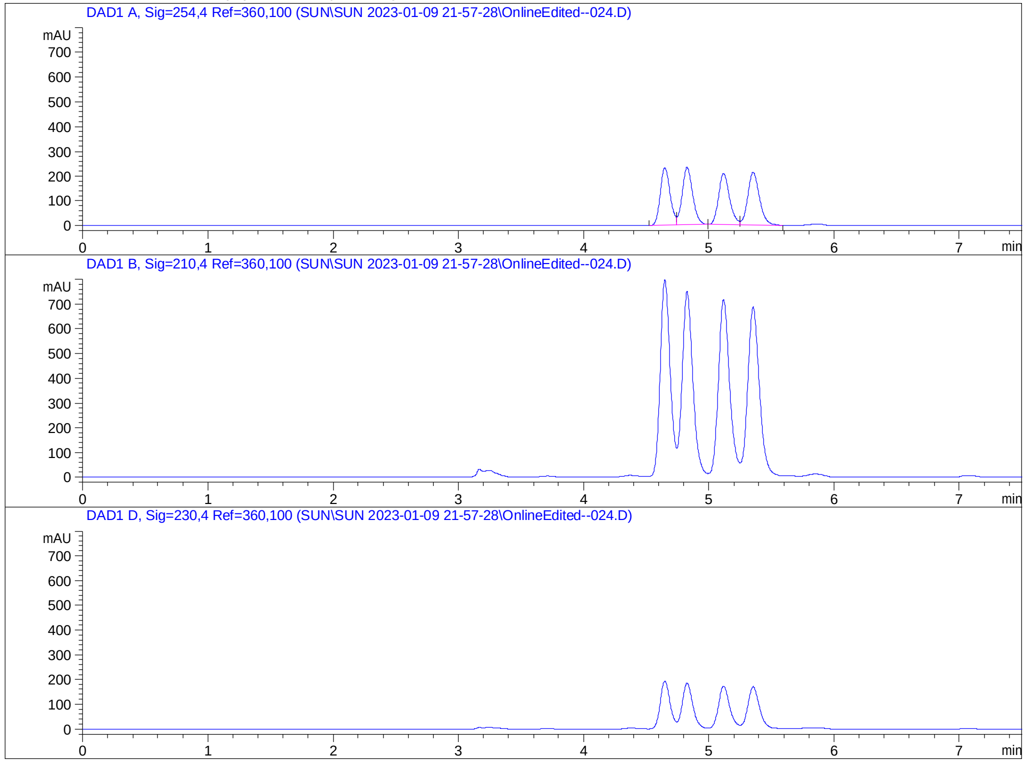

===
Area Percent Report
===

Sorted By : Signal
Multiplier : 1.0000
Dilution : 1.0000
Use Multiplier & Dilution Factor with ISTDs

Signal 1: DAD1 A, Sig=254,4 Ref=360,100

Peak #	RetTime [min]	Type	Width [min]	Area [mAU*s]	Height [mAU]	Area %
1	4.649	BV	0.0843	1270.92139	232.52348	24.1810
2	4.827	VB	0.0887	1348.22717	232.34956	25.6519
3	5.118	BV	0.0938	1259.84998	206.18832	23.9704
4	5.355	VB	0.0988	1376.86462	213.31604	26.1967

Totals : 5255.86316 884.37741

Signal 2: DAD1 B, Sig=210,4 Ref=360,100

Signal 3: DAD1 D, Sig=230,4 Ref=360,100

===
*** End of Report ***

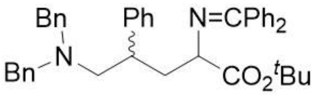

6q
racemic

===

Acq. Operator : SYSTEM   Seq. Line : 49
Sample Operator : SYSTEM
Acq. Instrument : HPLC   Location : P1-C-11
Injection Date : 12/1/2023 7:24:10 pm   Inj : 1
Inj Volume : 2.000 µl
Different Inj Volume from Sample Entry! Actual Inj Volume : 10.000 µl
Acq. Method : C:\Users\Public\Documents\ChemStation\1\Data\SUN\SUN 2023-01-11 22-34-39 \IE3-10-20.M
Last changed : 15/8/2022 10:27:27 pm by SYSTEM
Analysis Method : C:\Users\Public\Documents\ChemStation\1\Data\SUN\SUN 2023-01-11 22-34-39 \IE3-10-20.M (Sequence Method)
Last changed : 30/1/2023 4:33:13 pm by SYSTEM
(modified after loading)
Additional Info : Peak(s) manually integrated

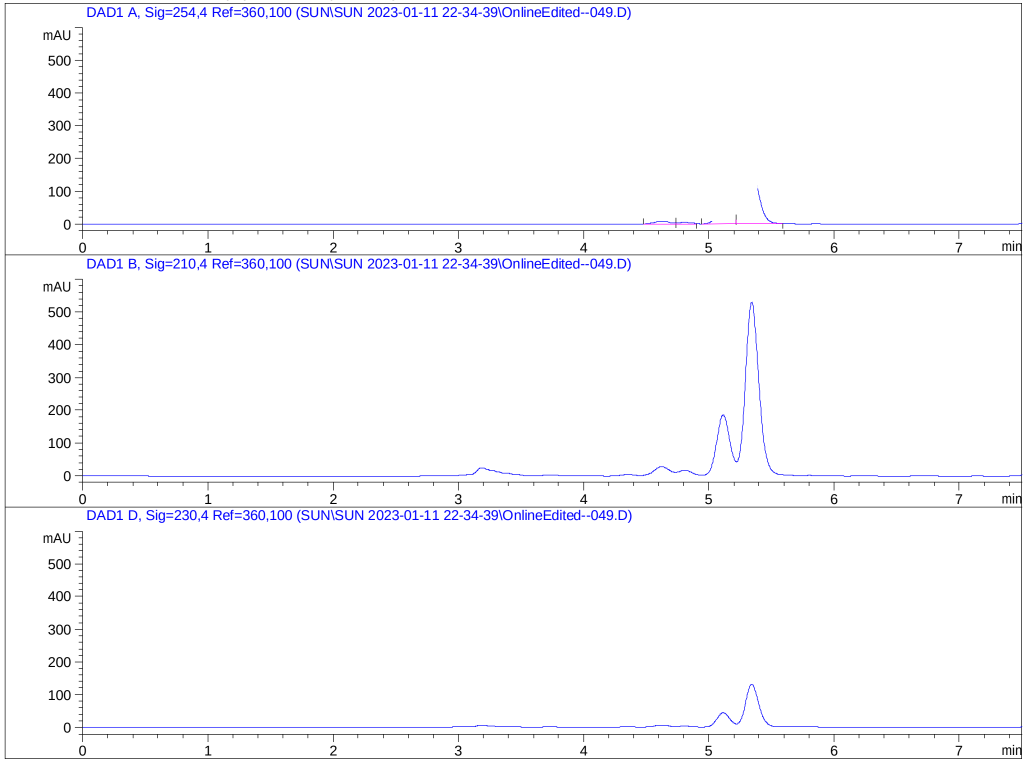

===

Area Percent Report

===

Sorted By : Signal
Multiplier : 1.0000
Dilution : 1.0000
Use Multiplier & Dilution Factor with ISTDs

Signal 1: DAD1 A, Sig=254,4 Ref=360,100

Peak #	RetTime [min]	Type	Width [min]	Area [mAU*s]	Height [mAU]	Area %
1	4.624	MF	0.1508	73.52736	8.12751	4.2139
2	4.809	FM	0.1248	42.25052	5.64250	2.4214
3	5.114	BV	0.1137	389.02011	53.24866	22.2949
4	5.343	VB	0.1155	1240.08313	165.39906	71.0698

Totals : 1744.88112 232.41774

Signal 2: DAD1 B, Sig=210,4 Ref=360,100

Signal 3: DAD1 D, Sig=230,4 Ref=360,100

===

*** End of Report ***

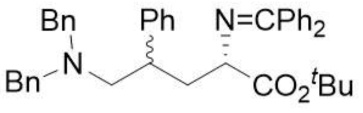

6q
enantioenriched

Data File C:\Users\P...on\1\Data\SUN\SUN 2025-08-13 21-21-01\074-P2-F1-zmh-8-66b-rac-xia.D
Sample Name: zmh-8-66b-rac-xia

===

```
Acq. Operator    : SYSTEM                        Seq. Line :   74
Sample Operator  : SYSTEM
Acq. Instrument  : HPLC                           Location :   P2-F-01
Injection Date   : 15/8/2025 12:10:16 am               Inj :    1
                                                Inj Volume : 2.000 µl
Different Inj Volume from Sample Entry!  Actual Inj Volume : 20.000 µl
Acq. Method      : C:\Users\Public\Documents\ChemStation\1\Data\SUN\SUN 2025-08-13 21-21-01\IC3-
                   10-30.M
Last changed     : 19/8/2022 10:41:24 pm by SYSTEM
Analysis Method  : C:\Users\Public\Documents\ChemStation\1\Data\SUN\SUN 2025-08-13 21-21-01\IC3-
                   10-30.M (Sequence Method)
Last changed     : 15/8/2025 8:37:03 pm by SYSTEM
                   (modified after loading)
Additional Info  : Peak(s) manually integrated
```

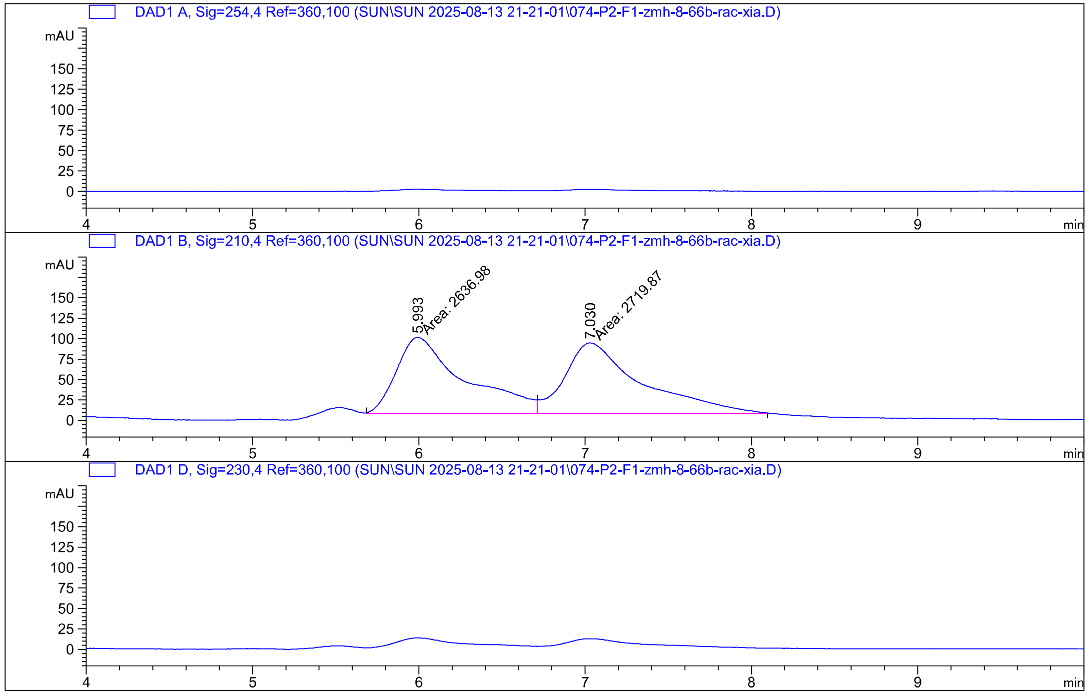

===
Area Percent Report
===

```
Sorted By             :      Signal
Multiplier            :      1.0000
Dilution              :      1.0000
Use Multiplier & Dilution Factor with ISTDs
```


Data File C:\Users\P...on\1\Data\SUN\SUN 2025-08-13 21-21-01\074-P2-F1-zmh-8-66b-rac-xia.D
Sample Name: zmh-8-66b-rac-xia

Signal 1: DAD1 A, Sig=254,4 Ref=360,100

Signal 2: DAD1 B, Sig=210,4 Ref=360,100

Peak #	RetTime [min]	Type	Width [min]	Area [mAU*s]	Height [mAU]	Area %
1	5.993	MF	0.4741	2636.97681	92.70108	49.2262
2	7.030	FM	0.5267	2719.87427	86.05951	50.7738

Totals : 5356.85107 178.76059

Signal 3: DAD1 D, Sig=230,4 Ref=360,100

===
*** End of Report ***

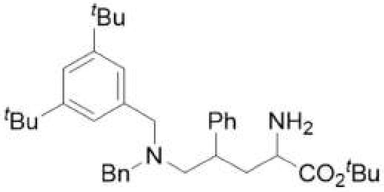

6r'
racemic

===

```
Acq. Operator   : SYSTEM                        Seq. Line :  75
Sample Operator : SYSTEM
Acq. Instrument : HPLC                           Location :   P2-F-02
Injection Date  : 15/8/2025 12:41:41 am               Inj :   1
                                              Inj Volume : 2.000 µl
Different Inj Volume from Sample Entry!  Actual Inj Volume : 20.000 µl
Acq. Method     : C:\Users\Public\Documents\ChemStation\1\Data\SUN\SUN 2025-08-13 21-21-01\IC3-
                  10-30.M
Last changed    : 19/8/2022 10:41:24 pm by SYSTEM
Analysis Method : C:\Users\Public\Documents\ChemStation\1\Data\SUN\SUN 2025-08-13 21-21-01\IC3-
                  10-30.M (Sequence Method)
Last changed    : 15/8/2025 8:41:15 pm by SYSTEM
                  (modified after loading)
Additional Info : Peak(s) manually integrated
```

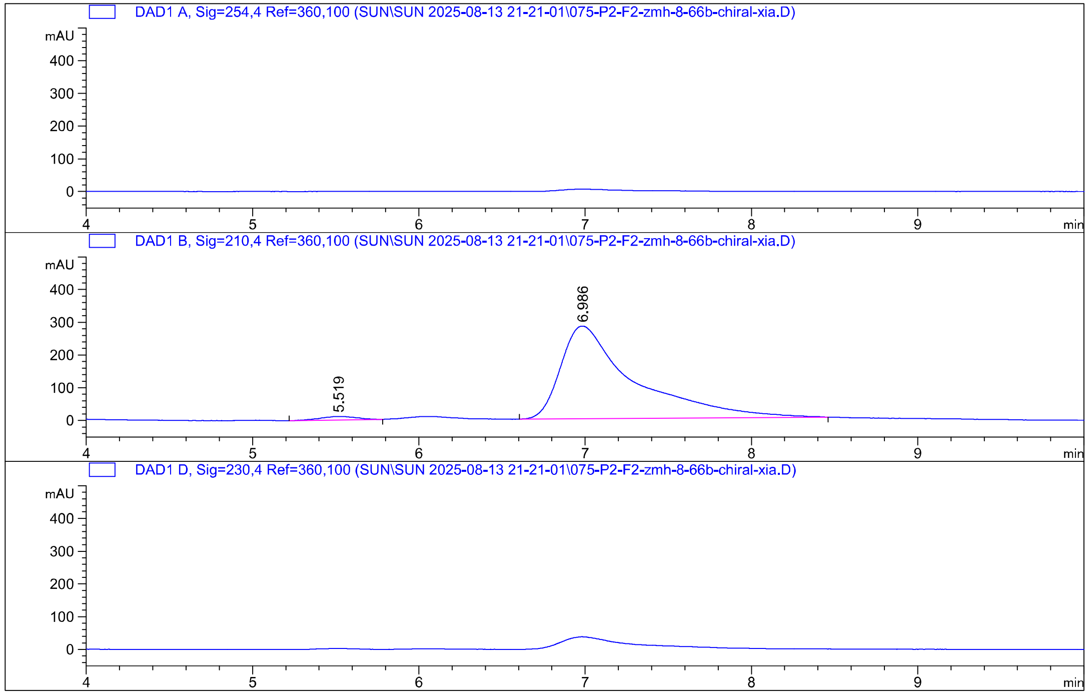

===
Area Percent Report
===

```
Sorted By             :      Signal
Multiplier            :      1.0000
Dilution              :      1.0000
Use Multiplier & Dilution Factor with ISTDs
```


Data File C:\Users\P...1\Data\SUN\SUN 2025-08-13 21-21-01\075-P2-F2-zmh-8-66b-chiral-xia.D
Sample Name: zmh-8-66b-chiral-xia

Signal 1: DAD1 A, Sig=254,4 Ref=360,100

Signal 2: DAD1 B, Sig=210,4 Ref=360,100

Peak #	RetTime [min]	Type	Width [min]	Area [mAU*s]	Height [mAU]	Area %
1	5.519	VV R	0.1789	159.80785	10.54157	1.8168
2	6.986	BV R	0.3693	8636.17188	283.20953	98.1832
Totals :				8795.97972	293.75110	

Signal 3: DAD1 D, Sig=230,4 Ref=360,100

===
*** End of Report ***

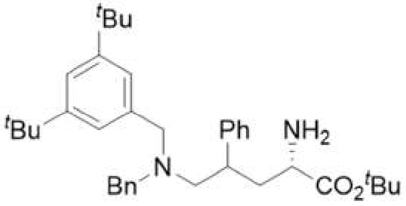

6r'
enantioenriched

===

```
Acq. Operator    : SYSTEM                         Seq. Line :  18
Sample Operator  : SYSTEM
Acq. Instrument  : HPLC                            Location :   P1-A-01
Injection Date   : 14/8/2025 3:14:39 am                 Inj :   1
                                               Inj Volume : 2.000 µl
Different Inj Volume from Sample Entry!  Actual Inj Volume : 20.000 µl
Acq. Method      : C:\Users\Public\Documents\ChemStation\1\Data\SUN\SUN 2025-08-13 21-21-01\IE3-
                   10-30.M
Last changed     : 26/9/2022 10:26:38 pm by SYSTEM
Analysis Method  : C:\Users\Public\Documents\ChemStation\1\Data\SUN\SUN 2025-08-13 21-21-01\IE3-
                   10-30.M (Sequence Method)
Last changed     : 15/8/2025 8:51:26 pm by SYSTEM
                   (modified after loading)
Additional Info  : Peak(s) manually integrated
```

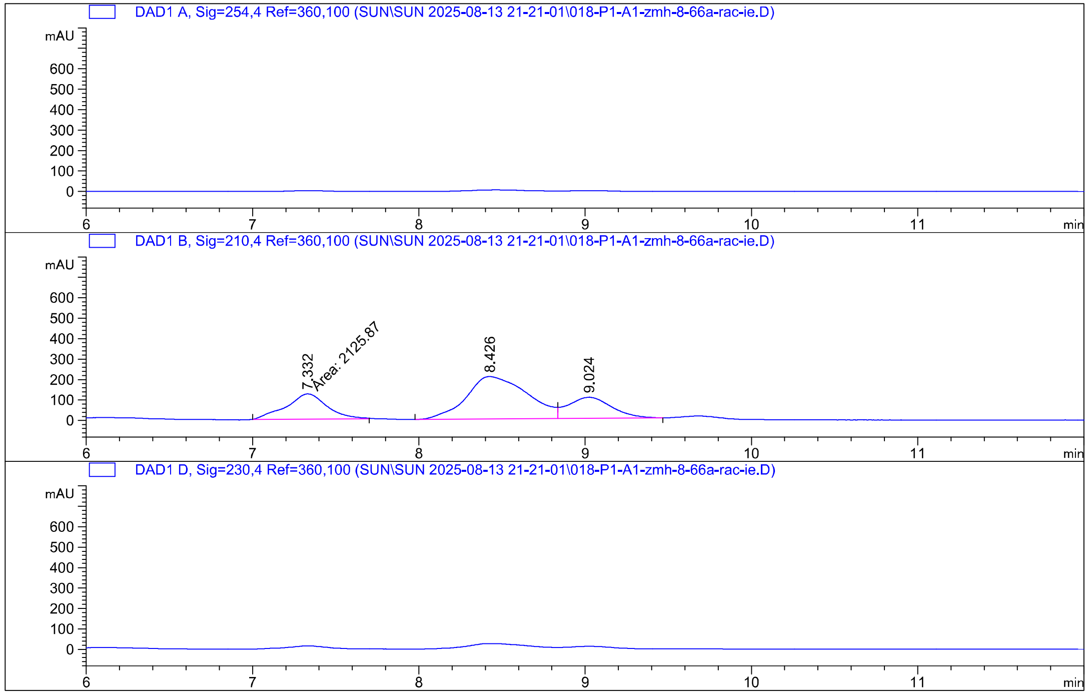

===
Area Percent Report
===

```
Sorted By             :      Signal
Multiplier            :      1.0000
Dilution              :      1.0000
Use Multiplier & Dilution Factor with ISTDs
```


Data File C:\Users\P...ion\1\Data\SUN\SUN 2025-08-13 21-21-01\018-P1-A1-zmh-8-66a-rac-ie.D
Sample Name: zmh-8-66a-rac-ie

Signal 1: DAD1 A, Sig=254,4 Ref=360,100

Signal 2: DAD1 B, Sig=210,4 Ref=360,100

Peak #	RetTime [min]	Type	Width [min]	Area [mAU*s]	Height [mAU]	Area %
1	7.332	MM	0.2850	2125.86523	124.32780	22.9431
2	8.426	BV	0.3013	5279.30127	207.14227	56.9761
3	9.024	VB	0.2282	1860.65601	103.26683	20.0809

Totals : 9265.82251 434.73690

Signal 3: DAD1 D, Sig=230,4 Ref=360,100

===
*** End of Report ***

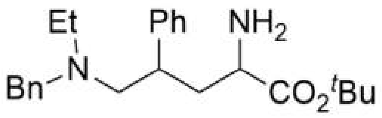

6s'
racemic

===

Acq. Operator : SYSTEM Seq. Line : 40
Sample Operator : SYSTEM
Acq. Instrument : HPLC Location : P1-A-03
Injection Date : 14/8/2025 11:11:00 am Inj : 1
Inj Volume : 2.000 µl
Different Inj Volume from Sample Entry! Actual Inj Volume : 10.000 µl
Acq. Method : C:\Users\Public\Documents\ChemStation\1\Data\SUN\SUN 2025-08-13 21-21-01\IE3-10-20.M
Last changed : 15/8/2022 10:27:27 pm by SYSTEM
Analysis Method : C:\Users\Public\Documents\ChemStation\1\Data\SUN\SUN 2025-08-13 21-21-01\IE3-10-20.M (Sequence Method)
Last changed : 15/8/2025 8:53:09 pm by SYSTEM
(modified after loading)
Additional Info : Peak(s) manually integrated

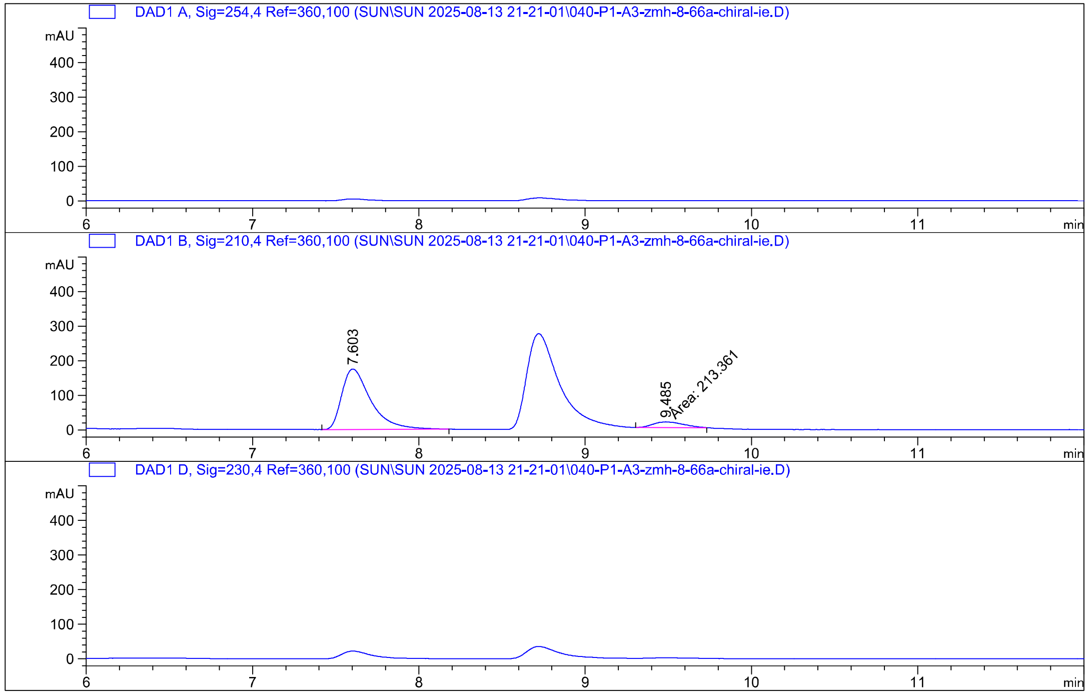

===

Area Percent Report

===

Sorted By : Signal
Multiplier : 1.0000
Dilution : 1.0000
Use Multiplier & Dilution Factor with ISTDs

Data File C:\Users\P...\1\Data\SUN\SUN 2025-08-13 21-21-01\040-P1-A3-zmh-8-66a-chiral-ie.D
Sample Name: zmh-8-66a-chiral-ie

Signal 1: DAD1 A, Sig=254,4 Ref=360,100

Signal 2: DAD1 B, Sig=210,4 Ref=360,100

Peak #	RetTime [min]	Type	Width [min]	Area [mAU*s]	Height [mAU]	Area %
1	7.603	BV R	0.1674	2117.07983	174.73648	90.8446
2	9.485	MM	0.2122	213.36130	16.75652	9.1554
Totals :				2330.44113	191.49300	

Signal 3: DAD1 D, Sig=230,4 Ref=360,100

===

*** End of Report ***

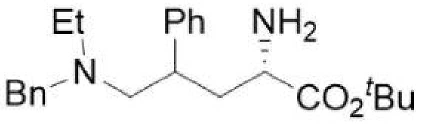

6s'
enantioenriched

Data File C:\Users\P...\Data\SUN\SUN 2025-08-12 15-12-00\058-P1-F2-zmh-8-66c-rac-ib-last.D
Sample Name: zmh-8-66c-rac-ib-last

===

Acq. Operator : SYSTEM Seq. Line : 58
Sample Operator : SYSTEM
Acq. Instrument : HPLC Location : P1-F-02
Injection Date : 13/8/2025 11:47:52 am Inj : 1
Inj Volume : 2.000 µl
Different Inj Volume from Sample Entry! Actual Inj Volume : 20.000 µl
Acq. Method : C:\Users\Public\Documents\ChemStation\1\Data\SUN\SUN 2025-08-12 15-12-00\IBN3-10-30.M
Last changed : 29/8/2022 6:09:10 pm by SYSTEM
Analysis Method : C:\Users\Public\Documents\ChemStation\1\Data\SUN\SUN 2025-08-12 15-12-00\IBN3-10-30.M (Sequence Method)
Last changed : 15/8/2025 8:45:07 pm by SYSTEM
(modified after loading)
Additional Info : Peak(s) manually integrated

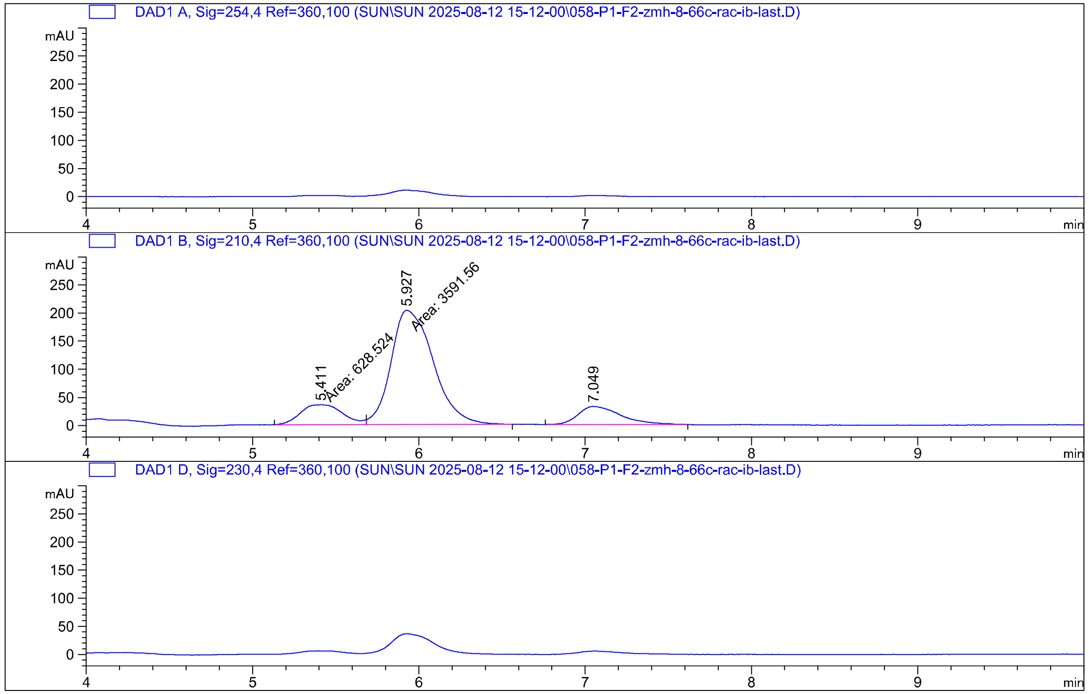

===

Area Percent Report

===

Sorted By : Signal
Multiplier : 1.0000
Dilution : 1.0000
Use Multiplier & Dilution Factor with ISTDs

Data File C:\Users\P...\Data\SUN\SUN 2025-08-12 15-12-00\058-P1-F2-zmh-8-66c-rac-ib-last.D
Sample Name: zmh-8-66c-rac-ib-last

Signal 1: DAD1 A, Sig=254,4 Ref=360,100

Signal 2: DAD1 B, Sig=210,4 Ref=360,100

Peak #	RetTime [min]	Type	Width [min]	Area [mAU*s]	Height [mAU]	Area %
1	5.411	MF	0.2965	628.52423	35.32851	13.0860
2	5.927	FM	0.2947	3591.56445	203.11841	74.7769
3	7.049	VV R	0.2125	582.94940	32.46632	12.1371

Totals : 4803.03809 270.91324

Signal 3: DAD1 D, Sig=230,4 Ref=360,100

===

*** End of Report ***

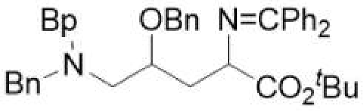

6t'

racemic

Data File C:\Users\P...ion\1\Data\SUN\SUN 2025-08-12 15-12-00\055-P1-F1-zmh-8-66c-chiral.D
Sample Name: zmh-8-66c-chiral

===

```
Acq. Operator   : SYSTEM                          Seq. Line :   55
Sample Operator : SYSTEM
Acq. Instrument : HPLC                             Location :   P1-F-01
Injection Date  : 13/8/2025 10:34:02 am                 Inj :   1
                                                Inj Volume : 2.000 µl
Different Inj Volume from Sample Entry!  Actual Inj Volume : 10.000 µl
Acq. Method     : C:\Users\Public\Documents\ChemStation\1\Data\SUN\SUN 2025-08-12 15-12-00\IBN3-
                  10-30.M
Last changed    : 29/8/2022 6:09:10 pm by SYSTEM
Analysis Method : C:\Users\Public\Documents\ChemStation\1\Data\SUN\SUN 2025-08-12 15-12-00\IBN3-
                  10-30.M (Sequence Method)
Last changed    : 15/8/2025 8:47:04 pm by SYSTEM
                  (modified after loading)
Additional Info : Peak(s) manually integrated
```

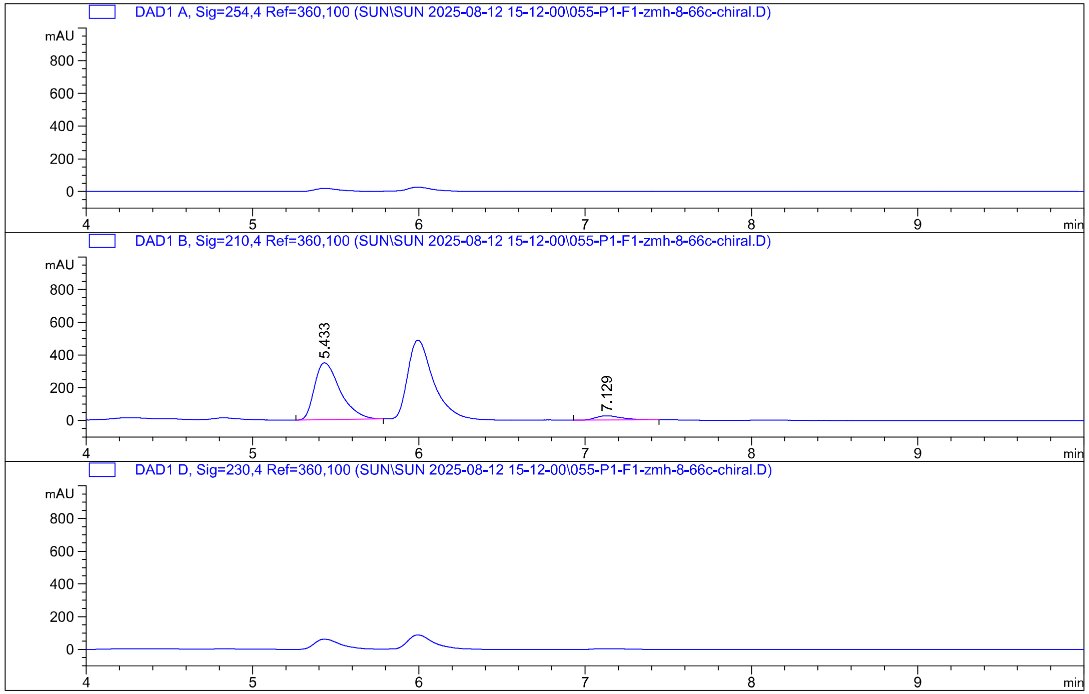

===
Area Percent Report
===

```
Sorted By             :      Signal
Multiplier            :      1.0000
Dilution              :      1.0000
Use Multiplier & Dilution Factor with ISTDs
```


Data File C:\Users\P...ion\1\Data\SUN\SUN 2025-08-12 15-12-00\055-P1-F1-zmh-8-66c-chiral.D
Sample Name: zmh-8-66c-chiral

Signal 1: DAD1 A, Sig=254,4 Ref=360,100

Signal 2: DAD1 B, Sig=210,4 Ref=360,100

Peak #	RetTime [min]	Type	Width [min]	Area [mAU*s]	Height [mAU]	Area %
1	5.433	BV R	0.1626	3719.53613	348.13928	93.0587
2	7.129	VV R	0.1264	277.44025	26.22421	6.9413

Totals : 3996.97638 374.36349

Signal 3: DAD1 D, Sig=230,4 Ref=360,100

===
*** End of Report ***

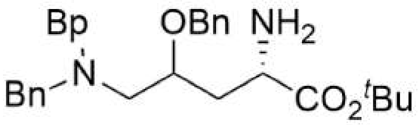

6t'
enantioenriched

===

Acq. Operator : SYSTEM Seq. Line : 5
Sample Operator : SYSTEM
Acq. Instrument : HPLC Location : P2-F-01
Injection Date : 10/10/2022 10:58:45 am Inj : 1
Inj Volume : 2.000 µl
Different Inj Volume from Sample Entry! Actual Inj Volume : 5.000 µl
Acq. Method : C:\Users\Public\Documents\ChemStation\1\Data\SUN\SUN 2022-10-10 10-03-12\AY3-10-20.M
Last changed : 15/8/2022 10:25:04 pm by SYSTEM
Analysis Method : C:\Users\Public\Documents\ChemStation\1\Data\SUN\SUN 2022-10-10 10-03-12\AY3-10-20.M (Sequence Method)
Last changed : 31/8/2023 10:07:06 pm by SYSTEM
(modified after loading)
Additional Info : Peak(s) manually integrated

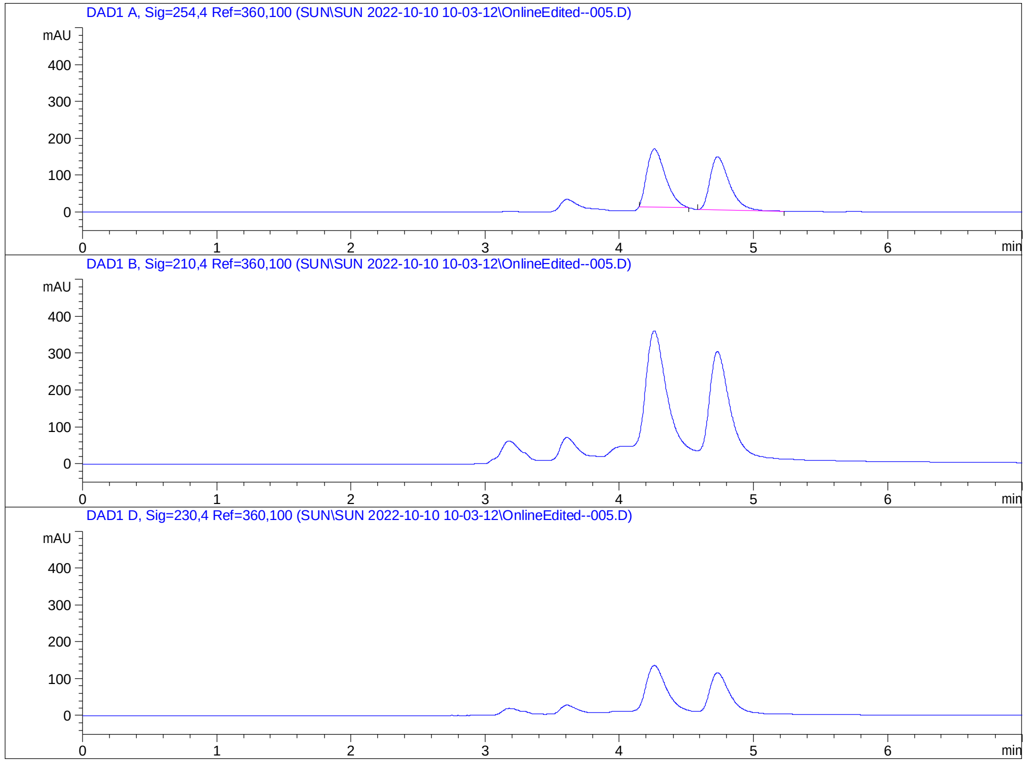

===

Area Percent Report

===

Sorted By : Signal
Multiplier : 1.0000
Dilution : 1.0000
Use Multiplier & Dilution Factor with ISTDs

Signal 1: DAD1 A, Sig=254,4 Ref=360,100

Peak #	RetTime [min]	Type	Width [min]	Area [mAU*s]	Height [mAU]	Area %
1	4.261	MM	0.1599	1514.25537	157.83392	50.7330
2	4.733	MM	0.1692	1470.49683	144.82106	49.2670
Totals :				2984.75220	302.65498	

Signal 2: DAD1 B, Sig=210,4 Ref=360,100

Signal 3: DAD1 D, Sig=230,4 Ref=360,100

===

*** End of Report ***

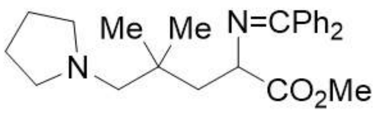

6u

racemic

===

Acq. Operator : SYSTEM Seq. Line : 3
Sample Operator : SYSTEM
Acq. Instrument : HPLC Location : P1-A-04
Injection Date : 13/1/2023 9:45:13 am Inj : 1
Inj Volume : 2.000 µl
Different Inj Volume from Sample Entry! Actual Inj Volume : 5.000 µl
Acq. Method : C:\Users\Public\Documents\ChemStation\1\Data\SUN\SUN 2023-01-13 09-11-35 \AY3-10-20.M
Last changed : 15/8/2022 10:25:04 pm by SYSTEM
Analysis Method : C:\Users\Public\Documents\ChemStation\1\Data\SUN\SUN 2023-01-13 09-11-35 \AY3-10-20.M (Sequence Method)
Last changed : 31/8/2023 10:12:17 pm by SYSTEM
(modified after loading)
Additional Info : Peak(s) manually integrated

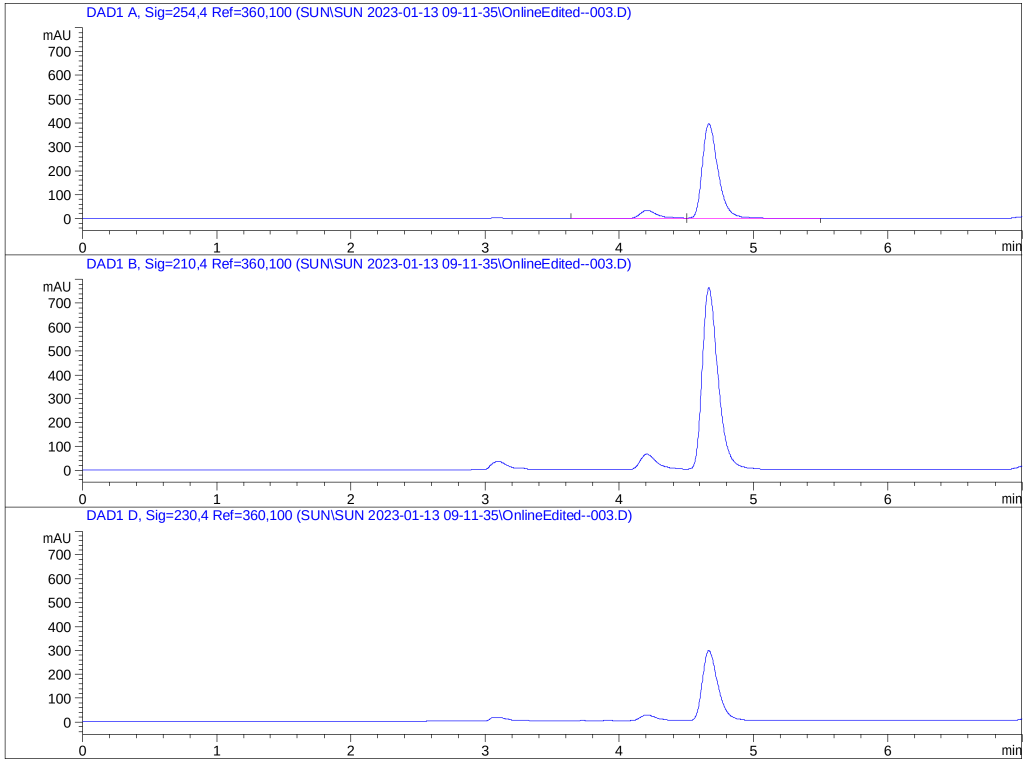

Data File C:\Users\P...\ChemStation\1\Data\SUN\SUN 2023-01-13 09-11-35\OnlineEdited--003.D
Sample Name: zmh-2-116a-15h

===
Area Percent Report
===

Sorted By : Signal
Multiplier : 1.0000
Dilution : 1.0000
Use Multiplier & Dilution Factor with ISTDs

Signal 1: DAD1 A, Sig=254,4 Ref=360,100

Peak #	RetTime [min]	Type	Width [min]	Area [mAU*s]	Height [mAU]	Area %
1	4.206	VV R	0.1367	303.70743	33.05610	8.6157
2	4.668	VB	0.1242	3221.35229	397.04892	91.3843

Totals : 3525.05972 430.10502

Signal 2: DAD1 B, Sig=210,4 Ref=360,100

Signal 3: DAD1 D, Sig=230,4 Ref=360,100

===
*** End of Report ***

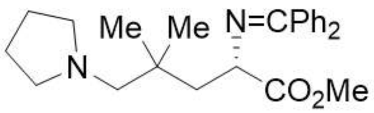

6u
enantioenriched

===

Acq. Operator : SYSTEM Seq. Line : 2
Sample Operator : SYSTEM
Acq. Instrument : HPLC Location : P2-A-11
Injection Date : 2/12/2022 9:59:43 am Inj : 1
Inj Volume : 2.000 µl
Different Inj Volume from Sample Entry! Actual Inj Volume : 10.000 µl
Acq. Method : C:\Users\Public\Documents\ChemStation\1\Data\SUN\SUN 2022-12-02 09-47-09\AY3-10-20.M
Last changed : 15/8/2022 10:25:04 pm by SYSTEM
Analysis Method : C:\Users\Public\Documents\ChemStation\1\Data\SUN\SUN 2022-12-02 09-47-09\AY3-10-20.M (Sequence Method)
Last changed : 26/1/2023 4:25:09 pm by SYSTEM
(modified after loading)
Additional Info : Peak(s) manually integrated

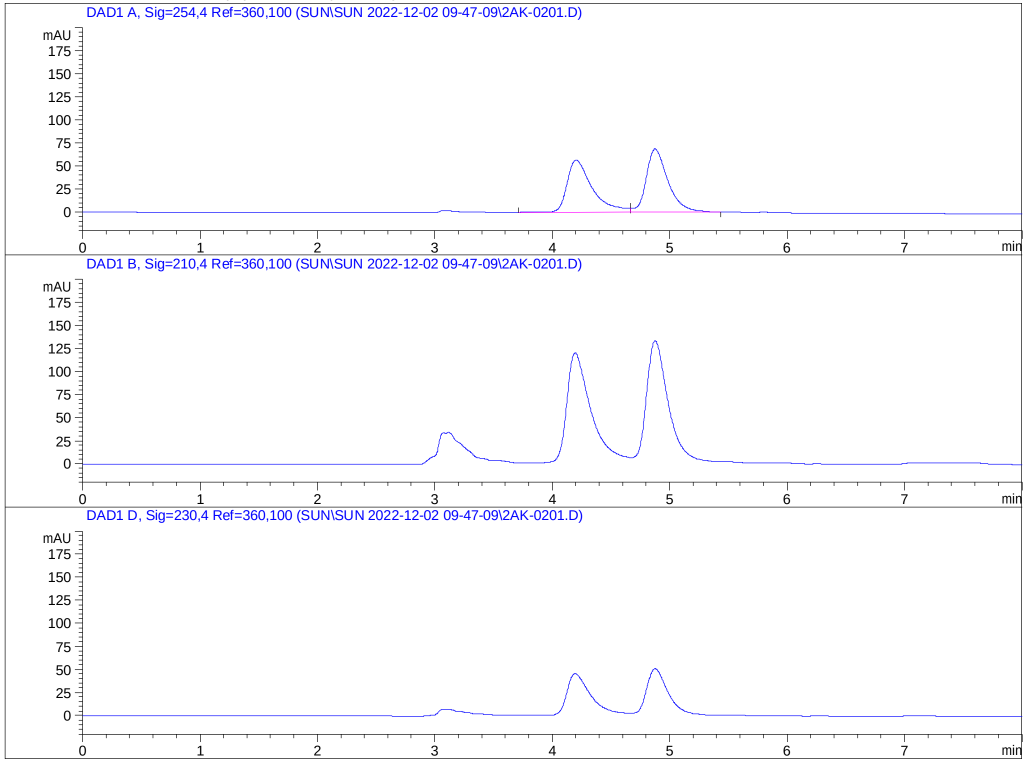

Data File C:\Users\P...Documents\ChemStation\1\Data\SUN\SUN 2022-12-02 09-47-09\2AK-0201.D
Sample Name: zmh-2-57a-rac

===

Area Percent Report

===

Sorted By : Signal
Multiplier : 1.0000
Dilution : 1.0000
Use Multiplier & Dilution Factor with ISTDs

Signal 1: DAD1 A, Sig=254,4 Ref=360,100

Peak #	RetTime [min]	Type	Width [min]	Area [mAU*s]	Height [mAU]	Area %
1	4.204	BV	0.2210	845.45947	56.69910	49.0791
2	4.875	VB	0.1919	877.18848	68.51388	50.9209

Totals : 1722.64795 125.21297

Signal 2: DAD1 B, Sig=210,4 Ref=360,100

Signal 3: DAD1 D, Sig=230,4 Ref=360,100

===

*** End of Report ***

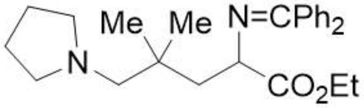

6v
racemic

===

Acq. Operator : SYSTEM   Seq. Line : 33
Sample Operator : SYSTEM
Acq. Instrument : HPLC   Location : P2-A-02
Injection Date : 30/8/2023 1:58:38 am   Inj : 1
Inj Volume : 2.000 µl
Different Inj Volume from Sample Entry! Actual Inj Volume : 20.000 µl
Acq. Method : C:\Users\Public\Documents\ChemStation\1\Data\SUN\SUN 2023-08-29 14-05-44\AY3-10-20.M
Last changed : 15/8/2022 10:25:04 pm by SYSTEM
Analysis Method : C:\Users\Public\Documents\ChemStation\1\Data\SUN\SUN 2023-08-29 14-05-44\AY3-10-20.M (Sequence Method)
Last changed : 31/8/2023 10:17:48 pm by SYSTEM
(modified after loading)
Additional Info : Peak(s) manually integrated

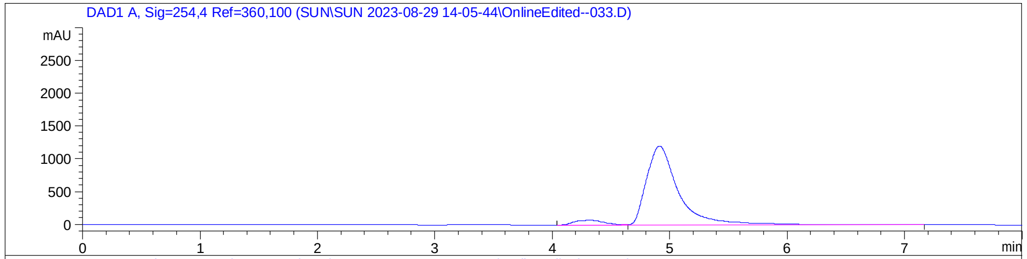

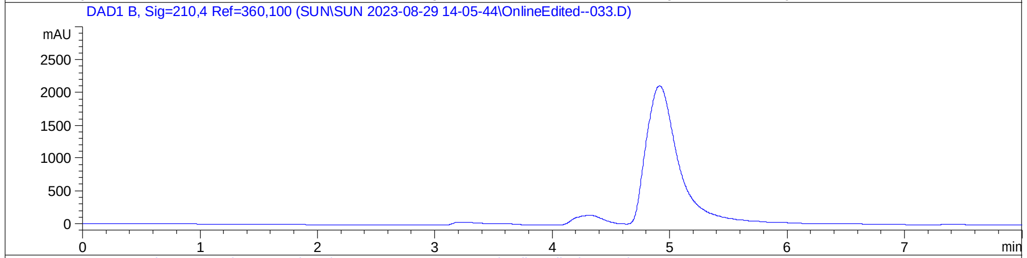

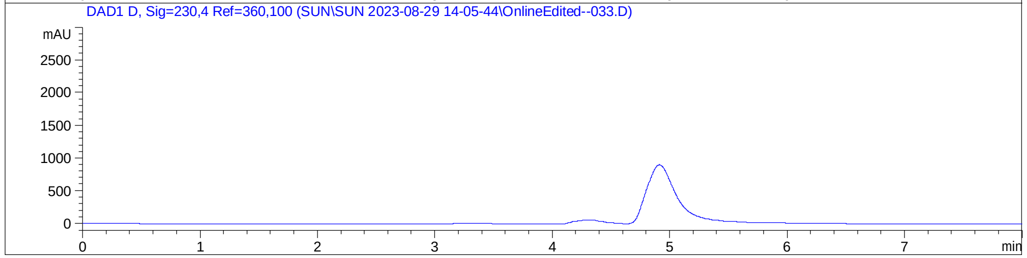

Data File C:\Users\P...\ChemStation\1\Data\SUN\SUN 2023-08-29 14-05-44\OnlineEdited--033.D
Sample Name: zmh-4-85b

===

Area Percent Report

===

Sorted By : Signal
Multiplier : 1.0000
Dilution : 1.0000
Use Multiplier & Dilution Factor with ISTDs

Signal 1: DAD1 A, Sig=254,4 Ref=360,100

Peak #	RetTime [min]	Type	Width [min]	Area [mAU*s]	Height [mAU]	Area %
1	4.321	BV E	0.2743	1315.85364	76.63140	5.3978
2	4.913	VB R	0.2940	2.30617e4	1203.57422	94.6022

Totals : 2.43775e4 1280.20562

Signal 2: DAD1 B, Sig=210,4 Ref=360,100

Signal 3: DAD1 D, Sig=230,4 Ref=360,100

===

*** End of Report ***

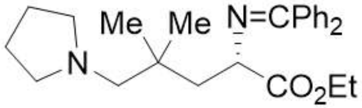

6v
enantioenriched

===

Acq. Operator : SYSTEM Seq. Line : 4
Sample Operator : SYSTEM
Acq. Instrument : HPLC Location : P2-A-10
Injection Date : 2/12/2022 10:31:44 am Inj : 1
Inj Volume : 2.000 µl
Different Inj Volume from Sample Entry! Actual Inj Volume : 10.000 µl
Acq. Method : C:\Users\Public\Documents\ChemStation\1\Data\SUN\SUN 2022-12-02 09-47-09\AY3-10-20.M
Last changed : 15/8/2022 10:25:04 pm by SYSTEM
Analysis Method : C:\Users\Public\Documents\ChemStation\1\Data\SUN\SUN 2022-12-02 09-47-09\AY3-10-20.M (Sequence Method)
Last changed : 26/1/2023 4:26:37 pm by SYSTEM
(modified after loading)
Additional Info : Peak(s) manually integrated

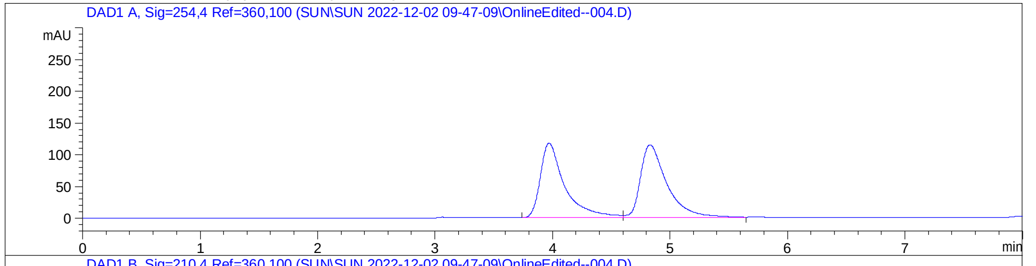

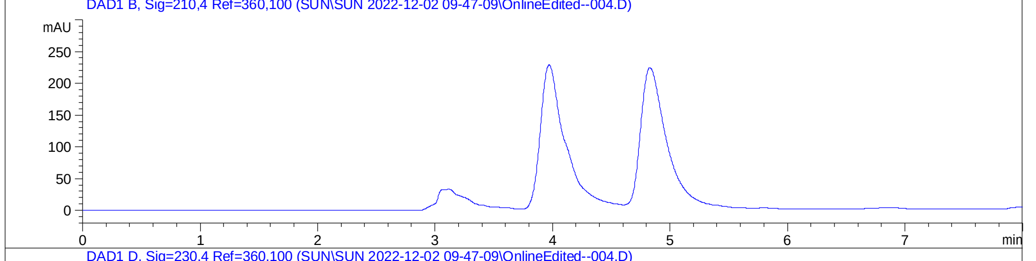

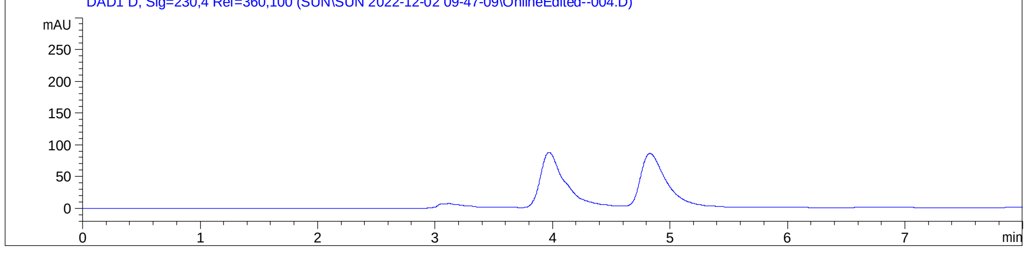

Data File C:\Users\P...\ChemStation\1\Data\SUN\SUN 2022-12-02 09-47-09\OnlineEdited--004.D
Sample Name: zmh-2-57b-rac

===

Area Percent Report

===

Sorted By : Signal
Multiplier : 1.0000
Dilution : 1.0000
Use Multiplier & Dilution Factor with ISTDs

Signal 1: DAD1 A, Sig=254,4 Ref=360,100

Peak #	RetTime [min]	Type	Width [min]	Area [mAU*s]	Height [mAU]	Area %
1	3.971	BV	0.2093	1697.47168	117.34574	49.0244
2	4.830	VB	0.2279	1765.03198	114.15398	50.9756

Totals : 3462.50366 231.49973

Signal 2: DAD1 B, Sig=210,4 Ref=360,100

Signal 3: DAD1 D, Sig=230,4 Ref=360,100

===

*** End of Report ***

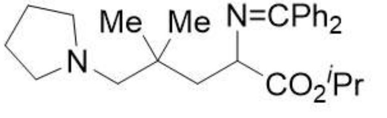

6w
racemic

===

Acq. Operator : SYSTEM Seq. Line : 35
Sample Operator : SYSTEM
Acq. Instrument : HPLC Location : P2-A-03
Injection Date : 30/8/2023 2:30:49 am Inj : 1
Inj Volume : 2.000 µl
Different Inj Volume from Sample Entry! Actual Inj Volume : 20.000 µl
Acq. Method : C:\Users\Public\Documents\ChemStation\1\Data\SUN\SUN 2023-08-29 14-05-44\AY3-10-20.M
Last changed : 15/8/2022 10:25:04 pm by SYSTEM
Analysis Method : C:\Users\Public\Documents\ChemStation\1\Data\SUN\SUN 2023-08-29 14-05-44\AY3-10-20.M (Sequence Method)
Last changed : 31/8/2023 10:17:48 pm by SYSTEM
(modified after loading)
Additional Info : Peak(s) manually integrated

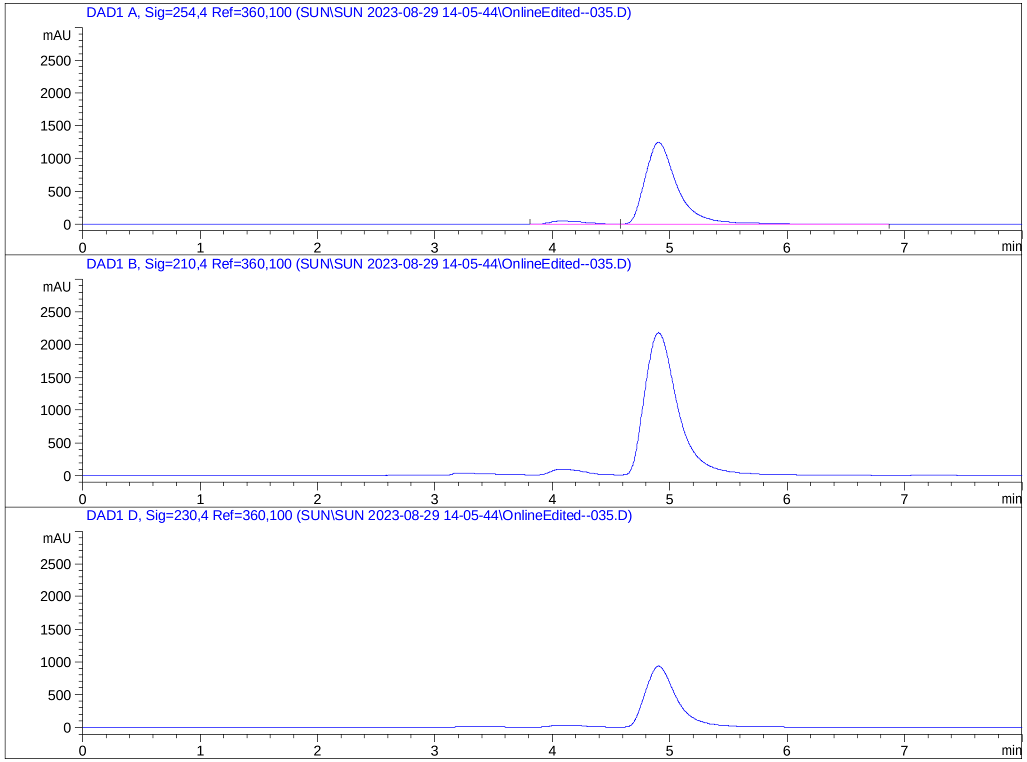

Data File C:\Users\P...\ChemStation\1\Data\SUN\SUN 2023-08-29 14-05-44\OnlineEdited--035.D
Sample Name: zmh-4-85c

===

Area Percent Report

===

Sorted By : Signal
Multiplier : 1.0000
Dilution : 1.0000
Use Multiplier & Dilution Factor with ISTDs

Signal 1: DAD1 A, Sig=254,4 Ref=360,100

Peak #	RetTime [min]	Type	Width [min]	Area [mAU*s]	Height [mAU]	Area %
1	4.080	BV	0.2672	914.14868	47.66854	3.7446
2	4.906	VB	0.2857	2.34984e4	1244.74036	96.2554

Totals : 2.44126e4 1292.40889

Signal 2: DAD1 B, Sig=210,4 Ref=360,100

Signal 3: DAD1 D, Sig=230,4 Ref=360,100

===

*** End of Report ***

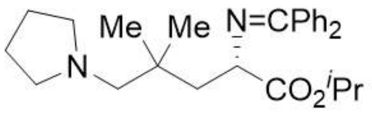

6w
enantioenriched

===

Acq. Operator : SYSTEM Seq. Line : 22
Sample Operator : SYSTEM
Acq. Instrument : HPLC Location : P2-A-01
Injection Date : 27/9/2025 7:00:49 pm Inj : 1
Inj Volume : 2.000 µl
Different Inj Volume from Sample Entry! Actual Inj Volume : 20.000 µl
Acq. Method : C:\Users\Public\Documents\ChemStation\1\Data\SUN\SUN 2025-09-27 13-20-43\IE3-03-40.M
Last changed : 10/6/2023 2:54:35 pm by SYSTEM
Analysis Method : C:\Users\Public\Documents\ChemStation\1\Data\SUN\SUN 2025-09-27 13-20-43\IE3-03-40.M (Sequence Method)
Last changed : 28/9/2025 5:08:30 pm by SYSTEM
(modified after loading)
Additional Info : Peak(s) manually integrated

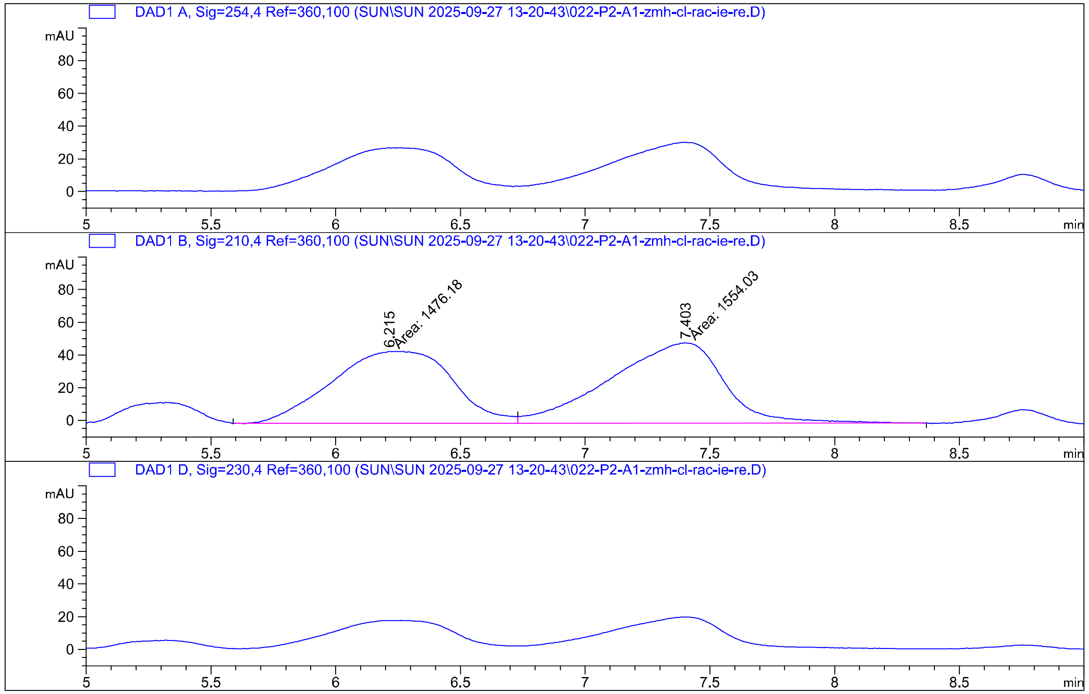

===

Area Percent Report

===

Sorted By : Signal
Multiplier : 1.0000
Dilution : 1.0000
Use Multiplier & Dilution Factor with ISTDs

Signal 1: DAD1 A, Sig=254,4 Ref=360,100

Signal 2: DAD1 B, Sig=210,4 Ref=360,100

Peak #	RetTime [min]	Type	Width [min]	Area [mAU*s]	Height [mAU]	Area %
1	6.215	MF	0.5606	1476.18091	43.88378	48.7155
2	7.403	FM	0.5283	1554.02551	49.02534	51.2845

Totals : 3030.20642 92.90912

Signal 3: DAD1 D, Sig=230,4 Ref=360,100

===

*** End of Report ***

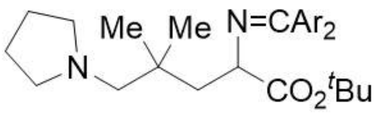

Ar = *p*-ClC_6H_4

6x

racemic

===

Acq. Operator : SYSTEM   Seq. Line : 2
Sample Operator : SYSTEM
Acq. Instrument : HPLC   Location : P2-A-02
Injection Date : 28/9/2025 2:10:42 pm   Inj : 1
Inj Volume : 2.000 µl
Different Inj Volume from Sample Entry! Actual Inj Volume : 10.000 µl
Acq. Method : C:\Users\Public\Documents\ChemStation\1\Data\SUN\SUN 2025-09-28 13-57-22\IE3-03-10.M
Last changed : 22/11/2023 10:31:59 am by SYSTEM
Analysis Method : C:\Users\Public\Documents\ChemStation\1\Data\SUN\SUN 2025-09-28 13-57-22\IE3-03-10.M (Sequence Method)
Last changed : 28/9/2025 5:10:26 pm by SYSTEM
(modified after loading)
Additional Info : Peak(s) manually integrated

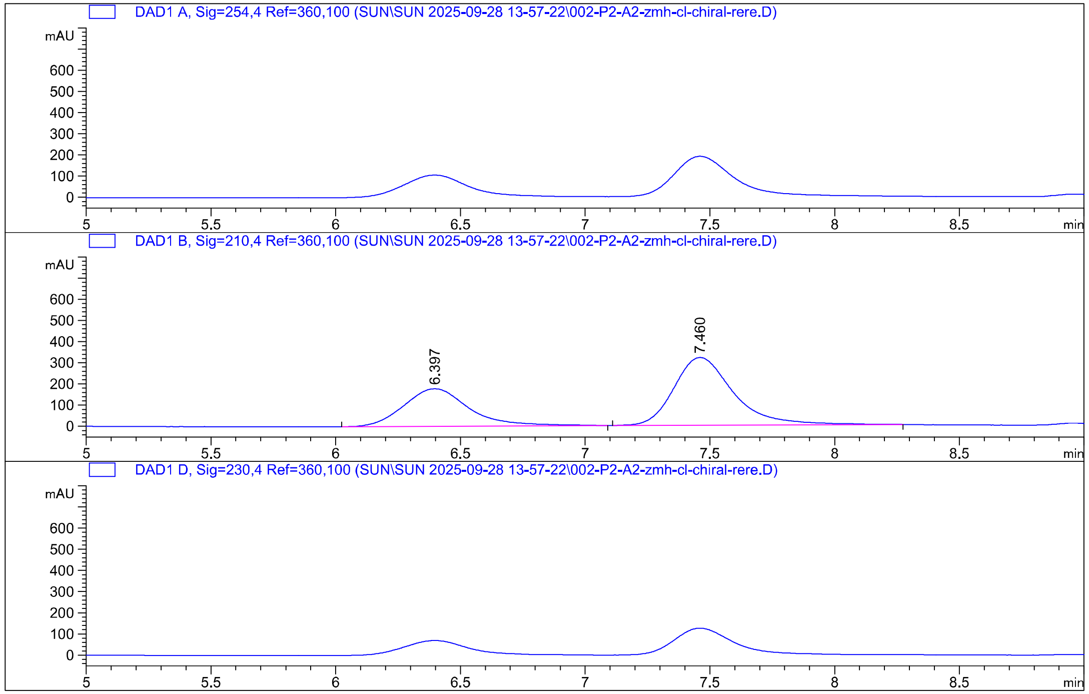

===

Area Percent Report

===

Sorted By : Signal
Multiplier : 1.0000
Dilution : 1.0000
Use Multiplier & Dilution Factor with ISTDs

Data File C:\Users\P...n\1\Data\SUN\SUN 2025-09-28 13-57-22\002-P2-A2-zmh-cl-chiral-rere.D
Sample Name: zmh-cl-chiral-rere

Signal 1: DAD1 A, Sig=254,4 Ref=360,100

Signal 2: DAD1 B, Sig=210,4 Ref=360,100

Peak #	RetTime [min]	Type	Width [min]	Area [mAU*s]	Height [mAU]	Area %
1	6.397	BV R	0.2525	3233.89771	177.57298	37.8666
2	7.460	BV R	0.2298	5306.33594	319.44064	62.1334

Totals : 8540.23364 497.01363

Signal 3: DAD1 D, Sig=230,4 Ref=360,100

===

*** End of Report ***

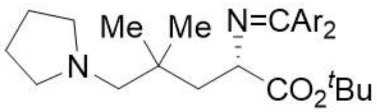

Ar = p-ClC_6H_4

6x

enantioenriched

Data File C:\Users\P...ation\1\Data\SUN\SUN 2025-09-25 22-42-05\045-P2-A2-zmh-ome-rac-ie.D
Sample Name: zmh-ome-rac-ie

===

```
Acq. Operator   : SYSTEM                          Seq. Line :  45
Sample Operator : SYSTEM
Acq. Instrument : HPLC                             Location :   P2-A-02
Injection Date  : 27/9/2025 1:01:26 am                  Inj :   1
                                               Inj Volume : 2.000 µl
Different Inj Volume from Sample Entry!  Actual Inj Volume : 20.000 µl
Acq. Method     : C:\Users\Public\Documents\ChemStation\1\Data\SUN\SUN 2025-09-25 22-42-05\IE3-
                  10-20.M
Last changed    : 15/8/2022 10:27:27 pm by SYSTEM
Analysis Method : C:\Users\Public\Documents\ChemStation\1\Data\SUN\SUN 2025-09-25 22-42-05\IE3-
                  10-20.M (Sequence Method)
Last changed    : 28/9/2025 5:03:25 pm by SYSTEM
                  (modified after loading)
Additional Info : Peak(s) manually integrated
```

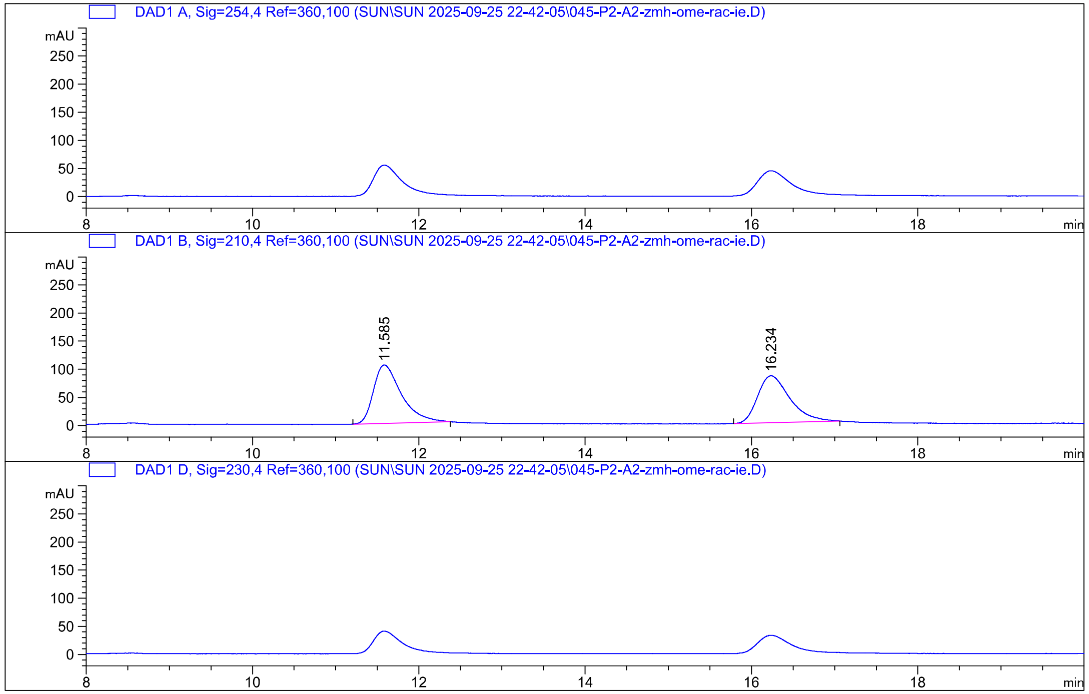

===
Area Percent Report
===

```
Sorted By             :      Signal
Multiplier            :      1.0000
Dilution              :      1.0000
Use Multiplier & Dilution Factor with ISTDs
```


Data File C:\Users\P...ation\1\Data\SUN\SUN 2025-09-25 22-42-05\045-P2-A2-zmh-ome-rac-ie.D
Sample Name: zmh-ome-rac-ie

Signal 1: DAD1 A, Sig=254,4 Ref=360,100

Signal 2: DAD1 B, Sig=210,4 Ref=360,100

Peak #	RetTime [min]	Type	Width [min]	Area [mAU*s]	Height [mAU]	Area %
1	11.585	BV R	0.2745	2428.74097	103.60870	51.7858
2	16.234	VV R	0.3190	2261.23413	83.60545	48.2142
Totals :				4689.97510	187.21416	

Signal 3: DAD1 D, Sig=230,4 Ref=360,100

===
*** End of Report ***

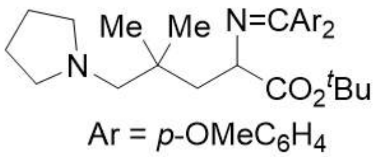

6y
racemic

===

Acq. Operator : SYSTEM Seq. Line : 2
Sample Operator : SYSTEM
Acq. Instrument : HPLC Location : P1-A-01
Injection Date : 28/9/2025 4:03:17 pm Inj : 1
Inj Volume : 2.000 µl
Different Inj Volume from Sample Entry! Actual Inj Volume : 10.000 µl
Acq. Method : C:\Users\Public\Documents\ChemStation\1\Data\SUN\SUN 2025-09-28 15-49-57\IE3-10-30.M
Last changed : 26/9/2022 10:26:38 pm by SYSTEM
Analysis Method : C:\Users\Public\Documents\ChemStation\1\Data\SUN\SUN 2025-09-28 15-49-57\IE3-10-30.M (Sequence Method)
Last changed : 28/9/2025 5:06:11 pm by SYSTEM
(modified after loading)
Additional Info : Peak(s) manually integrated

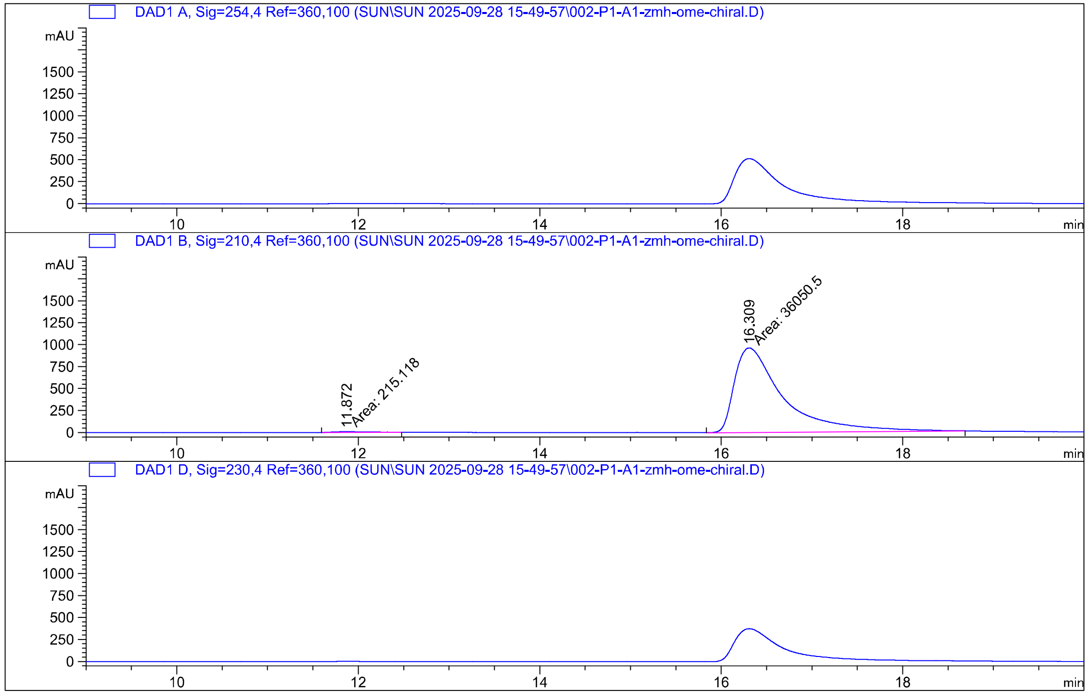

===

Area Percent Report

===

Sorted By : Signal
Multiplier : 1.0000
Dilution : 1.0000
Use Multiplier & Dilution Factor with ISTDs

Data File C:\Users\P...ation\1\Data\SUN\SUN 2025-09-28 15-49-57\002-P1-A1-zmh-ome-chiral.D
Sample Name: zmh-ome-chiral

Signal 1: DAD1 A, Sig=254,4 Ref=360,100

Signal 2: DAD1 B, Sig=210,4 Ref=360,100

Peak #	RetTime [min]	Type	Width [min]	Area [mAU*s]	Height [mAU]	Area %
1	11.872	MM	0.3664	215.11794	9.78537	0.5932
2	16.309	MM	0.6250	3.60505e4	961.33771	99.4068

Totals : 3.62656e4 971.12308

Signal 3: DAD1 D, Sig=230,4 Ref=360,100

===

*** End of Report ***

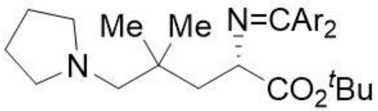

Ar = *p*-OMeC_6H_4

6y

enantioenriched

Data File C:\Users\P...Documents\ChemStation\1\Data\SUN\SUN 2023-01-16 11-03-49\1BJ-0801.D
Sample Name: zmh-2-112-rac-ia

===

```
Acq. Operator   : SYSTEM                        Seq. Line :    8
Sample Operator : SYSTEM
Acq. Instrument : HPLC                           Location :   P1-B-10
Injection Date  : 16/1/2023 12:52:58 pm               Inj :    1
                                               Inj Volume : 2.000 µl
Different Inj Volume from Sample Entry!  Actual Inj Volume : 10.000 µl
Acq. Method     : C:\Users\Public\Documents\ChemStation\1\Data\SUN\SUN 2023-01-16 11-03-49
                  \IA3-10-20.M
Last changed    : 15/8/2022 10:26:10 pm by SYSTEM
Analysis Method : C:\Users\Public\Documents\ChemStation\1\Data\SUN\SUN 2023-01-16 11-03-49
                  \IA3-10-20.M (Sequence Method)
Last changed    : 30/1/2023 4:54:23 pm by SYSTEM
                  (modified after loading)
Additional Info : Peak(s) manually integrated
```

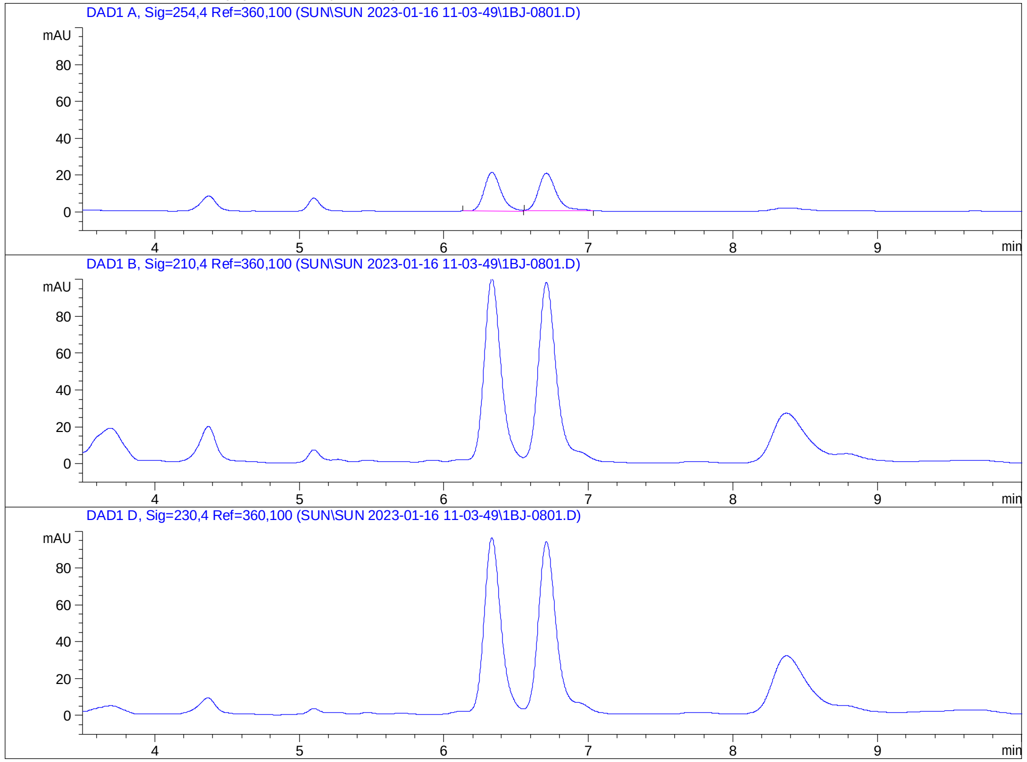

Data File C:\Users\P...Documents\ChemStation\1\Data\SUN\SUN 2023-01-16 11-03-49\1BJ-0801.D
Sample Name: zmh-2-112-rac-ia

===
Area Percent Report
===

```
Sorted By             :      Signal
Multiplier            :      1.0000
Dilution              :      1.0000
Use Multiplier & Dilution Factor with ISTDs
```

Signal 1: DAD1 A, Sig=254,4 Ref=360,100

Peak #	RetTime [min]	Type	Width [min]	Area [mAU*s]	Height [mAU]	Area %
1	6.332	BV	0.1245	172.30533	21.15444	49.7018
2	6.709	MM R	0.1431	174.37315	20.31386	50.2982

Totals : 346.67848 41.46829

Signal 2: DAD1 B, Sig=210,4 Ref=360,100

Peak #	RetTime [min]	Type	Width [min]	Area [mAU*s]	Height [mAU]	Area %
1	18.527	BBA	0.9248	651.04401	8.24435	100.0000

Totals : 651.04401 8.24435

Signal 3: DAD1 D, Sig=230,4 Ref=360,100

===
*** End of Report ***

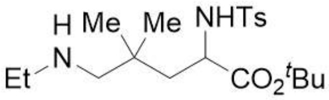

11
racemic

===

Acq. Operator : SYSTEM Seq. Line : 14
Sample Operator : SYSTEM
Acq. Instrument : HPLC Location : P1-A-02
Injection Date : 20/1/2023 6:01:53 pm Inj : 1
Inj Volume : 2.000 µl
Different Inj Volume from Sample Entry! Actual Inj Volume : 5.000 µl
Acq. Method : C:\Users\Public\Documents\ChemStation\1\Data\SUN\SUN 2023-01-20 13-06-19\IA3-10-20.M
Last changed : 15/8/2022 10:26:10 pm by SYSTEM
Analysis Method : C:\Users\Public\Documents\ChemStation\1\Data\SUN\SUN 2023-01-20 13-06-19\IA3-10-20.M (Sequence Method)
Last changed : 30/1/2023 4:58:38 pm by SYSTEM
(modified after loading)
Additional Info : Peak(s) manually integrated

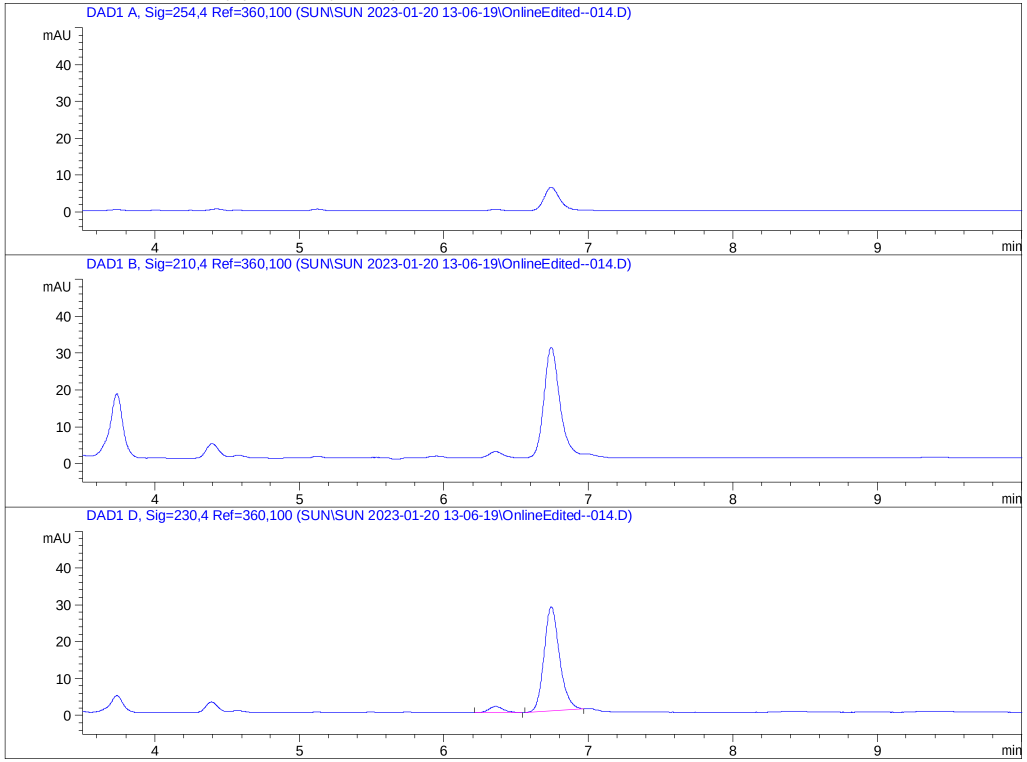

Data File C:\Users\P...\ChemStation\1\Data\SUN\SUN 2023-01-20 13-06-19\OnlineEdited--014.D
Sample Name: zmh-2-121-chiral

===

Area Percent Report

===

Sorted By : Signal
Multiplier : 1.0000
Dilution : 1.0000
Use Multiplier & Dilution Factor with ISTDs

Signal 1: DAD1 A, Sig=254,4 Ref=360,100

Signal 2: DAD1 B, Sig=210,4 Ref=360,100

Signal 3: DAD1 D, Sig=230,4 Ref=360,100

Peak #	RetTime [min]	Type	Width [min]	Area [mAU*s]	Height [mAU]	Area %
1	6.360	BB	0.1060	11.51791	1.64868	5.2084
2	6.743	BB	0.1135	209.62469	28.11124	94.7916

Totals : 221.14261 29.75992

===

*** End of Report ***

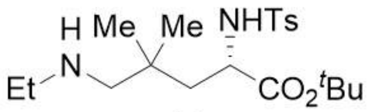

11
enantioenriched

===

Acq. Operator : SYSTEM Seq. Line : 14
Sample Operator : SYSTEM
Acq. Instrument : HPLC Location : P1-F-03
Injection Date : 19/1/2023 1:15:53 pm Inj : 1
Inj Volume : 2.000 µl
Different Inj Volume from Sample Entry! Actual Inj Volume : 5.000 µl
Acq. Method : C:\Users\Public\Documents\ChemStation\1\Data\SUN\SUN 2023-01-19 09-19-15 \OD3-10-20.M
Last changed : 15/8/2022 10:27:52 pm by SYSTEM
Analysis Method : C:\Users\Public\Documents\ChemStation\1\Data\SUN\SUN 2023-01-19 09-19-15 \OD3-10-20.M (Sequence Method)
Last changed : 31/8/2023 10:28:04 pm by SYSTEM
(modified after loading)
Additional Info : Peak(s) manually integrated

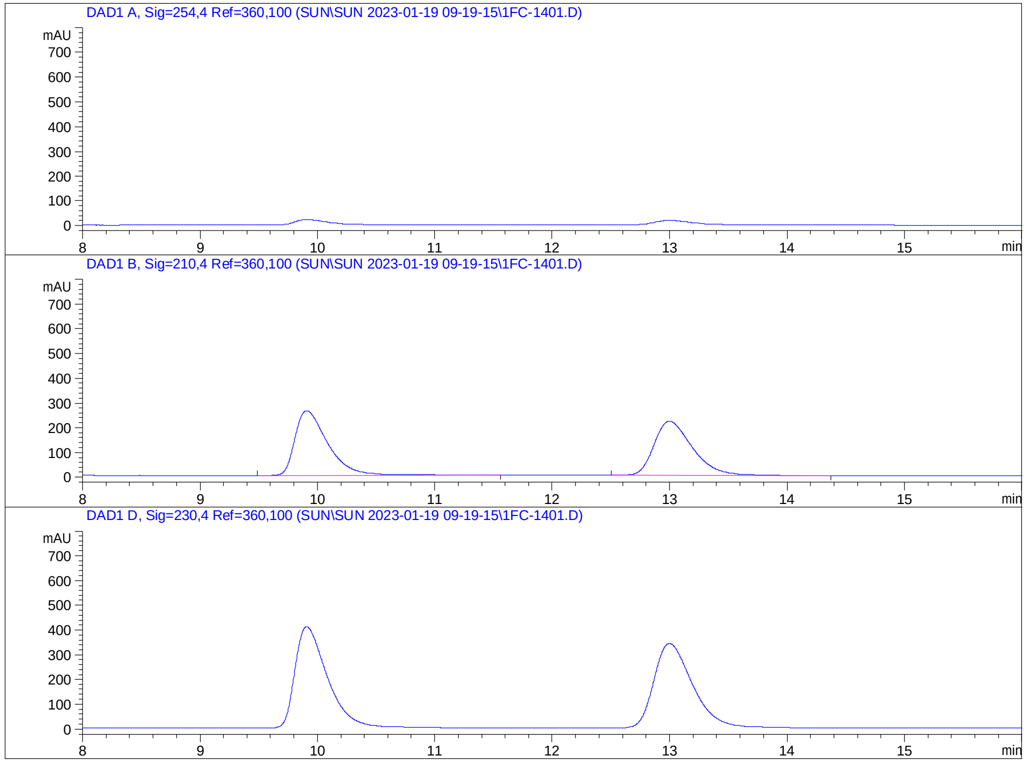

Data File C:\Users\P...Documents\ChemStation\1\Data\SUN\SUN 2023-01-19 09-19-15\1FC-1401.D
Sample Name: zmh-2-119-rac-od

===

Area Percent Report

===

Sorted By : Signal
Multiplier : 1.0000
Dilution : 1.0000
Use Multiplier & Dilution Factor with ISTDs

Signal 1: DAD1 A, Sig=254,4 Ref=360,100

Signal 2: DAD1 B, Sig=210,4 Ref=360,100

Peak #	RetTime [min]	Type	Width [min]	Area [mAU*s]	Height [mAU]	Area %
1	9.909	BB	0.2935	5068.68994	261.02487	50.5154
2	12.998	BB	0.3487	4965.26758	218.05627	49.4846

Totals : 1.00340e4 479.08115

Signal 3: DAD1 D, Sig=230,4 Ref=360,100

===

*** End of Report ***

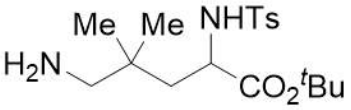

12
racemic

===

Acq. Operator : SYSTEM Seq. Line : 4
Sample Operator : SYSTEM
Acq. Instrument : HPLC Location : P1-A-04
Injection Date : 20/1/2023 10:03:14 pm Inj : 1
Inj Volume : 2.000 µl
Different Inj Volume from Sample Entry! Actual Inj Volume : 10.000 µl
Acq. Method : C:\Users\Public\Documents\ChemStation\1\Data\SUN\SUN 2023-01-20 21-08-22\OD3-10-20.M
Last changed : 15/8/2022 10:27:52 pm by SYSTEM
Analysis Method : C:\Users\Public\Documents\ChemStation\1\Data\SUN\SUN 2023-01-20 21-08-22\OD3-10-20.M (Sequence Method)
Last changed : 31/8/2023 10:30:43 pm by SYSTEM
(modified after loading)
Additional Info : Peak(s) manually integrated

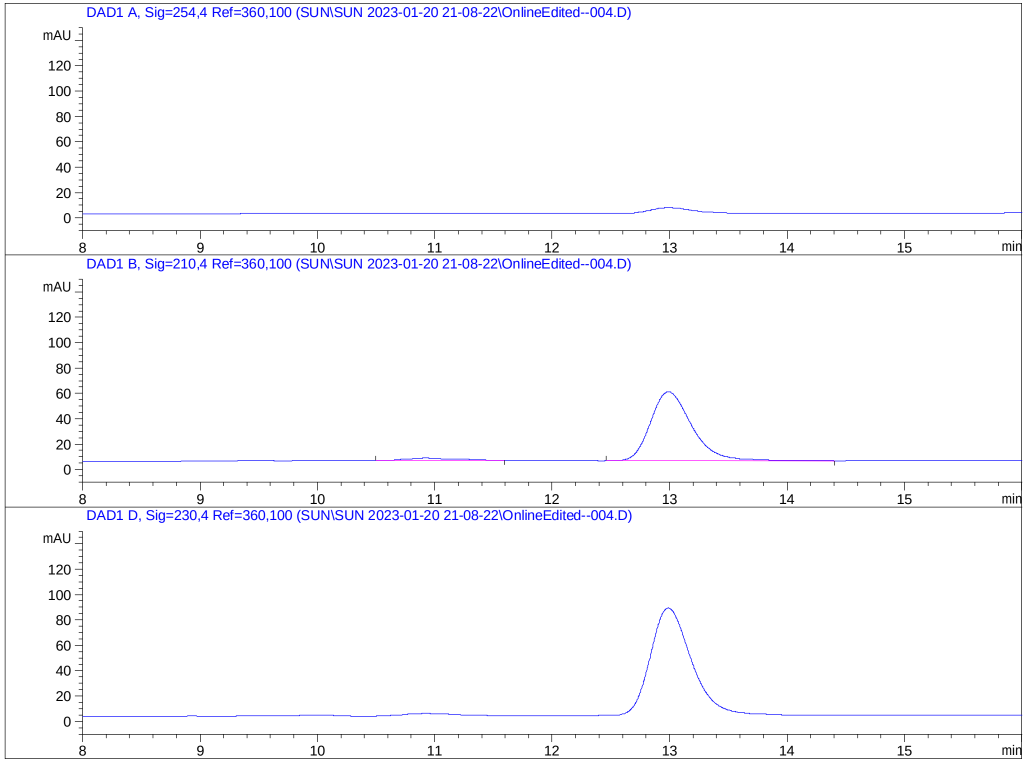

Data File C:\Users\P...\ChemStation\1\Data\SUN\SUN 2023-01-20 21-08-22\OnlineEdited--004.D
Sample Name: zmh-2-124-chiral

===

Area Percent Report

===

Sorted By : Signal
Multiplier : 1.0000
Dilution : 1.0000
Use Multiplier & Dilution Factor with ISTDs

Signal 1: DAD1 A, Sig=254,4 Ref=360,100

Signal 2: DAD1 B, Sig=210,4 Ref=360,100

Peak #	RetTime [min]	Type	Width [min]	Area [mAU*s]	Height [mAU]	Area %
1	10.927	BB	0.3397	49.15391	1.70059	3.5519
2	12.989	BB	0.3787	1334.70776	54.40072	96.4481

Totals : 1383.86168 56.10132

Signal 3: DAD1 D, Sig=230,4 Ref=360,100

===

*** End of Report ***

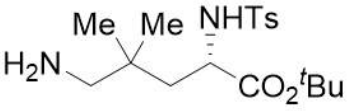

12
enantioenriched

===

Acq. Operator : SYSTEM   Seq. Line : 22
Sample Operator : SYSTEM
Acq. Instrument : HPLC   Location : P1-A-01
Injection Date : 20/1/2023 10:11:02 am   Inj : 1
Inj Volume : 2.000 µl
Different Inj Volume from Sample Entry! Actual Inj Volume : 5.000 µl
Acq. Method : C:\Users\Public\Documents\ChemStation\1\Data\SUN\SUN 2023-01-19 19-04-46 \IA3-10-20.M
Last changed : 15/8/2022 10:26:10 pm by SYSTEM
Analysis Method : C:\Users\Public\Documents\ChemStation\1\Data\SUN\SUN 2023-01-19 19-04-46 \IA3-10-20.M (Sequence Method)
Last changed : 5/9/2023 7:59:14 pm by SYSTEM
(modified after loading)
Additional Info : Peak(s) manually integrated

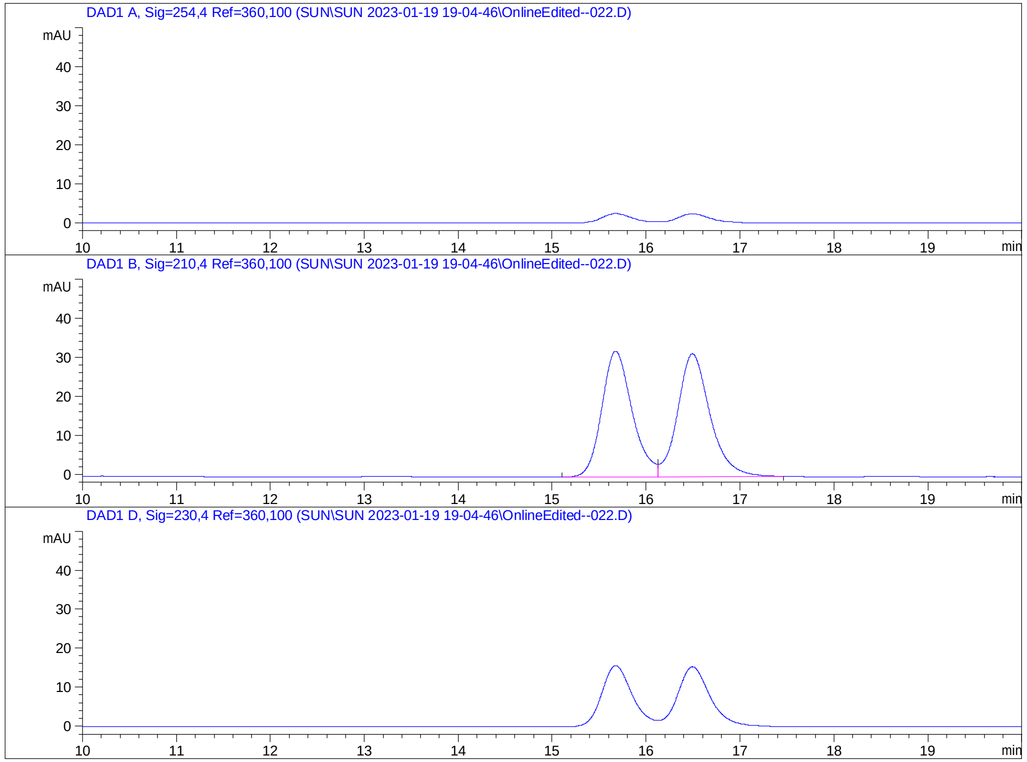

===

Area Percent Report

===

Sorted By : Signal
Multiplier : 1.0000
Dilution : 1.0000
Use Multiplier & Dilution Factor with ISTDs

Signal 1: DAD1 A, Sig=254,4 Ref=360,100

Signal 2: DAD1 B, Sig=210,4 Ref=360,100

Peak #	RetTime [min]	Type	Width [min]	Area [mAU*s]	Height [mAU]	Area %
1	15.676	BV	0.3333	728.92450	32.23090	49.0534
2	16.494	VB	0.3522	757.05573	31.52147	50.9466
Totals :				1485.98022	63.75237	

Signal 3: DAD1 D, Sig=230,4 Ref=360,100

===

*** End of Report ***

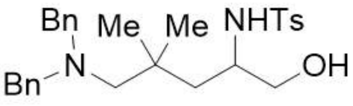

13
racemic

Data File C:\Users\P...Documents\ChemStation\1\Data\SUN\SUN 2023-01-20 21-08-22\1AC-0201.D
Sample Name: zmh-2-123-chiral

===

Acq. Operator : SYSTEM Seq. Line : 2
Sample Operator : SYSTEM
Acq. Instrument : HPLC Location : P1-A-03
Injection Date : 20/1/2023 9:21:06 pm Inj : 1
Inj Volume : 2.000 µl
Different Inj Volume from Sample Entry! Actual Inj Volume : 10.000 µl
Acq. Method : C:\Users\Public\Documents\ChemStation\1\Data\SUN\SUN 2023-01-20 21-08-22\IA3-10-30.M
Last changed : 19/8/2022 10:21:30 pm by SYSTEM
Analysis Method : C:\Users\Public\Documents\ChemStation\1\Data\SUN\SUN 2023-01-20 21-08-22\IA3-10-30.M (Sequence Method)
Last changed : 31/8/2023 10:36:13 pm by SYSTEM
(modified after loading)
Additional Info : Peak(s) manually integrated

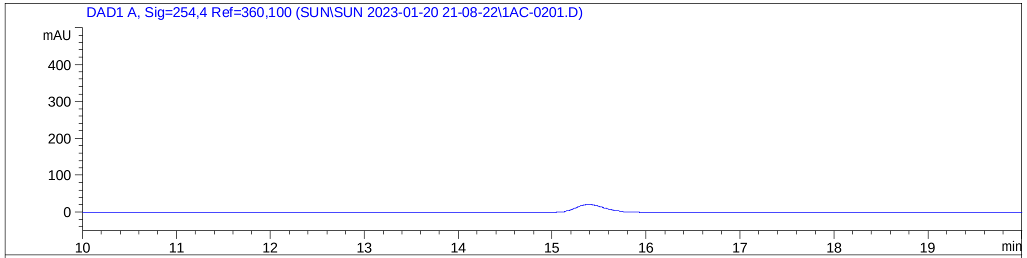

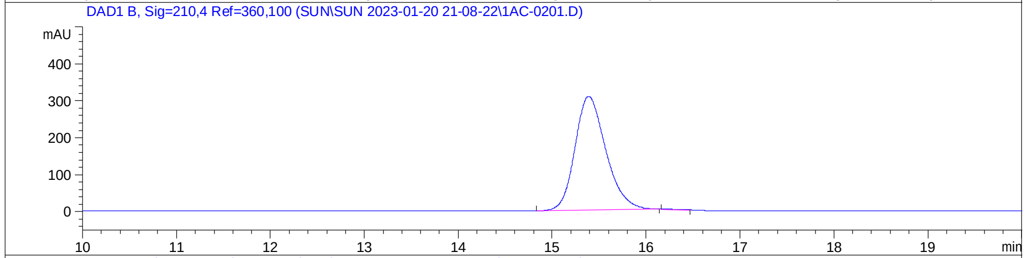

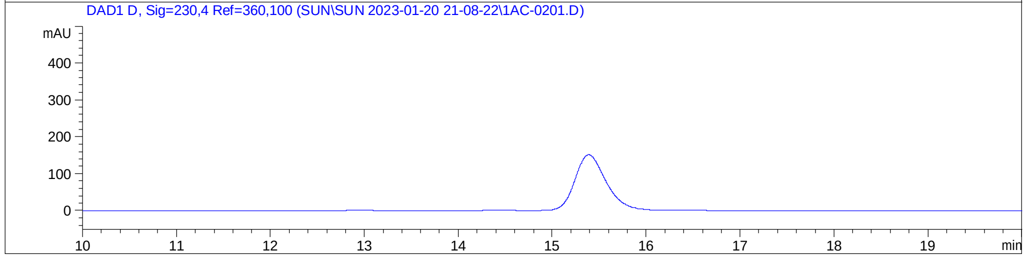

Data File C:\Users\P...Documents\ChemStation\1\Data\SUN\SUN 2023-01-20 21-08-22\1AC-0201.D
Sample Name: zmh-2-123-chiral

===

Area Percent Report

===

Sorted By : Signal
Multiplier : 1.0000
Dilution : 1.0000
Use Multiplier & Dilution Factor with ISTDs

Signal 1: DAD1 A, Sig=254,4 Ref=360,100

Signal 2: DAD1 B, Sig=210,4 Ref=360,100

Peak #	RetTime [min]	Type	Width [min]	Area [mAU*s]	Height [mAU]	Area %
1	15.387	BB	0.3487	7015.42773	307.44788	99.7430
2	16.301	MM	0.2290	18.07651	1.31542	0.2570

Totals : 7033.50424 308.76329

Signal 3: DAD1 D, Sig=230,4 Ref=360,100

===

*** End of Report ***

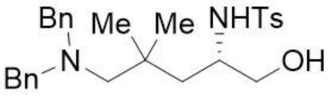

13

enantioenriched

===

Acq. Operator : SYSTEM   Seq. Line : 4
Sample Operator : SYSTEM
Acq. Instrument : HPLC   Location : P1-F-01
Injection Date : 25/5/2024 8:33:29 pm   Inj : 1
Inj Volume : 2.000 µl
Different Inj Volume from Sample Entry! Actual Inj Volume : 10.000 µl
Acq. Method : C:\Users\Public\Documents\ChemStation\1\Data\SUN\SUN 2024-05-25 19-38-12 \AD3-10-30.M
Last changed : 24/10/2022 9:57:21 am by SYSTEM
Analysis Method : C:\Users\Public\Documents\ChemStation\1\Data\SUN\SUN 2024-05-25 19-38-12 \AD3-10-30.M (Sequence Method)
Last changed : 29/6/2024 4:51:49 pm by SYSTEM
(modified after loading)
Additional Info : Peak(s) manually integrated

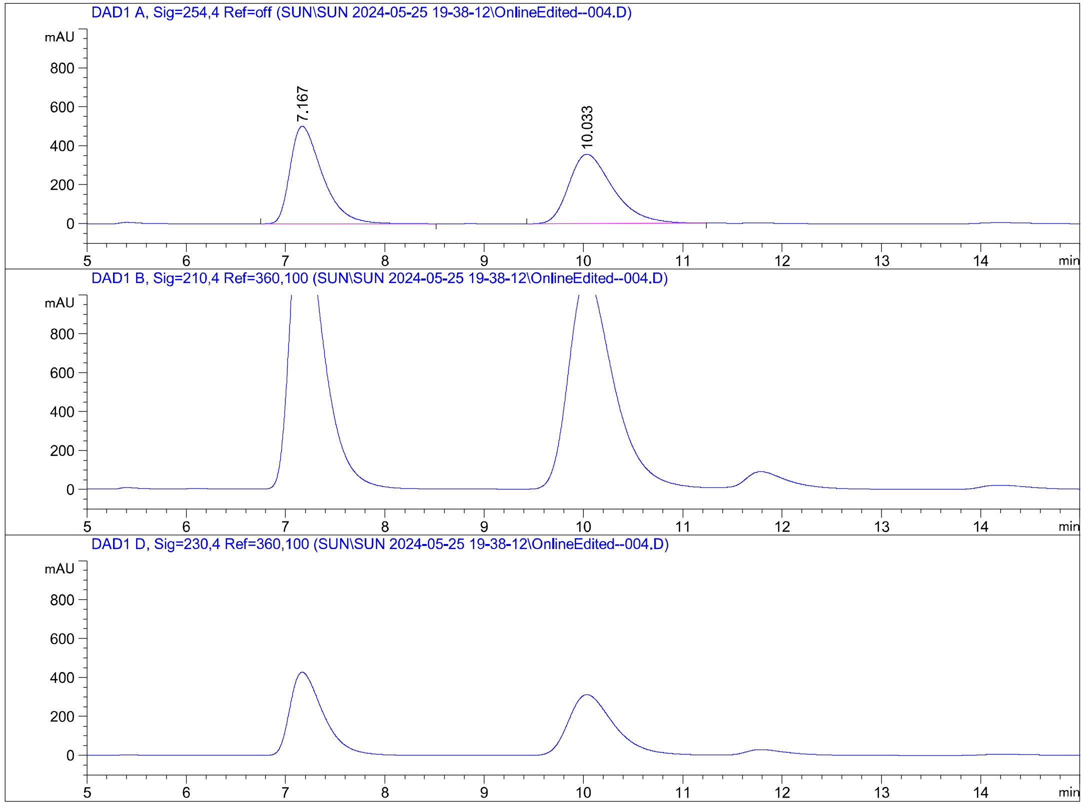

Data File C:\Users\P...\ChemStation\1\Data\SUN\SUN 2024-05-25 19-38-12\OnlineEdited--004.D
Sample Name: zmh-6-3-rac

===

Area Percent Report

===

Sorted By : Signal
Multiplier : 1.0000
Dilution : 1.0000
Use Multiplier & Dilution Factor with ISTDs

Signal 1: DAD1 A, Sig=254,4 Ref=off

Peak #	RetTime [min]	Type	Width [min]	Area [mAU*s]	Height [mAU]	Area %
1	7.167	BB	0.3422	1.12792e4	499.95630	50.6098
2	10.033	BB	0.4696	1.10074e4	355.64810	49.3902

Totals : 2.22866e4 855.60440

Signal 2: DAD1 B, Sig=210,4 Ref=360,100

Signal 3: DAD1 D, Sig=230,4 Ref=360,100

===

*** End of Report ***

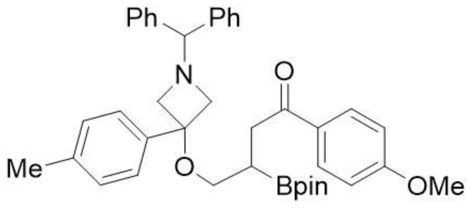

INT'
racemic

===

Acq. Operator : SYSTEM Seq. Line : 2
Sample Operator : SYSTEM
Acq. Instrument : HPLC Location : P1-A-01
Injection Date : 14/6/2024 1:33:14 pm Inj : 1
Inj Volume : 2.000 µl
Different Inj Volume from Sample Entry! Actual Inj Volume : 10.000 µl
Acq. Method : C:\Users\Public\Documents\ChemStation\1\Data\SUN\SUN 2024-06-14 13-16-06 \AD3-10-20.M
Last changed : 15/8/2022 10:21:32 pm by SYSTEM
Analysis Method : C:\Users\Public\Documents\ChemStation\1\Data\SUN\SUN 2024-06-14 13-16-06 \AD3-10-20.M (Sequence Method)
Last changed : 29/6/2024 4:55:35 pm by SYSTEM
(modified after loading)
Additional Info : Peak(s) manually integrated

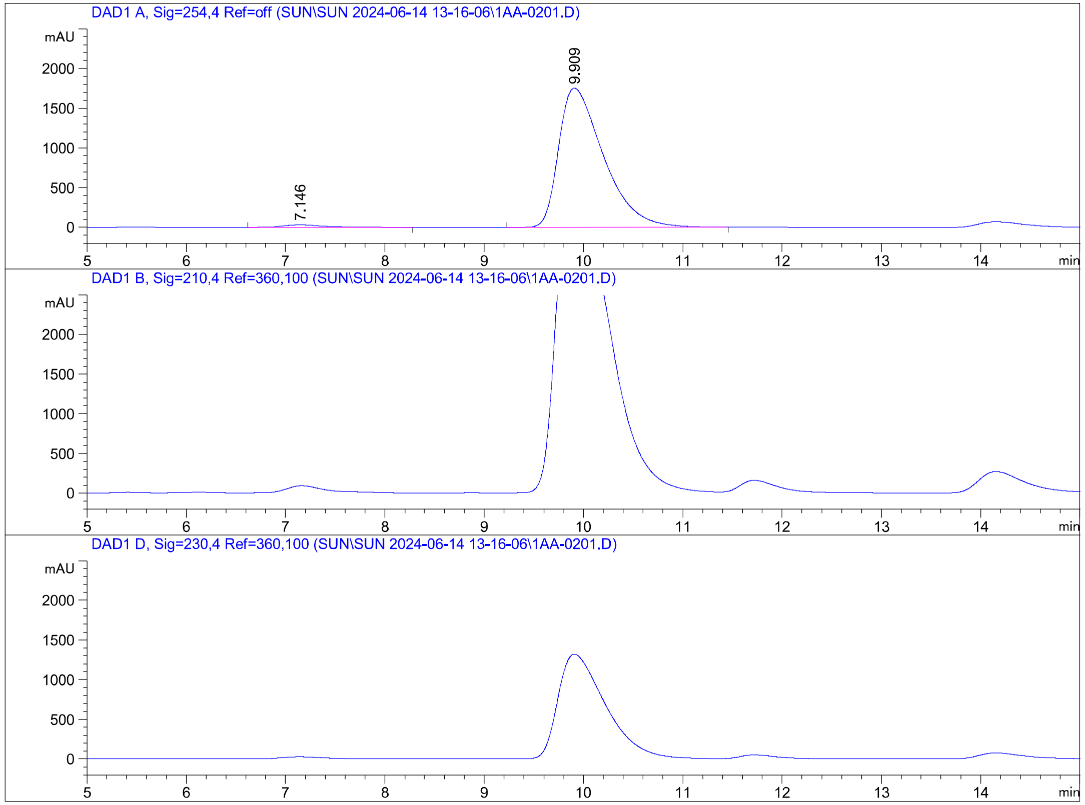

Data File C:\Users\P...Documents\ChemStation\1\Data\SUN\SUN 2024-06-14 13-16-06\1AA-0201.D
Sample Name: zmh-6-3-chiral-S

===

Area Percent Report

===

Sorted By : Signal
Multiplier : 1.0000
Dilution : 1.0000
Use Multiplier & Dilution Factor with ISTDs

Signal 1: DAD1 A, Sig=254,4 Ref=off

Peak #	RetTime [min]	Type	Width [min]	Area [mAU*s]	Height [mAU]	Area %
1	7.146	BB	0.4106	919.66858	31.18422	1.6444
2	9.909	BB	0.4606	5.50083e4	1752.83179	98.3556

Totals : 5.59280e4 1784.01600

Signal 2: DAD1 B, Sig=210,4 Ref=360,100

Signal 3: DAD1 D, Sig=230,4 Ref=360,100

===

*** End of Report ***

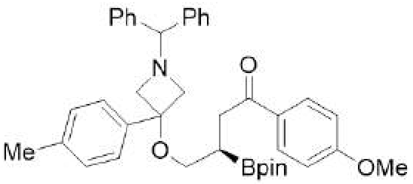

INT'
enantioenriched
